# Backbone-Determined Antiarrhythmic Structure–Activity Relationships for a Mirror Image, Oligomeric Depsipeptide Natural Product

**DOI:** 10.1021/acs.jmedchem.4c00923

**Published:** 2024-07-03

**Authors:** Madelaine P. Thorpe, Daniel J. Blackwell, Bjorn C. Knollmann, Jeffrey N. Johnston

**Affiliations:** † Department of Chemistry and Vanderbilt Institute of Chemical Biology, Vanderbilt University, Nashville, Tennessee 37235, United States; ‡ Department of Medicine, Vanderbilt University Medical Center, Nashville, Tennessee 37235, United States

## Abstract

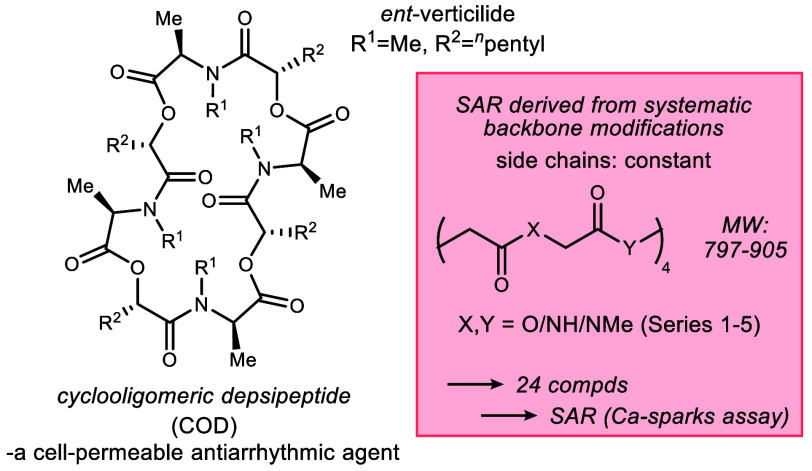

Cyclic oligomeric depsipeptides (COD) are a structural class within naturally occurring compounds with a wide range of biological activity. Verticilide is a COD (24-membered ring) that was identified by its inhibition of insect ryanodine receptor (RyR). We have since found that the enantiomer of verticilide (*ent*-verticilide, **1**) is a potent inhibitor of mammalian RyR2, a cardiac calcium channel, and therefore a potential antiarrhythmic agent. Oddly, *nat*-verticilide does not inhibit RyR2. To further develop *ent*-verticilide as an antiarrhythmic, we explored potential SAR through systematic modification of the ester’s functionality to both *N*–H and *N*–Me amides. The syntheses of these *ent*-verticilide-inspired analogs are detailed using a monomer-based platform enabled by enantioselective catalysis. Two analogs among 23 exhibited measurable reduction of calcium sparks in a functional assay of RyR2 activity. These findings illustrate the value of natural product-inspired therapeutic development, but the less-studied approach where the non-natural enantiomeric series harbors important SAR.

## Introduction

The natural product verticilide A1 was discovered by Omura in 2004^1^ and reported in 2006^2^ to have inhibitory activity against insect ryanodine receptor (RyR). In 2019, we reported that its non-natural enantiomer (**1**) exhibits activity at mammalian ryanodine receptor 2 (RyR2), while *nat*-verticilide was inactive, recording an evidently unprecedented case where all detectable activity against a specific target is harbored by the non-natural enantiomer of a natural product.^3,4^ RyR2 is a target for antiarrhythmic development, and selective inhibitors promise to develop a more complete picture of its role in heart disease.^5−7^ We have since discovered a second example of activity harbored entirely by the non-natural enantiomer in the antiarrhythmic *ent*-verticilide B1—the 18-membered oligomer of **1**.^8^ These discoveries provide a basis for the isoform-selective RyR inhibitors recently described, collectively spanning a broad range of molecular size and structure.^9−11^ Moreover, their non-natural configuration could lead to favorable pharmacokinetics and pharmacodynamics.^12^

**Figure 1:**
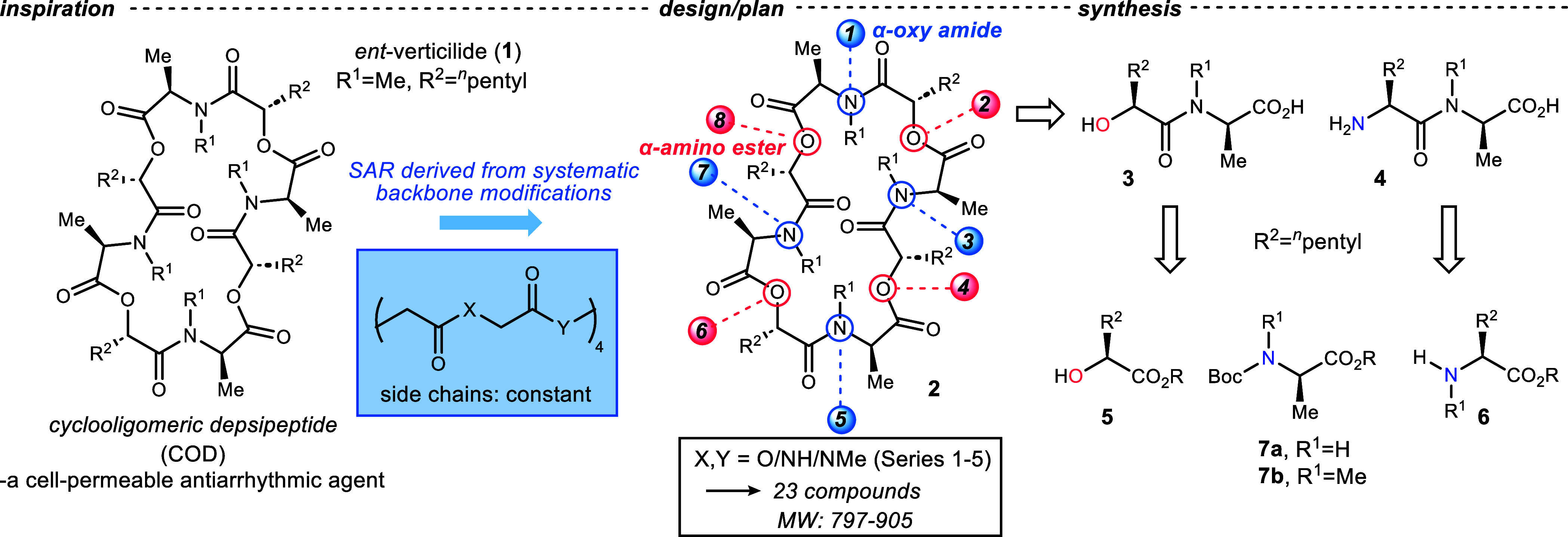
*ent*-Verticilide cyclooligomeric depsipeptide structure, backbone modification plan, and key retrosynthetic modules for the determination of the effect of backbone modifications on antiarrhythmic activity.

The verticilides are cyclic depsipeptides, and verticilide A1 is a 24-membered cyclic oligomeric depsipeptide (COD) comprised of alternating l-alanine and (*R*)-α-hydroxy heptanoic acid residues. The surprising activity of *ent*-verticilide is accented by its Beyond Rule of 5 (bRo5) classification, and its oligomeric nature suggests that the pharmacophore may be a fraction of the molecule’s total size. We have previously reported activity at RyR2 as a function of macrocycle size by examining COD ranging in ring size from 6 to 36, where the nature of the polar backbone is modified only by depsipeptide chain length.^13^ In this study, the aliphatic side chains thought to drive target engagement are held constant, while modifications to the polar backbone are explored, in order to determine the degree to which the backbone structure and functionality contribute to antiarrhythmic activity. The cyclic oligomeric nature of **1** lends itself to the systematic substitution of ester for *N*–H and *N*–Me amide (Figure 1, Series 1–5). This includes a few oligomeric analogs, but mostly analogs that require construction from didepsipeptide (**3**) or dipeptide (**4**) building blocks. The didepsi/peptides **3**–**4** simplify further to α-hydroxy acid **5** and N-protected α-amino acids **6** and **7**. While **7** was available commercially, both **5** and **6** required preparations by enantioselective synthesis. Overall, a collection of 23 compounds was prepared based on the *ent*-verticilide structure. Key to this approach was preservation of the methyl and pentyl side chains across all backbone modifications to isolate the contribution of backbone functionality to activity, either directly by contact (e.g., hydrogen bonding) or indirectly (e.g., conformational effects, permeability^14^).

To date, *ent*-verticilide is not known to be produced by a natural source, but three distinct syntheses by us and two by Omura and Sunazuka (*nat*-verticilide)^2a,2c^ provide a robust platform for the study of this natural product class, including the non-natural enantiomers described here. The first synthesis of *nat*-verticilide was reported by Omura in 2006 using a chiral auxiliary to establish the (*R*)-configuration of the heptanoic acid residue needed for *nat*-verticilide.^2^ We subsequently reported the use of an enantioselective Henry reaction to prepare the epimeric residue in order to use a Mitsunobu-based macrocyclooligomerization for *nat*-verticilide synthesis.^15,16^ This Henry reaction is equally effective for (*R*)- or (*S*)-α-hydroxy heptanoic acid residue preparation, and it has been used in our later preparations of *ent*-verticilide at larger scales.^12,17^

**Figure 2:**
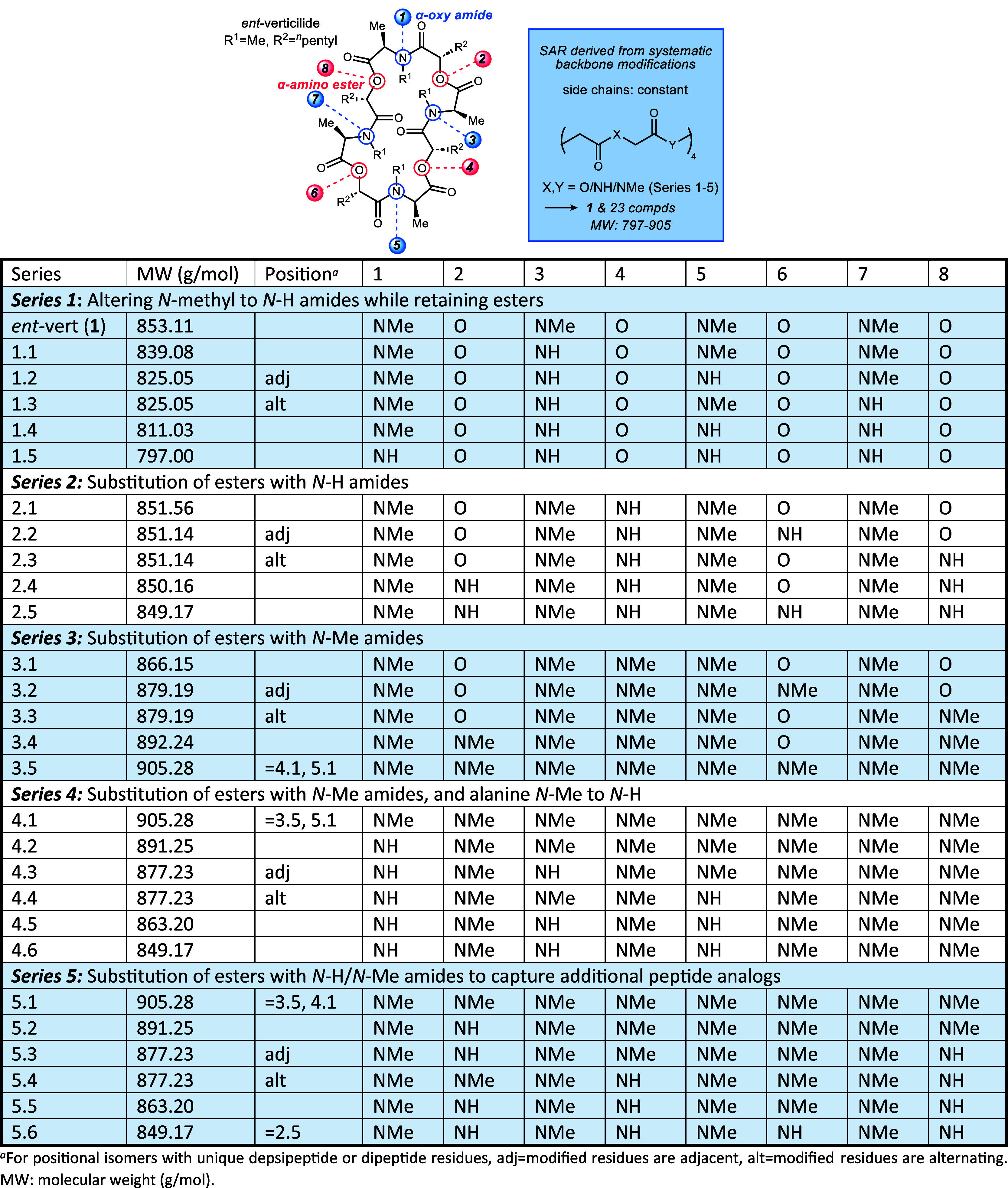
Series 1–5 analogs to probe contributions of the macrocycle backbone to antiarrhythmic activity.

Figure 2 summarizes five series of analogs (Series 1–5). Series 1 holds the ester functionality constant while converting each *N*–Me amide to *N*–H amide. Series 2 systematically substitutes each ester with *N*–H amide, while Series 3 similarly substitutes each ester with *N*–Me amide. Series 4 substitutes all esters in **1** with *N*–Me amide while systematically alternating the alanine *N*–Me amides to *N*–H amides. Series 5 is analogous to Series 4, except all esters are first substituted by *N*–Me amides, and then the *N*–Me alanines are systematically replaced by *N*–H alanines. In each series, there is a pair of adjacent and alternating oligomer isomers that offer an opportunity to examine the effect of symmetry on activity.

The analogs in Series 1–5 vary in complexity, varying in the number of distinct monomers required for synthesis. Since this analog campaign required (*S*)-α-hydroxy heptanoic acid ester and amide preparation (e.g., **3**), we chose to exploit the versatility of the Henry products, expecting that the nitroalkanes might bifurcate readily to a carboxylic acid^18^ for conventional ester and amide synthesis or be used directly to amide products (Figure 1).^19,20^ The development of a robust enantioselective synthesis of (*S*)-α-hydroxy acid was a critical centerpiece and its use to prepare α-hydroxy benzyl ester **5**, *N*–H amide (**3a**), and *N*–Me amide (**3b**). An enantioselective synthesis of α-amino ester **7** for both *N*–H and *N*–Me functionality was equally critical. Successful incorporation of these monomers into a sound synthesis scheme to prepare all members of Series 1–5 to drive a comprehensive study of their antiarrhythmic structure–activity relationships has now been accomplished (Figure 2, Series 1–5).

## Results and Discussion

### Monomer (M)^21^ Synthesis

**Scheme 1:**
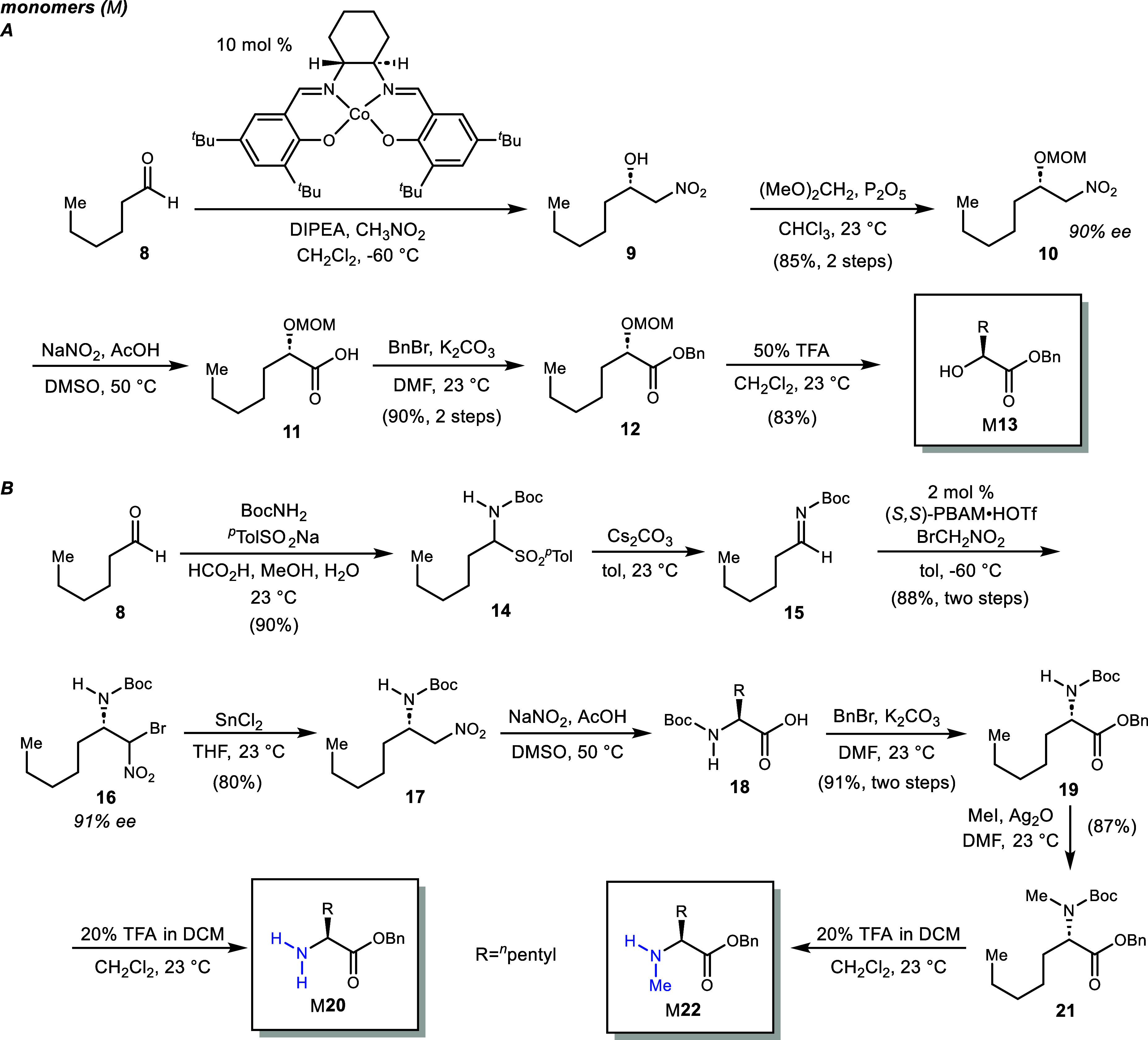
Enantioselective Synthesis of α-Hydroxy Ester and α-Amino Esters for *ent*-Verticilide Analog Synthesis

Beginning from hexanal (**8**), α-hydroxy ester M**13** (**5**, R = Bn) was prepared by enantioselective Henry addition of nitromethane and immediate transacetalization with dimethoxy methane to give terminal nitroalkane **10** in 90% ee and 85% yield over 2 steps.^16^ The (*S*)-enantiomer was prepared using the cobalt(II)-salen ligand complex, as described by Yamada.^22^ Treatment of nitroalkane **10** using the conditions outlined by Mioskowski^18^ produced the terminal carboxylic acid (**11**) which was immediately esterified to **12** in 90% yield (2 steps) under mildly basic conditions. Deprotection of the acetal using trifluoroacetic acid yielded α-hydroxy ester M**13** in 83% yield. Hexanal was also the starting point for α-amino esters M**20** and M**22** (**7**, R = Bn). Conversion of hexanal to α-amido sulfone **14**(23) preceded elimination to the N-Boc imine substrate (**15**)^23b^ for the aza-Henry reaction.^24^ Catalyzed addition of bromonitromethane^25^ using the chiral proton catalyst (*S*,*S*)-PBAM·HOTf^26^ furnished β-amino nitroalkane **16** in 91% ee as a 1:1 mixture of diastereomers. These diastereomers converged to a single terminal nitroalkane (**17**) in 90% ee upon treatment with stannous chloride. This two-step procedure using bromonitromethane provided high overall yields relative to the use of nitromethane in a single-step procedure to prepare **17** from **15**.^27^ Conversion of nitroalkane **17** to acid **18** using the Mioskowski-Nef reaction^18^ was followed by base-promoted esterification to give **19** and then M**20** after acid treatment with TFA to remove the N-Boc protecting group. Similarly, methylation of the N-Boc group gave **21** from **19**,^28^ which was also subjected to acid treatment to give α-amino ester M**22** for analogs containing N–Me amide(s). Monomer M**13** was routinely prepared at 16 g-scale and M**20** and M**22** at 5 g-scale (Scheme 1).

### Didepsi/Peptide Synthesis (D)

**Scheme 2:**
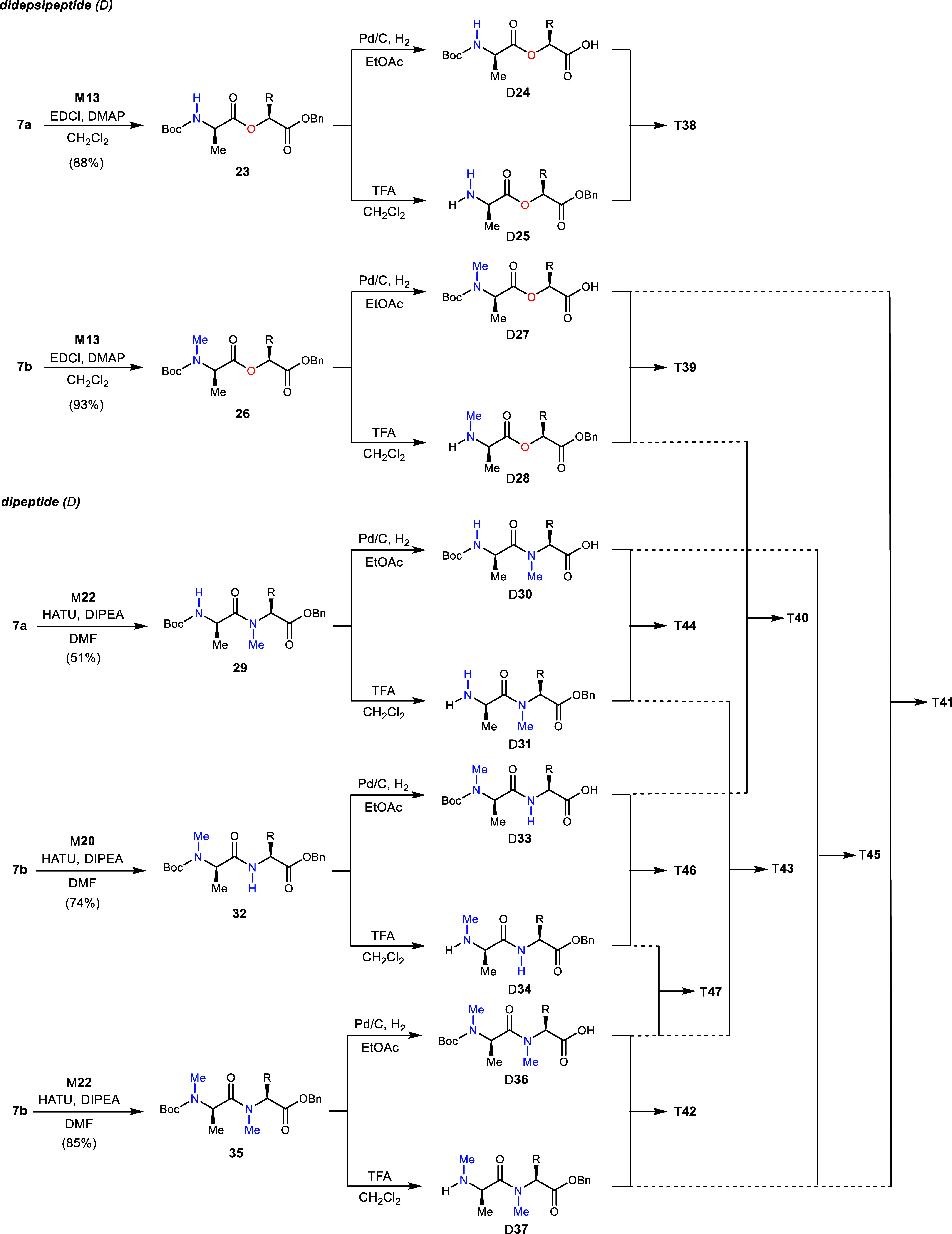
Conversion of Monomers into Didepsipeptide and Dipeptide Donors for Construction of *ent*-Verticilide Analogs

**Scheme 3:**
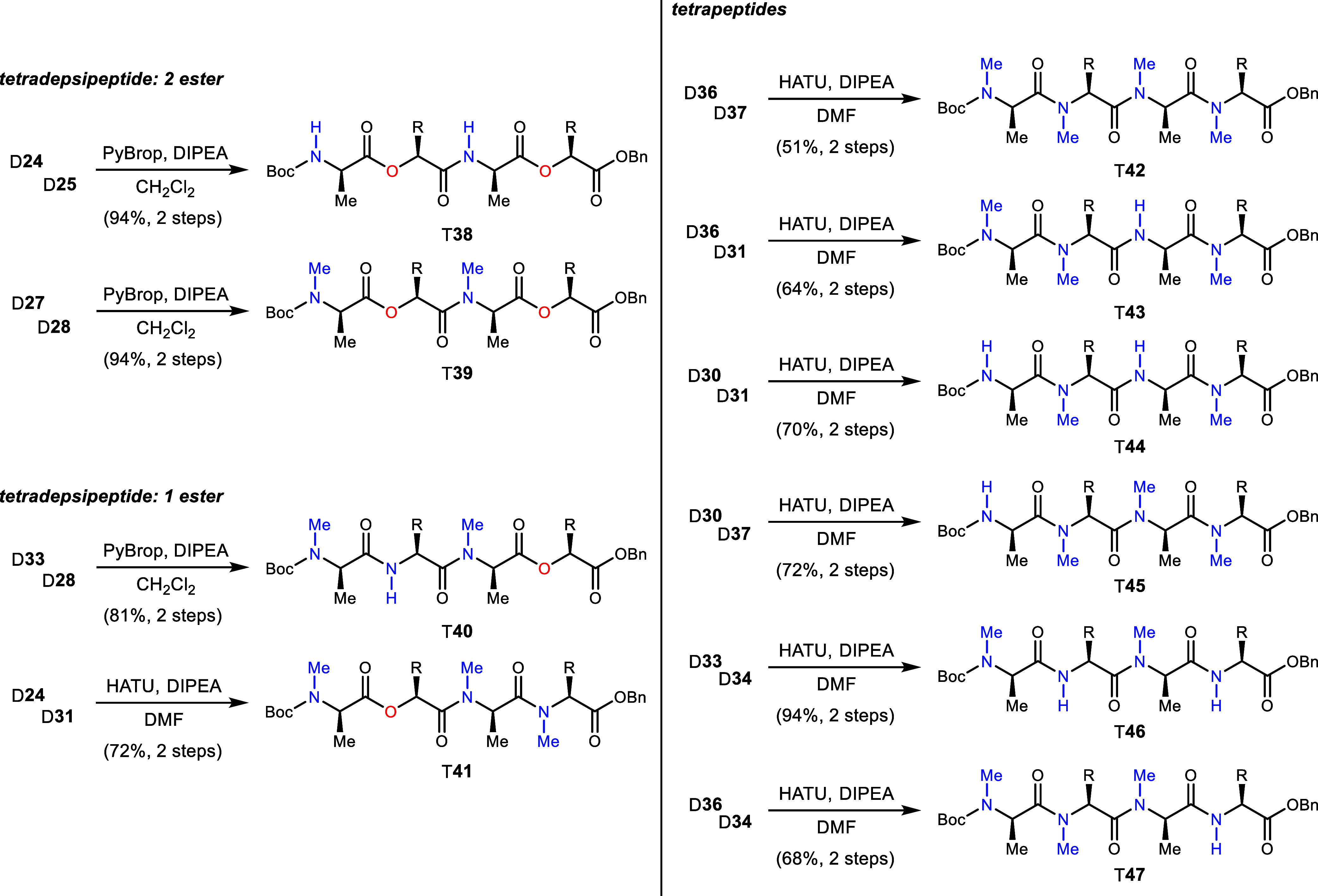
Construction of Tetradepsipeptide and Tetrapeptide Units for Efficient Construction of Series 1–5 Analogs Using Didepsipeptide and Dipeptide Precursors

Depsipeptides **23** and **26** were identified as common components needed for Series 1–3 (Scheme 2). Didepsipeptide **23** was prepared from α-hydroxy ester M**13** and N-Boc-protected d-alanine using EDCI^29^ and DMAP, in 88% yield.^13^ The preparation of N–Me didepsipeptide **26** followed the same procedure and was prepared in 93% yield. Amounts of didepsipeptides **23** and **26** could be converted to acids D**24** and D**27**, respectively, by hydrogenolysis. In parallel, deprotection to the terminal amines D**25** and D**28** was accomplished by standard treatment with TFA. Without purification, these units were coupled as needed to build toward the octadepsipeptides. A similar approach was used to prepare dipeptide units (Scheme 2), with dipeptide **29** formed by a coupling of **7a** with M**22** in 51% yield using HATU and DIPEA in DMF. N–Me dipeptides **32** and **35** were formed from **7b** using identical conditions. Also, analogous was the bifurcation of each doubly protected dipeptide into C-deprotected dipeptides D**30**, D**33**, and D**36**, as well as N-deprotected dipeptides D**31**, D**34**, and D**37**. Scheme 2 also diagrams the eventual use of each didepsi/peptide in subsequent conversions to tetradepsi/peptides, as detailed in Scheme 3.

### Tetradepsi/Peptide Synthesis (T)

Preparation of a collection of tetradepsipeptides and tetrapeptides was also needed for efficient construction of the final octadepsi/peptides. This was accomplished using the series of dipeptide couplings, as summarized in Scheme 3. Tetradepsipeptides containing two esters were prepared using PyBrop. Didepsipeptides D**24** and D**25** were joined to form T**38**, while D**27** and D**28** coupled to produce T**39** in 94% yield, respectively.^30^ Tetradepsipeptides T**40** and T**41** containing a single ester each were prepared from D**33**/D**28** and D**24**/D**31**, respectively. The latter required a change in coupling reagent from PyBrop to HATU in order to address poor solubility and slow reaction of D**31**.^31,32^ Tetrapeptides were synthesized uniformly using HATU in DMF. D**36** was coupled to N–Me amine D**37** to form T**42** in 51% yield overall. *N*–H amine D**31** was joined with D**36** and D**30** to prepare T**43** and T**44**, respectively, in 64–70% yield. *N*–Me amine D**37** was coupled to D**30** to form T**45** in 72% yield. Finally, D**34** was coupled to both D**33** and D**36** to prepare T**46** and T**47**, respectively, in good overall yield.

### Final Steps of Analog Synthesis (H,O)

**Scheme 4:**
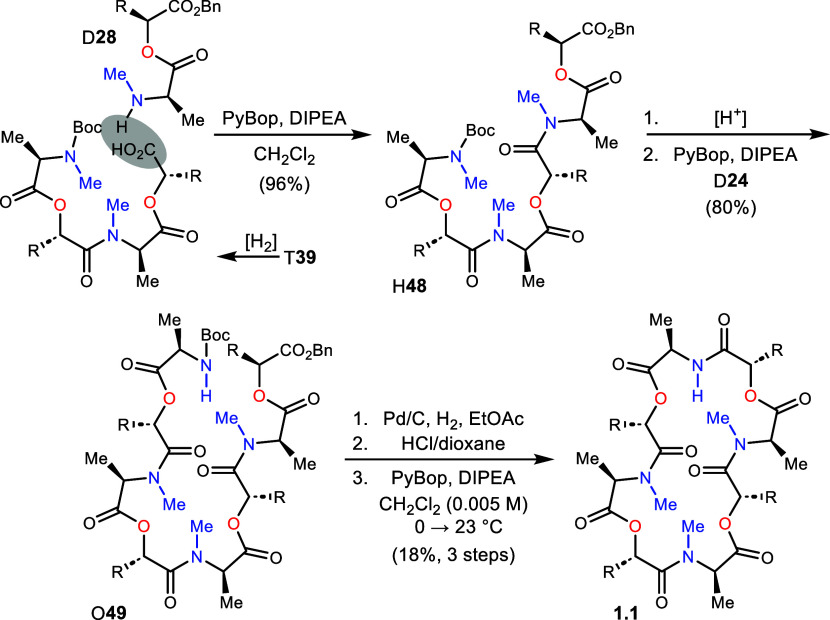
Final Steps to Complete the Synthesis of Analog 1.1

**Scheme 5:**
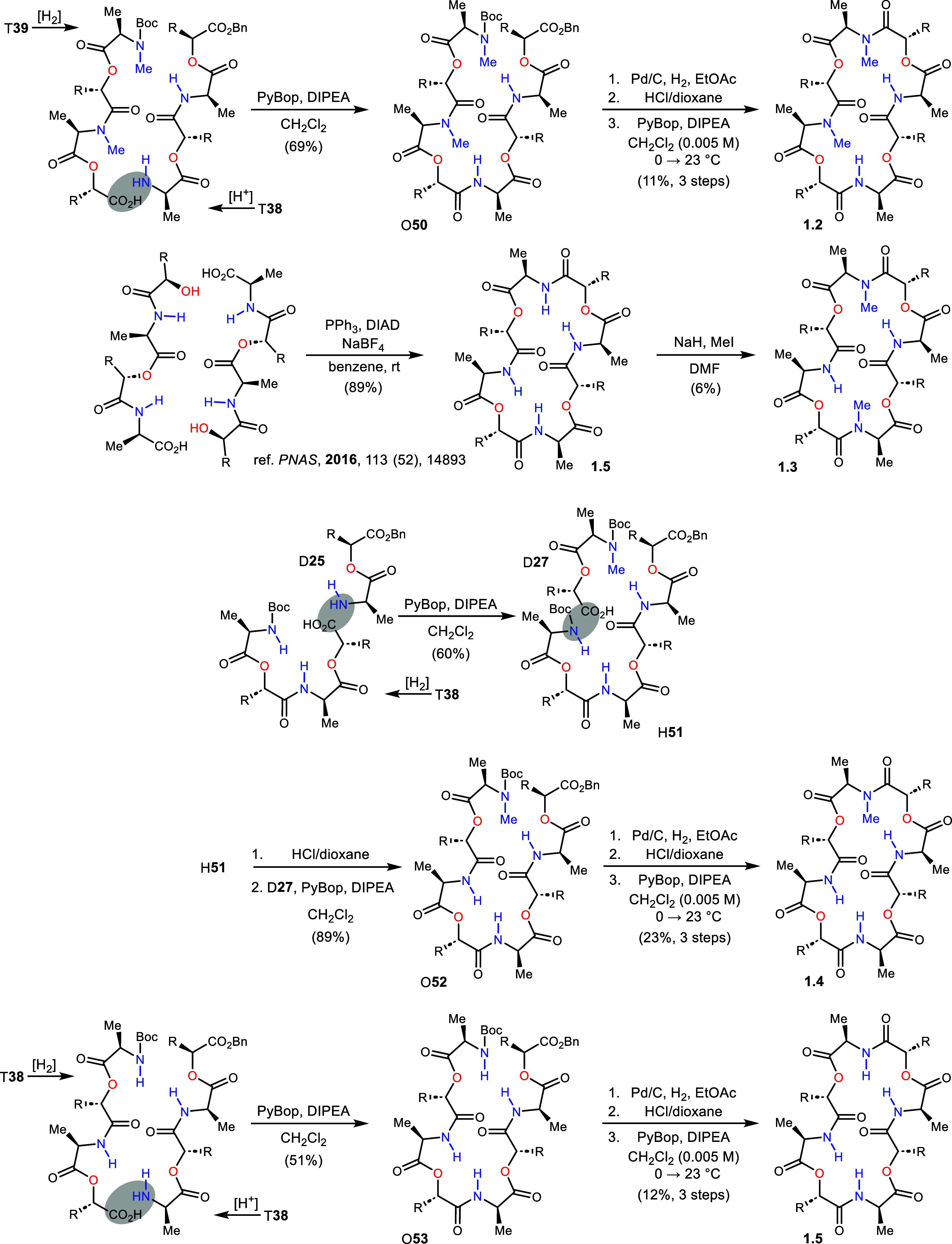
Completion of Analogs **1.2**–**1.5**

The final phase of each analog synthesis varied considerably to account for relative differences in pseudooligomer simplicity. Analog 1.1 was prepared from T**39** (after hydrogenolysis) by extension of the C-terminus with didepsipeptide D**28** in 96% yield (Scheme 4). This hexadepsipeptide (H**48**) was Boc-deprotected and coupled with D**24** to give octadepsipeptide O**49**. The final steps converting O**49** to 1.1 involved hydrogenolysis, Boc-deprotection, and PyBop-mediated macrocyclization at high dilution.^2^ Analog 1.1 was produced in 18% yield (3 steps), after purification by reverse phase HPLC. While 1.1 was prepared using a [4+2]+2 approach to octadepsipeptide O**49**, analog 1.2 used a 4+4 build from T**38** and T**39**, proceeding in 69% yield (Scheme 5). The final steps were identical to those for analog 1.1, furnishing 1.2 in 11% yield (3 steps). Of the five Series 1 analogs, 1.3 alone had been prepared previously.^15^ The approach was quite different, using a Mitsunobu-driven cyclodimerization reaction followed by base-promoted methylation (NaH, MeI). Alternating *N*–Me/*N*–H amide analog 1.3 was produced in only 6% yield, but the original goal (and major product) of this reaction was permethylation. Analog 1.4 proceeded from T**38** and D**25** in a [4+2]+2 build similar to 1.1 (Scheme 5). This led to hexadepsipeptide H**51** in 60% yield. Subsequent N-deprotection with HCl in dioxane and coupling with carboxylic acid D**27** provided O**52** in 89% yield overall. Hydrogenolysis and N-deprotection prepared the octamer for macrocyclization, which proceeded in 23% yield (3 steps) to furnish 1.4 in 23% yield. Depsipeptide 1.5, the final analog in this series, was prepared from tetradepsipeptide T**38**, with half deprotected at the N-terminus and the other at the C-terminus (Scheme 5). These fragments were joined in 51% yield to O**53**. Deprotection at each end, and macrocyclization, provided 1.5 in 12% yield (3 steps).

**Scheme 6:**
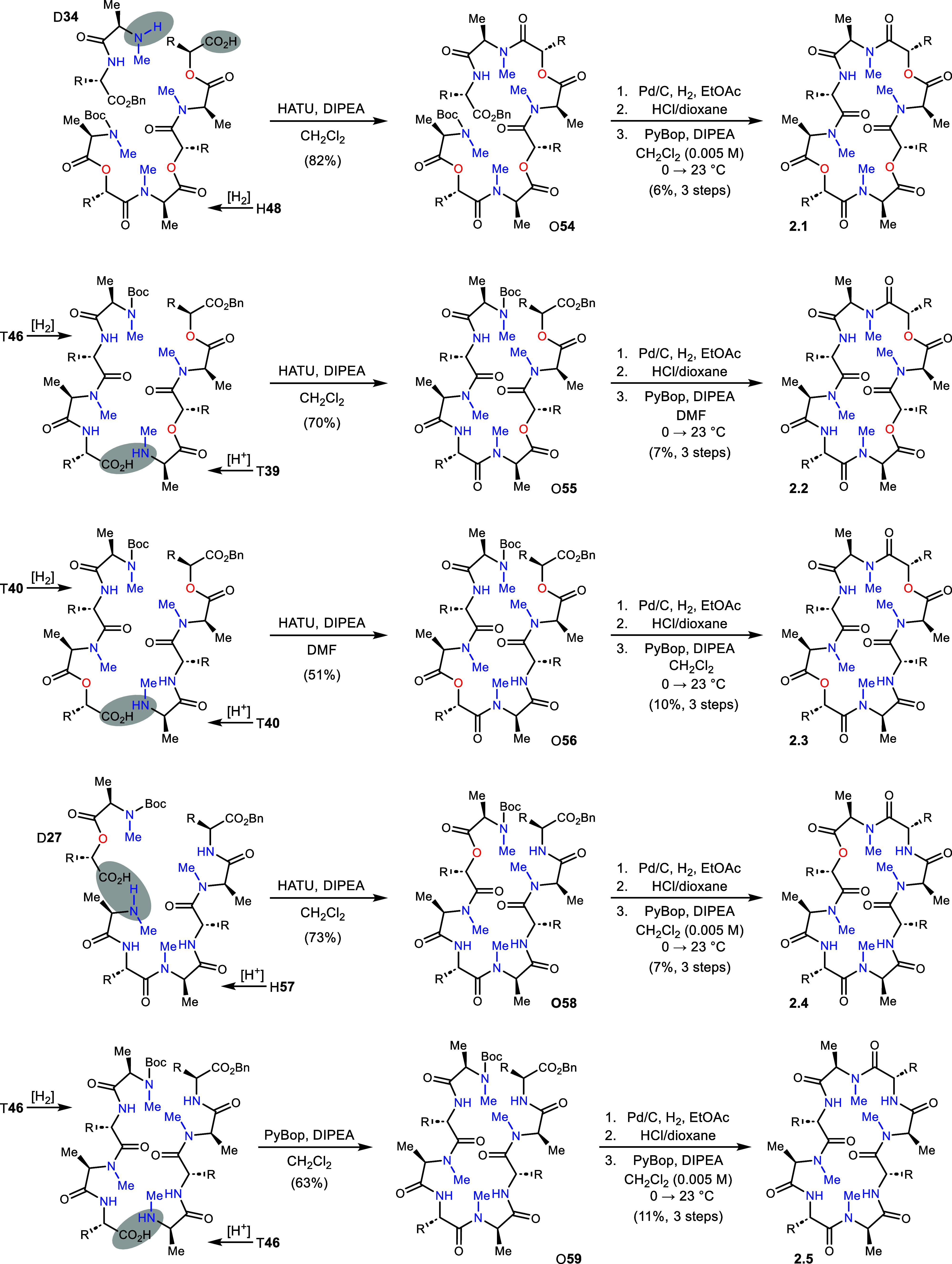
Construction of Series 2 Analogs (**2.1–2.5**)

All analogs in Series 2 were prepared from tetradepsi/peptide precursors in a [4+4] build, except 2.1 and 2.4 which required a [2+6] approach (Scheme 6). Analog 2.1 evolved from H**48** which, after C-deprotection, was coupled to *N*–Me amine D**34** to furnish O**54** in 82% yield. Deprotection of the termini in O**54**, and macrocyclization, provided 2.1 in 6% yield (3 steps).^30^ The low yield was a harbinger of future observations for most macrocyclizations, but reasons were not investigated.^33^ Analog 2.2 was constructed from depsipeptide T**39** and peptide T**46** to give intermediate O**55**. This octadepsipeptide was deprotected and macrocyclized to 2.2 in 7% yield (3 steps). Similarly, analog 2.3 began from tetradepsipeptide T**40** with half C-deprotected, and the other half N-deprotected. The coupling product O**56** was formed in 51% yield but required the solvent DMF for improved solubility. Deprotection at each terminus, and then macrocyclization, provided 2.3 in 10% yield (3 steps). Analog 2.4 was constructed from peptide H**57** and depsipeptide D**27** which were conjoined after deprotection to give depsipeptide O**58** in 70% yield. The standard deprotection and macrocyclization protocols gave 2.4 in 7% yield (3 steps). Analog 2.5 was prepared from peptide T**46** after its bifurcation to N- and C-deprotected fragments and coupling to O**59**. This peptide was deprotected and cyclized to 2.5 in 11% yield (3 steps).

**Scheme 7:**
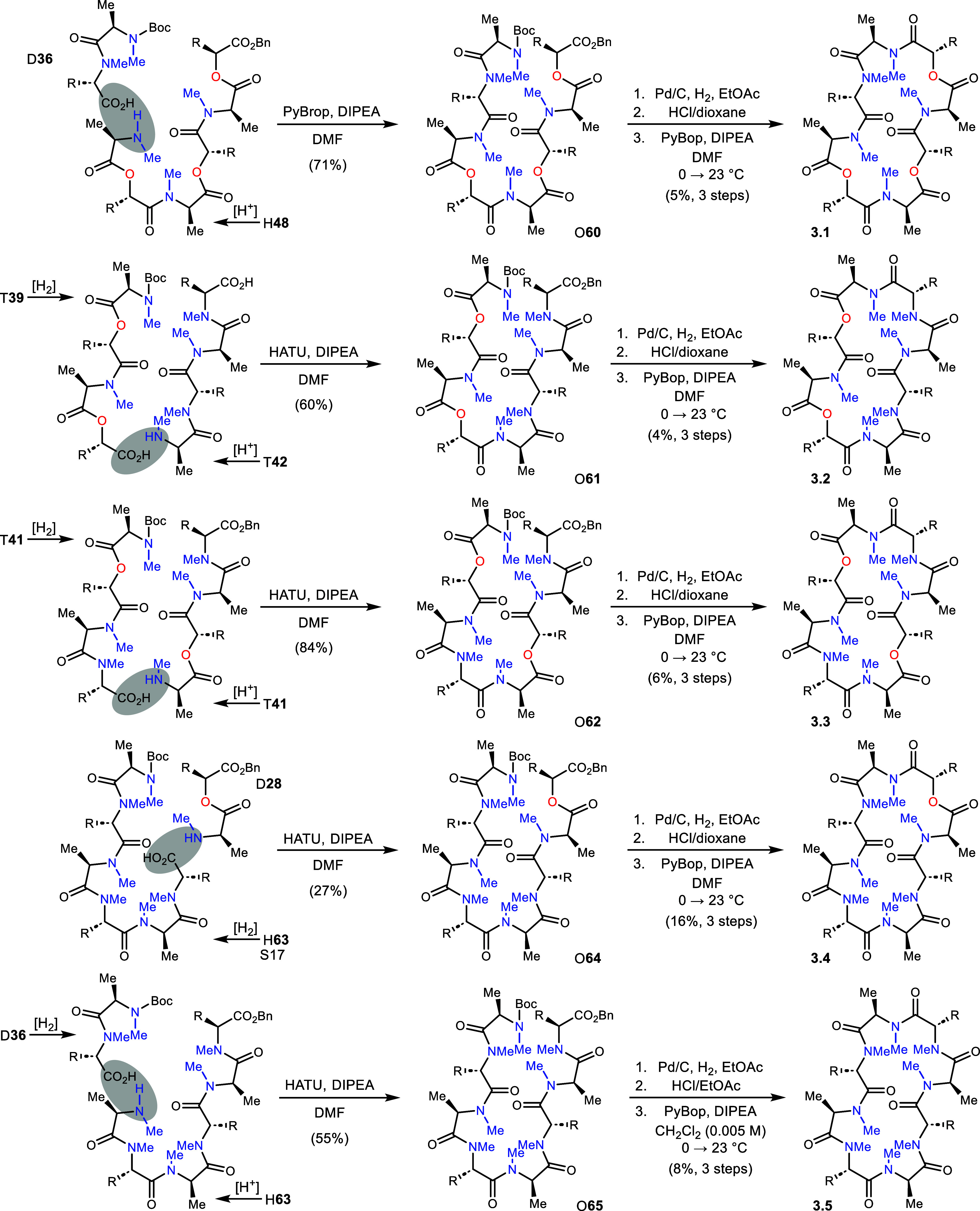
Construction of Series 3 Analogs

Construction of Series 3 analogs followed a similar approach. A [6+2] strategy for 3.1 required the hexadepsipeptide (H**48**) first used in the preparation of analog 1.1 (Scheme 7). After N-deprotection of H**48**, it was coupled with D**36** to form O**60** in 71% yield. Deprotection of the octadepsipeptide and cyclization of the *seco*-amide provided analog 3.1 in 5% yield. For analog 3.2, tetrapeptide T**42** and depsipeptide T**39** were deprotected at N- and C-termini, respectively. The octadepsipeptide O**61** resulting from their coupling (60% yield) was converted to analog 3.2 in a 4% yield (3 steps). Octadepsipeptide O**62** needed to prepare analog 3.3 was synthesized in 84% yield from two units of T**41** using the orthogonal deprotection approach used previously. It was then deprotected and macrocyclized to 3.3 in 6% yield (3 steps). The [6+2] approach was used for analog 3.4, beginning from H**63** and its eventual coupling with D**28**. This maneuver suffered from an unusually low yield (27%) but provided sufficient quantity of octadepsipeptide O**64** for finishing to analog 3.4 (16% yield, 3 steps). To finish Series 3, hexapeptide H**63** was N-deprotected and then coupled with dipeptide D**36** to form octapeptide O**65**. This intermediate was finished along standard lines to give analog 3.5 in 8% yield (3 steps).

**Scheme 8:**
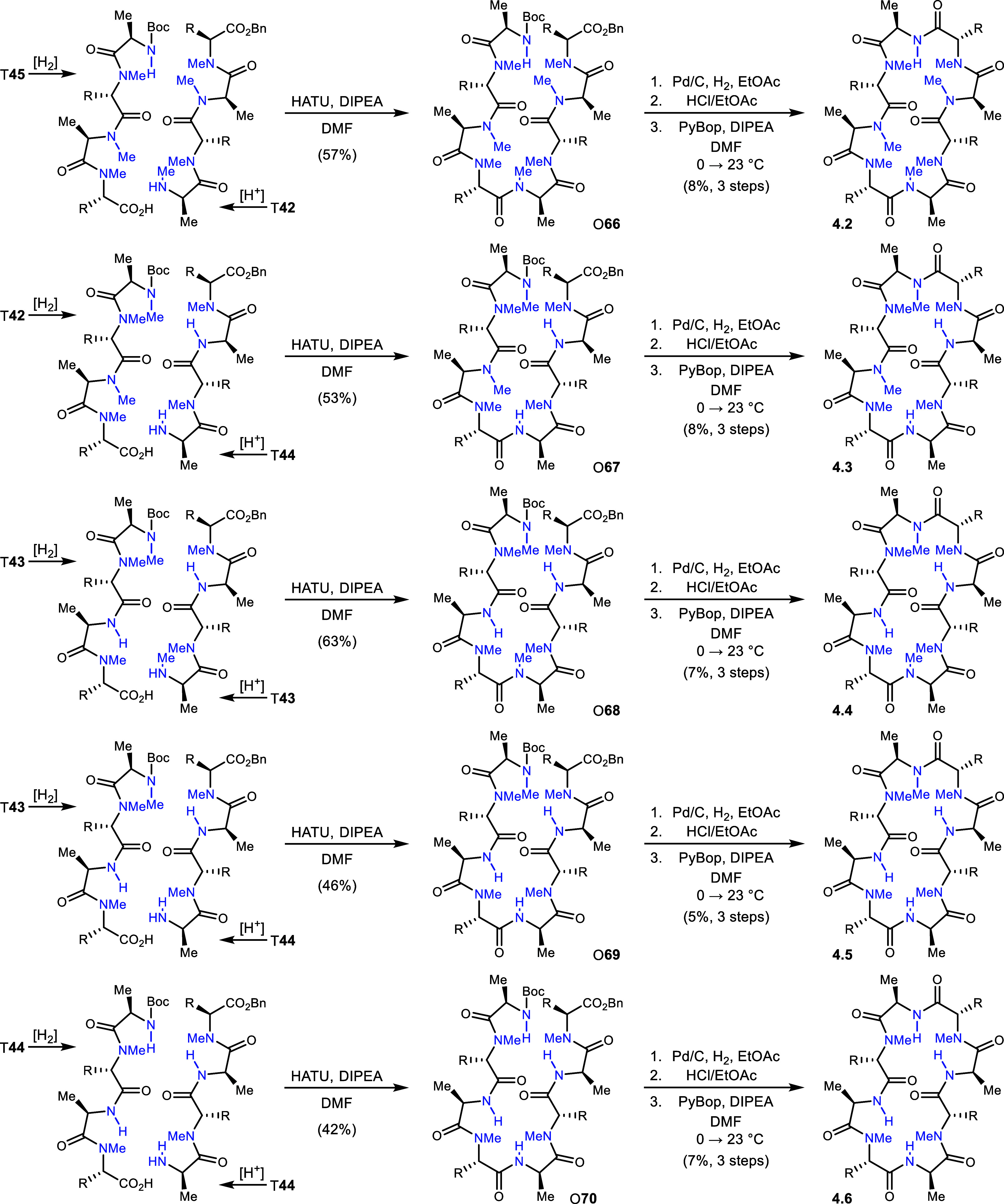
Construction of Series 4 analogs

The first member of Series 4, analog 4.1, is identical to analog 3.5 (and 5.1) whose preparation was described above (Scheme 8). The remaining members were prepared by a [4+4] build. Analog 4.2 was constructed beginning with peptides T**42** and T**45** through a coupling that provided octapeptide O**66** (57% yield). Conversion of O**66** to analog 4.2 leveraged the established three step procedure culminating in 8% yield. Analog 4.3 was sourced from peptides T**44** and T**42**, leading to a coupling yield of 53% to give peptide O**67**. Standard completion steps yielded analog 4.3 in 8% yield (3 steps). Peptide T**43** was dimerized after selective N-deprotection and, separately, C-deprotection. This provided peptide O**68** in 63% yield and ultimately analog 4.4 in 7% yield (3 steps). The penultimate analog in the series began from T**44** (N-deprotected) and T**43** (C-deprotected), providing peptide O**69** in 46% yield. From O**69**, analog 4.5 was formed in 5% yield (3 steps) following the standard sequence of finishing steps. The final Series 4 analog (4.6) used the homopeptide bifurcation approach using T**44**, providing peptide O**70** in 42% yield. The finishing sequence provided analog 4.6 in 7% yield (3 steps).

**Scheme 9:**
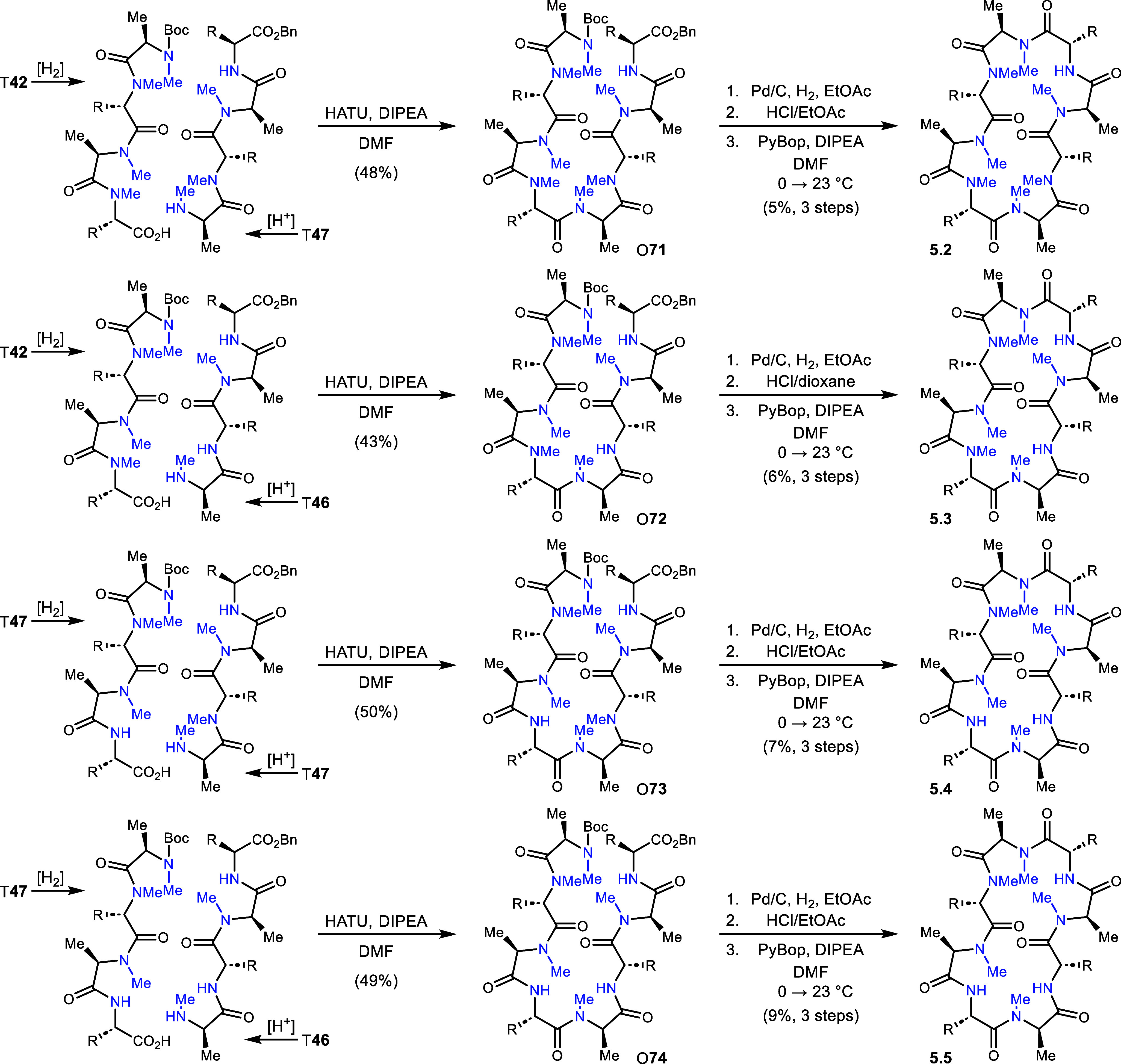
Construction of Series 5 Analogs

The first analog of Series 5 is identical to 3.5 (and 4.1), as described earlier (Scheme 9). Analog 5.2 was prepared from T**47** and T**42**, leading to peptide O**71** in 48% yield. Deprotection of both termini was followed by macrocyclization and 5.2 in 5% yield (3 steps). Analog 5.3 was built using the [4+4] approach as well, from T**46** and T**42**. Peptide O**72** was synthesized in 43% and then converted to 5.3 in 6% yield over the standard 3 final steps. Tetrapeptide T**47** was the bifurcation point to prepare O**73** in 50% yield. To this peptide was applied the standard finishing steps as well, leading to analog 5.4 in 7% yield (3 steps). The final analog (5.5) was prepared from T**46** and T**47** after N- and C-deprotections, respectively, to give O**74** in 49% yield. This peptide was converted to analog 5.5 in 9% yield (3 steps). The final analog of Series 5 is identical to 2.5, as described earlier.

All analogs were measured to >95% purity using a final purification by reverse phase HPLC. All analogs were noted to be oils at the point of preparation, and none have exhibited a tendency to solidify as of yet. Not unexpectedly, the ^1^H NMR spectra suggest more than one averaged conformation in each case, although some (e.g **2.2**, **3.4**) appeared more complex than others. A noteworthy finding is that the nonsymmetrical and highly *N*-methylated analogs displayed more complex ^1^H NMR spectra. In most cases, the region between 2.5 and 3.5 ppm highlights the presence of many different conformations. A key example of this is analog 1.2, as there are 2 major conformations, as well as many less prominent conformations seen by ^1^H NMR. Solubility issues became more apparent in the later series (3–5), but this was managed by using DMF as the reaction solvent. With the amide analogs, it was also shown that using HATU (rather than PyBrop) gave more efficient reactivity and conversion to product.

### Evaluation of Series **1–5** in the Calcium Sparks Assay

**Figure 3:**
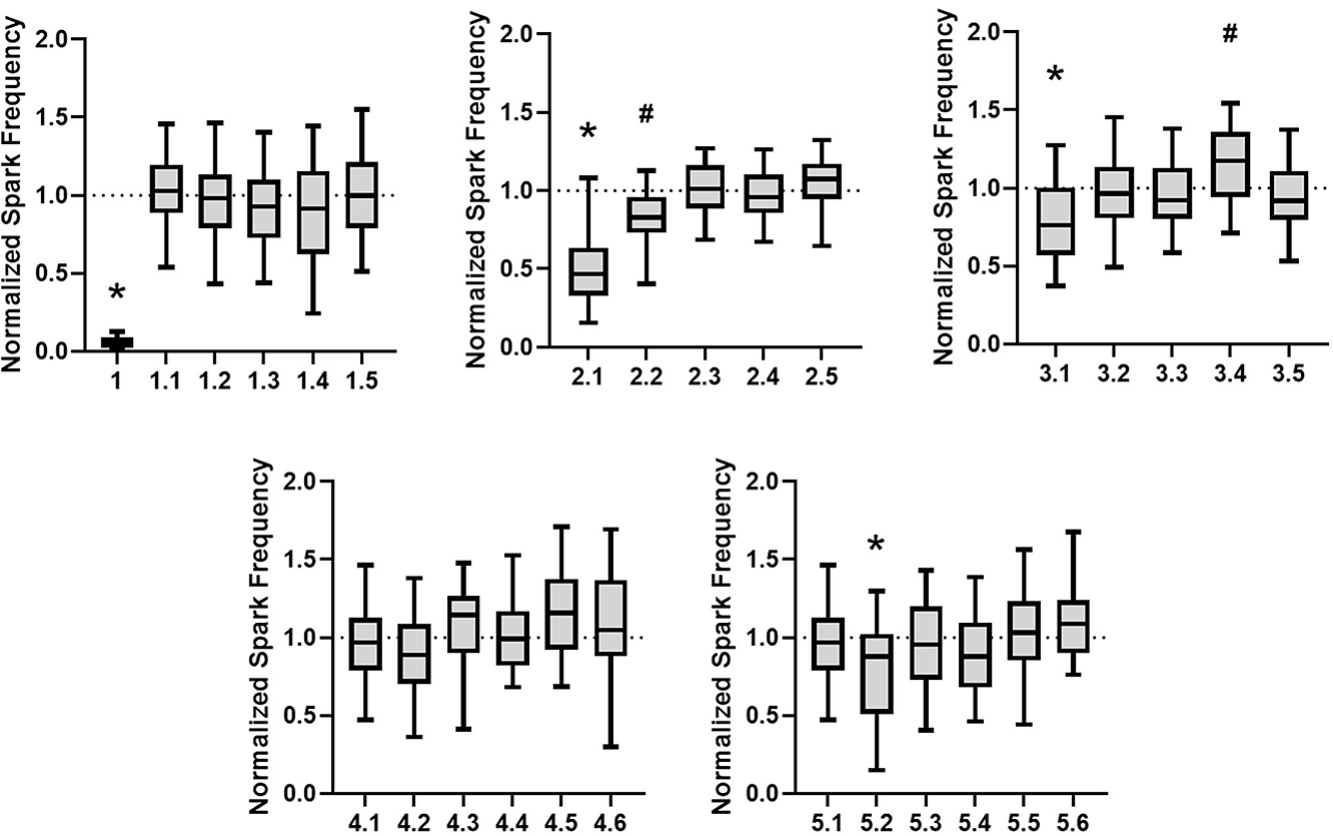
RyR2-mediated calcium spark frequency in permeabilized left ventricular myocytes incubated with 25 μM drug for 15 min. All data normalized to vehicle (DMSO) condition. Data displayed as interquartile range with median (boxes) and range (error bars). (A) *P < 0.0001 vs vehicle. *N* = 29 (2), 102 (5), 105 (5), 108 (5), 119 (4), and 87 cells (mice). Previously reported in Johnston et al. *ACS Med. Chem. Lett.*, 2022, **13**(11), 1755–1762. (B) *P < 0.0001 vs vehicle. # *P* = 0.00041 vs vehicle. *N* = 38, 46, 54, 50, and 50 cells, respectively, from 2 mice. (C) *P < 0.0001 vs vehicle. # *P* = 0.034 vs vehicle. *N* = 79 (4), 58 (3), 79 (4), 80 (5), and 91 (5) cells (mice). (D) *P* > 0.05 for all groups vs vehicle. *N* = 66 (4), 42 (2), 37 (2), 38 (2), 43(2), and 37 (2) cells (mice). (E) *P < 0.0001 vs vehicle. *N* = 66 (4), 85 (5), 93 (5), 100 (5), 89 (5), and 67 (4) cells (mice).

With a wide range of *ent*-verticilide analogs in hand, organized by five series that systematically change the backbone functionality without modifying the alternating methyl and pentyl side chains, we pursued their evaluation in the calcium sparks assay for insight into potential antiarrhythmic activity.^3^ Left ventricular myocytes were isolated from C57BL/6J mice and subjected to brief incubation with saponin, which permeabilizes the sarcolemma and enables equivalent access of compounds to RyR2, irrespective of their permeability.^14^ Cardiomyocytes were then incubated in an internal solution designed to promote spontaneous RyR2-mediated calcium release events, known as sparks,^34^ and the spark frequency was recorded following a 15 min incubation with 25 μM of each compound. Figure 3 summarizes these findings; no inhibition of RyR2 (lack of activity) is reflected as a value of 1 (dotted line). The relative activity of Series 1 compounds was reported previously, finding that introduction of one or more *N*–H amide(s) to the structure leads to loss of measurable activity.^35^ In that study, we noted that the 18-membered oligomer was more forgiving. Returning to the 24-membered ring oligomers studied here, Series 2 showed a significant trend, with introduction of a single *N*–H amide as a replacement for an ester (2.1) producing an analog with modest potency. A second ester conversion to an *N*–H amide was less tolerated (2.2), and further analogs with increased *N*–H amide:ester enrichment led to loss of activity. The SAR for Series 3 was less apparent, with 3.1 exhibiting partial inhibition, but less so than analog 2.1. Series 4–5 analogs produced nearly flat SAR, with no notable inhibition by any of the analogs. Although no rigorous examination of solubility under these conditions was made, solutions were freshly prepared for each assay and did not produce any observable precipitation.

## Conclusions

These results suggest that the native conformation of *ent*-verticilide is likely critical for target engagement. Insofar as increasing *N*–H amide content is somewhat tolerated with a single *N*–H amide/ester replacement, but less so with additional replacements, the single *N*–H amide may provide a transannular hydrogen bond that lightly affects the active conformation and pharmacophore. The ester content of the most active compounds (2.1, 3.1) appears beneficial when compared to those analogs enriched in *N*–Me or *N*–H amides. This finding suggests that replacement of an amide by an ester functionality can be beneficial. Our companion study that evaluates the permeability of Series 1–5 analogs using the PAMPA assay provides additional potential insight into the physicochemical impact of changes to the polar backbone.^14^ Importantly, the effects of these changes on activity do not overlay perfectly with changes to passive permeability, suggesting that the latter is not responsible for activity loss in Series 1–5. Overall, these analogs provide a picture of the structural and functional landscape for further optimization of the *ent*-verticilide template for antiarrhythmic drug development.

In conclusion, we explored the contribution of backbone functionality to activity using a systematic approach to comparison of ester, *N*–Me amide, and *N*–H amide functionality throughout a COD. This required the enantioselective synthesis of essential α-amino acid and α-oxy acid monomers and their use in the synthesis of *ent*-verticilide and 23 analogs. Evaluation of all compounds in the calcium sparks assay uncovered two analogs (2.1, 3.1) with measurable activity. Interestingly, both exhibit favorable passive permeability as well when measured by PAMPA.^14^ Further studies aimed at understanding the confluence of permeability and activity of these potential therapeutic will be reported in due course.

## Experimental Section

### Methods

#### Materials and Methods

Glassware was flame-dried under vacuum for all nonaqueous reactions. All reagents and solvents were commercial grade and purified prior to use when necessary. Toluene, THF, and dichloromethane (CH_2_Cl_2_) were dried by passage through a column of activated alumina, as described by Grubbs.^36^ Flash column chromatography was performed using Sorbent Technologies 230–400 mesh silica gel with solvent systems indicated. Analytical thin layer column chromatography was performed using Sorbent Technologies 250 μm glass backed UV254 silica gel plates and was visualized by fluorescence upon 250 nm radiation and/or the by use of TLC stain. Solvent removal was effected by rotary evaporation under vacuum (∼25–40 mmHg). All extracts were dried with Na_2_SO_4_ unless otherwise noted. Preparative HPLC was performed on an Agilent 1260 system (column: Zorbax Eclipse XDB-C18; 21.2 mm × 150 mm, 5 μm, flow rate 8 mL/min) with 210 nm monitoring wavelength and acetonitrile/water (+0.1% TFA) gradient as indicated. Nuclear magnetic resonance spectra (NMR) were acquired on a Bruker AV-400 (400 MHz) or Bruker AV II-600 (600 MHz) instrument. Mass spectra were recorded by use of electron impact ionization (EI) or electro-spray ionization (ESI) on a high-resolution TQ-Orbitrap 3 XL Penn or Orbitrap 2 Classic FPG in the Vanderbilt Mass Spectrometry Core Laboratory. IR spectra were recorded on a Nicolet Avatar 360 spectrophotometer and are reported in wavenumbers (cm^–1^) as neat films on a NaCl plate (transmission). Melting points were measured using an OptiMelt automated melting point system (Stanford Research Systems) and are not corrected. Chiral HPLC analysis was conducted on an Agilent 1200 series Infinity instrument using a ChiralPak column. Optical rotations were measured on a Jasco P-2000 polarimeter.

### Chemistry

The following procedures describe the synthesis of analog 2.5 and are representative. See the Supporting Information for complete experimental procedures and spectral data.

#### General Procedure for *tert*-Butyloxycarbonyl (Boc) Deprotection

A round bottom flask was charged with the depsipeptide (1 equiv) and dissolved in either 4 M HCl/ethyl acetate (1 M in depsipeptide) or 20% TFA in DCM. The reaction was allowed to stir for 1–3 h at ambient temperature. The crude reaction mixture was concentrated, ether was added, and the mixture was then reconcentrated. This procedure was repeated 3 times with diethyl ether.

#### General Procedure for Benzyl Deprotection

A round bottom flask was charged with the depsipeptide (1 equiv) dissolved in methanol or ethyl acetate (0.1 M) and treated with 10% Pd/C (20 mol %). The reaction flask was evacuated with a light vacuum (∼40 Torr). Hydrogen (balloon) was added, and then, the flask was cycled through a light vacuum three times. The reaction was stirred for 1.5–3 h. After purging the flask with argon, the crude reaction mixture was filtered through Celite and concentrated to afford the carboxylic acid.

#### 1-Tosylhexan-1-amine (**14**)

A round bottom flask was charged with hexanal (9.0 mL, 73 mmol), *tert*-butyl carbamate (5.7 g, 49 mmol), and MeOH (73 mL) and stirred until it became a homogeneous solution. NaSO_2_^*p*^Tol (17.4 g, 97.6 mmol) was added, along with enough water to dissolve the solids (100 mL). Then, formic acid (3.68 mL, 97.6 mmol) was added, and the mixture was allowed to stir at ambient temperature under argon for 4 days. The reaction mixture was filtered and washed with H_2_O and hexanes to afford the product as a white solid (15.5 g, 90%). M.p. 107–110 °C; *R*_f_ = 0.26 (10% EtOAc/hexanes); IR (film) 3334, 2958, 2930, 2861, 1721, 1597, 1518, 1456, 1392, 1316, 1245, 1167, 1142, 1085 cm^–1^; ^1^H NMR (600 MHz, CDCl_3_) the small doubling of peaks is due to cis and trans amide rotamers. The largely favored isomer is listed here: δ 7.76 (d, *J* = 8.8 Hz, 2H), 7.30 (d, *J* = 7.9 Hz, 2H), 5.10 (d, *J* = 11.0 Hz, 1H), 4.79 (td, *J* = 10.9, 3.4 Hz, 1H), 2.38 (s, 3H), 2.24–2.16 (m, 1H), 1.74–1.65 (m, 1H), 1.55–1.23 (m, 6H), 1.19 (s, 9H) 0.86 (t, *J* = 6.5 Hz, 3H); ^13^C NMR (150 MHz, CDCl_3_) ppm 154.0, 144.9, 134.0, 129.7, 129.4, 80.6, 31.2, 28.0, 27.7, 26.4, 25.0, 22.4, 21.7, 14.0; HRMS (EI): exact mass calcd for C_18_H_29_NnaO_4_S [M + Na]^+^ 378.1710, found 378.1719.

#### *tert*-Butyl((2*S*)-1-bromo-1-nitroheptan-2-yl)carbamate (**16**)

A round bottom flask was charged with sulfone **14** (6.00 g, 16.8 mmol), Cs_2_CO_3_ (27.5 g, 84.4 mmol), and toluene (160 mL). The mixture was allowed to stir at ambient temperature under argon for 6 h. The reaction mixture was filtered through a pad of Celite and concentrated. The crude oil was then dissolved in toluene (248 mL) and to it was added (*S*,*S*)-PBAM^26^ as its triflic acid salt (217.5 mg, 331 μmol). The reaction was cooled to −60 °C, and bromonitromethane (1.74 mL, 24.8 mmol) was added. The reaction was allowed to stir for 48 h. The reaction mixture was quenched by running it through a short silica plug while still cold, with 100% EtOAc. The fractions were combined and concentrated. The crude residue was subjected to flash column chromatography (SiO_2_, 5% ethyl acetate in hexanes) to afford the α-bromo nitroalkane (1:1 dr (^1^H NMR)) as a white solid (4.5 g, 88% 2-step); the diastereomers were determined to be 90 and 89% ee by chiral HPLC analysis (Chiralcel AD-H, 1% ^*i*^PrOH/hexanes, 1.0 mL/min, tr(d1, major) = 19.6 min, tr(d1, minor) = 21.1 min, tr(d2, minor) = 25.1 min, tr(d2, major) = 29.4 min). [α]_D_^23^ −20 (*c* 0.53, CHCl_3_); m.p. 62–66 °C; *R*_f_ = 0.60 (10% EtOAc/hexanes); IR (film) 3332, 2958, 2931, 2861, 1706, 1567, 1501, 1458, 1392, 1367, 1248, 1165, 1045 cm^–1^; ^1^H NMR (600 MHz, CDCl_3_, 1:1 mixture of diastereomers) δ 6.19 (d, *J* = 4.4 Hz, 1H), 6.17 (d, *J* = 3.0 Hz, 1H), 4.83 (d, *J* = 8.8 Hz, 1H), 4.73 (d, *J* = 9.1 Hz, 1H), 4.34 (dddd, *J* = 9.2, 9.2, 3.9, 3.9 Hz, 1H), 4.24 (dddd, *J* = 9.2, 9.2, 4.6, 4.6 Hz, 1H), 1.90–1.52 (series of m, 4H), 1.46 (s, 9H), 1.44 (s, 9H), 1.40–1.22 (m, 12H), 0.90 (t, *J* = 7.0 Hz, 3H), 0.88 (t, *J* = 6.9 Hz, 3H); ^13^C NMR (150 MHz, CDCl_3_, 1:1 mixture of diastereomers) ppm 155.2, 155.1, 84.5, 83.2, 80.9, 80.8, 55.0, 54.5, 31.32, 31.28, 30.5, 28.5, 28.4, 28.3, 25.6, 25.5, 22.52, 22.51, 14.0(2C); HRMS (EI): exact mass calcd for C_12_H_23_BrN_2_NaO_4_ [M + Na]^+^ 361.0733, found 361.0737.

#### Benzyl (*S*)-2-((*tert*-Butoxycarbonyl)amino)heptanoate (**19**)

A flame-dried round bottom flask was charged with halo-nitro alkane **16** (383 mg, 1.13 mmol), tin(II) chloride (428 mg, 2.26 mmol), and THF (11.3 mL). The mixture was allowed to stir at ambient temperature under argon for 12 h. The reaction mixture was then poured into H_2_O, and diethyl ether was added into the separatory funnel. The organic layer was filtered through a pad of Celite, washed with H_2_O (three times), dried, and concentrated to afford a pale yellow oil. The crude oil was then dissolved in DMSO (8.7 mL) and to it were added NaNO_2_ (234 mg, 3.39 mmol) and AcOH (885 μL, 17.0 mmol). The reaction was heated to 60 °C and allowed to stir for 12 h. The reaction mixture was allowed to cool to ambient temperature, quenched with 1 M aq HCl, poured into a separatory funnel, and extracted with EtOAc. The organic layers were washed with ice water (four times), dried, and concentrated. The crude reaction mixture was dissolved in DMF (2.1 mL), and to it were added K_2_CO_3_ (427 mg, 3.09 mmol) and BnBr (148 μL, 1.24 mmol). The reaction was allowed to stir at ambient temperature under argon for 12 h. The reaction was quenched with 1 M aq HCl and then extracted with EtOAc. The organic layers were then dried and concentrated. The crude residue was subjected to flash column chromatography (SiO_2_, 10% ethyl acetate in hexanes) to afford the product as a colorless oil (366 mg, 59% (3 steps)); [α]_D_^23^ −5.39 (*c* 1.08, CHCl_3_); *R*_f_ = 0.33 (10% EtOAc/hexanes); IR (film) 3365, 2957, 2931, 2861, 1717, 1499, 1456, 1366, 1249, 1161, 1048 cm^–1^; ^1^H NMR (400 MHz, CDCl_3_) δ 7.40–7.28 (m, 5H), 5.21 (d, *J* = 12.4 Hz, 1H), 5.12 (d, *J* = 12.4 Hz, 1H), 5.02 (d, *J* = 7.8 Hz, 1H), 4.35 (ddd, *J* = 11.7, 6.2, 6.2 Hz, 1H), 1.85–1.70 (m, 1H), 1.68–1.55 (m, 1H), 1.43 (s, 9H), 1.36–1.17 (m, 6H), 0.85 (t, *J* = 6.3 Hz, 3H); ^13^C NMR (100 MHz, CDCl_3_) ppm 172.9, 156.5, 136.6, 128.7, 128.4, 128.3, 79.8, 66.9, 53.7, 32.7, 31.4, 28.4, 24.9, 22.5, 14.0; HRMS (EI): exact mass calcd for C_19_H_29_NNaO_4_ [M + Na]^+^ 358.1989, found 358.1993.

#### Benzyl(*S*)-2-((*R*)-2-((*tert*-butoxycarbonyl)(methyl)amino)propanamido)heptanoate (**32**)

A round bottom flask was charged with amine M**20** (990 mg, 4.21 mmol), *N*-Boc*-N*-methyl alanine (855 mg, 4.21 mmol), and DMF (4.2 mL). The mixture was cooled to 0 °C, and then, DIPEA (2.20 mL, 12.6 mmol) and HATU (2.40 g, 6.32 mmol) were added. The reaction was stirred at 0 °C for 30 min, then warmed to room temperature, and stirred overnight. The reaction mixture was poured into cold 10% aq citric acid and extracted with EtOAc. The combined organic layers were washed with satd aq NaHCO_3_, brine, dried, and concentrated. The crude residue was subjected to flash column chromatography (SiO_2_, 5–30% ethyl acetate in hexanes) to afford the product as a white amorphous solid (1.15 g, 74%). [α]_D_^24^ + 29 (*c* 0.43, CHCl_3_); *R*_f_ = 0.42 (30% EtOAc/hexanes); IR (film) 3330, 2957, 2932, 2861, 1739, 1692, 1525, 1456, 1368, 1154, 1091, 1047 cm^–1^; ^1^H NMR (600 MHz, CDCl_3_) δ 7.37–7.32 (series of m, 5H), 6.72 (br m, 1H), 5.15–3.77 (series of m, 4H), 3.03–2.71 (series of m, 3H), 1.82 (br m, 1H), 1.65 (br m, 1H), 1.48–1.43 (series of m, 9H), 1.32–1.23 (series of m, 9H), 0.85 (br t, *J* = 6.9 Hz, 3H); ^13^C NMR (150 MHz, CDCl_3_) ppm 172.8, 172.2, 159.9, 130.7, 129.1, 128.9, 128.7, 81.0, 67.6, 52.8, 46.3, 32.7, 31.9, 30.4, 25.6, 25.5, 24.9, 23.0, 14.5; HRMS (EI): exact mass calcd for C_23_H_37_N_2_O_5_ [M + H]^+^ 421.2702, found 421.2684.

#### Benzyl(6*R*,9*S*,12*R*,15*S*)-2,2,5,6,11,12-hexamethyl-4,7,10,13-tetraoxo-9,15-dipentyl-3-oxa-5,8,11,14-tetraazahexadecan-16-oate (T**46**)

A round bottom flask was charged with amine D**34** (254 mg, 793 μmol), acid D**33** (262 mg, 793 μmol), and DCM (7.0 mL). The mixture was cooled to 0 °C, and then, DIPEA (414 μL, 2.38 mmol) and PyBrop (581 mg, 1.19 mmol) were added. The reaction was stirred at 0 °C for 30 min and then warmed to 23 °C for 2 h. The reaction mixture was poured into cold 10% aq citric acid and extracted with DCM. The combined organic layers were washed with satd aq NaHCO_3_, brine, dried, and concentrated. The crude residue was subjected to flash column chromatography (SiO_2_, 5–30% ethyl acetate in hexanes) to afford the product as a light yellow oil (611 mg, 94%). [α]_D_^24^ + 36 (*c* 0.86, CHCl_3_); *R*_f_ = 0.26 (30% EtOAc/hexanes); IR (film) 3318, 2956, 2931, 2860, 1742, 1661, 1530, 1456, 1367, 1256, 1154, 1090 cm^–1^; ^1^H NMR (600 MHz, CDCl_3_ δ 7.41–7.28 (series of m, 7H), 7.00 (br s, 1H), 6.70 (br s, 1H), 5.26 (br m, 1H), 5.13 (d, *J* = 12.1 Hz, 1H), 5.00 (d, *J* = 12.1 Hz, 1H), 4.71 (br m, 1H), 4.46 (m, 2H), 2.86–2.56 (series of m, 6H), 1.79 (br m, 1H), 1.64 (br m, 1H), 1.54 (br m, 1H), 1.43 (s, 9H), 1.37 (m, 3H), 1.27–1.17 (series of m, 16H), 0.83 (br t, *J* = 6.1 Hz, 3H), 0.78 (br t, *J* = 6.3 Hz, 3H); ^13^C NMR (150 MHz, CDCl_3_) ppm 172.5, 172.4, 172.0, 170.6, 161.0, 134.9, 128.7, 128.6, 128.5, 79.9, 68.9, 52.5, 52.0, 50.0, 42.2, 32.0, 31.9, 31.6, 31.5, 31.2, 30.8, 30.7, 29.9, 29.7, 29.4, 25.4, 25.0, 22.4, 14.0, 13.4; HRMS (EI): exact mass calcd for C_34_H_60_N_5_O_7_ [M + NH_4_]^+^ 650.4493, found 650.4487.

#### Benzyl(6*R*,9*S*,12*R*,15*S*,18*R*,21*S*,24*R*,27*S*)-2,2,5,6,11,12,17,18,23,24-decamethyl-4,7,10,13,16,19,22,25-octaoxo-9,15,21,27-tetrapentyl-3-oxa-5,8,11,14,17,20,23,26-octaazaoctacosan-28-oate (O**59**)

A round bottom flask was charged with amine T**46**-Bn (93.3 mg, 175 μmol), acid T**46**-Boc (95.0 mg, 175 μmol), and DMF (1.8 mL). The mixture was cooled to 0 °C, and then, DIPEA (91.5 μL, 525 μmol) and HATU (200 mg, 525 μmol) were added. The reaction was stirred at 0 °C for 30 min and then warmed to 23 °C for 18 h. The reaction mixture was poured into cold 10% aq citric acid and extracted with EtOAc. The combined organic layers were washed with satd aq NaHCO_3_, brine, dried, and concentrated. The crude residue was subjected to flash column chromatography (SiO_2_, 5–40% ethyl acetate in hexanes) to afford the product as a light yellow oil (117 mg, 63%). [α]_D_^24^ + 37 (*c* 0.45, CHCl_3_); *R*_f_ = 0.47 (30% EtOAc/hexanes); IR (film) 3405, 2956, 2929, 2859, 1741, 1652, 1540, 1457, 1391, 1260, 1155, 1092 cm^–1^; ^1^H NMR (600 MHz, CDCl_3_). This compound exists as a mixture of rotamers causing significant peak broadening and overlap. Refer to the image of the ^1^H NMR spectrum; ^13^C NMR (150 MHz, CDCl_3_) This compound is a mixture of rotamers causing significant peak broadening and overlap. Refer to the image of the ^13^C NMR spectrum; HRMS (EI): exact mass calcd for C_56_H_100_N_9_O_11_ [M + NH_4_]^+^ 1074.7542, found 1074.7544.

#### (3*S*,6*R*,9*S*,12*R*,15*S*,18*R*,21*S*,24*R*)-1,6,7,12,13,18,19,24-Octamethyl-3,9,15,21-tetrapentyl-1,4,7,10,13,16,19,22-octaazacyclotetracosan-2,5,8,11,14,17,20,23-octaone (**2.5**)

A round bottom flask was charged with depsipeptide O**59** (116 mg, 110 μmol), dissolved in EtOAc (1.1 mL), and treated with 10% Pd/C (2.4 mg). The reaction flask was evacuated with light vacuum. Hydrogen (balloon) was added, and the flask was cycled once more. The reaction was allowed to stir for 1 h. After purging the flask with argon, the crude reaction mixture was filtered through Celite. To the crude material was added 4 M HCl/dioxane (1.00 mL), and the reaction mixture was allowed to stir for 30 min. The reaction mixture was concentrated and added to a flame-dried round bottom flask. DMF (24.0 mL) was added, and the reaction was cooled to 0 °C. Once at 0 °C, DIPEA (43.4 μL, 330 μmol) and PyBop (60.0 mg, 115 μmol) were added. The reaction was stirred at 0 °C for 1.5 h and then allowed to warm to ambient temperature to stir for an additional 1 h. The reaction mixture was poured into cold 10% aq citric acid and extracted with EtOAc. The combined organic layers were washed with satd aq NaHCO_3_, brine, and then dried and concentrated. Preparative HPLC purification (5–95% aqueous acetonitrile, 210 nm, flow rate: 18 mL/min, *R*_t_ = 23.1 m) afforded the 24-membered macrocycle (10.2 mg, 11.0%) as a light yellow oil. [α]_D_^24^ − 11 (*c* 0.29, CHCl_3_); *R*_f_ = 0.33 (50% EtOAc in hexanes); IR (film) 3292, 2929, 1654, 1637, 1560, 1458 cm^–1^; ^1^H NMR (600 MHz, CDCl_3_). This compound exists in multiple conformations, causing significant peak overlap. Refer to image of the ^1^H NMR spectrum; ^13^C NMR (150 MHz, CDCl_3_) This compound exists in multiple conformations, causing significant peak overlap. Refer to image of the ^13^C NMR spectrum; HRMS (EI): exact mass calcd for C_44_H_84_N_9_O_8_ [M+NH_4_]^+^ 866.6443, found 866.6440.

### Biology

#### Ethics Statement

All animal work was approved (protocol number M1900081) and carried out in accordance with the guidelines and procedures set forth by the Vanderbilt Division of Animal Care.

#### Calcium Spark Assay

Calcium spark measurements were made, as previously described.^5^ Briefly, ventricular cardiomyocytes were isolated from C57Bl/6J mice, adhered to laminin-coated glass bottom culture dishes, permeabilized with saponin, and incubated with an internal solution containing the calcium sensitive dye, Fluo-4. Elementary spark recordings were made using an Olympus inverted microscope equipped with a solid-state diode laser at 488 nm for excitation, 40× silicone objective (1.25 NA), and Hamamatsu CMOS camera for detection. Spark detection analysis was performed using SparkMaster2.^37^

#### Statistics and Reproducibility

Statistical analysis was carried out in R using a hierarchical clustering model to cluster data by experiment and account for mouse-to-mouse variability.^38^ Bonferroni-adjusted *P* values are reported in the figure legends. A cutoff value of 0.05 was used as the threshold to reject the null hypothesis. The numbers of cells and replicates (mice) are indicated in the figure legend for each compound.

## Supporting Information Available

Compound preparations, analytical data, and spectroscopic data (PDF)
Molecular structures of all tested compounds and associated activity data (CSV)
Column and method information, detection methods, and method optimization (PDF)

## Abbreviations

Boc: *tert*-butoxy carbonyl
bRo5: beyond rule of 5
COD: cyclic oligomeric depsipeptide
DIPEA: diisopropyl ethyl amine
DMAP: dimethylamino pyridine
DMF: dimethylformamide
EDCI: 1-ethyl-3-(3-(dimethylamino)propyl)carbodiimide
HATU: hexafluorophosphate azabenzotriazole tetramethyl uronium
PBAM: pyrollidine bis(amidine)
PyBrop: bromotripyrrolidinophosphonium hexafluorophosphate
RyR: ryanodine receptor
RyR2: ryanodine receptor 2
SAR: structure–activity relationship
TFA: trifluoroacetic acid
