# Supplementary material for: Backbone-Determined Antiarrhythmic Structure–Activity Relationships for a Mirror Image, Oligomeric Depsipeptide Natural Product
Source: J Med Chem. 2024 Jul 3;67(14):12205–20. doi: 10.1021/acs.jmedchem.4c00923 (PMC11284792; doi:10.1021/acs.jmedchem.4c00923)

Backbone-Determined Antiarrhythmic Structure-Activity Relationships for a Mirror-Image, Oligomeric Depsipeptide Natural Product

Madelaine P. Thorpe,[1] Daniel J. Blackwell,[2] Bjorn C. Knollmann,[2] and Jeffrey N. Johnston[1]*

[1]Department of Chemistry and Vanderbilt Institute of Chemical Biology, Vanderbilt University, Nashville, Tennessee 37235

[2]Department of Medicine, Vanderbilt University Medical Center, Nashville, TN 37235

Table of Contents SI-X

Experimental Section ***9***

General Information **9**

Purity Statement **9**

Biological Experiments **9**

Biological Experiment Data: Calcium spark parameters 10

General Procedures ***11***

***tert*-Butyloxycarbonyl (Boc) Deprotection** **11**

Benzyl Deprotection **11**

PyBrop Coupling Reaction **11**

PyBOP Macrocyclization **11**

HATU Coupling Reaction **12**

General Reaction Schemes ***12***

Experimental Procedures and Characterization Data for Reported Compounds ***13***

1-Tosylhexan-1-amine (14). 13

tert-Butyl ((2*S*)-1-bromo-1-nitroheptan-2-yl)carbamate (16). 13

Benzyl (*S*)-2-((*tert*-butoxycarbonyl)amino)heptanoate (19). 14

Benzyl (*S*)-2-((*tert*-butoxycarbonyl)(methyl)amino)heptanoate (21). 14

Benzyl (*S*)-2-(((*tert*-butoxycarbonyl)-*D*-alanyl)oxy)heptanoate (23). 15

Benzyl (*S*)-2-((*R*)-2-((*tert*-butoxycarbonyl)amino)-*N*-methylpropanamido)heptanoate (29). 15

Benzyl (*S*)-2-((*R*)-2-((*tert*-butoxycarbonyl)(methyl)amino)propanamido)heptanoate (32). 16

Benzyl (*S*)-2-((*R*)-2-((*tert*-butoxycarbonyl)(methyl)amino)-*N*-methylpropanamido)heptanoate (35). 16

Benzyl (*R*)-2-(((6*R*,9*R*,12*R*)-2,2,5,6,11,12-hexamethyl-4,7,10-trioxo-9-pentyl-3,8-dioxa-5,11-diazatridecan-13-oyl)oxy) heptanoate (T38). 17

(*S*)-1-(benzyloxy)-1-oxoheptan-2-yl (6*R*,9*S*,12*R*)-2,2,5,6,11,12-hexamethyl-4,7,10-trioxo-9-pentyl-3-oxa-5,8,11-triazatridecan-13-oate (T40). 17

Benzyl (*S*)-2-((6*R*,9*S*,12*R*)-N,2,2,5,6,11,12-heptamethyl-4,7,10-trioxo-9-pentyl-3,8-dioxa-5,11-diazatridecan-13-amido)heptanoate (T41). 18

Benzyl (6*R*,9*S*,12*R*,15*S*)-2,2,5,6,8,11,12,14-octamethyl-4,7,10,13-tetraoxo-9,15-dipentyl-3-oxa-5,8,11,14-tetraazahexadecan-16-oate (T42). 18

Benzyl (6*R*,9*S*,12*R*,15*S*)-2,2,5,6,8,12,14-heptamethyl-4,7,10,13-tetraoxo-9,15-dipentyl-3-oxa-5,8,11,14-tetraazahexadecan-16-oate (T43). 19

Benzyl (6*R*,9*S*,12*R*,15*S*)-2,2,6,8,12,14-hexamethyl-4,7,10,13-tetraoxo-9,15-dipentyl-3-oxa-5,8,11,14-tetraazahexadecan-16-oate (T44). 19

Benzyl (6*R*,9*S*,12*R*,15*S*)-2,2,6,8,11,12,14-heptamethyl-4,7,10,13-tetraoxo-9,15-dipentyl-3-oxa-5,8,11,14-tetraazahexadecan-16-oate (T45). 20

Benzyl (6*R*,9*S*,12*R*,15*S*)-2,2,5,6,11,12-hexamethyl-4,7,10,13-tetraoxo-9,15-dipentyl-3-oxa-5,8,11,14-tetraazahexadecan-16-oate (T46). 20

Benzyl (6*R*,9*S*,12*R*,15*S*)-2,2,5,6,8,11,12-heptamethyl-4,7,10,13-tetraoxo-9,15-dipentyl-3-oxa-5,8,11,14-tetraazahexadecan-16-oate (T47). 21

Benzyl (*S*)-2-(((6*R*,9*S*,12*R*,15*S*,18*R*,21*S*,24*R*)-2,2,6,11,12,17,18,23,24-nonamethyl-4,7,10,13,16,19,22-heptaoxo-9,15,21-tripentyl-3,8,14,20-tetraoxa-5,11,17,23-tetraazapentacosan-25-oyl)oxy)heptanoate (O49). 21

Benzyl (*S*)-2-(((6*R*,9*S*,12*R*,15*S*,18*R*,21*S*,24*R*)-2,2,5,6,11,12,18,24-octamethyl-4,7,10,13,16,19,22-heptaoxo-9,15,21-tripentyl-3,8,14,20-tetraoxa-5,11,17,23-tetraazapentacosan-25-oyl)oxy)heptanoate (O50). 22

Benzyl (*S*)-2-(((6*R*,9*S*,12*R*,15*S*,18*R*)-2,2,6,12,18-pentamethyl-4,7,10,13,16-pentaoxo-9,15-dipentyl-3,8,14-trioxa- 5,11,17-triazanonadecan-19-oyl)oxy)heptanoate (H51). 23

Benzyl (*S*)-2-(((6*R*,9*S*,12*R*,15*S*,18*R*,21*S*,24*R*)-2,2,5,6,12,18,24-heptamethyl-4,7,10,13,16,19,22-heptaoxo-9,15,21-tripentyl-3,8,14,20-tetraoxa-5,11,17,23-tetraazapentacosan-25-oyl)oxy)heptanoate (O52). 23

(4*S*,7*R*,10*S*,13*R*,16*S*)-7,8,13,14-tetramethyl-3,6,9,12,15-pentaoxo-4,10-dipentyl-1-phenyl-2,5,11-trioxa-8,14-diazahenicosan-16-yl (6*R*,9*S*,12*R*)-2,2,5,6,11,12-hexamethyl-4,7,10-trioxo-9-pentyl-3-oxa-5,8,11-triazatridecan-13-oate (O54). 24

(*S*)-1-(((*R*)-1-(((*S*)-1-(benzyloxy)-1-oxoheptan-2-yl)oxy)-1-oxopropan-2-yl)(methyl)amino)-1-oxoheptan-2-yl (6*R*,9*S*,12*R*,15*S*,18*R*)-2,2,5,6,11,12,17,18-octamethyl-4,7,10,13,16-pentaoxo-9,15-dipentyl-3-oxa-5,8,11,14,17-pentaazanonadecan-19-oate (O55). 25

(4*S*,7*R*,10*S*,13*R*,16*S*)-7,8,13,14-tetramethyl-3,6,9,12,15-pentaoxo-4,10-dipentyl-1-phenyl-2,5-dioxa-8,11,14-triazahenicosan-16-yl (6*R*,9*S*,12*R*)-2,2,5,6,11,12-hexamethyl-4,7,10-trioxo-9-pentyl-3-oxa-5,8,11-triazatridecan-13-oate (O56). 25

Benzyl (6*R*,9*S*,12*R*,15*S*,18*R*,21*S*)-2,2,5,6,11,12,17,18-octamethyl-4,7,10,13,16,19-hexaoxo-9,15,21-tripentyl-3-oxa-5,8,11,14,17,20-hexaazadocosan-22-oate (H57). 26

Benzyl (2*S*,5*R*,8*S*,11*R*,14*S*,17*R*,20*S*)-5,6,11,12,17,18-hexamethyl-20-((methyl-*D*-alanyl)oxy)-4,7,10,13,16,19-hexaoxo-2,8,14-tripentyl-3,6,9,12,15,18-hexaazapentacosanoate (O58, Boc-deprotected). 27

Benzyl (6*R*,9*S*,12*R*,15*S*,18*R*,21*S*,24*R*,27*S*)-2,2,5,6,11,12,17,18,23,24-decamethyl-4,7,10,13,16,19,22,25-octaoxo-9,15,21,27-tetrapentyl-3-oxa-5,8,11,14,17,20,23,26-octaazaoctacosan-28-oate (O59). 28

Benzyl (4*S*,7*R*,10*S*,13*R*,16*S*)-7,8,13,14-tetramethyl-3,6,9,12,15-pentaoxo-4,10-dipentyl-1-phenyl-2,5,11-trioxa-8,14-diazahenicosan-16-yl (6*R*,9*S*,12*R*)-2,2,5,6,8,11,12-heptamethyl-4,7,10-trioxo-9-pentyl-3-oxa-5,8,11-triazatridecan-13-oate (O60). 28

Benzyl (2*S*,5*R*,8*S*,11*R*,14*S*)-14-(((6R,9S,12R)-2,2,5,6,11,12-hexamethyl-4,7,10-trioxo-9-pentyl-3,8-dioxa-5,11-diazatridecan-13-oyl)oxy)-3,5,6,9,11,12-hexamethyl-4,7,10,13-tetraoxo-2,8-dipentyl-3,6,9,12-tetraazanonadecanoate (O61). 29

Benzyl (*S*)-2-((6*R*,9*S*,12*R*,15*S*,18*R*,21*S*,24*R*)-N,2,2,5,6,11,12,14,17,18,23,24-dodecamethyl-4,7,10,13,16,19,22-heptaoxo-9,15,21-tripentyl-3,8,20-trioxa-5,11,14,17,23-pentaazapentacosan-25-amido)heptanoate (O62). 30

Benzyl (6*R*,9*S*,12*R*,15*S*,18*R*,21*S*)-2,2,5,6,8,11,12,14,17,18,20-undecamethyl-4,7,10,13,16,19-hexaoxo-9,15,21-tripentyl-3-oxa-5,8,11,14,17,20-hexaazadocosan-22-oate (H63). 30

(*S*)-1-(benzyloxy)-1-oxoheptan-2-yl (6*R*,9*S*,12*R*,15*S*,18*R*,21*S*,24*R*)-2,2,5,6,8,11,12,14,17,18,20,23,24-tridecamethyl-4,7,10,13,16,19,22-heptaoxo-9,15,21-tripentyl-3-oxa-5,8,11,14,17,20,23-heptaazapentacosan-25-oate (O64). 31

Benzyl (6*R*,9*S*,12*R*,15*S*,18*R*,21*S*,24*R*,27*S*)-2,2,5,6,8,11,12,14,17,18,20,23,24,26-tetradecamethyl-4,7,10,13,16,19,22,25-octaoxo-9,15,21,27-tetrapentyl-3-oxa-5,8,11,14,17,20,23,26-octaazaoctacosan-28-oate (O65). 32

Benzyl (6*R*,9*S*,12*R*,15*S*,18*R*,21*S*,24*R*,27*S*)-2,2,6,8,11,12,14,17,18,20,23,24,26-tridecamethyl-4,7,10,13,16,19,22,25-octaoxo-9,15,21,27-tetrapentyl-3-oxa-5,8,11,14,17,20,23,26-octaazaoctacosan-28-oate (O66). 33

Benzyl (6*R*,9*S*,12*R*,15*S*,18*R*,21*S*,24*R*,27*S*)-2,2,5,6,8,11,12,14,18,20,24,26-dodecamethyl-4,7,10,13,16,19,22,25-octaoxo-9,15,21,27-tetrapentyl-3-oxa-5,8,11,14,17,20,23,26-octaazaoctacosan-28-oate (O67). 34

Benzyl (6*R*,9*S*,12*R*,15*S*,18*R*,21*S*,24*R*,27*S*)-2,2,5,6,8,12,14,17,18,20,24,26-dodecamethyl-4,7,10,13,16,19,22,25-octaoxo-9,15,21,27-tetrapentyl-3-oxa-5,8,11,14,17,20,23,26-octaazaoctacosan-28-oate (O68). 35

Benzyl (6*R*,9*S*,12*R*,15*S*,18*R*,21*S*,24*R*,27*S*)-2,2,5,6,8,12,14,18,20,24,26-undecamethyl-4,7,10,13,16,19,22,25-octaoxo-9,15,21,27-tetrapentyl-3-oxa-5,8,11,14,17,20,23,26-octaazaoctacosan-28-oate (O69). 36

Benzyl (6*R*,9*S*,12*R*,15*S*,18*R*,21*S*,24*R*,27*S*)-2,2,6,8,12,14,18,20,24,26-decamethyl-4,7,10,13,16,19,22,25-octaoxo-9,15,21,27-tetrapentyl-3-oxa-5,8,11,14,17,20,23,26-octaazaoctacosan-28-oate (O70). 37

Benzyl (6*R*,9*S*,12*R*,15*S*,18*R*,21*S*,24*R*,27*S*)-2,2,5,6,8,11,12,14,17,18,20,23,24-tridecamethyl-4,7,10,13,16,19,22,25-octaoxo-9,15,21,27-tetrapentyl-3-oxa-5,8,11,14,17,20,23,26-octaazaoctacosan-28-oate (O71). 37

Benzyl (6*R*,9*S*,12*R*,15*S*,18*R*,21*S*,24*R*,27*S*)-2,2,5,6,8,11,12,14,17,18,23,24-dodecamethyl-4,7,10,13,16,19,22,25-octaoxo-9,15,21,27-tetrapentyl-3-oxa-5,8,11,14,17,20,23,26-octaazaoctacosan-28-oate (O72). 38

Benzyl (6*R*,9*S*,12*R*,15*S*,18*R*,21*S*,24*R*,27*S*)-2,2,5,6,8,11,12,17,18,20,23,24-dodecamethyl-4,7,10,13,16,19,22,25-octaoxo-9,15,21,27-tetrapentyl-3-oxa-5,8,11,14,17,20,23,26-octaazaoctacosan-28-oate (O73). 39

Benzyl (6*R*,9*S*,12*R*,15*S*,18*R*,21*S*,24*R*,27*S*)-2,2,5,6,8,11,12,17,18,23,24-undecamethyl-4,7,10,13,16,19,22,25-octaoxo-9,15,21,27-tetrapentyl-3-oxa-5,8,11,14,17,20,23,26-octaazaoctacosan-28-oate (O74). 40

(3*R*,6*S*,9*R*,12*S*,15*R*,18*S*,21*R*,24*S*)-3,4,9,10,15,16,21-heptamethyl-6,12,18,24-tetrapentyl-1,7,13,19-tetraoxa-4,10,16,22-tetraazacyclotetracosan-2,5,8,11,14,17,20,23-octaone (1.1). 40

(3*R*,6*S*,9*R*,12*S*,15*R*,18*S*,21*R*,24*S*)-3,4,9,10,15,21-hexamethyl-6,12,18,24-tetrapentyl-1,7,13,19-tetraoxa-4,10,16,22-tetraazacyclotetracosan-2,5,8,11,14,17,20,23-octaone (1.2). 41

(3*R*,6*S*,9*R*,12*S*,15*R*,18*S*,21*R*,24*S*)-3,4,9,15,16,21-hexamethyl-6,12,18,24-tetrapentyl-1,7,13,19-tetraoxa-4,10,16,22-tetraazacyclotetracosan-2,5,8,11,14,17,20,23-octaone (1.3). 42

(3*R*,6*S*,9*R*,12*S*,15*R*,18*S*,21*R*,24*S*)-3,4,9,15,21-pentamethyl-6,12,18,24-tetrapentyl-1,7,13,19-tetraoxa-4,10,16,22-tetraazacyclotetracosan-2,5,8,11,14,17,20,23-octaone (1.4). 42

(3*R*,6*S*,9*R*,12*S*,15*R*,18*S*,21*R*,24*S*)-3,4,9,10,15,16,21,22-octamethyl-6,12,18,24-tetrapentyl-1,7,13-trioxa-4,10,16,19,22-pentaazacyclotetracosan-2,5,8,11,14,17,20,23-octaone (2.1). 43

(3*R*,6*S*,9*R*,12*S*,15*R*,18*S*,21*R*,24*S*)-3,4,9,10,15,16,21,22-octamethyl-6,12,18,24-tetrapentyl-1,7,13-trioxa-4,10,16,19,22-pentaazacyclotetracosan-2,5,8,11,14,17,20,23-octaone (2.2). 44

(3*R*,6*S*,9*R*,12*S*,15*R*,18*S*,21*R*,24*S*)-3,4,9,10,15,16,21,22-octamethyl-6,12,18,24-tetrapentyl-1,13-dioxa-4,7,10,16,19,22-hexaazacyclotetracosan-2,5,8,11,14,17,20,23-octaone (2.3). 45

(3*R*,6*S*,9*R*,12*S*,15*R*,18*S*,21*R*,24*S*)-3,4,9,10,15,16,21,22-octamethyl-6,12,18,24-tetrapentyl-1-oxa-4,7,10,13,16,19,22-heptaazacyclotetracosan-2,5,8,11,14,17,20,23-octaone (2.4). 45

(3*S*,6*R*,9*S*,12*R*,15*S*,18*R*,21*S*,24*R*)-1,6,7,12,13,18,19,24-octamethyl-3,9,15,21-tetrapentyl-1,4,7,10,13,16,19,22-octaazacyclotetracosan-2,5,8,11,14,17,20,23-octaone (2.5). 46

(3*R*,6*S*,9*R*,12*S*,15*R*,18*S*,21*R*,24*S*)-3,4,9,10,15,16,19,21,22-nonamethyl-6,12,18,24-tetrapentyl-1,7,13-trioxa-4,10,16,19,22-pentaazacyclotetracosan-2,5,8,11,14,17,20,23-octaone (3.1) 47

(3*R*,6*S*,9*R*,12*S*,15*R*,18*S*,21*R*,24*S*)-3,4,9,10,13,15,16,19,21,22-decamethyl-6,12,18,24-tetrapentyl-1,7-dioxa-4,10,13,16,19,22-hexaazacyclotetracosan-2,5,8,11,14,17,20,23-octaone (3.2). 47

(3*R*,6*S*,9*R*,12*S*,15*R*,18*S*,21*R*,24*S*)-3,4,7,9,10,15,16,19,21,22-decamethyl-6,12,18,24-tetrapentyl-1,13-dioxa-4,7,10,16,19,22-hexaazacyclotetracosan-2,5,8,11,14,17,20,23-octaone (3.3). 48

(3*R*,6*S*,9*R*,12*S*,15*R*,18*S*,21*R*,24*S*)-3,4,7,9,10,13,15,16,19,21,22-undecamethyl-6,12,18,24-tetrapentyl-1-oxa-4,7,10,13,16,19,22-heptaazacyclotetracosan-2,5,8,11,14,17,20,23-octaone (3.4). 49

(3*R*,6*S*,9*R*,12*S*,15*R*,18*S*,21*R*,24*S*)-1,3,4,7,9,10,13,15,16,19,21,22-dodecamethyl-6,12,18,24-tetrapentyl-1,4,7,10,13,16,19,22-octaazacyclotetracosan-2,5,8,11,14,17,20,23-octaone (3.5). 49

(3*R*,6*S*,9*R*,12*S*,15*R*,18*S*,21*R*,24*S*)-1,3,4,7,9,10,13,15,16,19,21-undecamethyl-6,12,18,24-tetrapentyl-1,4,7,10,13,16,19,22-octaazacyclotetracosan-2,5,8,11,14,17,20,23-octaone (4.2). 50

(3*R*,6*S*,9*R*,12*S*,15*R*,18*S*,21*R*,24*S*)-1,3,4,7,9,10,13,15,19,21-decamethyl-6,12,18,24-tetrapentyl-1,4,7,10,13,16,19,22-octaazacyclotetracosan-2,5,8,11,14,17,20,23-octaone (4.3). 51

(3*R*,6*S*,9*R*,12*S*,15*R*,18*S*,21*R*,24*S*)-1,3,4,7,9,10,13,15,19,21-decamethyl-6,12,18,24-tetrapentyl-1,4,7,10,13,16,19,22-octaazacyclotetracosan-2,5,8,11,14,17,20,23-octaone (4.4). 51

(3*R*,6*S*,9*R*,12*S*,15*R*,18*S*,21*R*,24*S*)-1,3,4,7,9,13,15,19,21-nonamethyl-6,12,18,24-tetrapentyl-1,4,7,10,13,16,19,22-octaazacyclotetracosan-2,5,8,11,14,17,20,23-octaone (4.5). 52

(3*R*,6*S*,9*R*,12*S*,15*R*,18*S*,21*R*,24*S*)-1,3,7,9,13,15,19,21-octamethyl-6,12,18,24-tetrapentyl-1,4,7,10,13,16,19,22-octaazacyclotetracosan-2,5,8,11,14,17,20,23-octaone (4.6). 53

(3*S*,6*R*,9*S*,12*R*,15*S*,18*R*,21*S*,24*R*)-1,4,6,7,10,12,13,16,18,19,24-undecamethyl-3,9,15,21-tetrapentyl-1,4,7,10,13,16,19,22-octaazacyclotetracosan-2,5,8,11,14,17,20,23-octaone (5.2). 53

(3*S*,6*R*,9*S*,12*R*,15*S*,18*R*,21*S*,24*R*)-1,4,6,7,10,12,13,18,19,24-decamethyl-3,9,15,21-tetrapentyl-1,4,7,10,13,16,19,22-octaazacyclotetracosan-2,5,8,11,14,17,20,23-octaone (5.3). 54

(3*S*,6*R*,9*S*,12*R*,15*S*,18*R*,21*S*,24*R*)-1,4,6,7,12,13,16,18,19,24-decamethyl-3,9,15,21-tetrapentyl-1,4,7,10,13,16,19,22-octaazacyclotetracosan-2,5,8,11,14,17,20,23-octaone (5.4). 55

(3*S*,6*R*,9*S*,12*R*,15*S*,18*R*,21*S*,24*R*)-1,4,6,7,12,13,18,19,24-nonamethyl-3,9,15,21-tetrapentyl-1,4,7,10,13,16,19,22-octaazacyclotetracosan-2,5,8,11,14,17,20,23-octaone (5.5). 55

Characterization NMR Spectra 57

Figure S1. ^{1}H NMR (600 MHz, $CDCl_3$) of 14 57

Figure S2. ^{13}C NMR (150 MHz, $CDCl_3$) of 14 58

Figure S3. ^{1}H NMR (600 MHz, $CDCl_3$) of 16 59

Figure S4. ^{13}C NMR (150 MHz, $CDCl_3$) of 16 60

Figure S5. ^{1}H NMR (600 MHz, $CDCl_3$) of 19 61

Figure S6. ^{13}C NMR (150 MHz, $CDCl_3$) of 19 62

Figure S7. ^{1}H NMR (600 MHz, $CDCl_3$) of 21 63

Figure S8. ^{13}C NMR (150 MHz, $CDCl_3$) of 21 64

Figure S9. ^{1}H NMR (600 MHz, $CDCl_3$) of 23 65

Figure S10. ^{13}C NMR (150 MHz, $CDCl_3$) of 23 66

Figure S11. ^{1}H NMR (600 MHz, $CDCl_3$) of 29 67
Figure S12. ^{13}C NMR (150 MHz, $CDCl_3$) of 29 68
Figure S13. ^{1}H NMR (600 MHz, $CDCl_3$) of 32 69
Figure S14. ^{13}C NMR (150 MHz, $CDCl_3$) of 32 70
Figure S15. ^{1}H NMR (600 MHz, $CDCl_3$) of 35 71
Figure S16. ^{13}C NMR (150 MHz, $CDCl_3$) of 35 72
Figure S17. ^{1}H NMR (600 MHz, $CDCl_3$) of T38 73
Figure S18. ^{13}C NMR (150 MHz, $CDCl_3$) of T38 74
Figure S19. ^{1}H NMR (600 MHz, $CDCl_3$) of T40 75
Figure S20. ^{13}C NMR (150 MHz, $CDCl_3$) of T40 76
Figure S21. ^{1}H NMR (600 MHz, $CDCl_3$) of T41 77
Figure S22. ^{13}C NMR (150 MHz, $CDCl_3$) of T41 78
Figure S23. ^{1}H NMR (600 MHz, $CDCl_3$) of T42 79
Figure S24. ^{13}C NMR (150 MHz, $CDCl_3$) of T42 80
Figure S25. ^{1}H NMR (600 MHz, $CDCl_3$) of T43 81
Figure S26. ^{13}C NMR (150 MHz, $CDCl_3$) of T43 82
Figure S27. ^{1}H NMR (600 MHz, $CDCl_3$) of T44 83
Figure S28. ^{13}C NMR (150 MHz, $CDCl_3$) of T44 84
Figure S29. ^{1}H NMR (600 MHz, $CDCl_3$) of T45 85
Figure S30. ^{13}C NMR (150 MHz, $CDCl_3$) of T45 86
Figure S31. ^{1}H NMR (600 MHz, $CDCl_3$) of T46 87
Figure S32. ^{13}C NMR (150 MHz, $CDCl_3$) of T46 88
Figure S33. ^{1}H NMR (600 MHz, $CDCl_3$) of T47 89
Figure S34. ^{13}C NMR (150 MHz, $CDCl_3$) of T47 90
Figure S35. ^{1}H NMR (600 MHz, $CDCl_3$) of O49 91
Figure S36. ^{13}C NMR (150 MHz, $CDCl_3$) of O49 92
Figure S37. ^{1}H NMR (600 MHz, $CDCl_3$) of O50 93
Figure S38. ^{13}C NMR (150 MHz, $CDCl_3$) of O50 94
Figure S39. ^{1}H NMR (600 MHz, $CDCl_3$) of H51 95
Figure S40. ^{13}C NMR (150 MHz, $CDCl_3$) of H51 96
Figure S41. ^{1}H NMR (600 MHz, $CDCl_3$) of O52 97
Figure S42. ^{13}C NMR (150 MHz, $CDCl_3$) of O52 98
Figure S43. ^{1}H NMR (600 MHz, $CDCl_3$) of O54 99
Figure S44. ^{13}C NMR (150 MHz, $CDCl_3$) of O54 100
Figure S45. ^{1}H NMR (600 MHz, $CDCl_3$) of O55 101

Figure S46. ^{13}C NMR (150 MHz, $CDCl_3$) of O55 102
Figure S47. ^{1}H NMR (600 MHz, $CDCl_3$) of O56 103
Figure S48. ^{13}C NMR (150 MHz, $CDCl_3$) of O56 104
Figure S49. ^{1}H NMR (600 MHz, $CDCl_3$) of H57 105
Figure S50. ^{13}C NMR (150 MHz, $CDCl_3$) of H57 106
Figure S51. ^{1}H NMR (600 MHz, $CDCl_3$) of O58 (Boc deprotected) 107
Figure S52. ^{13}C NMR (150 MHz, $CDCl_3$) of O58 (Boc deprotected) 108
Figure S53. ^{1}H NMR (600 MHz, $CDCl_3$) of O59 109
Figure S54. ^{13}C NMR (150 MHz, $CDCl_3$) of O59 110
Figure S55. ^{1}H NMR (600 MHz, $CDCl_3$) of O60 111
Figure S56. ^{13}C NMR (150 MHz, $CDCl_3$) of O60 112
Figure S57. ^{1}H NMR (600 MHz, $CDCl_3$) of O61 113
Figure S58. ^{13}C NMR (150 MHz, $CDCl_3$) of O61 114
Figure S59. ^{1}H NMR (600 MHz, $CDCl_3$) of O62 115
Figure S60. ^{13}C NMR (150 MHz, $CDCl_3$) of O62 116
Figure S61. ^{1}H NMR (600 MHz, $CDCl_3$) of H63 117
Figure S62. ^{13}C NMR (150 MHz, $CDCl_3$) of H63 118
Figure S63. ^{1}H NMR (600 MHz, $CDCl_3$) of O64 119
Figure S64. ^{13}C NMR (150 MHz, $CDCl_3$) of O64 120
Figure S65. ^{1}H NMR (600 MHz, $CDCl_3$) of O65 121
Figure S66. ^{13}C NMR (150 MHz, $CDCl_3$) of O65 122
Figure S67. ^{1}H NMR (600 MHz, $CDCl_3$) of O66 123
Figure S68. ^{13}C NMR (150 MHz, $CDCl_3$) of O66 124
Figure S69. ^{1}H NMR (600 MHz, $CDCl_3$) of O67 125
Figure S70. ^{13}C NMR (150 MHz, $CDCl_3$) of O67 126
Figure S71. ^{1}H NMR (600 MHz, $CDCl_3$) of O68 127
Figure S72. ^{13}C NMR (150 MHz, $CDCl_3$) of O68 128
Figure S73. ^{1}H NMR (600 MHz, $CDCl_3$) of O69 129
Figure S74. ^{13}C NMR (150 MHz, $CDCl_3$) of O69 130
Figure S75. ^{1}H NMR (600 MHz, $CDCl_3$) of O70 131
Figure S76. ^{13}C NMR (150 MHz, $CDCl_3$) of O70 132
Figure S77. ^{1}H NMR (600 MHz, ($CDCl_3$) of O71 133
Figure S78. ^{13}C NMR (150 MHz, $CDCl_3$) of O71 134
Figure S79. ^{1}H NMR (600 MHz, CD_3COCD_3) of O72........135
Figure S80. ^{13}C NMR (150 MHz, $CDCl_3$) of O72 136

Figure S81. ^{1}H NMR (600 MHz, $CDCl_3$) of O73 137
Figure S82. ^{13}C NMR (150 MHz, $CDCl_3$) of O73 138
Figure S83. ^{1}H NMR (600 MHz, $CDCl_3$) of O74 139
Figure S84. ^{13}C NMR (150 MHz, $CDCl_3$) of O74 140
Figure S85. ^{1}H NMR (600 MHz, $CDCl_3$) of 1.1 141
Figure S86. ^{13}C NMR (150 MHz, $CDCl_3$) of 1.1 142
Figure S87. ^{1}H NMR (600 MHz, $CDCl_3$) of 1.2 143
Figure S88. ^{13}C NMR (150 MHz, $CDCl_3$) of 1.2 144
Figure S89. ^{1}H NMR (600 MHz, $CDCl_3$) of 1.3 145
Figure S90. ^{13}C NMR (150 MHz, $CDCl_3$) of 1.3 146
Figure S91. ^{1}H NMR (600 MHz, $CDCl_3$) of 1.4 147
Figure S92. ^{13}C NMR (150 MHz, $CDCl_3$) of 1.4 148
Figure S93. ^{1}H NMR (600 MHz, CD_3COCD_3) of 2.1 149
Figure S94. ^{13}C NMR (150 MHz, $CDCl_3$) of 2.1 150
Figure S95. ^{1}H NMR (600 MHz, $CDCl_3$) of 2.2 151
Figure S96. ^{13}C NMR (150 MHz, $CDCl_3$) of 2.2 152
Figure S97. ^{1}H NMR (600 MHz, $CDCl_3$) of 2.3 153
Figure S98. ^{13}C NMR (150 MHz, $CDCl_3$) of 2.3 154
Figure S99. ^{1}H NMR (600 MHz, $CDCl_3$) of 2.4 155
Figure S100. ^{13}C NMR (150 MHz, $CDCl_3$) of 2.4 156
Figure S101. ^{1}H NMR (600 MHz, $CDCl_3$) of 2.5 157
Figure S102. ^{13}C NMR (150 MHz, $CDCl_3$) of 2.5 158
Figure S103. ^{1}H NMR (600 MHz, $CDCl_3$) of 3.1 159
Figure S104. ^{13}C NMR (150 MHz, $CDCl_3$) of 3.1 160
Figure S105. ^{1}H NMR (600 MHz, $CDCl_3$) of 3.2 161
Figure S106. ^{13}C NMR (150 MHz, $CDCl_3$) of 3.2 162
Figure S107. ^{1}H NMR (600 MHz, $CDCl_3$) of 3.3 163
Figure S108. ^{13}C NMR (150 MHz, $CDCl_3$) of 3.3 164
Figure S109. ^{1}H NMR (600 MHz, $CDCl_3$) of 3.4 165
Figure S110. ^{13}C NMR (150 MHz, $CDCl_3$) of 3.4 166
Figure S111. ^{1}H NMR (600 MHz, $CDCl_3$) of 3.5 167
Figure S112. ^{13}C NMR (150 MHz, $CDCl_3$) of 3.5 168
Figure S113. ^{1}H NMR (600 MHz, $CDCl_3$) of 4.2 169
Figure S114. ^{13}C NMR (150 MHz, $CDCl_3$) of 4.2 170
Figure S115. ^{1}H NMR (600 MHz, $CDCl_3$) of 4.3 171

Figure S116. ^{13}C NMR (150 MHz, $CDCl_3$) of 4.3 172
Figure S117. ^{1}H NMR (600 MHz, $CDCl_3$) of 4.4 173
Figure S118. ^{13}C NMR (150 MHz, $CDCl_3$) of 4.4 174
Figure S119. ^{1}H NMR (600 MHz, $CDCl_3$) of 4.5 175
Figure S120. ^{13}C NMR (150 MHz, $CDCl_3$) of 4.5 176
Figure S121. ^{1}H NMR (600 MHz, $CDCl_3$) of 4.6 177
Figure S122. ^{13}C NMR (150 MHz, $CDCl_3$) of 4.6 178
Figure S123. ^{1}H NMR (600 MHz, $CDCl_3$) of 5.2 179
Figure S124. ^{13}C NMR (150 MHz, $CDCl_3$) of 5.2 180
Figure S125. ^{1}H NMR (600 MHz, $CDCl_3$) of 5.3 181
Figure S126. ^{13}C NMR (150 MHz, $CDCl_3$) of 5.3 182
Figure S127. ^{1}H NMR (600 MHz, $CDCl_3$) of 5.4 183
Figure S128. ^{13}C NMR (150 MHz, $CDCl_3$) of 5.4 184
Figure S129. ^{1}H NMR (600 MHz, $CDCl_3$) of 5.5 185
Figure S130. ^{13}C NMR (150 MHz, $CDCl_3$) of 5.5 186

Experimental Section

General Information: Glassware was flame-dried under vacuum for all non-aqueous reactions. All reagents and solvents were commercial grade and purified prior to use when necessary. Toluene, THF and dichloromethane (CH_2Cl_2) were dried by passage through a column of activated alumina as described by Grubbs.[1] Flash column chromatography was performed using Sorbent Technologies 230-400 mesh silica gel with solvent systems indicated. Analytical thin layer column chromatography was performed using Sorbent Technologies 250 µm glass backed UV254 silica gel plates and were visualized by fluorescence upon 250 nm radiation and/or the by use of TLC stain. Solvent removal was effected by rotary evaporation under vacuum (~ 25-40 mm Hg). All extracts were dried with Na_2SO_4 unless otherwise noted. Preparative HPLC was performed on an Agilent 1260 system (column: Zorbax Eclipse XDB-C18; 21.2 mm x 150 mm, 5 µm, flow rate 8 mL/min) with 210 nm monitoring wavelength and acetonitrile/water (+0.1% TFA) gradient as indicated. Nuclear magnetic resonance spectra (NMR) were acquired on a Bruker AV-400 (400 MHz) or Bruker AV II-600 (600 MHz) instrument. Mass spectra were recorded by use of electron impact ionization (EI), or electro-spray ionization (ESI) on a high resolution TQ-Orbitrap 3 XL Penn or Orbitrap 2 Classic FPG in the Vanderbilt Mass Spectrometry Core Laboratory. IR spectra were recorded on a Nicolet Avatar 360 spectrophotometer and are reported in wavenumbers (cm^{-1}) as neat films on a NaCl plate (transmission). Melting points were measured using an OptiMelt automated melting point system (Stanford Research Systems) and are not corrected. Chiral HPLC analysis was conducted on an Agilent 1200 series Infinity instrument using a ChiralPak column. Optical rotations were measured on a Jasco P-2000 polarimeter.

Purity Statement: Although each macrocycle was purified by preparative HPLC, and detected by UV (210 nm), the compounds reported often lack a good chromophore. However, the use of HPLC in combination with NMR analysis led us to judge compounds to be a minimum of 90% pure, likely >95%. Images of NMR spectra of all new compounds are provided.

Biological Experiments: All animal studies were approved (protocol number M1900081) and carried out in accordance with the guidelines and procedures set forth by the Vanderbilt Division of Animal Care. Cardiomyocytes were isolated from C57Bl/6J mice, as previously reported.[2] Calcium spark experiments were conducted, as previously reported.[3] Briefly, ventricular cardiomyocytes were adhered to laminin-coated glass bottom culture dishes, permeabilized with saponin, and incubated with an internal solution containing the calcium sensitive dye, Fluo-4. Elementary spark recordings were made using an Olympus inverted microscope equipped with a solid-state diode laser at 488 nm for excitation, 40× silicone objective (1.25 NA), and Hamamatsu CMOS camera for detection. The internal solution contained vehicle or compound and all data were collected 15 – 30 minutes after drug application. Spark detection analysis was performed using SparkMaster2.[4] Statistical analysis was carried out in R using a hierarchical clustering model to account for mouse-to-

---

[1] Pangborn, A. B.; Giardello, M. A.; Grubbs, R. H.; Rosen, R. K.; Timmers, F. J. Safe and convenient procedure for solvent purification. *Organometallics* **1996,** *15*, (5), 1518.

[2] Batiste, S. M.; Blackwell, D. J.; Kim, K.; Kryshtal, D. O.; Gomez-Hurtado, N.; Rebbeck, R. T.; Cornea, R. L.; Johnston, J. N.; Knollmann, B. C. Unnatural verticilide enantiomer inhibits type 2 ryanodine receptor-mediated calcium leak and is antiarrhythmic. *Proc. Natl. Acad. Sci. U. S. A.* **2019,** *116*, 4810.

[3] Smith, A. N.; Blackwell, D. J.; Knollmann, B. C.; Johnston, J. N. Ring Size as an Independent Variable in Cyclooligomeric Depsipeptide Antiarrhythmic Activity. *ACS Med. Chem. Lett.* **2021,** *12*, (12), 1942.

[4] Tomek, J.; Nieves-Cintron, M.; Navedo, M. F.; Ko, C. Y.; Bers, D. M. SparkMaster 2: A New Software for Automatic Analysis of Calcium Spark Data. *Circ Res* **2023,** *133*, (6), 450. .

mouse variability provided by Sikkel et al.[5] Bonferroni-adjusted P values are reported in the figure legends. A cutoff value of 0.05 was used as the threshold to reject the null hypothesis.

Safety: No unexpected or unusually high safety hazards were encountered.

Biological Experiment Data: Calcium spark parameters

For all series, assay is run with 25 µM solution of drug. Values are normalized to the vehicle (DMSO) condition and reported as mean ± standard deviation. Statistical comparisons were made using a hierarchical clustering model with Bonferroni correction. Adjusted p-values < 0.05 vs vehicle are indicated with an asterisk. Each mouse was an independent experiment, the replicates are listed as n (cells).

**Series 1**	**Vehicle (DMSO)**	***ent*-verticilide (1)**	**1.1**	**1.2**	**1.3**	**1.4**	**1.5**
**N (mice)**	12	2	5	5	5	4	4
**n (cells)**	292	29	102	105	108	119	87
**Frequency**	1.00 ± 0.28	0.51 ± 0.36*	1.03 ± 0.27	0.92 ± 0.28	0.96 ± 0.29	0.90 ± 0.36	1.00 ± 0.31
**Amplitude**	1.00 ± 0.26	0.82 ± 0.43*	1.01 ± 0.25	0.99 ± 0.24	1.04 ± 0.28	0.93 ± 0.24	1.00 ± 0.25
**Mass**	1.00 ± 1.15	0.057 ± 0.47*	0.98 ± 0.59	0.95 ± 0.51	0.91 ± 0.48	0.84 ± 0.32	1.00 ± 0.32
**Leak**	1.00 ± 1.20	0.36 ± 0.38*	1.02 ± 0.71	0.91 ± 0.63	0.90 ± 0.60	0.77 ± 0.53	0.91 ± 0.46

Supplement Table 1. Calcium spark parameters from murine cardiomyocytes for ent*-verticilide and series 1 analogs.*

**Series 2**	**2.1**	**2.2**	**2.3**	**2.4**	**2.5**
**N (mice)**	2	2	2	2	2
**n (cells)**	38	46	54	50	50
**Frequency**	0.51 ± 0.25	0.83 ± 0.20	1.01 ± 0.20	0.97 ± 0.17	1.04 ± 0.20
**Amplitude**	0.93 ± 0.31	1.05 ± 0.24	0.92 ± 0.19	1.08 ± 0.22	1.17 ± 0.34
**Mass**	1.04 ± 0.64	0.95 ± 0.39	1.02 ± 0.43	0.98 ± 0.39	1.06 ± 0.35
**Leak**	0.60 ± 0.65	0.79 ± 0.44	1.03 ± 0.53	0.97 ± 0.56	1.09 ± 0.46

Supplement Table 2. Calcium spark parameters from murine cardiomyocytes for series 2 analogs.

**Series 3**	**3.1**	**3.2**	**3.3**	**3.4**	**3.5**
**N (mice)**	4	3	4	5	5
**n (cells)**	79	58	79	80	91
**Frequency**	0.79 ± 0.29	0.97 ± 0.28	0.96 ± 0.26	1.15 ± 0.26	0.94 ± 0.25
**Amplitude**	0.99 ± 0.20	1.02 ± 0.20	1.00 ± 0.16	0.99 ± 0.18	0.97 ± 0.19
**Mass**	0.81 ± 0.55	1.03 ± 0.50	0.96 ± 0.42	0.98 ± 0.45	1.07 ± 0.64
**Leak**	0.75 ± 0.63	0.99 ± 0.53	0.93 ± 0.45	1.20 ± 0.63	1.04 ± 0.71

Supplement Table 3. Calcium spark parameters from murine cardiomyocytes for series 3 analogs.

[5] Sikkel, M. B.; Francis, D. P.; Howard, J.; Gordon, F.; Rowlands, C.; Peters, N. S.; Lyon, A. R.; Harding, S. E.; MacLeod, K. T. Hierarchical statistical techniques are necessary to draw reliable conclusions from analysis of isolated cardiomyocyte studies. *Cardiovasc. Res.* **2017,** *113*, (14), 1743.

Series 4	4.1	4.2	4.3	4.4	4.5	4.6
N (mice)	4	2	2	2	2	2
n (cells)	66	42	37	38	43	37
Frequency	0.96 ± 0.28	0.87 ± 0.29	1.08 ± 0.27	1.02 ± 0.24	1.16 ± 0.30	1.08 ± 0.36
Amplitude	1.01 ± 0.31	1.24 ± 0.35	1.32 ± 0.31	1.16 ± 0.31	1.17 ± 0.22	1.15 ± 0.22
Mass	1.19 ± 1.03	1.57 ± 0.89	1.66 ± 0.94	1.42 ± 1.09	1.29 ± 0.40	1.10 ± 0.52
Leak	1.19 ± 1.10	1.35 ± 1.14	1.79 ± 1.49	1.38 ± 1.21	1.43 ± 0.66	1.15 ± 0.71

Supplement Table 4. Calcium spark parameters from murine cardiomyocytes for series 4 analogs.

Series 5	5.1	5.2	5.3	5.4	5.5	5.6
N (mice)	4	5	5	5	5	4
n (cells)	66	85	93	100	89	67
Frequency	0.96 ± 0.28	0.79 ± 0.35	0.94 ± 0.31	0.89 ± 0.29	1.02 ± 0.33	1.11 ± 0.29
Amplitude	1.01 ± 0.31	1.13 ± 0.23	1.08 ± 0.18	1.02 ± 0.21	1.06 ± 0.18	1.07 ± 0.19
Mass	1.19 ± 1.03	1.11 ± 0.47	1.17 ± 0.47	1.13 ± 0.69	0.96 ± 0.42	1.30 ± 0.75
Leak	1.19 ± 1.10	1.08 ± 0.66	1.23 ± 0.57	1.14 ± 0.69	1.14 ± 0.65	1.54 ± 1.17

Supplement Table 5. Calcium spark parameters from murine cardiomyocytes for series 5 analogs.

General Procedures

***tert*-Butyloxycarbonyl (Boc) Deprotection**: A round-bottom flask was charged with the depsipeptide (1 equiv) and dissolved in either 4 M HCl/ethyl acetate (1 M in depsipeptide) or 20% TFA in DCM. The reaction was allowed to stir for 1-3 h at ambient temperature. The crude reaction mixture was concentrated, ether was added, and the mixture was then reconcentrated. This procedure was repeated 3 times with diethyl ether.

Benzyl Deprotection: A round-bottom flask was charged with the depsipeptide (1 equiv) dissolved in methanol or ethyl acetate (0.1 M) and treated with 10% Pd/C (20 mol%). The reaction flask was evacuated with a light vacuum (~40 Torr). Hydrogen (balloon) was added and then the flask was cycled through a light vacuum three times. The reaction was stirred for 1.5-3 h. After purging with argon, the crude reaction mixture was filtered through Celite and concentrated to afford the carboxylic acid.

PyBrop Coupling Reaction: A round-bottom flask was charged with the two depsipeptide coupling partners (1:1), along with dry DCM or DMF (0.1 M). The solution was cooled to 0 °C and placed under argon. PyBrop (1.5 equiv) and freshly distilled DIPEA (3 equiv) were added. The reaction was allowed to stir for 30 minutes at 0 °C and 1.5-18 h at ambient temperature. Reaction progression was monitored by TLC and/or TLC-MS. The reaction mixture was poured into cold 10% aq citric acid and extracted with EtOAc. The combined organic layers were washed with satd aq $NaHCO_3$, brine, dried, and concentrated. The crude residue was subjected to flash column chromatography.

PyBOP Macrocyclization: The reaction mixture was added to a flame-dried round bottom flask. Dry DMF or DCM (0.005 M) was added and the reaction was cooled to 0 °C. Once at 0 °C, freshly distilled DIPEA (3 equiv) and PyBop (1.05 equiv) were added. The reaction was stirred at 0 °C for 30 minutes, then allowed to warm to ambient temperature and stir for an additional 1.5-3 h. Reaction progression was monitored by TLC and/or TLC-MS. The reaction mixture was poured into cold 10% aq citric acid and extracted with EtOAc. The combined organic layers were washed with satd aq $NaHCO_3$, brine, and then dried and concentrated. The crude reaction mixture was purified via preparative HPLC.

HATU Coupling Reaction: A round-bottom flask was charged with the amine and acid (1:1), and dry DMF or DCM (0.1 M). The mixture was cooled to 0 °C and then freshly distilled DIPEA (3 equiv) and HATU (3 equiv) were added. The reaction was stirred at 0 °C for 30 min, then warmed to 23 °C for 3-18 h. Reaction progression was monitored by TLC and/or TLC-MS. The reaction mixture was poured into cold 10% aq citric acid and extracted with EtOAc. The combined organic layers were washed with satd aq $NaHCO_3$, brine, dried, and concentrated. The crude residue was subjected to flash column chromatography.

General Reaction Schemes

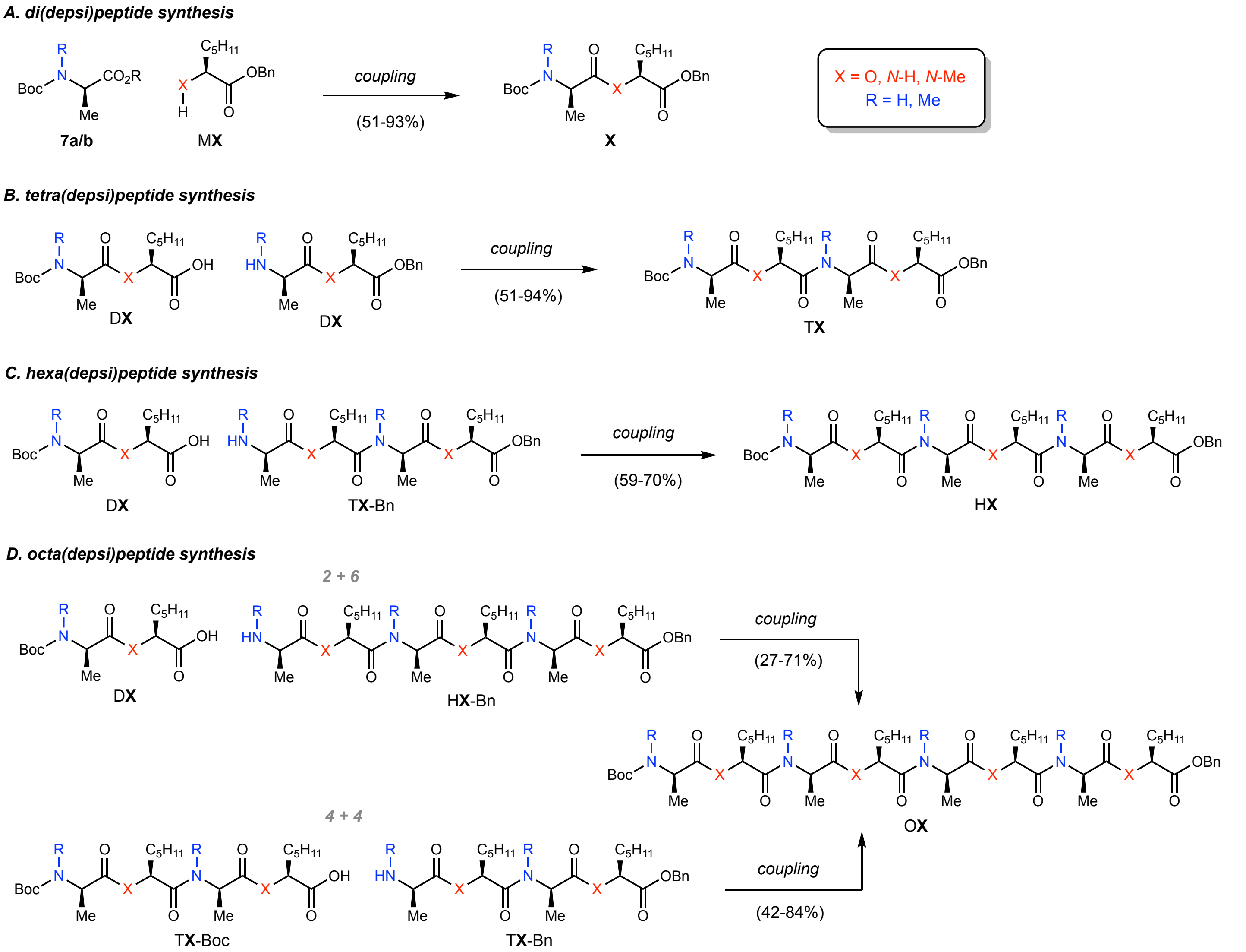

Scheme 1. A-D. Generalized reaction schemes for the synthesis of various intermediates. Individual reaction conditions can be found in the Experimental Procedures section.

Experimental Procedures and Characterization Data for Reported Compounds

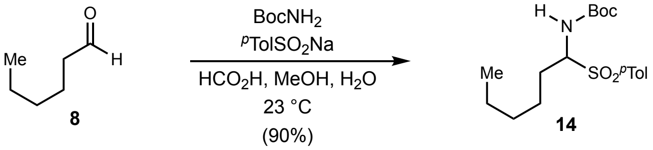

1-Tosylhexan-1-amine (14). A round-bottom flask was charged with hexanal (9.0 mL, 73 mmol), *tert*-butyl carbamate (5.7 g, 49 mmol), and MeOH (73 mL) and stirred until it became a homogenous solution. $NaSO_2{}^{p}Tol$ (17.4 g, 97.6 mmol) was added, along with enough H_2O to dissolve the solids (100 mL). Then formic acid (3.68 mL, 97.6 mmol) was added, and the mixture was allowed to stir at ambient temperature under argon for 4 d. The reaction mixture was filtered and washed with H_2O and hexanes to afford the product as a white solid (15.5 g, 90%). Mp 107- 110 °C; R*f* = 0.26 (10% EtOAc/hexanes); IR (film) 3334, 2958, 2930, 2861, 1721, 1597, 1518, 1456, 1392, 1316, 1245, 1167, 1142, 1085 cm^{-1}; ^1H NMR (600 MHz, $CDCl_3$) The small doubling of peaks is due to cis and trans amide rotamers. The largely favored isomer is listed here: δ 7.76 (d, *J* = 8.8 Hz, 2H), 7.30 (d, *J* = 7.9 Hz, 2H), 5.10 (d, *J* = 11.0 Hz, 1H), 4.79 (td, *J* = 10.9, 3.4 Hz, 1H), 2.38 (s, 3H), 2.24-2.16 (m, 1H), 1.74-1.65 (m, 1H), 1.55-1.23 (m, 6H), 1.19 (s, 9H) 0.86 (t, *J* = 6.5 Hz, 3H); ^{13}C NMR (150 MHz, $CDCl_3$) ppm 154.0, 144.9, 134.0, 129.7, 129.4, 80.6, 31.2, 28.0, 27.7, 26.4, 25.0, 22.4, 21.7, 14.0; HRMS (EI): Exact mass calcd for $C_{18}H_{29}NNaO_4S$ $[M+Na]^+$ 378.1710, found 378.1719.

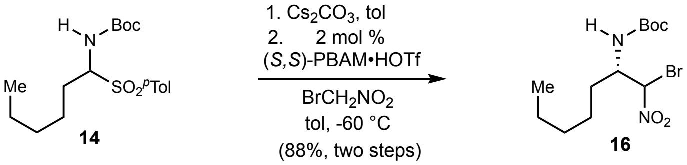

***tert*-Butyl ((2*S*)-1-bromo-1-nitroheptan-2-yl)carbamate (16).** A round-bottom flask was charged with sulfone (6.00 g, 16.8 mmol), Cs_2CO_3 (27.5 g, 84.4 mmol), and toluene (160 mL). The mixture was allowed to stir at ambient temperature under argon for 6 h. The reaction mixture was filtered through a pad of Celite and concentrated. The crude oil was then dissolved in toluene (248 mL) and to it was added (*S*,*S*)-PBAM as its triflic acid salt (217.5 mg, 331 µmol). The reaction was cooled to -60 °C and bromonitromethane (1.74 mL, 24.8 mmol) was added. The reaction was allowed to stir for 48 h. The reaction mixture was quenched by running it through a short silica plug while still cold, with 100% EtOAc. The fractions were combined and concentrated. The crude residue was subjected to flash column chromatography (SiO_2, 5% ethyl acetate in hexanes) to afford the α-bromo nitroalkane (1:1 dr (^1H NMR)) as a white solid (4.5 g, 88% 2-step); The diastereomers were determined to be 90% and 89% ee by chiral HPLC analysis (Chiralcel AD-H, 1% ^{i}PrOH /hexanes, 1.0 mL/min, *t*r(*d1*, major) = 19.6 min, *t*r(*d1*, minor) = 21.1 min, *t*r(*d2*, minor) = 25.1 min, *t*r(*d2*, major) = 29.4 min). $[\alpha]_D^{23}$ -20 (*c* 0.53, $CHCl_3$); Mp 62-66 °C; R*f* = 0.60 (10% EtOAc/hexanes); IR (film) 3332, 2958, 2931, 2861, 1706, 1567, 1501, 1458, 1392, 1367, 1248, 1165, 1045 cm^{-1}; ^1H NMR (600 MHz, $CDCl_3$, 1:1 mixture of diastereomers) δ 6.19 (d, *J* = 4.4 Hz, 1H), 6.17 (d, *J* = 3.0 Hz, 1H), 4.83 (d, *J* = 8.8 Hz, 1H), 4.73 (d, *J* = 9.1 Hz, 1H), 4.34 (dddd, *J* = 9.2, 9.2, 3.9, 3.9 Hz, 1H), 4.24 (dddd, *J* = 9.2, 9.2, 4.6, 4.6 Hz, 1H), 1.90-1.52 (series of m, 4H), 1.46 (s, 9H), 1.44 (s, 9H), 1.40-1.22 (m, 12H), 0.90 (t, *J* = 7.0 Hz, 3H), 0.88 (t, *J* = 6.9 Hz, 3H); ^{13}C NMR (150 MHz, $CDCl_3$, 1:1 mixture of diastereomers) ppm 155.2, 155.1, 84.5, 83.2, 80.9, 80.8, 55.0, 54.5, 31.32, 31.28, 30.5, 28.5, 28.4, 28.3, 25.6, 25.5, 22.52, 22.51, 14.0(2C); HRMS (EI): Exact mass calcd for $C_{12}H_{23}BrN_2NaO_4$ $[M+Na]^+$ 361.0733, found 361.0737.

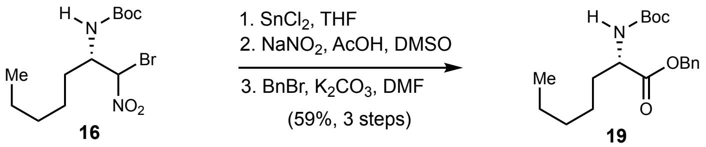

Benzyl (*S*)-2-((*tert*-butoxycarbonyl)amino)heptanoate (19). A flame-dried round-bottom flask was charged with halo-nitro alkane (383 mg, 1.13 mmol), tin chloride (428 mg, 2.26 mmol), and THF (11.3 mL). The mixture was allowed to stir at ambient temperature under argon for 12 h. The reaction mixture was then poured into H_2O, and diethyl ether was added into the separatory funnel. The organic layer was filtered through a pad of Celite, washed with H_2O (three times), dried, and concentrated to afford a pale-yellow oil. The crude oil was then dissolved in DMSO (8.7 mL) and to it was added $NaNO_2$ (234 mg, 3.39 mmol) and AcOH (885 µL, 17.0 mmol). The reaction was heated to 60 °C and allowed to stir for 12 h. The reaction mixture was allowed to cool to ambient temperature, quenched with 1 M aq HCl, poured into a separatory funnel, and extracted with EtOAc. The organic layers were washed with ice water (four times), dried, and concentrated. The crude reaction mixture was dissolved in DMF (2.1 mL), and to it was added K_2CO_3 (427 mg, 3.09 mmol) and BnBr (148 µL, 1.24 mmol). The reaction was allowed to stir at ambient temperature under argon for 12 h. The reaction was quenched with 1 M aq HCl and then extracted with EtOAc. The organic layers were then dried and concentrated. The crude residue was subjected to flash column chromatography (SiO_2, 10% ethyl acetate in hexanes) to afford the product as a colorless oil (366 mg, 59% (3 steps)); $[\alpha]_D^{23}$ -5.39 (*c* 1.08, $CHCl_3$); R*f* = 0.33 (10% EtOAc/hexanes); IR (film) 3365, 2957, 2931, 2861, 1717, 1499, 1456, 1366, 1249, 1161, 1048 cm^{-1}; ^1H NMR (400 MHz, $CDCl_3$) δ 7.40-7.28 (m, 5H), 5.21 (d, *J* = 12.4 Hz, 1H), 5.12 (d, *J* = 12.4 Hz, 1H), 5.02 (d, *J* = 7.8 Hz, 1H), 4.35 (ddd, *J* = 11.7, 6.2, 6.2 Hz, 1H), 1.85-1.70 (m, 1H), 1.68-1.55 (m, 1H), 1.43 (s, 9H), 1.36-1.17 (m, 6H), 0.85 (t, *J* = 6.3 Hz, 3H); ^{13}C NMR (100 MHz, $CDCl_3$) ppm 172.9, 156.5, 136.6, 128.7, 128.4, 128.3, 79.8, 66.9, 53.7, 32.7, 31.4, 28.4, 24.9, 22.5, 14.0; HRMS (EI): Exact mass calcd for $C_{19}H_{29}NNaO_4$ $[M+Na]^+$ 358.1989, found 358.1993.

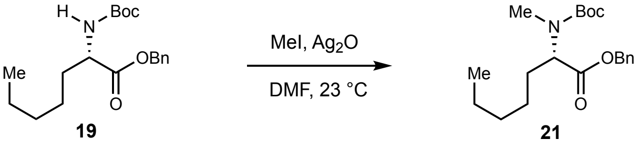

Benzyl (*S*)-2-((*tert*-butoxycarbonyl)(methyl)amino)heptanoate (21). A flame-dried round-bottom flask was charged with the α-amino ester (266 mg, 793 µmol), MeI (789 µg, 12.69 mmol), Ag_2O (735 mg, 3.17 mmol) and DMF (7.9 mL). The reaction vessel was wrapped in aluminum foil, and the mixture was allowed to stir at ambient temperature under argon for 12 h. The reaction mixture was filtered through a pad of Celite and concentrated, and then dissolved in EtOAc, washed with H_2O, dried, and concentrated to afford a pale-yellow oil. The crude residue was subjected to flash column chromatography (SiO_2, 5% ethyl acetate in hexanes) to afford the product as a colorless oil (244 mg, 87%);$[\alpha]_D^{23}$ -20.6 (*c* 1.09, $CHCl_3$); R*f* = 0.57 (10% EtOAc/hexanes); IR (film) 3033, 2931, 2860, 1743, 1697, 1455, 1391, 1366, 1327, 1149 cm^{-1}; ^1H NMR (600 MHz, $CDCl_3$) Several peaks show a clear doubling for the *cis* and *trans* amide products. Other peaks are overlapping and appear only once. Those that are doubled have both values listed. δ 7.40-7.28 (m, 5H), 5.15 (broad s, 2H), 4.80/4.47 (dd, *J* = 10.5, 4.8 Hz, 1H), 2.82/2.75 (s, 3H), 1.97-1.86 (m, 1H), 1.79-1.63 (m, 1H), 1.45/1.40 (s, 9H), 1.36-1.23 (m, 6H), 0.88/0.87 (t, *J* = 6.5 Hz, 3H); ^{13}C NMR (150 MHz, $CDCl_3$) ppm) The line listing for both *cis* and *trans* amide isomers is given, as a clear doubling of many peaks is evident. Whenever the isomer peaks overlap, it is stated that the particular peak is representing 2 or more carbons. 172.2, 172.0, 156.5, 155.8, 136.0, 135.8, 128.7, 128.6, 128.4, 128.2, 128.1, 128.0, 80.3, 80.0, 66.7, 66.6, 59.3, 57.9, 31.4(2C), 30.9, 30.5, 29.1, 28.7, 28.5, 28.4, 25.8(2C), 22.6(2C), 14.1, 14.0; HRMS (EI): Exact mass calcd for $C_{20}H_{31}NNaO_4$ $[M+Na]^+$ 372.2145, found 372.2149.

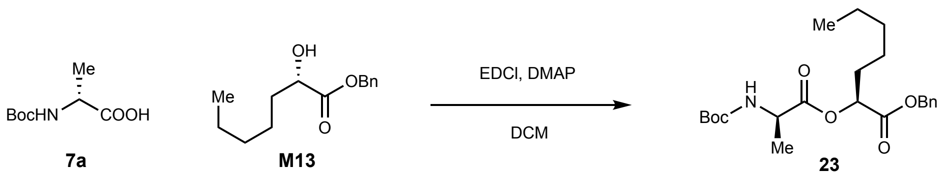

Benzyl (*S*)-2-(((*tert*-butoxycarbonyl)-*D*-alanyl)oxy)heptanoate (23). A round-bottom flask was charged with the amine (1.98 g, 9.73 mmol), alcohol (2.00 g, 8.46 mmol), and DCM (84.6 mL). The mixture was cooled to 0 °C and then EDCI (1.95 g, 10.2 mmol) and DMAP (51.7 mg, 424 μmol) were added. The reaction mixture was stirred at 0 °C for 30 min, and then allowed to warm to ambient temperature and stir for an additional 1.5 h before it was poured into water and extracted with DCM. The combined organic layers were washed with satd aq $NaHCO_3$, brine, dried, and concentrated. The crude residue was subjected to flash column chromatography (SiO_2, 2.5-20% ethyl acetate in hexanes) to afford the product as a white amorphous solid (2.54 g, 88%). $[\alpha]_D^{24}$ –11.2 (*c* 0.43, $CHCl_3$); R_f = 0.29 (10% EtOAc/hexanes); IR (film) 2954, 2926, 2856, 1745, 1666, 1455, 1408, 1306, 1212, 1107, 1076 cm^{-1}; ^1H NMR (600 MHz, $CDCl_3$) δ 7.39-7.31 (series of m, 5H), 5.20 (d, *J* = 12.1 Hz, 1H), 5.14 (d, *J* = 12.1 Hz, 1H), 5.07 (t, *J* = 3.7 Hz, 1H), 5.02 (br m, 1H), 4.46-4.36 (br m, 1H), 1.89-1.82 (br m, 2H), 1.44 (s, 9H), 1.40 (br d, *J* = 7.3 Hz, 3H), 1.37-1.33 (br m, 2H), 1.28-1.24 (br m, 4H), 0.88 (br t, *J* = 6.8 Hz, 3H); ^{13}C NMR (150 MHz, $CDCl_3$) ppm 172.7, 169.7, 155.9, 135.2, 128.6, 128.4, 128.3, 72.9, 70.5, 67.3, 67.0, 49.5, 31.2, 30.9, 28.3, 24.7, 18.6, 14.0; HRMS (EI): Exact mass calcd for $C_{22}H_{33}NO_6Na$ $[M+Na]^+$ 430.2206, found 430.2203. MPT-2-139.

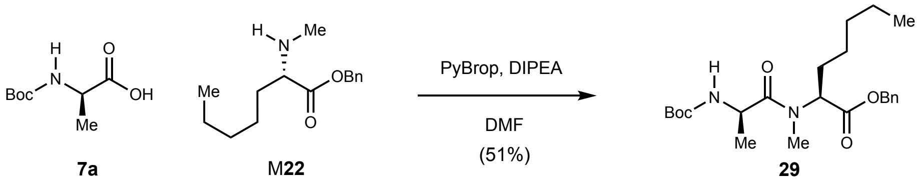

Benzyl (*S*)-2-((*R*)-2-((*tert*-butoxycarbonyl)amino)-*N*-methylpropanamido)heptanoate (29). A round-bottom flask was charged with the amine (1.20 g, 4.80 mmol), acid (908 mg, 4.80 mmol), and DMF (4.2 mL). The mixture was cooled to 0 °C and then DIPEA (2.52 mL, 14.4 mmol) and PyBrop (3.52 g, 7.20 mmol) were added. The reaction was stirred at 0 °C for 30 min, then warmed to room temperature and stirred overnight. The reaction mixture was poured into cold 10% aq citric acid and extracted with EtOAc. The combined organic layers were washed with satd aq $NaHCO_3$, brine, dried, and concentrated. The crude residue was subjected to flash column chromatography (SiO_2, 5-30% ethyl acetate in hexanes) to afford the product as a white amorphous solid (1.07 g, 51%). $[\alpha]_D^{24}$ + 7.2 (*c* 0.44, $CHCl_3$); R_f = 0.60 (25% EtOAc/hexanes); IR (film) 2930, 1744, 1698, 1456, 1390, 1156 cm^{-1}; ^1H NMR (600 MHz, $CDCl_3$) δ 7.38-7.32 (series of m, 5H), 6.85 (br d, *J* = 7.2 Hz, 1H), 5.25-5.21 (series of m, 2H), 5.03 (m, 1H), 4.70-4.21 (m, 1H), 3.16-2.62 (series of m, 3H), 1.84 (br m, 1H), 1.58 (br m, 2H), 1.45 (series of m, 9H), 1.36 (m, 2H), 1.28-1.23 (series of m, 6H), 0.86 (m J = 6.9 Hz, 3H); ^{13}C NMR (150 MHz, $CDCl_3$) ppm 175.3, 171.0, 156.1, 135.3, 128.9, 128.4, 128.6, 128.5, 79.9, 72.8, 67.8, 52.8, 34.4, 31.9, 29.7, 28.4, 24.9, 22.3, 13.9; HRMS (EI): Exact mass calcd for $C_{23}H_{37}N_2O_5$ $[M+H]^+$ 421.2702, found 421.2700. MPT-4-163.

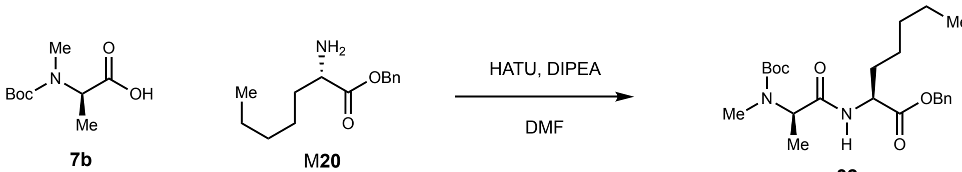

Benzyl (*S*)-2-((*R*)-2-((*tert*-butoxycarbonyl)(methyl)amino)propanamido)heptanoate (32). A round-bottom flask was charged with the amine (990 mg, 4.21 mmol), acid (855 mg, 4.21 mmol), and DMF (4.2 mL). The mixture was cooled to 0 °C and then DIPEA (2.20 mL, 12.6 mmol) and HATU (2.40 g, 6.32 mmol) were added. The reaction was stirred at 0 °C for 30 min, then warmed to room temperature and stirred overnight. The reaction mixture was poured into cold 10% aq citric acid and extracted with EtOAc. The combined organic layers were washed with satd aq $NaHCO_3$, brine, dried, and concentrated. The crude residue was subjected to flash column chromatography (SiO_2, 5-30% ethyl acetate in hexanes) to afford the product as a white amorphous solid (1.15 g, 74%). $[\alpha]_D^{24}$ + 29 (*c* 0.43, $CHCl_3$); R_f = 0.42 (30% EtOAc/hexanes); IR (film) 3330, 2957, 2932, 2861, 1739, 1692, 1525, 1456, 1368, 1154, 1091, 1047 cm^{-1}; ^1H NMR (600 MHz, $CDCl_3$) δ 7.37-7.32 (series of m, 5H), 6.72 (br m, 1H), 5.15-3.77 (series of m, 4H), 3.03-2.71 (series of m, 3H), 1.82 (br m, 1H), 1.65 (br m, 1H), 1.48-1.43 (series of m, 9H), 1.32-1.23 (series of m, 9H), 0.85 (br t, *J* = 6.9 Hz, 3H); ^{13}C NMR (150 MHz, $CDCl_3$) ppm 172.8, 172.2, 159.9, 130.7, 129.1, 128.9, 128.7, 81.0, 67.6, 52.8, 46.3, 32.7, 31.9, 30.4, 25.6, 25.5, 24.9, 23.0, 14.5; HRMS (EI): Exact mass calcd for $C_{23}H_{37}N_2O_5$ $[M+H]^+$ 421.2702, found 421.2684. MPT-4-135

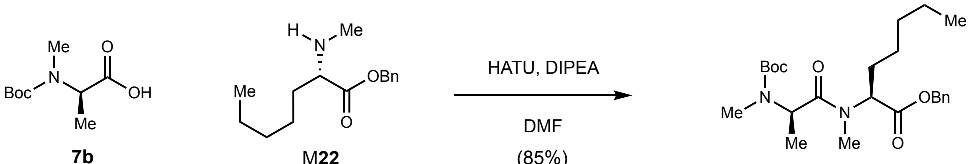

Benzyl (*S*)-2-((*R*)-2-((*tert*-butoxycarbonyl)(methyl)amino)-*N*-methylpropanamido)heptanoate (35). A round-bottom flask was charged with the amine (900 mg, 3.61 mmol), acid (734 mg, 3.61 mmol), and DMF (36.1 mL). The mixture was cooled to 0 °C and then DIPEA (1.89 mL, 10.8 mmol) and HATU (4.11 g, 10.8 mmol) were added. The reaction was stirred at 0 °C for 30 min, then warmed to room temperature and stirred overnight. The reaction mixture was poured into cold 10% aq citric acid and extracted with EtOAc. The combined organic layers were washed with satd aq $NaHCO_3$, ice water, dried, and concentrated. The crude residue was subjected to flash column chromatography (SiO_2, 5-20% ethyl acetate in hexanes) to afford the product as a white amorphous solid (1.33 g, 85%). $[\alpha]_D^{23}$ + 50 (*c* 0.34, $CHCl_3$); R_f = 0.20 (10% EtOAc/hexanes); IR (film) 2957, 2931, 2861, 1740, 1659, 1456, 1390, 1156 cm^{-1}; ^1H NMR (600 MHz, $CDCl_3$) δ 7.37-7.29 (series of m, 5H), 5.25-4.51 (series of m, 4H), 2.92-2.55 (series of m, 6H), 1.98 (m, 1H), 1.69 (m, 1H), 1.46 (s, 9H), 1.34-1.20 (series of m, 9H), 0.88 (t, *J* = 6.0 Hz, 3H); ^{13}C NMR (150 MHz, $CDCl_3$) ppm 171.9, 171.3, 155.3, 135.6, 128.6, 128.4, 128.2, 80.0, 66.9, 56.9, 50.9, 31.3, 30.5, 29.0, 28.7, 28.4, 28.0, 25.7, 22.4, 13.9; HRMS (EI): Exact mass calcd for $C_{24}H_{38}N_2NaO_5$ $[M+Na]^+$ 457.2678, found 457.2675. MPT-3-032

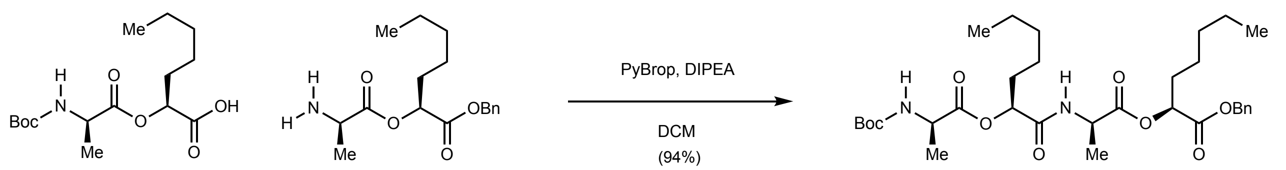

D**24** D**25** T**38**

Benzyl (*R*)-2-(((6*R*,9*R*,12*R*)-2,2,5,6,11,12-hexamethyl-4,7,10-trioxo-9-pentyl-3,8-dioxa-5,11-diazatridecan-13-oyl)oxy) heptanoate (T38). A round-bottom flask was charged with the amine (402 mg, 1.31 mmol), acid (415 mg, 1.31 mmol), and DCM (12.0 mL). The mixture was cooled to 0 °C and then DIPEA (683 µL, 3.92 mmol) and PyBrop (638 mg, 1.31 mmol) were added. The reaction was stirred at 0 °C for 1 h. The reaction mixture was poured into cold 10% aq citric acid and extracted with DCM. The combined organic layers were washed with satd aq $NaHCO_3$, brine, dried, and concentrated. The crude residue was subjected to flash column chromatography (SiO_2, 5-50% ethyl acetate in hexanes) to afford the product as a light yellow oil (745 mg, 94%). $[\alpha]_D^{24}$ –20.1 (*c* 0.62, $CHCl_3$); R_f = 0.52 (30% EtOAc/hexanes); IR (film) 3312, 2956, 2930, 2861, 1747, 1692, 1530, 1455, 1367, 1255, 1160, 1069 cm^{-1}; ^{1}H NMR (600 MHz, $CDCl_3$) δ 7.40-7.35 (series of m, 5H), 7.05 (br d, *J* = 5.3 Hz, 1H), 5.27-5.16 (series of m, 3H), 5.11 (br t, *J* = 6.5 Hz, 1H), 4.99 (br d, *J* = 5.3 Hz, 1H), 4.69 (dd, *J* = 7.1, 7.1 Hz, 1H), 4.25 (dd, *J* = 7.1, 7.1 Hz, 1H), 1.99-1.93 (b m, 1H), 1.88-1.80 (br m, 3H), 1.49-1.43 (series of m, 14H), 1.39-1.28 (series of m, 13H), 0.90-0.88 (series of m, 6H); ^{13}C NMR (150 MHz, $CDCl_3$) ppm 173.4, 172.5, 170.3, 169.9, 156.0, 135.9, 129.2, 129.0, 128.9, 128.8, 80.9, 74.9, 73.5, 67.9, 67.6, 50.4, 48.7, 32.2, 31.9, 31.8, 31.6, 28.9, 25.3, 23.0, 22.9, 18.2, 18.0, 14.5; HRMS (EI): Exact mass calcd for $C_{32}H_{50}N_2O_9Na$ [M+Na]$^+$ 629.3414, found 629.3409. MPT-2-151.

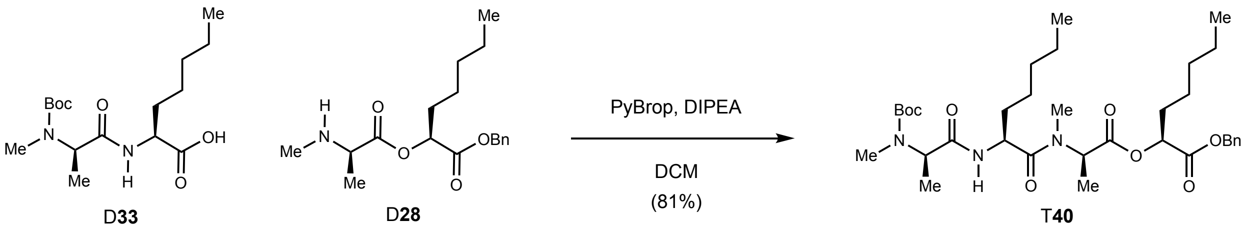

D**33** D**28** T**40**

(*S*)-1-(benzyloxy)-1-oxoheptan-2-yl (6*R*,9*S*,12*R*)-2,2,5,6,11,12-hexamethyl-4,7,10-trioxo-9-pentyl-3-oxa-5,8,11-triazatridecan-13-oate (T40). A round-bottom flask was charged with the amine (122 mg, 378 µmol), acid (125 mg, 378 µmol), and DCM (7.0 mL). The mixture was cooled to 0 °C and then DIPEA (198 µL, 1.13 mmol) and PyBrop (277 mg, 567 µmol) were added. The reaction was stirred at 0 °C for 30 min, then warmed to 23 °C for 2 h. The reaction mixture was poured into cold 10% aq citric acid and extracted with DCM. The combined organic layers were washed with satd aq $NaHCO_3$, brine, dried, and concentrated. The crude residue was subjected to flash column chromatography (SiO_2, 5-30% ethyl acetate in hexanes) to afford the product as a light yellow oil (195 mg, 81%). $[\alpha]_D^{24}$ +19 (*c* 0.83, $CHCl_3$); R_f = 0.36 (30% EtOAc/hexanes); IR (film) 2954, 2926, 2857, 1746, 1691, 1650, 1456, 1368, 1319, 1155, 1021 cm^{-1}; ^{1}H NMR (600 MHz, $CDCl_3$) δ 7.33-7.25 (series of m, 5H), 6.75 (br d, *J* = 7.3 Hz, 1H), 5.21-5.10 (series of m, 2H), 5.05 (m, 1H), 4.97 (br m, 1H), 4.86 (dt, *J* = 8.0, 7.9 Hz, 1H), 4.77 (m, 1H), 3.28-2.72 (series of m, 6H), 1.94 (br m, 1H), 1.81-1.72 (series of m, 3H), 1.69-1.61 (series of m, 2H), 1.44 (br s, 9H), 1.34-1.31 (br series of m, 4H) 1.27-1.17 (series of m, 12H), 0.90-0.88 (br t, *J* = 6.8 Hz, 3H), 0.90-0.88 (br t, *J* = 6.2 Hz, 3H); ^{13}C NMR (150 MHz, $CDCl_3$) ppm 171.1, 170.9, 169.8, 169.3, 153.2, 135.2, 128.7, 128.5, 128.3, 80.6, 73.4, 73.0, 67.3, 67.0, 55.0, 52.6, 49.1, 32.8, 31.6, 31.2, 30.8, 29.7, 29.4, 28.4, 24.8, 24.6, 22.5, 22.3, 14.4, 13.9; HRMS (EI): Exact mass calcd for $C_{34}H_{59}N_4O_8$ [M+NH$_4$]$^+$ 651.4327, found 651.4327. MPT-2-196

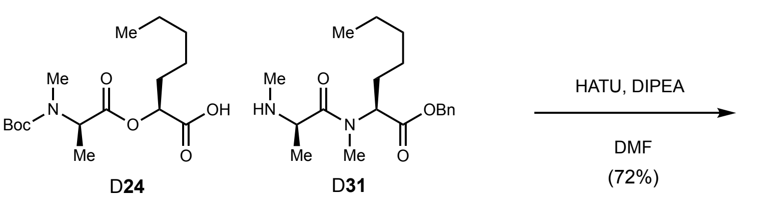

Benzyl (*S*)-2-((6*R*,9*S*,12*R*)-N,2,2,5,6,11,12-heptamethyl-4,7,10-trioxo-9-pentyl-3,8-dioxa-5,11-diazatridecan-13-amido)heptanoate (T41). A round-bottom flask was charged with the amine (158 mg, 474 µmol), acid (157 mg, 474 µmol), and DMF (4.7 mL). The mixture was cooled to 0 °C and then DIPEA (248 µL, 1.42 mmol) and HATU (540 mg, 1.42 mmol) were added. The reaction was stirred at 0 °C for 30 min, then warmed to 23 °C for 18 h. The reaction mixture was poured into cold 10% aq citric acid and extracted with EtOAc. The combined organic layers were washed with satd aq $NaHCO_3$, brine, dried, and concentrated. The crude residue was subjected to flash column chromatography (SiO_2, 5-40% ethyl acetate in hexanes) to afford the product as a light yellow foam (222 mg, 72%). $[\alpha]_D^{24}$ + 65 (*c* 0.87, $CHCl_3$); R_f = 0.33 (25% EtOAc/hexanes); IR (film) 2955, 2930, 2860, 1739, 1699, 1657, 1456, 1367, 1316, 1256, 1154 cm^1; ^1H NMR (600 MHz, $CDCl_3$) δ 7.38-7.32 (series of m, 5H), 5.47 (br m, 1H), 5.26 (br dd, *J* = 6.5, 6.5 Hz, 1H), 5.13 (m, 2H), 4.97 (dd, *J* = 8.0, 8.0 Hz, 1H), 2.92-2.60 (series of m, 9H), 2.03 (m, 1H), 1.91 (m, 1H), 1.73 (m, 1H), 1.61 (m, 1H), 1.49 (s, 9H) 1.40-1.19 (series of m, 18H), 0.90 (br t, *J* = 6.7 Hz, 3H), 0.88 (br t, *J* = 6.5 Hz, 3H); ^{13}C NMR (150 MHz, $CDCl_3$) ppm 172.2, 171.2, 170.8, 168.9, 156.1, 135.4, 128.9, 128.6, 128.4, 79.9, 71.3, 68.0, 67.2, 66.9, 57.9, 56.8, 54.4, 50.0, 31.3, 31.4, 30.3, 29.9, 29.1, 28.4, 27.7, 25.6, 24.9, 22.4, 21.9, 14.4, 14.0; HRMS (EI): Exact mass calcd for $C_{35}H_{58}N_3O_8$ $[M+H]^+$ 648.4218, found 648.4216. MPT-3-006

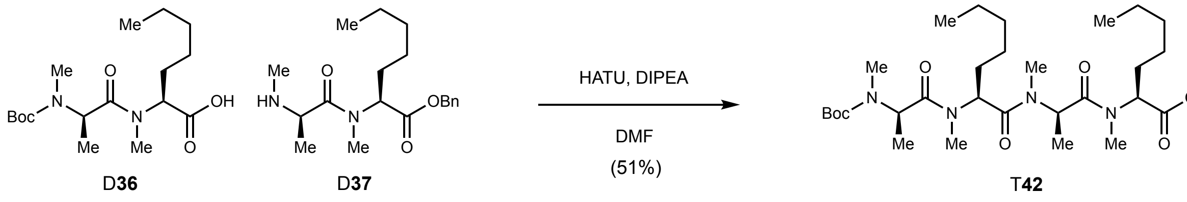

Benzyl (6*R*,9*S*,12*R*,15*S*)-2,2,5,6,8,11,12,14-octamethyl-4,7,10,13-tetraoxo-9,15-dipentyl-3-oxa-5,8,11,14-tetraazahexadecan-16-oate (T42). A round-bottom flask was charged with the amine (200 mg, 581 µmol), acid (200 mg, 581 µmol), and DMF (5.8 mL). The mixture was cooled to 0 °C and then DIPEA (304 µL, 1.74 mmol) and HATU (662 g, 2.76 mmol) were added. The reaction was stirred at 0 °C for 30 min, then warmed to 23 °C for 18 h. The reaction mixture was poured into cold 10% aq citric acid and extracted with EtOAc. The combined organic layers were washed with satd aq $NaHCO_3$, brine, dried, and concentrated. The crude residue was subjected to flash column chromatography (SiO_2, 5-40% ethyl acetate in hexanes) to afford the product as a light yellow oil (196 mg, 51%). $[\alpha]_D^{24}$ + 25 (*c* 0.29, $CHCl_3$); R_f = 0.24 (30% EtOAc/hexanes); IR (film) 2956, 2931, 2860, 1742, 1691, 1650, 1456, 1390, 1321, 1156, 1078 cm^{-1}; ^1H NMR (600 MHz, $CDCl_3$) δ 7.39-7.27 (series of m, 5H), 5.50 (m, 1H), 5.39 (m, 1H), 5.20-5.06 (series of m, 3H), 4.65 (m, 1H), 3.20-2.66 (series of m, 12H), 1.99 (m, 1H), 1.81-1.60 (series of m, 5H), 1.49 (s, 9H), 1.37-1.21 (series of m, 16H), 0.90-0.86 (series of m, 6H); ^{13}C NMR (150 MHz, $CDCl_3$) ppm 172.2, 171.8, 171.4, 170.9, 155.3, 135.5, 128.6, 128.2, 128.1, 80.3, 79.9, 66.8, 58.8, 56.5, 53.0, 52.1, 50.8, 49.6, 31.5, 31.2, 31.1, 30.4, 29.8, 29.3, 29.1, 28.9, 28.0, 25.6, 22.4, 22.3, 14.6, 13.9; HRMS (EI): Exact mass calcd for $C_{36}H_{61}N_4O_7$ $[M+H]^+$ 661.4540, found 661.4537. MPT-4-300

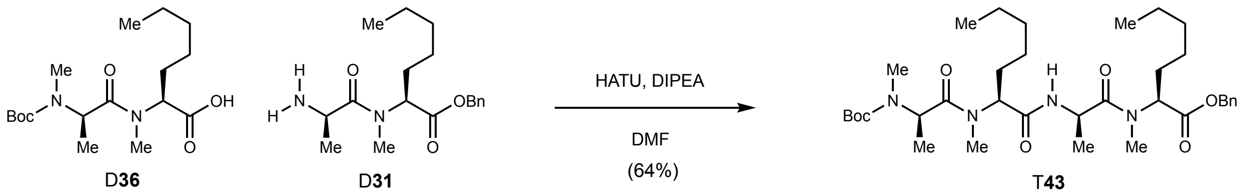

Benzyl (6*R*,9*S*,12*R*,15*S*)-2,2,5,6,8,12,14-heptamethyl-4,7,10,13-tetraoxo-9,15-dipentyl-3-oxa-5,8,11,14-tetraazahexadecan-16-oate (T43). A round-bottom flask was charged with the amine (172 mg, 537 µmol), acid (185 mg, 537 µmol), and DMF (4.0 mL). The mixture was cooled to 0 °C and then DIPEA (281 µL, 1.61 mmol) and HATU (613 mg, 1.61 mmol) were added. The reaction was stirred at 0 °C for 30 min, then warmed to 23 °C for 18 h.. The reaction mixture was poured into cold 10% aq citric acid and extracted with EtOAc. The combined organic layers were washed with satd aq $NaHCO_3$, brine, dried, and concentrated. The crude residue was subjected to flash column chromatography (SiO_2, 5-30% ethyl acetate in hexanes) to afford the product as a light yellow oil (221 mg, 64%). $[\alpha]_D^{24}$ + 2.9 (*c* 0.68, $CHCl_3$); R_f = 0.23 (25% EtOAc/hexanes); IR (film) 3330, 2957, 2929, 2859, 1741, 1682, 1455, 1367, 1254, 1152, 1081 cm^{-1}; ^1H NMR (600 MHz, $CDCl_3$) δ 7.39-7.30 (series of m, 5H), 7.00 (br s, 1H), 5.44-3.74 (series of m, 6H) 3.23-2.69 (series of m, 9H), 1.98 (m, 1H), 1.74 (m, 1H), 1.56 (m, 1H), 1.46 (m, 9H), 1.36-1.22 (series of m, 19H), 0.89-0.86 (series of m, 3H); ^{13}C NMR (150 MHz, $CDCl_3$) This compound exists in multiple conformations, causing significant peak overlap. Refer to image of the 13C NMR spectrum; HRMS (EI): Exact mass calcd for $C_{35}H_{62}N_5O_7$ $[M+NH_4]^+$ 664.4644, found 664.4642. MPT-3-056

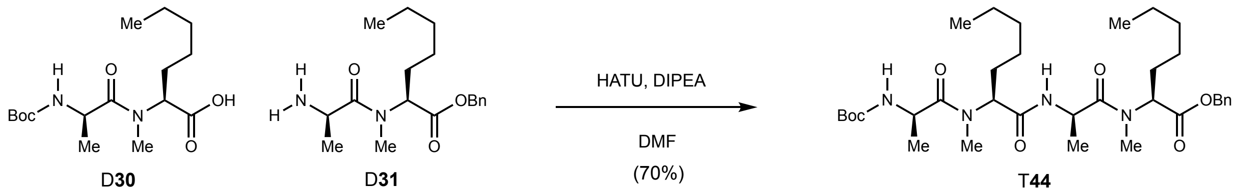

Benzyl (6*R*,9*S*,12*R*,15*S*)-2,2,6,8,12,14-hexamethyl-4,7,10,13-tetraoxo-9,15-dipentyl-3-oxa-5,8,11,14-tetraazahexadecan-16-oate (T44). A round-bottom flask was charged with the amine (400 mg, 1.21 mmol), acid (400 mg, 1.21 mmol), and DMF (3.0 mL). The mixture was cooled to 0 °C and then DIPEA (633 µL, 3.63 mmol) and HATU (1.38 g, 3.63 mmol) were added. The reaction was stirred at 0 °C for 30 min, then warmed to 23 °C for 18 h. The reaction mixture was poured into cold 10% aq citric acid and extracted with EtOAc. The combined organic layers were washed with satd aq $NaHCO_3$, brine, dried, and concentrated. The crude residue was subjected to flash column chromatography (SiO_2, 5-30% ethyl acetate in hexanes) to afford the product as a light yellow oil (536 mg, 70%). $[\alpha]_D^{24}$ - 31 (*c* 0.57, $CHCl_3$); R_f = 0.13 (25% EtOAc/hexanes); IR (film) 3300, 2956, 2931, 2860, 1741, 1713, 1643, 1518, 1455, 1367, 1247, 1170, 1091 cm^{-1}; ^1H NMR (600 MHz, $CDCl_3$) δ 7.39-7.30 (series of m, 5H), 7.01 (series of m, 2H), 5.33-4.14 (series of m, 6H), 3.33-2.82 (series of m, 6H), 1.98 (m, 2H), 1.71 (m, 2H), 1.55 (m, 1H), 1.44 (s, 9H), 1.35-1.23 (series of m, 17H), 0.91-0.88 (series of m, 6H); ^{13}C NMR (150 MHz, $CDCl_3$) ppm 173.2, 171.8, 171.6, 170.9, 151.7, 135.5, 128.7, 128.2, 127.8, 79.9, 79.5, 66.9, 59.6, 56.6, 52.3, 50.2, 47.0, 45.6, 45.6, 36.7, 31.4, 31.3, 31.1, 30.4, 29.7, 29.4, 28.3, 25.7, 25.4, 22.4, 14.0, 13.9; HRMS (EI): Exact mass calcd for $C_{34}H_{57}N_4O_7$ $[M+H]^+$ 633.4227, found 633.4222. MPT-4-057

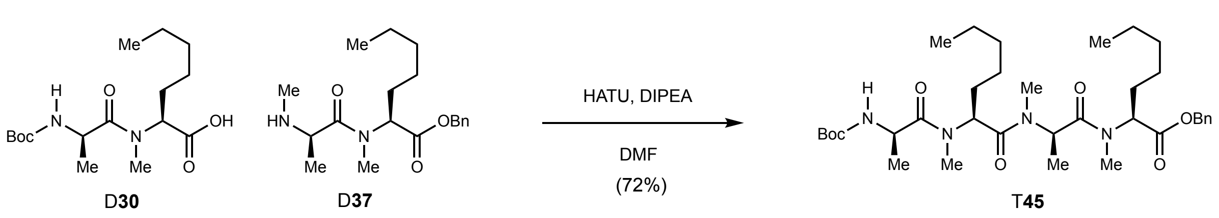

Benzyl (6*R*,9*S*,12*R*,15*S*)-2,2,6,8,11,12,14-heptamethyl-4,7,10,13-tetraoxo-9,15-dipentyl-3-oxa-5,8,11,14-tetraazahexadecan-16-oate (T45). A round-bottom flask was charged with the amine (300 mg, 909 µmol), acid (300 mg, 909 µmol), and DMF (1.0 mL). The mixture was cooled to 0 °C and then DIPEA (475 µL, 2.72 mmol) and HATU (1.03 g, 2.72 mmol) were added. The reaction was stirred at 0 °C for 30 min, then warmed to 23 °C for 18 h.. The reaction mixture was poured into cold 10% aq citric acid and extracted with EtOAc. The combined organic layers were washed with satd aq $NaHCO_3$, brine, dried, and concentrated. The crude residue was subjected to flash column chromatography (SiO_2, 5-30% ethyl acetate in hexanes) to afford the product as a light yellow foam (423 mg, 72%). $[\alpha]_D^{24}$ + 7.8 (*c* 0.43, $CHCl_3$); R_f = 0.23 (25% EtOAc/hexanes); IR (film) 2956, 2930, 2860, 1739, 1712, 1643, 1456, 1405, 1247, 1079 cm^{-1}; ^1H NMR (600 MHz, $CDCl_3$) δ 7.36-7.30 (series of m, 5H), 5.50 (m, 2H), 5.22-5.06 (series of m, 3H), 4.61 (m, 1H), 3.00-2.75 (series of m, 9H), 1.98 (br m, 1H), 1.79-1.64 (br series of m, 4H), 1.42 (br s, 9H), 1.32-1.21 (series of m, 15H), 0.88 (br m, 6H); ^{13}C NMR (150 MHz, ($CDCl_3$) ppm 173.1, 172.0, 171.1, 170.3, 155.1, 135.6, 128.0, 128.4, 128.2, 79.5, 67.0, 57.0, 56.7, 56.5, 52.9, 52.1, 49.9, 49.6, 31.5, 31.2, 30.2, 30.0, 29.3, 28.9, 28.0, 25.8, 22.5, 22.4, 18.8, 14.5, 13.9; HRMS (EI): Exact mass calcd for $C_{35}H_{59}N_4O_7$ $[M+H]^+$ 647.4384, found 646.4388. MPT-4-159

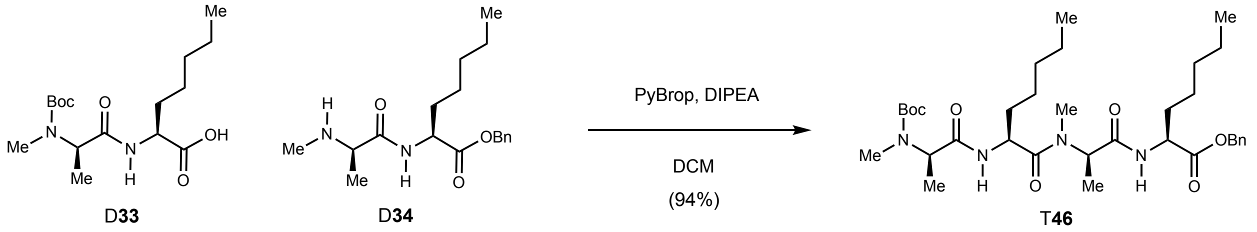

Benzyl (6*R*,9*S*,12*R*,15*S*)-2,2,5,6,11,12-hexamethyl-4,7,10,13-tetraoxo-9,15-dipentyl-3-oxa-5,8,11,14-tetraazahexadecan-16-oate (T46). A round-bottom flask was charged with the amine (254 mg, 793 µmol), acid (262 mg, 793 µmol), and DCM (7.0 mL). The mixture was cooled to 0 °C and then DIPEA (414 µL, 2.38 mmol) and PyBrop (581 mg, 1.19 mmol) were added. The reaction was stirred at 0 °C for 30 min, then warmed to 23 °C for 2 h. The reaction mixture was poured into cold 10% aq citric acid and extracted with DCM. The combined organic layers were washed with satd aq $NaHCO_3$, brine, dried, and concentrated. The crude residue was subjected to flash column chromatography (SiO_2, 5-30% ethyl acetate in hexanes) to afford the product as a light yellow oil (611 mg, 94%). $[\alpha]_D^{24}$ + 36 (*c* 0.86, $CHCl_3$); R_f = 0.26 (30% EtOAc/hexanes); IR (film) 3318, 2956, 2931, 2860, 1742, 1661, 1530, 1456, 1367, 1256, 1154, 1090 cm^{-1}; ^1H NMR (600 MHz, $CDCl_3$ δ 7.41-7.28 (series of m, 7H), 7.00 (br s, 1H), 6.70 (br s, 1H), 5.26 (br m, 1H), 5.13 (d, *J* = 12.1 Hz, 1H), 5.00 (d, *J* = 12.1 Hz, 1H), 4.71 (br m, 1H), 4.46 (m, 2H), 2.86-2.56 (series of m, 6H), 1.79 (br m, 1H), 1.64 (br m, 1H), 1.54 (br m, 1H), 1.43 (s, 9H), 1.37 (m, 3H), 1.27-1.17 (series of m, 16H), 0.83 (br t, *J* = 6.1 Hz, 3H), 0.78 (br t, *J* = 6.3 Hz, 3H); ^{13}C NMR (150 MHz, $CDCl_3$) ppm 172.5, 172.4, 172.0, 170.6, 161.0, 134.9, 128.7, 128.6, 128.5, 79.9, 68.9, 52.5, 52.0, 50.0, 42.2, 32.0, 31.9, 31.6, 31.5, 31.2, 30.8, 30.7, 29.9, 29.7, 29.4, 25.4, 25.0, 22.4, 14.0, 13.4; HRMS (EI): Exact mass calcd for $C_{34}H_{60}N_5O_7$ $[M+NH_4]^+$ 650.4493, found 650.4487. MPT-2-193

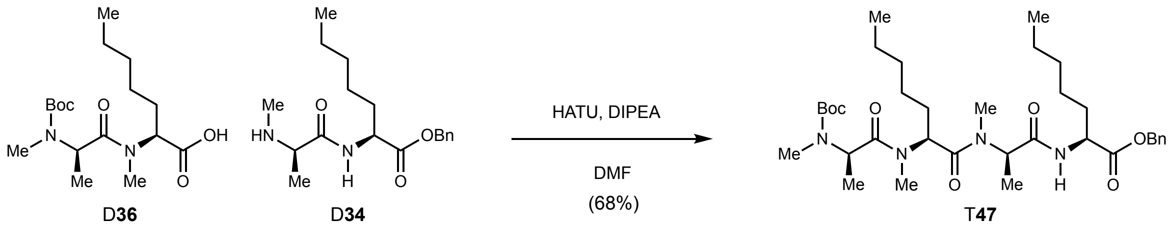

Benzyl (6*R*,9*S*,12*R*,15*S*)-2,2,5,6,8,11,12-heptamethyl-4,7,10,13-tetraoxo-9,15-dipentyl-3-oxa-5,8,11,14-tetraazahexadecan-16-oate (T47). A round-bottom flask was charged with the amine (171 mg, 535 µmol), acid (184 mg, 535 µmol), and DMF (5.4 mL). The mixture was cooled to 0 °C and then DIPEA (283.3 µL, 1.61 mmol) and HATU (610 mg, 1.61 mol) were added. The reaction was stirred at 0 °C for 30 min, then warmed to 23 °C for 18 h. The reaction mixture was poured into cold 10% aq citric acid and extracted with EtOAc. The combined organic layers were washed with satd aq $NaHCO_3$, brine, dried, and concentrated. The crude residue was subjected to flash column chromatography (SiO_2, 5-35% ethyl acetate in hexanes) to afford the product as a yellow foam (235 mg, 68%). $[\alpha]_D^{24}$ + 24 (*c* 0.34, $CHCl_3$); R_f = 0.31 (25% EtOAc/hexanes); IR (film) 3448, 2956, 2929, 2810, 1745, 1701, 1686, 1473, 1389, 1259, 1182, 1087 cm^{-1}; ^{1}H NMR (600 MHz, $(CD_3)_2CO$) δ 7.45-7.27 (series of m, 5H), 7.04 (br m, 1H), 5.33-4.53 (series of m, 6H), 3.07-2.69 (series of m, 9H), 2.01-1.78 (series of m, 3H), 1.62 (m, 1H), 1.45 (m, 9H), 1.37-1.16 (series of m, 18H), 0.93-0.83 (series of m, 6H); ^{13}C NMR (150 MHz, $CDCl_3$) ppm 173.2, 171.9, 171.2, 170.5, 155.3, 135.6, 128.7, 128.4, 128.1, 79.5, 67.0, 66.8, 56.9, 56.3, 53.1, 52.2, 49.6, 46.9, 31.5, 31.2, 30.2, 29.9, 28.9, 28.3, 27.9, 25.7, 25.4, 22.4, 18.9, 14.4, 13.9; HRMS (EI): Exact mass calcd for $C_{35}H_{58}N_4NaO_7$ $[M+Na]^+$ 647.4384, found 647.4388. MPT-4-156

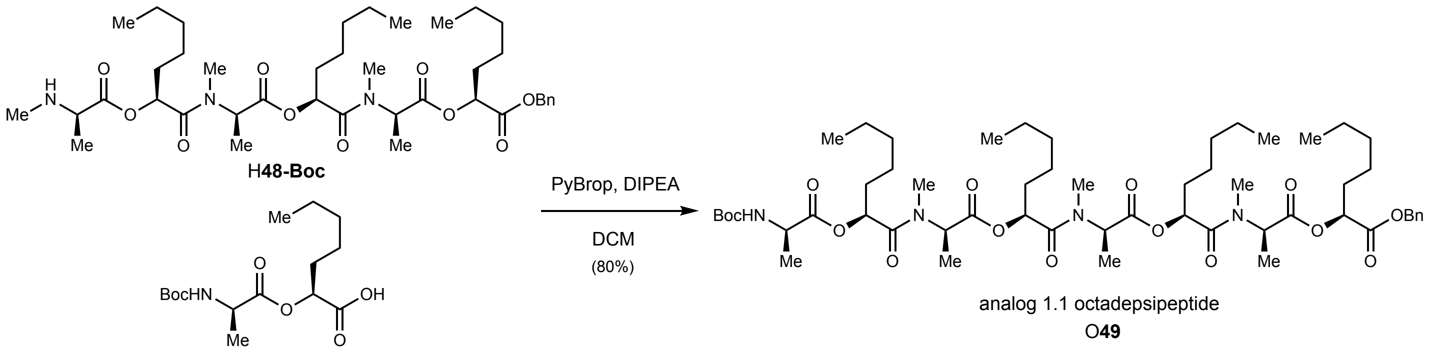

Benzyl (*S*)-2-(((6*R*,9*S*,12*R*,15*S*,18*R*,21*S*,24*R*)-2,2,6,11,12,17,18,23,24-nonamethyl-4,7,10,13,16,19,22-heptaoxo-9,15,21-tripentyl-3,8,14,20-tetraoxa-5,11,17,23-tetraazapentacosan-25-oyl)oxy)heptanoate (O49). A round-bottom flask was charged with the amine (141 mg, 189 µmol), acid (66.0 mg, 189 µmol), and DCM (1.8 mL). The mixture was cooled to 0 °C and then DIPEA (98.8 µL, 567 µmol) and PyBrop (92.3 mg, 189 µmol) were added. The reaction was stirred at 0 °C for 30 min, then allowed to warm to ambient temperature and stir for an additional 1.5 h. The reaction mixture was poured into cold 10% aq citric acid and extracted with DCM. The combined organic layers were washed with satd aq $NaHCO_3$, brine, dried, and concentrated. The crude residue was subjected to flash column chromatography (SiO_2, 10-60% ethyl acetate in hexanes) to afford the product as a light yellow oil (158 mg, 80%). $[\alpha]_D^{24}$ +34 (*c* 1.36, $CHCl_3$); R_f = 0.40 (60% EtOAc/hexanes); IR (film) 3302, 2930, 1743, 1663, 1456, 1186 cm^{-1}; ^{1}H NMR (600 MHz, $CDCl_3$) This compound exists in multiple conformations, causing significant peak overlap. Refer to image of the ^{1}H NMR spectrum; ^{13}C NMR (150 MHz, $CDCl_3$) This

compound exists in multiple conformations, causing significant peak overlap. Refer to image of the ^{13}C NMR spectrum; HRMS (EI): Exact mass calcd for $C_{55}H_{94}N_5O_{15}$ [M+NH$_4$]$^+$ 1064.6746, found [1064.6742]. MPT-2-166

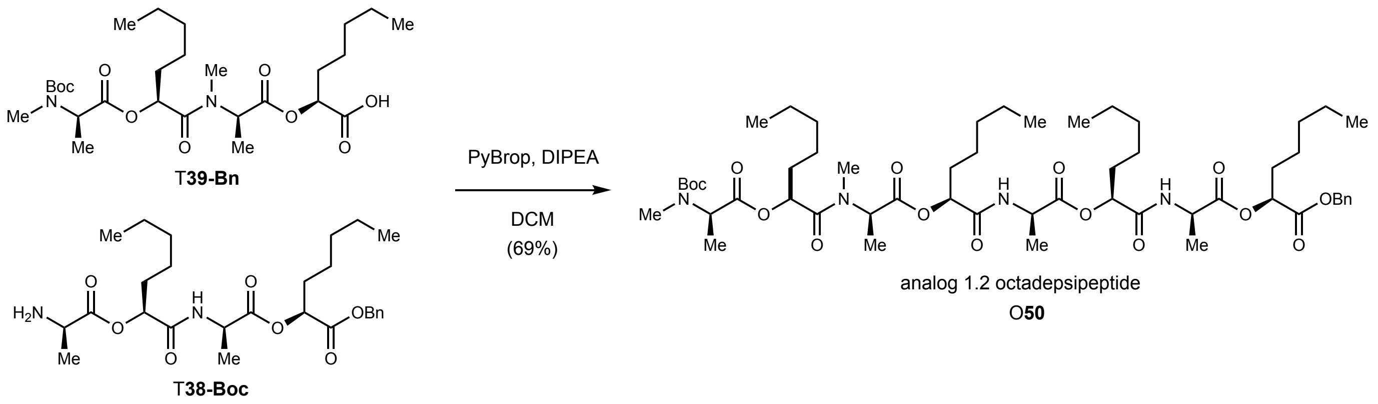

Benzyl (*S*)-2-(((6*R*,9*S*,12*R*,15*S*,18*R*,21*S*,24*R*)-2,2,5,6,11,12,18,24-octamethyl-4,7,10,13,16,19,22-heptaoxo-9,15,21-tripentyl-3,8,14,20-tetraoxa-5,11,17,23-tetraazapentacosan-25-oyl)oxy)heptanoate (O50). A round-bottom flask was charged with the amine (93.0 mg, 184 µmol), acid (100 mg, 184 µmol), and DCM (1.8 mL). The mixture was cooled to 0 °C and then DIPEA (95.9 µL, 551 µmol) and PyBrop (108 mg, 220 µmol) were added. The reaction was stirred at 0 °C for 30 min, then allowed to warm to ambient temperature and stir for an additional 1.5 h. The reaction mixture was poured into cold 10% aq citric acid and extracted with DCM. The combined organic layers were washed with satd aq $NaHCO_3$, brine, dried, and concentrated. The crude residue was subjected to flash column chromatography (SiO_2, 10-50% ethyl acetate in hexanes) to afford the product as a light-yellow oil (130 mg, 69%). $[\alpha]_D^{24}$ –6.5 (*c* 0.53, $CHCl_3$); R$_f$ = 0.21 (30% EtOAc/hexanes); IR (film) 3303, 2956, 2929, 2871, 2860, 1747, 1671, 1538, 1455, 1389, 1210, 1086 cm^{-1}; ^{1}H NMR (600 MHz, $CDCl_3$) δ 7.65 (br s, 1H), 7.40 (br d, *J* = 7.3 Hz, 1H), 5.21-5.07 (series of m, 7H), 4.91 and 4.07 (br q, *J* = 6.9 Hz, 1H), 4.67-4.63 (m, 1H), 4.25-4.20 (m, 1H), 3.19 (br m, 3H), 2.81 (br m, 3H), 1.85-1.68 (series of m, 8H), 1.48-1.43 (series of m, 18H), 1.38 (br s, 9H), 1.32-1.26 (series of m, 18H), 0.89-0.85 (series of m, 12H); ^{13}C NMR (150 MHz, $CDCl_3$) This compound is a mixture of rotamers causing significant peak broadening and overlap. Refer to image of the ^{13}C NMR spectrum; HRMS (EI): Exact mass calcd for $C_{54}H_{88}N_4NaO_{15}$ [M+Na]$^+$ 1055.6144, found 1055.6144. MPT-2-154

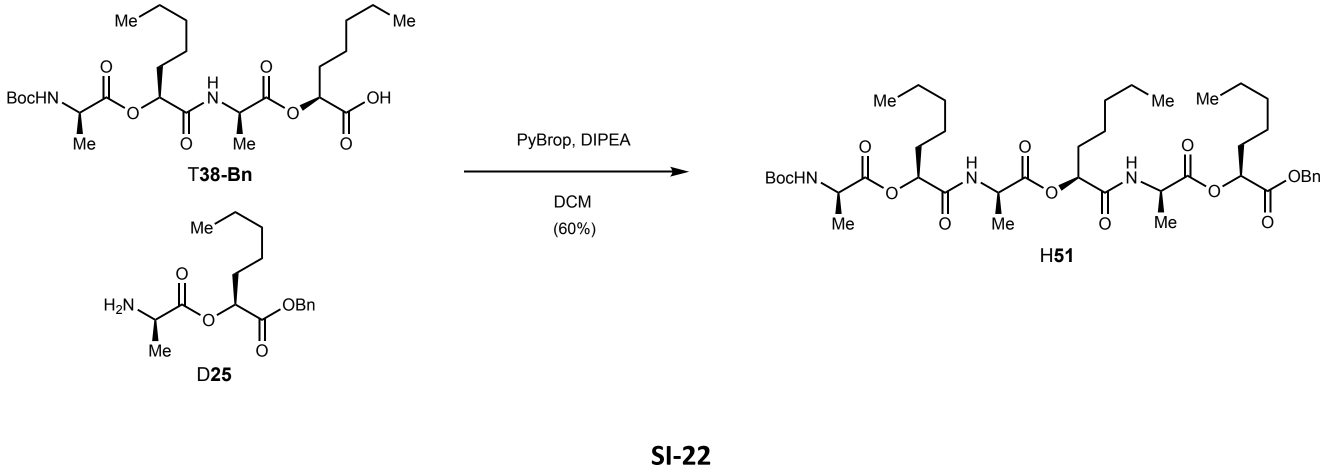

Benzyl (*S*)-2-(((6*R*,9*S*,12*R*,15*S*,18*R*)-2,2,6,12,18-pentamethyl-4,7,10,13,16-pentaoxo-9,15-dipentyl-3,8,14-trioxa-5,11,17-triazanonadecan-19-oyl)oxy)heptanoate (H51). A round-bottom flask was charged with the amine (75.0 mg, 244 µmol), acid (126 mg, 244 µmol), and DCM (2.4 mL). The mixture was cooled to 0 °C and then DIPEA (128 µL, 732 µmol) and PyBrop (119 mg, 244 µmol) were added. The reaction was stirred at 0 °C for 30 min, then allowed to warm to ambient temperature and stir for an additional 1.5 h. The reaction mixture was poured into cold 10% aq citric acid and extracted with DCM. The combined organic layers were washed with satd aq $NaHCO_3$, brine, dried, and concentrated. The crude residue was subjected to flash column chromatography (SiO_2, 10-60% ethyl acetate in hexanes) to afford the product as a light yellow oil (117 mg, 60%). $[\alpha]_D^{24}$ – 29 (*c* 0.32, $CHCl_3$); R_f = 0.49 (30% EtOAc/hexanes); IR (film) 3346, 2956, 2927, 2858, 1751, 1662, 1534, 1457, 1157, 1069 cm^{-1}; ^{1}H NMR (600 MHz, $CDCl_3$) δ 7.37-7.31 (series of m, 5H), 5.23-5.14 (series of m, 3H), 5.09 (br t, *J* = 6.3 Hz, 1H), 5.00 (br d, *J* = 5.4 Hz, 1H), 4.66 (dd, *J* = 7.4, 7.4 Hz, 1H), 4.31 (dd, *J* = 6.8, 5.8 Hz, 1H), 4.14 (dd, *J* = 6.8, 6.8 Hz, 1H), 1.99-1.95 (series of m, 1H), 1.88-1.78 (series of m, 5H), 1.50 (br d, *J* = 7.6 Hz, 3H), 1.45 (br d, *J* = 7.4 Hz, 3H), 1.42 (s, 8H), 1.41 (br d, *J* = 7.6 Hz, 4H), 1.36-1.25 (series of m, 18H), 0.87 (t, *J* = 6.7 Hz, 3H), 0.86 (t, *J* = 7.0 Hz, 3H), 0.85 (t, *J* = 6.6 Hz, 3H) [three N-*H* peaks not observed]; ^{13}C NMR (150 MHz, $CDCl_3$) ppm 173.5, 172.8, 172.6, 171.0, 170.3, 170.0, 156.4, 135.9, 129.1, 128.9, 128.8, 81.1, 74.9, 74.4, 73.4, 67.5, 50.5, 49.8, 48.6, 48.3, 32.2, 31.9, 31.8, 31.6, 30.5, 30.3, 28.8, 25.3, 25.2, 24.8, 23.0, 18.0, 17.5, 16.9, 14.7, 14.6, 14.52, 14.47 HRMS (EI): Exact mass calcd for $C_{42}H_{67}N_3O_{12}Na$ [M+Na]$^+$ 828.4623, found 828.4622. MPT-2-157

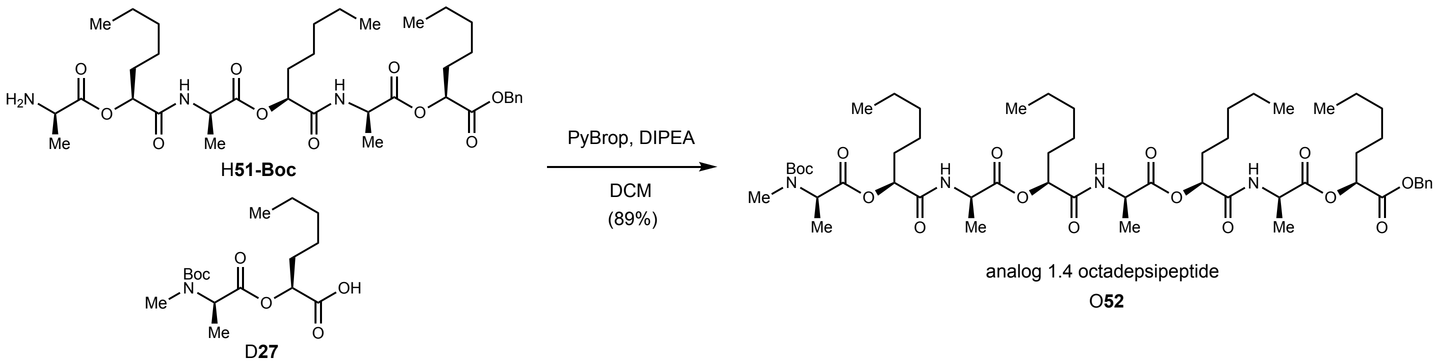

Benzyl (*S*)-2-(((6*R*,9*S*,12*R*,15*S*,18*R*,21*S*,24*R*)-2,2,5,6,12,18,24-heptamethyl-4,7,10,13,16,19,22-heptaoxo-9,15,21-tripentyl-3,8,14,20-tetraoxa-5,11,17,23-tetraazapentacosan-25-oyl)oxy)heptanoate (O52). A round-bottom flask was charged with the amine (102 mg, 184 µmol), acid (47 mg, 184 µmol), and DCM (1.8 mL). The mixture was cooled to 0 °C and then DIPEA (95.0 µL, 540 µmol) and PyBrop (88.0 mg, 184 µmol) were added. The reaction was stirred at 0 °C for 30 min, then allowed to warm to ambient temperature and stir for an additional 1.5 h. The reaction mixture was poured into cold 10% aq citric acid and extracted with DCM. The combined organic layers were washed with satd aq $NaHCO_3$, brine, dried, and concentrated. The crude residue was subjected to flash column chromatography (SiO_2, 10-60% ethyl acetate in hexanes) to afford the product as a light yellow oil (132 mg, 89%). $[\alpha]_D^{24}$ –21 (*c* 0.93, $CHCl_3$); R_f = 0.28 (30% EtOAc/hexanes); IR (film) 3300, 2956, 2930, 2860, 1749, 1665, 1455, 1382, 1156, 1086 cm^{-1}; ^{1}H NMR (600 MHz, $CDCl_3$) δ 7.95 (br d, *J* = 4.0 Hz, 1H), 7.65 (br d, *J* = 4.0 Hz, 1H), 7.37-7.31 (series of m, 6H), 5.21-3.70 (series of m, 10H), 2.98 (br s, 3H), 1.88-1.77 (series of m, 8H), 1.52 (m, 1H), 1.51-1.43 (series of m, 19H), 1.37-1.32 (series of m, 7H), 1.30-1.24 (series of m, 18H), 0.89-0.85 (series of m, 12H); ^{13}C NMR (150 MHz, $CDCl_3$) This compound is a mixture of rotamers causing significant peak broadening and overlap. Refer to the image of the ^{13}C NMR spectrum; HRMS (EI): Exact mass calcd for $C_{53}H_{86}N_4NaO_{15}$ [M+Na]$^+$ 1041.5988, found 1041.5987. MPT-2-160

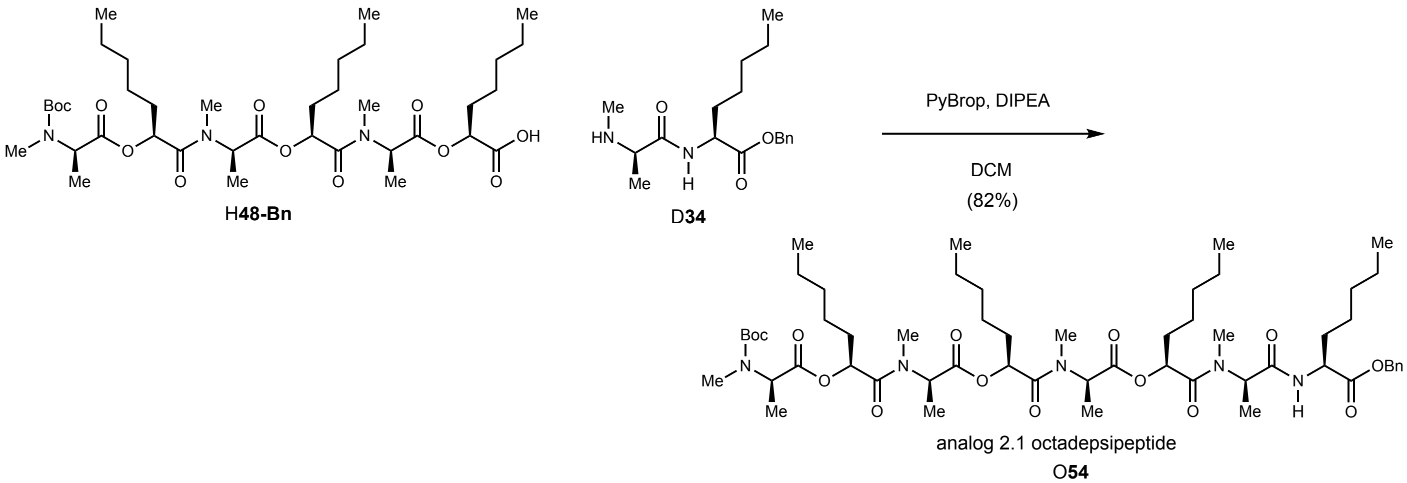

(4*S*,7*R*,10*S*,13*R*,16*S*)-7,8,13,14-tetramethyl-3,6,9,12,15-pentaoxo-4,10-dipentyl-1-phenyl-2,5,11-trioxa-8,14-diazahenicosan-16-yl (6*R*,9*S*,12*R*)-2,2,5,6,11,12-hexamethyl-4,7,10-trioxo-9-pentyl-3-oxa-5,8,11-triazatridecan-13-oate (O54). A round-bottom flask was charged with the amine (88.0 mg, 120 µmol), acid (39.0 mg, 120 µmol), and DCM (1.2 mL). The mixture was cooled to 0 °C and then DIPEA (61.0 µL, 350 µmol) and PyBrop (86.0 mg, 180 µmol) were added. The reaction was stirred at 0 °C for 30 min, then warmed to 23 °C for 2 h. The reaction mixture was poured into cold 10% aq citric acid and extracted with DCM. The combined organic layers were washed with satd aq $NaHCO_3$, brine, dried, and concentrated. The crude residue was subjected to flash column chromatography (SiO_2, 5-40% ethyl acetate in hexanes) to afford the product as a light yellow oil (104 mg, 82%). $[\alpha]_D^{24}$ + 9.9 (*c* 0.75, $CHCl_3$); R_f = 0.72 (30% EtOAc/hexanes); IR (film) 2925, 2856, 1746, 1668, 1456, 1377, 1215, 1103 cm^{-1}; ^{1}H NMR (600 MHz, $CDCl_3$) δ 7.31-7.24 (series of m, 5H), 6.89 (br s, 1H), 5.19-4.25 (series of m, 10H), 3.09-2.60 (series of m, 12H), 2.23 (m, 1H), 2.00 (m, 1H), 1.84-1.75 (br m, 3H), 1.61-1.56 (series of m, 3H), 1.43-1.40 (series of m, 9H), 1.34-1.30 (series of m, 3H), 1.27-1.24 (series of m, 22H), 1.23-1.20 (series of m, 11H), 0.90-0.88 (series of m, 12H); ^{13}C NMR (150 MHz, $CDCl_3$) This compound exists in multiple conformations, causing significant peak overlap. Refer to image of the ^{13}C NMR spectrum; HRMS (EI): Exact mass calcd for $C_{56}H_{97}N_6O_{14}$ $[M+NH_4]^+$ 1077.7063, found 1077.7058. MPT-2-189

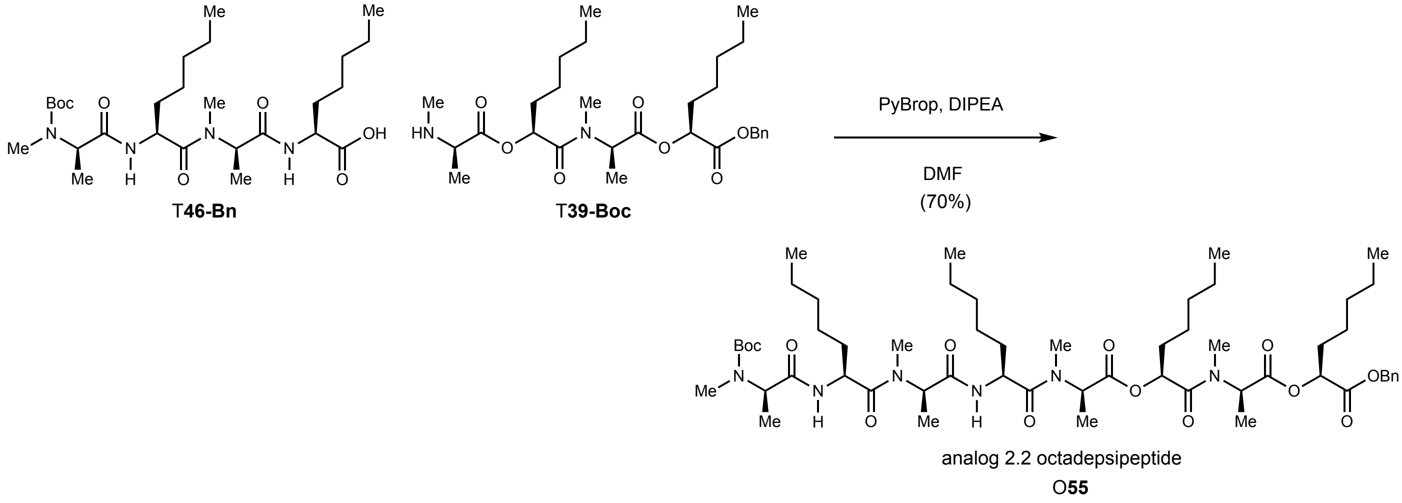

(*S*)-1-(((*R*)-1-(((*S*)-1-(benzyloxy)-1-oxoheptan-2-yl)oxy)-1-oxopropan-2-yl)(methyl)amino)-1-oxoheptan-2-yl (6*R*,9*S*,12*R*,15*S*,18*R*)-2,2,5,6,11,12,17,18-octamethyl-4,7,10,13,16-pentaoxo-9,15-dipentyl-3-oxa-5,8,11,14,17-pentaazanonadecan-19-oate (O55). A round-bottom flask was charged with the amine (110 mg, 206 µmol), acid (110 mg, 206 µmol), and DMF (2.0 mL). The mixture was cooled to 0 °C and then DIPEA (108 µL, 618 mmol) and PyBrop (638 mg, 1.31 mmol) were added. The reaction was stirred at 0 °C for 30 min, then warmed to 23 °C for 2 h. The reaction mixture was poured into cold 10% aq citric acid and extracted with EtOAc. The combined organic layers were washed with satd aq $NaHCO_3$, brine, dried, and concentrated. The crude residue was subjected to flash column chromatography (SiO_2, 5-50% ethyl acetate in hexanes) to afford the product as a light yellow oil (150 mg, 70%). $[\alpha]_D^{24}$ + 63 (*c* 0.64, $CHCl_3$); R_f = 0.25 (30% EtOAc/hexanes); IR (film) 3387, 2956, 2932, 2861, 1742, 1651, 1522, 1457, 1391, 1214, 1154, 1093 cm^{-1}; ^{1}H NMR (600 MHz, $CDCl_3$) δ 7.30-7.24 (series of m, 5H), 7.05 (br s, 1H), 5.18-4.98 (series of m, 6H), 4.73-4.36 (series of m, 4H), 2.99-2.71 (series of m, 12H), 1.82-1.73 (series of m, 3H), 1.66-1.56 (series of m, 6H), 1.47-1.41 (series of m, 9H), 1.40-1.34 (series of m, 14H), 1.28-1.18 (series of m, 20H), 0.90-0.88 (series of m, 12H); ^{13}C NMR (150 MHz, $CDCl_3$) This compound exists in multiple conformations, causing significant peak overlap. Refer to image of the ^{13}C NMR spectrum; HRMS (EI): Exact mass calcd for $C_{56}H_{98}N_7O_{13}$ $[M+NH_4]^+$ 1076.7223 found 1076.7217. MPT-2-279

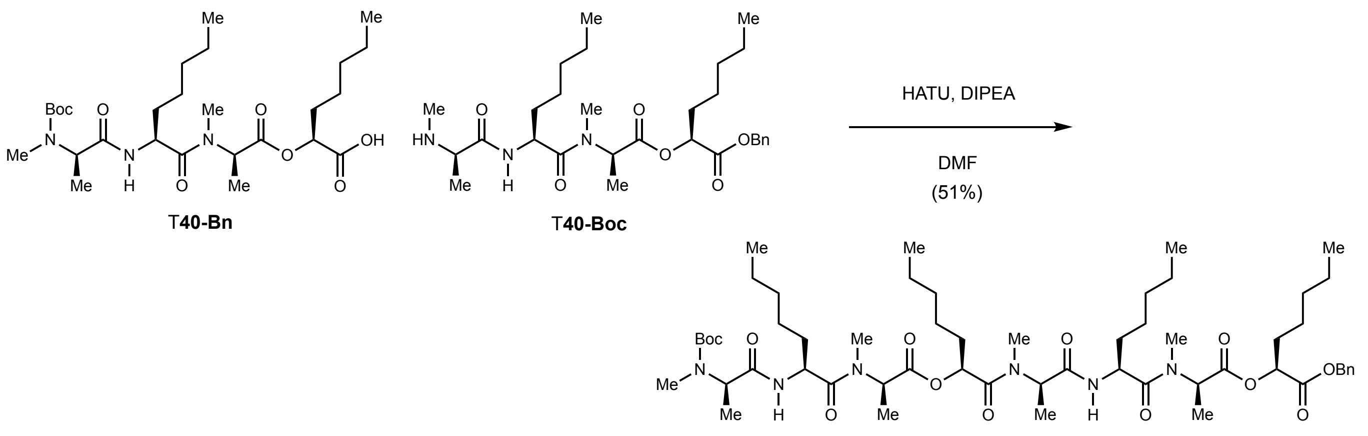

analog 2.3 octadepsipeptide
O**56**

(4*S*,7*R*,10*S*,13*R*,16*S*)-7,8,13,14-tetramethyl-3,6,9,12,15-pentaoxo-4,10-dipentyl-1-phenyl-2,5-dioxa-8,11,14-triazahenicosan-16-yl (6*R*,9*S*,12*R*)-2,2,5,6,11,12-hexamethyl-4,7,10-trioxo-9-pentyl-3-oxa-5,8,11-triazatridecan-13-oate (O56). A round-bottom flask was charged with the amine (96.2 mg, 180 µmol), acid (98.0 mg, 180 µmol), and DCM (1.8 mL). The mixture was cooled to 0 °C and then DIPEA (95 µL, 541 µmol) and HATU (206 mg, 541 µmol) were added. The reaction was stirred at 0 °C for 30 min, then warmed to 23 °C for 18 h. The reaction mixture was poured into cold 10% aq citric acid and extracted with DCM. The combined organic layers were washed with satd aq $NaHCO_3$, brine, dried, and concentrated. The crude residue was subjected to flash column chromatography (SiO_2, 5-50% ethyl acetate in hexanes) to afford the product as a light yellow oil (97 mg, 51%). $[\alpha]_D^{24}$ + 51 (*c* 0.70, $CHCl_3$); R_f = 0.16 (30% EtOAc in hexanes); IR (film) 2929, 1747, 1652, 1514, 1473, 1212 cm^{-1}; ^{1}H NMR (600 MHz, $CDCl_3$) δ 7.38-7.32 (series of m, 5H), 6.83 (br m, 2H), 5.37 (m, 1H), 5.22-5.15 (m, 2H), 5.13 (m, 3H), 5.02 (m, 1H), 4.91 (m, 1H), 4.86-4.74 (m, 2H), 3.32 (series of m, 12H), 1.86-1.61 (br m, 12H), 1.49 (br s, 9H), 1.38-1.23 (series of m, 32H), 0.90-0.88 (series of m, 12H); ^{13}C NMR (150 MHz, $CDCl_3$) This

compound exists in multiple conformations, causing significant peak overlap. Refer to image of the ^{13}C NMR spectrum; HRMS (EI): Exact mass calcd for $C_{56}H_{98}N_7O_{13}$ $[M+NH_4]^+$ 1076.7223, found 1076.7217. MPT-2-247

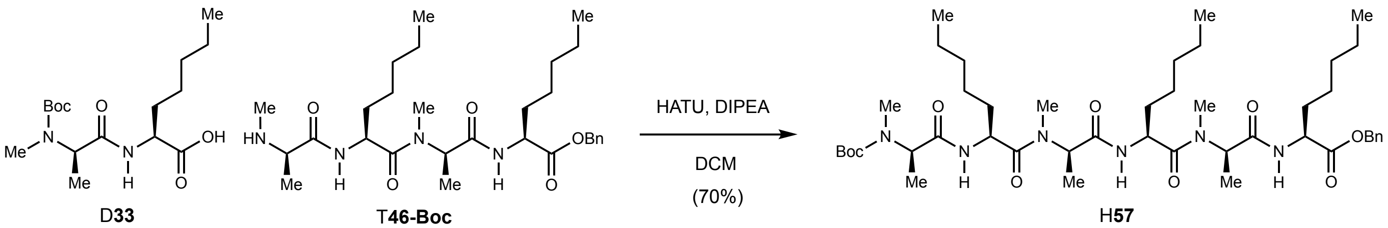

Benzyl (6*R*,9*S*,12*R*,15*S*,18*R*,21*S*)-2,2,5,6,11,12,17,18-octamethyl-4,7,10,13,16,19-hexaoxo-9,15,21-tripentyl-3-oxa-5,8,11,14,17,20-hexaazadocosan-22-oate (H57). A round-bottom flask was charged with the amine (112 mg, 338 μmol), acid (180 mg, 338 μmol), and DCM (2.1 mL). The mixture was cooled to 0 °C and then DIPEA (177 μL, 210 μmol) and HATU (385 mg, 1.01 mmol) were added. The reaction was stirred at 0 °C for 30 min, then warmed to 23 °C for 18 h. The reaction mixture was poured into cold 10% aq citric acid and extracted with DCM. The combined organic layers were washed with satd aq $NaHCO_3$, brine, dried, and concentrated. The crude residue was subjected to flash column chromatography (SiO_2, 5-60% ethyl acetate in hexanes) to afford the product as a light yellow oil (200 mg, 70%). $[\alpha]_D^{24}$ + 6.3 (*c* 0.48, $CHCl_3$); R_f = 0.23 (30% EtOAc/hexanes); IR (film) 3313, 2957, 2931, 2861, 1748, 1660, 1539, 1456, 1368, 1259, 1081 cm^{-1}; ^1H NMR (600 MHz, $CDCl_3$) δ 7.37-7.34 (series of m, 5H), 7.10 (br s, 1H), 6.91 (br s, 1H), 6.50 (br s, 1H), 5.20-5.14 (series of m, 3H), 5.10-5.17 (series of m, 2H), 4.66 (series of m, 2H), 4.35 (m, 1H), 3.28-2.69 (series of m, 9H), 1.96 (m, 1H), 1.80-1.73 (series of m, 2H), 1.66 (m, 1H), 1.42 (s, 9H), 1.40-1.38 (series of m, 4H), 1.37-1.35 (series of m, 4H), 1.29-1.26 (series of m, 2H), 1.24-1.21 (series of m, 9H), 1.21-1.18 (series of m, 10H), 0.89-0.85 (series of m, 9H); ^{13}C NMR (150 MHz, $CDCl_3$) ppm 172.2, 172.1, 172.0, 171.6, 169.7, 169.3, 157.1, 135.4, 128.6, 128.4, 128.2, 80.9, 75.1, 74.6, 73.1, 72.8, 72.1, 67.2, 49.1, 48.9, 48.1, 47.9, 47.8, 31.7, 31.5, 31.3, 31.1, 31.0, 30.9, 30.8, 29.7, 29.6, 28.4, 28.3, 24.7, 24.6, 22.4, 22.3, 17.6, 17.4, 17.1; HRMS (EI): Exact mass calcd for $C_{45}H_{80}N_7O_9$ $[M+NH_4]^+$ 862.6018, found 862.6019. MPT-2-287

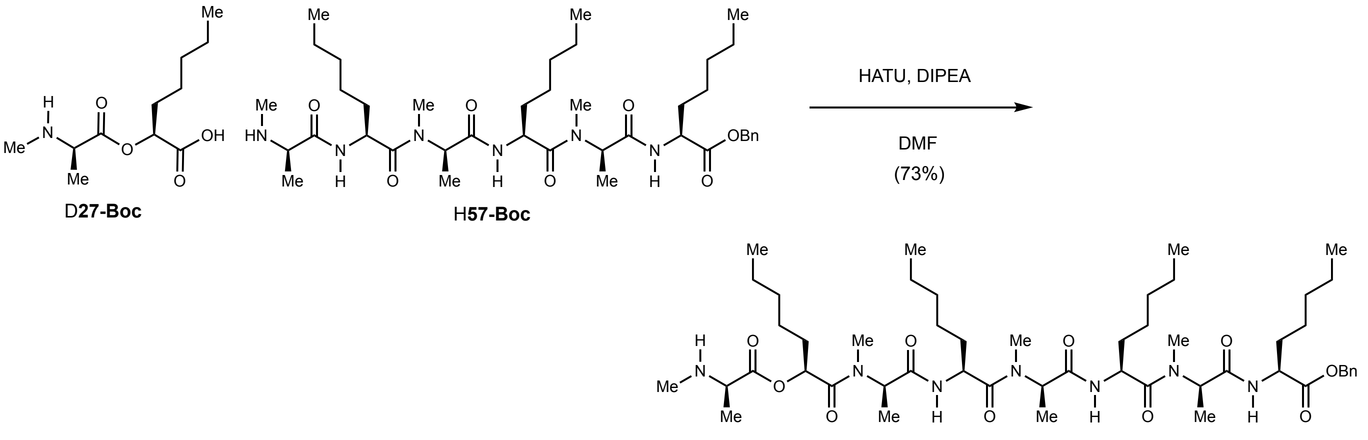

D**27-Boc** H**57-Boc**

analog 2.4 octadepsipeptide
O58 (Boc deprotected)

Benzyl (2*S*,5*R*,8*S*,11*R*,14*S*,17*R*,20*S*)-5,6,11,12,17,18-hexamethyl-20-((methyl-*D*-alanyl)oxy)-4,7,10,13,16,19-hexaoxo-2,8,14-tripentyl-3,6,9,12,15,18-hexaazapentacosanoate (O58, Boc-deprotected). A round-bottom flask was charged with the amine (162 mg, 217 µmol), acid (72.0 mg, 217 µmol), and DMF (2.2 mL). The mixture was cooled to 0 °C and then DIPEA (114 µL, 652 µmol) and HATU (248 mg, 652 µmol) were added. The reaction was stirred at 0 °C for 30 min, then warmed to 23 °C for 18 h. The reaction mixture was poured into cold 10% aq citric acid and extracted with EtOAc. The combined organic layers were washed with satd aq $NaHCO_3$, brine, dried, and concentrated. The crude residue was subjected to flash column chromatography (SiO_2, 5-60% ethyl acetate in hexanes) to afford the product[6] as a light yellow oil (168 mg, 73%). $[\alpha]_D^{24}$ + 22 (*c* 0.48, $CHCl_3$); R_f = 0.14 (50% EtOAc/hexanes); IR (film) 3333, 2956, 2930, 2860, 1747, 1667, 1532, 1456, 1381, 1187, 1156, 1082 cm^{-1}; ^{1}H NMR (600 MHz, $CDCl_3$) This compound exists as a mixture of rotamers causing significant peak broadening and overlap. Refer to the image of the ^{1}H NMR spectrum; ^{13}C NMR (150 MHz, $CDCl_3$) This compound is a mixture of rotamers causing significant peak broadening and overlap. Refer to the image of the ^{13}C NMR spectrum; HRMS (EI): Exact mass calcd for $C_{51}H_{91}N_8O_{10}$ $[M+NH_4]^+$ 975.6858, found 975.6856. MPT-2-273

[6] The product isolated, unexpectedly, was the Boc-deprotected O**58**. Therefore, it was characterized as such.

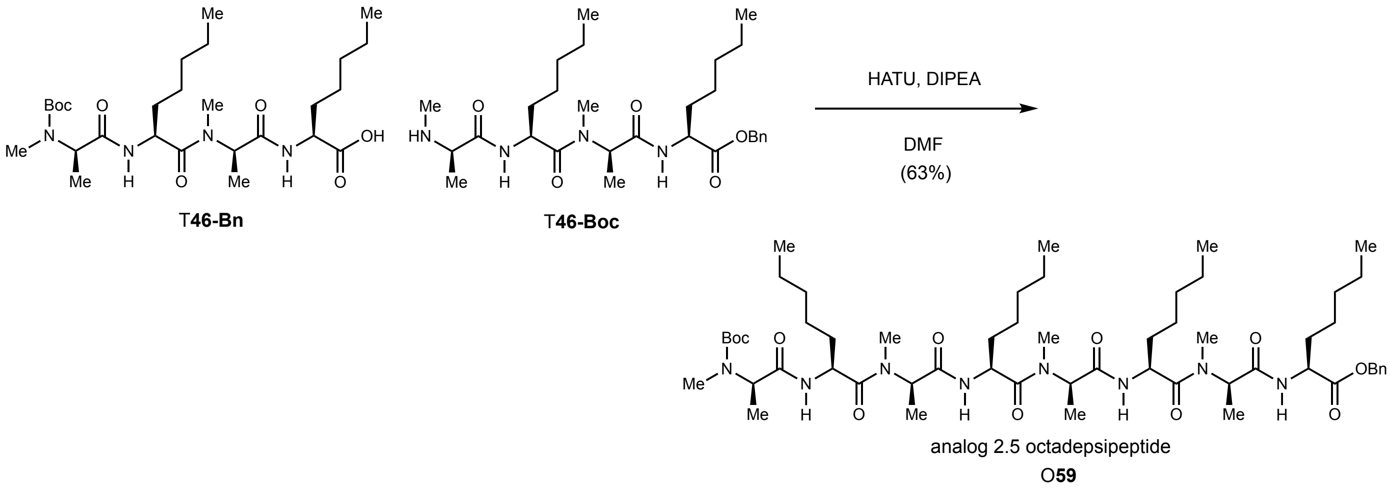

Benzyl (6*R*,9*S*,12*R*,15*S*,18*R*,21*S*,24*R*,27*S*)-2,2,5,6,11,12,17,18,23,24-decamethyl-4,7,10,13,16,19,22,25-octaoxo-9,15,21,27-tetrapentyl-3-oxa-5,8,11,14,17,20,23,26-octaazaoctacosan-28-oate (O59). A round-bottom flask was charged with the amine (93.3 mg, 175 µmol), acid (95.0 mg, 175 µmol), and DMF (1.8 mL). The mixture was cooled to 0 °C and then DIPEA (91.5 µL, 525 µmol) and HATU (200 mg, 525 µmol) were added. The reaction was stirred at 0 °C for 30 min, then warmed to 23 °C for 18 h. The reaction mixture was poured into cold 10% aq citric acid and extracted with EtOAc. The combined organic layers were washed with satd aq $NaHCO_3$, brine, dried, and concentrated. The crude residue was subjected to flash column chromatography (SiO_2, 5-40% ethyl acetate in hexanes) to afford the product as a light yellow oil (117 mg, 63%). $[\alpha]_D^{24}$ + 37 (*c* 0.45, $CHCl_3$); R_f = 0.47 (30% EtOAc/hexanes); IR (film) 3405, 2956, 2929, 2859, 1741, 1652, 1540, 1457, 1391, 1260, 1155, 1092 cm^{-1}; ^1H NMR (600 MHz, $CDCl_3$) This compound exists as a mixture of rotamers causing significant peak broadening and overlap. Refer to the image of the ^1H NMR spectrum; ^{13}C NMR (150 MHz, $CDCl_3$) This compound is a mixture of rotamers causing significant peak broadening and overlap. Refer to the image of the ^{13}C NMR spectrum; HRMS (EI): Exact mass calcd for $C_{56}H_{100}N_9O_{11}$ $[M+NH_4]^+$ 1074.7542, found 1074.7544. MPT-2-276

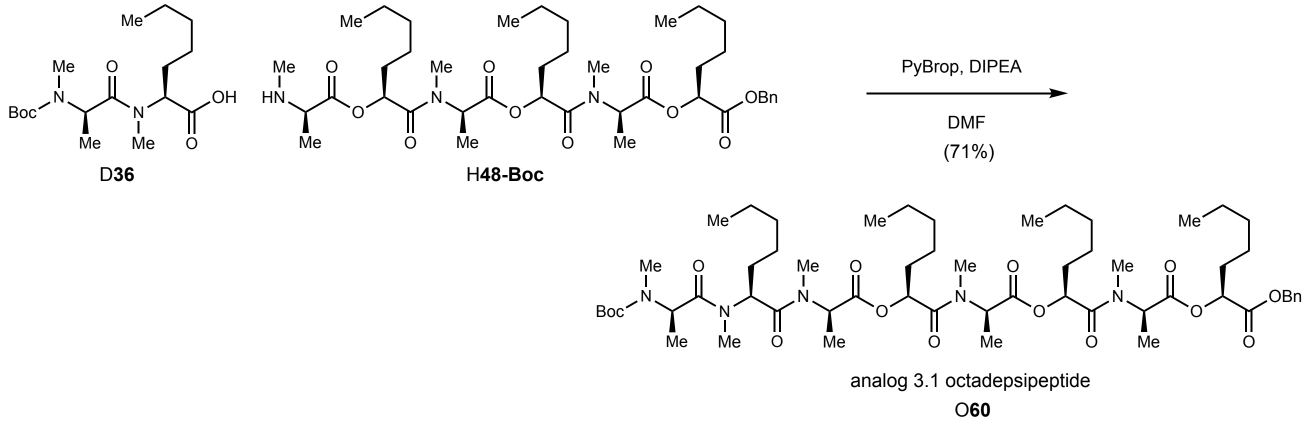

Benzyl (4*S*,7*R*,10*S*,13*R*,16*S*)-7,8,13,14-tetramethyl-3,6,9,12,15-pentaoxo-4,10-dipentyl-1-phenyl-2,5,11-trioxa-8,14-diazahenicosan-16-yl (6*R*,9*S*,12*R*)-2,2,5,6,8,11,12-heptamethyl-4,7,10-trioxo-9-pentyl-3-oxa-5,8,11-triazatridecan-13-

oate (O60). A round-bottom flask was charged with the amine (86.0 mg, 250 µmol), acid (186 mg, 250 µmol), and DMF (2.5 mL). The mixture was cooled to 0 °C and then DIPEA (130 µL, 750 µmol) and PyBrop (183 mg, 375 µmol) were added. The reaction was stirred at 0 °C for 30 min, then warmed to 23 °C for 18 h. The reaction mixture was poured into cold 10% aq citric acid and extracted with EtOAc. The combined organic layers were washed with satd aq $NaHCO_3$, brine, dried, and concentrated. The crude residue was subjected to flash column chromatography (SiO_2, 5-50% ethyl acetate in hexanes) to afford the product as a light yellow oil (189 mg, 71%). $[\alpha]_D^{24}$ + 22 (*c* 0.46, $CHCl_3$); R_f = 0.27 (25% EtOAc/hexanes); IR (film) 2956, 2931, 2860, 1745, 1698, 1455, 1390, 1258, 1150, 1092 cm^{-1}; ^1H NMR (600 MHz, $CDCl_3$) This compound exists as a mixture of rotamers causing significant peak broadening and overlap. Refer to the image of the ^1H NMR spectrum; ^{13}C NMR (150 MHz, $CDCl_3$) This compound exists in multiple conformations, causing significant peak overlap. Refer to image of the ^{13}C NMR spectrum; HRMS (EI): Exact mass calcd for $C_{57}H_{99}N_6O_{14}$ $[M+NH_4]^+$ 1091.7214, found 1091.7212. MPT-3-072

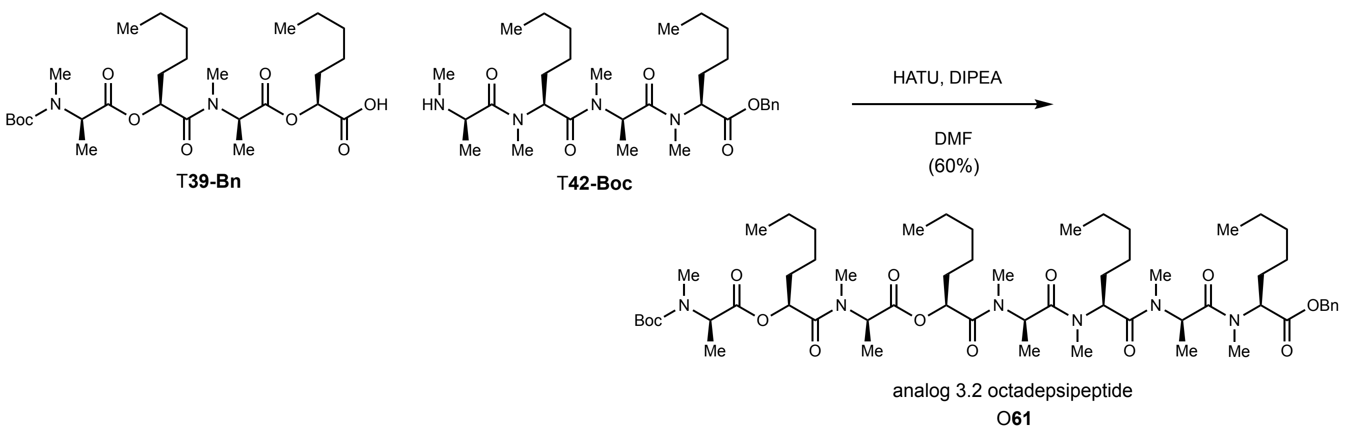

Benzyl (2*S*,5*R*,8*S*,11*R*,14*S*)-14-(((6R,9S,12R)-2,2,5,6,11,12-hexamethyl-4,7,10-trioxo-9-pentyl-3,8-dioxa-5,11-diazatridecan-13-oyl)oxy)-3,5,6,9,11,12-hexamethyl-4,7,10,13-tetraoxo-2,8-dipentyl-3,6,9,12-tetraazanonadecanoate (O61). A round-bottom flask was charged with the amine (125 mg, 230 µmol), acid (125 mg, 230 µmol), and DMF (2.3 mL). The mixture was cooled to 0 °C and then DIPEA (120 µL, 690 µmol) and HATU (262 mg, 690 µmol) were added. The reaction was stirred at 0 °C for 30 min, then warmed to 23 °C for 18 h. The reaction mixture was poured into cold 10% aq citric acid and extracted with EtOAc. The combined organic layers were washed with satd aq $NaHCO_3$, brine, dried, and concentrated. The crude residue was subjected to flash column chromatography (SiO_2, 5-50% ethyl acetate in hexanes) to afford the product as a yellow oil (149 mg, 60%). $[\alpha]_D^{24}$ - 22 (*c* 0.25, $CHCl_3$); R_f = 0.37 (30% EtOAc/hexanes); IR (film) 2955, 2933, 2858, 1745, 1699, 1450, 1258, 1156, 1092 cm^{-1}; ^1H NMR (600 MHz, $CDCl_3$) δ 7.37-7.31 (series of m, 5H), 5.24-5.20 (series of m, 5H), 5.15 (m, 2H), 5.04 (m, 1H), 4.97 (m, 1H), 4.70 (m, 1H), 3.11-2.81 (series of m, 18H), 1.88-1.80 (series of m, 6H), 1.76-1.68 (series of m, 4H), 1.57-1.52 (m, 3H), 1.45 (s, 9H), 1.41-1.38 (series of m, 6H), 1.38-1.35 (series of m, 6H), 1.34-1.27 (series of m, 19H), 0.90-0.88 (series of m, 12H); ^{13}C NMR (150 MHz, $CDCl_3$) ppm 171.3, 171.1, 170.7, 170.5, 170.1, 169.9, 169.5, 169.4, 156.0, 135.3, 128.6, 128.5, 128.3, 79.7, 73.4, 73.0, 72.1, 71.8, 71.7, 71.6, 71.4, 67.2, 67.0, 55.0, 54.9, 54.5, 53.1, 52.4, 32.1, 31.6, 31.5, 31.4, 31.3, 31.2, 31.1, 30.9, 30.7, 30.2, 29.6, 28.4, 25.1, 24.9, 24.8, 24.5, 22.5, 22.4, 22.3, 15.6, 15.5, 14.4, 14.3, 14.0, 13.98, 13.94; HRMS (EI): Exact mass calcd for $C_{58}H_{102}N_7O_{13}$ $[M+NH_4]^+$ 1104.7536, found 1104.7536. MPT-3-109

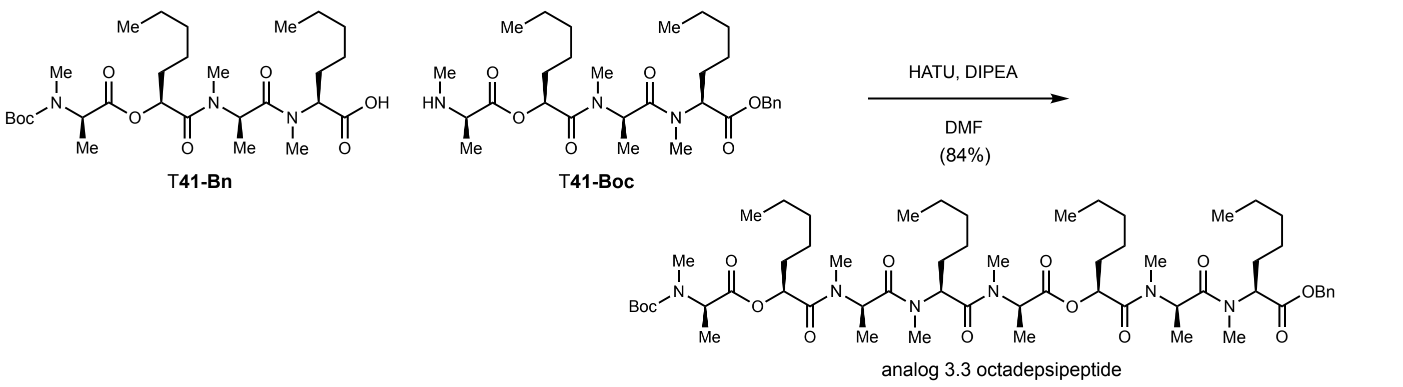

Benzyl (*S*)-2-((6*R*,9*S*,12*R*,15*S*,18*R*,21*S*,24*R*)-N,2,2,5,6,11,12,14,17,18,23,24-dodecamethyl-4,7,10,13,16,19,22-heptaoxo-9,15,21-tripentyl-3,8,20-trioxa-5,11,14,17,23-pentaazapentacosan-25-amido)heptanoate (O62). A round-bottom flask was charged with the amine (102 mg, 186 µmol), acid (104 mg, 186 µmol), and DMF (1.9 mL). The mixture was cooled to 0 °C and then DIPEA (100 µL, 559 µmol) and HATU (213 mg, 559 µmol) were added. The reaction was stirred at 0 °C for 30 min, then warmed to 23 °C for 18 h. The reaction mixture was poured into cold 10% aq citric acid and extracted with EtOAc. The combined organic layers were washed with satd aq $NaHCO_3$, brine, dried, and concentrated. The crude residue was subjected to flash column chromatography (SiO_2, 5-40% ethyl acetate in hexanes) to afford the product as a light yellow oil (170 mg, 84%). $[\alpha]_D^{24}$ - 13 (*c* 0.13, $CHCl_3$); R_f = 0.17 (25% EtOAc/hexanes); IR (film) 2927, 2858 2665, 1847, 1743, 1456, 1392, 1321, 1258, 1152, 1092 cm^{-1}; ^1H NMR (600 MHz, $CDCl_3$) This compound exists in multiple conformations, causing significant peak overlap. Refer to image of the ^1H NMR spectrum; ^{13}C NMR (150 MHz, $CDCl_3$) ppm 171.8, 171.6, 171.3, 170.7, 170.6, 167.9, 166.2, 165.6, 151.7, 135.6, 128.4, 128.2, 128.1, 80.9, 79.2, 77.5, 74.9, 71.3, 67.3, 67.2, 66.9, 66.7, 66.6, 60.7, 60.4, 60.1, 56.7, 56.5, 55.9, 54.7, 51.5, 34.7, 33.6, 32.5, 32.1, 31.8, 31.6, 31.4, 31.1, 30.4, 29.7, 29.1, 28.4, 28.2, 27.9, 25.9, 25.7, 25.2, 24.9, 24.7, 24.1, 22.4, 21.9, 21.7, 16.6, 14.6, 14.1, 13.9; HRMS (EI): Exact mass calcd for $C_{58}H_{102}N_7O_{13}$ $[M+NH_4]^+$ 1104.7536, found 1104.7532. MPT-3-020

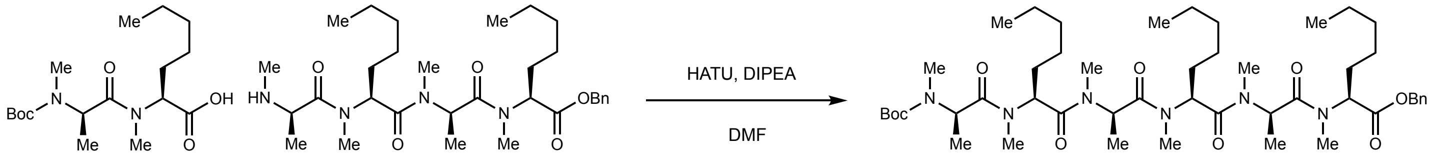

Benzyl (6*R*,9*S*,12*R*,15*S*,18*R*,21*S*)-2,2,5,6,8,11,12,14,17,18,20-undecamethyl-4,7,10,13,16,19-hexaoxo-9,15,21-tripentyl-3-oxa-5,8,11,14,17,20-hexaazadocosan-22-oate (H63). A round-bottom flask was charged with the amine (180 mg, 343 µmol), acid (118 mg, 343 µmol), and DMF (3.4 mL). The mixture was cooled to 0 °C and then DIPEA (179 µL, 1.03 mmol) and HATU (392 mg, 1.03 µmol) were added. The reaction was stirred at 0 °C for 30 min, then warmed to 23 °C for 18 h.. The reaction mixture was poured into cold 10% aq citric acid and extracted with EtOAc. The combined organic layers were washed with satd aq $NaHCO_3$, brine, dried, and concentrated. The crude residue was subjected to flash column chromatography (SiO_2, 5-50% ethyl acetate in hexanes) to afford the product as a yellow oil (179 mg, 59%). $[\alpha]_D^{24}$ + 14 (*c* 0.33, $CHCl_3$); R_f = 0.18 (30% EtOAc/hexanes); IR (film) 2954, 2931, 2860, 1744, 1699, 1649, 1455, 1390, 1322, 1259, 1156 cm^{-1}; ^1H NMR (600 MHz, $CDCl_3$) δ 7.39-7.30 (series of m, 5H), 5.26-5.20 (series of m, 4H), 5.12 (m, 1H), 5.05 (m, 1H), 4.98

(m, 1H), 4.69 (m, 1H), 3.15-2.81 (series of m, 18H), 1.86 (m, 2H), 1.73 (m, 2H), 1.59 (m, 2H), 1.47 (s, 9H), 1.40-1.35 (series of m, 8H), 1.34-1.25 (series of m, 19H), 0.90-0.88 (series of m, 9H); ^{13}C NMR (150 MHz, $CDCl_3$) ppm 171.1, 170.6, 170.1, 169.9, 169.5, 169.4, 156.0, 135.3, 128.6, 128.5, 128.3, 79.7, 73.4, 73.0, 72.1, 71.6, 67.2, 67.0, 54.9, 54.4, 53.0, 52.4, 32.0, 31.6, 31.4, 31.3, 31.2, 31.1, 30.9, 30.7, 30.2, 29.7, 29.6, 28.4, 25.1, 24.8, 24.5, 22.4, 15.6, 15.3, 14.6, 14.3, 14.0, 13.9; HRMS (EI): Exact mass calcd for $C_{48}H_{86}N_7O_9$ $[M+NH_4]^+$ 904.6487, found 904.6484. MPT-4-204

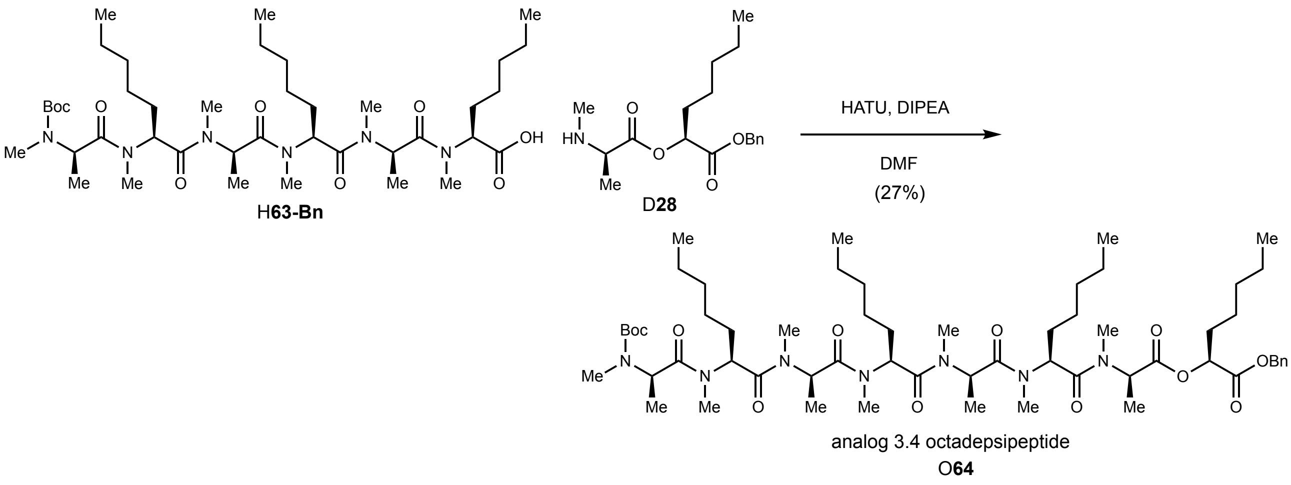

(*S*)-1-(benzyloxy)-1-oxoheptan-2-yl (6*R*,9*S*,12*R*,15*S*,18*R*,21*S*,24*R*)-2,2,5,6,8,11,12,14,17,18,20,23,24-tridecamethyl-4,7,10,13,16,19,22-heptaoxo-9,15,21-tripentyl-3-oxa-5,8,11,14,17,20,23-heptaazapentacosan-25-oate (O64). A round-bottom flask was charged with the amine (32.0 mg, 100 µmol), acid (80.0 mg, 100 µmol), and DMF (1.0 mL). The mixture was cooled to 0 °C and then DIPEA (60.0 µL, 300 µmol) and HATU (114 mg, 300 µmol) were added. The reaction was stirred at 0 °C for 30 min, then warmed to 23 °C for 18 h. The reaction mixture was poured into cold 10% aq citric acid and extracted with EtOAc. The combined organic layers were washed with satd aq $NaHCO_3$, brine, dried, and concentrated. The crude residue was subjected to flash column chromatography (SiO_2, 5-40% ethyl acetate in hexanes) to afford the product as a light yellow oil (30 mg, 27%). $[\alpha]_D^{24}$ + 2.3 (*c* 0.53, $CHCl_3$); R_f = 0.13 (30% EtOAc/hexanes); IR (film) 2956, 2928, 2858, 1742, 1651, 1456, 1393, 1218, 1155, 1083 cm^{-1}; ^{1}H NMR (600 MHz, $CDCl_3$) This compound exists in multiple conformations, causing significant peak overlap. Refer to image of the ^{1}H NMR spectrum; ^{13}C NMR (150 MHz, $CDCl_3$) ppm 172.6, 171.8, 171.1, 170.6, 170.0, 169.8, 169.5, 169.3, 156.0, 135.2, 128.6, 128.4, 128.3, 80.0, 79.8, 73.4, 73.0, 72.9, 71.5, 71.3, 71.2, 71.0, 70.9, 67.2, 67.1, 67.0, 54.9, 54.4, 54.3, 53.4, 53.3, 53.1, 52.8, 52.6, 52.5, 51.4, 31.6, 31.5, 31.4, 31.2, 30.9, 30.2, 29.7, 29.5, 28.9, 28.7, 28.3, 25.3, 25.0, 24.7, 22.6, 15.5, 14.7, 14.3, 14.0; HRMS (EI): Exact mass calcd for $C_{59}H_{105}N_8O_{12}$ $[M+NH_4]^+$ 1117.7852, found 1117.7848. MPT-5-168

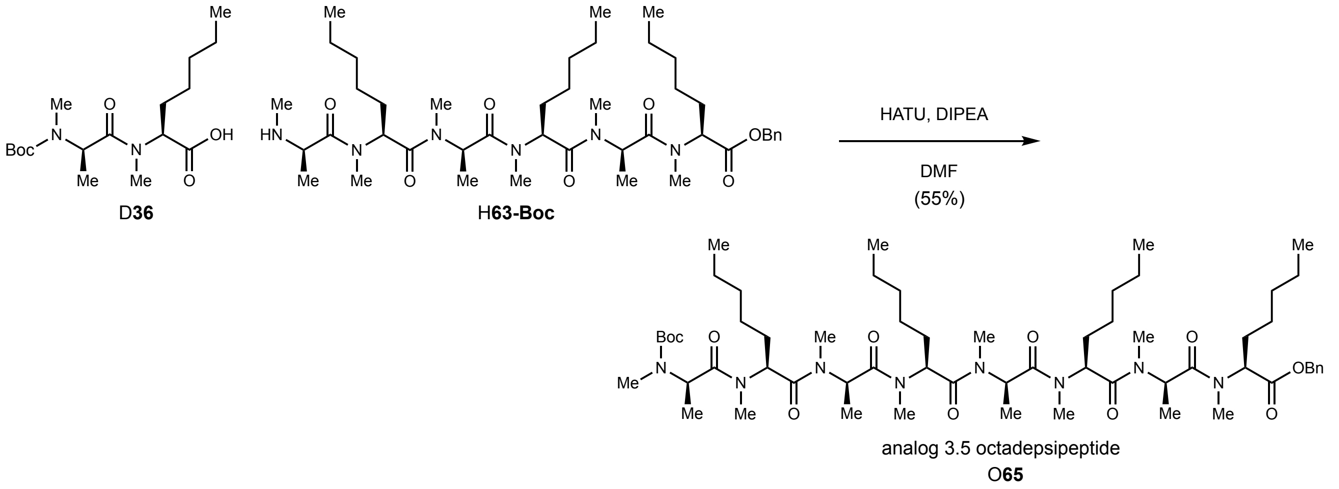

Benzyl (6*R*,9*S*,12*R*,15*S*,18*R*,21*S*,24*R*,27*S*)-2,2,5,6,8,11,12,14,17,18,20,23,24,26-tetradecamethyl-4,7,10,13,16,19,22,25-octaoxo-9,15,21,27-tetrapentyl-3-oxa-5,8,11,14,17,20,23,26-octaazaoctacosan-28-oate (O65). A round-bottom flask was charged with the amine (95.2 mg, 121 µmol), acid (41.7 mg, 121 µmol), and DMF (1.2 mL). The mixture was cooled to 0 °C and then DIPEA (63.3 µL, 363 µmol) and HATU (238 mg, 363 µmol) were added. The reaction was stirred at 0 °C for 1 h. The reaction mixture was poured into cold 10% aq citric acid and extracted with EtOAc. The combined organic layers were washed with satd aq $NaHCO_3$, brine, dried, and concentrated. The crude residue was subjected to flash column chromatography (SiO_2, 5-60% ethyl acetate in hexanes) to afford the product as a yellow oil (74.1 mg, 55%). $[\alpha]_D^{24}$ + 9.0 (*c* 0.33, $CHCl_3$); R_f = 0.23 (40% EtOAc/hexanes); IR (film) 2955, 2930, 2860, 1743, 1695, 1667, 1456, 1389, 1315, 1185 cm^{-1}; ^1H NMR (600 MHz, $CDCl_3$) δ 7.37-7.31 (series of m, 5H), 5.23-5.17 (m, 4H), 5.12 (d, *J* = 4.8 Hz, 1H), 5.09 (d, *J* = 4.8 Hz, 1H), 5.06 (series of m, 2H), 4.98 (m, 1H), 4.69 (m, 1H), 3.20-2.81 (series of m, 24H), 1.87-1.80 (series of m, 6H), 1.74-1.69 (m, 2H), 1.54 (m, 3H), 1.47 (s, 9H), 1.42-1.39 (series of m, 4H), 1.38-1.35 (series of m, 10H), 1.31-1.24 (series of m, 19H), 0.90-0.84 (series of m, 12H); ^{13}C NMR (150 MHz, $CDCl_3$) ppm 172.5, 171.7, 171.1, 170.5, 170.1, 169.9, 169.5, 169.3, 156.0, 135.3, 128.6, 128.5, 128.4, 80.0, 79.7, 73.4, 73.0, 72.0, 71.6, 71.3, 71.1, 67.2, 67.0, 55.0, 54.9, 54.4, 53.1, 52.5, 31.6, 31.4, 31.3, 31.2, 31.1, 31.0, 30.9, 30.6, 30.4, 30.2, 29.7, 29.6, 29.5, 28.4, 25.1, 24.8, 24.7, 24.5, 24.4, 22.5, 22.4, 22.3, 15.4, 15.3, 14.6, 14.3, 14.0, 13.9; HRMS (EI): Exact mass calcd for $C_{60}H_{108}N_9O_{11}$ $[M+NH_4]^+$ 1130.8168, found 1130.8166. MPT-5-184

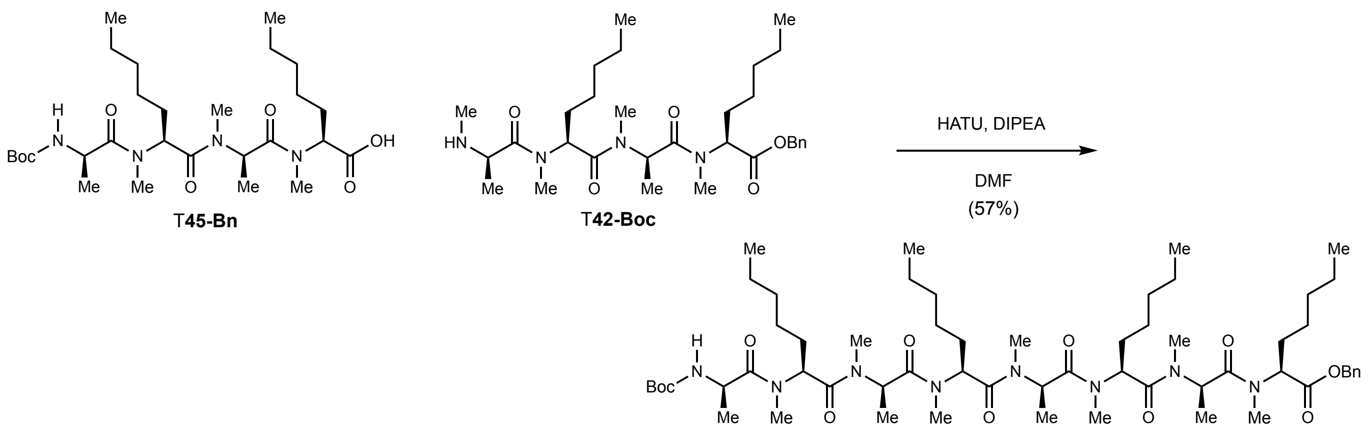

analog 4.2 octadepsipeptide
O**66**

Benzyl (6*R*,9*S*,12*R*,15*S*,18*R*,21*S*,24*R*,27*S*)-2,2,6,8,11,12,14,17,18,20,23,24,26-tridecamethyl-4,7,10,13,16,19,22,25-octaoxo-9,15,21,27-tetrapentyl-3-oxa-5,8,11,14,17,20,23,26-octaazaoctacosan-28-oate (O66). A round-bottom flask was charged with the amine (124 mg, 193 µmol), acid (124 mg, 193 µmol), and DMF (1.9 mL). The mixture was cooled to 0 °C and then DIPEA (83.0 µL, 579 µmol) and HATU (177 mg, 559 µmol) were added. The reaction was stirred at 0 °C for 30 min, then warmed to 23 °C for 18 h. The reaction mixture was poured into cold 10% aq citric acid and extracted with EtOAc. The combined organic layers were washed with satd aq $NaHCO_3$, brine, dried, and concentrated. The crude residue was subjected to flash column chromatography (SiO_2, 5-50% ethyl acetate in hexanes) to afford the product as a yellow oil (121 mg, 57%). $[\alpha]_D^{24}$ - 19 (*c* 0.31, $CHCl_3$); R_f = 0.19 (30% EtOAc/hexanes); IR (film) 3650, 2958, 2932, 2861, 1731, 1660, 1466, 1408, 1381, 1141 cm^{-1}; ^1H NMR (600 MHz, $CDCl_3$) δ 7.31-7.25 (series of m, 6H), 5.17-5.13 (m, 5H), 5.06 (d, *J* = 4.6 Hz, 1H), 5.05 (d, *J* = 4.6 Hz, 1H), 4.97 (m, 1H), 4.92 (m, 1H), 4.64 (m, 1H), 3.12-2.64 (series of m, 21H), 1.81-1.74 (series of m, 6H), 1.70-1.62 (m, 4H), 1.48 (m, 3H), 1.41 (s, 9H), 1.36-1.33 (series of m, 4H), 1.32-1.29 (series of m, 8H), 1.26-1.16 (series of m, 19H), 0.85-0.73 (series of m, 12H); ^{13}C NMR (150 MHz, $CDCl_3$) 171.7, 171.4, 171.1, 170.8, 170.3, 170.1, 169.9, 169.5, 156.0, 135.3, 128.7, 128.4, 128.3, 79.7, 73.4, 73.0, 72.1, 72.0, 71.7, 71.6, 71.4, 67.6, 67.2, 67.0, 55.0, 54.9, 53.3, 52.4, 46.1, (29) 31.6, 31.5, 31.4, 31.3, 31.2, 31.1, 30.8, 30.7, 30.6, 30.7, 30.2, 29.6, 28.9, 28.3, 25.3, 25.1, 24.8, 24.5, 22.6, 22.5, 22.4, 22.3, 15.4, 14.7, 14.3, 14.0; HRMS (EI): Exact mass calcd for $C_{59}H_{106}N_9O_{11}$ $[M+NH_4]^+$ 1116.8012, found 1116.8007 MPT-4-178

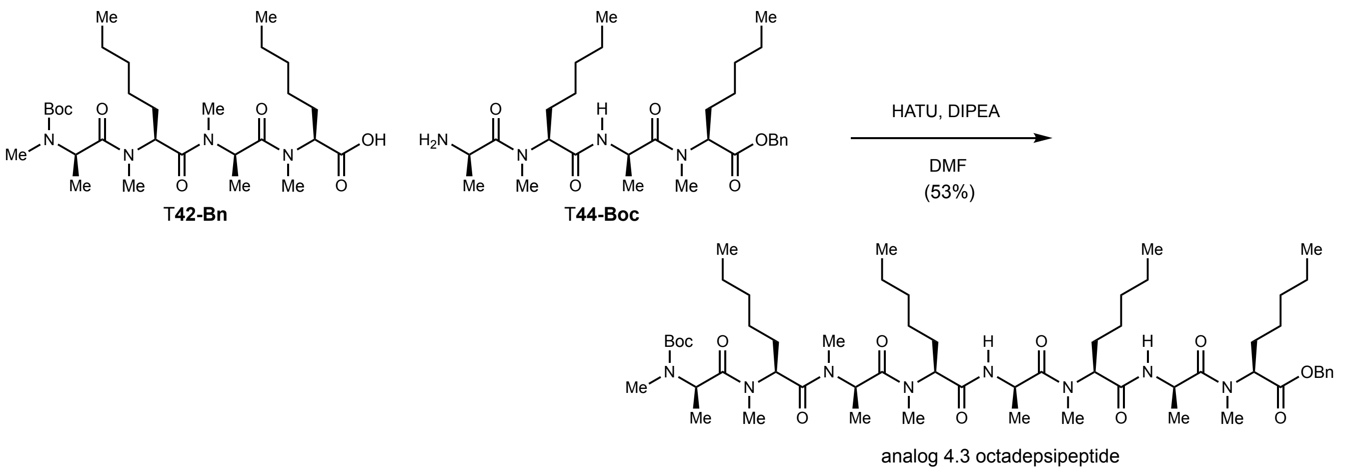

Benzyl (6*R*,9*S*,12*R*,15*S*,18*R*,21*S*,24*R*,27*S*)-2,2,5,6,8,11,12,14,18,20,24,26-dodecamethyl-4,7,10,13,16,19,22,25-octaoxo-9,15,21,27-tetrapentyl-3-oxa-5,8,11,14,17,20,23,26-octaazaoctacosan-28-oate (O67). A round-bottom flask was charged with the amine (63.6 mg, 111 µmol), acid (59.3 mg, 111 µmol), and DMF (1.1 mL). The mixture was cooled to 0 °C and then DIPEA (49.4 µL, 333 µmol) and HATU (105 mg, 333 µmol) were added. The reaction was stirred at 0 °C for 30 min, then warmed to 23 °C for 18 h. The reaction mixture was poured into cold 10% aq citric acid and extracted with EtOAc. The combined organic layers were washed with satd aq $NaHCO_3$, brine, dried, and concentrated. The crude residue was subjected to flash column chromatography (SiO_2, 5-50% ethyl acetate in hexanes) to afford the product as a yellow oil (64.1 mg, 53%). $[\alpha]_D^{24}$ - 11 (*c* 0.27, $CHCl_3$); R_f = 0.22 (30% EtOAc/hexanes); IR (film) 3322, 2921, 2862, 1748, 1669, 1539, 1456, 1392, 1158, 1092 cm^{-1}; ^1H NMR (600 MHz, $CDCl_3$) δ 7.40-7.32 (series of m, 7H), 5.31 (m, 1H), 5.22-5.12 (series of m, 5H), 5.06 (m, 1H), 4.99 (m, 1H), 4.70 (m, 2H), 3.36-2.80 (series of m, 18H), 1.82 (m, 2H), 1.73 (m, 2H), 1.63 (m, 2H), 1.56-1.51 (series of m, 6H), 1.46 (m, 9H), 1.41-1.37 (series of m, 6H), 1.32-1.29 (series of m, 6H), 1.27-1.20 (series of m, 20H), 0.90-0.83 (series of m, 12H); ^{13}C NMR (150 MHz, $CDCl_3$) ppm 171.8, 171.1, 170.5, 170, 169.9, 169.8, 169.5, 169.3, 156.0, 135.3, 128.6, 128.4, 128.3, 79.8, 73.4, 73.0, 71.4, 67.2, 67.0, 54.1, 52.7, 52.5, 31.7, 31.6, 31.55, 31.5, 31.4, 31.37, 31.3, 31.2, 31.1, 31.0, 30.9, 30.8, 30.4, 30.3, 30.2, 30.1, 29.7, 29.6, 29.4, 28.3, 25.0, 24.8, 24.7, 24.4, 22.42, 22.4, 22.32, 22.3, 15.3, 14.6, 14.2, 14.0; HRMS (EI): Exact mass calcd for $C_{58}H_{104}N_9O_{11}$ $[M+NH_4]^+$ 1102.7855, found 1102.7850. MPT-4-246

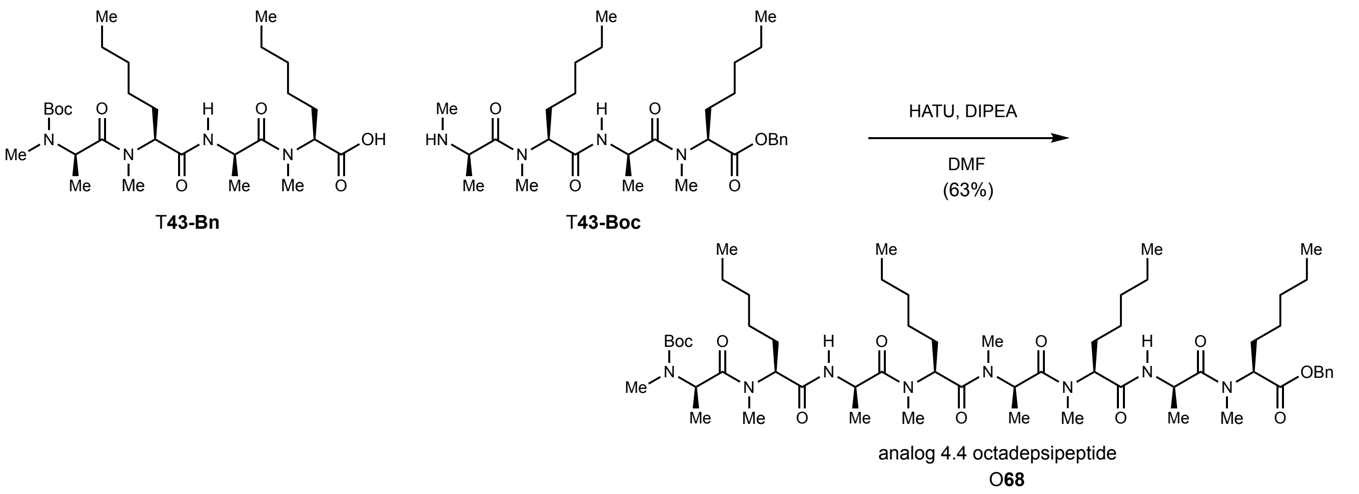

Benzyl (6*R*,9*S*,12*R*,15*S*,18*R*,21*S*,24*R*,27*S*)-2,2,5,6,8,12,14,17,18,20,24,26-dodecamethyl-4,7,10,13,16,19,22,25-octaoxo-9,15,21,27-tetrapentyl-3-oxa-5,8,11,14,17,20,23,26-octaazaoctacosan-28-oate (O68). A round-bottom flask was charged with the amine (83.0 mg, 149 µmol), acid (81.3 mg, 149 µmol), and DMF (1.5 mL). The mixture was cooled to 0 °C and then DIPEA (80.0 µL, 448 µmol) and HATU (171 mg, 559 µmol) were added. The reaction was stirred at 0 °C for 30 min, then warmed to 23 °C for 18 h. The reaction mixture was poured into cold 10% aq citric acid and extracted with EtOAc. The combined organic layers were washed with satd aq $NaHCO_3$, brine, dried, and concentrated. The crude residue was subjected to flash column chromatography (SiO_2, 5-40% ethyl acetate in hexanes) to afford the product as a light yellow oil (102 mg, 63%). $[\alpha]_D^{24}$ - 18 (*c* 0.16, $CHCl_3$); R_f = 0.10 (25% EtOAc/hexanes); IR (film) 3418, 2958, 2925, 2854, 1740, 1644, 1557, 1540, 1375, 1260, 1080 cm^{-1}; ^1H NMR (600 MHz, $CDCl_3$) δ 7.40-7.29 (series of m, 6H), 7.09 (m, 1H), 5.29 (m, 1H), 5.22-5.08 (series of m, 4H), 5.03 (m, 1H), 4.96 (m, 1H), 4.90-4.72 (series of m, 3H), 3.03-2.71 (series of m, 18H), 2.02-1.88 (series of m, 3H), 1.65 (br s, 9H), 1.48-1.44 (series of m, 10H), 1.35-1.23 (series of m, 28H), 0.90-0.83 (series of m, 12H); ^{13}C NMR (150 MHz, $CDCl_3$) This compound exists in multiple conformations, causing significant peak overlap. Refer to image of the ^{13}C NMR spectrum; HRMS (EI): Exact mass calcd for $C_{58}H_{104}N_9O_{11}$ $[M+NH_4]^+$ 1102.7855, found 1102.7850. MPT-3-087

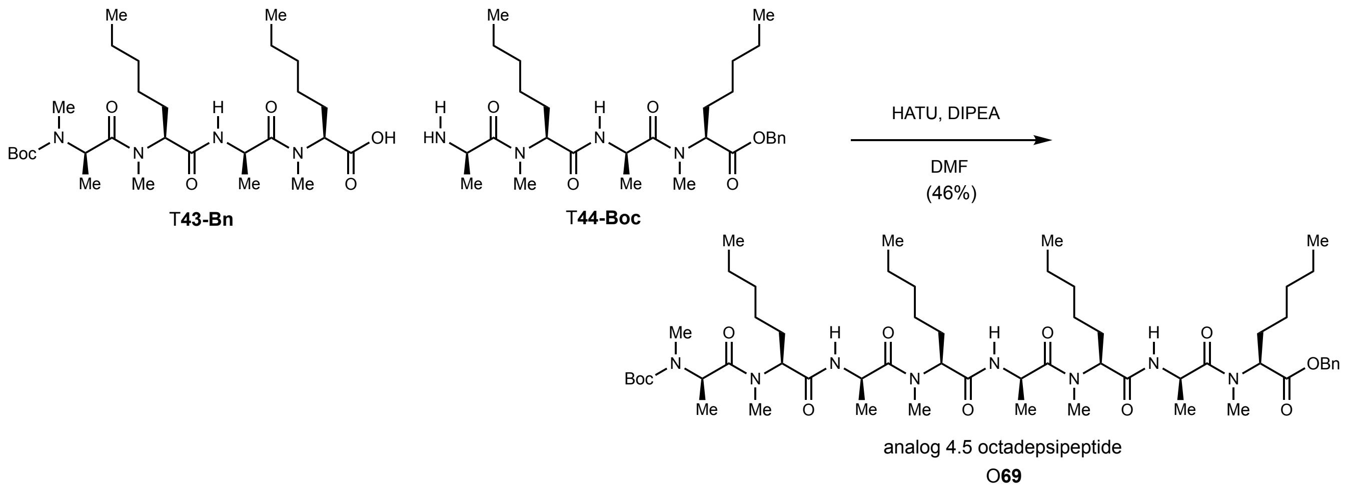

Benzyl (6*R*,9*S*,12*R*,15*S*,18*R*,21*S*,24*R*,27*S*)-2,2,5,6,8,12,14,18,20,24,26-undecamethyl-4,7,10,13,16,19,22,25-octaoxo-9,15,21,27-tetrapentyl-3-oxa-5,8,11,14,17,20,23,26-octaazaoctacosan-28-oate (O69). A round-bottom flask was charged with the amine (85.8 mg, 154 μmol), acid (82.0 mg, 154 μmol), and DMF (1.5 mL). The mixture was cooled to 0 °C and then DIPEA (48.7 μL, 462 μmol) and HATU (240 mg, 462 μmol) were added. The reaction was stirred at 0 °C for 30 min, then warmed to 23 °C for 18 h. The reaction mixture was poured into cold 10% aq citric acid and extracted with EtOAc. The combined organic layers were washed with satd aq $NaHCO_3$, brine, dried, and concentrated. The crude residue was subjected to flash column chromatography (SiO_2, 5-65% ethyl acetate in hexanes) to afford the product as a light yellow oil (76 mg, 46%). $[\alpha]_D^{24}$ - 29 (*c* 0.39, $CHCl_3$); R_f = 0.15 (40% EtOAc/hexanes); IR (film) 3419, 2960, 2923, 2853, 1747, 1652, 1540, 1456, 1378, 1261, 1100 cm^{-1}; ^{1}H NMR (600 MHz, $CDCl_3$) δ 7.32-7.24 (series of m, 5H), 7.20-7.09 (*N*-H, series of m, 3H), 5.22-5.09 (series of m, 6H), 5.05 (m, 1H), 4.97 (m, 1H), 4.86 (m, 1H), 4.63 (m, 1H), 3.29-2.69 (series of m, 15H), 1.81-1.75 (series of m, 4H), 1.66 (m, 3H), 1.54 (s, 6H), 1.49 (m, 3H), 1.38-1.28 (series of m, 18H), 1.25-1.17 (series of m, 19H), 0.83-0.77 (series of m, 12H); ^{13}C NMR (150 MHz, $CDCl_3$) ppm 172.0, 171.4, 171.1, 170.8, 170.2, 170.0, 169.5, 169.3, 155.0, 135.3, 128.9, 128.6, 128.3, 80.0, 79.7, 73.5, 73.0, 72.3, 71.7, 71.4, 68.6, 67.3, 60.5, 58.8, 55.0, 53.0, 52.5, 35.1, 34.7, (29) 32.1, 31.7, 31.5, 31.4, 31.3, 31.2, 31.1, 30.9, 30.8, 30.7, 29.7, 29.6, 28.2, 28.1, 25.1, 24.8, 24.5, 24.1, 22.5, 22.3, 15.5, 14.3, 14.0, 13.9; HRMS (EI): Exact mass calcd for $C_{57}H_{102}N_9O_{11}$ $[M+NH_4]^+$ 1088.7699, found 1088.7696. MPT-5-237

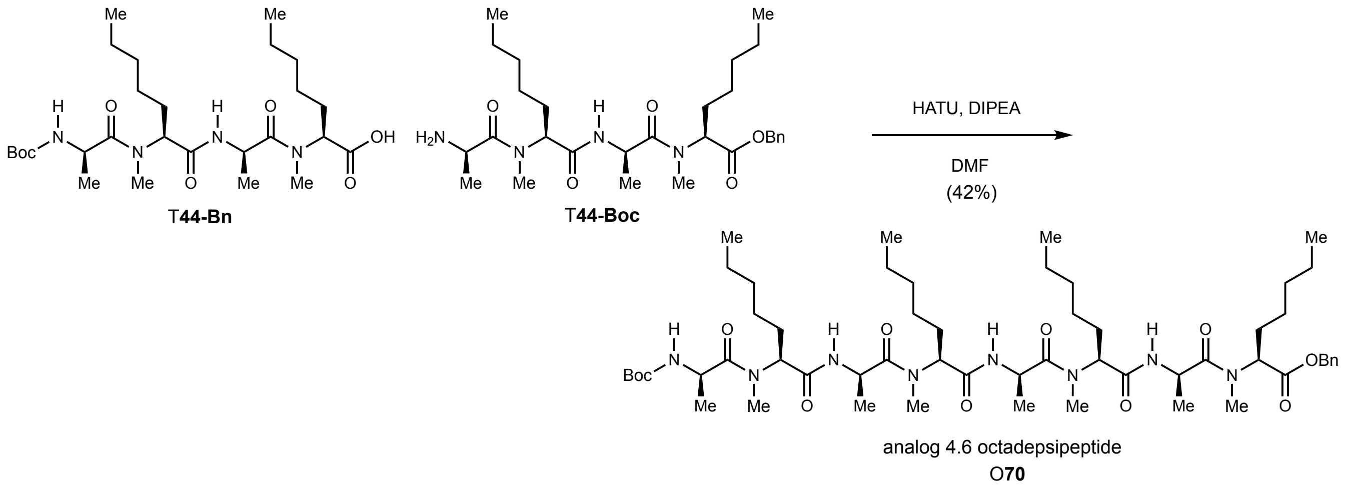

Benzyl (6*R*,9*S*,12*R*,15*S*,18*R*,21*S*,24*R*,27*S*)-2,2,6,8,12,14,18,20,24,26-decamethyl-4,7,10,13,16,19,22,25-octaoxo-9,15,21,27-tetrapentyl-3-oxa-5,8,11,14,17,20,23,26-octaazaoctacosan-28-oate (O70). A round-bottom flask was charged with the amine (170 mg, 316 µmol), acid (170 mg, 316 µmol), and DMF (3.0 mL). The mixture was cooled to 0 °C and then DIPEA (136 µL, 948 µmol) and HATU (291 mg, 559 µmol) were added. The reaction was stirred at 0 °C for 30 min, then warmed to 23 °C for 18 h. The reaction mixture was poured into cold 10% aq citric acid and extracted with EtOAc. The combined organic layers were washed with satd aq $NaHCO_3$, brine, dried, and concentrated. The crude residue was subjected to flash column chromatography (SiO_2, 5-50% ethyl acetate in hexanes) to afford the product as a light yellow oil (139 mg, 42%). $[\alpha]_D^{24}$ - 16 (*c* 0.22, $CHCl_3$); R_f = 0.11 (40% EtOAc/hexanes); IR (film) 3444, 2960, 2931, 2863, 1717, 1694, 1682, 1443, 1326, 1206, 1137 cm^{-1}; ^1H NMR (600 MHz, $CDCl_3$) This compound exists as a mixture of rotamers causing significant peak broadening and overlap. Refer to the image of the ^1H NMR spectrum; ^{13}C NMR (150 MHz, $CDCl_3$) ppm 172.5, 172.0, 170.9, 170.2, 169.5, 167.3, 166.7, 166.0, 156.0, 134.9, 128.7, 128.6, 128.5, 79.8, 71.5, 67.3, 61.6, 61.1, 55.0, 54.5, 53.4, 51.7, 46.1, 32.2, 31.8, 31.5, 31.4, 31.3, 31.1, 31.0, 30.8, 30.7, 30.6, 30.3, 30.2, 29.8, 29.6, 28.4, 26.2, 25.0, 24.9, 24.8, 24.7, 24.0, 22.4, 15.6, 15.3, 14.7, 14.4, 14.1, 14.0, 13.9; HRMS (EI): Exact mass calcd for $C_{56}H_{100}N_9O_{11}$ $[M+NH_4]^+$ 1074.7542, found 1074.7538. MPT-4-067

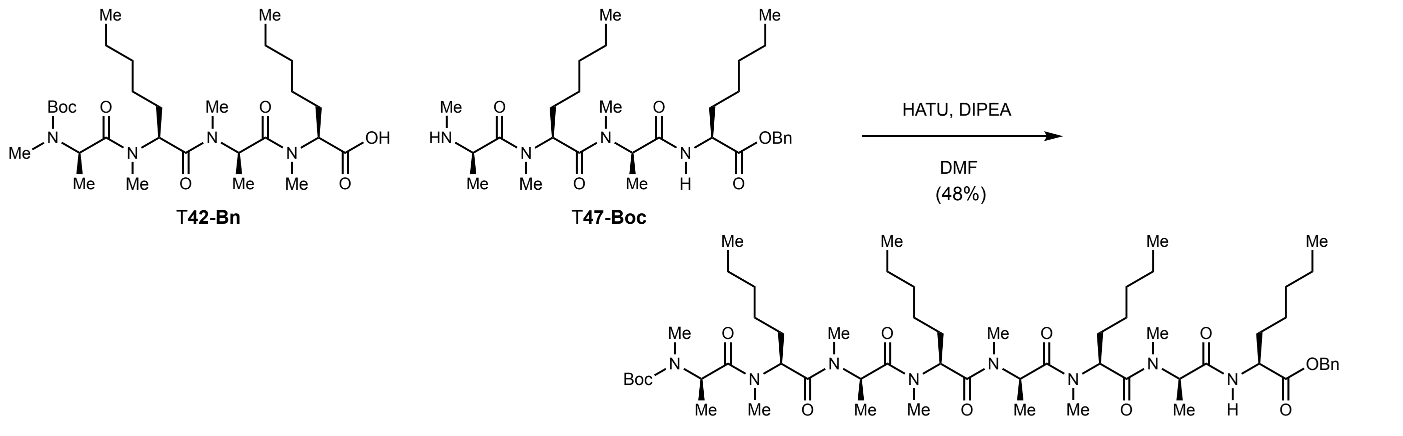

Benzyl (6*R*,9*S*,12*R*,15*S*,18*R*,21*S*,24*R*,27*S*)-2,2,5,6,8,11,12,14,17,18,20,23,24-tridecamethyl-4,7,10,13,16,19,22,25-octaoxo-9,15,21,27-tetrapentyl-3-oxa-5,8,11,14,17,20,23,26-octaazaoctacosan-28-oate (O71). A round-bottom flask was charged with the amine (103 mg, 181 µmol), acid (99.0 mg, 181 µmol), and DMF (1.8 mL). The mixture was cooled to 0 °C and then DIPEA (57.2 µL, 543 µmol) and HATU (282 mg, 543 µmol) were added. The reaction was stirred at 0 °C for 30 min, then warmed to 23 °C for 18 h. The reaction mixture was poured into cold 10% aq citric acid and extracted with EtOAc. The combined organic layers were washed with satd aq $NaHCO_3$, brine, dried, and concentrated. The crude residue was subjected to flash column chromatography (SiO_2, 5-60% ethyl acetate in hexanes) to afford the product as a light yellow oil (95.9 mg, 48%). $[\alpha]_D^{24}$ - 13 (*c* 0.86, $CHCl_3$); R_f = 0.16 (50% EtOAc/hexanes); IR (film) 3317, 2956, 2930, 2859, 1739, 1644, 1525, 1458, 1369, 1275, 1090 cm^{-1}; ^1H NMR (600 MHz, $CDCl_3$) δ 7.57 and 6.57 (br m, 1H), 7.41-7.32 (series of m, 5H), 5.23-4.53 (series of m, 10H), 3.16-2.66 (series of m, 21H), 1.83 (m, 3H), 1.77-1.62 (series of m, 5H), 1.52-1.36 (series of m, 20H), 1.38-1.24 (series of m, 25H), 0.91-0.84 (series of m, 12H); ^{13}C NMR (150 MHz, $CDCl_3$) ppm 172.4, 172.2, 172.0, 171.5, 171.2, 170.5, 169.8, 169.6, 156.2, 135.3, 128.5, 128.3, 128.2, 80.9, 74.5, 74.2, 73.7, 72.8, 66.9, 57.4, 49.5, 49.1, 47.9, 34.9, 31.6, 31.4, 31.35, 31.27, 31.2, 31.0, 30.9, 30.8, 29.7, 29.6, 29.3, 28.4, 28.3, 28.2, 24.61, 24.57, 24.50, 24.4, 24.2, 23.9, 22.6,

22.4, 22.3, 22.3, 17.4, 16.4, 14.7, 13.92, 13.89, 13.87, 13.83; HRMS (EI): Exact mass calcd for $C_{59}H_{106}N_9O_{11}$ $[M+NH_4]^+$ 1116.8012, found 1116.8009. MPT-4-105

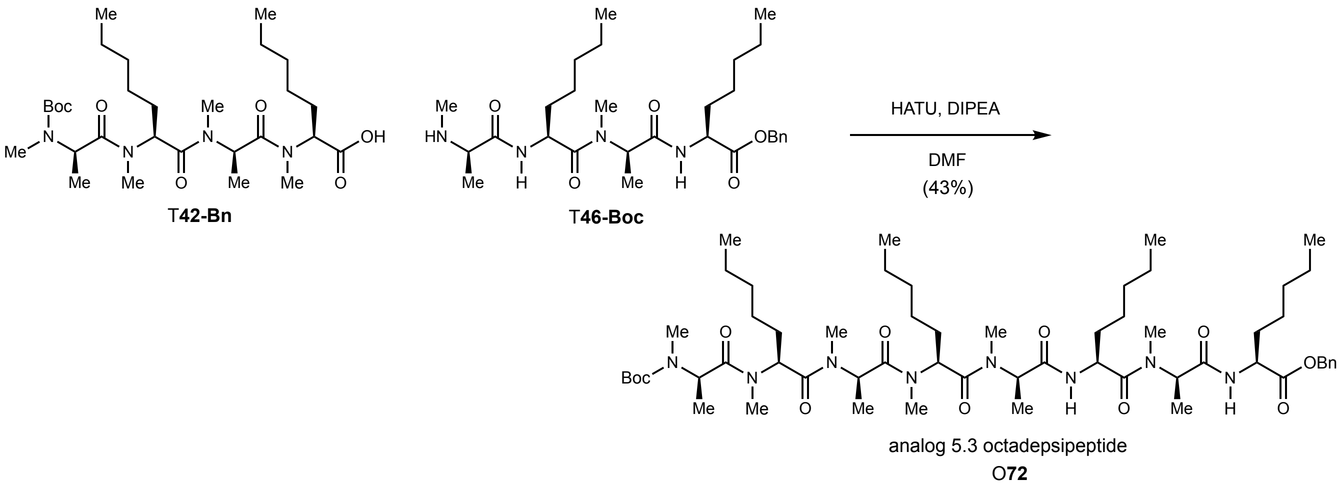

Benzyl (6*R*,9*S*,12*R*,15*S*,18*R*,21*S*,24*R*,27*S*)-2,2,5,6,8,11,12,14,17,18,23,24-dodecamethyl-4,7,10,13,16,19,22,25-octaoxo-9,15,21,27-tetrapentyl-3-oxa-5,8,11,14,17,20,23,26-octaazaoctacosan-28-oate (O72). A round-bottom flask was charged with the amine (104 mg, 196 µmol), acid (112 mg, 196 µmol), and DMF (2.0 mL). The mixture was cooled to 0 °C and then DIPEA (62.0 µL, 588 µmol) and HATU (305 mg, 588 µmol) were added. The reaction was stirred at 0 °C for 30 min, then warmed to 23 °C for 18 h. The reaction mixture was poured into cold 10% aq citric acid and extracted with EtOAc. The combined organic layers were washed with satd aq $NaHCO_3$, brine, dried, and concentrated. The crude residue was subjected to flash column chromatography (SiO_2, 5-60% ethyl acetate in hexanes) to afford the product as a light yellow oil (90.2 mg, 43%). $[\alpha]_D^{24}$ - 12 (*c* 0.56, $CHCl_3$); R_f = 0.13 (50% EtOAc/hexanes); IR (film) 3300, 2957, 2933, 2861, 1742, 1650, 1458, 1408, 1275, 1086 cm^{-1}; ^1H NMR (600 MHz, $(CD_3)_2CO$) δ 7.29-7.18 (series of m, 5H), 7.07 (br d, *J* = 7.9, 1H), 7.02 (br d, *J* = 7.6, 1H), 5.22-3.55 (series of m, 10H), 2.96-2.61 (series of m, 18H), 1.88-1.63 (series of m, 6H), 1.60-1.42 (series of m, 3H), 1.34-1.09 (series of m, 44H), 0.85-0.69 (series of m, 12H); ^{13}C NMR (150 MHz, $CDCl_3$) ppm 172.4, 171.1, 170.7, 170.1, 169.9, 169.7, 169.5, 169.3, 155.0, 135.3, 128.6, 128.5, 128.3, 79.6, 73.4, 73.0, 71.7, 71.6, 67.0, 55.0, 52.5, 49.3, 46.1, 42.7, 32.1, 31.6, 31.5, 31.43, 31.38, 31.3, 31.2, 31.1, 30.9, 30.8, 30.7, 29.7, 29.5, 28.3, 25.1, 24.9, 24.9, 24.8, 24.5, 22.42, 22.39, 22.3, 18.7, 18.4, 15.6, 15.4, 14.3, 14.3, 14.0, 13.9; HRMS (EI): Exact mass calcd for $C_{46}H_{85}N_8O_9$ $[M-Boc-Bn]^-$ 893.6445, found 893.6446. MPT-4-008

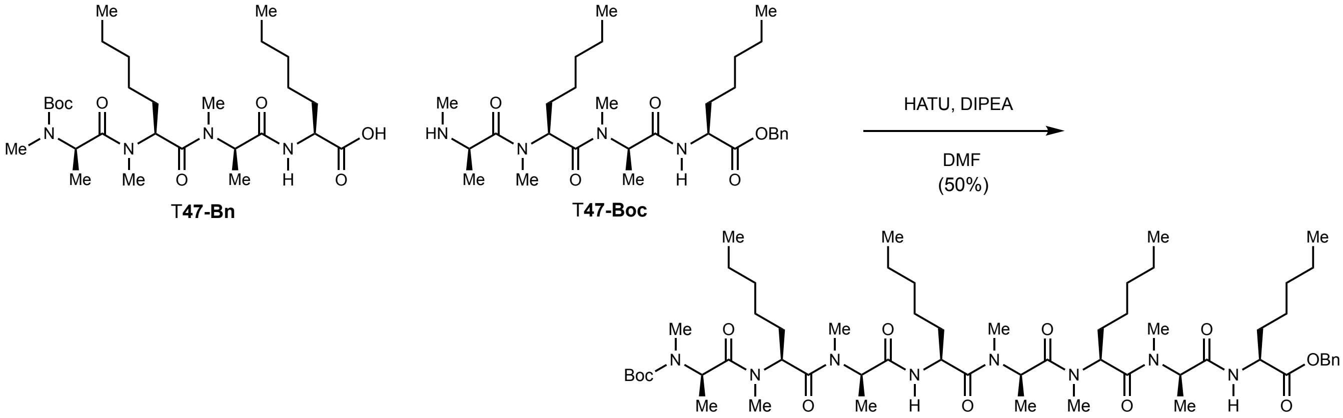

analog 5.4 octadepsipeptide
O**73**

Benzyl (6*R*,9*S*,12*R*,15*S*,18*R*,21*S*,24*R*,27*S*)-2,2,5,6,8,11,12,17,18,20,23,24-dodecamethyl-4,7,10,13,16,19,22,25-octaoxo-9,15,21,27-tetrapentyl-3-oxa-5,8,11,14,17,20,23,26-octaazaoctacosan-28-oate (O73). A round-bottom flask was charged with the amine (68.8 mg, 124 µmol), acid (68.0 mg, 124 µmol), and DMF (1.2 mL). The mixture was cooled to 0 °C and then DIPEA (39.2 µL, 372 µmol) and HATU (193 mg, 462 µmol) were added. The reaction was stirred at 0 °C for 30 min, then warmed to 23 °C for 18 h. The reaction mixture was poured into cold 10% aq citric acid and extracted with EtOAc. The combined organic layers were washed with satd aq $NaHCO_3$, brine, dried, and concentrated. The crude residue was subjected to flash column chromatography (SiO_2, 5-60% ethyl acetate in hexanes) to afford the product as a light yellow oil (66.9 mg, 50%). $[\alpha]_D^{24}$ - 27 (*c* 0.34, $CHCl_3$); R_f = 0.152 (25% EtOAc/hexanes); IR (film) 3384, 2955, 2932, 2861, 1742, 1651, 1522, 1417, 1392, 1215, 1093 cm^{-1}; ^1H NMR (600 MHz, $CDCl_3$) This compound exists in multiple conformations, causing significant peak overlap. Refer to image of the ^1H NMR spectrum; ^{13}C NMR (150 MHz, $CDCl_3$) ppm 171.7, 171.6, 171.3, 171.3, 170.7, 170.5, 170.0, 169.3, 155.6, 135.2, 128.6, 128.4, 128.3, 79.9, 79.6, 73.3, 72.9, 72.7, 71.4, 71.3, 71.2, 66.9, 57.5, 56.7, 55.7, 54.5, 38.8, 37.6, 37.2, 37.0, 36.6, 31.4, 31.3, 31.1, 30.9, 30.8, 30.3, 29.8, 29.6, 28.3, 24.9, 24.7, 24.5, 24.4, 23.3, 23.2, 22.8, 22.3, 22.2, 21.0, 14.0, 13.9, 13.84, 13.82; HRMS (EI): Exact mass calcd for $C_{58}H_{104}N_9O_{11}$ $[M+NH_4]^+$ 1102.7855, found 1102.7855. MPT-5-190

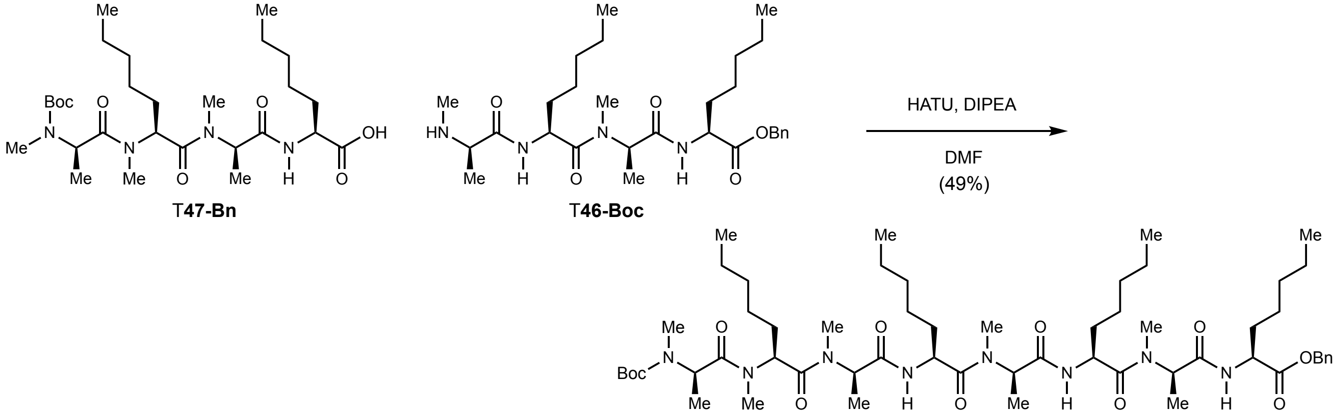

analog 5.5 octadepsipeptide
O**74**

Benzyl (6*R*,9*S*,12*R*,15*S*,18*R*,21*S*,24*R*,27*S*)-2,2,5,6,8,11,12,17,18,23,24-undecamethyl-4,7,10,13,16,19,22,25-octaoxo-9,15,21,27-tetrapentyl-3-oxa-5,8,11,14,17,20,23,26-octaazaoctacosan-28-oate (O74). A round-bottom flask was charged with the amine (104 mg, 187 μmol), acid (100 mg, 187 μmol), and DMF (1.9 mL). The mixture was cooled to 0 °C and then DIPEA (59.1 μL, 561 μmol) and HATU (292 mg, 561 μmol) were added. The reaction was stirred at 0 °C for 30 min, then warmed to 23 °C for 18 h. The reaction mixture was poured into cold 10% aq citric acid and extracted with EtOAc. The combined organic layers were washed with satd aq $NaHCO_3$, brine, dried, and concentrated. The crude residue was subjected to flash column chromatography (SiO_2, 5-60% ethyl acetate in hexanes) to afford the product as a light yellow oil (98.0 mg, 49%). $[\alpha]_D^{24}$ - 12 (*c* 0.25, $CHCl_3$); R_f = 0.22 (40% EtOAc/hexanes); IR (film) 3429, 2960, 2931, 2862, 1694, 1651, 1633, 1443, 1326, 1206, 1137 cm^{-1}; ^1H NMR (600 MHz, $CDCl_3$) This compound exists in multiple conformations, causing significant peak overlap. Refer to image of the ^1H NMR spectrum; ^{13}C NMR (150 MHz, $CDCl_3$) ppm 172.4, 171.1, 170.8, 170.4, 169.8, 169.5, 168.9, 168.6, 156.0, 135.2, 128.6, 128.5, 128.3, 80.1, 73.4, 73.1, 73.0, 72.2, 71.9, 71.6, 71.2, 71.1, 67.1, 54.9, 54.4, 53.5, 53.4, 50.8, 49.3, 36.0, 35.9, 32.2, 32.1, 31.3, 31.2, 30.9, 30.7, 30.4, 30.1, 29.7, 28.3, 25.0, 24.9, 24.8, 24.8, 24.6, 24.5, 22.4, 22.3, 15.5, 14.6, 14.2, 13.9; HRMS (EI): Exact mass calcd for $C_{57}H_{108}N_9O_{11}$ $[M+NH_4]^+$ 1088.7699, found 1088.7695. MPT-4-181

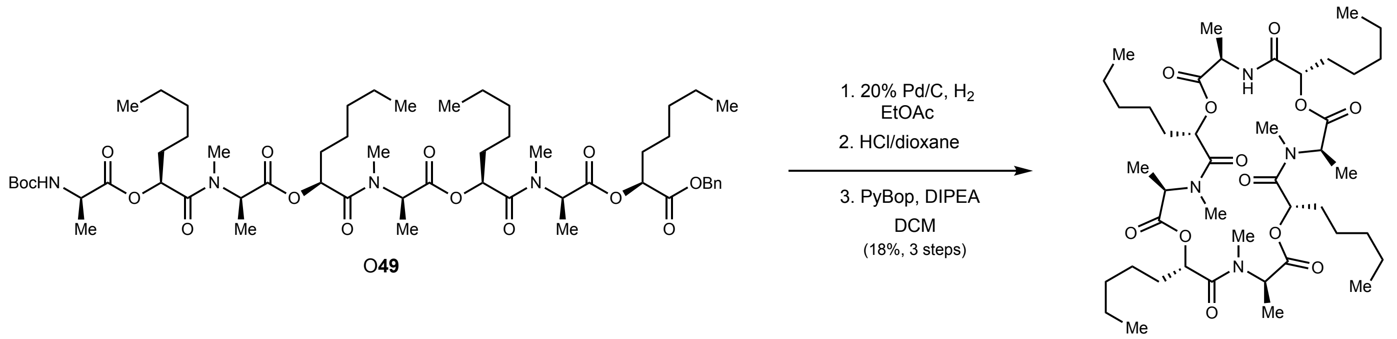

(3*R*,6*S*,9*R*,12*S*,15*R*,18*S*,21*R*,24*S*)-3,4,9,10,15,16,21-heptamethyl-6,12,18,24-tetrapentyl-1,7,13,19-tetraoxa-4,10,16,22-tetraazacyclotetracosan-2,5,8,11,14,17,20,23-octaone (1.1). A round-bottom flask was charged with the depsipeptide (108 mg, 103 μmol), dissolved in EtOAc (1.0 mL), and treated with 10% Pd/C (2.2 mg). The reaction flask was evacuated with light vacuum. Hydrogen (balloon) was added, and the flask was cycled once more. The reaction was allowed to stir for 1 h. After purging the flask with argon, the crude reaction mixture was filtered through Celite. To the crude material was added HCl/dioxane (1.0 mL) and the reaction mixture was allowed to stir for 30 min. The reaction mixture was concentrated and added to a flame-dried round bottom flask. DCM (20.8 mL) was added and the reaction was cooled to 0 °C. Once at 0 °C, DIPEA (40.0 μL, 228 μmol) and PyBop (56.7 mg, 108 μmol) were added. The reaction was stirred at 0 °C for 1.5 h, and then allowed to warm to ambient temperature to stir for an additional 1 h. The reaction mixture was poured into cold 10% aq citric acid and extracted with DCM. The combined organic layers were washed with satd aq $NaHCO_3$, brine, and then dried and concentrated. Preparative HPLC purification (5 – 95% aqueous acetonitrile, 210 nm, flow rate: 18 mL/min, R_t = 20.2 m) afforded the 24-membered macrocycle (16.8 mg, 18%) as a light yellow oil. $[\alpha]_D^{24}$ +25 (*c* 0.18, $CHCl_3$); R_f = 0.34 (60% EtOAc in hexanes); IR (film) 3331, 2927, 2855, 2359, 1747, 1653, 15457, 1196 cm^{-1}; ^1H NMR

(600 MHz, $CDCl_3$) This compound exists in multiple conformations, causing significant peak overlap. Refer to image of the ^1H NMR spectrum; ^{13}C NMR (150 MHz, $CDCl_3$) This compound exists in multiple conformations, causing significant peak overlap. Refer to image of the ^{13}C NMR spectrum; HRMS (EI): Exact mass calcd for $C_{43}H_{76}N_4O_{13}$ $[M+CH_3OH]^+$ 856.5409 found [856.5443]. MPT-3-261

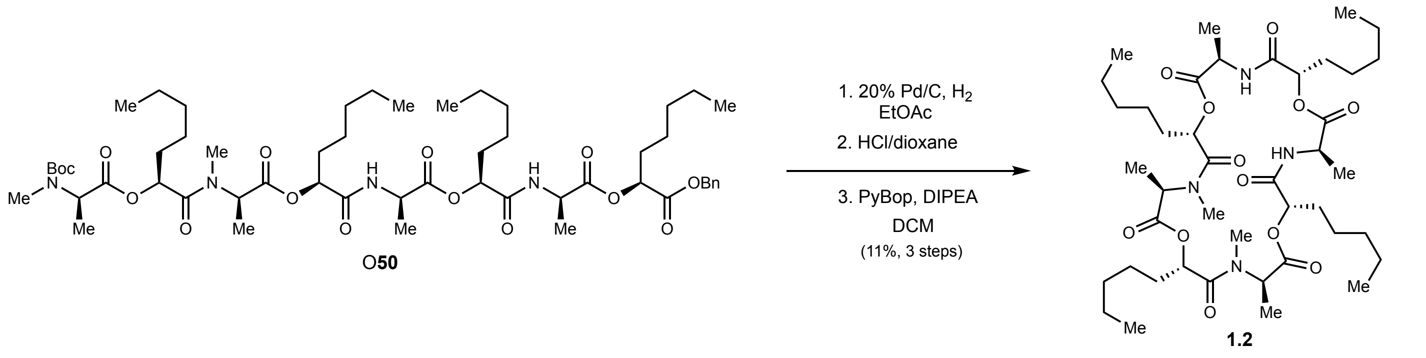

(3*R*,6*S*,9*R*,12*S*,15*R*,18*S*,21*R*,24*S*)-3,4,9,10,15,21-hexamethyl-6,12,18,24-tetrapentyl-1,7,13,19-tetraoxa-4,10,16,22-tetraazacyclotetracosan-2,5,8,11,14,17,20,23-octaone (1.2). A round-bottom flask was charged with the depsipeptide (130 mg, 126 µmol), dissolved in EtOAc (1.3 mL), and treated with 10% Pd/C (2.7 mg). The reaction flask was evacuated with light vacuum. Hydrogen (balloon) was added, and the flask was cycled once more. The reaction was allowed to stir for 1 h. After purging the flask with argon, the crude reaction mixture was filtered through Celite. To the crude material was added HCl/dioxane (1.0 mL) and the reaction mixture was allowed to stir for 30 min. The reaction mixture was concentrated and added to a flame-dried round bottom flask. DCM (25.0 mL) was added and the reaction was cooled to 0 °C. Once at 0 °C, DIPEA (48.0 µL, 275 µmol) and PyBop (68.4 mg, 131 µmol) were added. The reaction was stirred at 0 °C for 1.5 h, and then allowed to warm to ambient temperature to stir for an additional 1 h. The reaction mixture was poured into cold 10% aq citric acid and extracted with DCM. The combined organic layers were washed with satd aq $NaHCO_3$, brine, and then dried and concentrated. Preparative HPLC purification (5 – 95% aqueous acetonitrile, 210 nm, flow rate: 18 mL/min, R_t = 20.2 m) afforded the 24-membered macrocycle (24.6 mg, 11%) as a light yellow oil. $[\alpha]_D^{24}$ +19 (*c* 0.27, $CHCl_3$); R_f = 0.42 (60% EtOAc in hexanes); IR (film) 3300, 2954, 2928, 2859, 1744, 1648, 1546, 1460, 1219, 1082 cm^{-1}; ^1H NMR (600 MHz, $CDCl_3$) This compound exists in multiple conformations, causing significant peak overlap. Refer to image of the ^1H NMR spectrum; ^{13}C NMR (150 MHz, $CDCl_3$) This compound exists in multiple conformations, causing significant peak overlap. Refer to image of the ^{13}C NMR spectrum; HRMS (EI): Exact mass calcd for $C_{42}H_{72}N_4O_{12}Na$ $[M+Na]^+$ 847.5045, found 847.5046. MPT-2-169

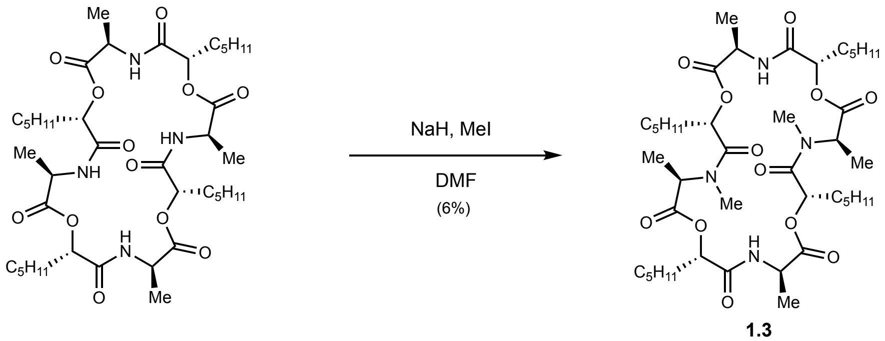

(3*R*,6*S*,9*R*,12*S*,15*R*,18*S*,21*R*,24*S*)-3,4,9,15,16,21-hexamethyl-6,12,18,24-tetrapentyl-1,7,13,19-tetraoxa-4,10,16,22-tetraazacyclotetracosan-2,5,8,11,14,17,20,23-octaone (1.3). A flame-dried round-bottom flask was charged with N-H depsipeptide (30.0 mg, 37.6 µmol) and dry DMF (527 µL) at 0 °C. Methyl iodide (94 µL, 1.51 mmol) was then added to the reaction mixture, and NaH (9.0 mg, 380 µmol in DMF (225 µL)) was added in 3 aliquots of 75 µL over 15 m. The reaction was allowed to stir at 0 °C for 20 m, and it was then quenched by the dropwise addition of satd aq NH_4Cl. The aqueous layer was extracted with EtOAc. The combined organic layers were washed with satd aq $NaHCO_3$, satd aq $Na_2S_2O_3$, water, brine, dried, concentrated. The residue was subjected to flash column chromatography (SiO_2, 1-10% methanol in dichloromethane) to afford 2 mg (6%) of the desired macrocycle as a colorless foam, as well as 9.3 mg (31%) of *ent*-verticilide. $[\alpha]_D^{24}$ +11 (*c* 0.06, $CHCl_3$); R_f = 0.40 (4% MeOH/DCM); IR (film) 3298, 2926, 2856, 1739, 1644, 1556, 1455, 1261, 1092 cm^{-1}; ^1H NMR (600 MHz, $CDCl_3$) This compound exists in multiple conformations, causing significant peak overlap. Refer to image of the 1H NMR spectrum; ^{13}C NMR (150 MHz, $CDCl_3$) This compound exists in multiple conformations, causing significant peak overlap. Refer to image of the ^{13}C NMR spectrum; HRMS (EI): Exact mass calcd for $C_{42}H_{76}N_5O_{12}$ $[M+NH_4]^+$ 842.5490, found [842.5482]. MPT-5-040

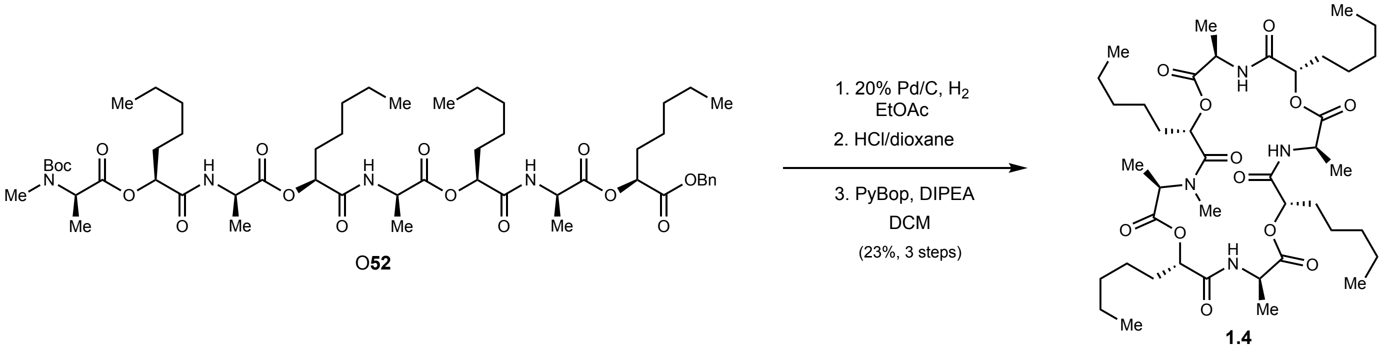

(3*R*,6*S*,9*R*,12*S*,15*R*,18*S*,21*R*,24*S*)-3,4,9,15,21-pentamethyl-6,12,18,24-tetrapentyl-1,7,13,19-tetraoxa-4,10,16,22-tetraazacyclotetracosan-2,5,8,11,14,17,20,23-octaone (1.4). A round-bottom flask was charged with the depsipeptide (200 mg, 246 µmol), dissolved in EtOAc (2.5 mL), and treated with 10% Pd/C (4.1 mg). The reaction flask was evacuated with light vacuum. Hydrogen (balloon) was added, and the flask was cycled once more. The reaction was allowed to stir for 1 h. After purging the flask with argon, the crude reaction mixture was filtered through Celite. To the crude material was added HCl/dioxane (1.0 mL) and the reaction mixture was allowed to stir for 30 min. The reaction mixture was concentrated and added to a flame-dried round bottom flask. DCM (38.5 mL) was added and the reaction was cooled to

0 °C. Once at 0 °C, DIPEA (73.8 µL, 422 µmol) and PyBop (105 mg, 201 µmol) were added. The reaction was stirred at 0 °C for 1.5 h, and then allowed to warm to ambient temperature to stir for an additional 1 h. The reaction mixture was poured into cold 10% aq citric acid and extracted with DCM. The combined organic layers were washed with satd aq $NaHCO_3$, brine, and then dried and concentrated. Preparative HPLC purification (5 – 95% aqueous acetonitrile, 210 nm, flow rate: 18 mL/min, R_t = 20.2 m) afforded the 24-membered macrocycle (29.0 mg, 23%) as a white amorphous solid $[\alpha]_D^{24}$ +25 (*c* 0.98, $CHCl_3$); R_f = 0.33 (60% EtOAc in hexanes); IR (film) 3284, 2929, 2860, 2359, 1744, 1649, 1555, 1456, 1377, 1210 cm^{-1}; ^1H NMR (600 MHz, $CDCl_3$) δ 7.70 (br d, 1H), 7.17 (br m, 2H), 5.09 (br m, 2H), 5.00 (dd, *J* = 8.9, 6.0 Hz, 1H), 4.88 (dd, *J* = 5.8, 5.8 Hz, 1H), 4.61 (br m, 2H), 4.06 (br s, 1H), 3.80 (br s, 1H), 3.17 (s, 3H), 2.02-1.93 (m, 2H), 1.88-1.60 (series of m, 8H), 1.52-1.46 (series of m, 8H), 1.42 (br d, *J* = 6.5 Hz, 4H), 1.34-1.24 (series of m, 22H), 0.93-0.86 (series of m, 12H); ^{13}C NMR (150 MHz, $CDCl_3$) ppm 171.9, 171.7, 170.7, 170.5, 170.4, 170.0, 169.7, 169.2, 74.4, 74.3, 58.9, 49.2, 48.0, 47.7, 35.7, 31.7, 31.6, 31.5, 31.4, 31.35, 31.33, 31.25, 31.18, 31.0, 30.3, 29.7, 29.6, 25.3, 25.1, 25.0, 24.4, 22.6, 22.5, 22.4, 17.7, 16.7, 16.2, 14.0, 13.9, 13.9, 13.5; HRMS (EI): Exact mass calcd for $C_{41}H_{74}N_5O_{12}$ $[M+NH_4]^+$ 828.5334, found [828.5316]. MPT-3-237

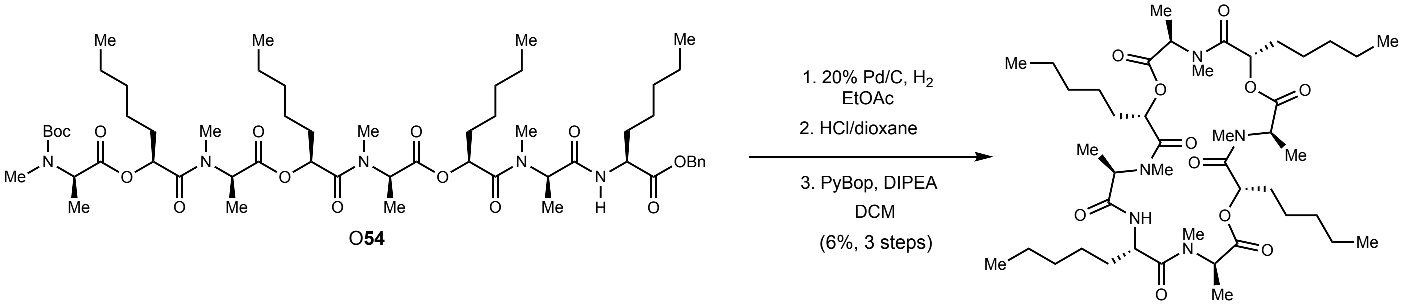

2.1

(3*R*,6*S*,9*R*,12*S*,15*R*,18*S*,21*R*,24*S*)-3,4,9,10,15,16,21,22-octamethyl-6,12,18,24-tetrapentyl-1,7,13-trioxa-4,10,16,19,22-pentaazacyclotetracosan-2,5,8,11,14,17,20,23-octaone (2.1). A round-bottom flask was charged with the depsipeptide (100 mg, 94.4 µmol), dissolved in EtOAc (944 µL), and treated with 10% Pd/C (20.0 mg). The reaction flask was evacuated with light vacuum. Hydrogen (balloon) was added, and the flask was cycled once more. The reaction was allowed to stir for 1 h. After purging the flask with argon, the crude reaction mixture was filtered through Celite. To the crude material was added 4M HCl/dioxane (1.0 mL) and the reaction mixture was allowed to stir for 30 min. The reaction mixture was concentrated and added to a flame-dried round bottom flask. DCM (18.9 mL) was added and the reaction was cooled to 0 °C. Once at 0 °C, DIPEA (49.3 µL, 283 µmol) and PyBop (51.8 mg, 99.1 µmol) were added. The reaction was stirred at 0 °C for 1.5 h, and then allowed to warm to ambient temperature to stir for an additional 1 h. The reaction mixture was poured into cold 10% aq citric acid and extracted with DCM. The combined organic layers were washed with satd aq $NaHCO_3$, brine, and then dried and concentrated. Preparative HPLC purification (5 – 95% aqueous acetonitrile, 210 nm, flow rate: 18 mL/min, R_t = 20.5 m) afforded the 24-membered macrocycle (6.24 mg, 6.0%) as a light yellow oil. $[\alpha]_D^{24}$ – 6.2 (*c* 0.29, $CHCl_3$); R_f = 0.50 (30% EtOAc in hexanes); IR (film) 3381, 2955, 2925, 2854, 1744, 1660, 1540, 1260, 1093 cm^{-1}; ^1H NMR (600 MHz, $CDCl_3$) This compound exists in multiple conformations, causing significant peak overlap. Refer to image of the ^1H NMR spectrum; ^{13}C NMR (150 MHz, $CDCl_3$) This compound exists in multiple conformations, causing significant peak overlap. Refer to image of the ^{13}C NMR spectrum; HRMS (EI): Exact mass calcd for $C_{44}H_{78}N_5O_{11}$ $[M+H]^+$ 852.5698, found 852.6592. MPT-2-201

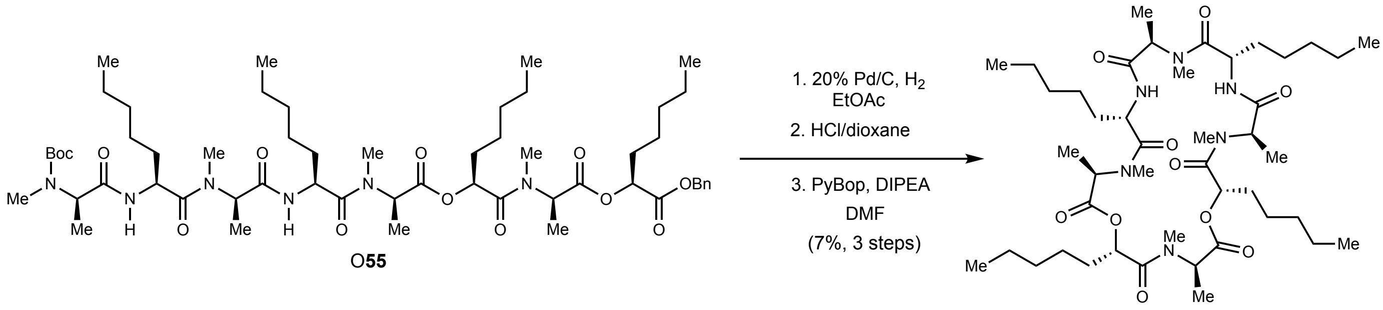

(3*R*,6*S*,9*R*,12*S*,15*R*,18*S*,21*R*,24*S*)-3,4,9,10,15,16,21,22-octamethyl-6,12,18,24-tetrapentyl-1,7,13-trioxa-4,10,16,19,22-pentaazacyclotetracosan-2,5,8,11,14,17,20,23-octaone (2.2). A round-bottom flask was charged with the depsipeptide (150 mg, 142 µmol), dissolved in EtOAc (1.9 mL), and treated with 10% Pd/C (30 mg). The reaction flask was evacuated with light vacuum. Hydrogen (balloon) was added, and the flask was cycled once more. The reaction was allowed to stir for 1 h. After purging the flask with argon, the crude reaction mixture was filtered through Celite. To the crude material was added 4M HCl/dioxane (1.0 mL) and the reaction mixture was allowed to stir for 30 min. The reaction mixture was concentrated and added to a flame-dried round bottom flask. DMF (24.0 mL) was added and the reaction was cooled to 0 °C. Once at 0 °C, DIPEA (66.0 µL, 426 µmol) and PyBop (111 mg, 213 µmol) were added. The reaction was stirred at 0 °C for 1.5 h, and then allowed to warm to ambient temperature to stir for an additional 1 h. The reaction mixture was poured into cold 10% aq citric acid and extracted with EtOAc. The combined organic layers were washed with satd aq $NaHCO_3$, brine, and then dried and concentrated. Preparative HPLC purification (5 – 95% aqueous acetonitrile, 210 nm, flow rate: 18 mL/min, R_t = 24.2 m) afforded the 24-membered macrocycle (8.43 mg, 7.0%) as a light yellow oil. $[\alpha]_D^{24}$ - 13 (*c* 0.15, $CHCl_3$); R_f = 0.50 (50% EtOAc in hexanes); IR (film) 3443, 2959, 2920, 2851, 1650, 1633, 1556, 1455 cm^{-1}; ^1H NMR (600 MHz, $CDCl_3$) This compound exists in multiple conformations, causing significant peak overlap. Refer to image of the ^1H NMR spectrum; ^{13}C NMR (150 MHz, $CDCl_3$) This compound exists in multiple conformations, causing significant peak overlap. Refer to image of the ^{13}C NMR spectrum; HRMS (EI): Exact mass calcd for $C_{44}H_{79}N_6O_{10}$ $[M+H]^+$ 851.5858, found 851.5853. MPT-2-293

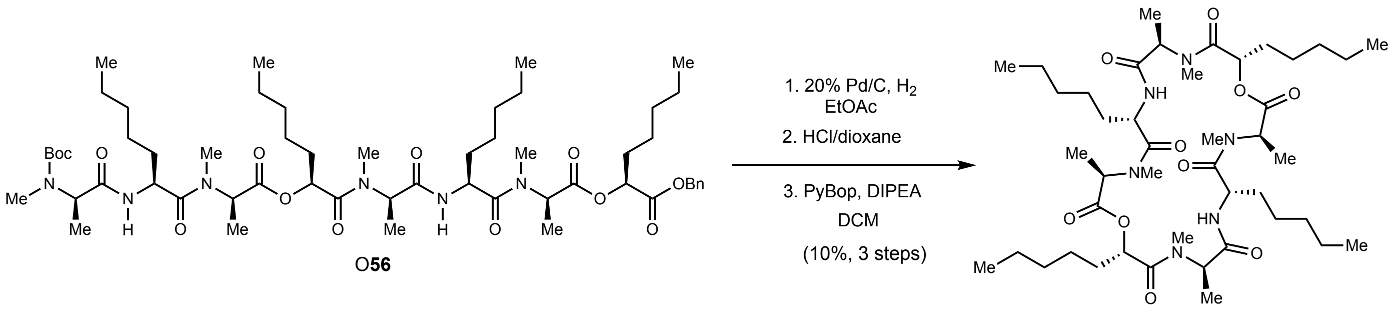

(3*R*,6*S*,9*R*,12*S*,15*R*,18*S*,21*R*,24*S*)-3,4,9,10,15,16,21,22-octamethyl-6,12,18,24-tetrapentyl-1,13-dioxa-4,7,10,16,19,22-hexaazacyclotetracosan-2,5,8,11,14,17,20,23-octaone (2.3). A round-bottom flask was charged with the depsipeptide (97.0 mg, 92.0 µmol), dissolved in EtOAc (2.0 mL), and treated with 10% Pd/C (2.0 mg). The reaction flask was evacuated with light vacuum. Hydrogen (balloon) was added, and the flask was cycled once more. The reaction was allowed to stir for 1 h. After purging the flask with argon, the crude reaction mixture was filtered through Celite. To the crude material was added 4M HCl/dioxane (1.0 mL) and the reaction mixture was allowed to stir for 30 min. The reaction mixture was concentrated and added to a flame-dried round bottom flask. DCM (18.0 mL) was added and the reaction was cooled to 0 °C. Once at 0 °C, DIPEA (35.0 µL, 200 µmol) and PyBop (50.0 mg, 97.0 µmol) were added. The reaction was stirred at 0 °C for 1.5 h, and then allowed to warm to ambient temperature to stir for an additional 1 h. The reaction mixture was poured into cold 10% aq citric acid and extracted with DCM. The combined organic layers were washed with satd aq $NaHCO_3$, brine, and then dried and concentrated. Preparative HPLC purification (5 – 95% aqueous acetonitrile, 210 nm, flow rate: 18 mL/min, R_t = 22.2 m) afforded the 24-membered macrocycle (7.8 mg, 10.0%) as a light yellow oil. $[\alpha]_D^{24}$ - 16 (*c* 0.23, $CHCl_3$); R_f = 0.43 (50% EtOAc in hexanes); IR (film) 3478, 2922, 2851, 1740, 1633, 1455, 1260 cm^{-1}; ^1H NMR (600 MHz, $CDCl_3$) This compound exists in multiple conformations, causing significant peak overlap. Refer to image of the ^1H NMR spectrum; ^{13}C NMR (150 MHz, $CDCl_3$) This compound exists in multiple conformations, causing significant peak overlap. Refer to image of the ^{13}C NMR spectrum; HRMS (EI): Exact mass calcd for $C_{44}H_{82}N_7O_{10}$ $[M+NH_4]^+$ 868.6123, found 868.6119. MPT-2-267

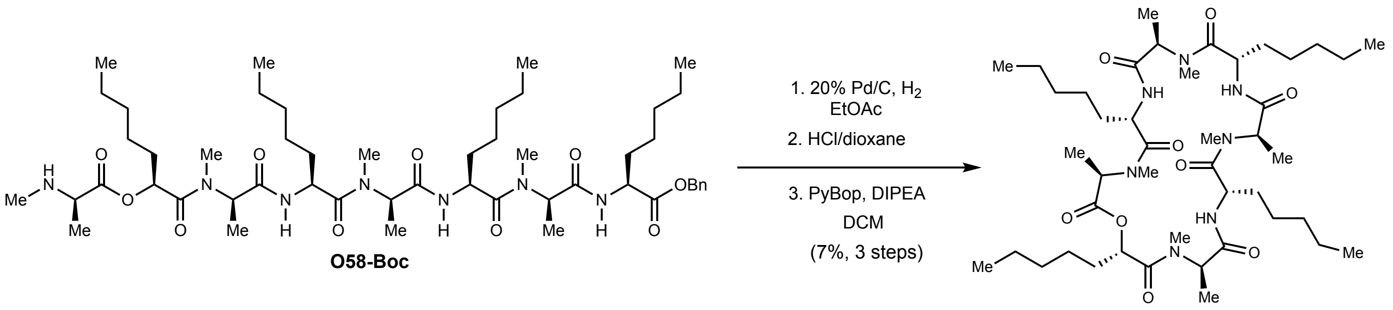

(3*R*,6*S*,9*R*,12*S*,15*R*,18*S*,21*R*,24*S*)-3,4,9,10,15,16,21,22-octamethyl-6,12,18,24-tetrapentyl-1-oxa-4,7,10,13,16,19,22-heptaazacyclotetracosan-2,5,8,11,14,17,20,23-octaone (2.4). A round-bottom flask was charged with the depsipeptide (204 mg, 193 µmol), dissolved in EtOAc (1.9 mL), and treated with 10% Pd/C (4.90 mg). The reaction flask was evacuated with light vacuum. Hydrogen (balloon) was added, and the flask was cycled once more. The reaction was allowed to stir for 1 h. After purging the flask with argon, the crude reaction mixture was filtered through Celite. To the crude material

was added HCl/dioxane (1.0 mL) and the reaction mixture was allowed to stir for 30 min. The reaction mixture was concentrated and added to a flame-dried round bottom flask. DCM (38.6 mL) was added and the reaction was cooled to 0 °C. Once at 0 °C, DIPEA (73.9 µL, 424 µmol) and PyBop (105 mg, 202 µmol) were added. The reaction was stirred at 0 °C for 1.5 h, and then allowed to warm to ambient temperature to stir for an additional 1 h. The reaction mixture was poured into cold 10% aq citric acid and extracted with DCM. The combined organic layers were washed with satd aq $NaHCO_3$, brine, and then dried and concentrated. Preparative HPLC purification (5 – 95% aqueous acetonitrile, 210 nm, flow rate: 18 mL/min, R_t = 24.2 m) afforded the 24-membered macrocycle (12.0 mg, 7.3%) as a yellow oil. $[\alpha]_D^{24}$ + 6.0 (*c* 0.14, $CHCl_3$); R_f = 0.41 (60% EtOAc in hexanes); IR (film) 3389, 2952, 2851, 1743, 1633, 1455, 1165cm^{-1}; ^{1}H NMR (600 MHz, $CDCl_3$) This compound exists in multiple conformations, causing significant peak overlap. Refer to image of the ^{1}H NMR spectrum; ^{13}C NMR (150 MHz, $CDCl_3$) This compound exists in multiple conformations, causing significant peak overlap. Refer to image of the ^{13}C NMR spectrum; HRMS (EI): Exact mass calcd for $C_{44}H_{83}N_8O_9$ $[M+NH_4]^+$ 867.6283, found 867.6283. MPT-3-015

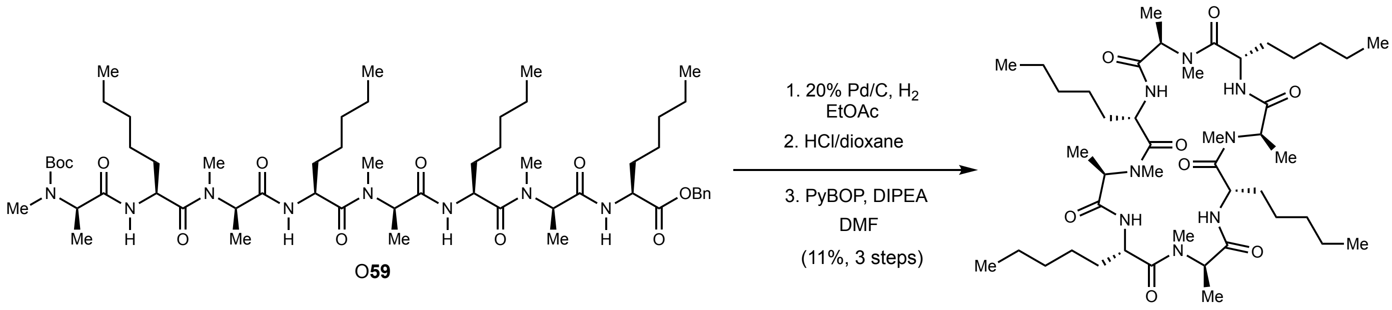

(3*S*,6*R*,9*S*,12*R*,15*S*,18*R*,21*S*,24*R*)-1,6,7,12,13,18,19,24-octamethyl-3,9,15,21-tetrapentyl-1,4,7,10,13,16,19,22-octaazacyclotetracosan-2,5,8,11,14,17,20,23-octaone (2.5). A round-bottom flask was charged with the depsipeptide (116 mg, 110 µmol), dissolved in EtOAc (1.1 mL), and treated with 10% Pd/C (2.4 mg). The reaction flask was evacuated with light vacuum. Hydrogen (balloon) was added, and the flask was cycled once more. The reaction was allowed to stir for 1 h. After purging the flask with argon, the crude reaction mixture was filtered through Celite. To the crude material was added 4M HCl/dioxane (1.00 mL) and the reaction mixture was allowed to stir for 30 min. The reaction mixture was concentrated and added to a flame-dried round bottom flask. DMF (24.0 mL) was added and the reaction was cooled to 0 °C. Once at 0 °C, DIPEA (43.4 µL, 330 µmol) and PyBop (60.0 mg, 115 µmol) were added. The reaction was stirred at 0 °C for 1.5 h, and then allowed to warm to ambient temperature to stir for an additional 1 h. The reaction mixture was poured into cold 10% aq citric acid and extracted with EtOAc. The combined organic layers were washed with satd aq $NaHCO_3$, brine, and then dried and concentrated. Preparative HPLC purification (5 – 95% aqueous acetonitrile, 210 nm, flow rate: 18 mL/min, R_t = 23.1 m) afforded the 24-membered macrocycle (10.2 mg, 11.0%) as a light yellow oil. $[\alpha]_D^{24}$ - 11 (*c* 0.29, $CHCl_3$); R_f = 0.33 (50% EtOAc in hexanes); IR (film) 3292, 2929, 1654, 1637, 1560, 1458 cm^{-1}; ^{1}H NMR (600 MHz, $CDCl_3$) This compound exists in multiple conformations, causing significant peak overlap. Refer to image of the ^{1}H NMR spectrum; ^{13}C NMR (150 MHz, $CDCl_3$) This compound exists in multiple conformations, causing significant peak overlap. Refer to image of the ^{13}C NMR spectrum; HRMS (EI): Exact mass calcd for $C_{44}H_{84}N_9O_8$ $[M+NH_4]^+$ 866.6443, found 866.6440. MPT-2-290

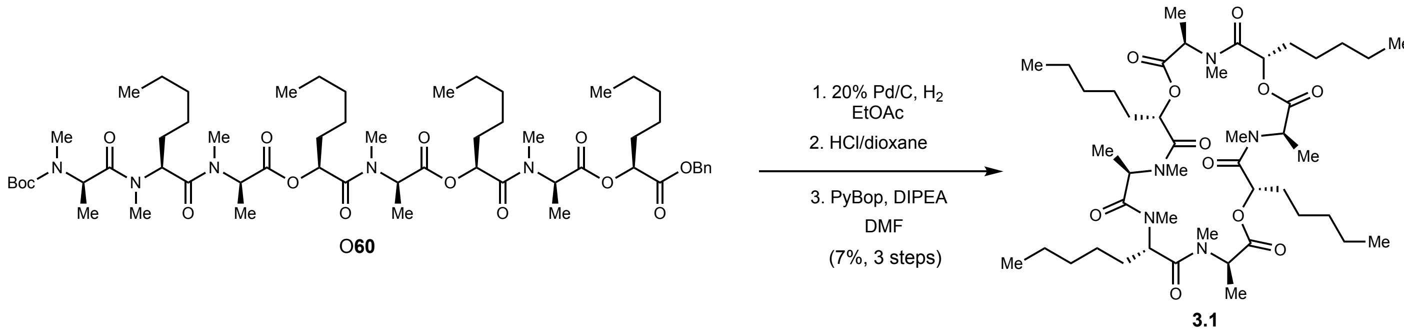

(3*R*,6*S*,9*R*,12*S*,15*R*,18*S*,21*R*,24*S*)-3,4,9,10,15,16,19,21,22-nonamethyl-6,12,18,24-tetrapentyl-1,7,13-trioxa-4,10,16,19,22-pentaazacyclotetracosan-2,5,8,11,14,17,20,23-octaone (3.1). A round-bottom flask was charged with the depsipeptide (189 mg, 176 µmol), dissolved in EtOAc (1.8 mL), and treated with 10% Pd/C (37.8 mg). The reaction flask was evacuated with light vacuum. Hydrogen (balloon) was added, and the flask was cycled once more. The reaction was allowed to stir for 1 h. After purging the flask with argon, the crude reaction mixture was filtered through Celite. To the crude material was added 4M HCl/dioxane (1.00 mL) and the reaction mixture was allowed to stir for 30 min. The reaction mixture was concentrated and added to a flame-dried round bottom flask. DMF (49.5mL) was added and the reaction was cooled to 0 °C. Once at 0 °C, DIPEA (49.5 µL, 284 µmol) and PyBop (70.6 mg, 129 µmol) were added. The reaction was stirred at 0 °C for 1.5 h, and then allowed to warm to ambient temperature to stir for an additional 1 h. The reaction mixture was poured into cold 10% aq citric acid and extracted with EtOAc. The combined organic layers were washed with satd aq $NaHCO_3$, brine, and then dried and concentrated. Preparative HPLC purification (5 – 95% aqueous acetonitrile, 210 nm, flow rate: 18 mL/min, R_t = 23.0 m) afforded the 24-membered macrocycle (7.6 mg, 5.1%) as a yellow oil. $[\alpha]_D^{24}$ + 5.0 (*c* 0.19, $CHCl_3$); R_f = 0.48 (50% EtOAcEtOAc in hexanes); IR (film) 2930, 2861, 1747, 1715, 1669, 1539, 1157 cm^{-1}; ^1H NMR (600 MHz, $CDCl_3$) This compound exists in multiple conformations, causing significant peak overlap. Refer to image of the ^1H NMR spectrum; ^{13}C NMR (150 MHz, $CDCl_3$) This compound exists in multiple conformations, causing significant peak overlap. Refer to image of the ^{13}C NMR spectrum; HRMS (EI): Exact mass calcd for $C_{45}H_{83}N_6O_{11}$ $[M+NH_4]^+$ 883.6120, found 883.6122. MPT-3-078

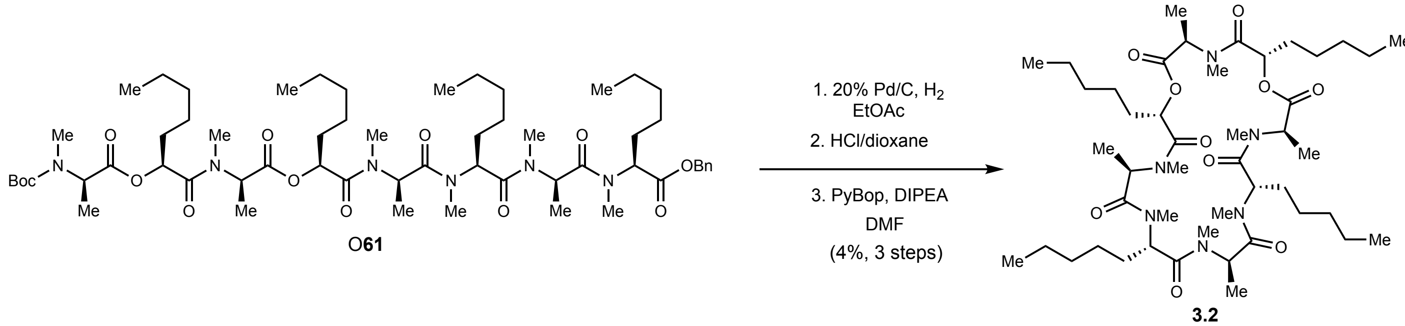

(3*R*,6*S*,9*R*,12*S*,15*R*,18*S*,21*R*,24*S*)-3,4,9,10,13,15,16,19,21,22-decamethyl-6,12,18,24-tetrapentyl-1,7-dioxa-4,10,13,16,19,22-hexaazacyclotetracosan-2,5,8,11,14,17,20,23-octaone (3.2). A round-bottom flask was charged with the depsipeptide (149 mg, 138 µmol), dissolved in EtOAc (1.4 mL), and treated with 10% Pd/C (29.8 mg). The reaction flask was evacuated with light vacuum. Hydrogen (balloon) was added, and the flask was cycled once more. The reaction was allowed to stir for 1 h. After purging the flask with argon, the crude reaction mixture was filtered through Celite. To the crude material was added 4M HCl/dioxane (1.0 mL) and the reaction mixture was allowed to stir for 30 min. The reaction

mixture was concentrated and added to a flame-dried round bottom flask. DMF (48.0 mL) was added and the reaction was cooled to 0 °C. Once at 0 °C, DIPEA (55.0 µL, 414 µmol) and PyBop (76.0 mg, 150 µmol) were added. The reaction was stirred at 0 °C for 1.5 h, and then allowed to warm to ambient temperature to stir for an additional 1 h. The reaction mixture was poured into cold 10% aq citric acid and extracted with EtOAc. The combined organic layers were washed with satd aq $NaHCO_3$, brine, and then dried and concentrated. Preparative HPLC purification (5 – 95% aqueous acetonitrile, 210 nm, flow rate: 18 mL/min, R_t = 23.0 m) afforded the 24-membered macrocycle (4.80 mg, 4.0%) as a yellow oil. $[\alpha]_D^{24}$ - 13 (*c* 0.15, $CHCl_3$); R_f = 0.40 (60% EtOAc in hexanes); IR (film) 2930, 2850, 1744, 1698, 1456, 1156 cm^{-1}; ^1H NMR (600 MHz, $CDCl_3$) This compound exists in multiple conformations, causing significant peak overlap. Refer to image of the ^1H NMR spectrum; ^{13}C NMR (150 MHz, $CDCl_3$) This compound exists in multiple conformations, causing significant peak overlap. Refer to image of the ^{13}C NMR spectrum; HRMS (EI): Exact mass calcd for $C_{46}H_{86}N_7O_{10}$ $[M+NH_4]^+$ 896.6436, found 896.6433. MPT-4-037

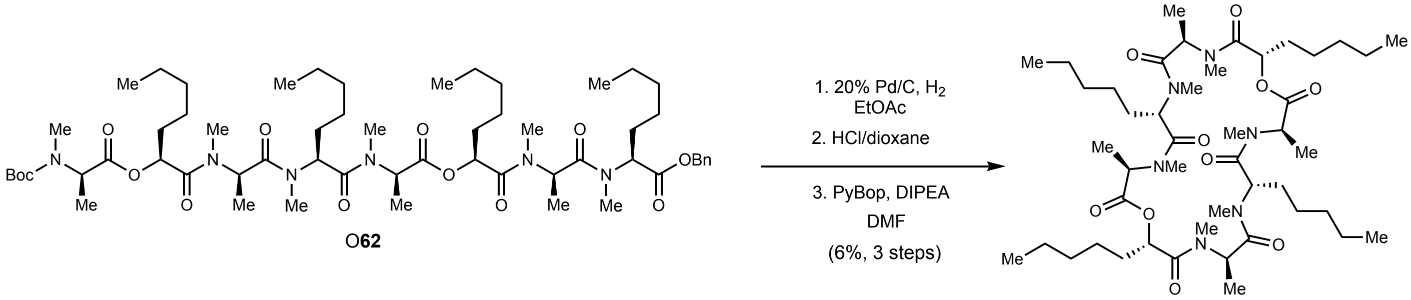

(3*R*,6*S*,9*R*,12*S*,15*R*,18*S*,21*R*,24*S*)-3,4,7,9,10,15,16,19,21,22-decamethyl-6,12,18,24-tetrapentyl-1,13-dioxa-4,7,10,16,19,22-hexaazacyclotetracosan-2,5,8,11,14,17,20,23-octaone (3.3). A round-bottom flask was charged with the depsipeptide (154 mg, 142 µmol), dissolved in EtOAc (1.4 mL), and treated with 10% Pd/C (30.8 mg). The reaction flask was evacuated with light vacuum. Hydrogen (balloon) was added, and the flask was cycled once more. The reaction was allowed to stir for 1 h. After purging the flask with argon, the crude reaction mixture was filtered through Celite. To the crude material was added 4M HCl/dioxane (1.00 mL) and the reaction mixture was allowed to stir for 30 min. The reaction mixture was concentrated and added to a flame-dried round bottom flask. DMF (28.4 mL) was added and the reaction was cooled to 0 °C. Once at 0 °C, DIPEA (49.5 µL, 284 µmol) and PyBop (70.6 mg, 136 µmol) were added. The reaction was stirred at 0 °C for 1.5 h, then allowed to warm to ambient temperature and stir for an additional 1 h. The reaction mixture was poured into cold 10% aq citric acid and extracted with EtOAc. The combined organic layers were washed with satd aq $NaHCO_3$, brine, and then dried and concentrated. Preparative HPLC purification (5 – 95% aqueous acetonitrile, 210 nm, flow rate: 18 mL/min, R_t = 23.0 m) afforded the 24-membered macrocycle (7.0 mg, 6.1%) as a yellow oil. $[\alpha]_D^{24}$ - 23 (*c* 0.22, $CHCl_3$); R_f = 0.44 (50% EtOAc in hexanes); IR (film) 2932, 2871, 1745, 1667, 1456, 1190 cm^{-1}; ^1H NMR (600 MHz, $CDCl_3$) This compound exists in multiple conformations, causing significant peak overlap. Refer to image of the ^1H NMR spectrum; ^{13}C NMR (150 MHz, $CDCl_3$) This compound exists in multiple conformations, causing significant peak overlap. Refer to image of the ^{13}C NMR spectrum; HRMS (EI): Exact mass calcd for $C_{46}H_{86}N_7O_{10}$ $[M+NH_4]^+$ 896.6436, found 896.6435. MPT-3-035

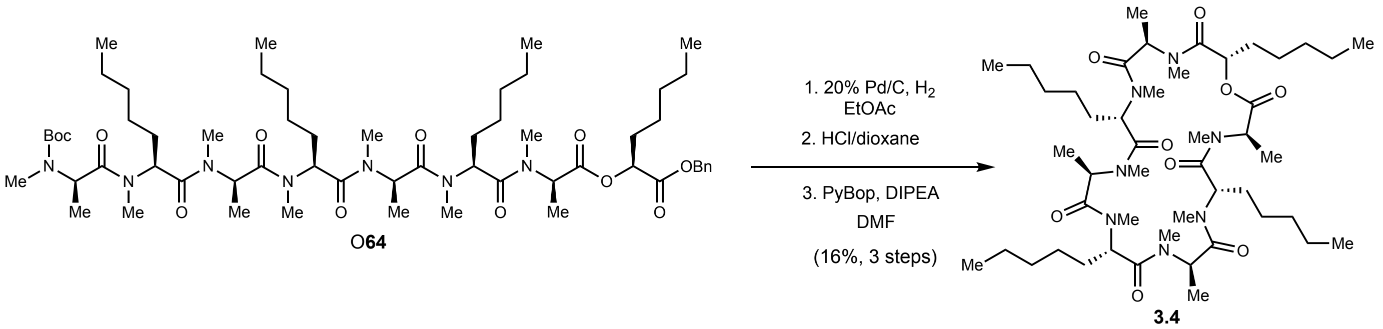

(3*R*,6*S*,9*R*,12*S*,15*R*,18*S*,21*R*,24*S*)-3,4,7,9,10,13,15,16,19,21,22-undecamethyl-6,12,18,24-tetrapentyl-1-oxa-4,7,10,13,16,19,22-heptaazacyclotetracosan-2,5,8,11,14,17,20,23-octaone (3.4). A round-bottom flask was charged with the depsipeptide (30 mg, 27 µmol), dissolved in EtOAc (270 µL), and treated with 10% Pd/C (6.0 mg). The reaction flask was evacuated with light vacuum. Hydrogen (balloon) was added, and the flask was cycled once more. The reaction was allowed to stir for 1 h. After purging the flask with argon, the crude reaction mixture was filtered through Celite. To the crude material was added 4M HCl/dioxane (1.00 mL) and the reaction mixture was allowed to stir for 30 min. The reaction mixture was concentrated and added to a flame-dried round bottom flask. DMF (5.4 mL) was added and the reaction was cooled to 0 °C. Once at 0 °C, DIPEA (15 µL, 81 µmol) and PyBop (15 mg, 28 µmol) were added. The reaction was stirred at 0 °C for 1.5 h, and then allowed to warm to ambient temperature to stir for an additional 1 h. The reaction mixture was poured into cold 10% aq citric acid and extracted with EtOAc. The combined organic layers were washed with satd aq $NaHCO_3$, brine, and then dried and concentrated. Preparative HPLC purification (5 – 95% aqueous acetonitrile, 210 nm, flow rate: 18 mL/min, R_t = 23.1 m) afforded the 24-membered macrocycle (2.9 mg, 16%) as a yellow oil. $[\alpha]_D^{24}$ + 4.0 (*c* 0.11, $CHCl_3$); R_f = 0.40 (60% EtOAc in hexanes); IR (film) 2931, 2860, 1743, 1663, 1456, 1185 cm^{-1}; ^1H NMR (600 MHz, $CDCl_3$) This compound exists in multiple conformations, causing significant peak overlap. Refer to image of the ^1H NMR spectrum; ^{13}C NMR (150 MHz, $CDCl_3$) This compound exists in multiple conformations, causing significant peak overlap. Refer to image of the ^{13}C NMR spectrum; HRMS (EI): Exact mass calcd for $C_{47}H_{89}N_8O_9$ $[M+NH_4]^+$ 909.6753, found 909.6748. MPT-5-181

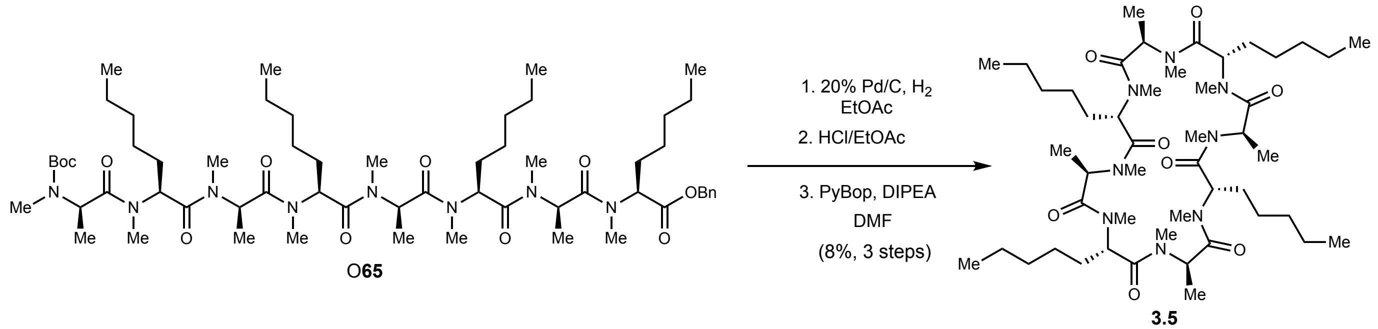

(3*R*,6*S*,9*R*,12*S*,15*R*,18*S*,21*R*,24*S*)-1,3,4,7,9,10,13,15,16,19,21,22-dodecamethyl-6,12,18,24-tetrapentyl-1,4,7,10,13,16,19,22-octaazacyclotetracosan-2,5,8,11,14,17,20,23-octaone (3.5). A round-bottom flask was charged with the depsipeptide (74.1 mg, 66.6 µmol), dissolved in EtOAc (666 µL), and treated with 10% Pd/C (14.8 mg). The reaction flask was evacuated with light vacuum. Hydrogen (balloon) was added, and the flask was cycled once more. The reaction was allowed to stir for 1 h. After purging the flask with argon, the crude reaction mixture was filtered through Celite. To

the crude material was added 4M HCl/EtOAc (1.0 mL) and the reaction mixture was allowed to stir for 30 min. The reaction mixture was concentrated and added to a flame-dried round bottom flask. DMF (13.3 mL) was added and the reaction was cooled to 0 °C. Once at 0 °C, DIPEA (26.3 µL, 200 µmol) and PyBop (36.5 mg, 69.9 µmol) were added. The reaction was stirred at 0 °C for 1.5 h, and then allowed to warm to ambient temperature to stir for an additional 1 h. The reaction mixture was poured into cold 10% aq citric acid and extracted with EtOAc. The combined organic layers were washed with satd aq $NaHCO_3$, brine, and then dried and concentrated. Preparative HPLC purification (5 – 95% aqueous acetonitrile, 210 nm, flow rate: 18 mL/min, R_t = 24.7 m) afforded the 24-membered macrocycle (5.5 mg, 8.3%) as a yellow oil. $[\alpha]_D^{24}$ + 4.9 (*c* 0.21, $CHCl_3$); R_f = 0.31 (40% EtOAc in hexanes); IR (film) 2927, 2855, 1747, 1653, 1457, 1196 cm^{-1}; ^1H NMR (600 MHz, $CDCl_3$) This compound exists in multiple conformations, causing significant peak overlap. Refer to image of the ^1H NMR spectrum; ^{13}C NMR (150 MHz, $CDCl_3$) This compound exists in multiple conformations, causing significant peak overlap. Refer to image of the ^{13}C NMR spectrum; HRMS (EI): Exact mass calcd for $C_{48}H_{89}N_8O_8$ $[M+H]^+$ 905.6803, found 905.6800. MPT-5-242

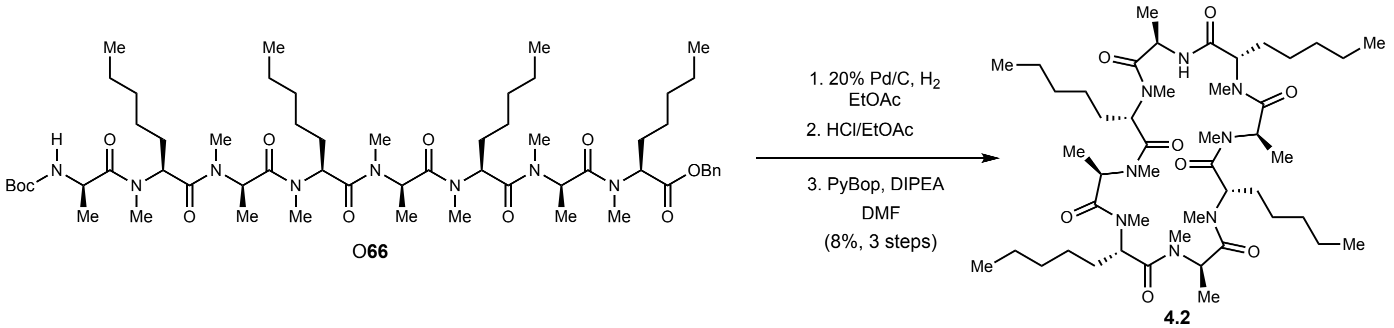

(3*R*,6*S*,9*R*,12*S*,15*R*,18*S*,21*R*,24*S*)-1,3,4,7,9,10,13,15,16,19,21-undecamethyl-6,12,18,24-tetrapentyl-1,4,7,10,13,16,19,22-octaazacyclotetracosan-2,5,8,11,14,17,20,23-octaone (4.2). A round-bottom flask was charged with the depsipeptide (116 mg, 110 µmol), dissolved in EtOAc (1.1 mL), and treated with 10% Pd/C (23.2 mg). The reaction flask was evacuated with light vacuum. Hydrogen (balloon) was added, and the flask was cycled once more. The reaction was allowed to stir for 1 h. After purging the flask with argon, the crude reaction mixture was filtered through Celite. To the crude material was added 4M HCl/EtOAc (1.00 mL) and the reaction mixture was allowed to stir for 30 min. The reaction mixture was concentrated and added to a flame-dried round bottom flask. Dry DMF (24.0 mL) was added and the reaction was cooled to 0 °C. Once at 0 °C, DIPEA (43.4 µL, 330 µmol) and PyBop (60.0 mg, 115 µmol) were added. The reaction was stirred at 0 °C for 1.5 h, and then allowed to warm to ambient temperature to stir for an additional 1 h. The reaction mixture was poured into cold 10% aq citric acid and extracted with EtOAc. The combined organic layers were washed with satd aq $NaHCO_3$, brine, and then dried and concentrated. Preparative HPLC purification (5 – 95% aqueous acetonitrile, 210 nm, flow rate: 18 mL/min, R_t = 24.1 m) afforded the 24-membered macrocycle (6.0 mg, 7.5%) as a light yellow oil. $[\alpha]_D^{24}$ – 8.1 (*c* 0.30, $CHCl_3$); R_f = 0.55 (60% EtOAcEtOAc in hexanes); IR (film) 3380, 2925, 2854, 1744, 1661, 1458, 1202, 1092 cm^{-1}; ^1H NMR (600 MHz, $CDCl_3$) This compound exists in multiple conformations, causing significant peak overlap. Refer to image of the ^1H NMR spectrum; ^{13}C NMR (150 MHz, $CDCl_3$) This compound exists in multiple conformations, causing significant peak overlap. Refer to image of the ^{13}C NMR spectrum; HRMS (EI): Exact mass calcd for $C_{47}H_{87}N_8O_8$ $[M+H]^+$ 891.6641, found 891.6640. MPT-4-224

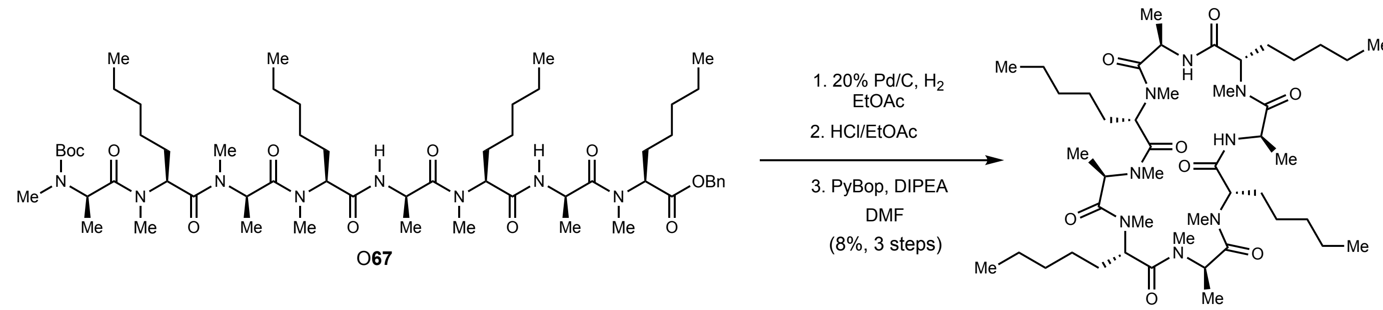

O**67** → **4.3**

(3*R*,6*S*,9*R*,12*S*,15*R*,18*S*,21*R*,24*S*)-1,3,4,7,9,10,13,15,19,21-decamethyl-6,12,18,24-tetrapentyl-1,4,7,10,13,16,19,22-octaazacyclotetracosan-2,5,8,11,14,17,20,23-octaone (4.3). A round-bottom flask was charged with the depsipeptide (64.0 mg, 59.2 µmol), dissolved in EtOAc (592 µL), and treated with 10% Pd/C (12.8 mg). The reaction flask was evacuated with light vacuum. Hydrogen (balloon) was added, and the flask was cycled once more. The reaction was allowed to stir for 1 h. After purging the flask with argon, the crude reaction mixture was filtered through Celite. To the crude material was added 4M HCl/EtOAc (1.00 mL) and the reaction mixture was allowed to stir for 30 min. The reaction mixture was concentrated and added to a flame-dried round bottom flask. Dry DMF (11.9 mL) was added and the reaction was cooled to 0 °C. Once at 0 °C, DIPEA (23.4 µL, 178 µmol) and PyBop (32.4 mg, 62.2 µmol) were added. The reaction was stirred at 0 °C for 0.5 h, and then allowed to warm to ambient temperature to stir for an additional 2.5 h. The reaction mixture was poured into cold 10% aq citric acid and extracted with EtOAc. The combined organic layers were washed with satd aq $NaHCO_3$, brine, and then dried and concentrated. Preparative HPLC purification (5 – 95% aqueous acetonitrile, 210 nm, flow rate: 18 mL/min, R_t = 22.9 m) afforded the 24-membered macrocycle (4.2 mg, 8.1%) as a light yellow oil. $[\alpha]_D^{24}$ – 3.7 (*c* 0.16, $CHCl_3$); R_f = 0.50 (60% EtOAc in hexanes); IR (film) 3319, 2926, 2857, 1745, 1692, 1456, 1155, 1028 cm^{-1}; ^{1}H NMR (600 MHz, $CDCl_3$) This compound exists in multiple conformations, causing significant peak overlap. Refer to image of the ^{1}H NMR spectrum; ^{13}C NMR (150 MHz, $CDCl_3$) This compound exists in multiple conformations, causing significant peak overlap. Refer to image of the ^{13}C NMR spectrum; HRMS (EI): Exact mass calcd for $C_{46}H_{85}N_8O_8$ [M+H]$^+$ 877.6490, found 877.6486. MPT-4-251

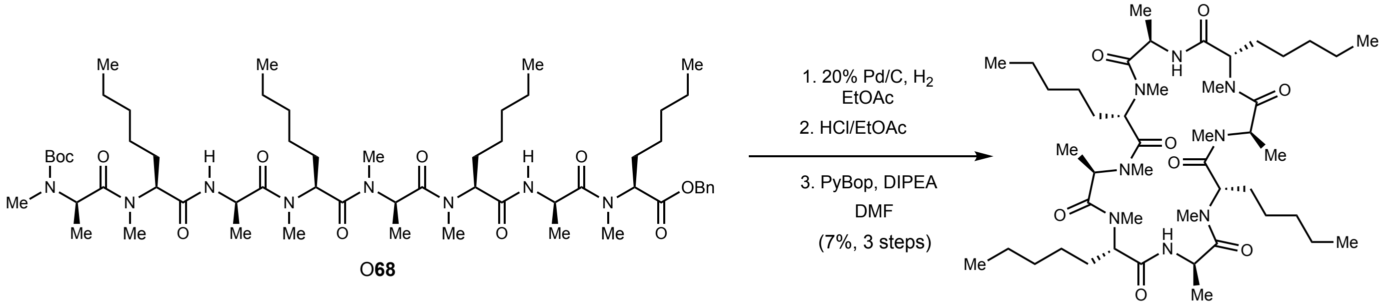

O**68** → **4.4**

(3*R*,6*S*,9*R*,12*S*,15*R*,18*S*,21*R*,24*S*)-1,3,4,7,9,10,13,15,19,21-decamethyl-6,12,18,24-tetrapentyl-1,4,7,10,13,16,19,22-octaazacyclotetracosan-2,5,8,11,14,17,20,23-octaone (4.4). A round-bottom flask was charged with the depsipeptide (102 mg, 94.0 µmol), dissolved in EtOAc (940 µL), and treated with 10% Pd/C (18.8 mg). The reaction flask was evacuated with light vacuum. Hydrogen (balloon) was added, and the flask was cycled once more. The reaction was allowed to stir

for 1 h. After purging the flask with argon, the crude reaction mixture was filtered through Celite. To the crude material was added 4M HCl/EtOAc (1.00 mL) and the reaction mixture was allowed to stir for 30 min. The reaction mixture was concentrated and added to a flame-dried round bottom flask. Dry DMF (19.0 mL) was added and the reaction was cooled to 0 °C. Once at 0 °C, DIPEA (37.3 µL, 282 µmol) and PyBop (51.6 mg, 99.1 µmol) were added. The reaction was stirred at 0 °C for 0.5 h, and then allowed to warm to ambient temperature to stir for an additional 2.5 h. The reaction mixture was poured into cold 10% aq citric acid and extracted with EtOAc. The combined organic layers were washed with satd aq $NaHCO_3$, brine, and then dried and concentrated. Preparative HPLC purification (5 – 95% aqueous acetonitrile, 210 nm, flow rate: 18 mL/min, R_t = 23.2 m) afforded the 24-membered macrocycle (5.3 mg, 6.6%) as a light yellow oil. $[\alpha]_D^{24}$ - 3.2 (*c* 0.13, $CHCl_3$); R_f = 0.34 (60% EtOAc in hexanes); IR (film) 3287, 2922, 2860, 1744, 1649, 1555, 1456, 1210 cm^{-1}; ^1H NMR (600 MHz, $CDCl_3$) This compound exists in multiple conformations, causing significant peak overlap. Refer to image of the ^1H NMR spectrum; ^{13}C NMR (150 MHz, $CDCl_3$) This compound exists in multiple conformations, causing significant peak overlap. Refer to image of the ^{13}C NMR spectrum; HRMS (EI): Exact mass calcd for $C_{46}H_{88}N_9O_8$ $[M+NH_4]^+$ 894.6756, found 894.6753. MPT-3-103

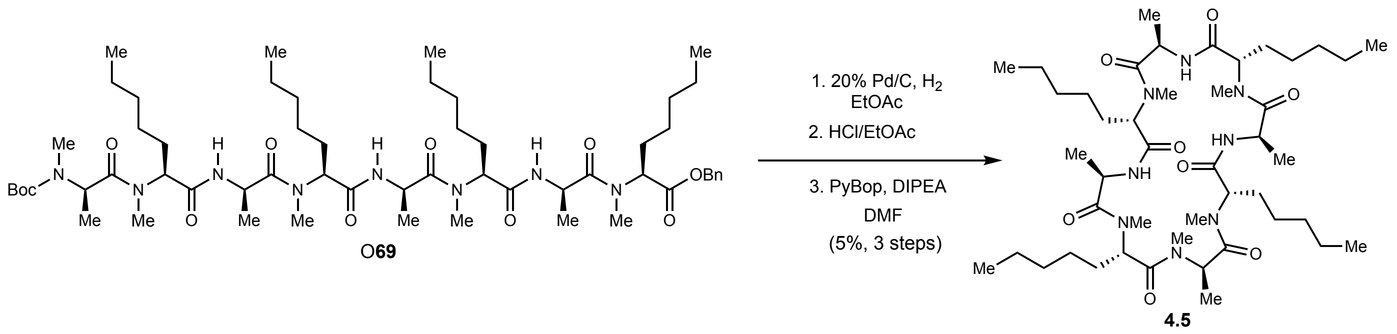

(3*R*,6*S*,9*R*,12*S*,15*R*,18*S*,21*R*,24*S*)-1,3,4,7,9,13,15,19,21-nonamethyl-6,12,18,24-tetrapentyl-1,4,7,10,13,16,19,22-octaazacyclotetracosan-2,5,8,11,14,17,20,23-octaone (4.5). A round-bottom flask was charged with the depsipeptide (76 mg, 71.0 µmol), dissolved in EtOAc (710 µL), and treated with 10% Pd/C (15.2 mg). The reaction flask was evacuated with light vacuum. Hydrogen (balloon) was added, and the flask was cycled once more. The reaction was allowed to stir for 1 h. After purging the flask with argon, the crude reaction mixture was filtered through Celite. To the crude material was added 4M HCl/dioxane (1.00 mL) and the reaction mixture was allowed to stir for 30 min. The reaction mixture was concentrated and added to a flame-dried round bottom flask. Dry DMF (14.2 mL) was added and the reaction was cooled to 0 °C. Once at 0 °C, DIPEA (9.34 µL, 215 µmol) and PyBop (13.0 mg, 74.6 µmol) were added. The reaction was stirred at 0 °C for 1.5 h, and then allowed to warm to ambient temperature to stir for an additional 1 h. The reaction mixture was poured into cold 10% aq citric acid and extracted with EtOAc. The combined organic layers were washed with satd aq $NaHCO_3$, brine, and then dried and concentrated. Preparative HPLC purification (5 – 95% aqueous acetonitrile, 210 nm, flow rate: 18 mL/min, R_t = 23.0 m) afforded the 24-membered macrocycle (3.0 mg, 4.9%) as a light yellow oil. $[\alpha]_D^{24}$ – 7.2 (*c* 0.14, $CHCl_3$); R_f = 0.18 (60% EtOAcin hexanes); IR (film) 3340, 2934, 2861, 1747, 1666, 1456, 1391, 1084 cm^{-1}; ^1H NMR (600 MHz, $CDCl_3$) This compound exists in multiple conformations, causing significant peak overlap. Refer to image of the ^1H NMR spectrum; ^{13}C NMR (150 MHz, $CDCl_3$) This compound exists in multiple conformations, causing significant peak overlap. Refer to image of the ^{13}C NMR spectrum; HRMS (EI): Exact mass calcd for $C_{45}H_{86}N_9O_8$ $[M+NH_4]^+$ 880.6599, found 880.6603. MPT-5-254

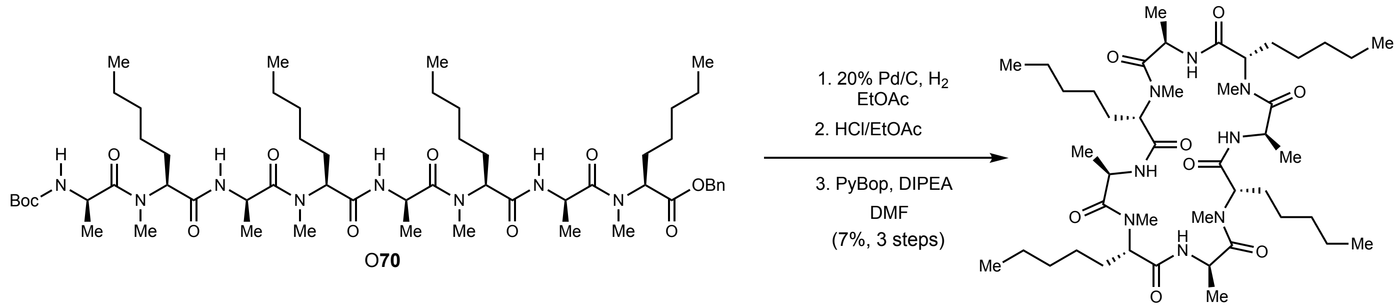

4.6

(3*R*,6*S*,9*R*,12*S*,15*R*,18*S*,21*R*,24*S*)-1,3,7,9,13,15,19,21-octamethyl-6,12,18,24-tetrapentyl-1,4,7,10,13,16,19,22-octaazacyclotetracosan-2,5,8,11,14,17,20,23-octaone (4.6). A round-bottom flask was charged with the depsipeptide (136 mg, 128 µmol), dissolved in EtOAc (1.3 mL), and treated with 10% Pd/C (27.2 mg). The reaction flask was evacuated with light vacuum. Hydrogen (balloon) was added, and the flask was cycled once more. The reaction was allowed to stir for 1 h. After purging the flask with argon, the crude reaction mixture was filtered through Celite. To the crude material was added 4M HCl/EtOAc (1.00 mL) and the reaction mixture was allowed to stir for 30 min. The reaction mixture was concentrated and added to a flame-dried round bottom flask. DMF (25.7 mL) was added and the reaction was cooled to 0 °C. Once at 0 °C, DIPEA (50.5 µL, 384 µmol) and PyBop (69.8 mg, 134 µmol) were added. The reaction was stirred at 0 °C for 0.5 h, then allowed to warm to ambient temperature and stir for an additional 2.5 h. The reaction mixture was poured into cold 10% aq citric acid and extracted with EtOAc. The combined organic layers were washed with satd aq $NaHCO_3$, brine, and then dried and concentrated. Preparative HPLC purification (5 – 95% aqueous acetonitrile, 210 nm, flow rate: 18 mL/min, R_t = 23.1 m) afforded the 24-membered macrocycle (7.6 mg, 7.0%) as a light yellow oil. $[\alpha]_D^{24}$ – 9.9 (*c* 0.23, $CHCl_3$); R_f = 0.48 (60% EtOAc in hexanes); IR (film) 3443, 2821, 2851, 1741, 1650, 1260, 1019 cm^{-1}; ^1H NMR (600 MHz, $CDCl_3$) This compound exists in multiple conformations, causing significant peak overlap. Refer to image of the ^1H NMR spectrum; ^{13}C NMR (150 MHz, $CDCl_3$) This compound exists in multiple conformations, causing significant peak overlap. Refer to image of the ^{13}C NMR spectrum; HRMS (EI): Exact mass calcd for $C_{44}H_{81}N_8O_8$ $[M+H]^+$ 849.6177, found 849.6176. MPT-4-081

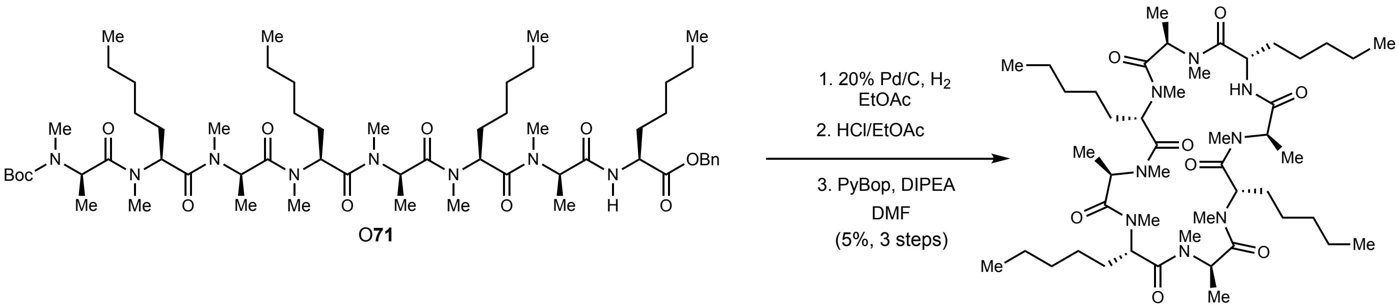

5.2

(3*S*,6*R*,9*S*,12*R*,15*S*,18*R*,21*S*,24*R*)-1,4,6,7,10,12,13,16,18,19,24-undecamethyl-3,9,15,21-tetrapentyl-1,4,7,10,13,16,19,22-octaazacyclotetracosan-2,5,8,11,14,17,20,23-octaone (5.2). A round-bottom flask was charged with the depsipeptide (95.9 mg, 86.9 µmol), dissolved in EtOAc (869 µL), and treated with 10% Pd/C (19.2 mg). The reaction flask was evacuated with light vacuum. Hydrogen (balloon) was added, and the flask was cycled once more. The reaction was allowed to stir for 1 h. After purging the flask with argon, the crude reaction mixture was filtered through Celite. To the crude material was added 4M HCl/EtOAc (1.0 mL) and the reaction mixture was allowed to stir for 30 min. The reaction

mixture was concentrated and added to a flame-dried round bottom flask. DMF (17.4 mL) was added and the reaction was cooled to 0 °C. Once at 0 °C, DIPEA (34.3 µL, 261 µmol) and PyBop (47.5 mg, 91.2 µmol) were added. The reaction was stirred at 0 °C for 0.5 h, and then allowed to warm to ambient temperature to stir for an additional 2.5 h. The reaction mixture was poured into cold 10% aq citric acid and extracted with EtOAc. The combined organic layers were washed with satd aq $NaHCO_3$, brine, and then dried and concentrated. Preparative HPLC purification (5 – 95% aqueous acetonitrile, 210 nm, flow rate: 18 mL/min, R_t = 24.2 m) afforded the 24-membered macrocycle (4.1 mg, 5.3%) as a yellow oil. $[\alpha]_D^{24}$ - 5.1 (*c* 0.12, $CHCl_3$); R_f = 0.61 (60% EtOAc in hexanes); IR (film) 3478, 2922, 2851, 1740, 1633, 1455, 1260 cm^{-1}; ^1H NMR (600 MHz, $CDCl_3$) This compound exists in multiple conformations, causing significant peak overlap. Refer to image of the ^1H NMR spectrum; ^{13}C NMR (150 MHz, $CDCl_3$) This compound exists in multiple conformations, causing significant peak overlap. Refer to image of the ^{13}C NMR spectrum; HRMS (EI): Exact mass calcd for $C_{47}H_{87}N_8O_8$ $[M+H]^+$ 891.6647, found 891.6643. MPT-4-184

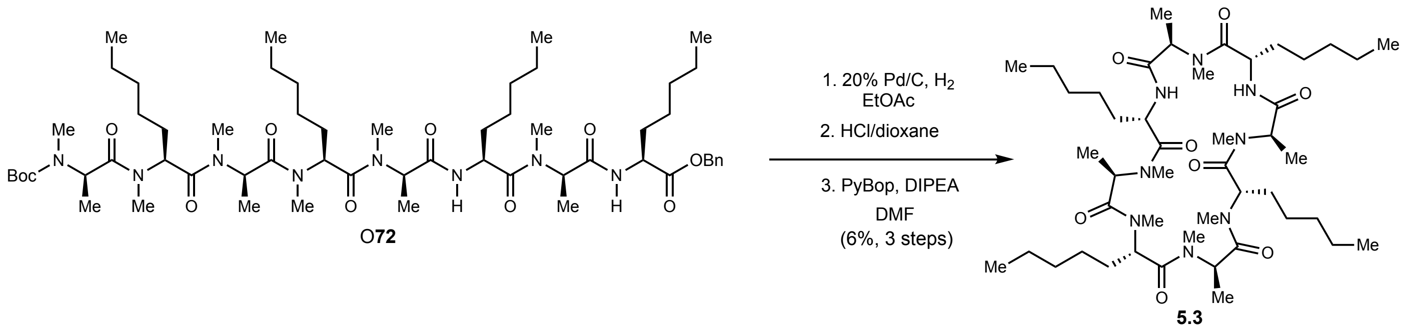

(3*S*,6*R*,9*S*,12*R*,15*S*,18*R*,21*S*,24*R*)-1,4,6,7,10,12,13,18,19,24-decamethyl-3,9,15,21-tetrapentyl-1,4,7,10,13,16,19,22-octaazacyclotetracosan-2,5,8,11,14,17,20,23-octaone (5.3). A round-bottom flask was charged with the depsipeptide (90.2 mg, 83.2 µmol), dissolved in EtOAc (832 µL), and treated with 10% Pd/C (18.0 mg). The reaction flask was evacuated with light vacuum. Hydrogen (balloon) was added, and the flask was cycled once more. The reaction was allowed to stir for 1 h. After purging the flask with argon, the crude reaction mixture was filtered through Celite. To the crude material was added 4M HCl/dioxane (1.00 mL) and the reaction mixture was allowed to stir for 30 min. The reaction mixture was concentrated and added to a flame-dried round bottom flask. DMF (16.7 mL) was added and the reaction was cooled to 0 °C. Once at 0 °C, DIPEA (32.8 µL, 250 µmol) and PyBop (44.9 mg, 87.4 µmol) were added. The reaction was stirred at 0 °C for 0.5 h, and then allowed to warm to ambient temperature to stir for an additional 2.5 h. The reaction mixture was poured into cold 10% aq citric acid and extracted with EtOAc. The combined organic layers were washed with satd aq $NaHCO_3$, brine, and then dried and concentrated. Preparative HPLC purification (5 – 95% aqueous acetonitrile, 210 nm, flow rate: 18 mL/min, R_t = 22.9 m) afforded the 24-membered macrocycle (4.3 mg, 5.9%) as a light yellow oil. $[\alpha]_D^{24}$ - 3.3 (*c* 0.19, $CHCl_3$); R_f = 0.55 (60% EtOAc in hexanes); IR (film) 3284, 2929, 2860, 1744, 1649, 1554, 1210, 1066 cm^{-1}; ^1H NMR (600 MHz, $CDCl_3$) This compound exists in multiple conformations, causing significant peak overlap. Refer to image of the ^1H NMR spectrum; ^{13}C NMR (150 MHz, $CDCl_3$) This compound exists in multiple conformations, causing significant peak overlap. Refer to image of the ^{13}C NMR spectrum; HRMS (EI): Exact mass calcd for $C_{46}H_{85}N_8O_8$ $[M+H]^+$ 877.6490, found 877.6489. MPT-5-149

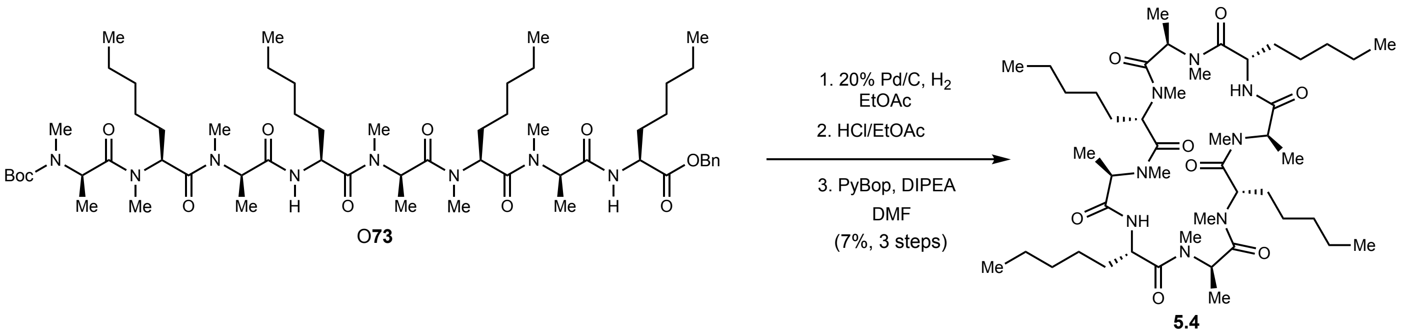

(3*S*,6*R*,9*S*,12*R*,15*S*,18*R*,21*S*,24*R*)-1,4,6,7,12,13,16,18,19,24-decamethyl-3,9,15,21-tetrapentyl-1,4,7,10,13,16,19,22-octaazacyclotetracosan-2,5,8,11,14,17,20,23-octaone (5.4). A round-bottom flask was charged with the depsipeptide (66.9 mg, 61.7 µmol), dissolved in EtOAc (617 µL), and treated with 10% Pd/C (13.4 mg). The reaction flask was evacuated with light vacuum. Hydrogen (balloon) was added, and the flask was cycled once more. The reaction was allowed to stir for 1 h. After purging the flask with argon, the crude reaction mixture was filtered through Celite. To the crude material was added 4M HCl/EtOAc (1.00 mL) and the reaction mixture was allowed to stir for 30 min. The reaction mixture was concentrated and added to a flame-dried round bottom flask. Dry DMF (12.4 mL) was added and the reaction was cooled to 0 °C. Once at 0 °C, DIPEA (24.3 µL, 185 µmol) and PyBop (33.4 mg, 64.5 µmol) were added. The reaction was stirred at 0 °C for 0.5 h, then allowed to warm to ambient temperature and stir for an additional 2.5 h. The reaction mixture was poured into cold 10% aq citric acid and extracted with EtOAc. The combined organic layers were washed with satd aq $NaHCO_3$, brine, and then dried and concentrated. Preparative HPLC purification (5 – 95% aqueous acetonitrile, 210 nm, flow rate: 18 mL/min, R_t = 23.1 m) afforded the 24-membered macrocycle (3.9 mg, 7.2%) as a light yellow oil. $[\alpha]_D^{24}$ - 4.2 (*c* 0.11, $CHCl_3$); R_f = 0.37 (60% EtOAc in hexanes); IR (film) 3419, 2923, 2852, 1747, 1653, 1541, 1260, 1100 cm^{-1}; ^{1}H NMR (600 MHz, $CDCl_3$) This compound exists in multiple conformations, causing significant peak overlap. Refer to image of the ^{1}H NMR spectrum; ^{13}C NMR (150 MHz, $CDCl_3$); HRMS (EI): Exact mass calcd for $C_{46}H_{85}N_8O_8$ [M+H]$^+$ 877.6490, found 877.6486. MPT-5-212

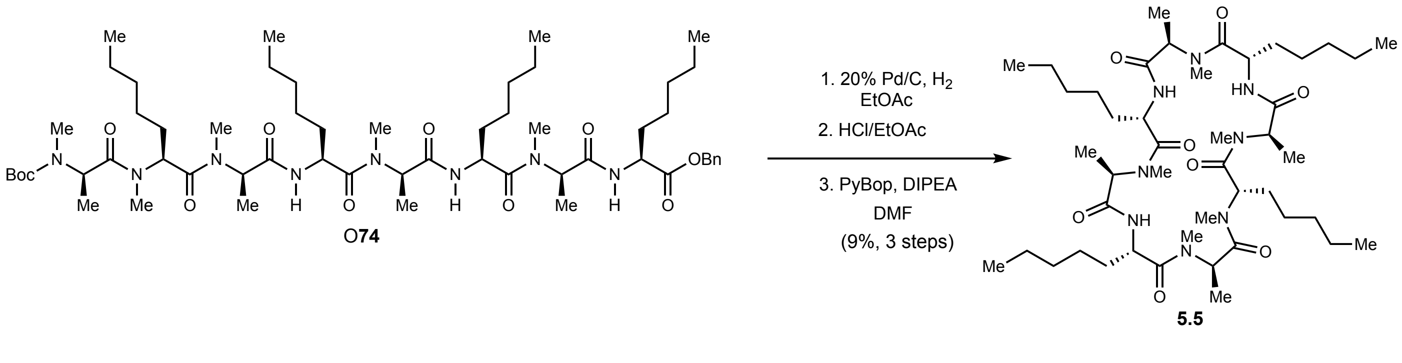

(3*S*,6*R*,9*S*,12*R*,15*S*,18*R*,21*S*,24*R*)-1,4,6,7,12,13,18,19,24-nonamethyl-3,9,15,21-tetrapentyl-1,4,7,10,13,16,19,22-octaazacyclotetracosan-2,5,8,11,14,17,20,23-octaone (5.5). A round-bottom flask was charged with the depsipeptide (98 mg, 91.5 µmol), dissolved in EtOAc (915 µL), and treated with 10% Pd/C (19.6 mg). The reaction flask was evacuated with

light vacuum. Hydrogen (balloon) was added, and the flask was cycled once more. The reaction was allowed to stir for 1 h. After purging the flask with argon, the crude reaction mixture was filtered through Celite. To the crude material was added 4M HCl/EtOAc (1.0 mL) and the reaction mixture was allowed to stir for 30 min. The reaction mixture was concentrated and added to a flame-dried round bottom flask. Dry DMF (21.4 mL) was added and the reaction was cooled to 0 °C. Once at 0 °C, DIPEA (36.1 µL, 275 µmol) and PyBop (50.0 mg, 96.1 µmol) were added. The reaction was stirred at 0 °C for 0.5 h, and then allowed to warm to ambient temperature to stir for an additional 2.5 h. The reaction mixture was poured into cold 10% aq citric acid and extracted with EtOAc. The combined organic layers were washed with satd aq $NaHCO_3$, brine, and then dried and concentrated. Preparative HPLC purification (5 – 95% aqueous acetonitrile, 210 nm, flow rate: 18 mL/min, R_t = 23.9 m) afforded the 24-membered macrocycle (6.1 mg, 9.0%) as a light yellow oil. $[\alpha]_D^{24}$ - 2.1 (*c* 0.12, $CHCl_3$); R_f = 0.43 (60% EtOAc in hexanes); IR (film) 3292, 2929, 2843, 1654, 1637, 1560, 1458 cm^{-1}; ^{1}H NMR (600 MHz, $CDCl_3$) This compound exists in multiple conformations, causing significant peak overlap. Refer to image of the ^{1}H NMR spectrum; ^{13}C NMR (150 MHz, $CDCl_3$) This compound exists in multiple conformations, causing significant peak overlap. Refer to image of the ^{13}C NMR spectrum; HRMS (EI): Exact mass calcd for $C_{45}H_{83}N_8O_8$ [M+H]$^+$ 863.6328, found 863.6325. MPT-4-227

Characterization NMR Spectra

Figure S1. ^{1}H NMR (600 MHz, $CDCl_3$) of **14**

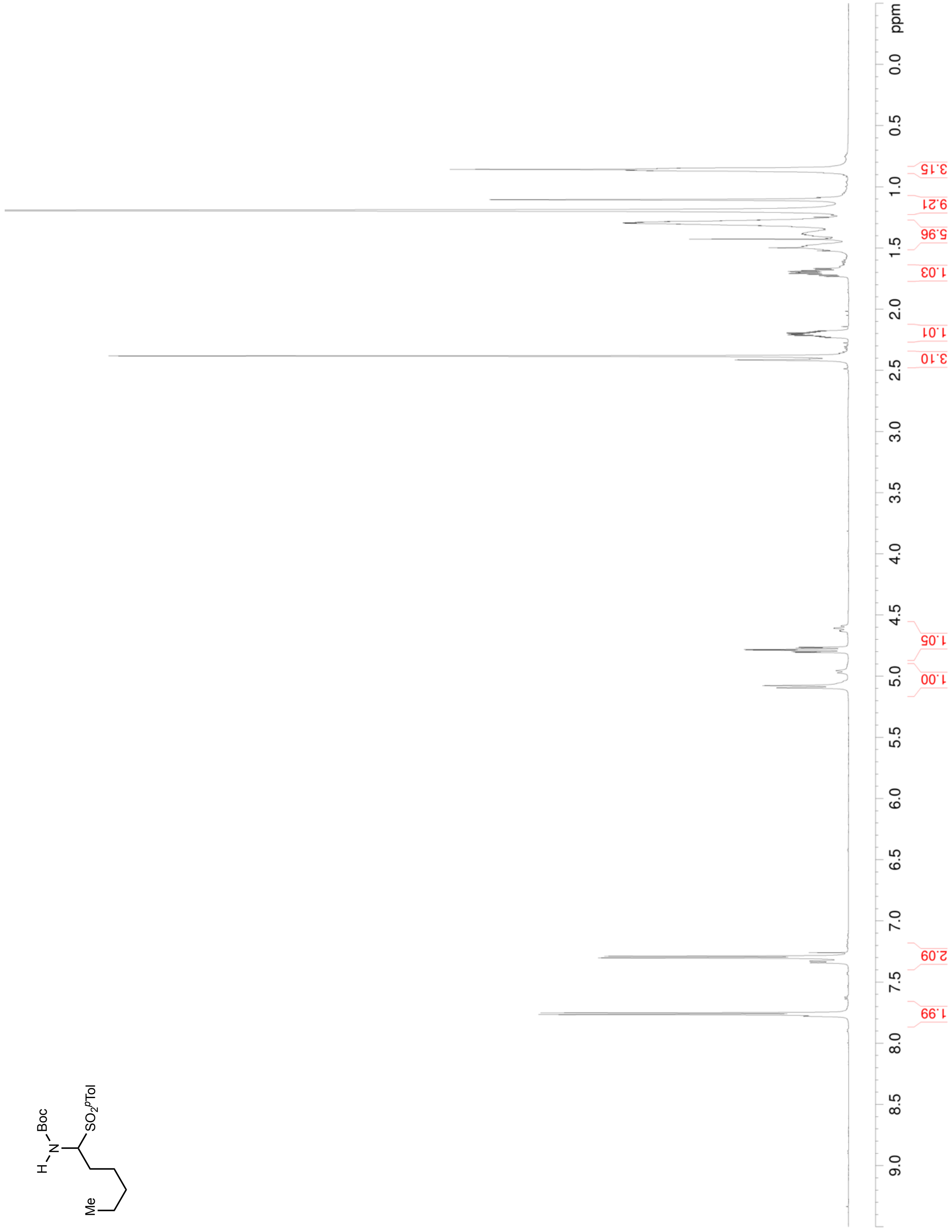

Figure S2. ^{13}C NMR (150 MHz, $CDCl_3$) of **14**

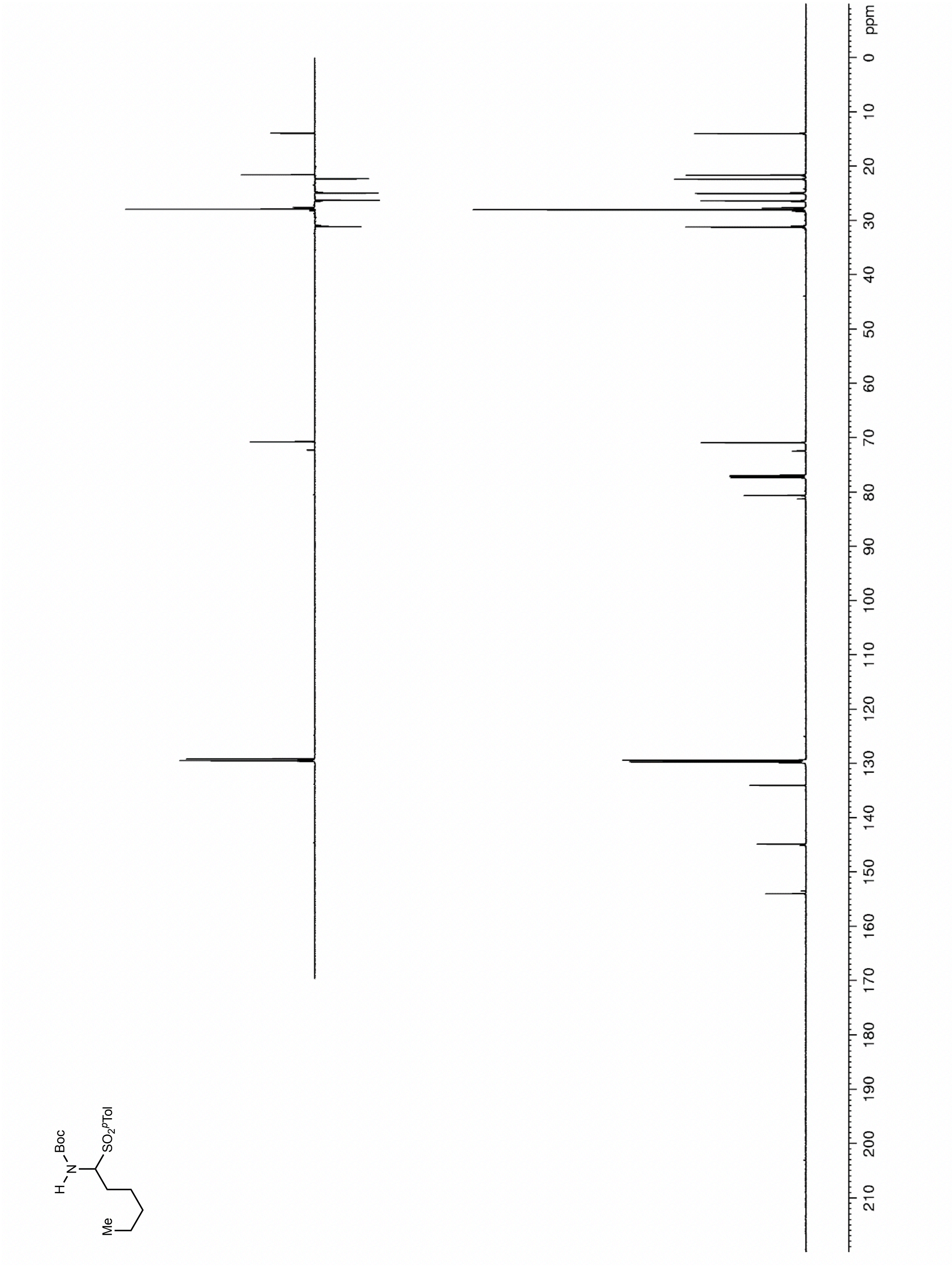

Figure S3. ^{1}H NMR (600 MHz, $CDCl_3$) of **16**

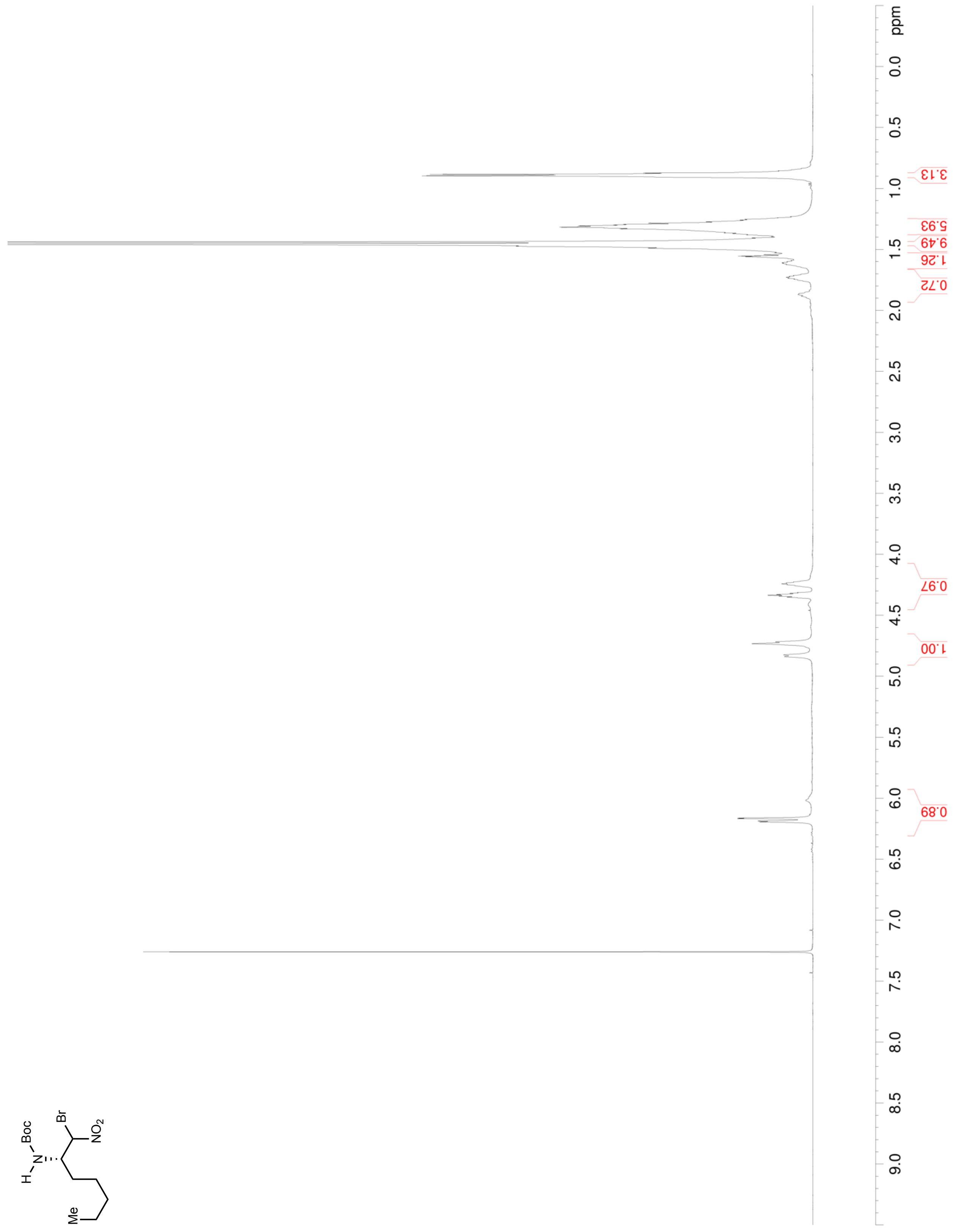

Figure S4. ^{13}C NMR (150 MHz, $CDCl_3$) of **16**

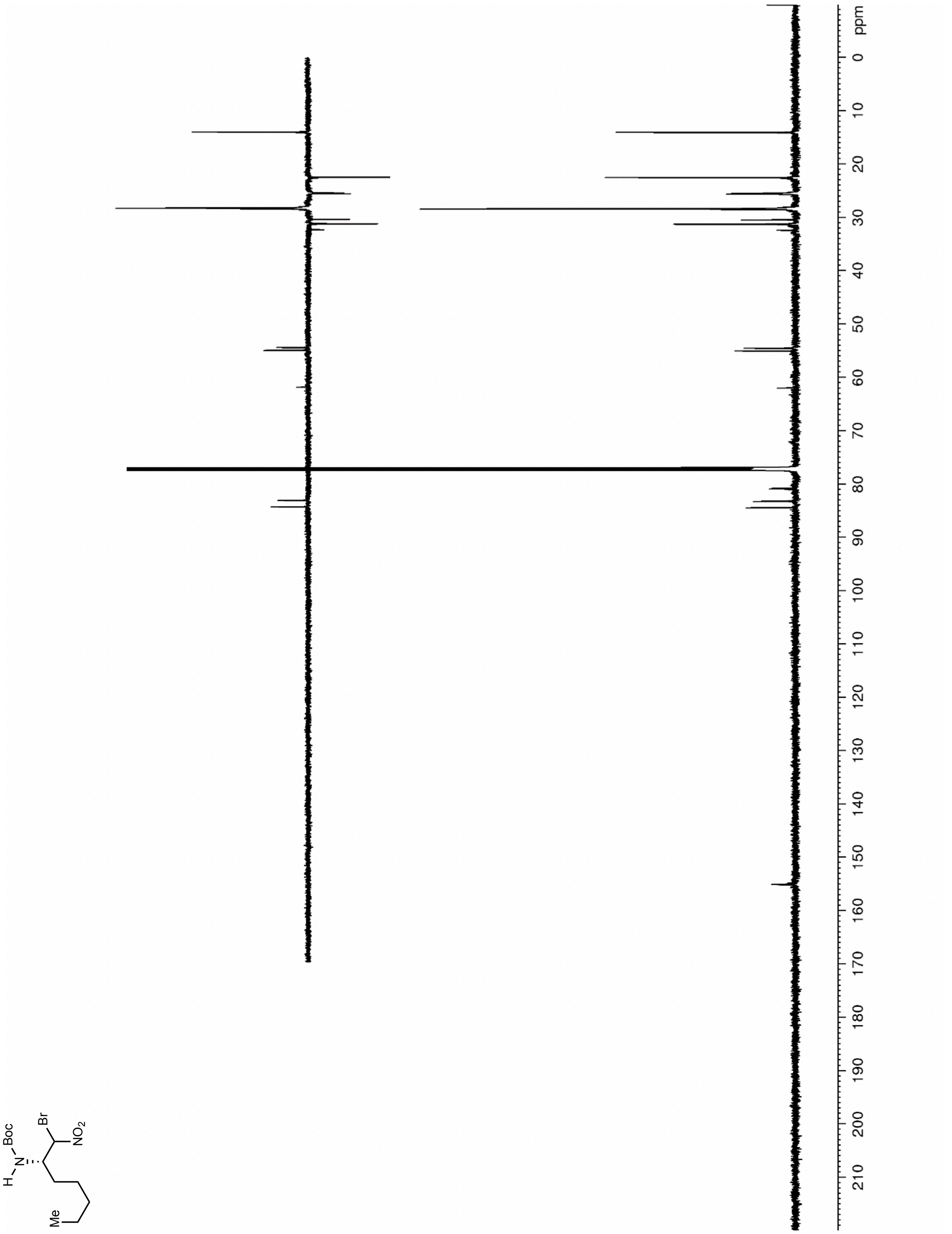

Figure S5. ^{1}H NMR (600 MHz, $CDCl_3$) of **19**

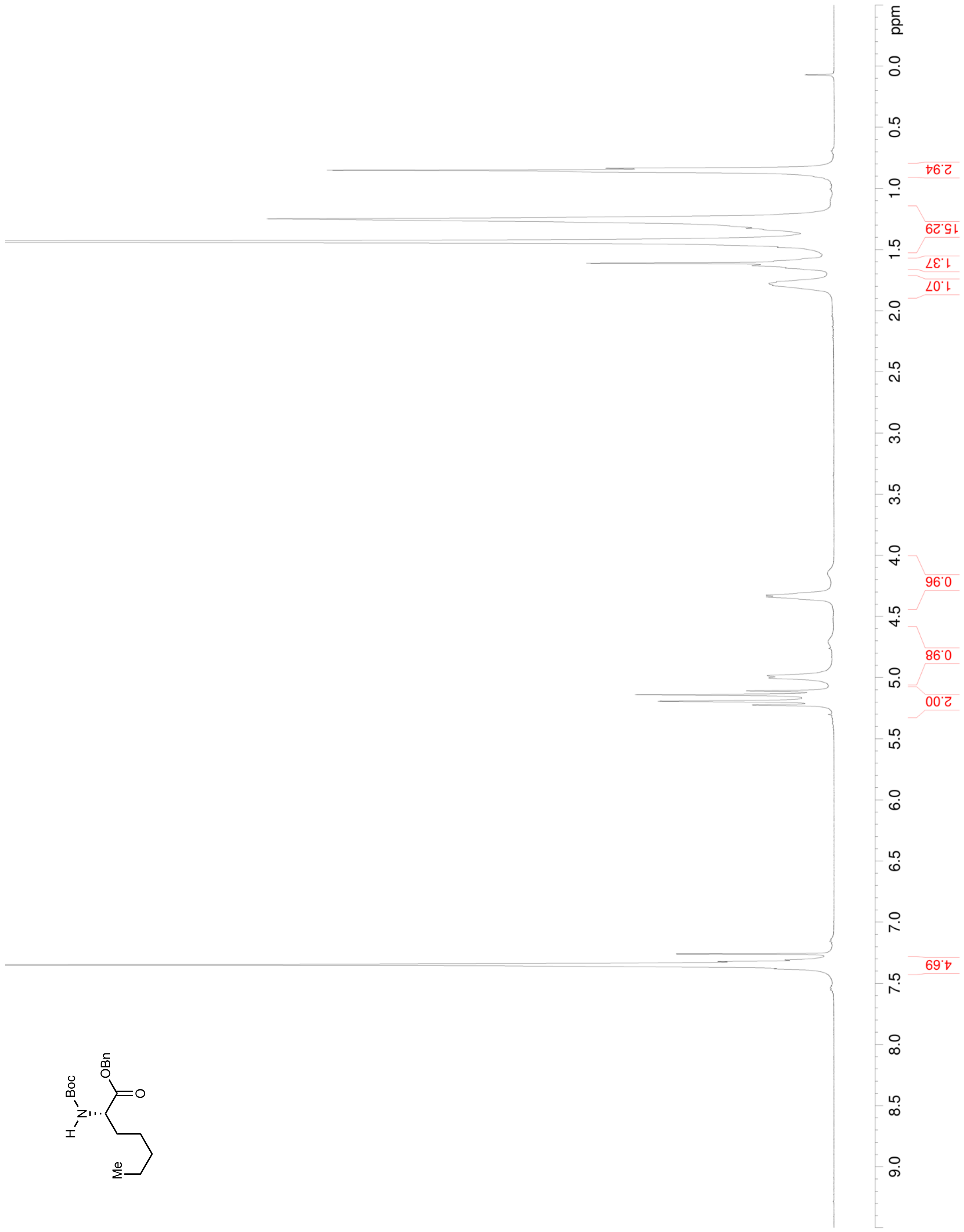

Figure S6. ^{13}C NMR (150 MHz, $CDCl_3$) of **19**

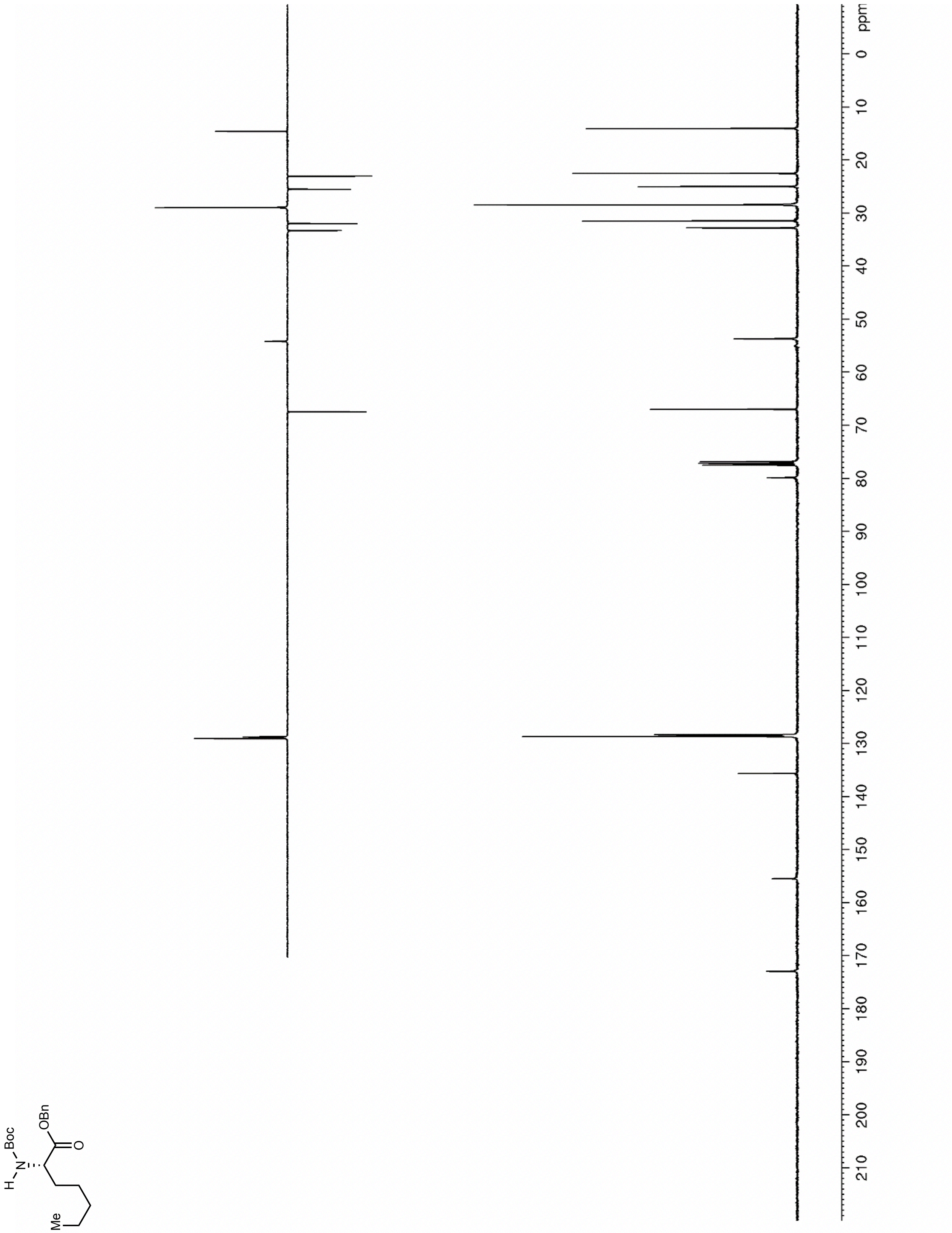

Figure S7. ^{1}H NMR (600 MHz, $CDCl_3$) of **21**

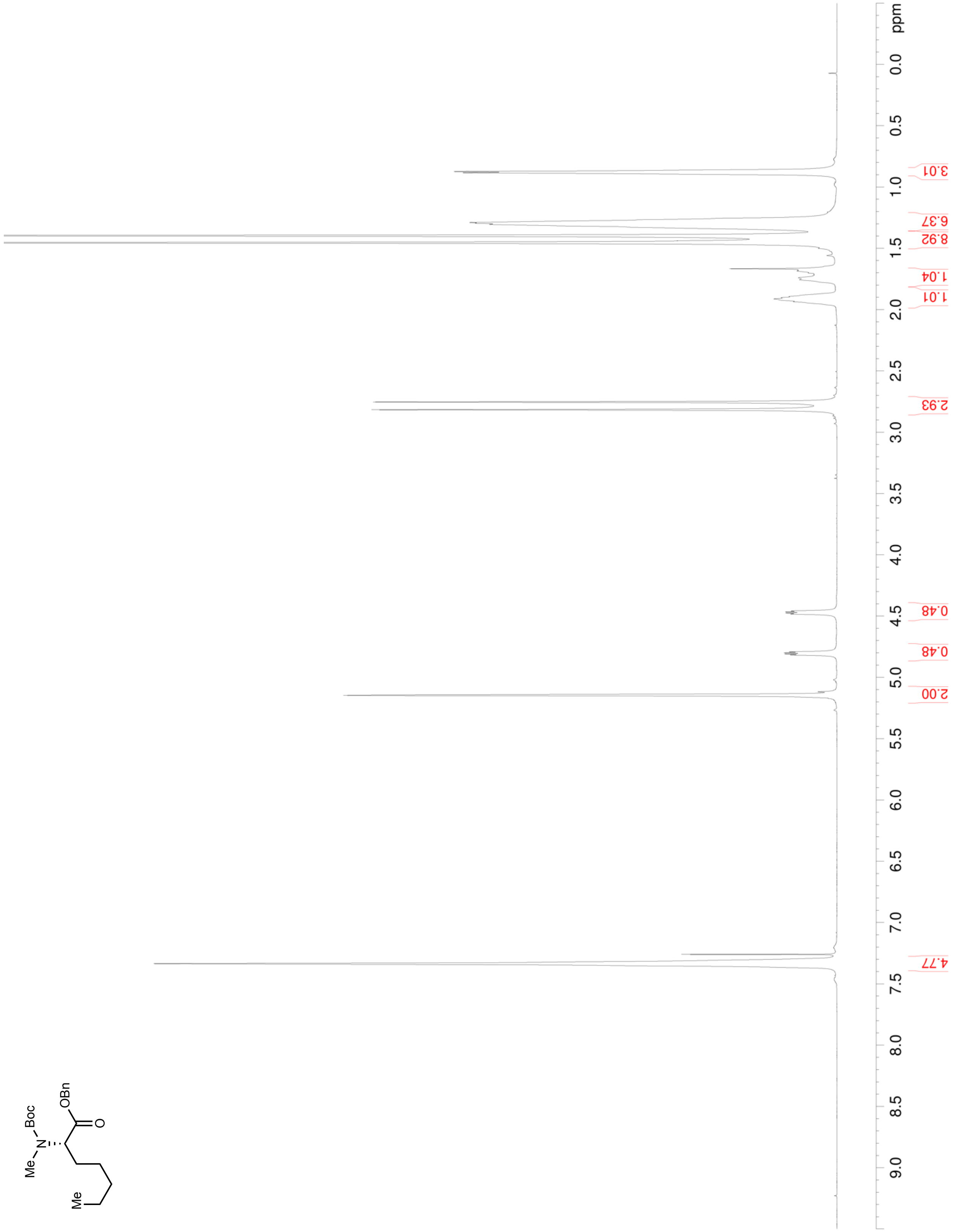

Figure S8. ^{13}C NMR (150 MHz, $CDCl_3$) of **21**

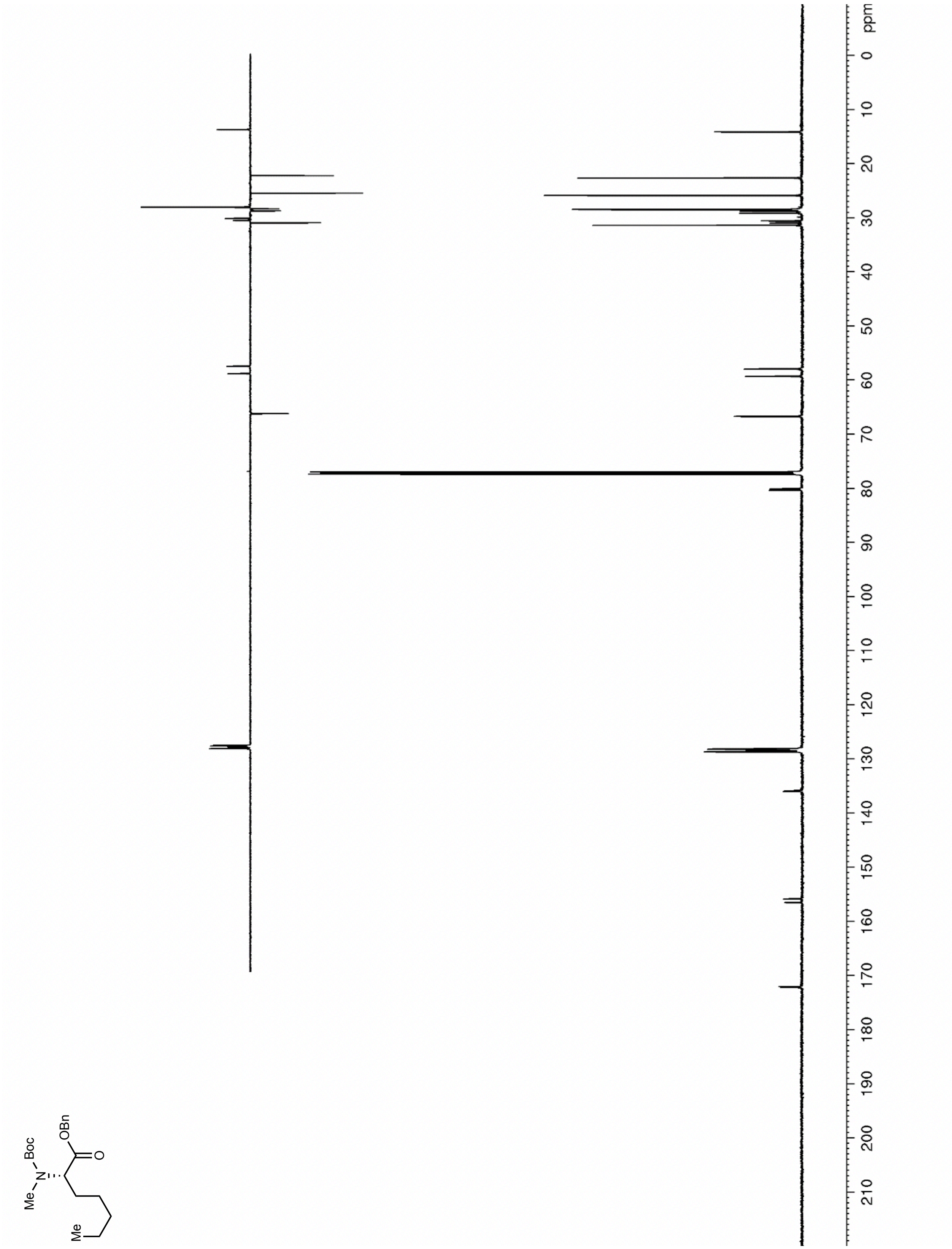

Figure S9. ^{1}H NMR (600 MHz, $CDCl_3$) of **23**

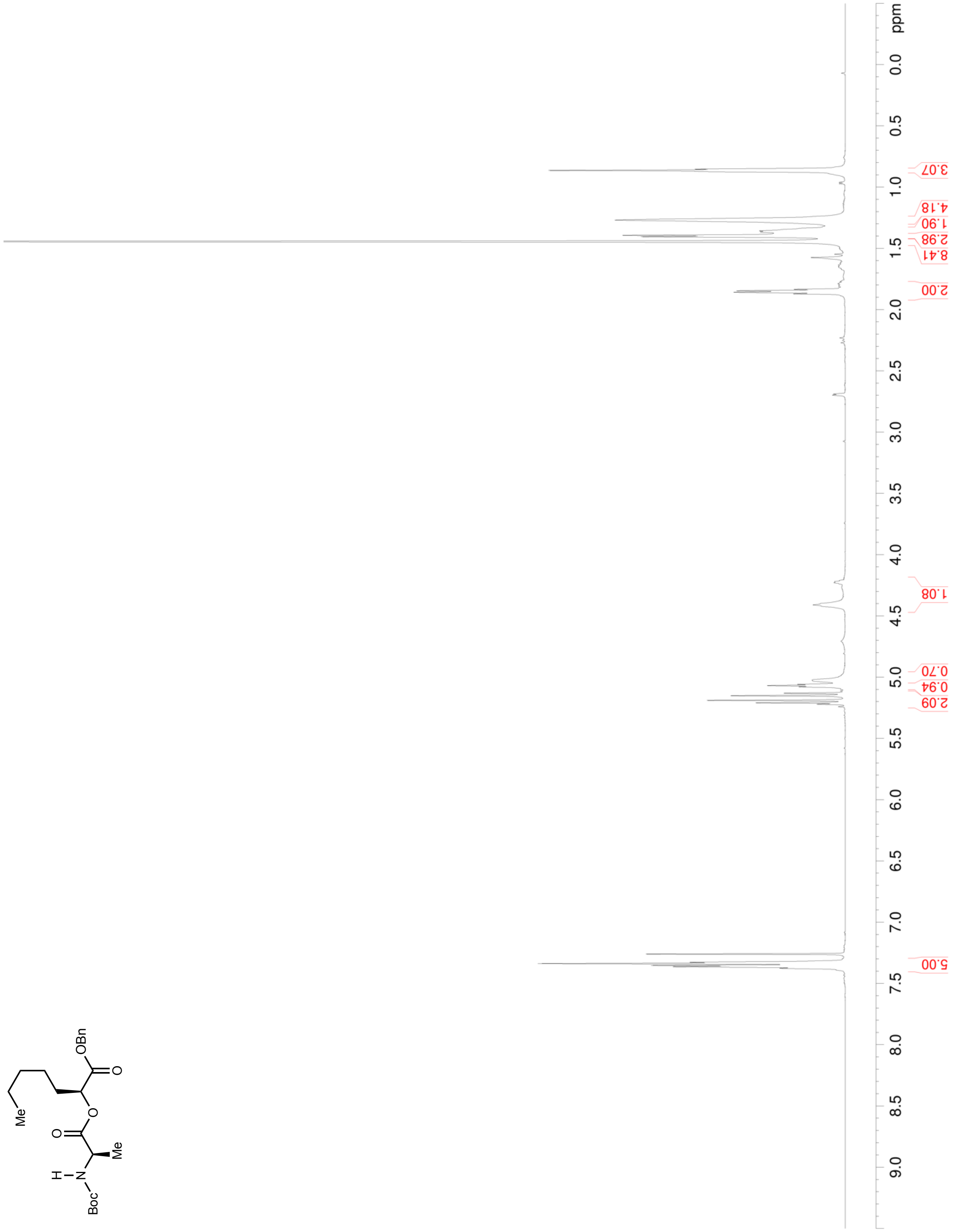

Figure S10. ^{13}C NMR (150 MHz, $CDCl_3$) of **23**

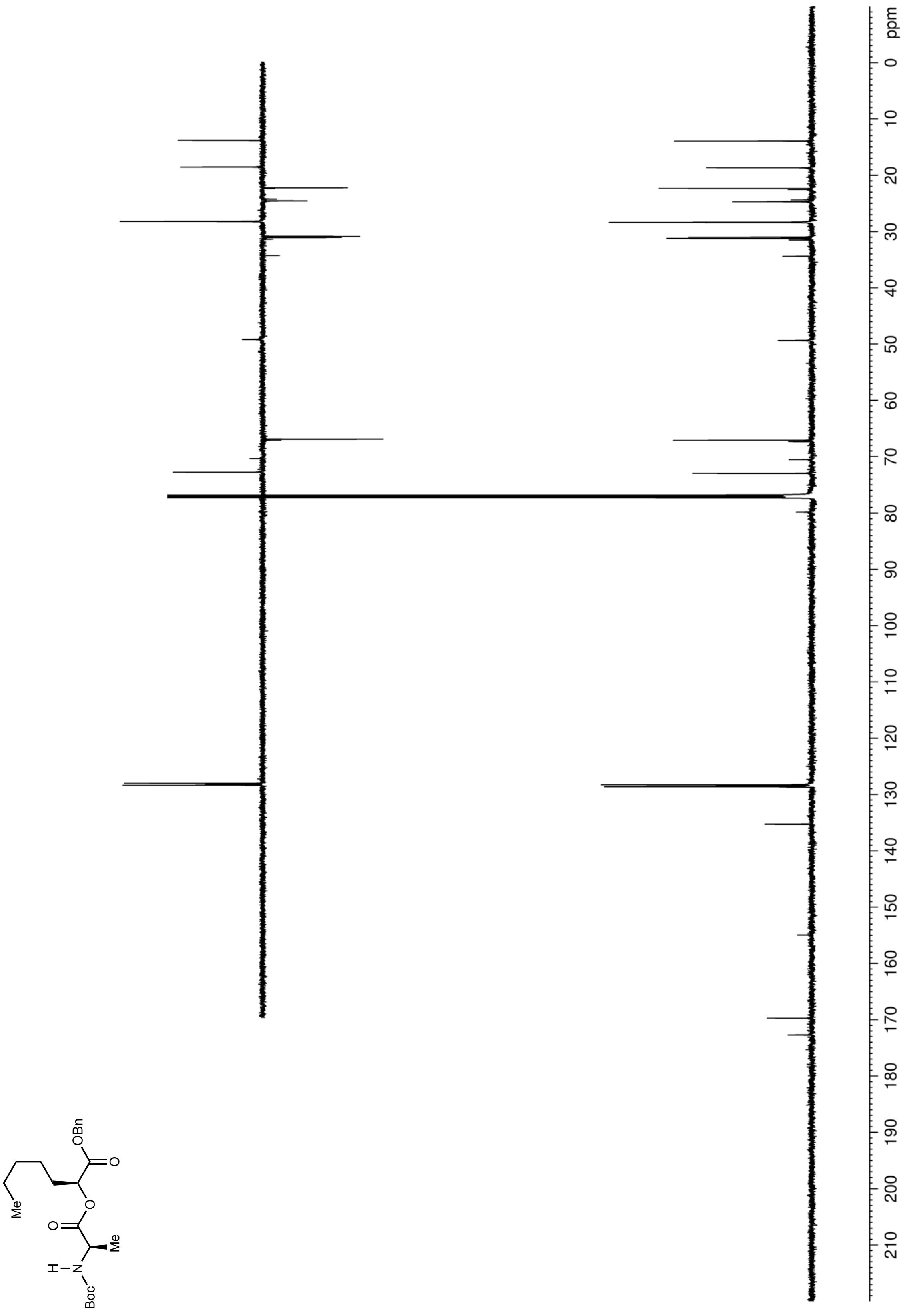

Figure S11. ^{1}H NMR (600 MHz, $CDCl_3$) of **29**

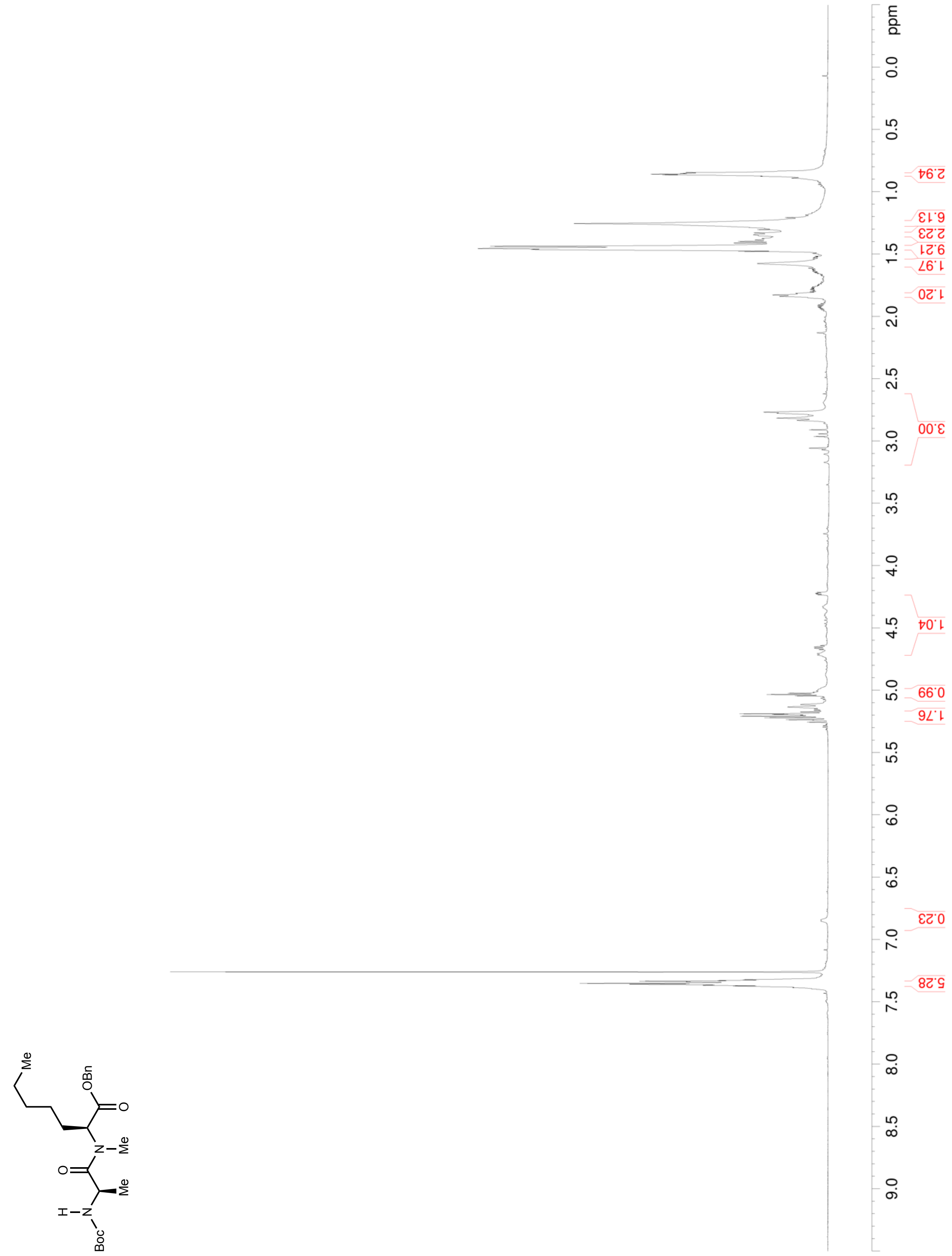

Figure S12. ^{13}C NMR (150 MHz, $CDCl_3$) of **29**

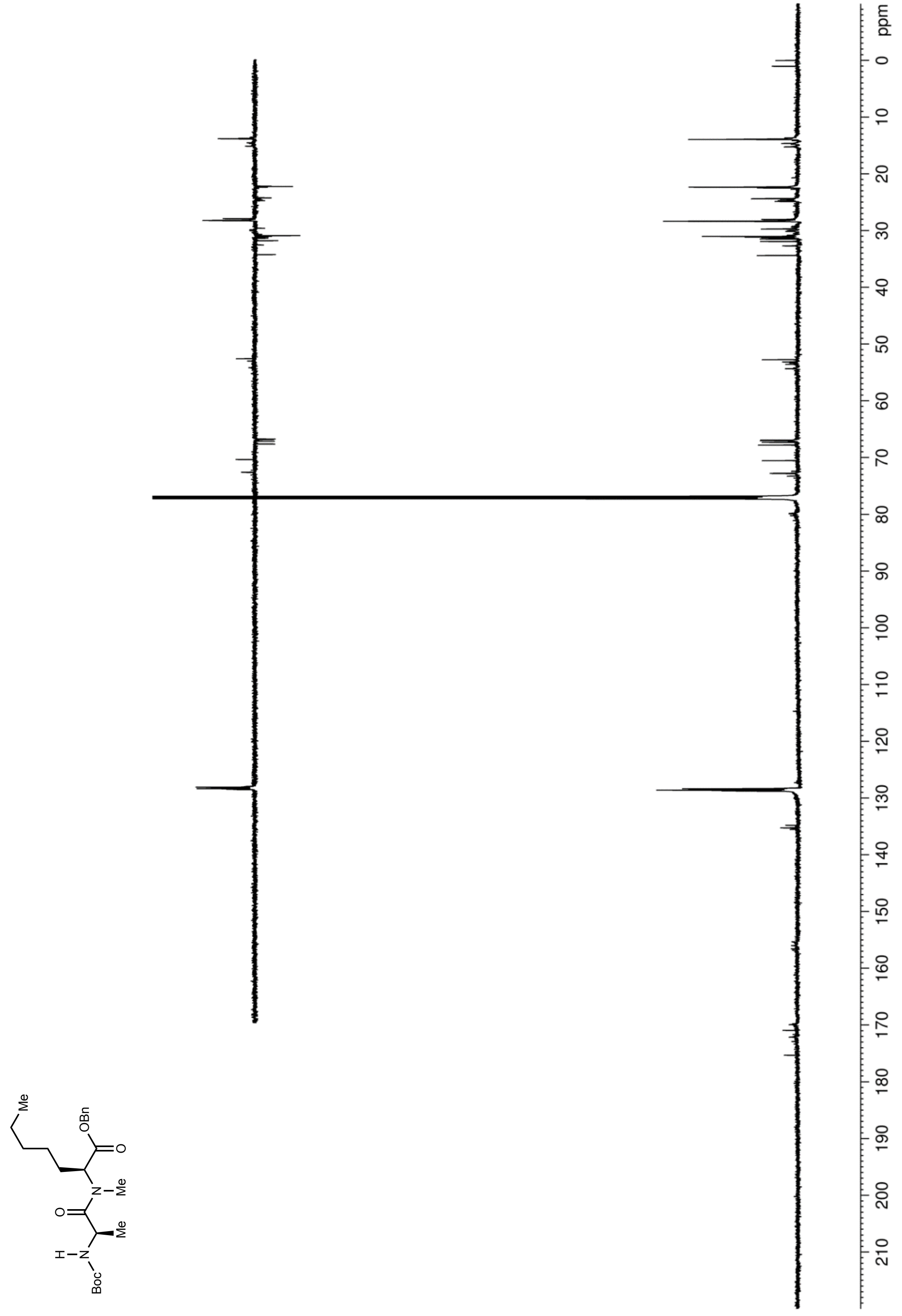

Figure S13. ^{1}H NMR (600 MHz, $CDCl_3$) of **32**

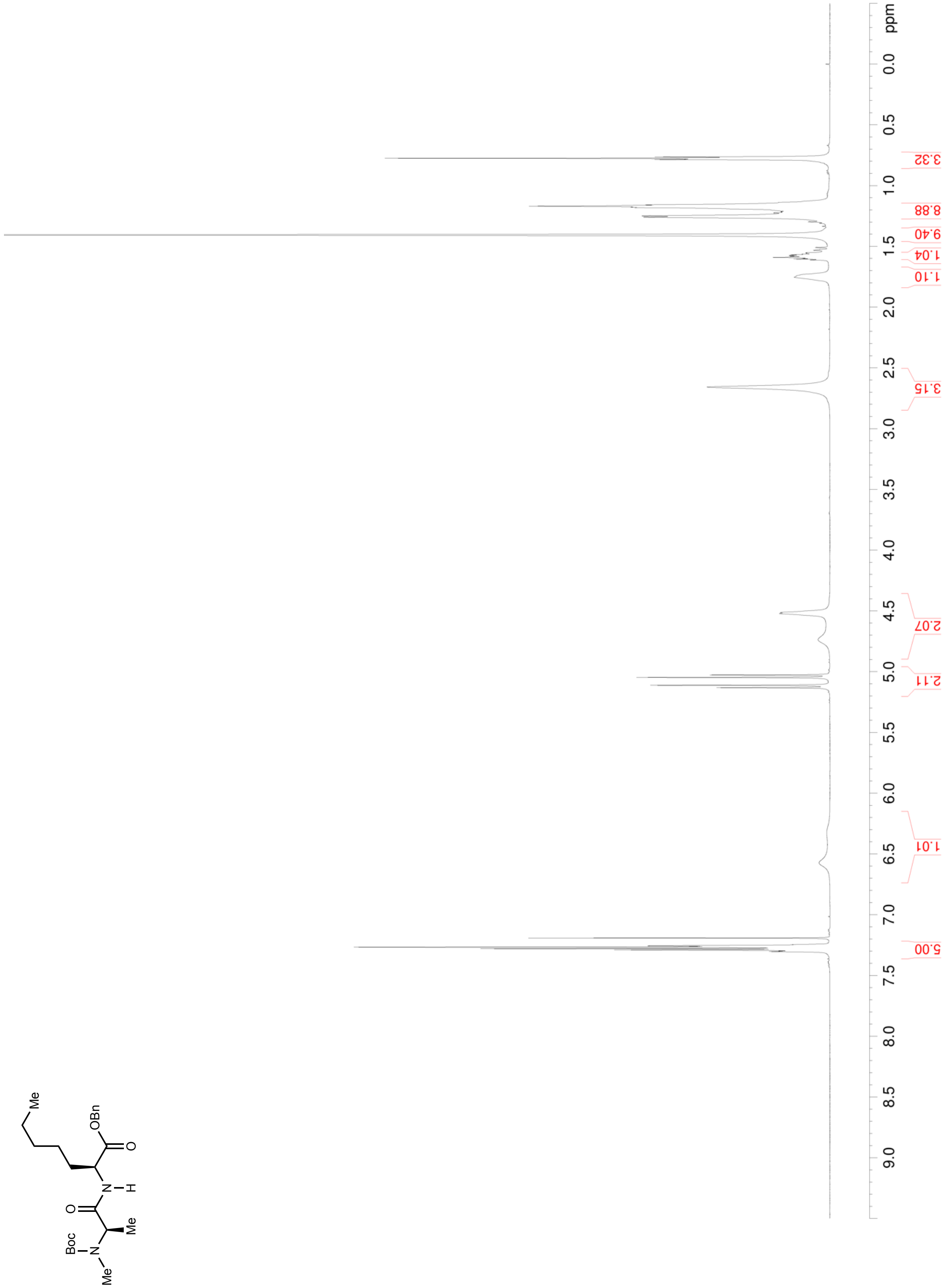

Figure S14. ^{13}C NMR (150 MHz, $CDCl_3$) of **32**

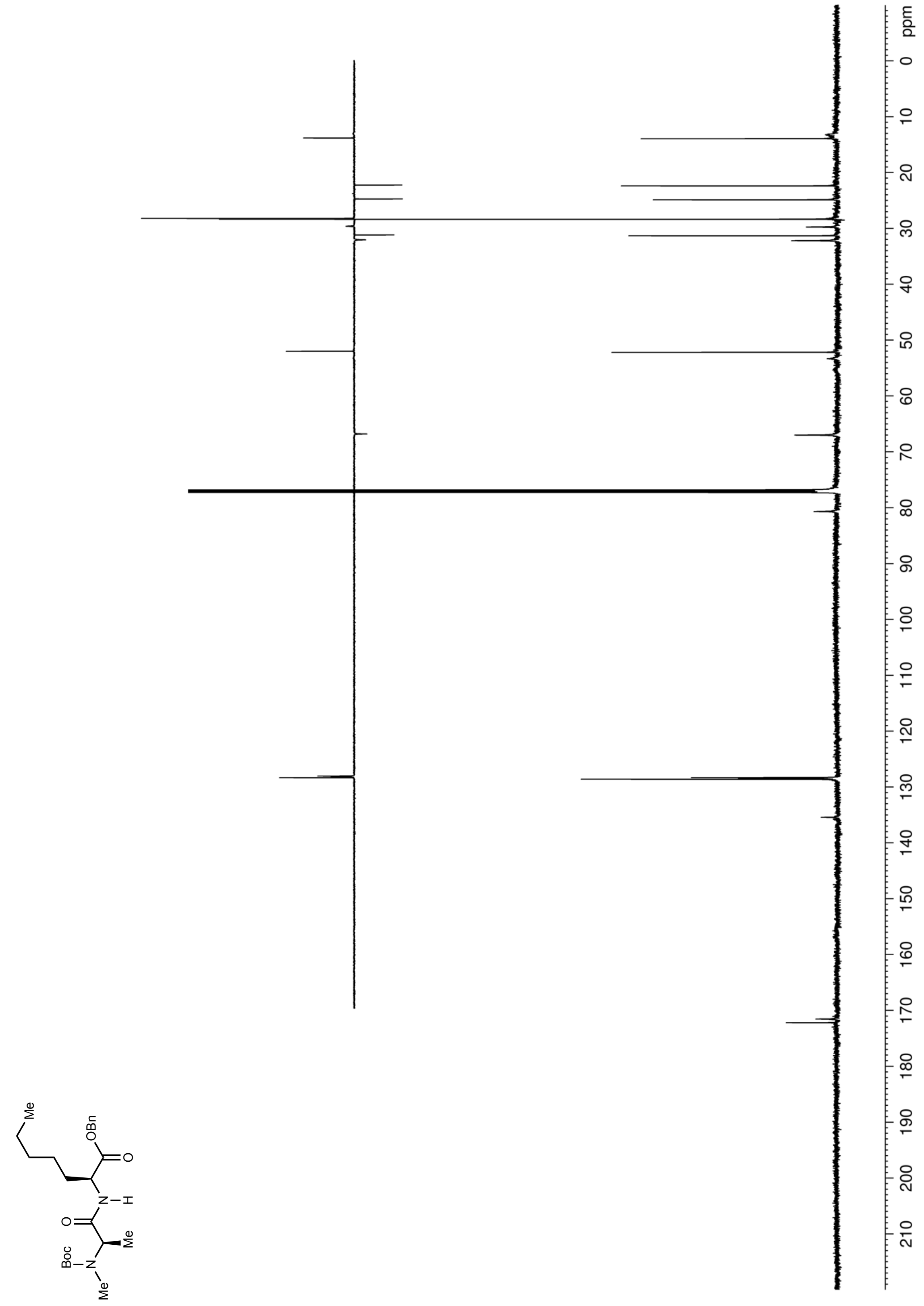

Figure S15. ^{1}H NMR (600 MHz, $CDCl_3$) of **35**

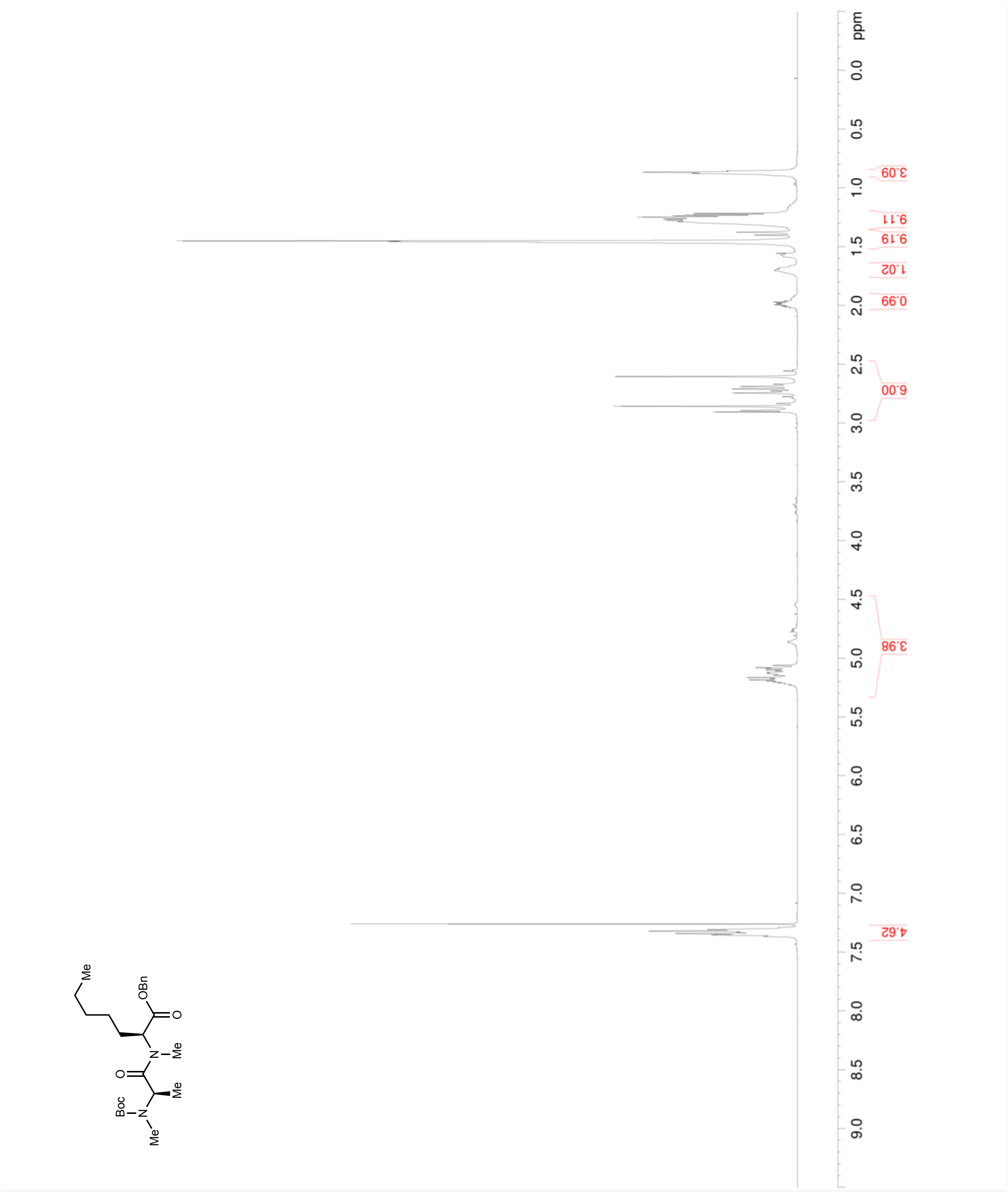

Figure S16. ^{13}C NMR (150 MHz, $CDCl_3$) of **35**

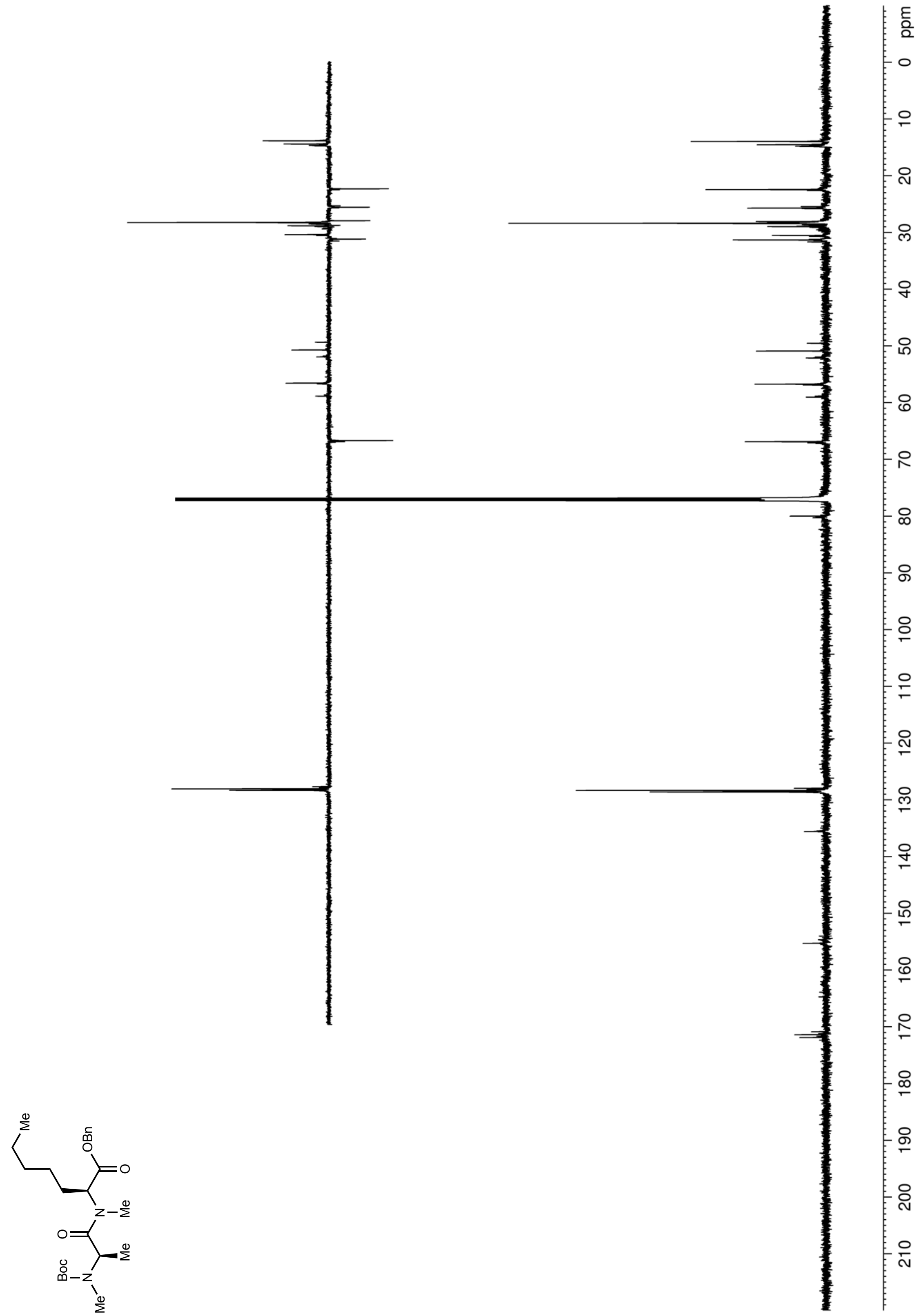

Figure S17. ^{1}H NMR (600 MHz, $CDCl_3$) of **T38**

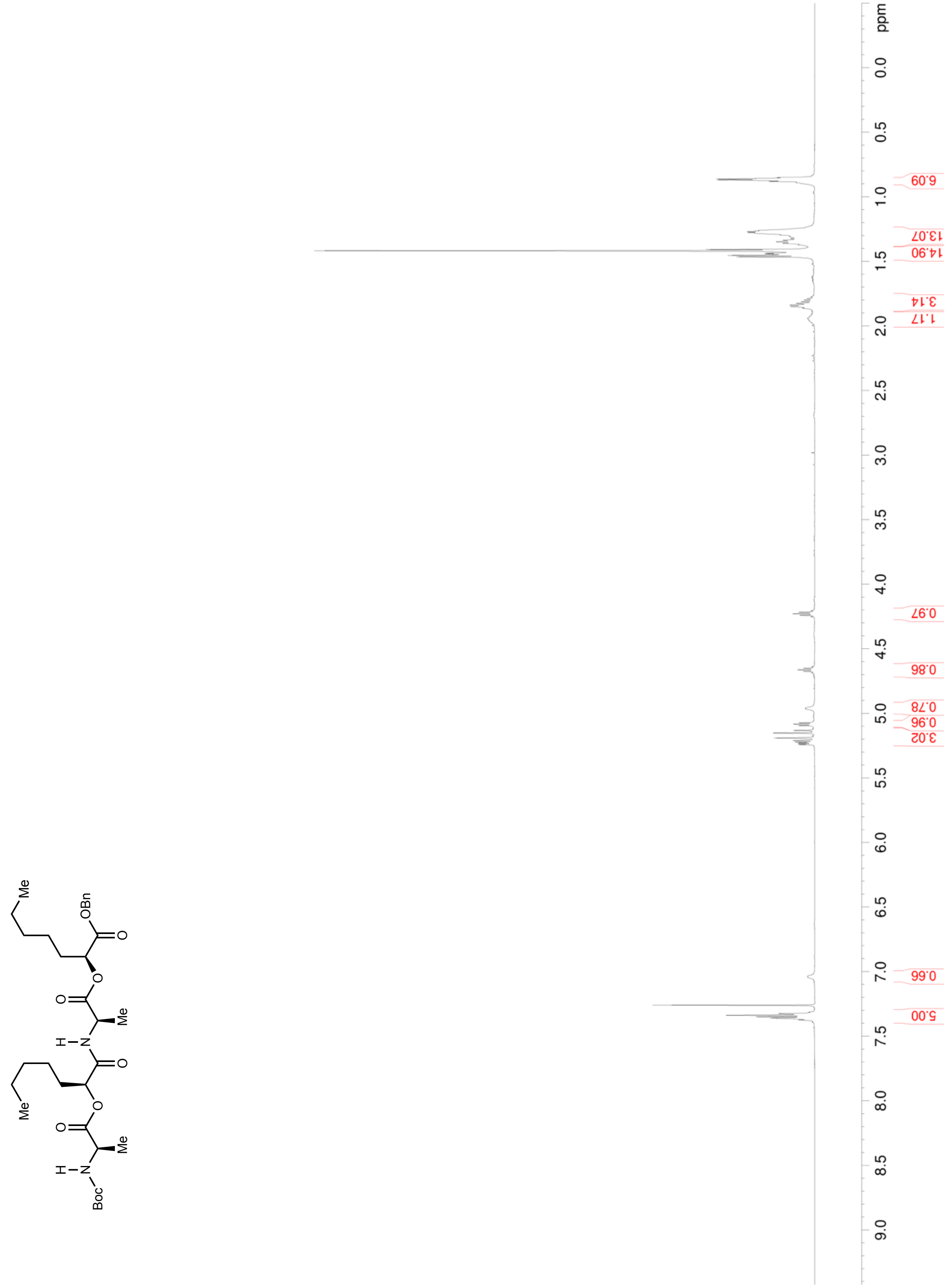

Figure S18. ^{13}C NMR (150 MHz, $CDCl_3$) of **T38**

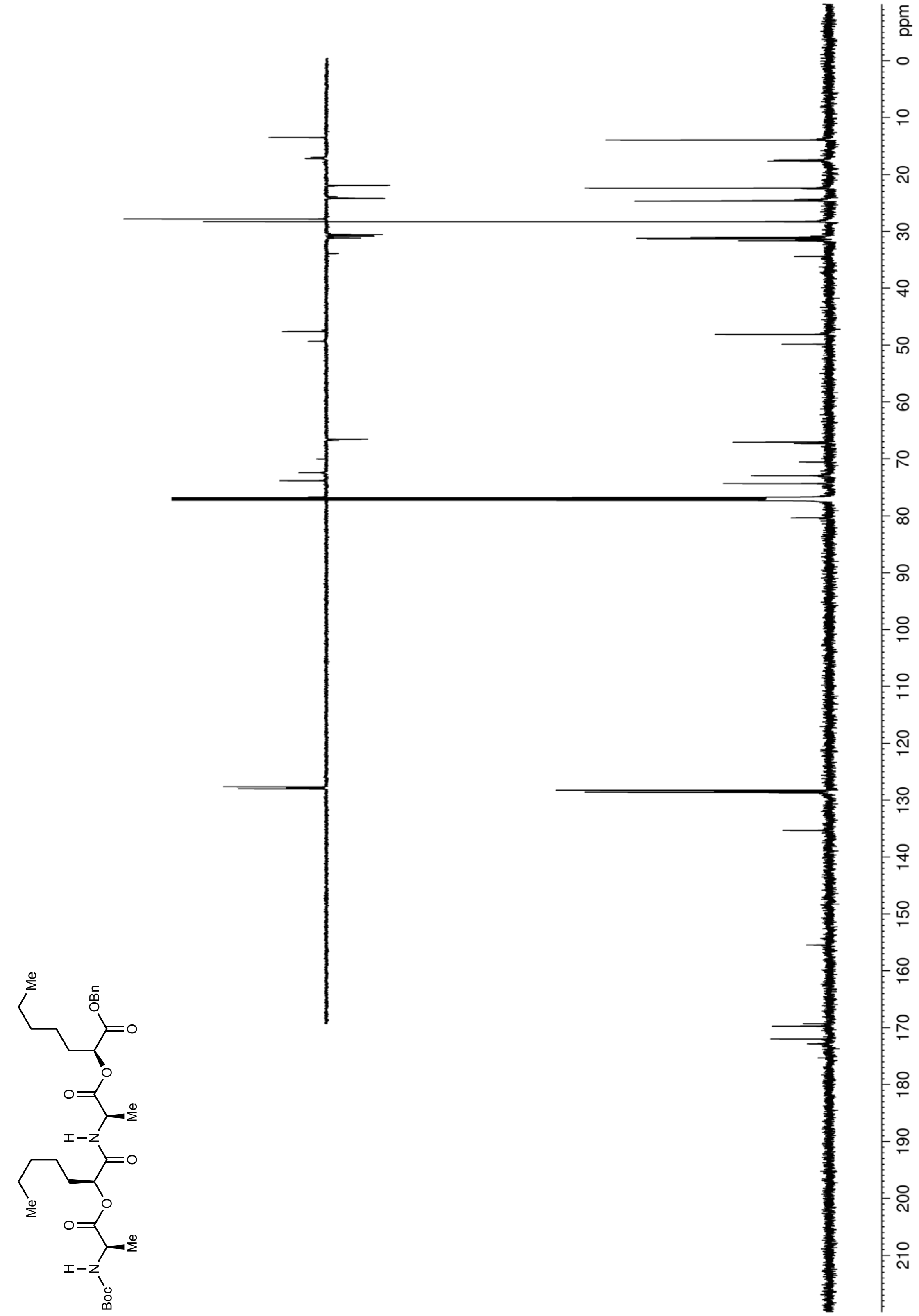

Figure S19. ^{1}H NMR (600 MHz, $CDCl_3$) of **T40**

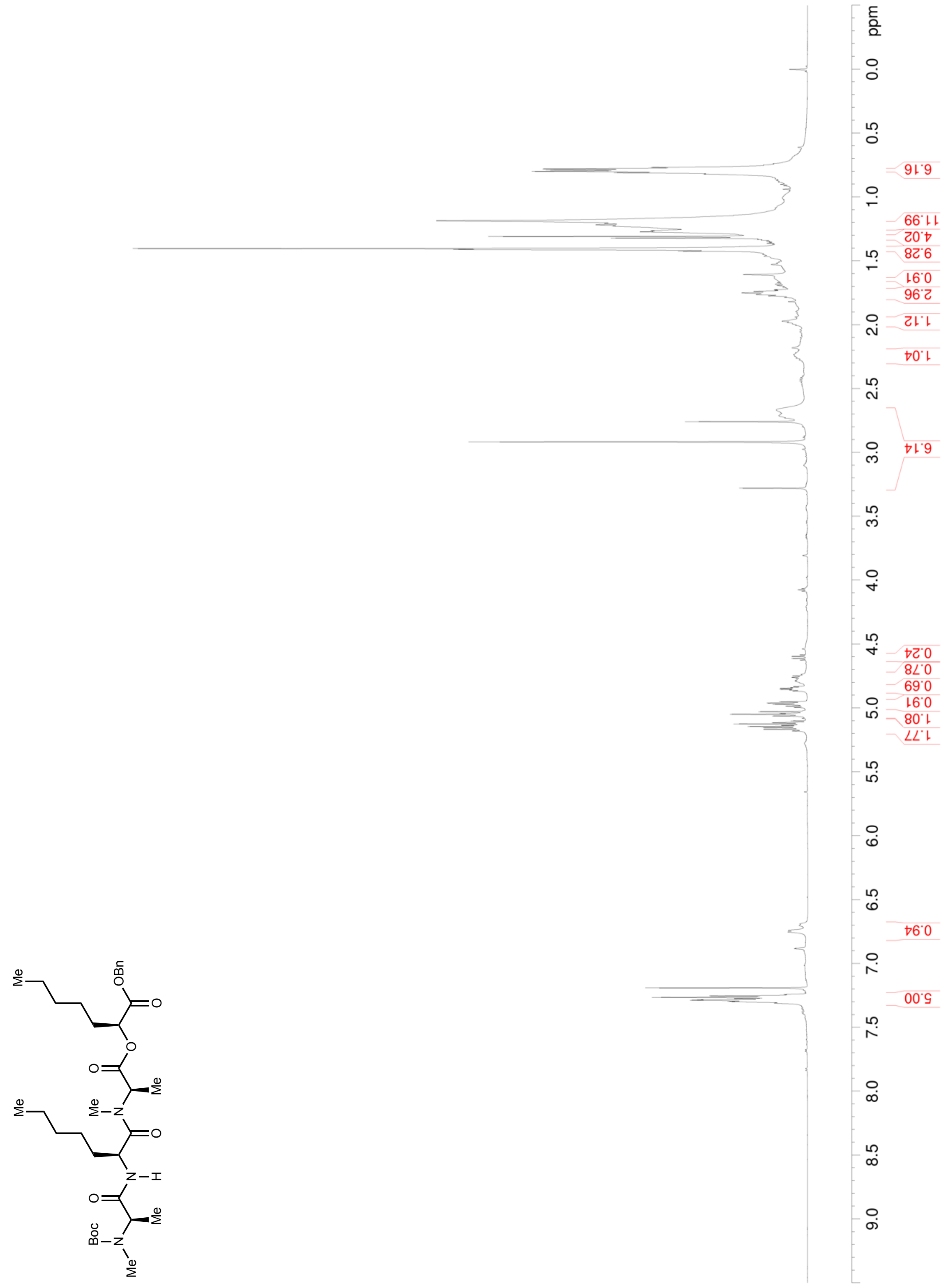

Figure S20. ^{13}C NMR (150 MHz, $CDCl_3$) of **T40**

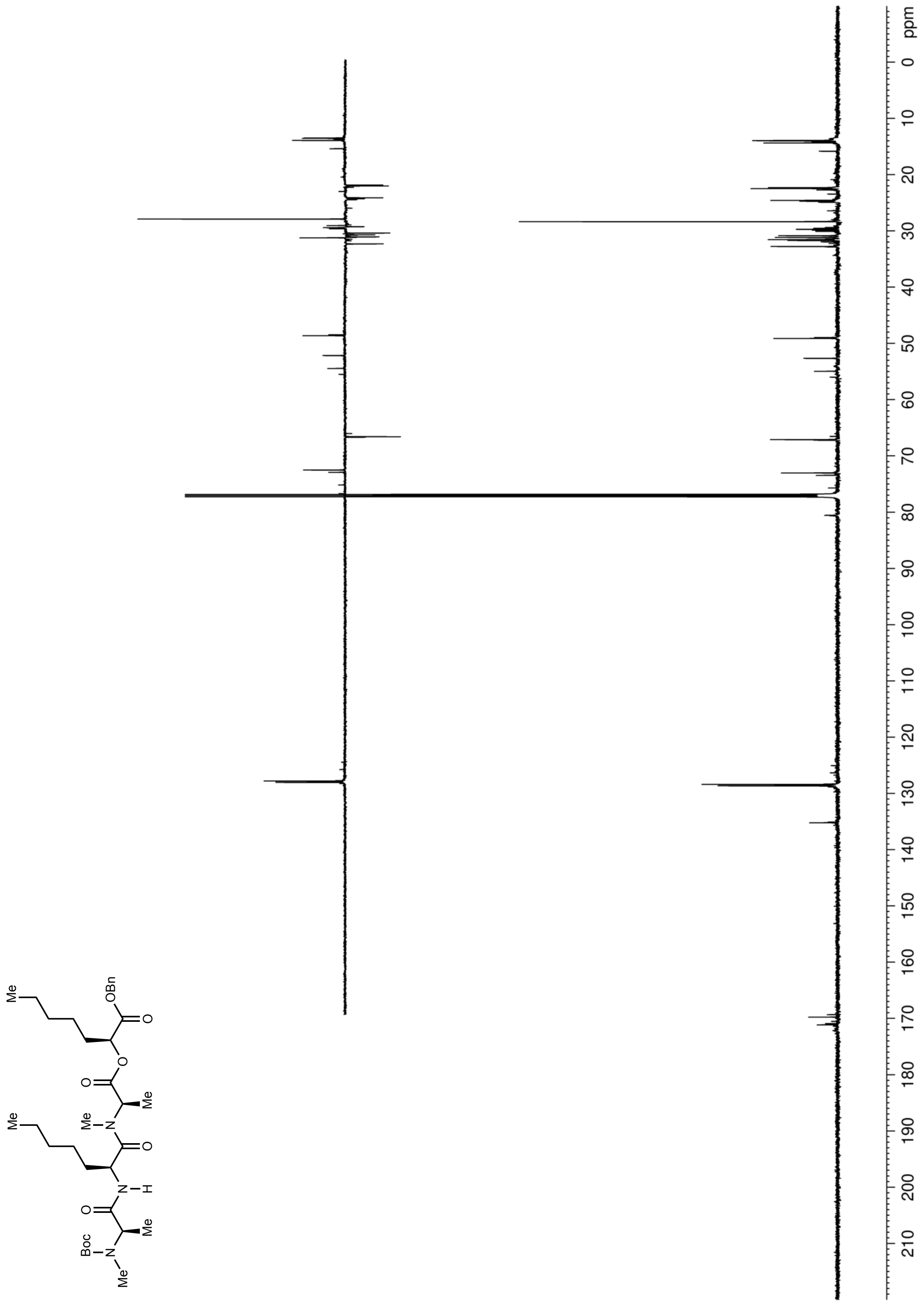

Figure S21. ^{1}H NMR (600 MHz, $CDCl_3$) of **T41**

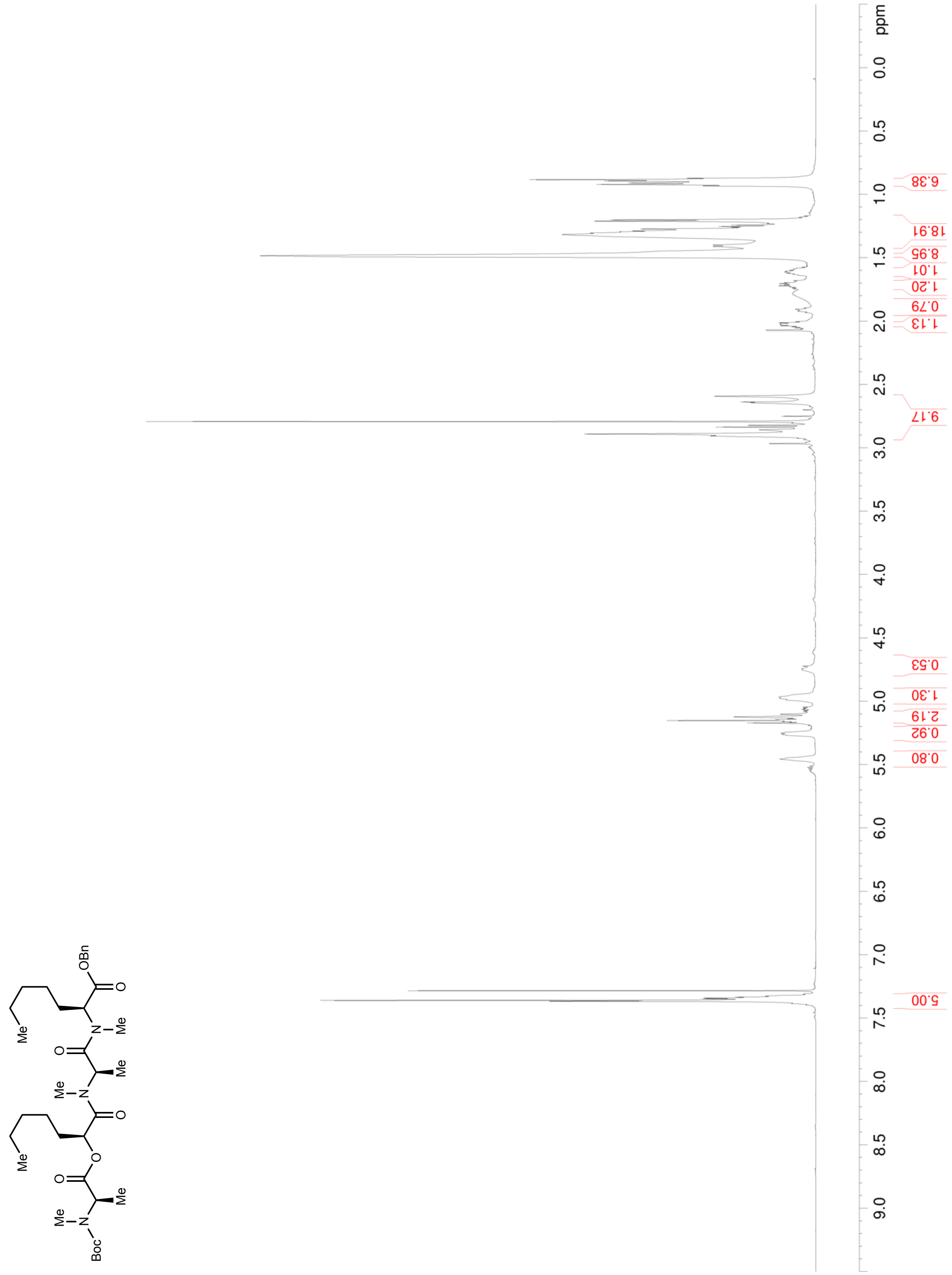

Figure S22. ^{13}C NMR (150 MHz, $CDCl_3$) of **T41**

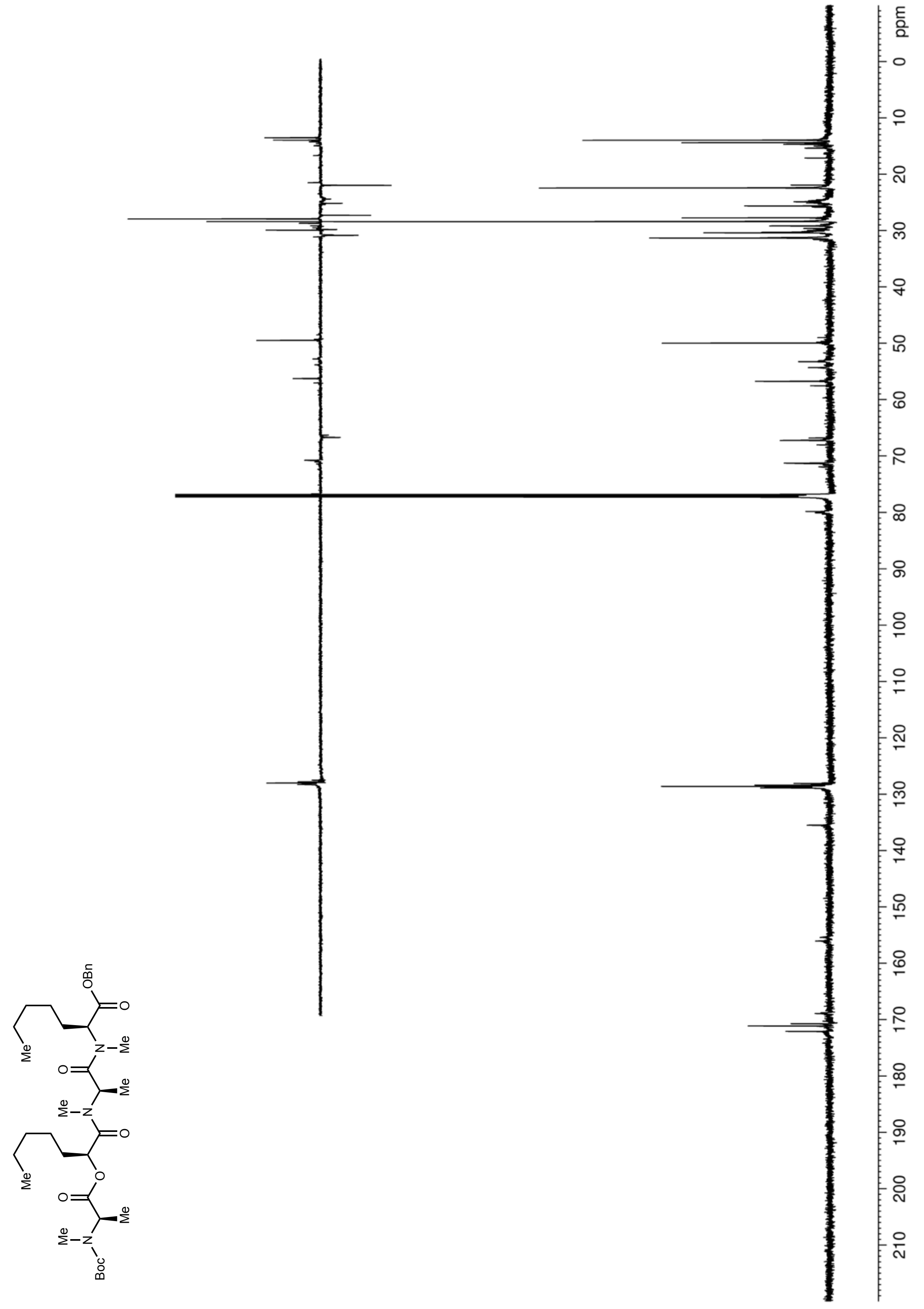

Figure S23. ^{1}H NMR (600 MHz, $CDCl_3$) of **T42**

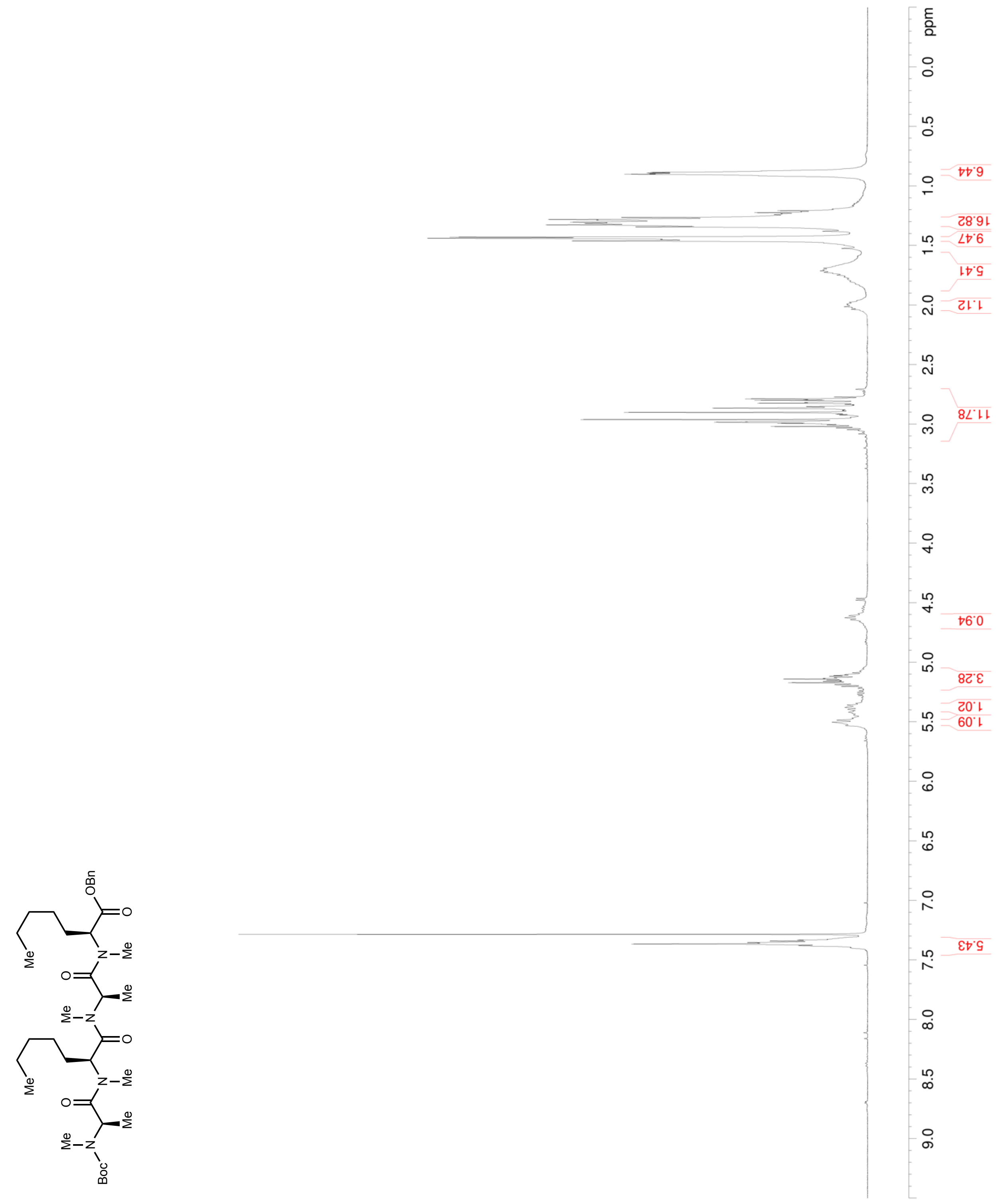

Figure S24. ^{13}C NMR (150 MHz, $CDCl_3$) of **T42**

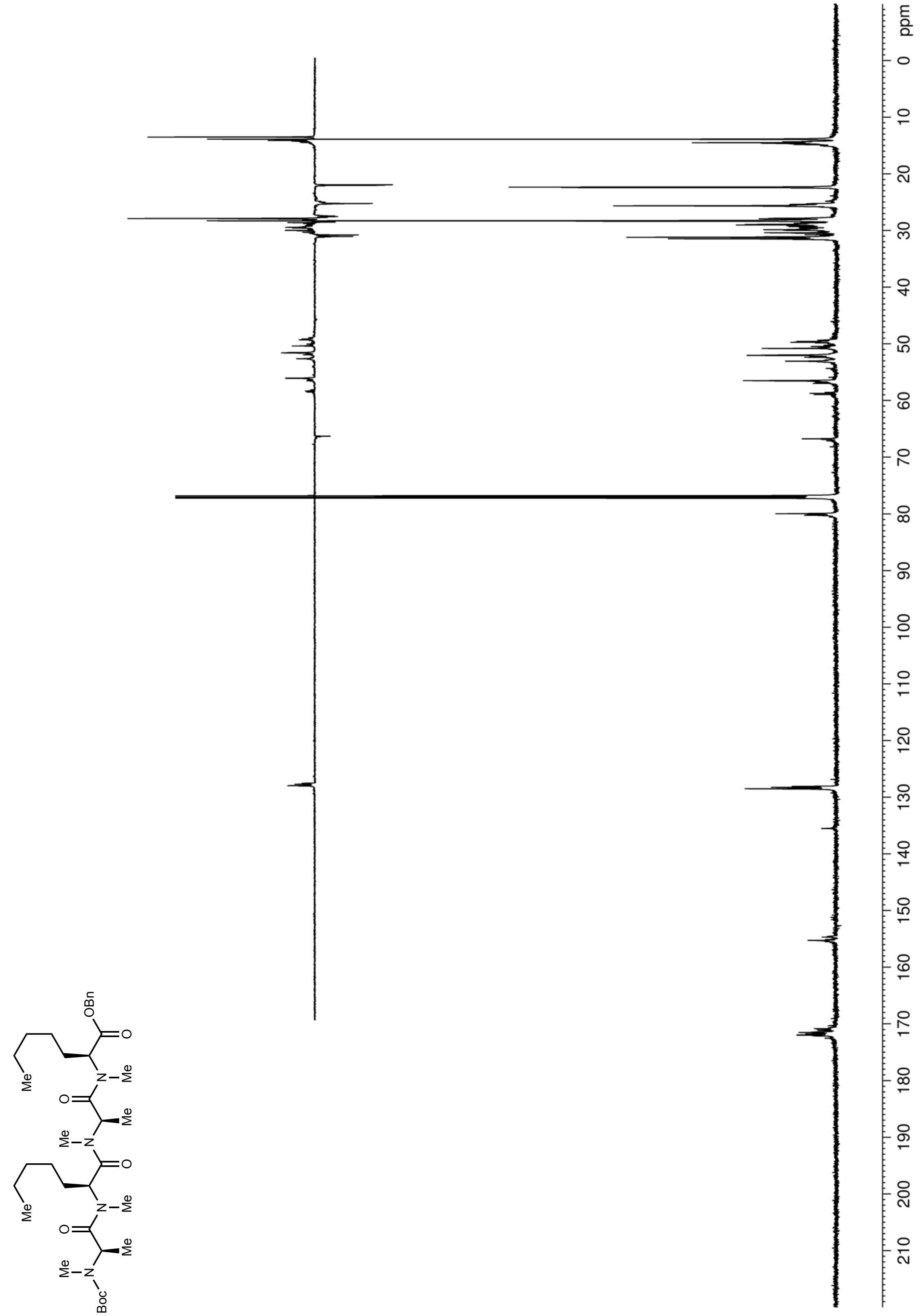

Figure S25. ^{1}H NMR (600 MHz, $CDCl_3$) of **T43**

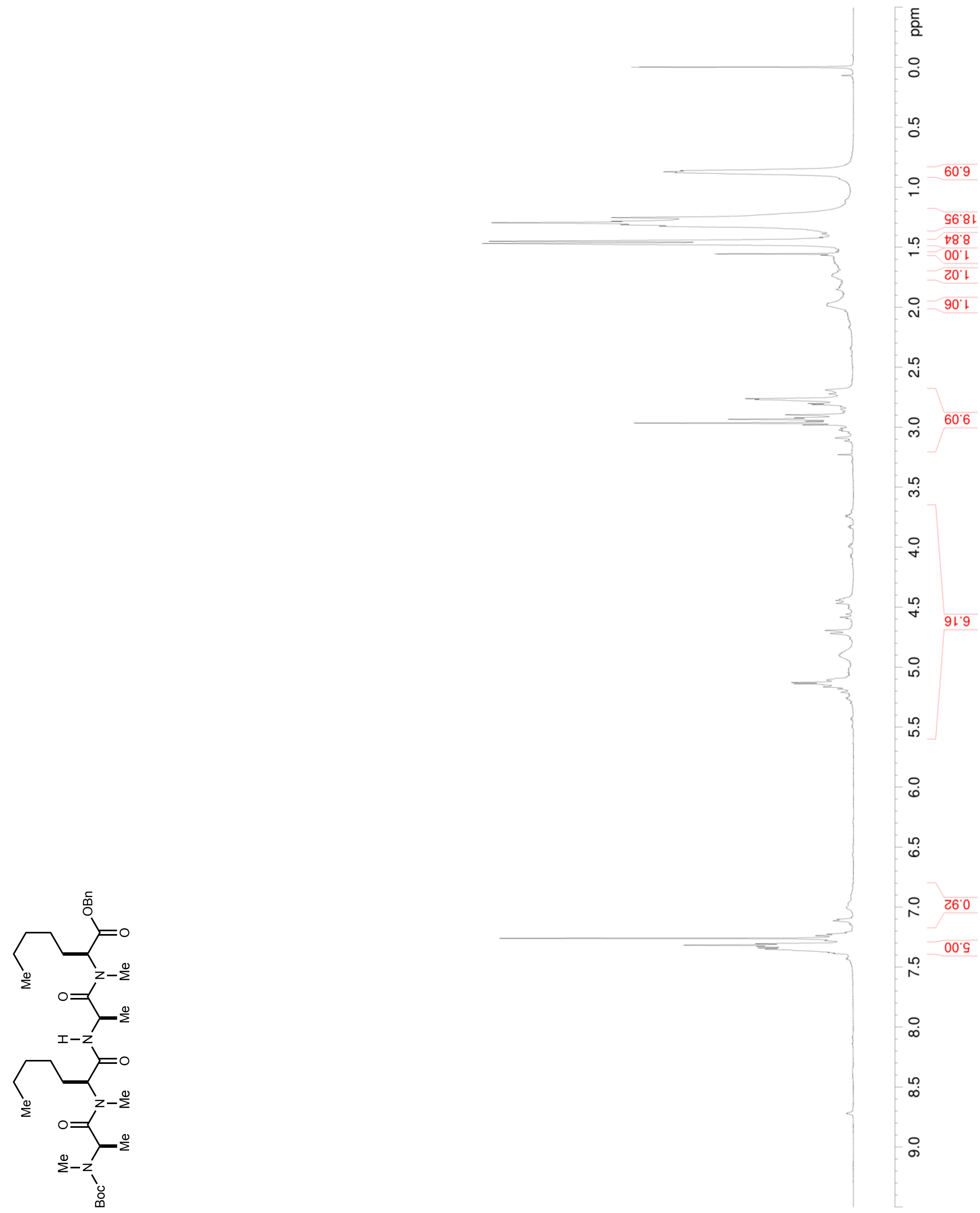

Figure S26. ^{13}C NMR (150 MHz, $CDCl_3$) of **T43**

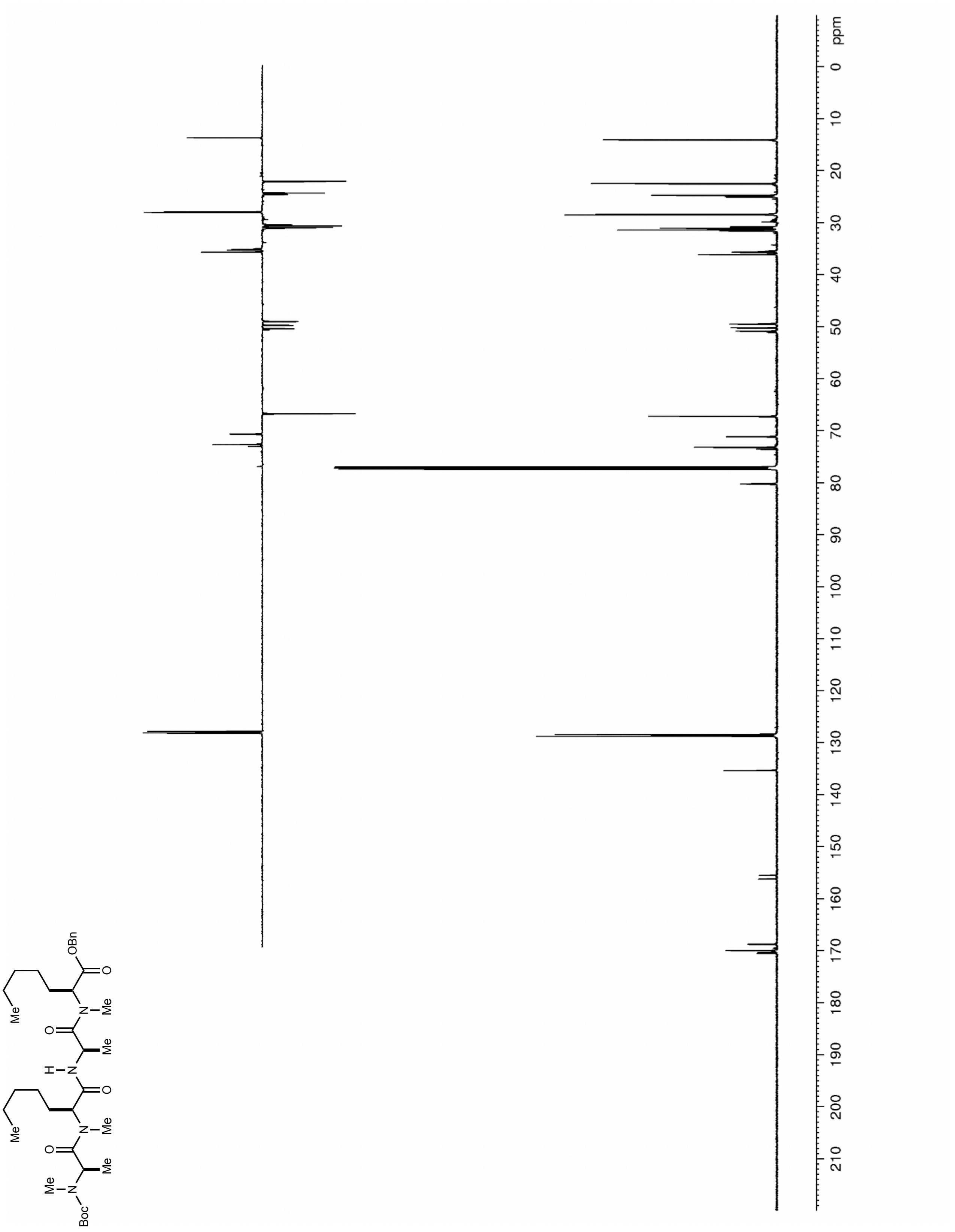

Figure S27. ^{1}H NMR (600 MHz, $CDCl_3$) of **T44**

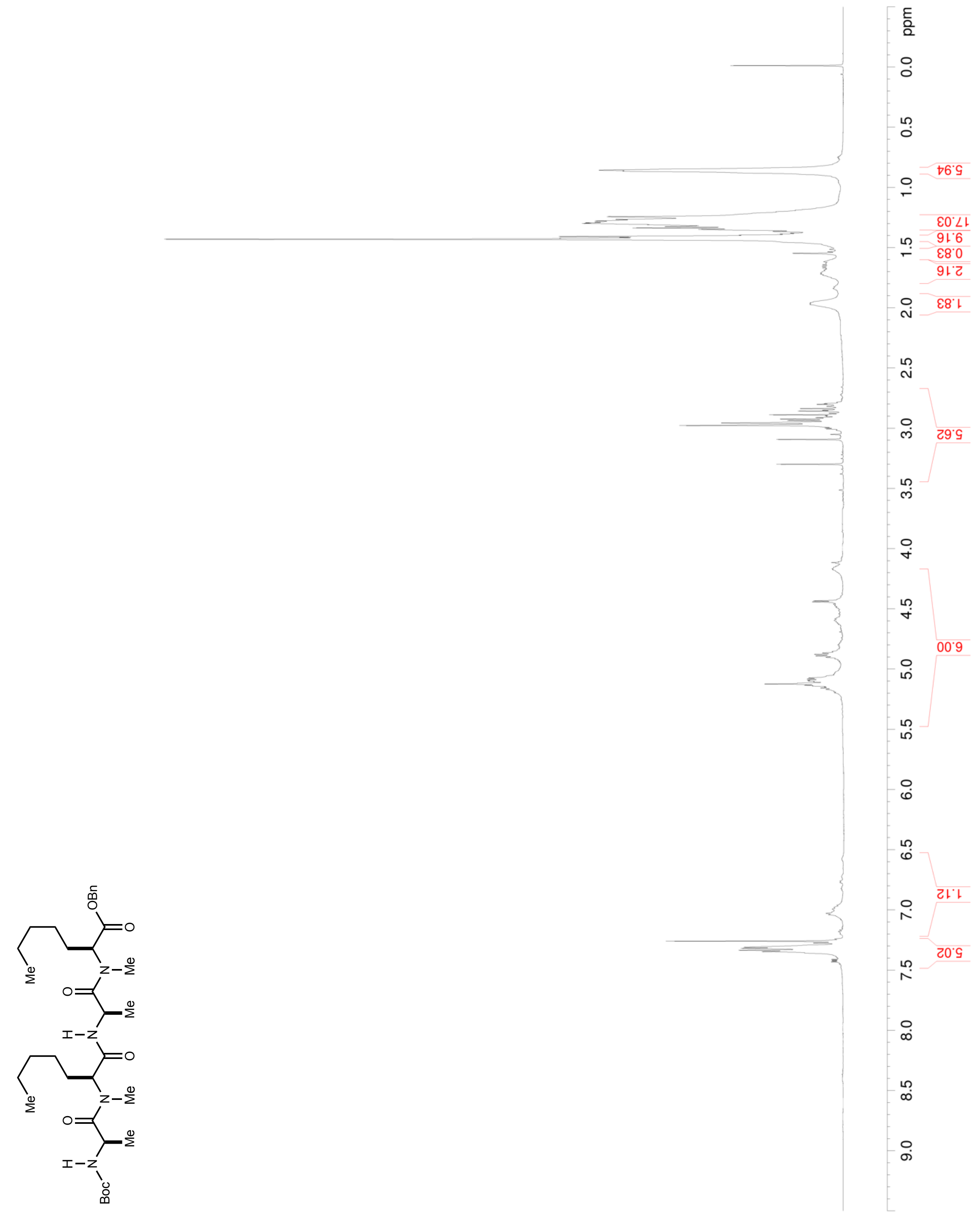

Figure S28. ^{13}C NMR (150 MHz, $CDCl_3$) of **T44**

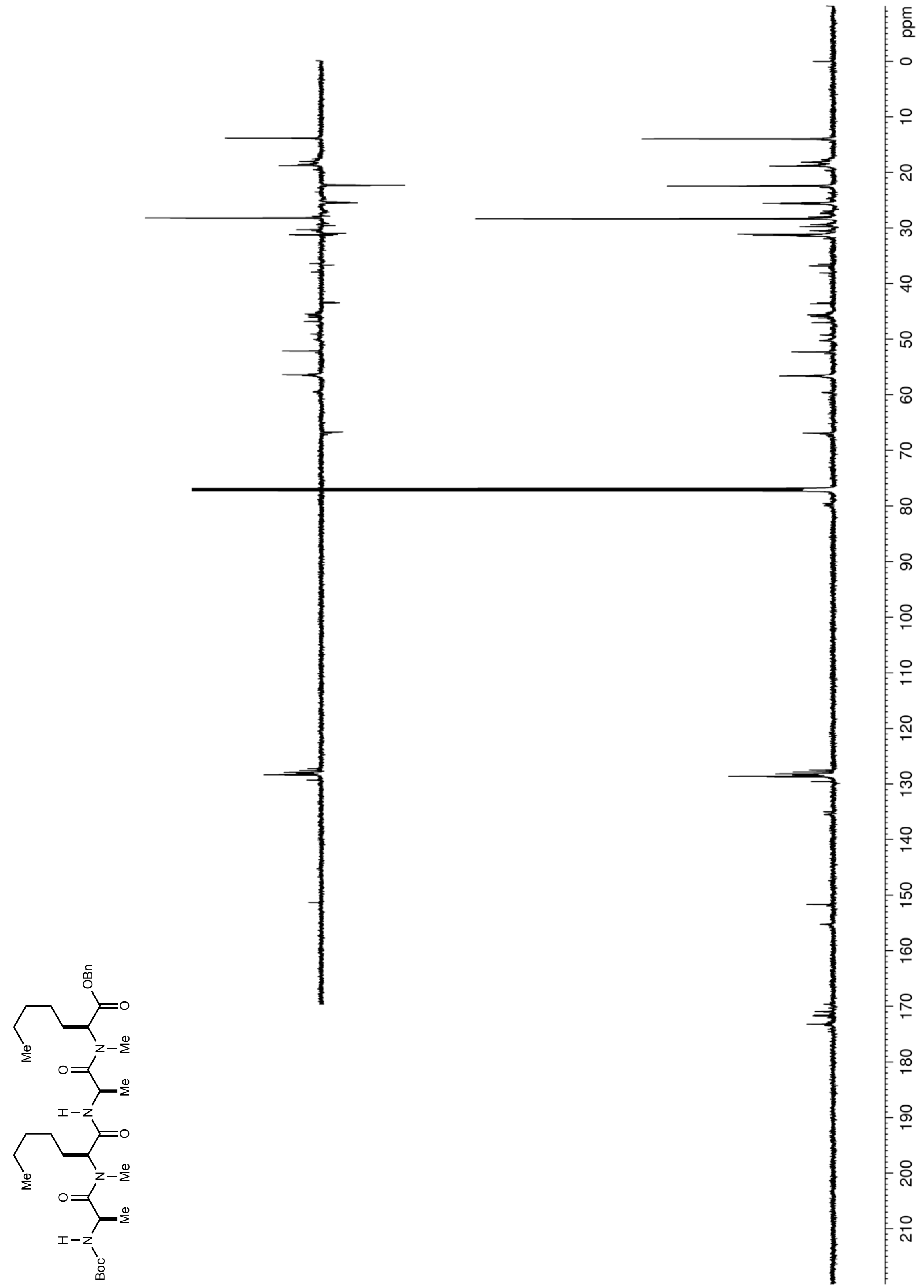

Figure S29. ^{1}H NMR (600 MHz, $CDCl_3$) of **T45**

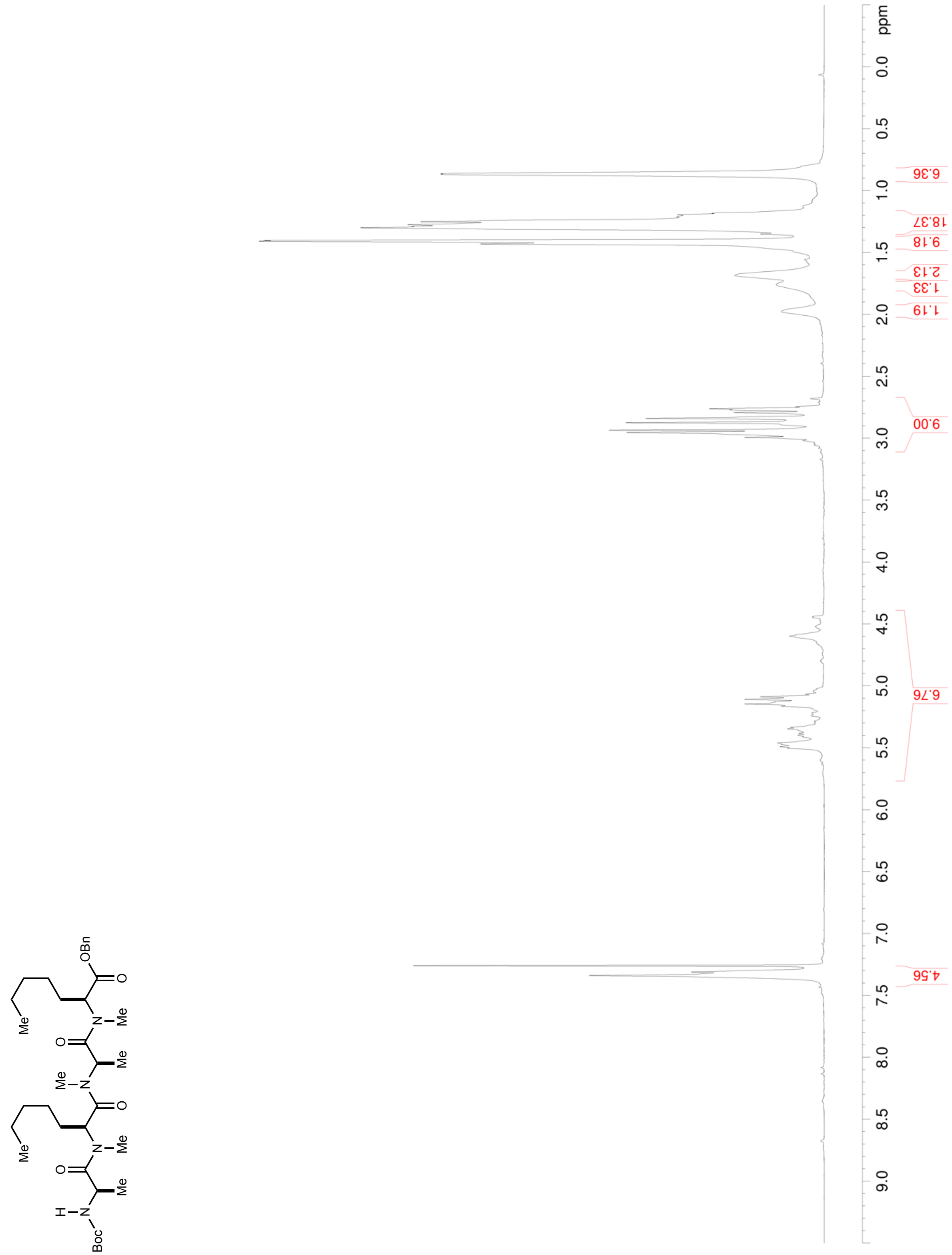

Figure S30. ^{13}C NMR (150 MHz, $CDCl_3$) of **T45**

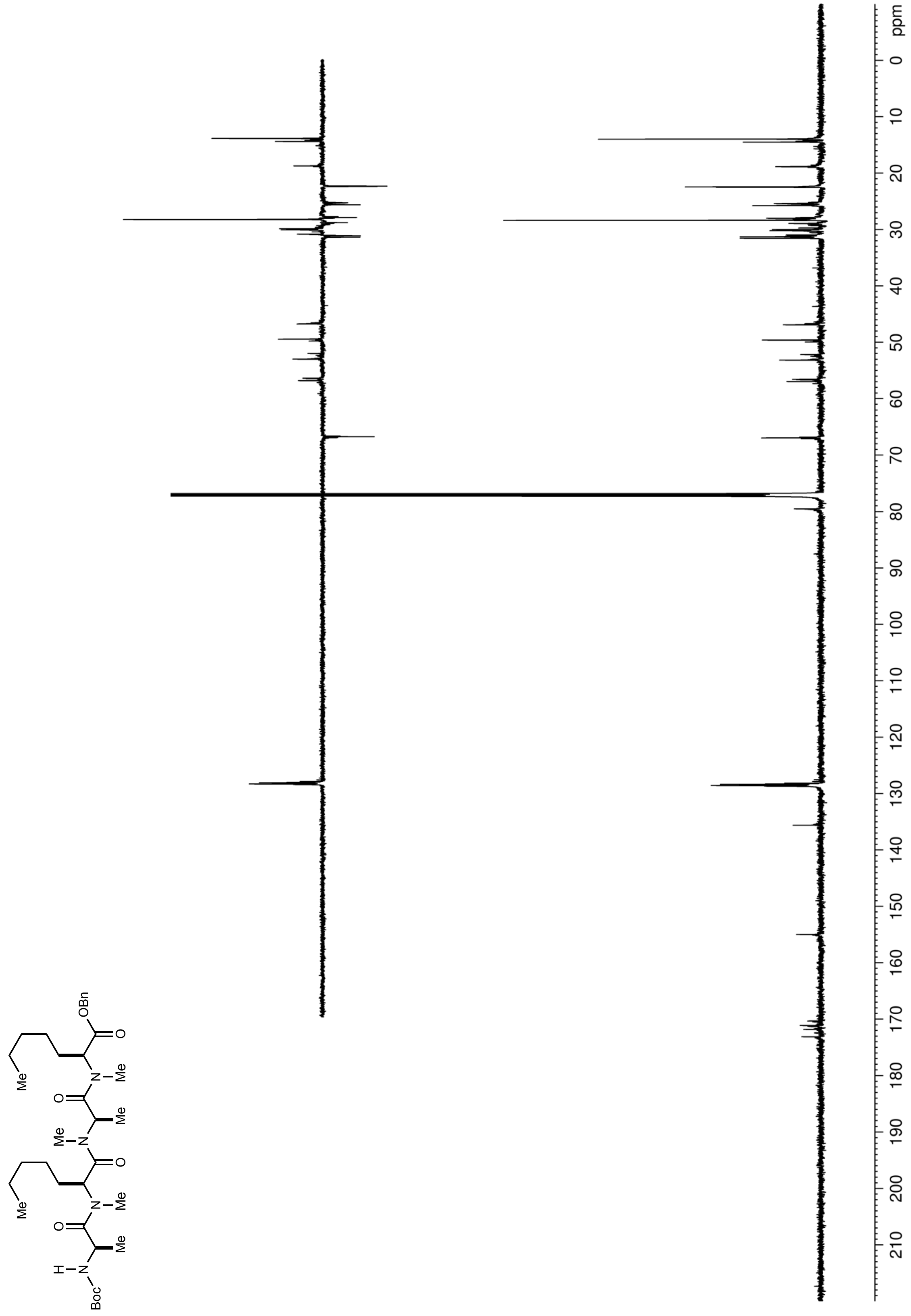

Figure S31. ^{1}H NMR (600 MHz, $CDCl_3$) of **T46**

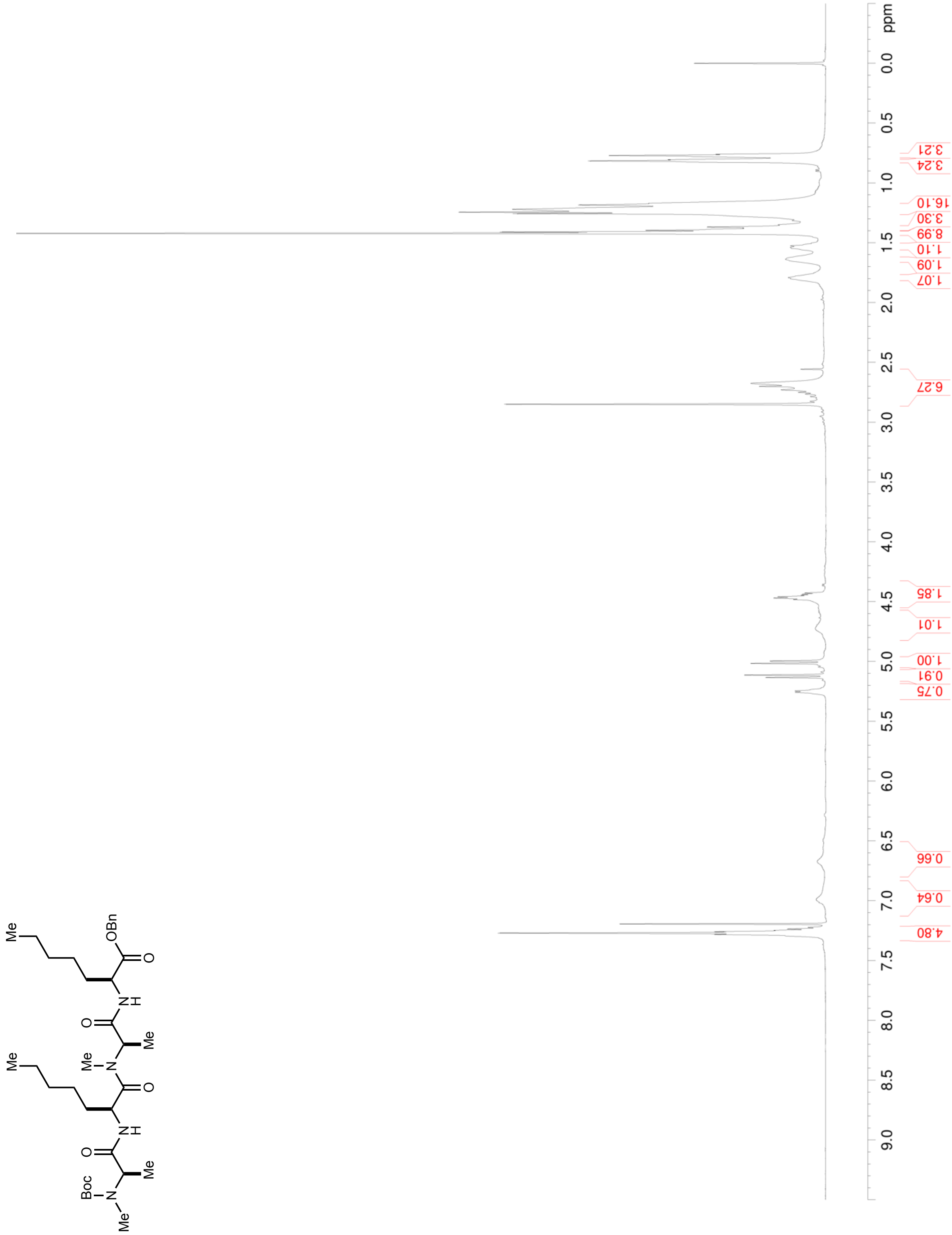

Figure S32. ^{13}C NMR (150 MHz, $CDCl_3$) of **T46**

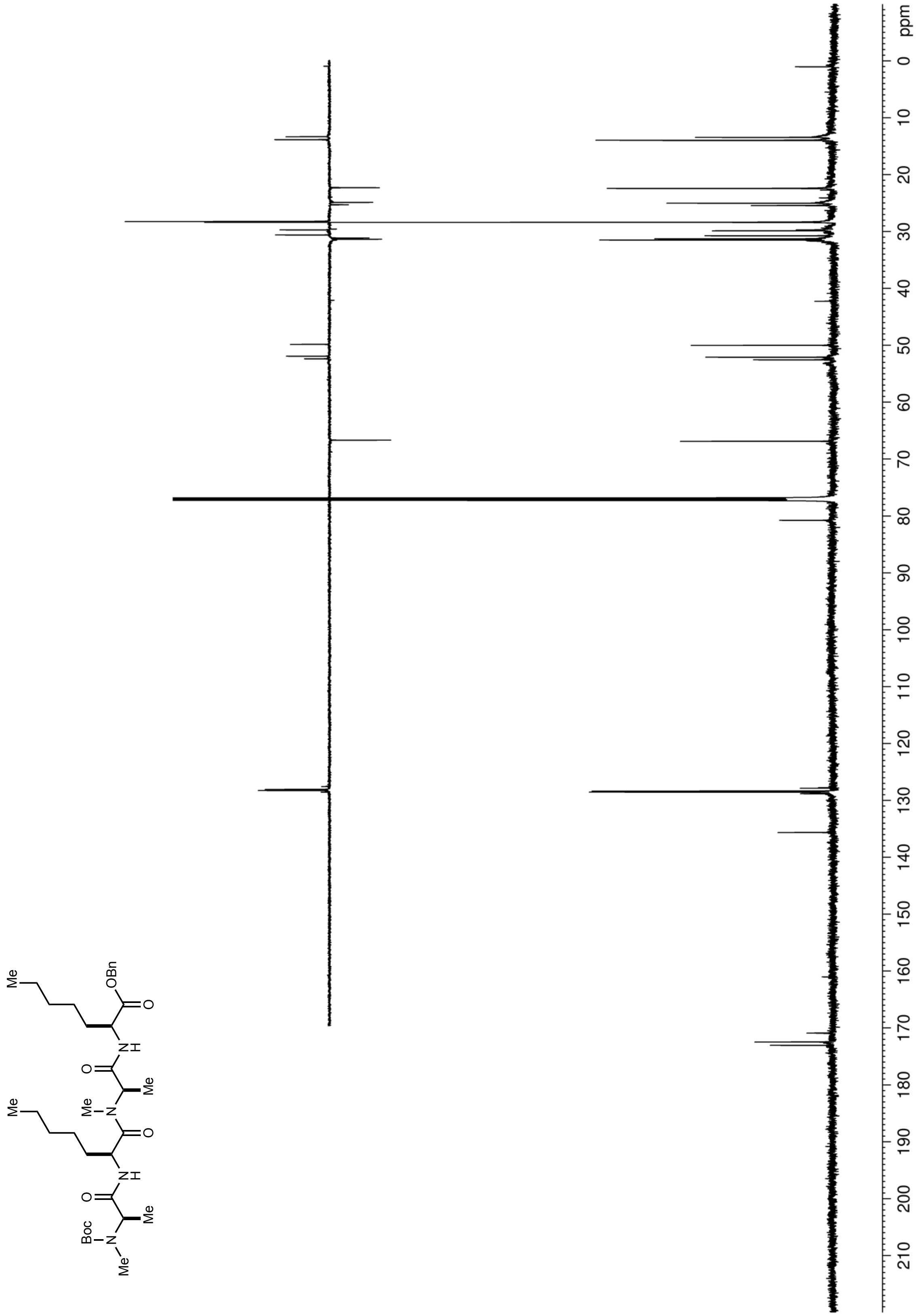

Figure S33. ^{1}H NMR (600 MHz, $CDCl_3$) of **T47**

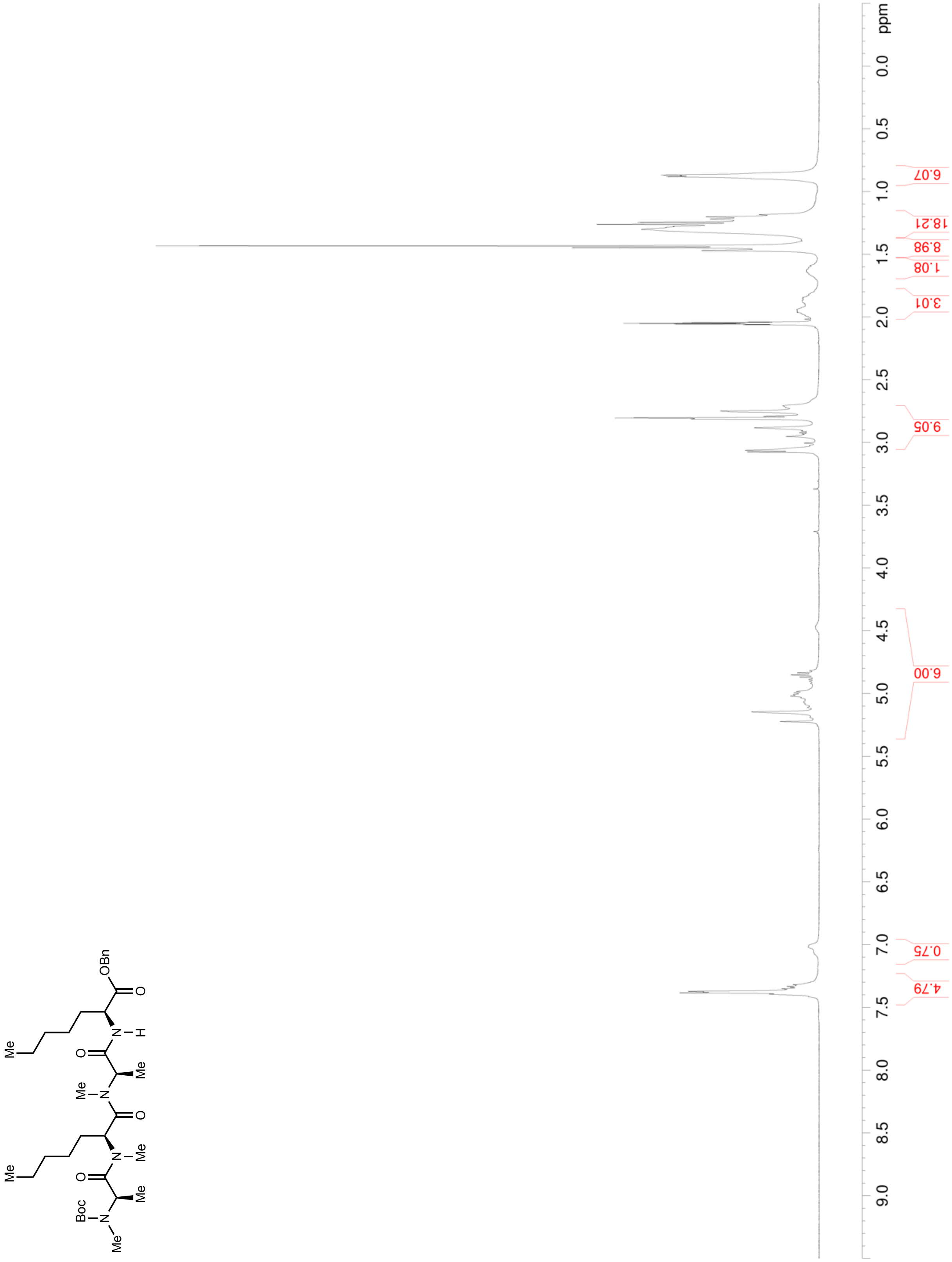

Figure S34. ^{13}C NMR (150 MHz, $CDCl_3$) of **T47**

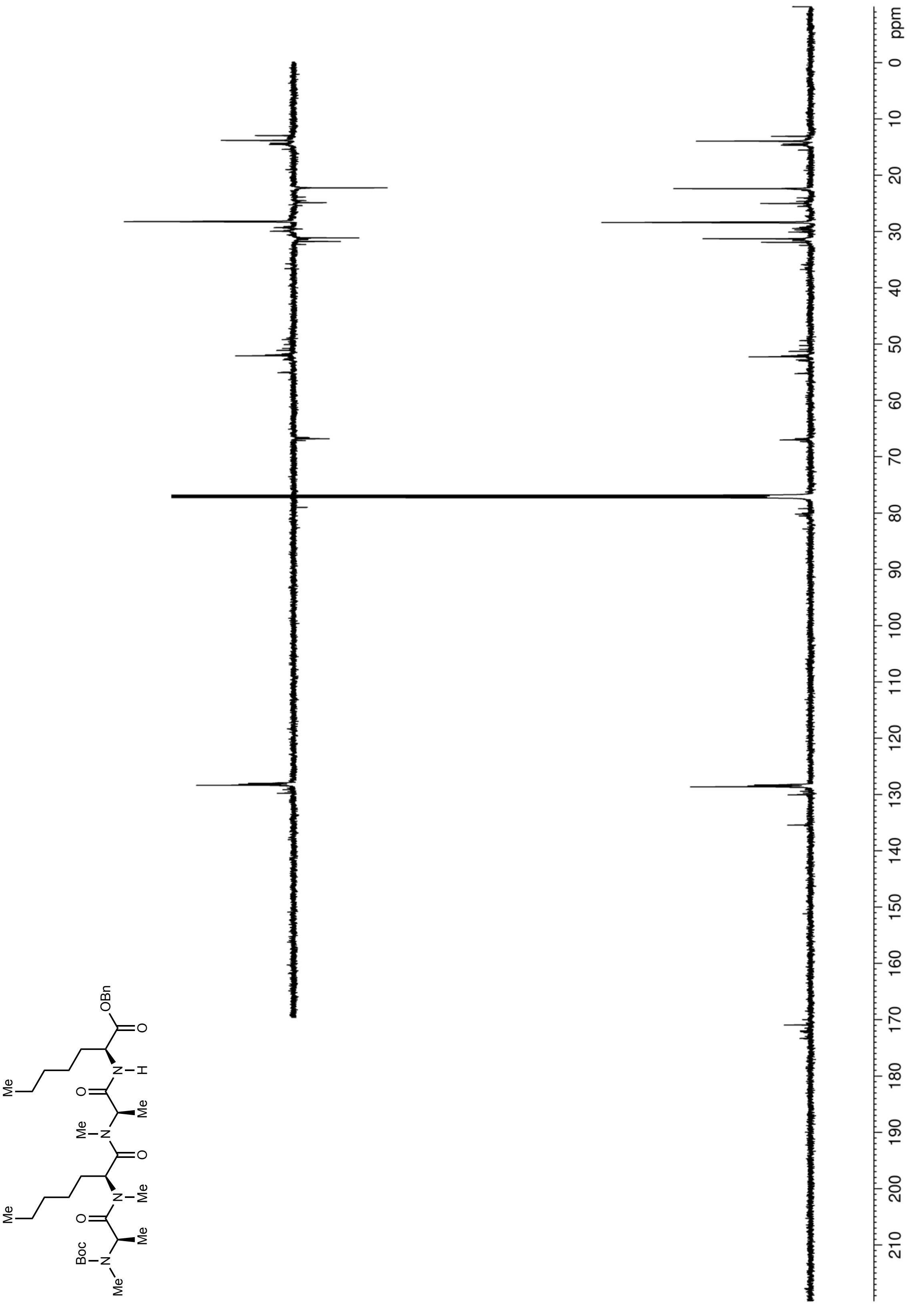

Figure S35. ^{1}H NMR (600 MHz, $CDCl_3$) of **O49**

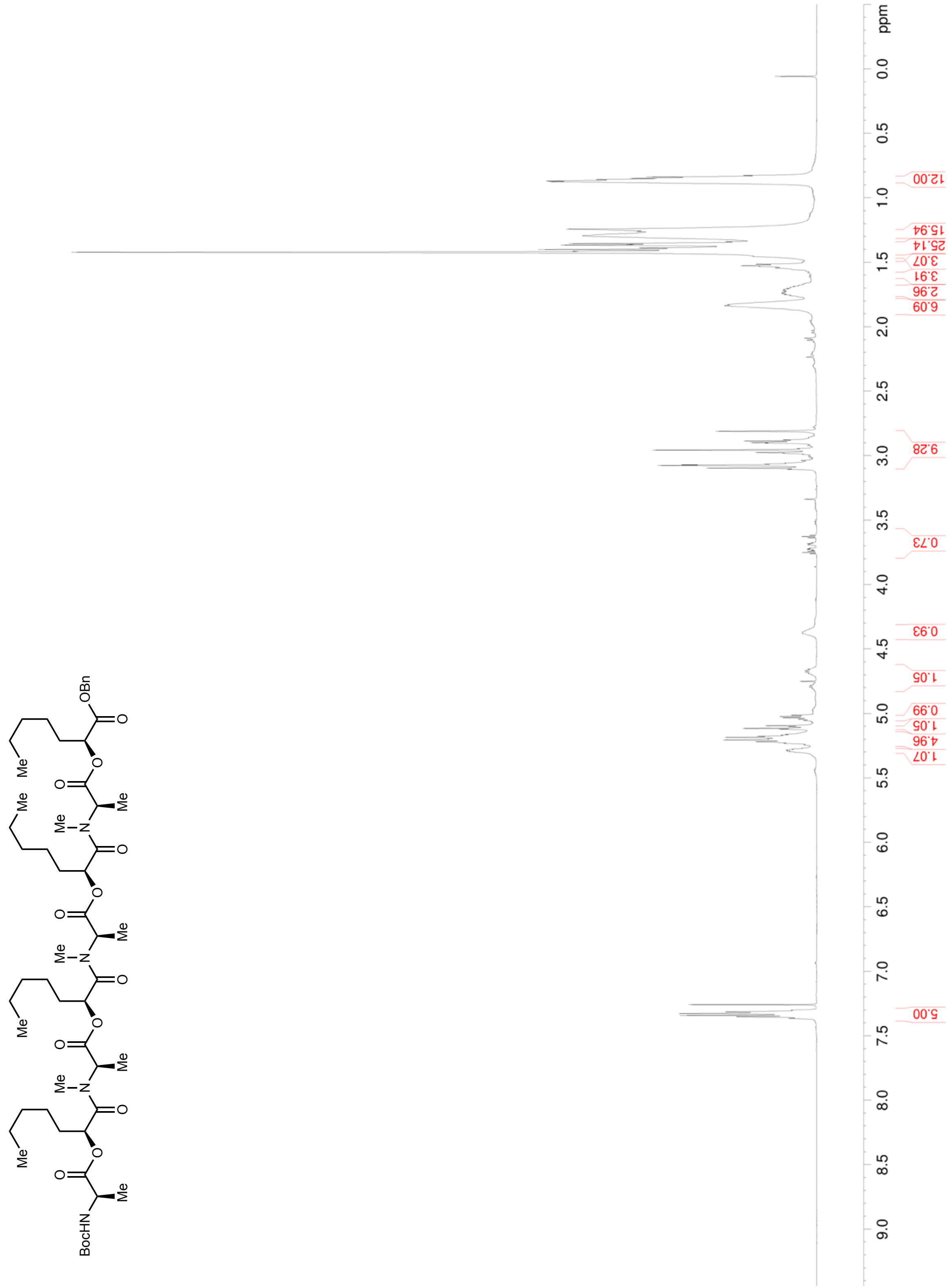

Figure S36. ^{13}C NMR (150 MHz, $CDCl_3$) of **O49**

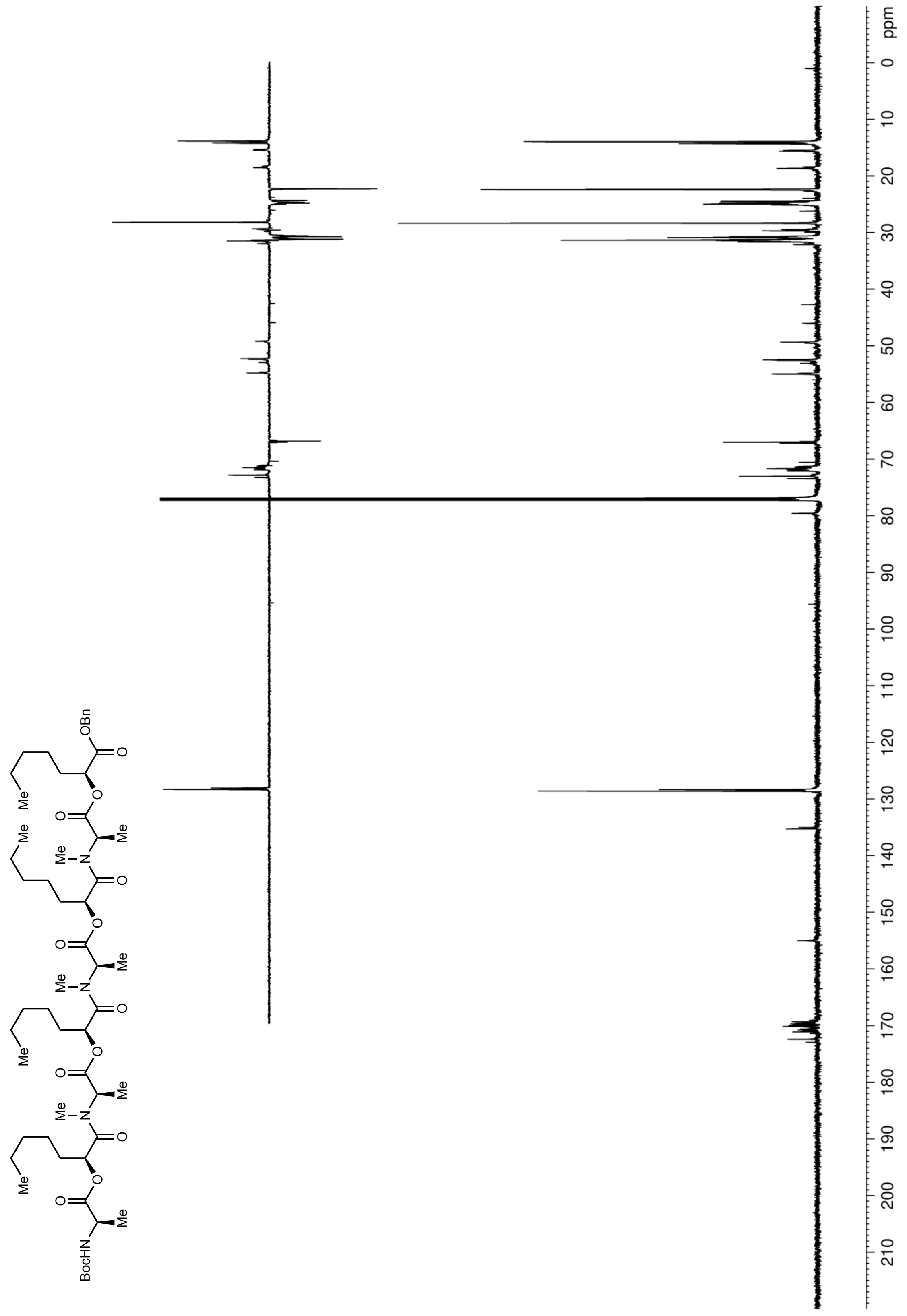

Figure S37. ^{1}H NMR (600 MHz, $CDCl_3$) of **O50**

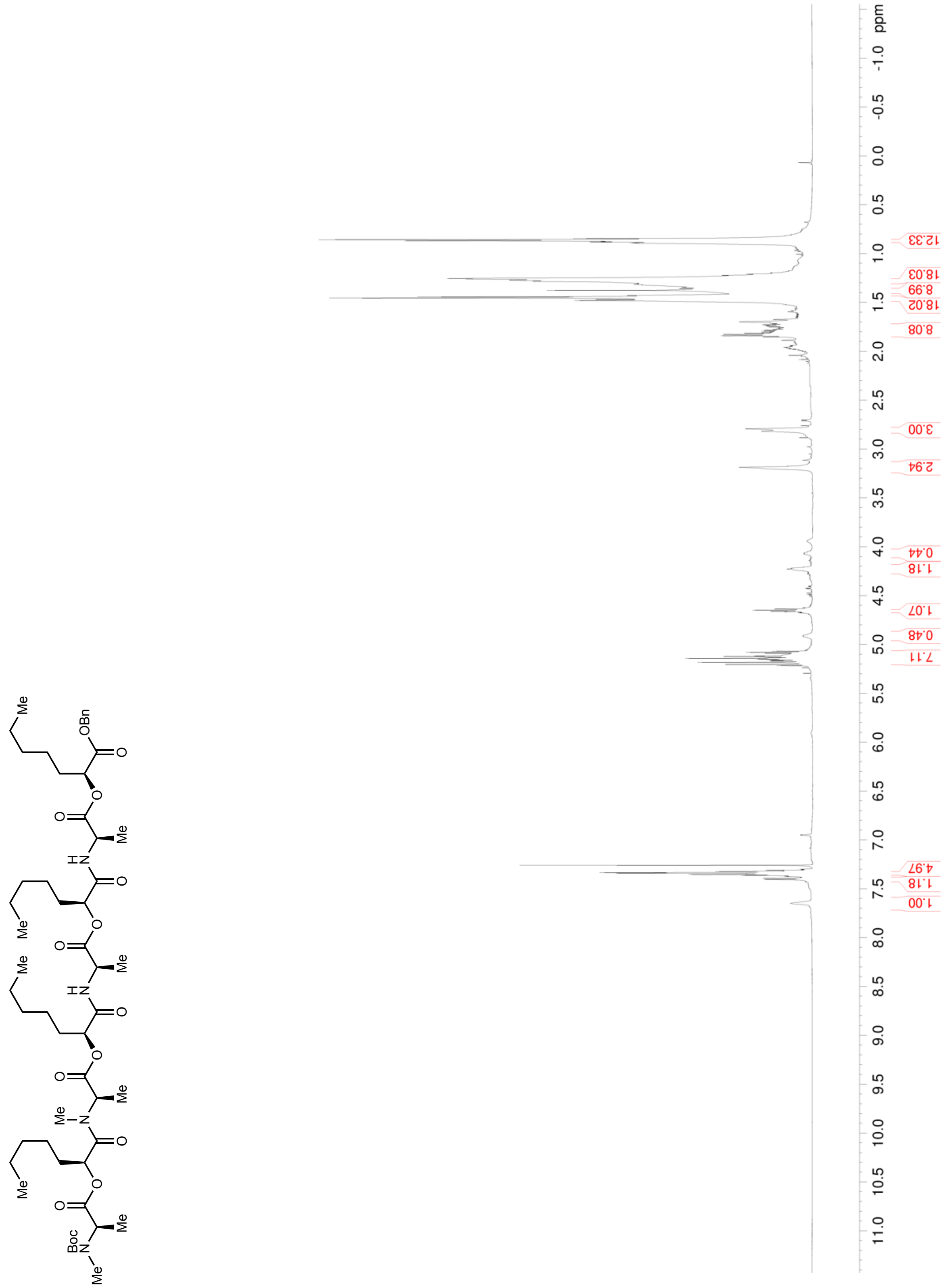

Figure S38. ^{13}C NMR (150 MHz, $CDCl_3$) of **O50**

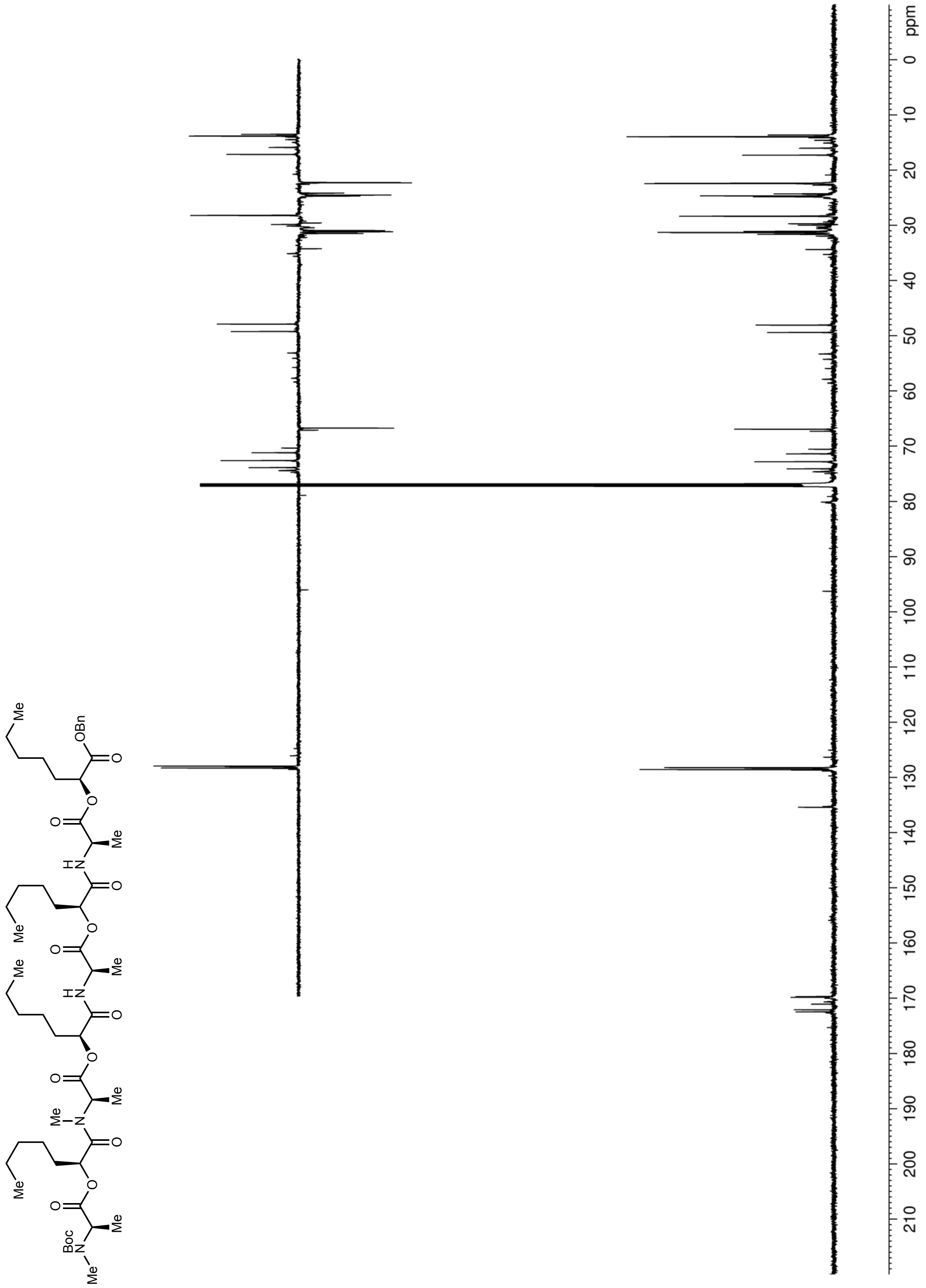

Figure S39. ^{1}H NMR (600 MHz, $CDCl_3$) of **H51**

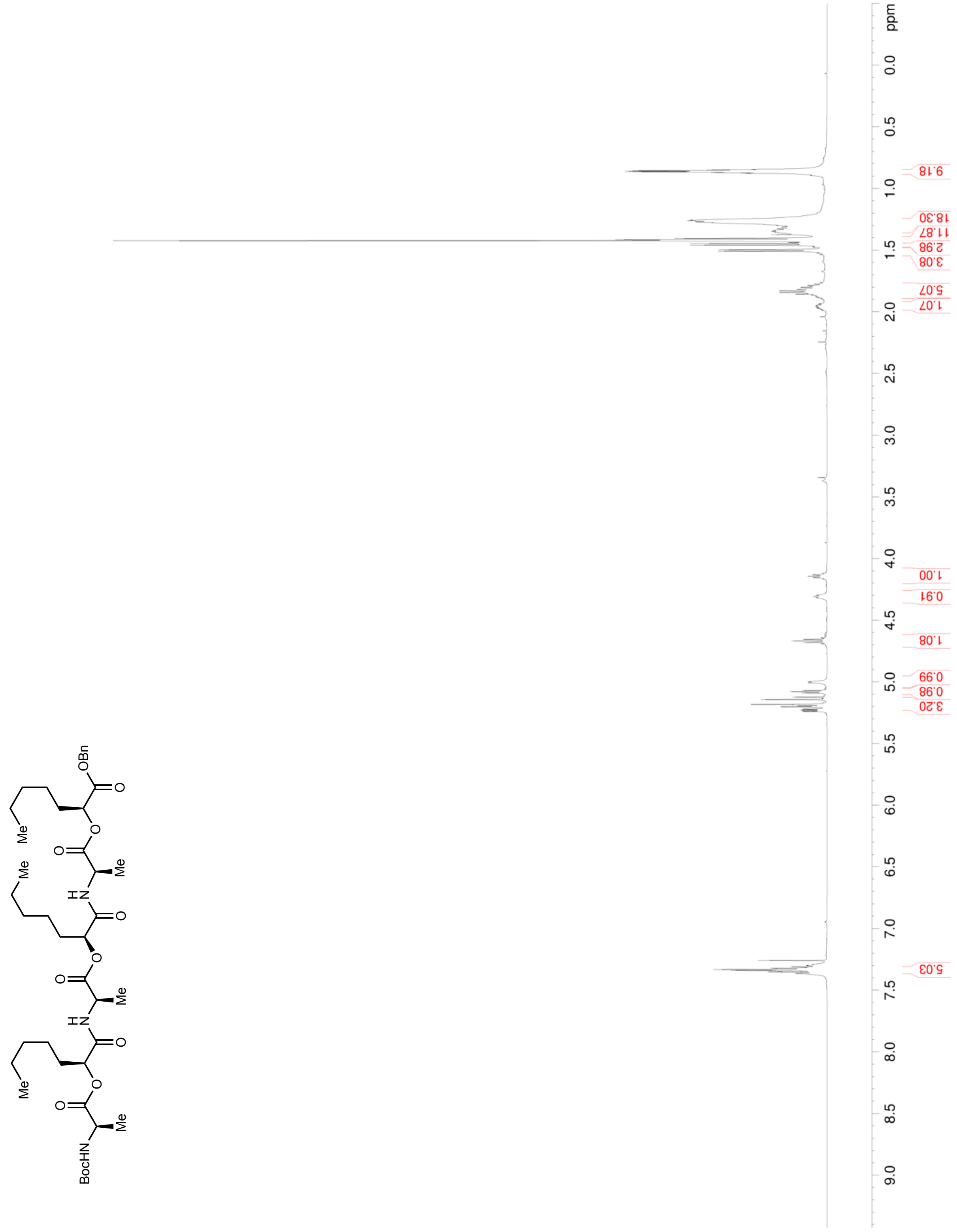

Figure S40. ^{13}C NMR (150 MHz, $CDCl_3$) of **H51**

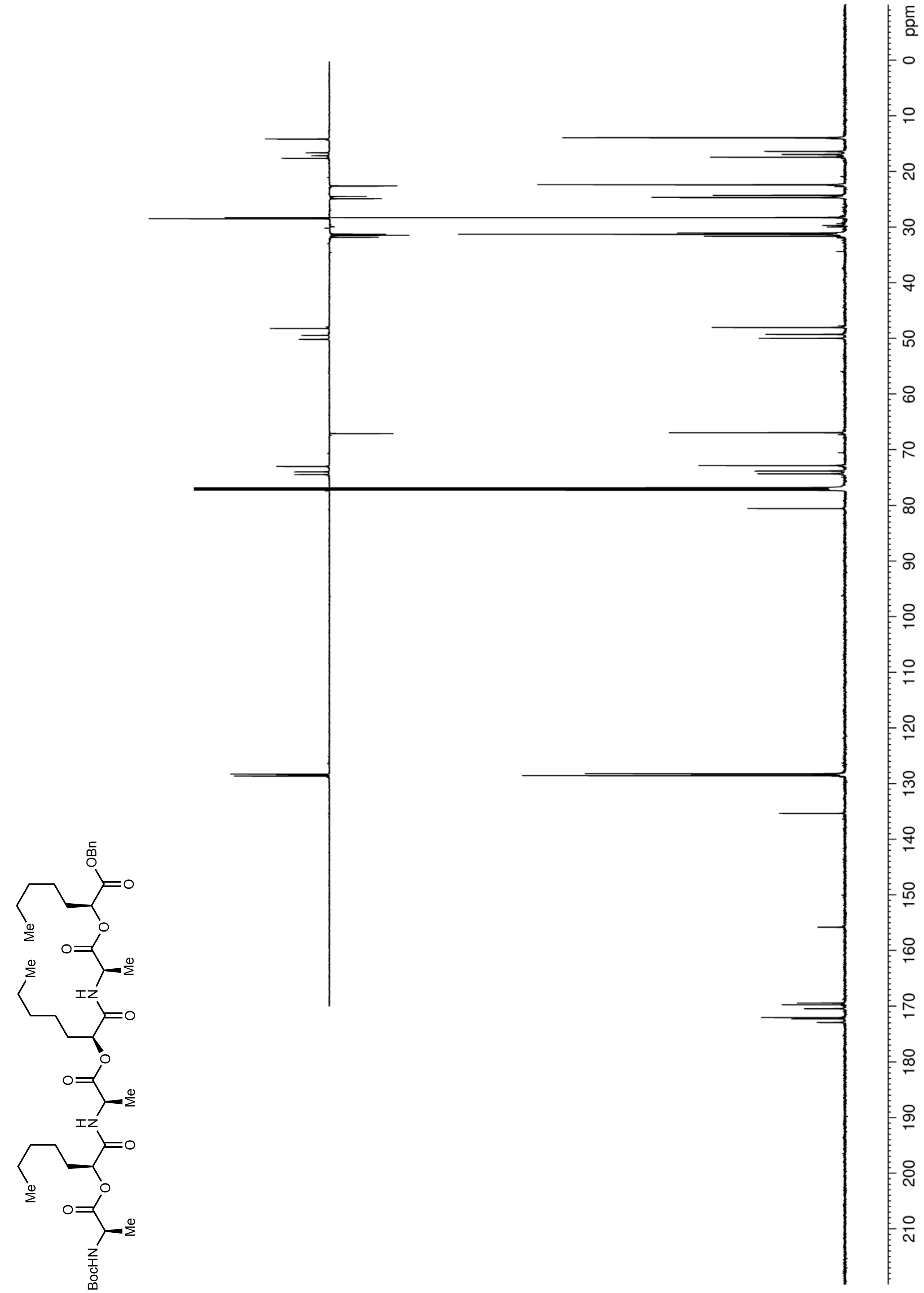

Figure S41. ^{1}H NMR (600 MHz, $CDCl_3$) of **O52**

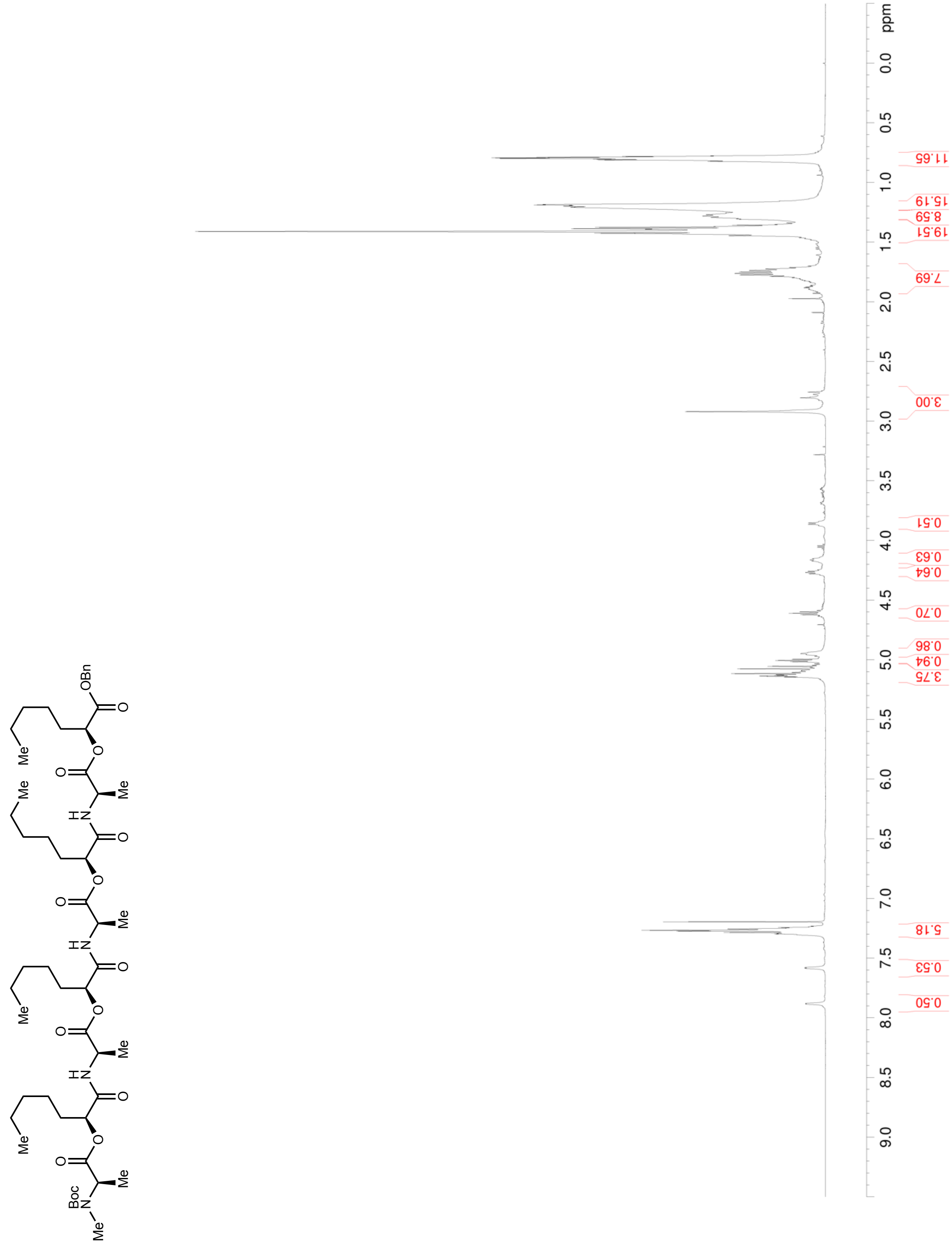

Figure S42. ^{13}C NMR (150 MHz, $CDCl_3$) of **O52**

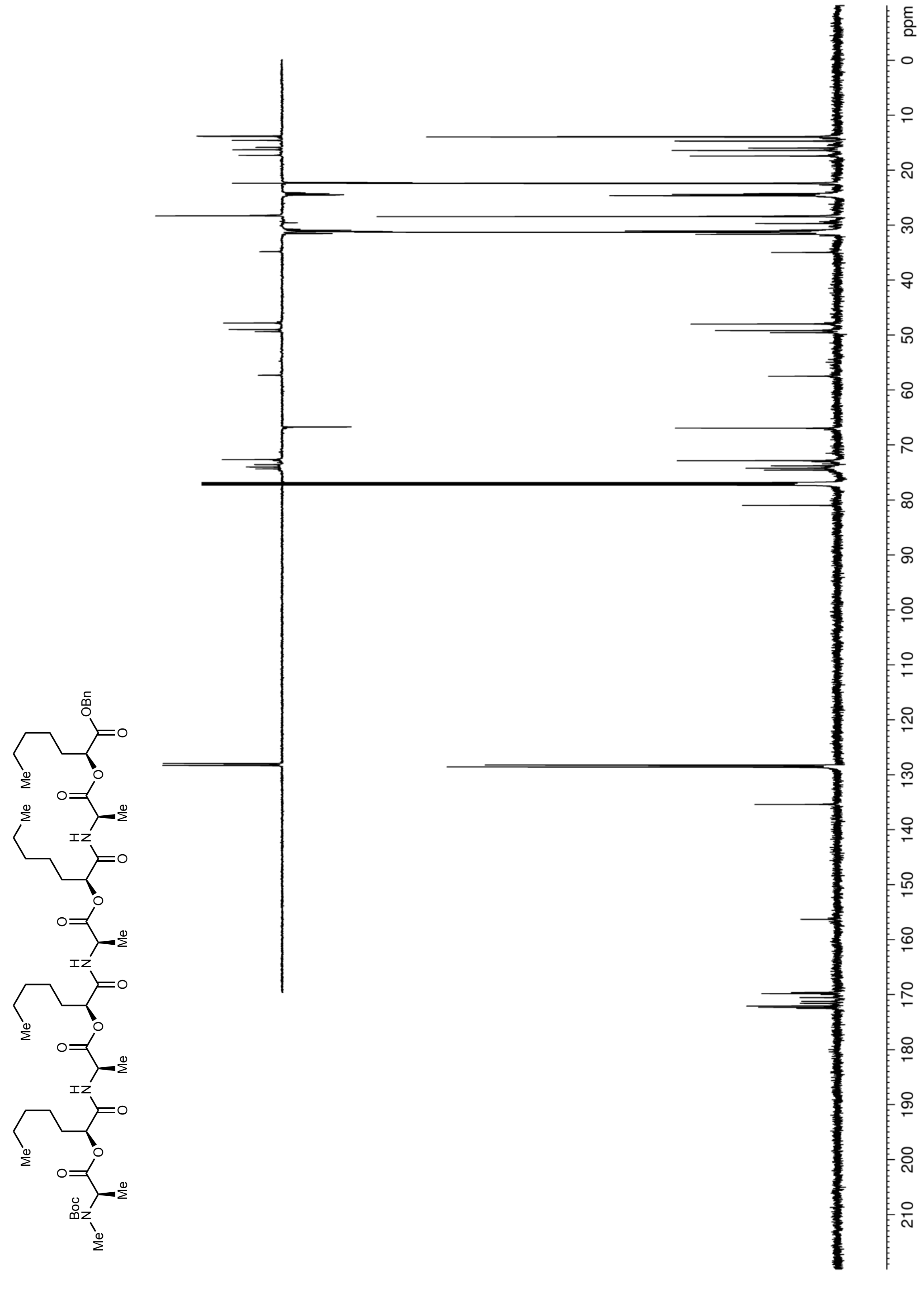

Figure S43. ^{1}H NMR (600 MHz, $CDCl_3$) of **O54**

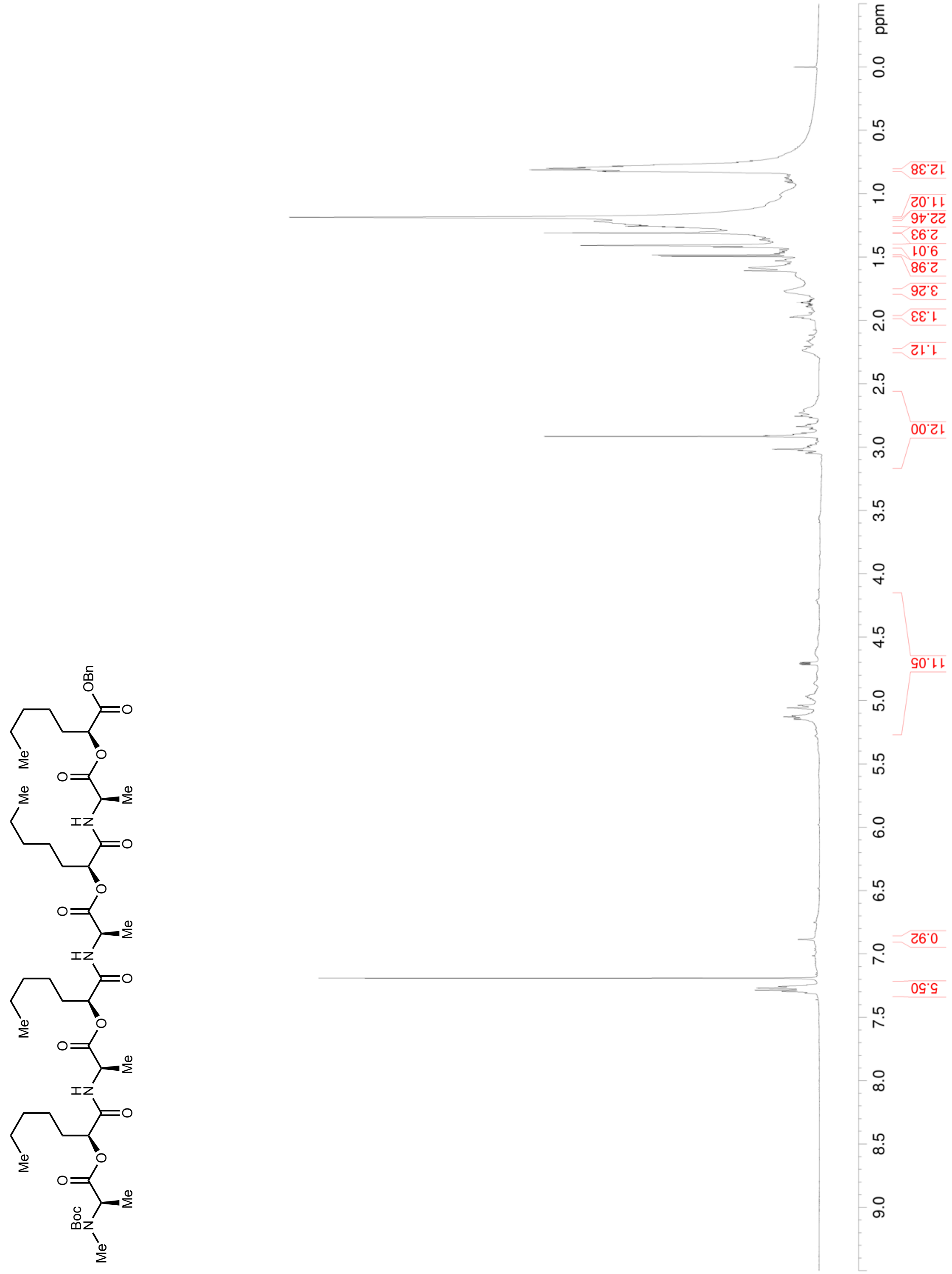

Figure S44. ^{13}C NMR (150 MHz, $CDCl_3$) of **O54**

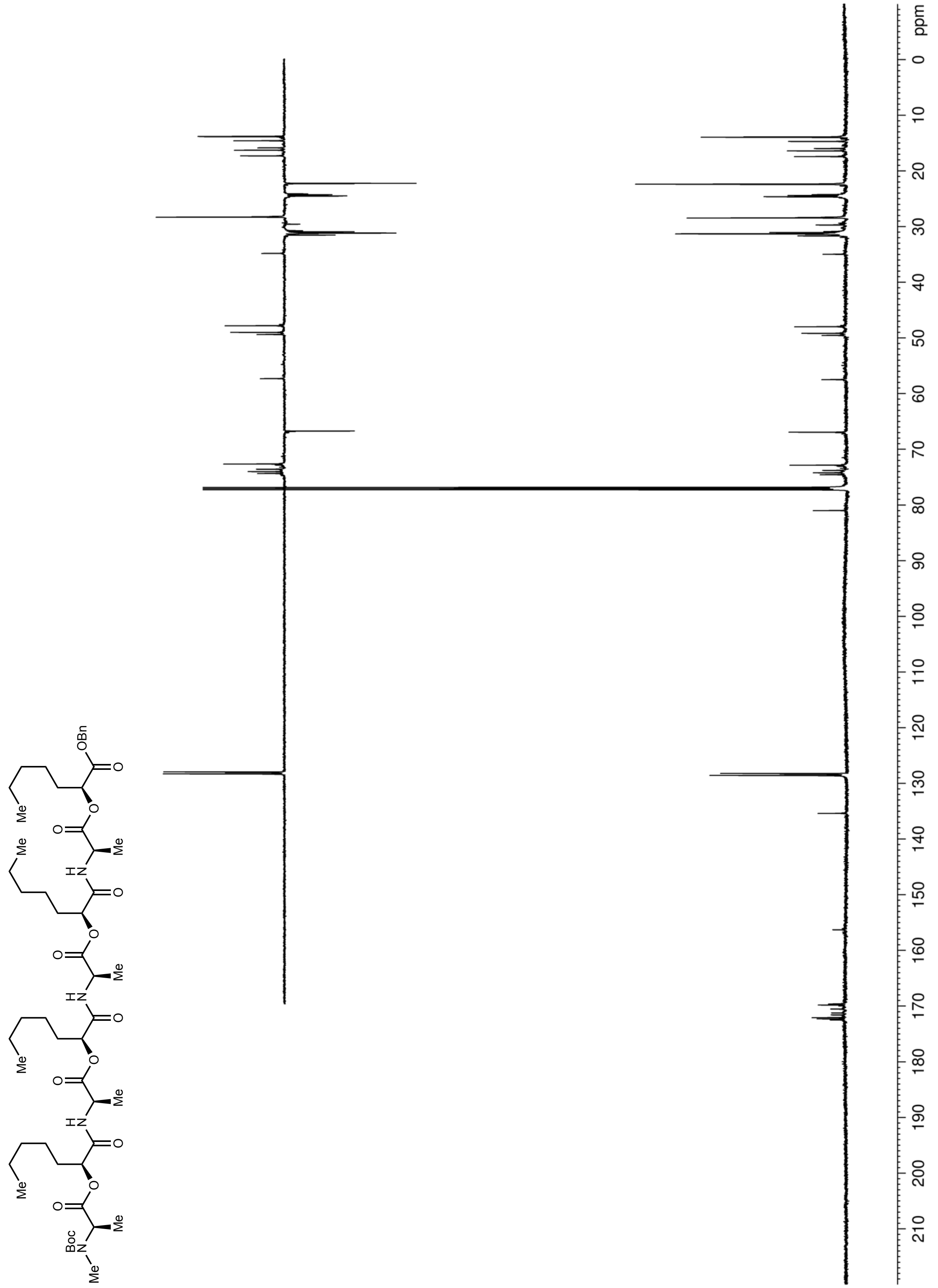

Figure S45. ^{1}H NMR (600 MHz, $CDCl_3$) of **O55**

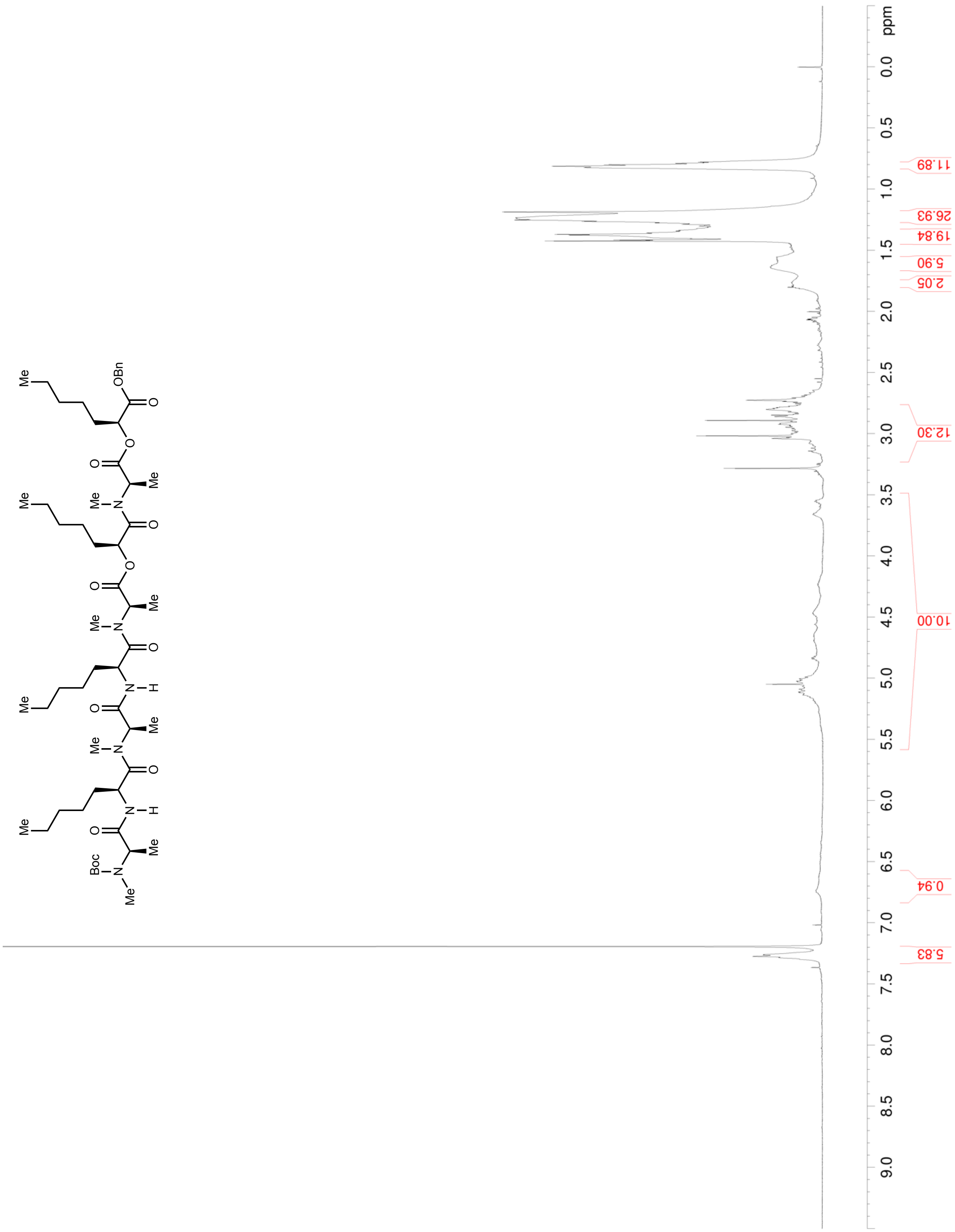

Figure S46. ^{13}C NMR (150 MHz, $CDCl_3$) of **O55**

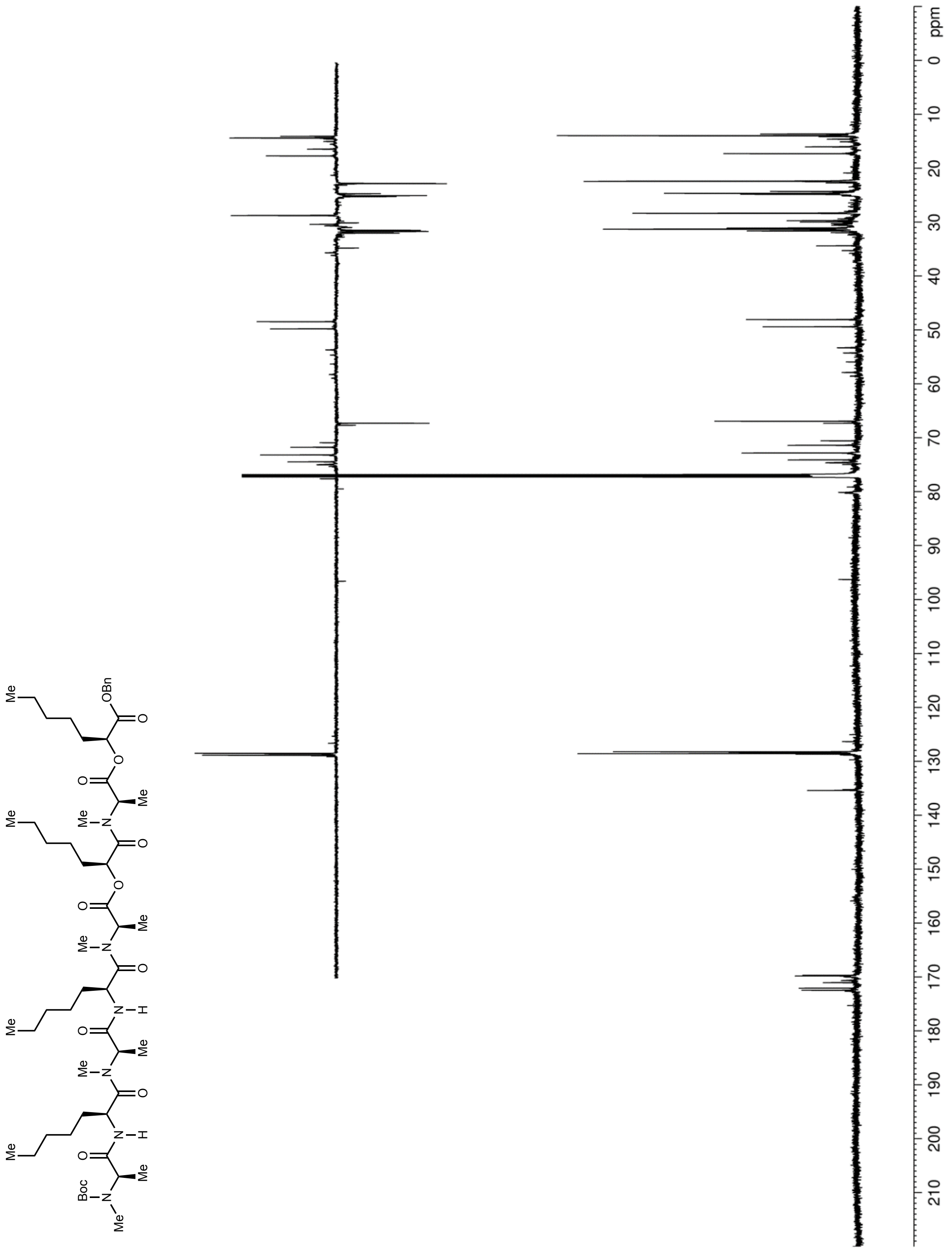

Figure S47. ^{1}H NMR (600 MHz, $CDCl_3$) of **O56**

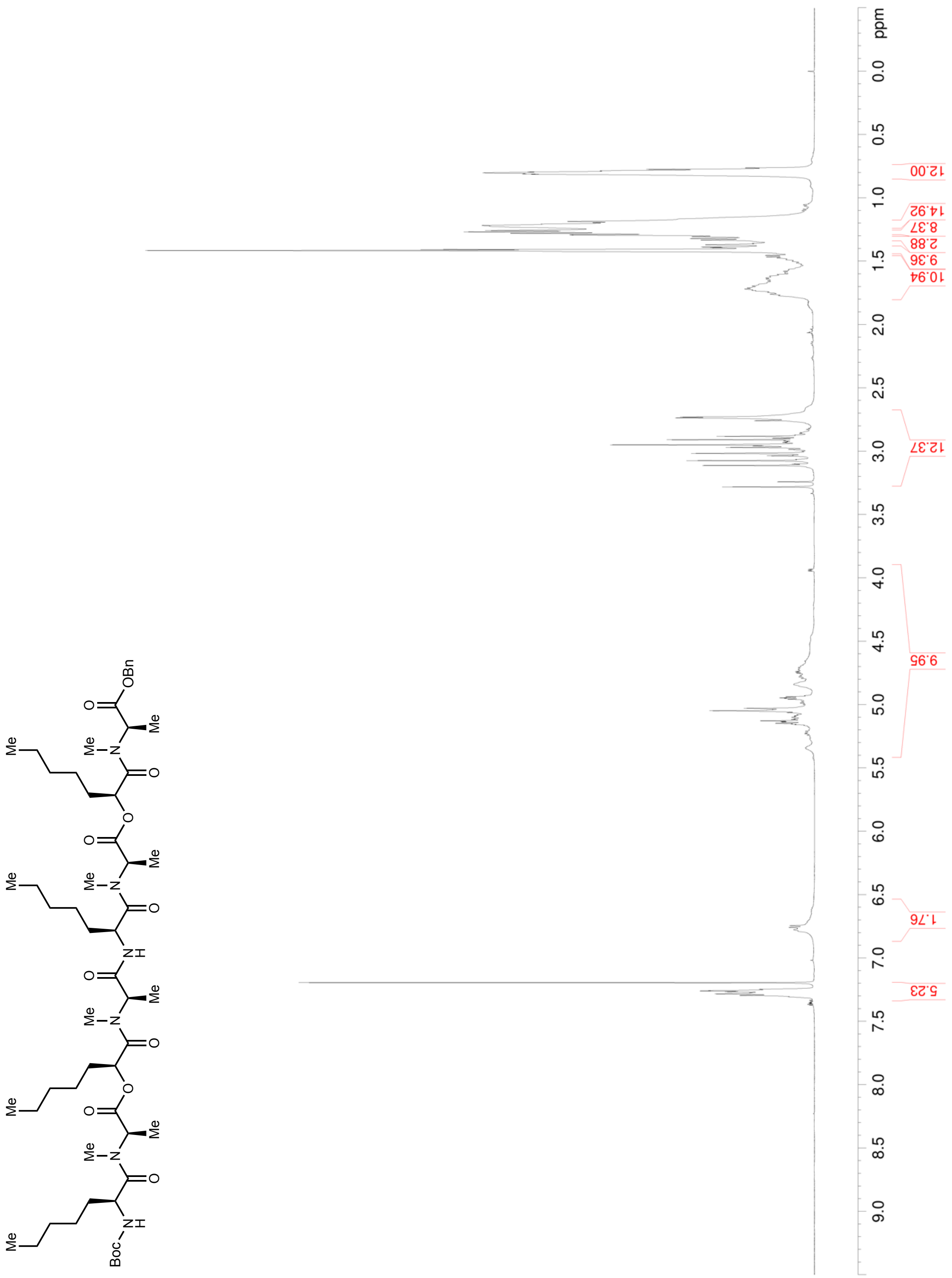

Figure S48. ^{13}C NMR (150 MHz, $CDCl_3$) of **O56**

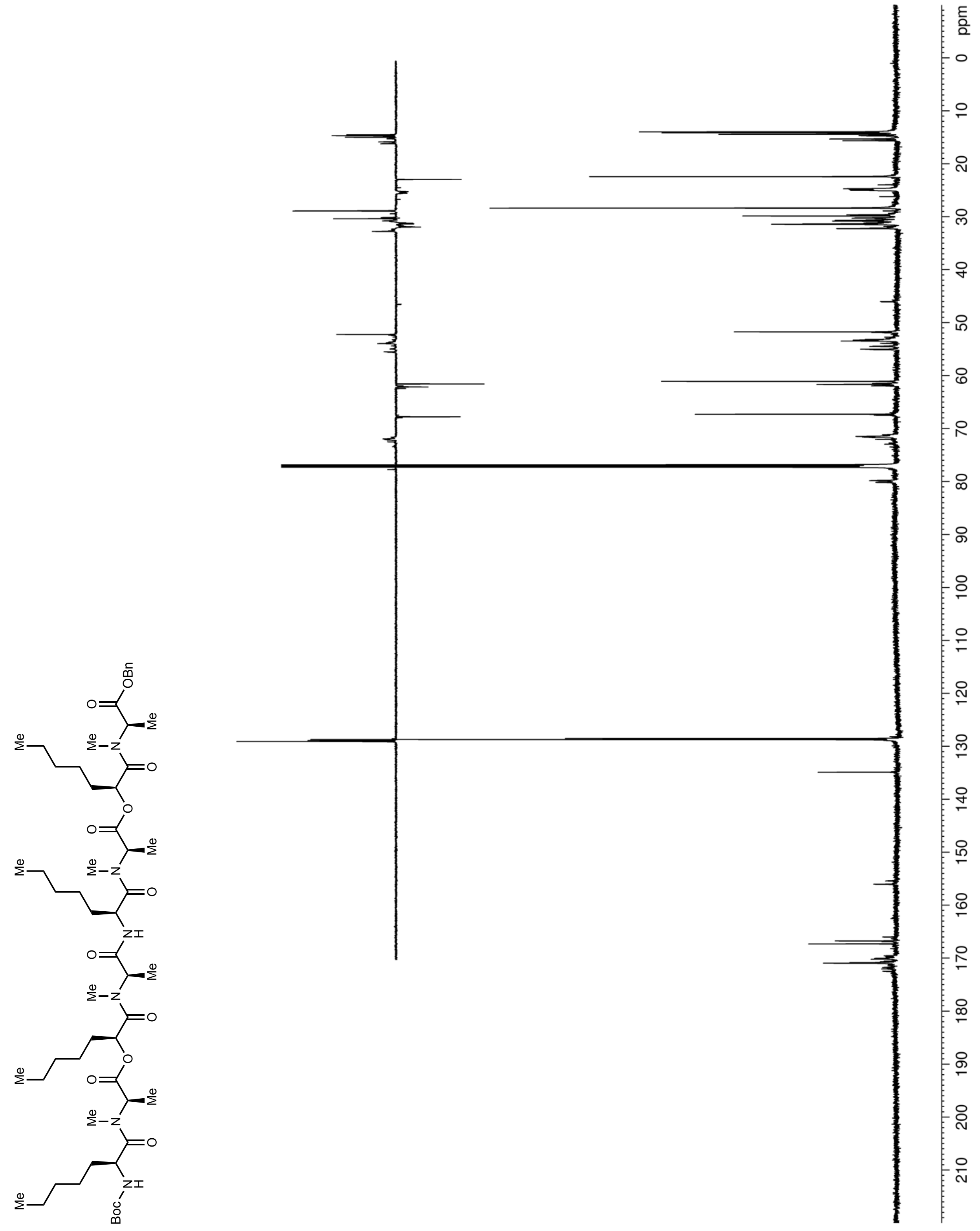

Figure S49. ^{1}H NMR (600 MHz, $CDCl_3$) of **H57**

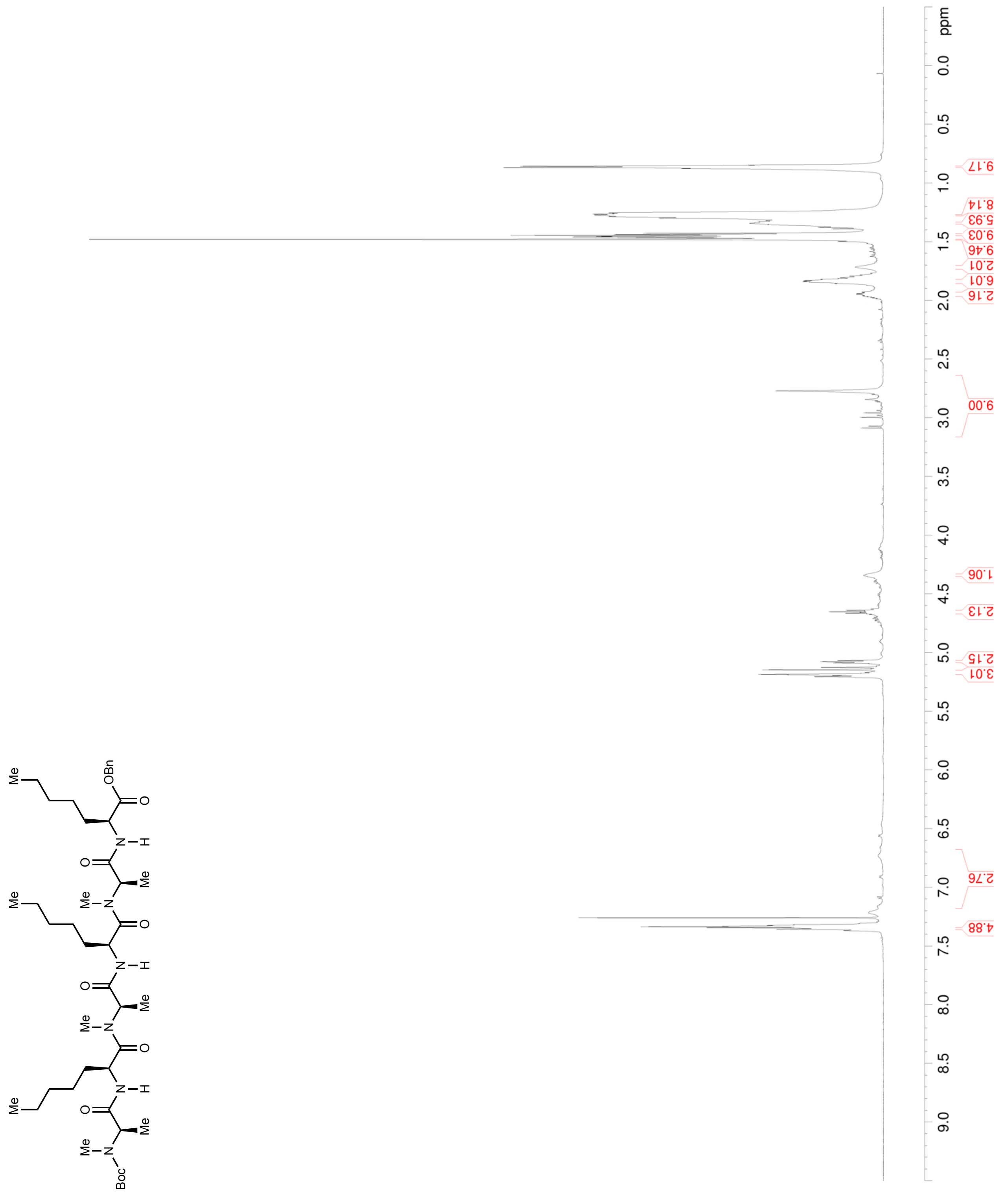

Figure S50. ^{13}C NMR (150 MHz, $CDCl_3$) of **H57**

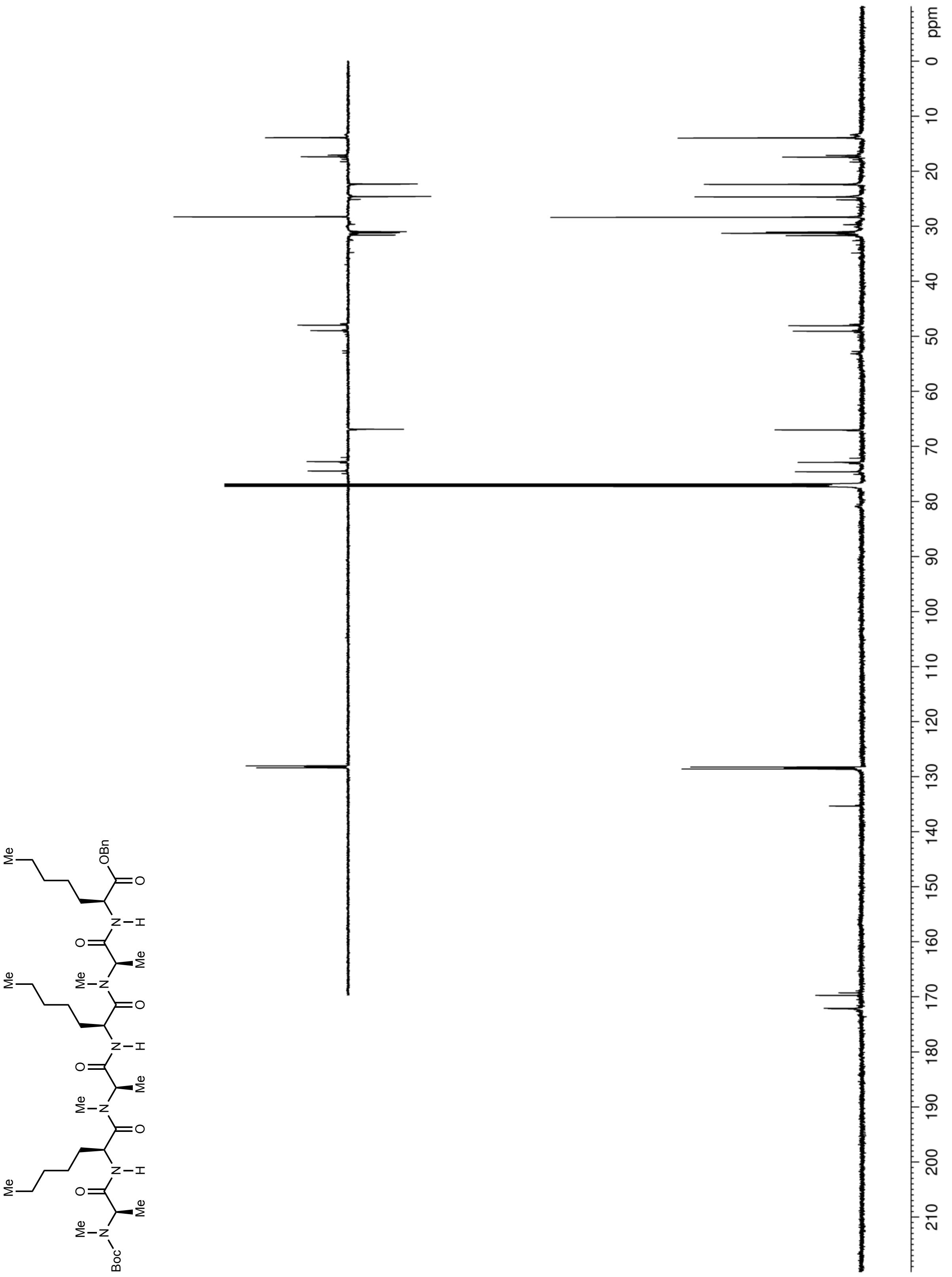

Figure S51. ^{1}H NMR (600 MHz, $CDCl_3$) of **O58 (Boc deprotected)**

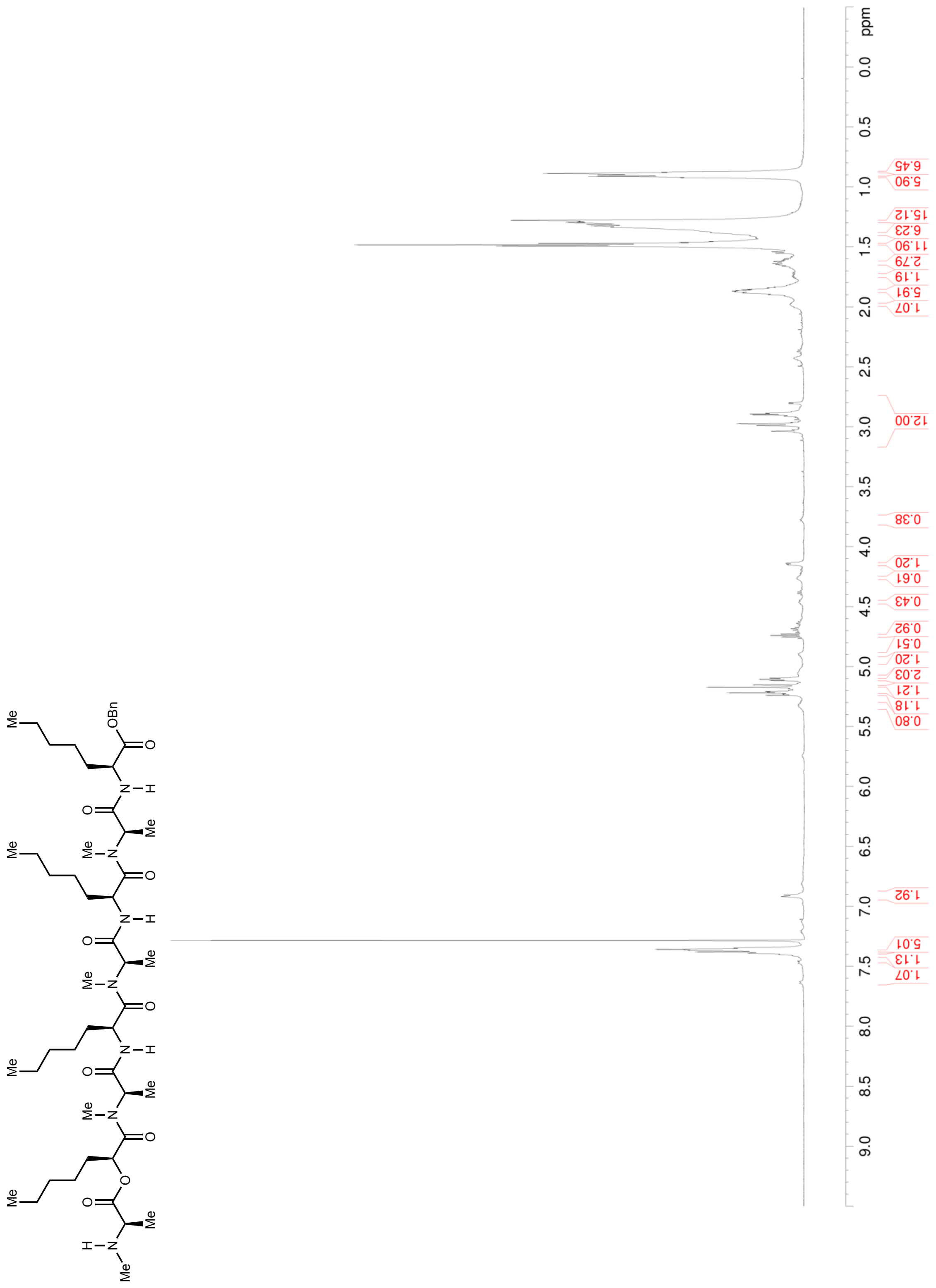

Figure S52. ^{13}C NMR (150 MHz, $CDCl_3$) of **O58 (Boc deprotected)**

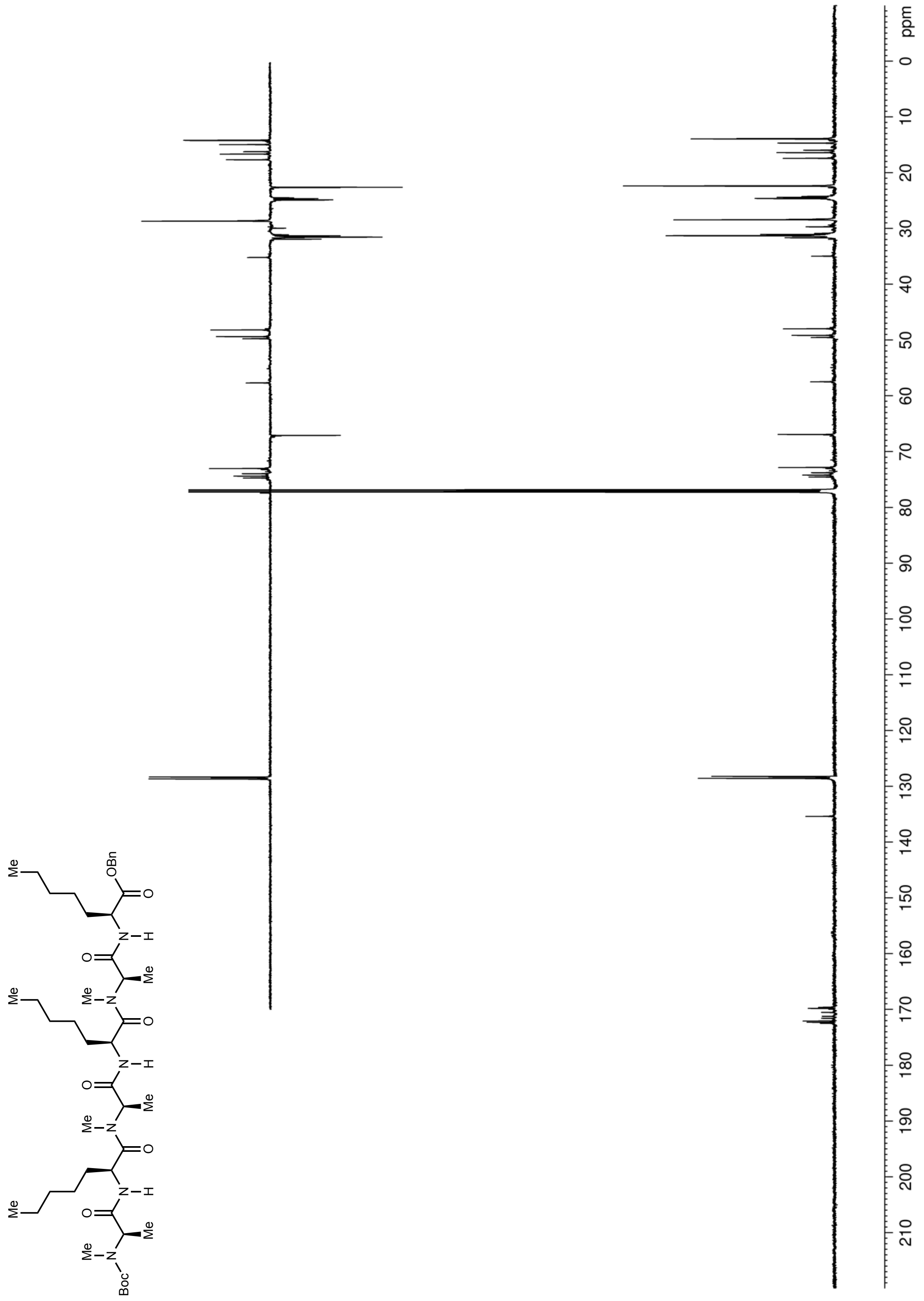

Figure S53. ^{1}H NMR (600 MHz, $CDCl_3$) of **O59**

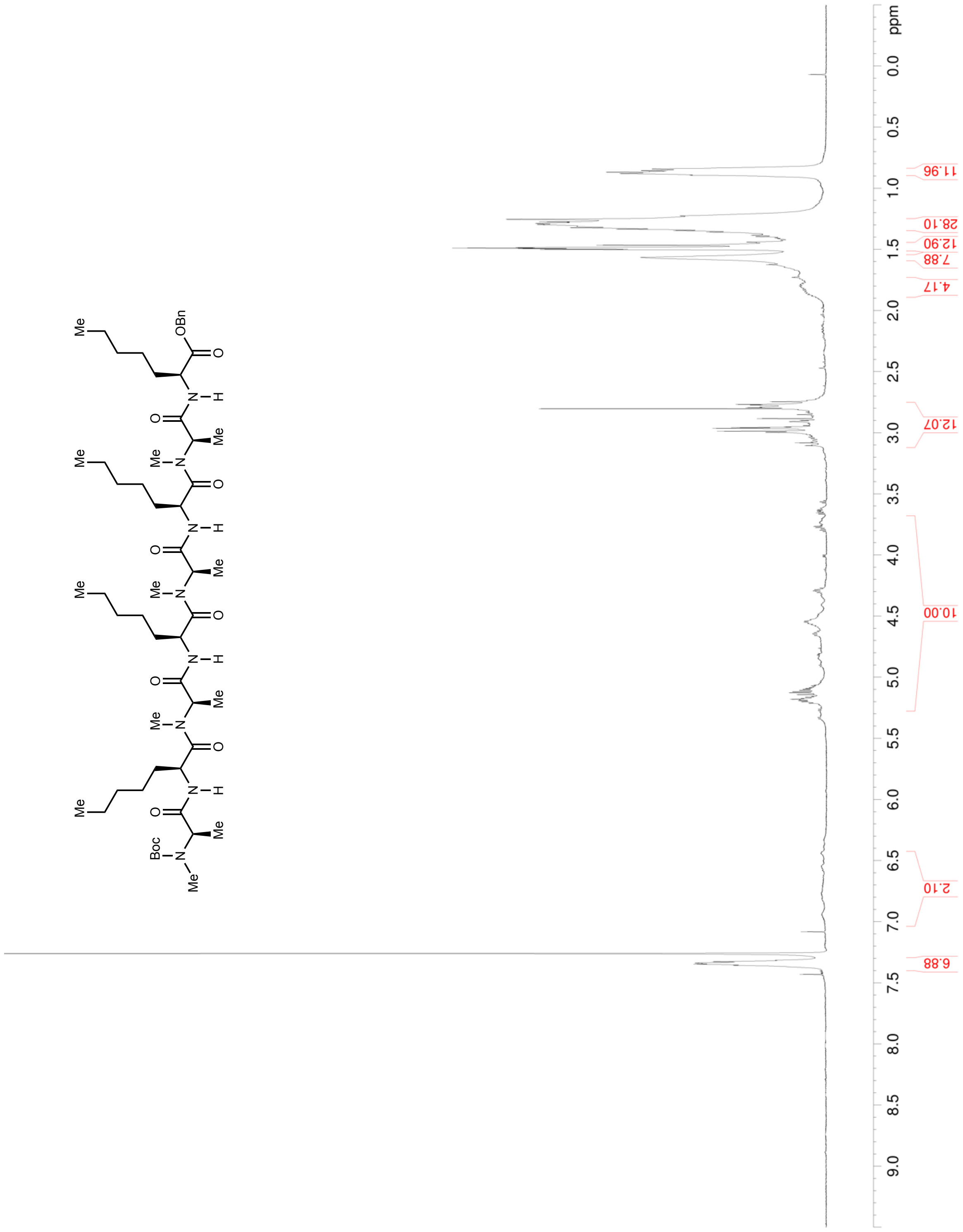

Figure S54. ^{13}C NMR (150 MHz, CDCl$_3$) of **O59**

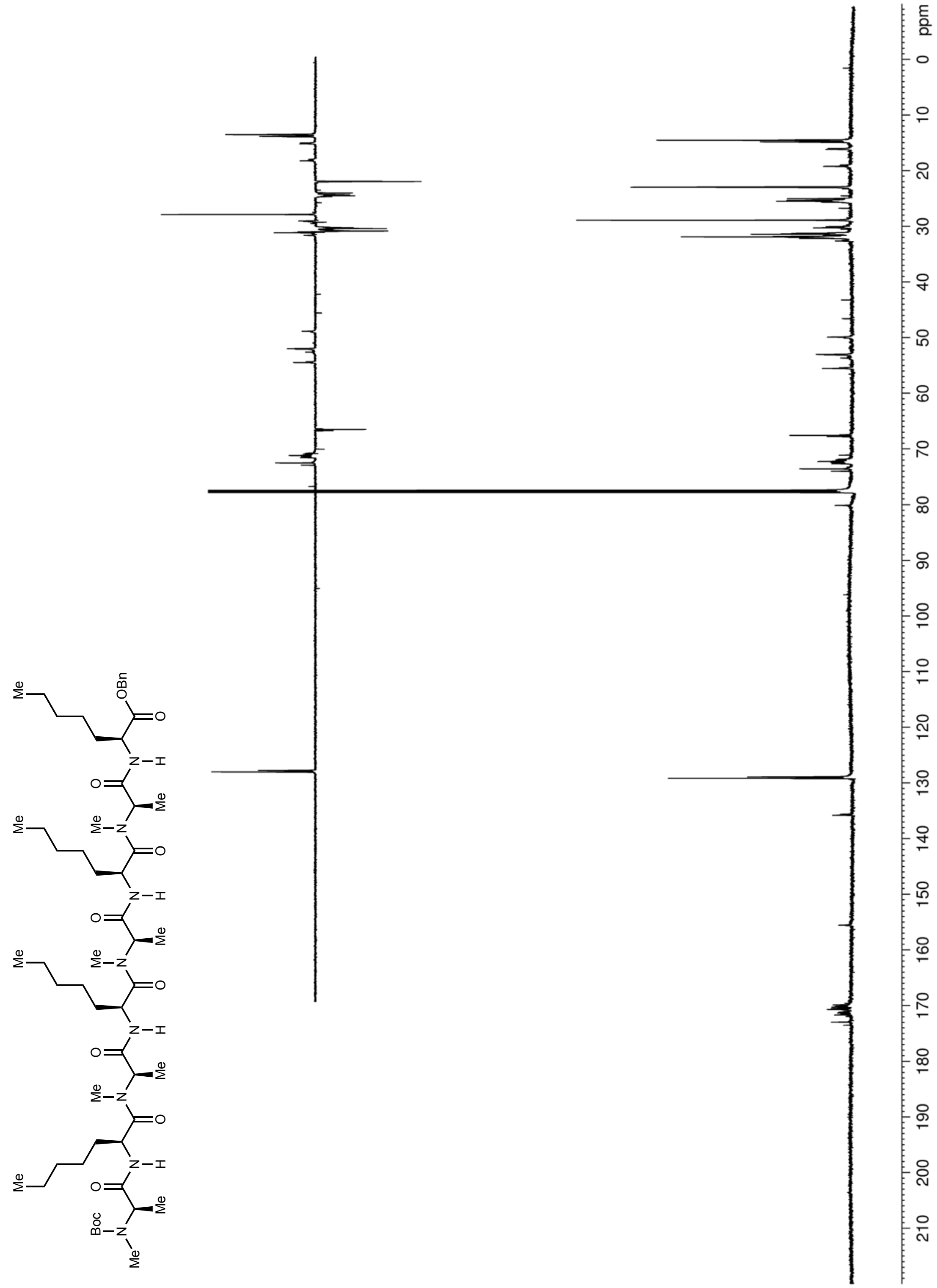

Figure S55. ^{1}H NMR (600 MHz, $CDCl_3$) of **O60**

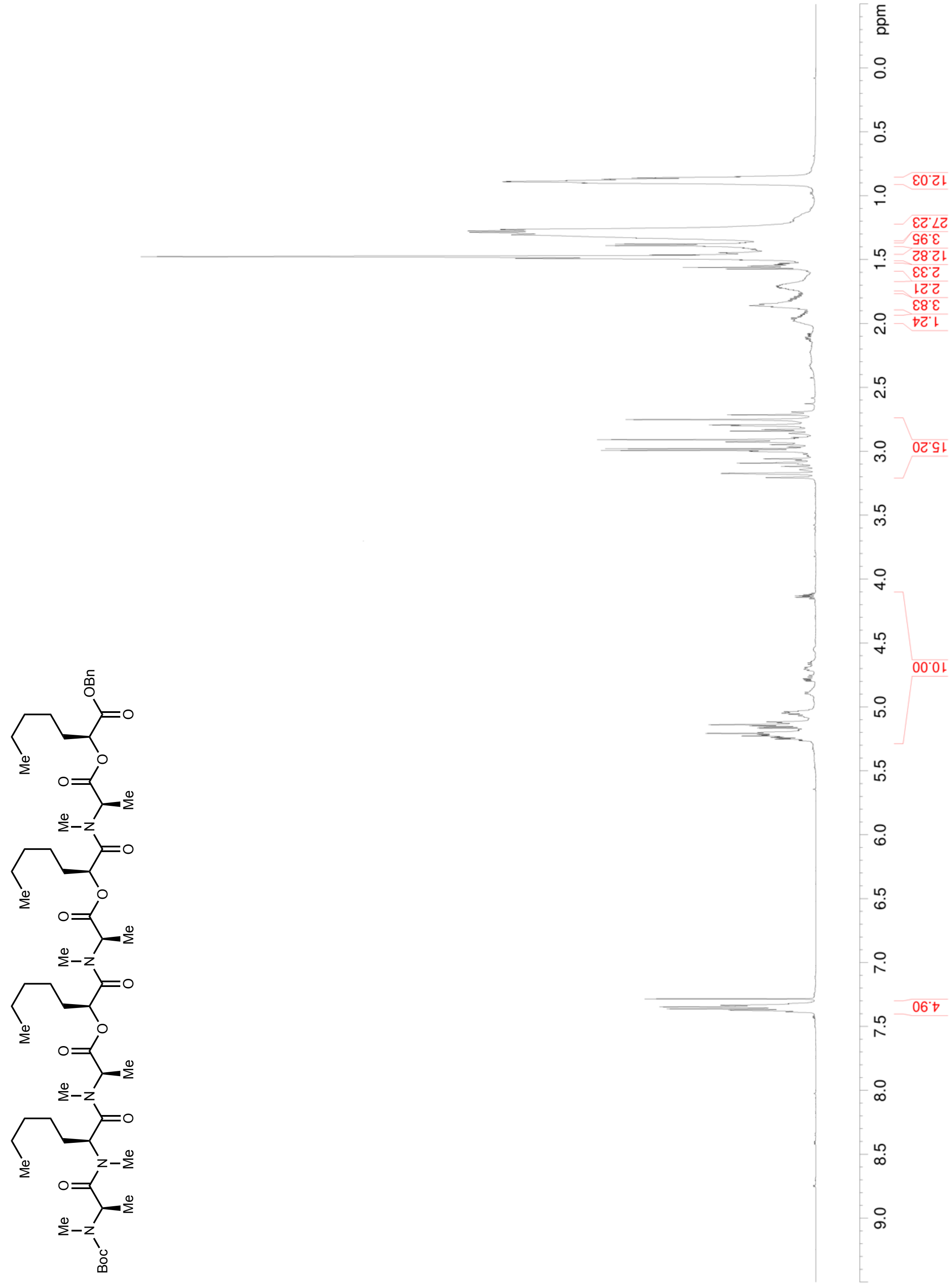

Figure S56. ^{13}C NMR (150 MHz, $CDCl_3$) of **O60**

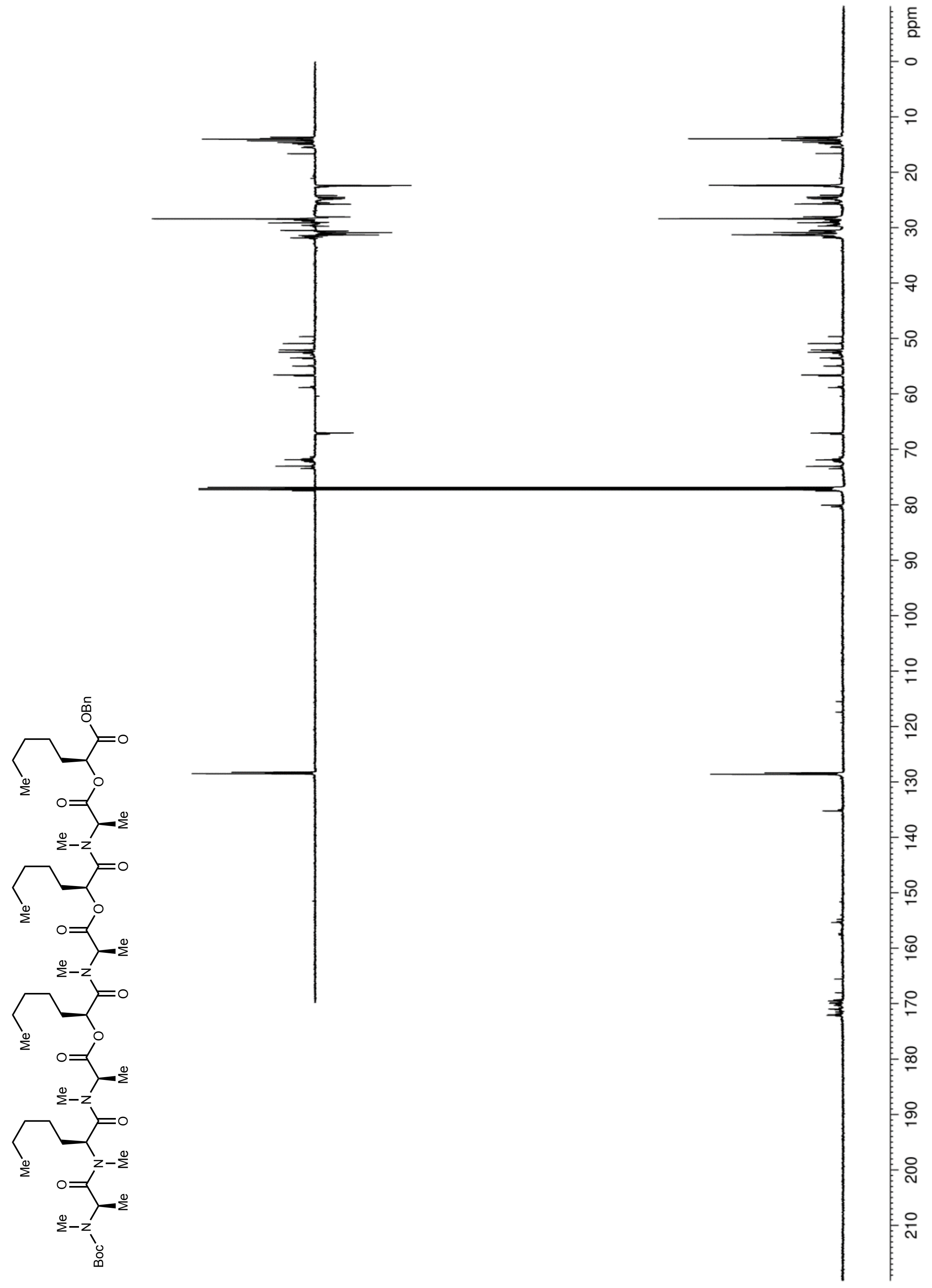

Figure S57. ^{1}H NMR (600 MHz, $CDCl_3$) of **O61**

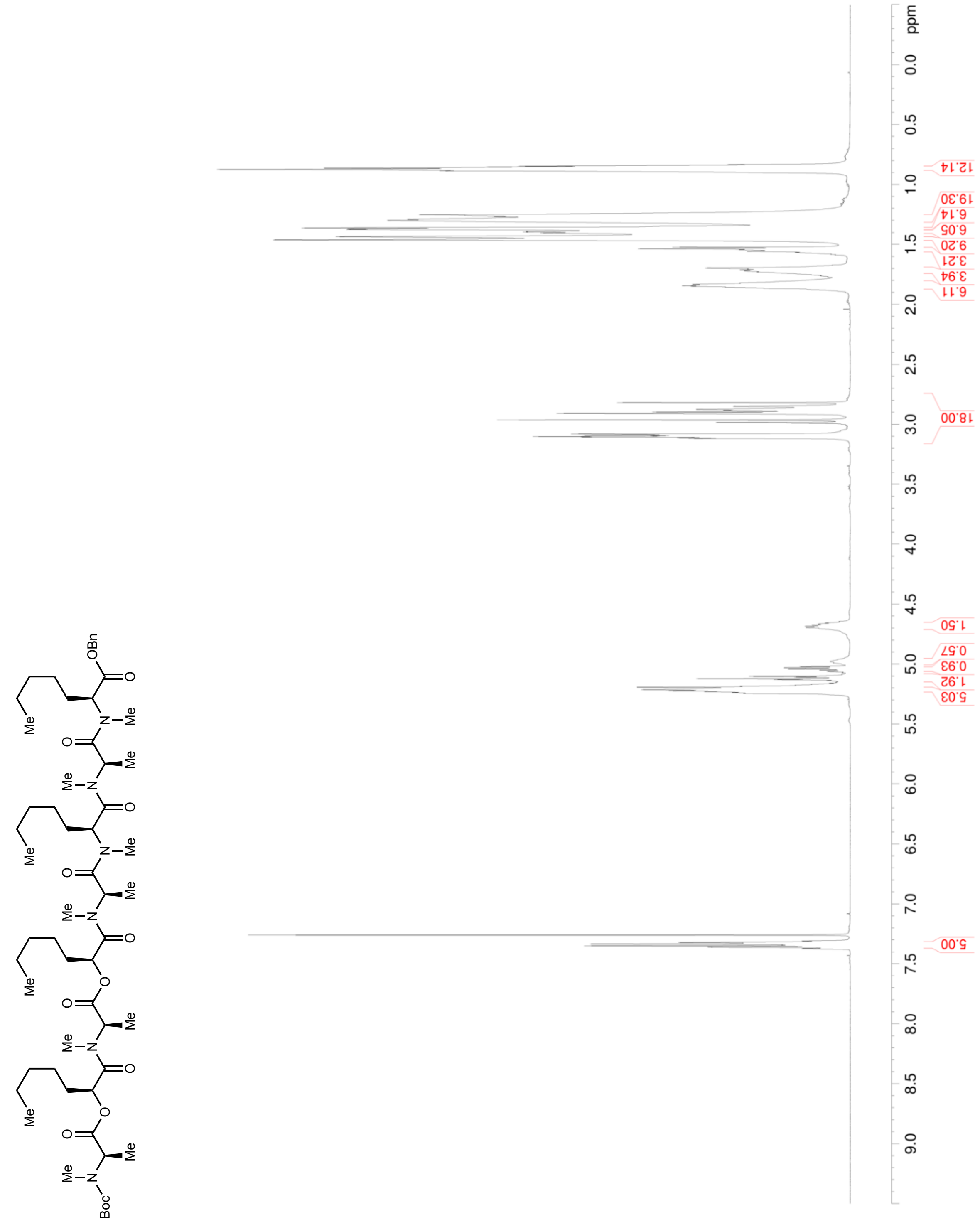

Figure S58. ^{13}C NMR (150 MHz, $CDCl_3$) of **O61**

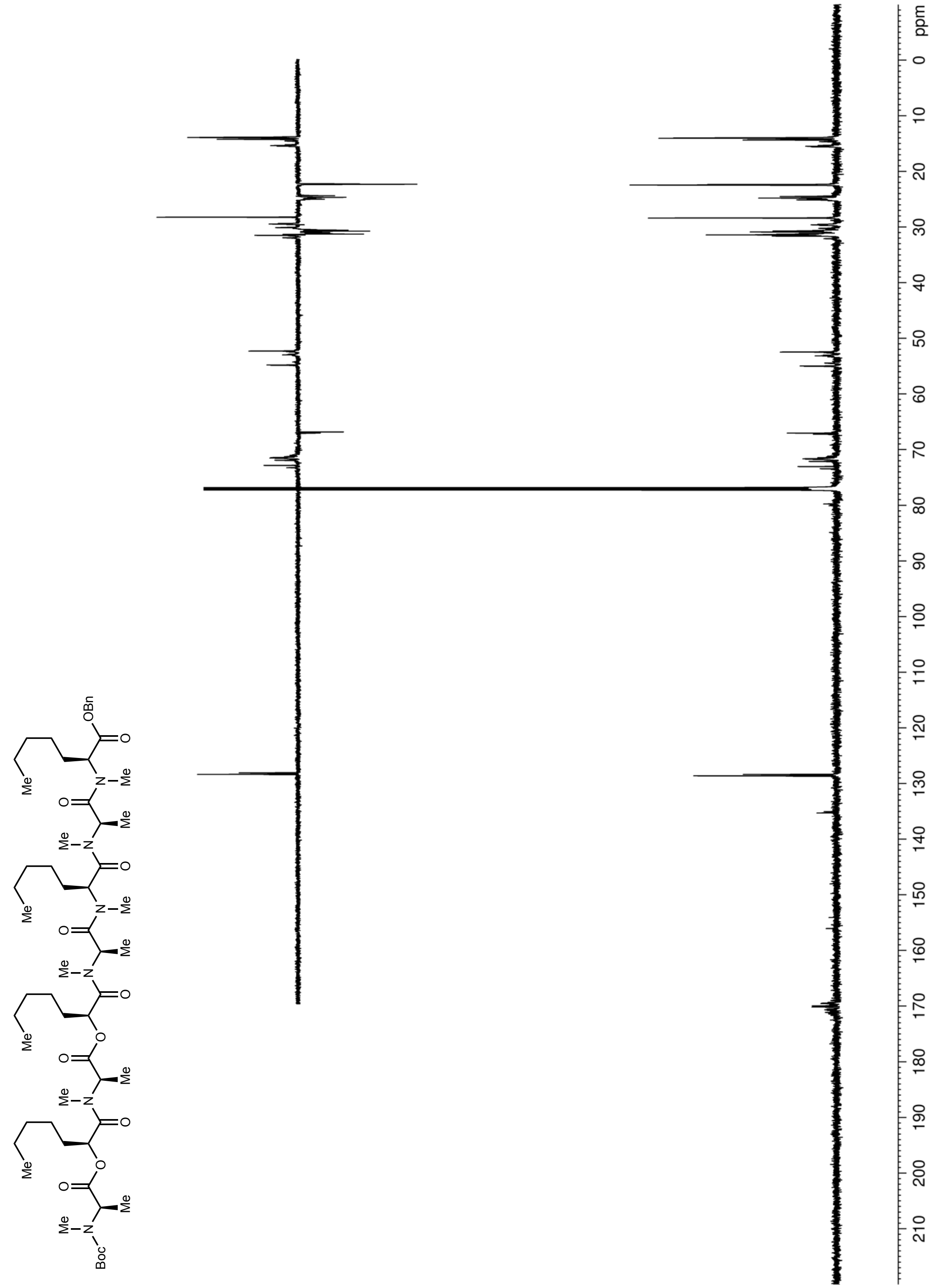

Figure S59. ^{1}H NMR (600 MHz, $CDCl_3$) of **O62**

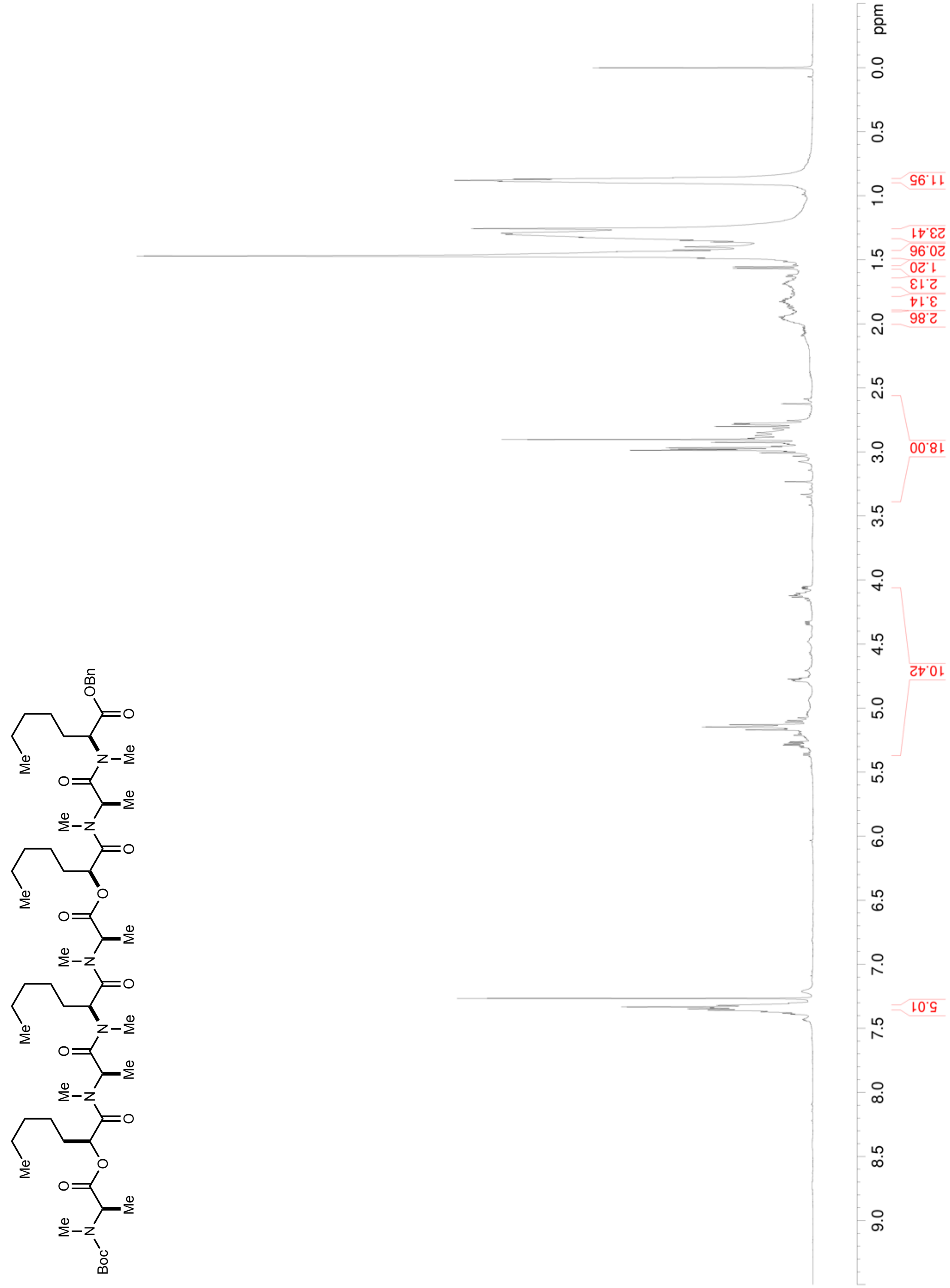

Figure S60. ^{13}C NMR (150 MHz, $CDCl_3$) of **O62**

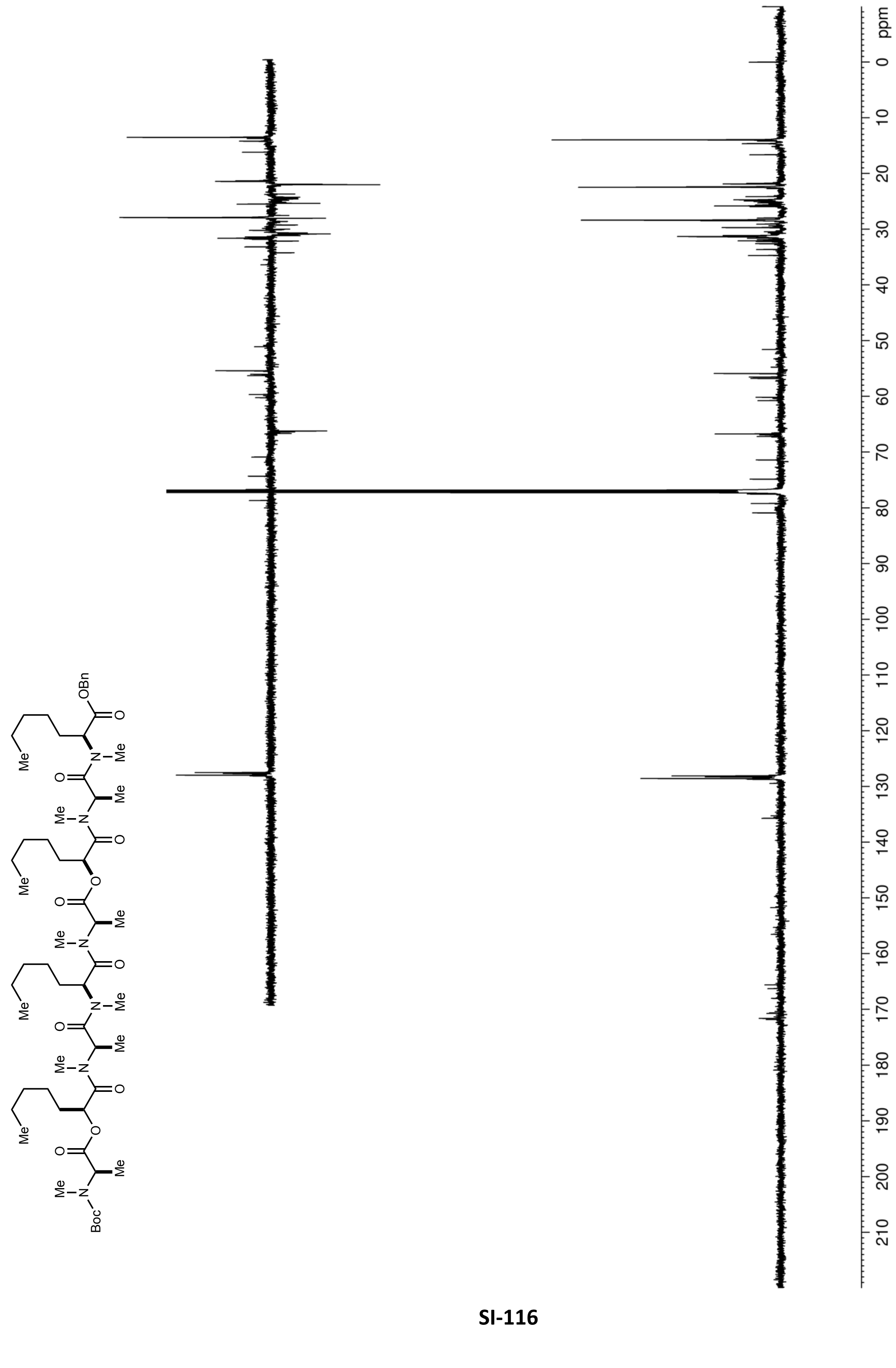

Figure S61. ^{1}H NMR (600 MHz, $CDCl_3$) of **H63**

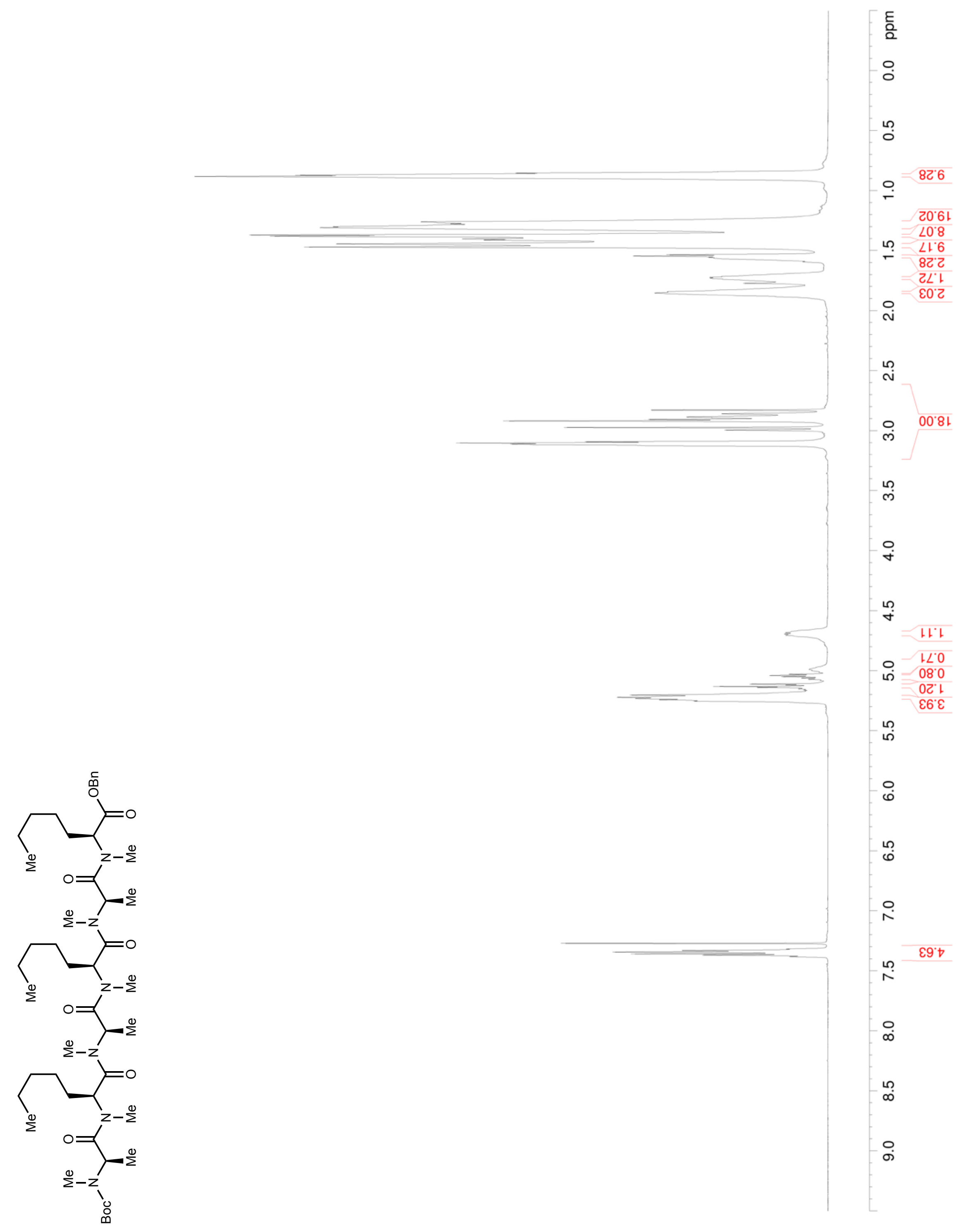

Figure S62. ^{13}C NMR (150 MHz, $CDCl_3$) of **H63**

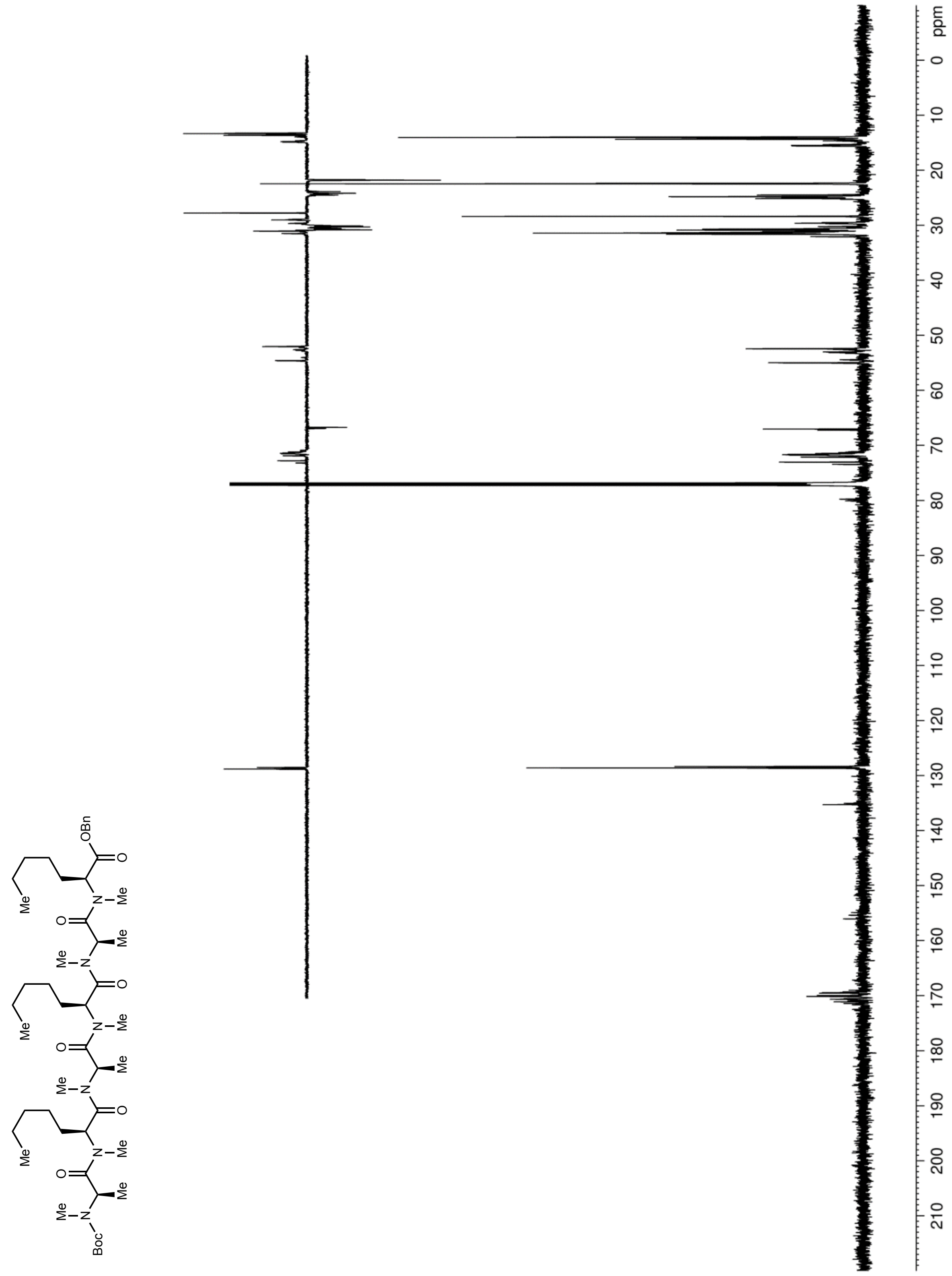

Figure S63. ^{1}H NMR (600 MHz, $CDCl_3$) of **O64**

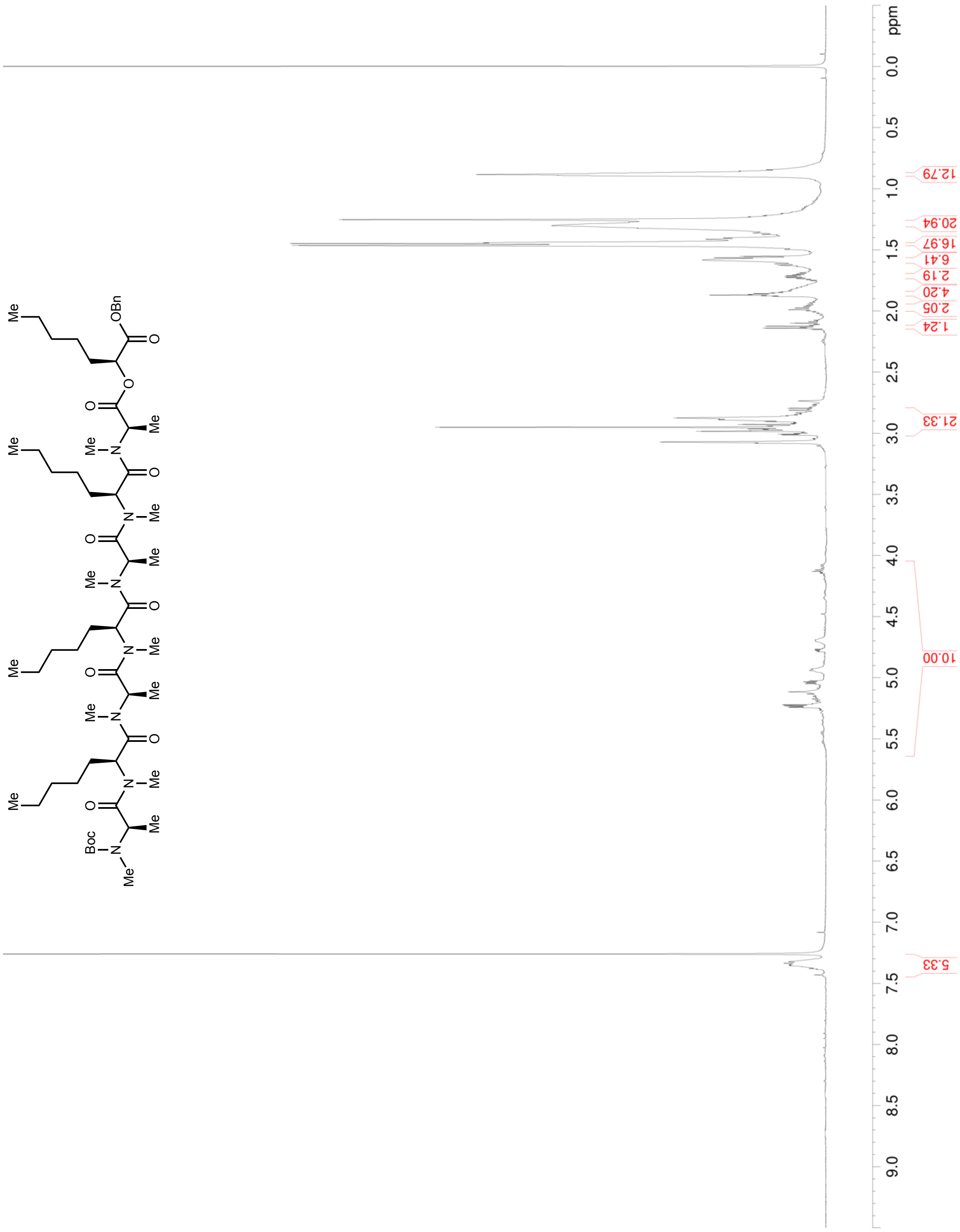

Figure S64. ^{13}C NMR (150 MHz, $CDCl_3$) of **O64**

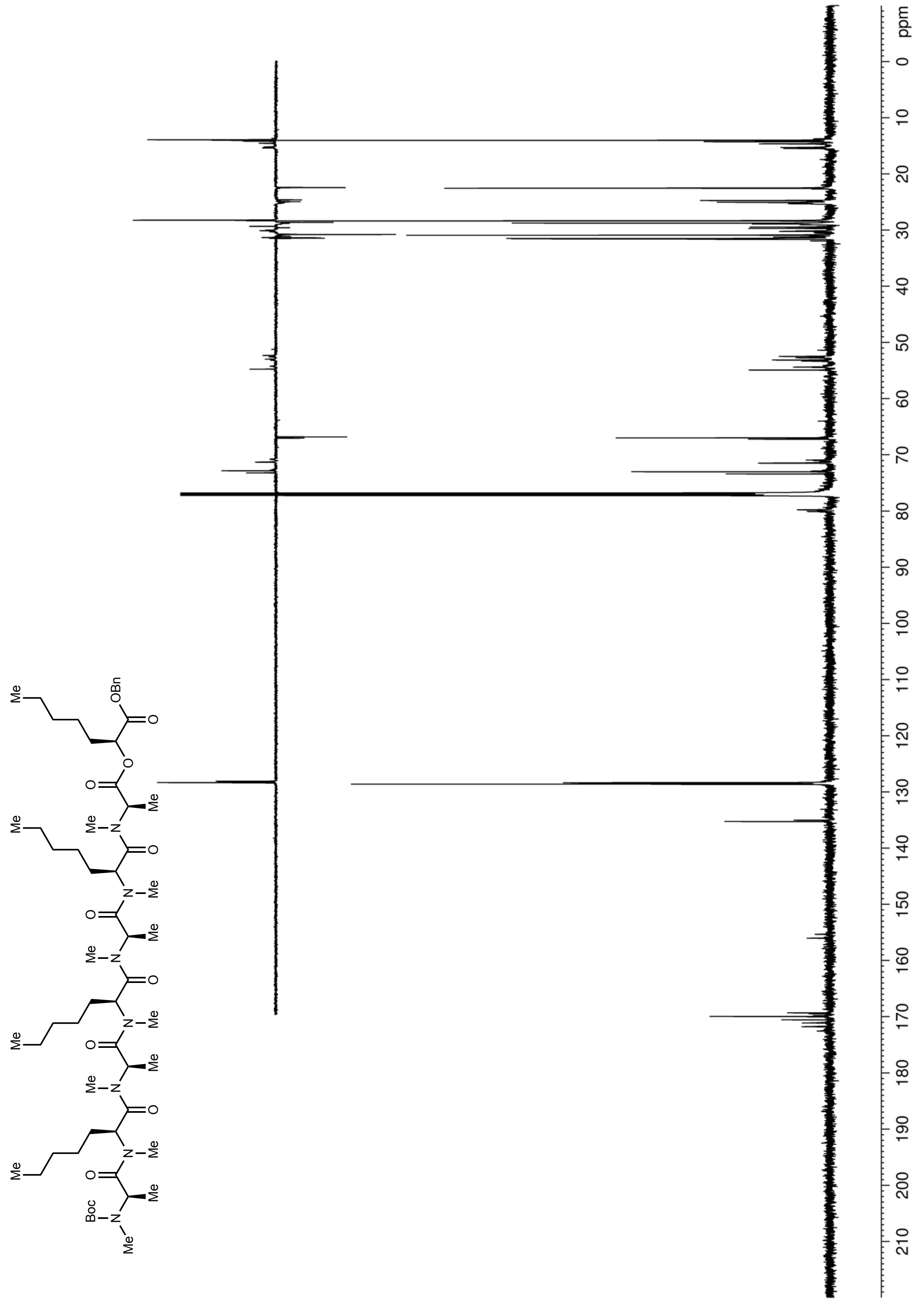

Figure S65. ^{1}H NMR (600 MHz, $CDCl_3$) of **O65**

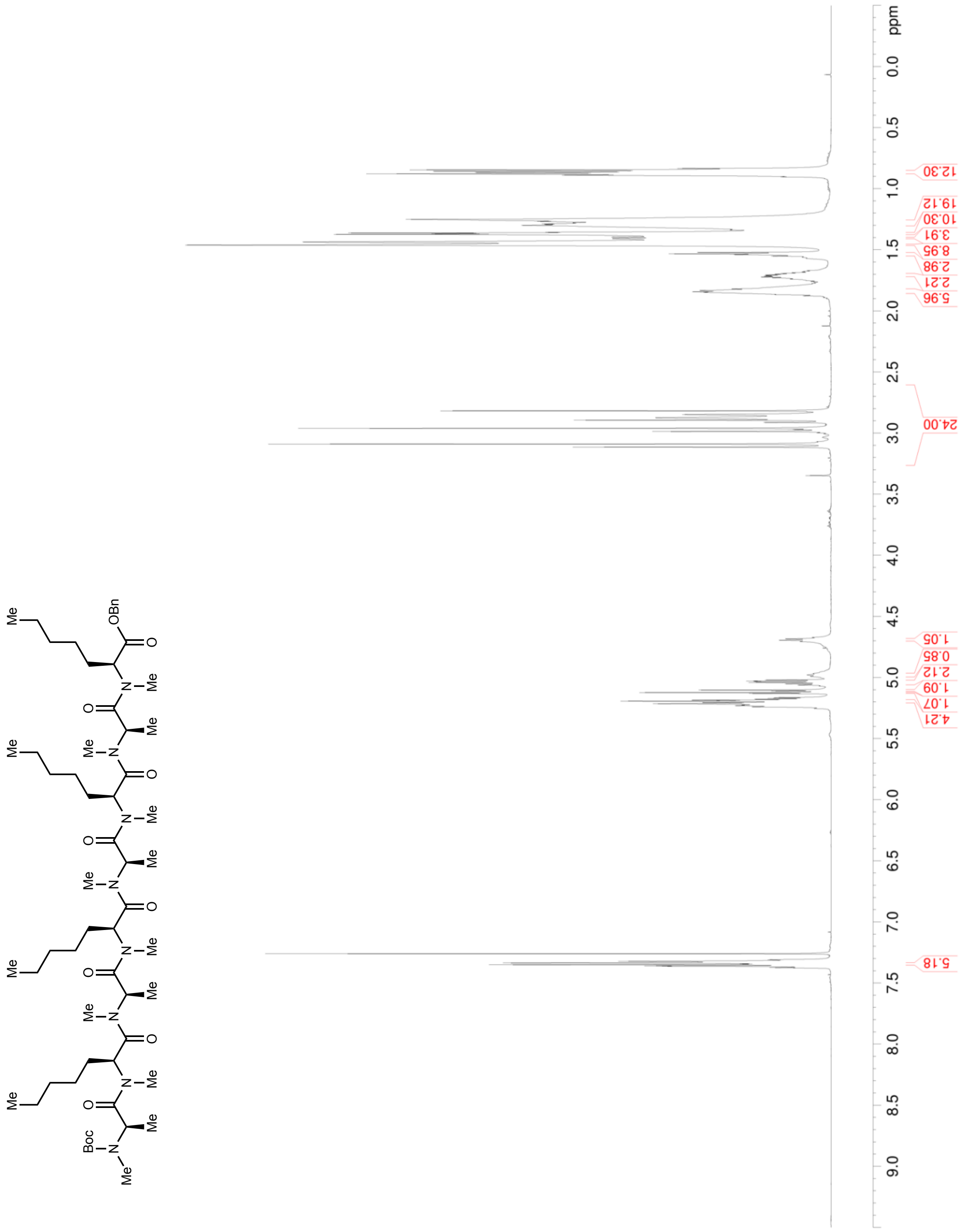

Figure S66. ^{13}C NMR (150 MHz, $CDCl_3$) of **O65**

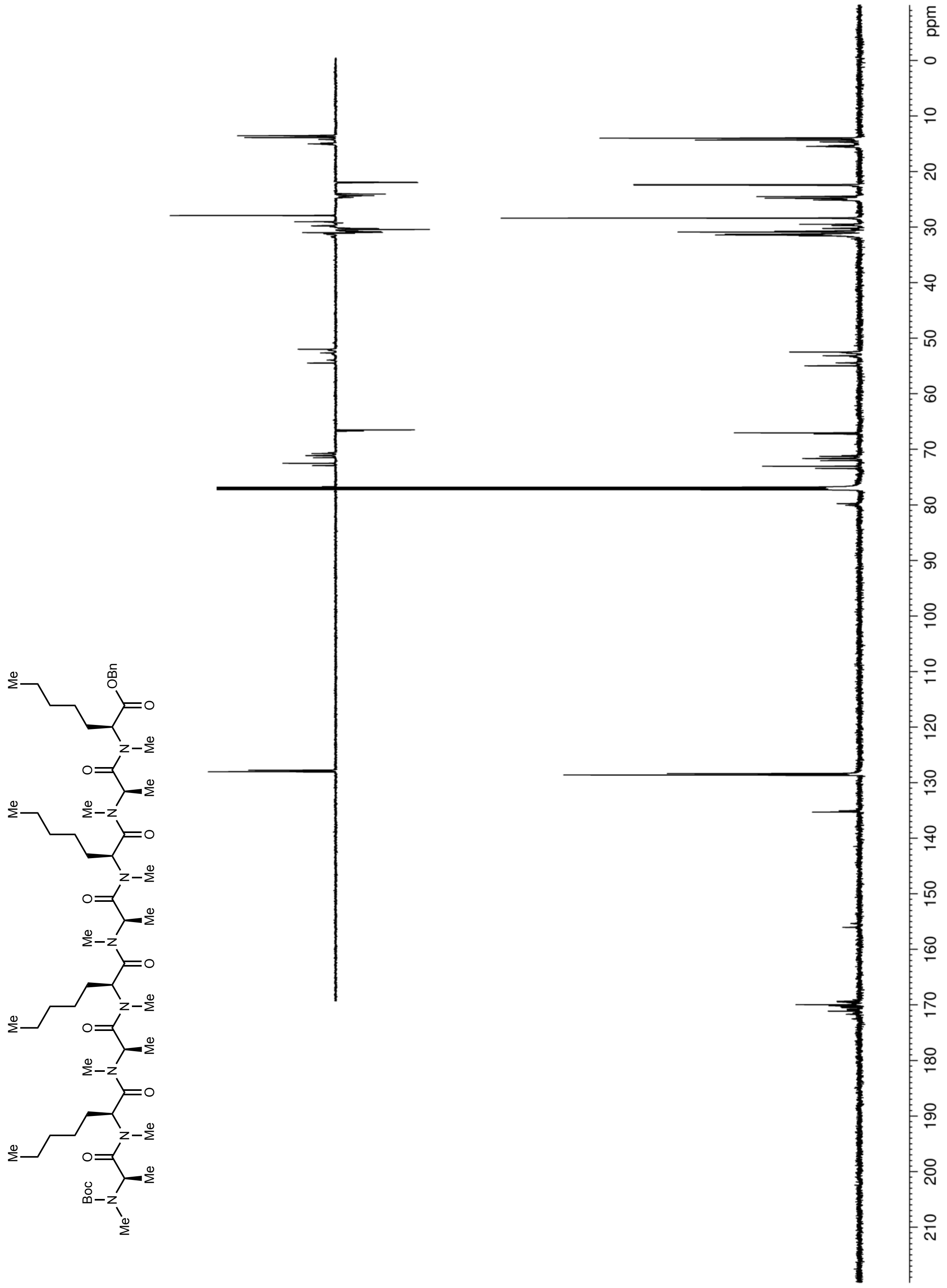

Figure S67. ^{1}H NMR (600 MHz, $CDCl_3$) of **O66**

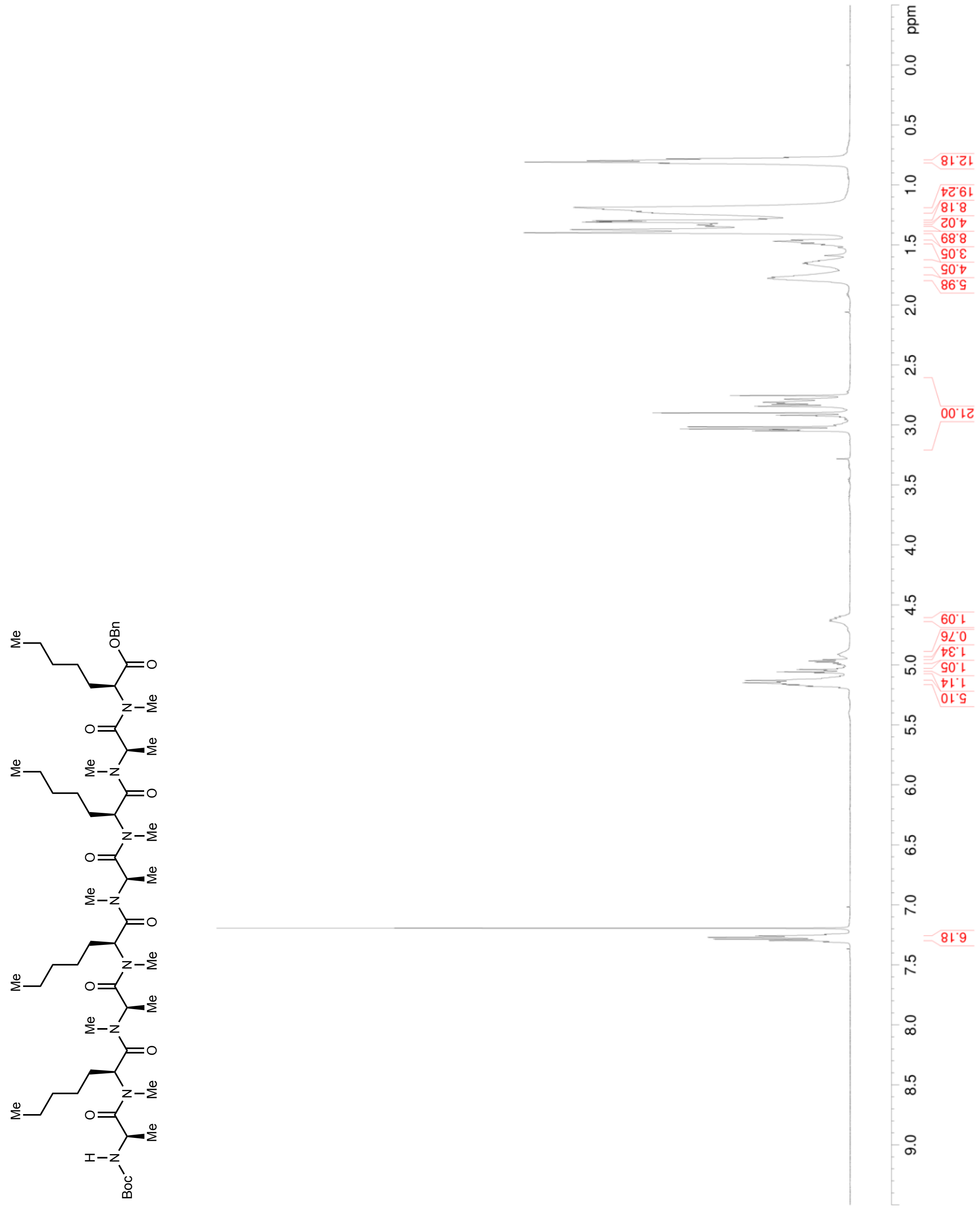

Figure S68. ^{13}C NMR (150 MHz, $CDCl_3$) of **O66**

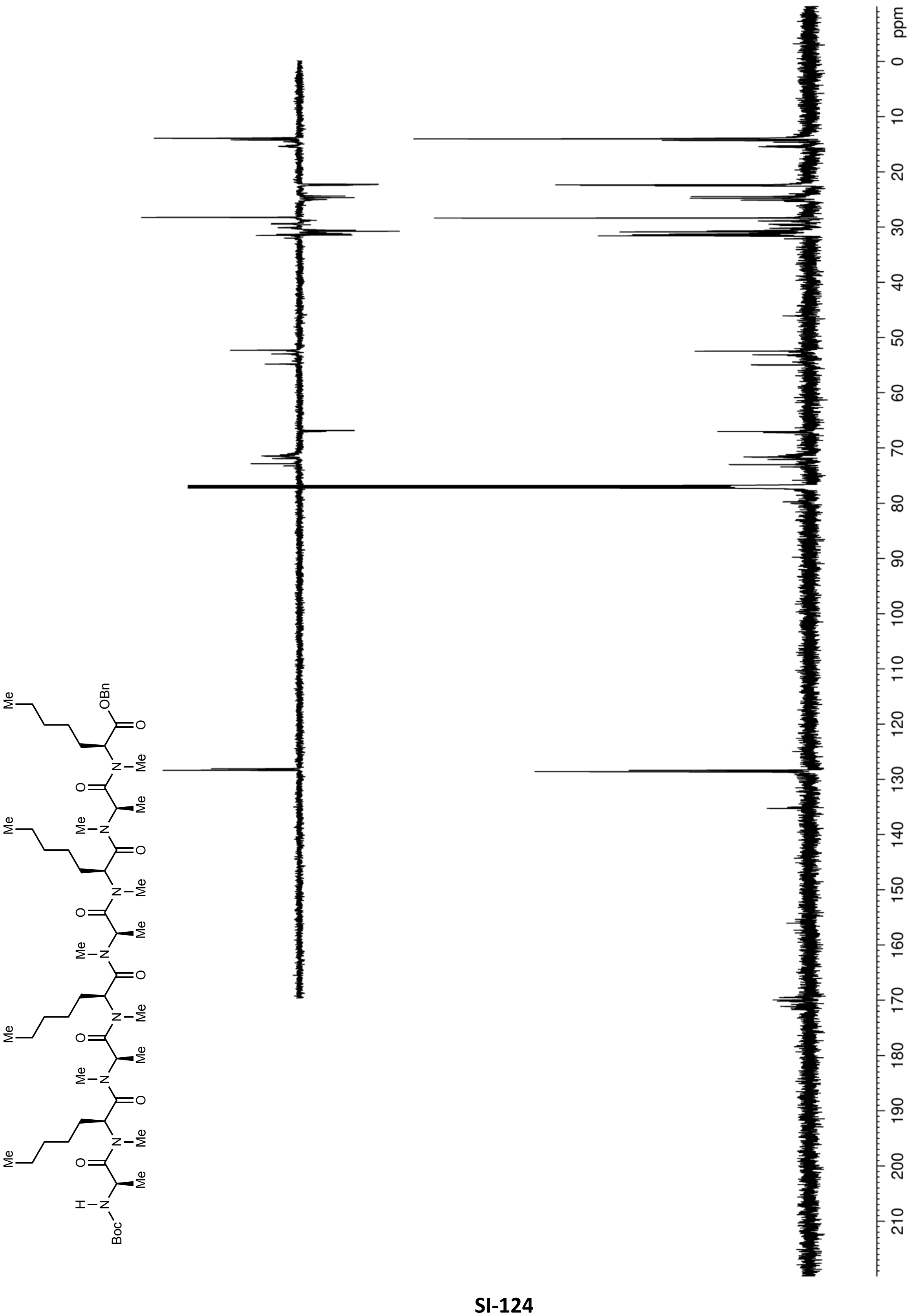

Figure S69. ^{1}H NMR (600 MHz, $CDCl_3$) of **O67**

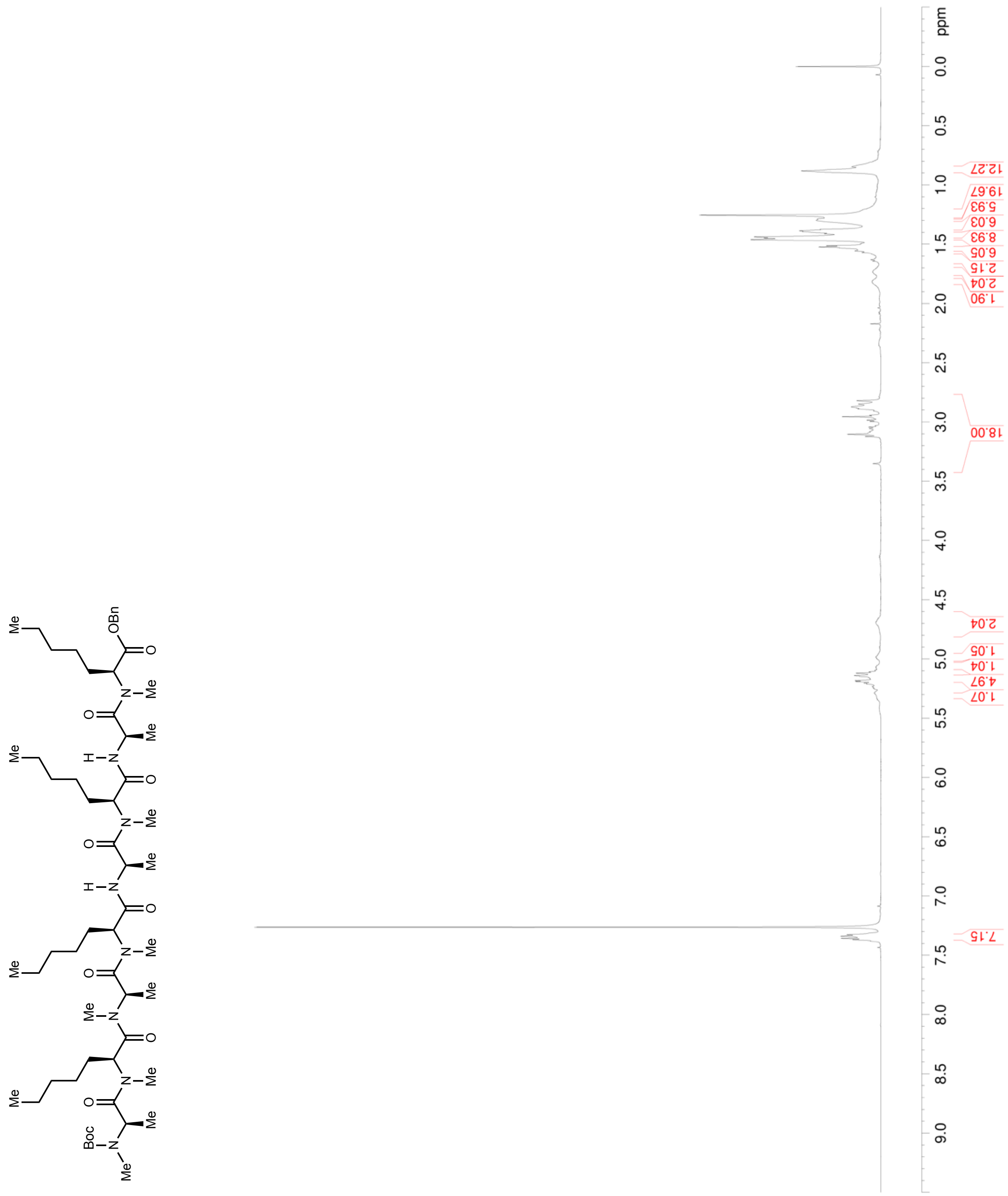

Figure S70. ^{13}C NMR (150 MHz, CDCl$_3$) of **O67**

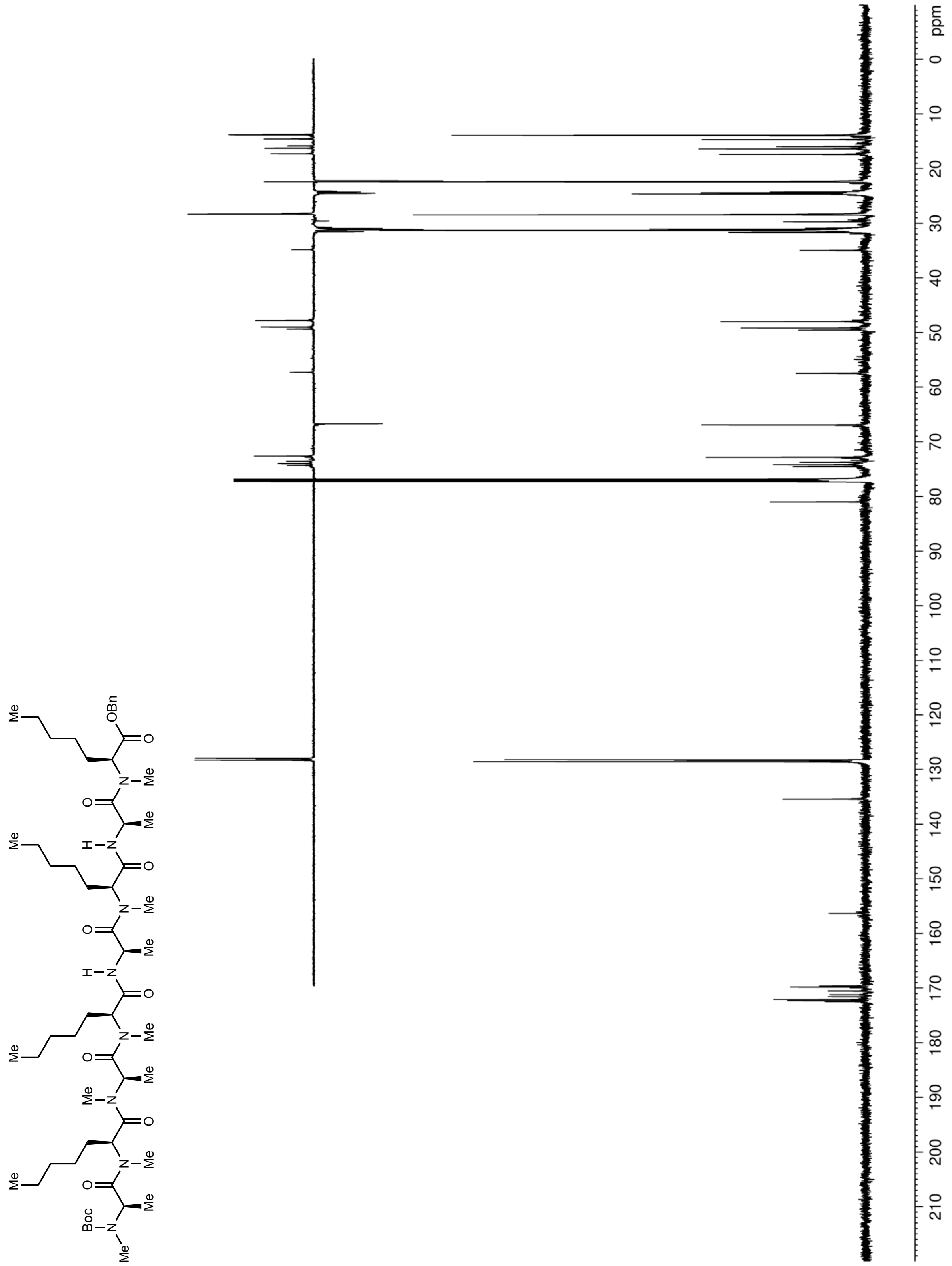

Figure S71. ^{1}H NMR (600 MHz, $CDCl_3$) of **O68**

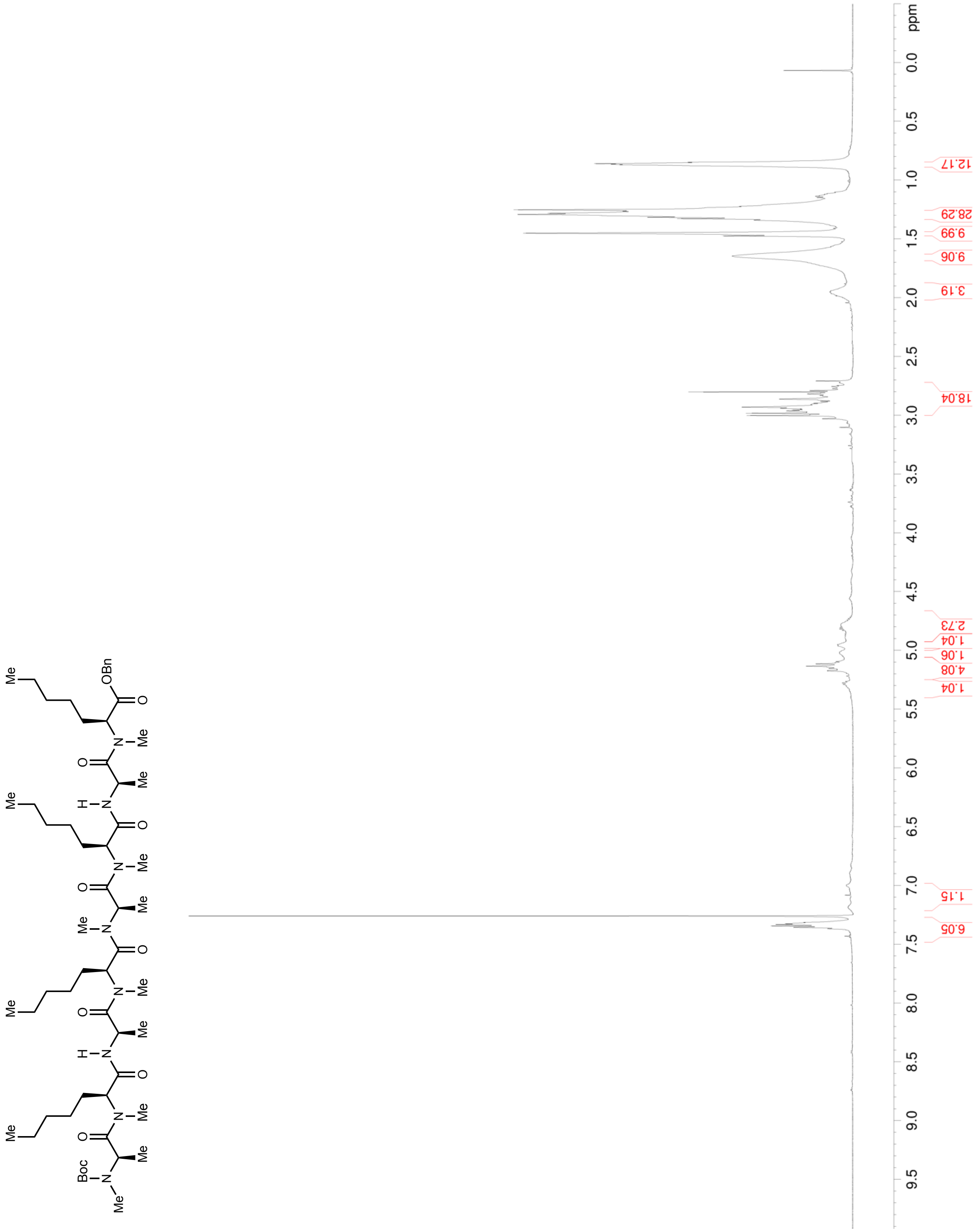

Figure S72. ^{13}C NMR (150 MHz, $CDCl_3$) of **O68**

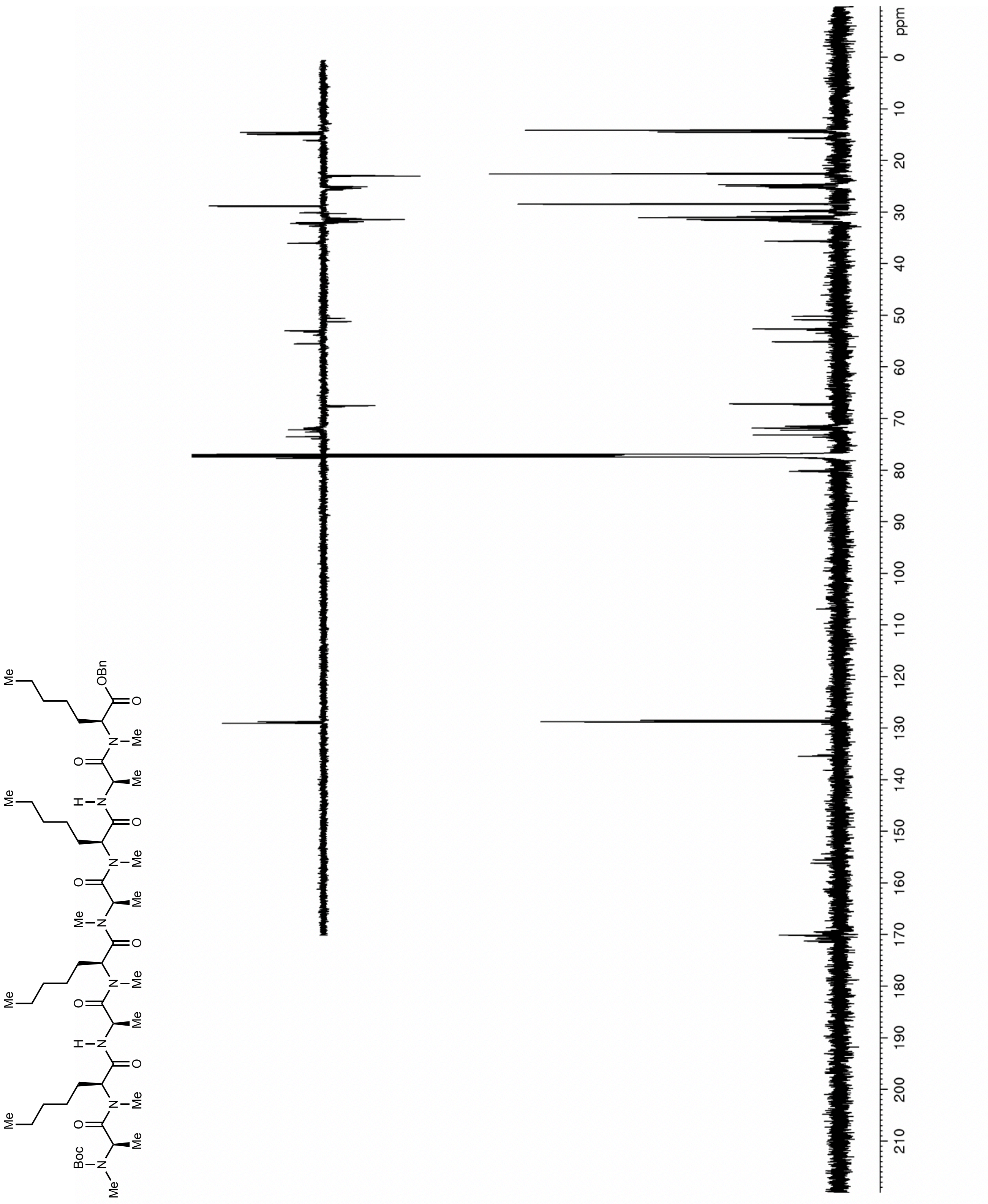

Figure S73. ^{1}H NMR (600 MHz, $CDCl_3$) of **O69**

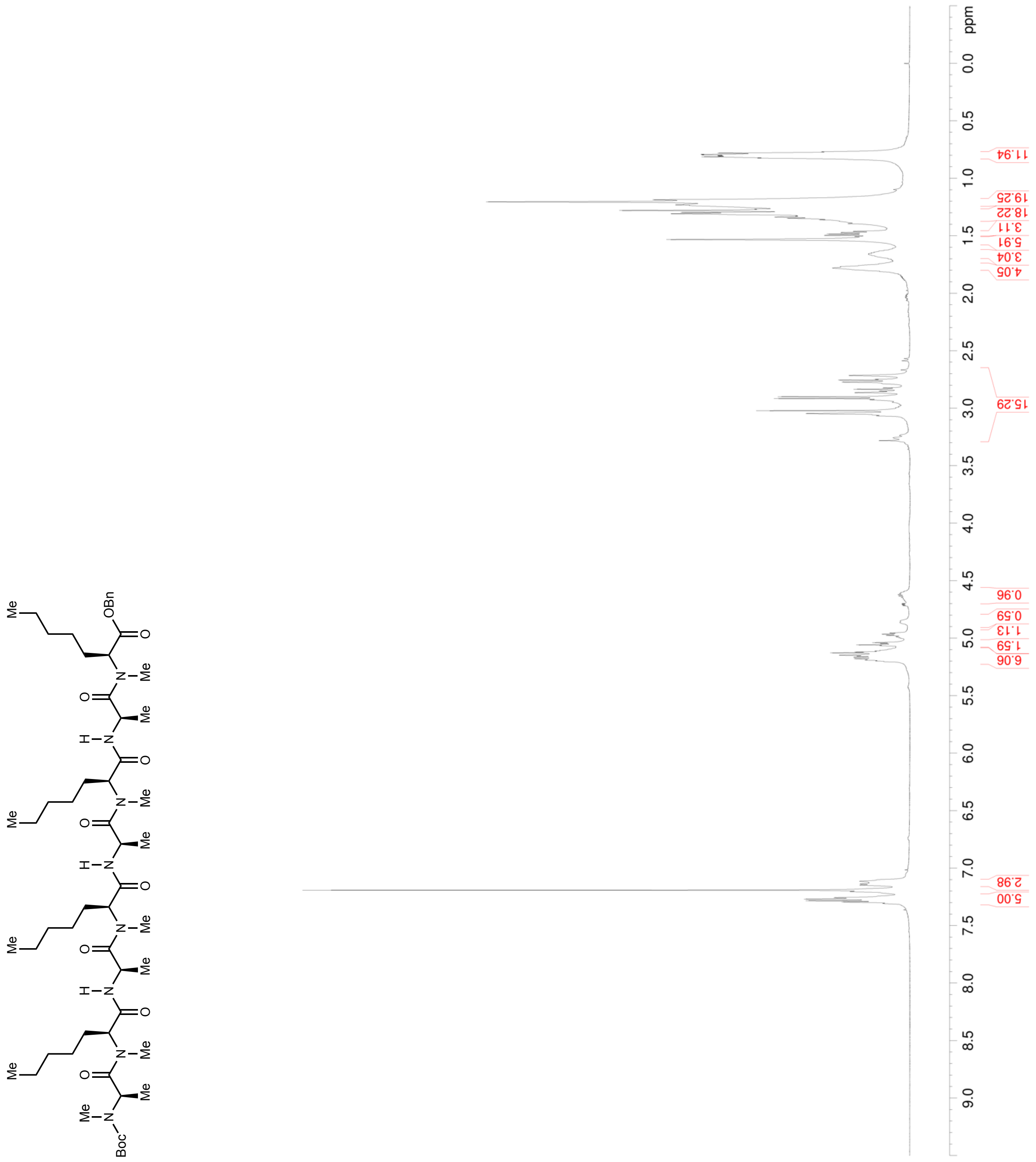

Figure S74. ^{13}C NMR (150 MHz, $CDCl_3$) of **O69**

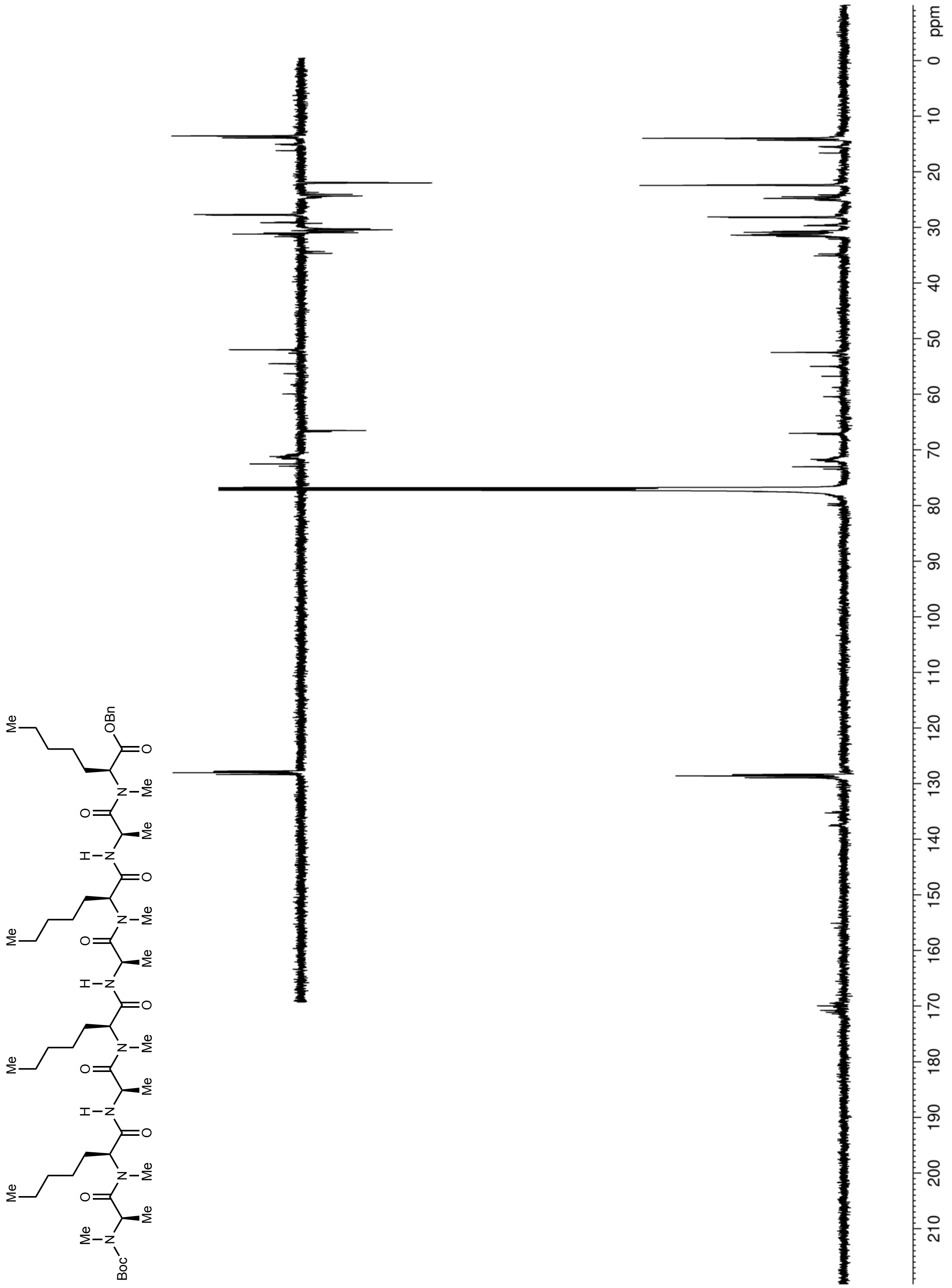

Figure S75. ^{1}H NMR (600 MHz, $CDCl_3$) of **O70**

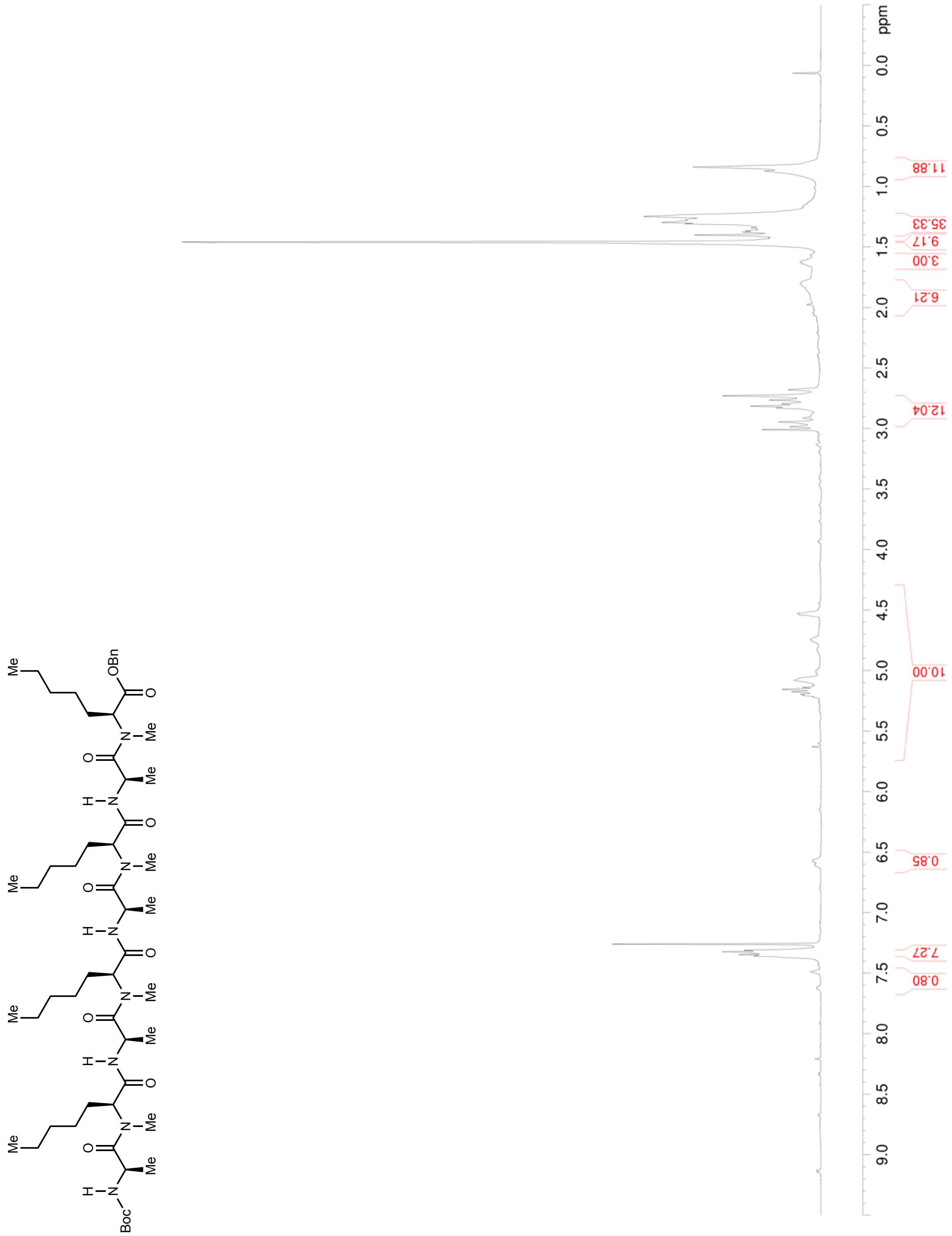

Figure S76. ^{13}C NMR (150 MHz, $CDCl_3$) of **O70**

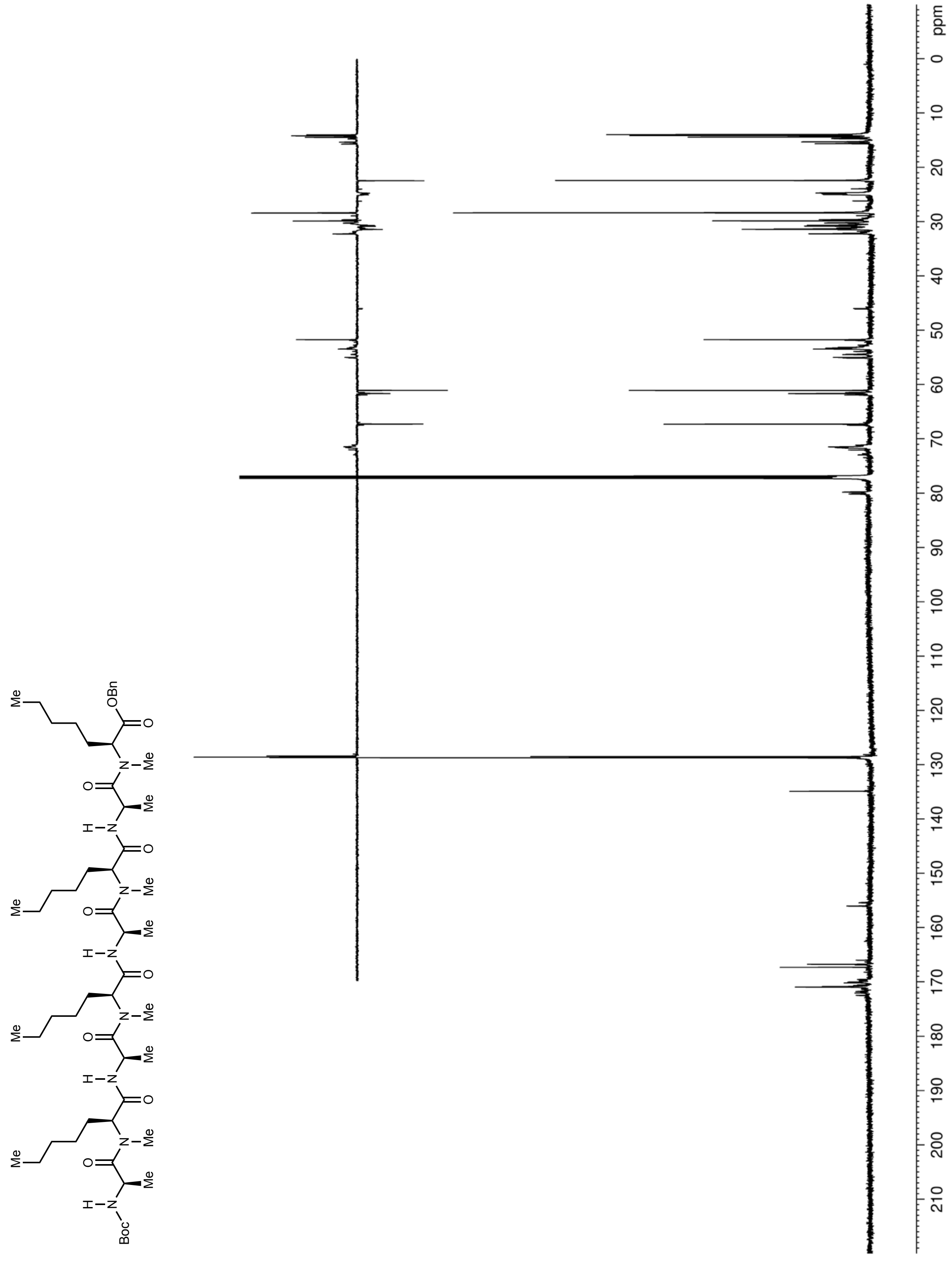

Figure S77. ^{1}H NMR (600 MHz, ($CDCl_3$) of **O71**

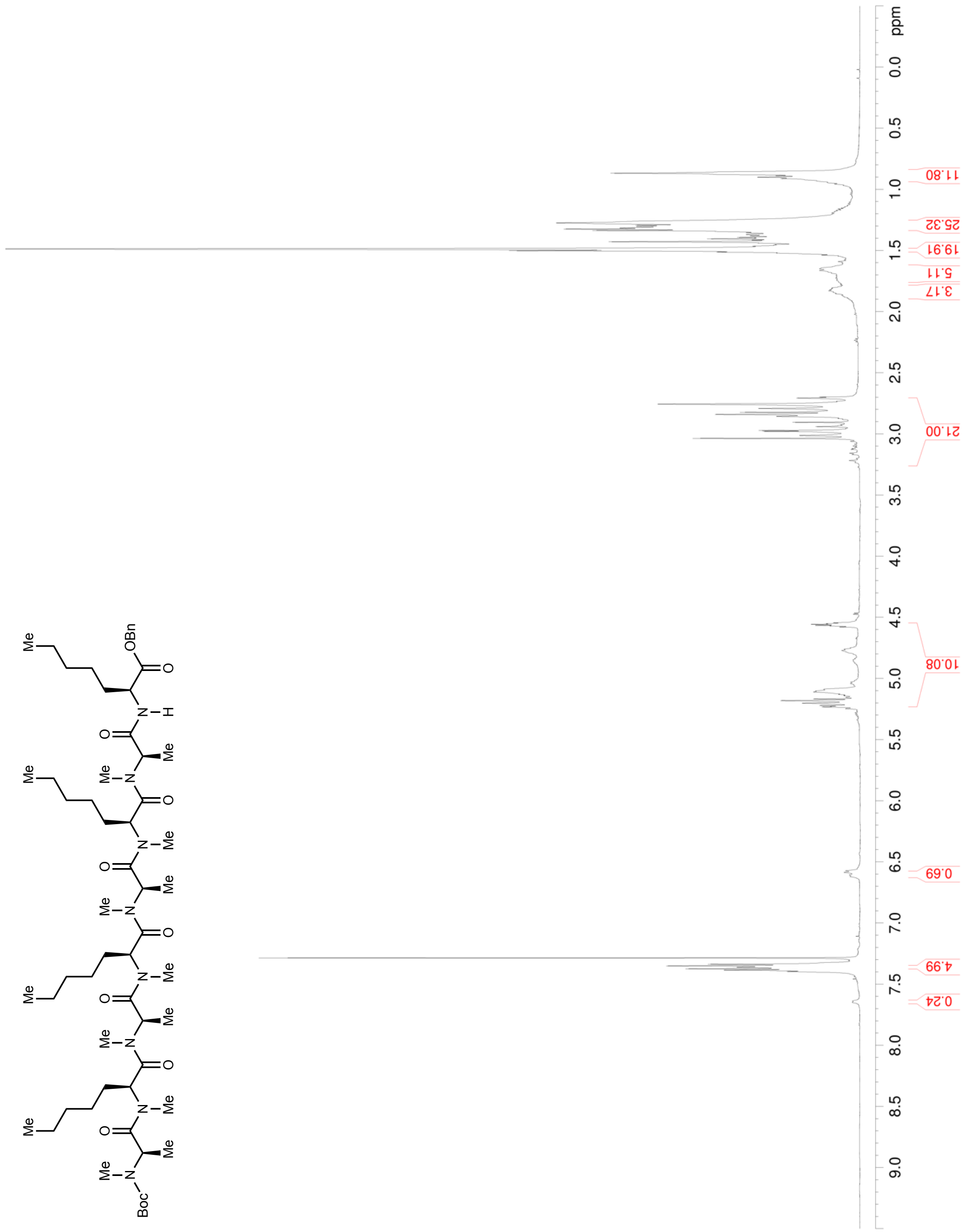

Figure S78. ^{13}C NMR (150 MHz, $CDCl_3$) of **O71**

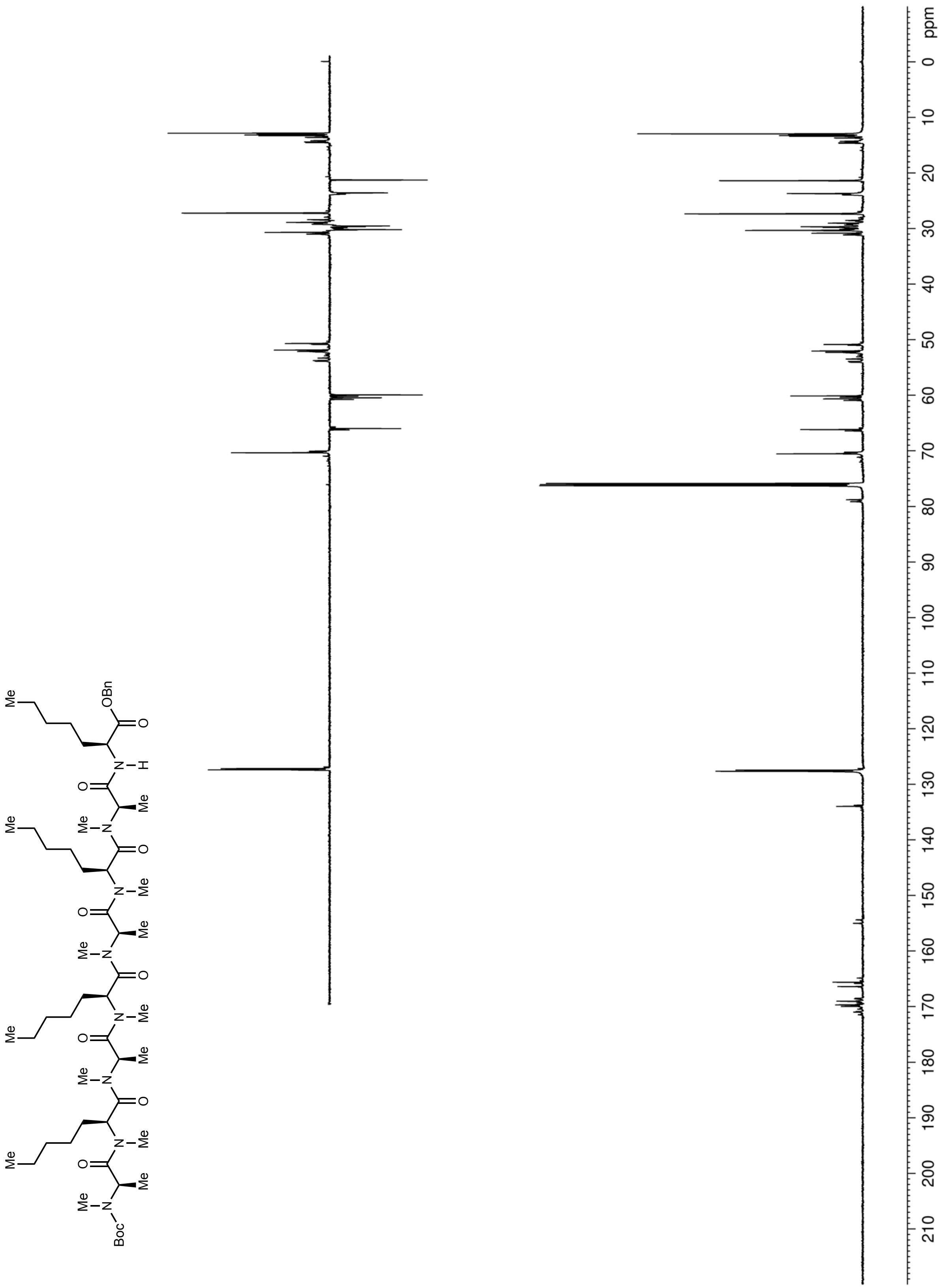

Figure S79. ^{1}H NMR (600 MHz, CD_3COCD_3) of **O72**

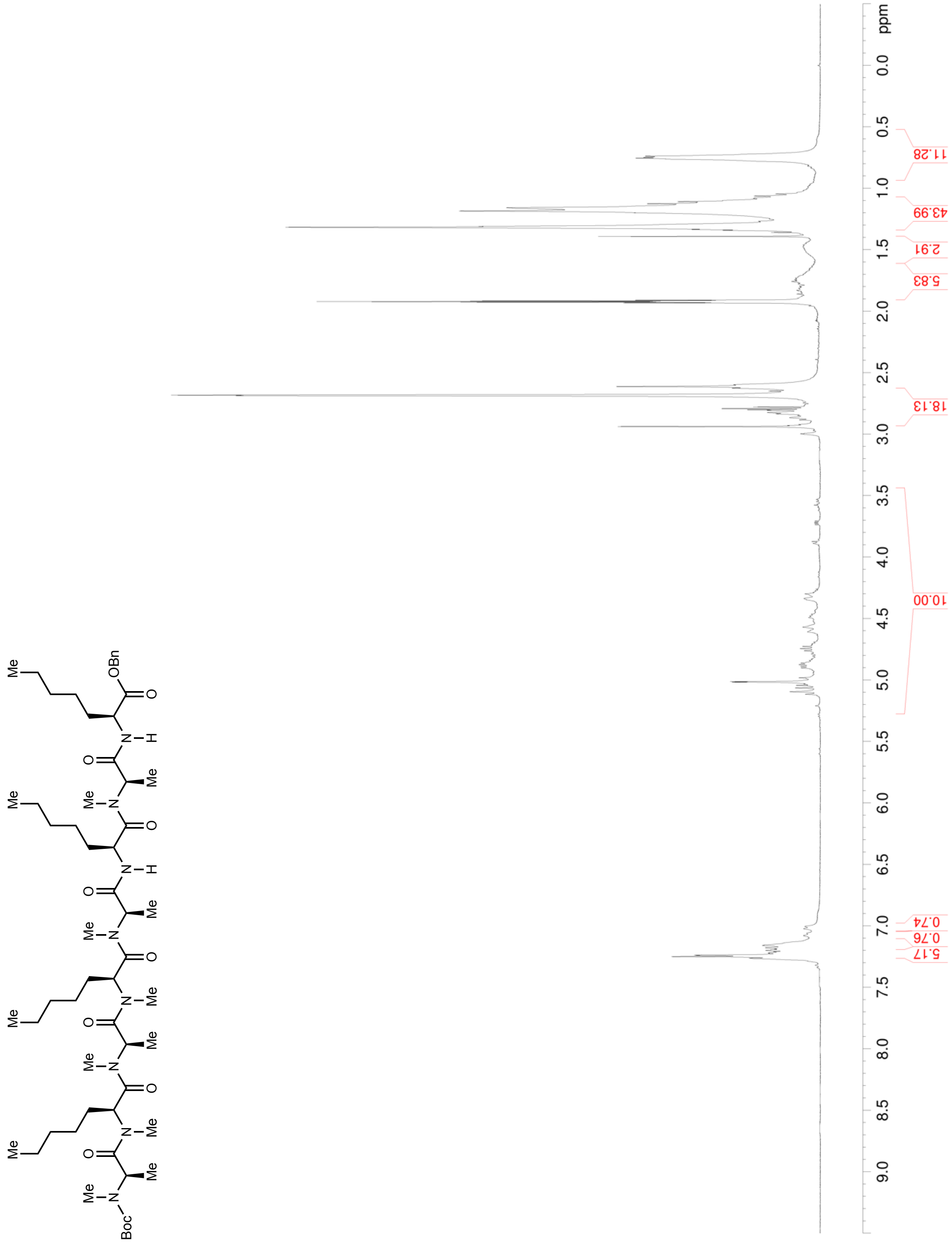

Figure S80. ^{13}C NMR (150 MHz, $CDCl_3$) of **O72**

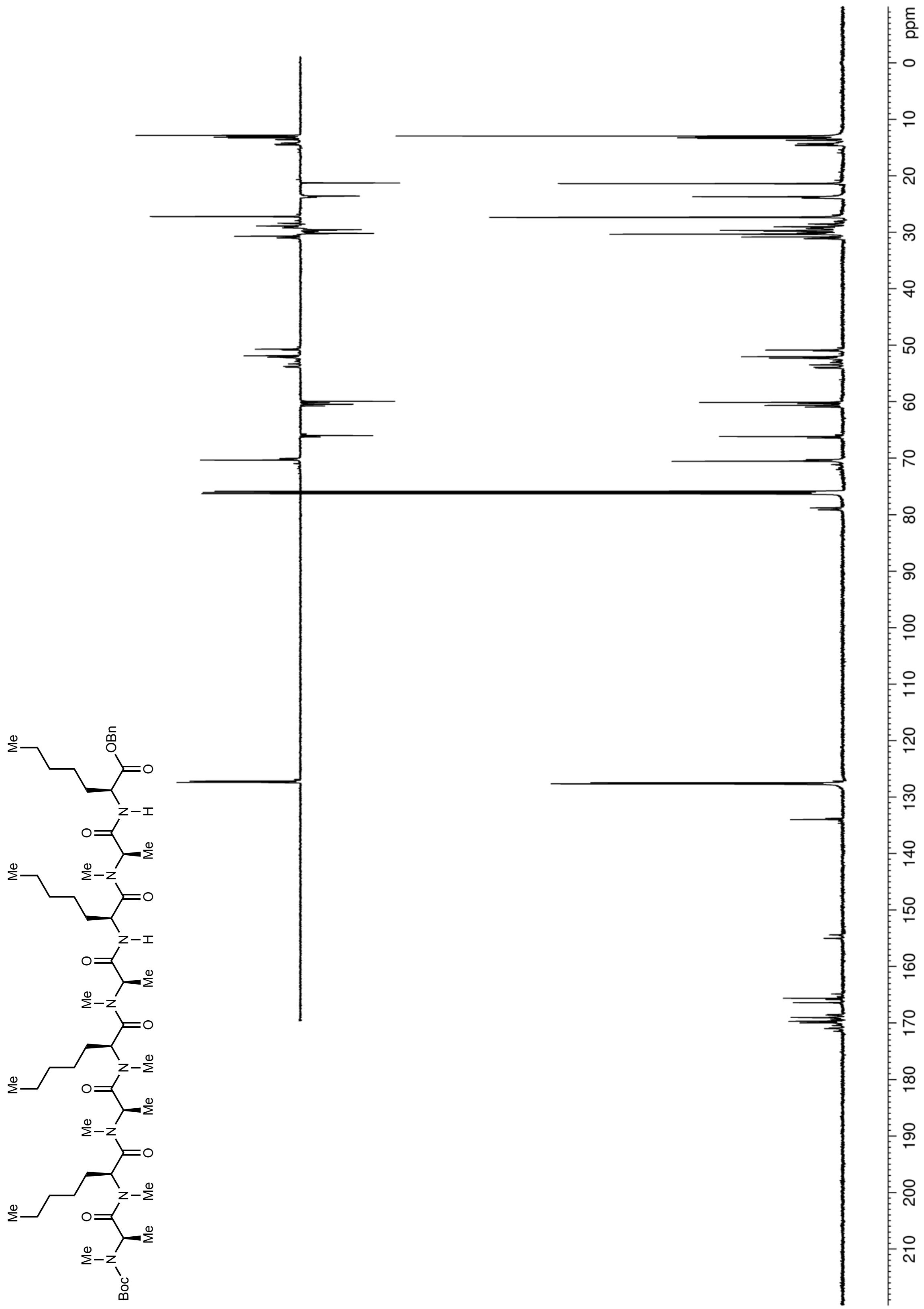

Figure S81. ¹H NMR (600 MHz, $CDCl_3$) of **O73**

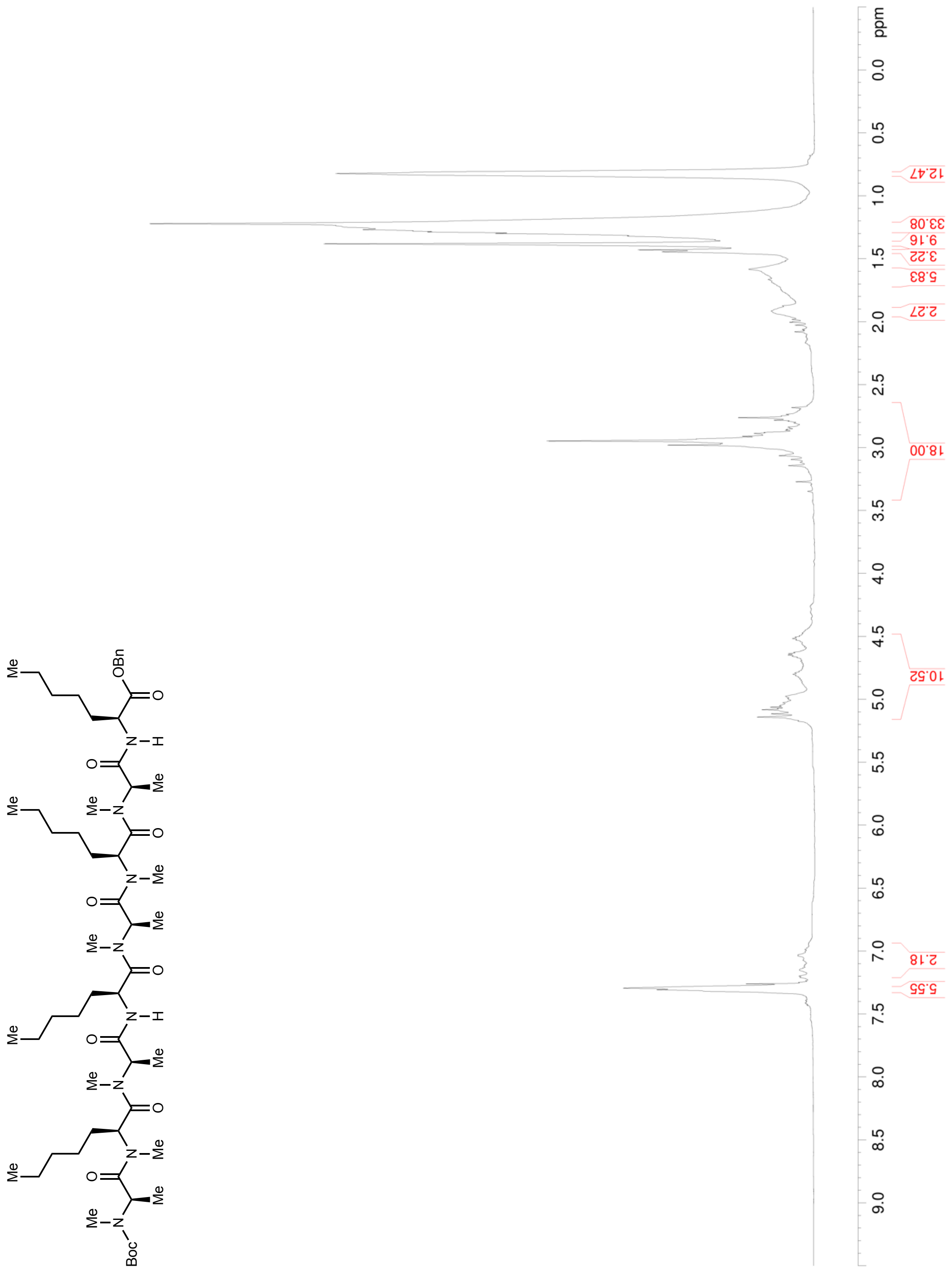

Figure S82. ^{13}C NMR (150 MHz, $CDCl_3$) of **O73**

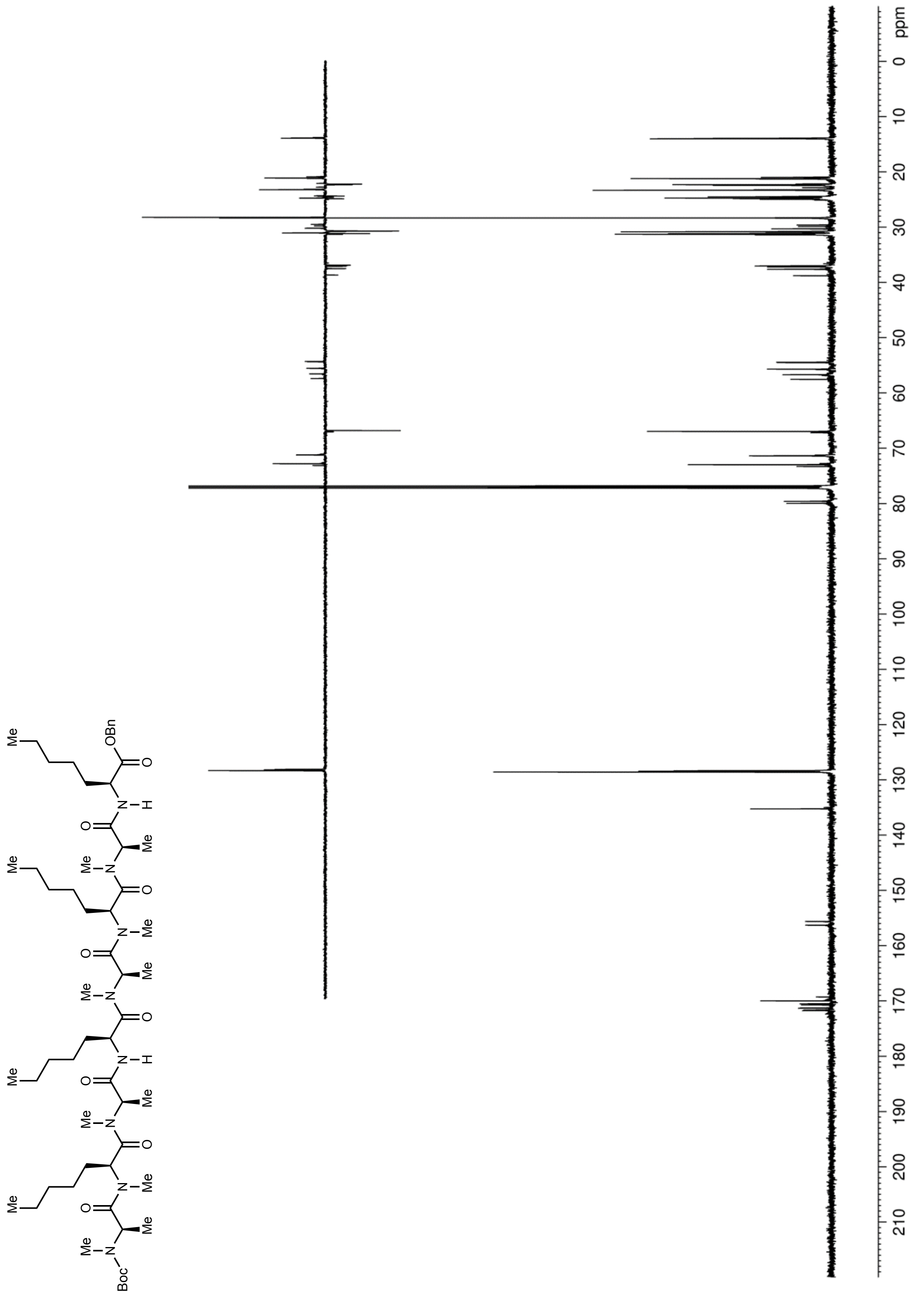

Figure S83. ^{1}H NMR (600 MHz, $CDCl_3$) of **O74**

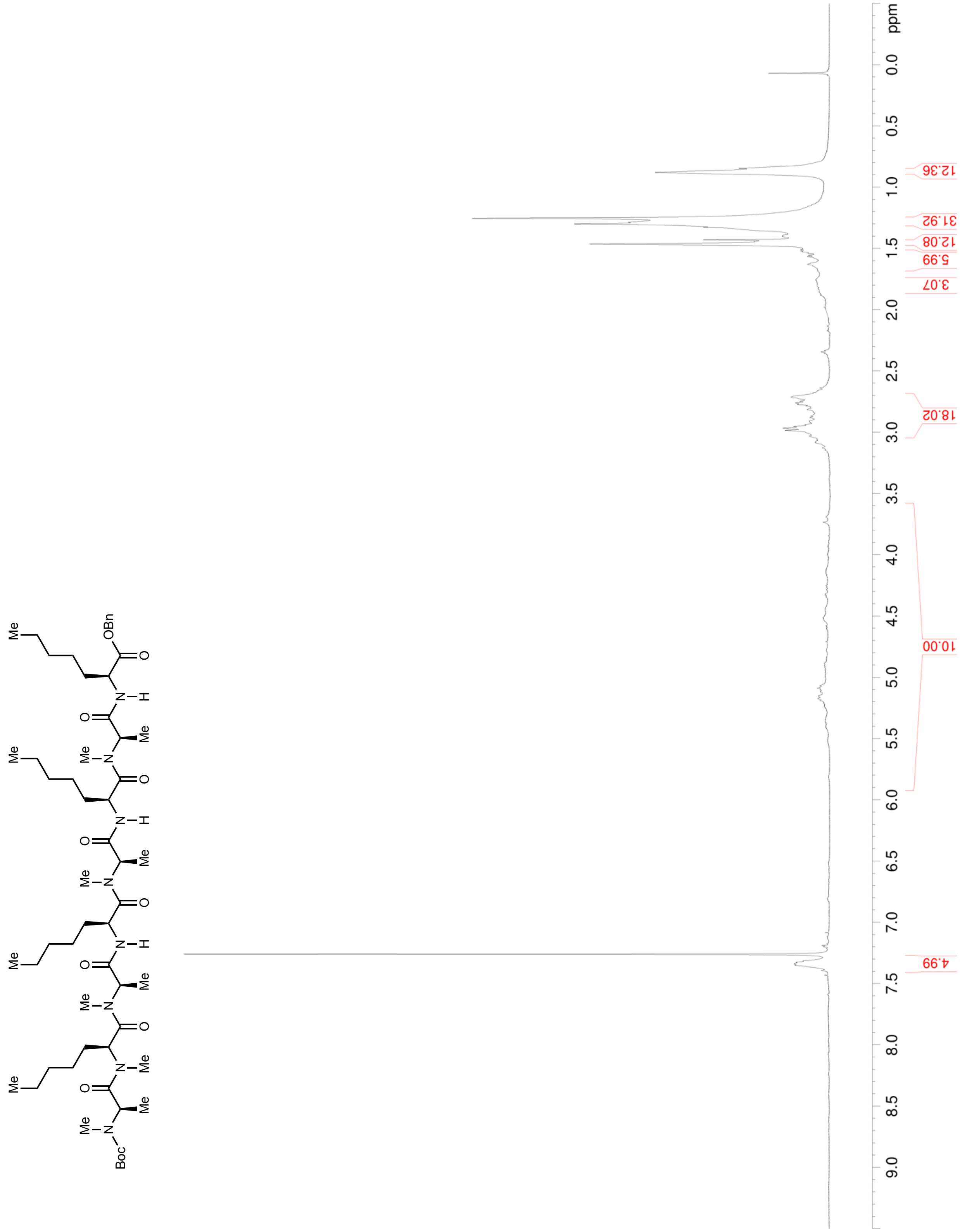

Figure S84. ^{13}C NMR (150 MHz, $CDCl_3$) of **O74**

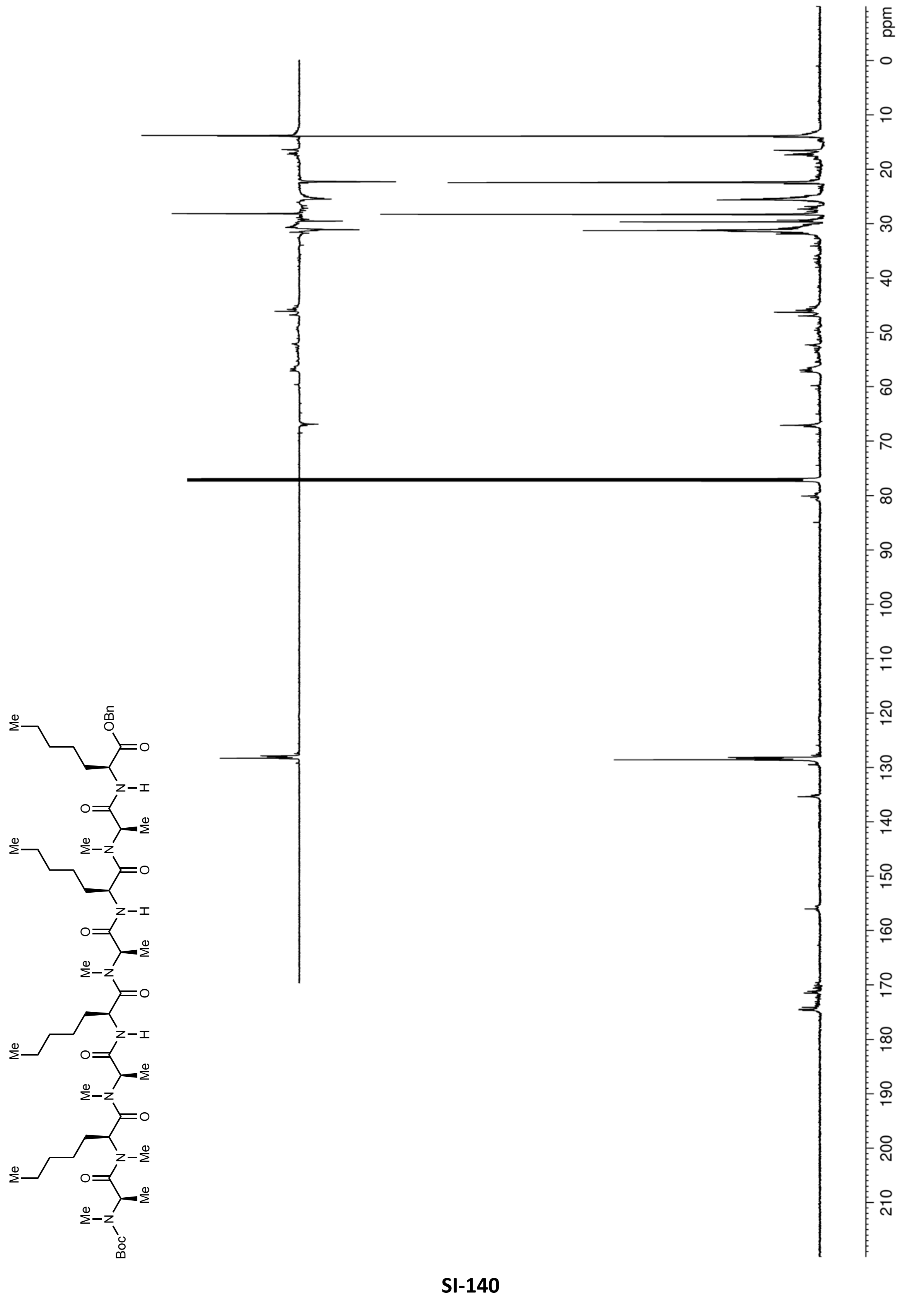

Figure S85. ^{1}H NMR (600 MHz, $CDCl_3$) of **1.1**

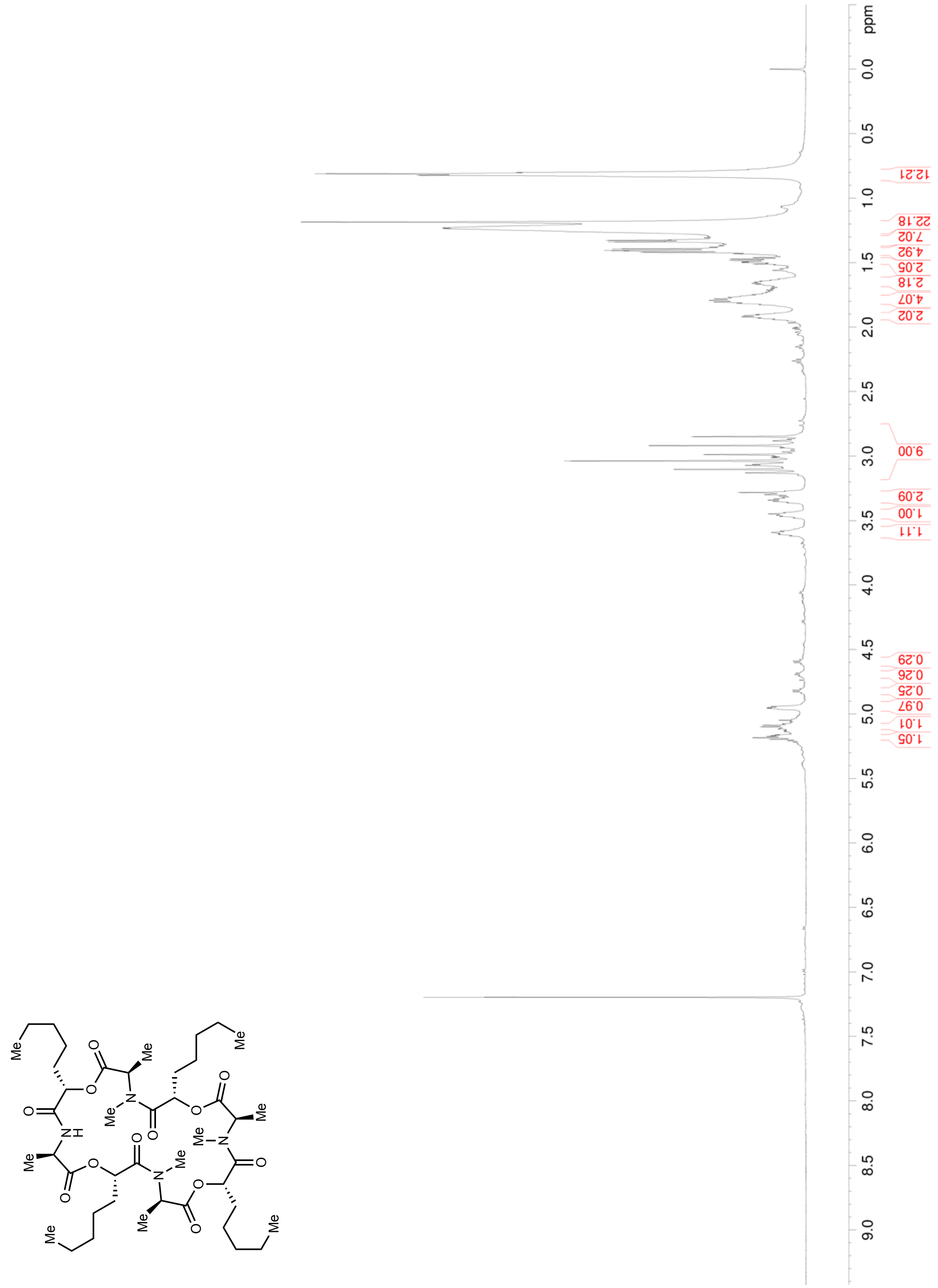

Figure S86. ^{13}C NMR (150 MHz, $CDCl_3$) of **1.1**

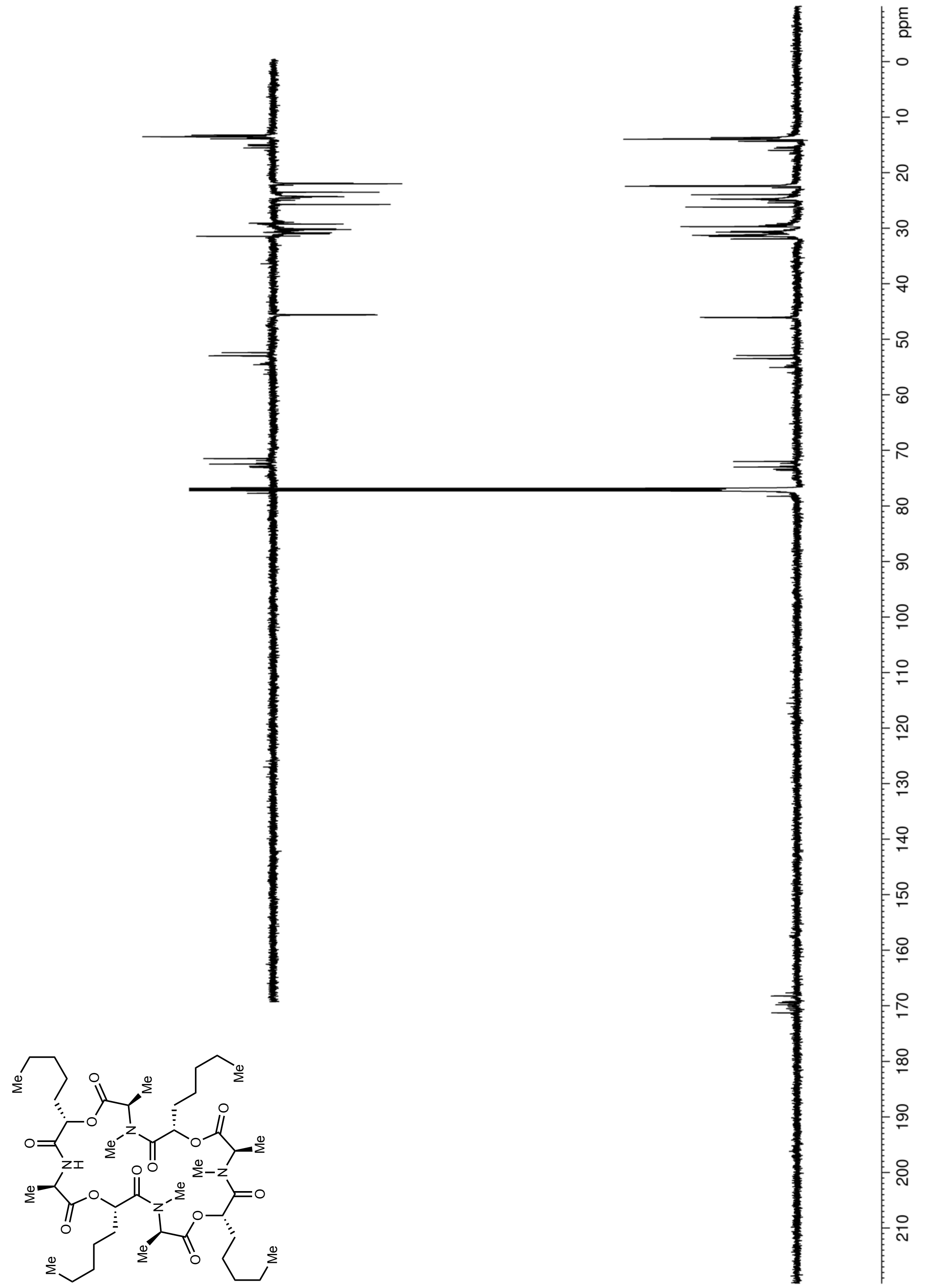

Figure S87. ^{1}H NMR (600 MHz, $CDCl_3$) of **1.2**

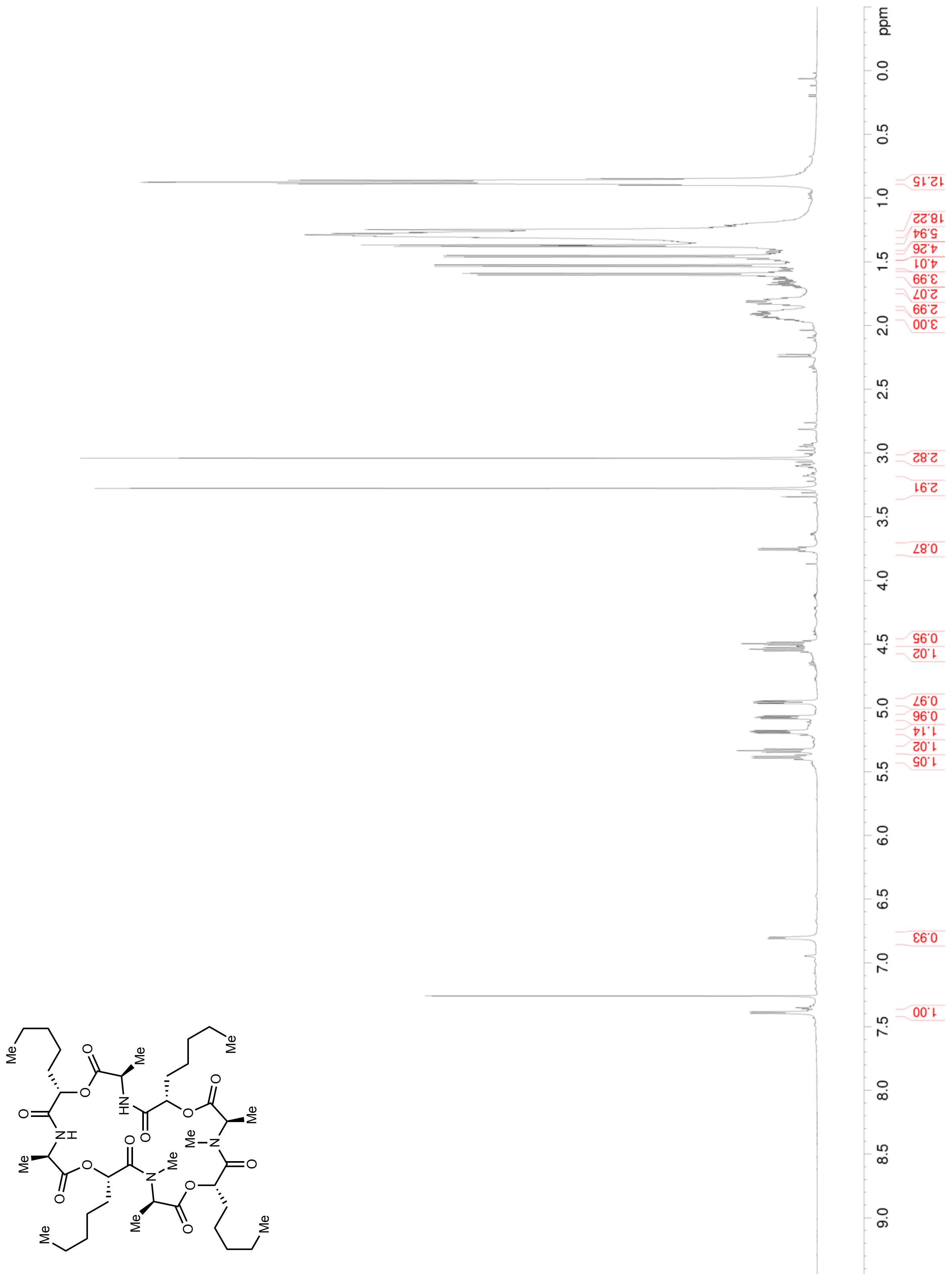

Figure S88. ^{13}C NMR (150 MHz, $CDCl_3$) of **1.2**

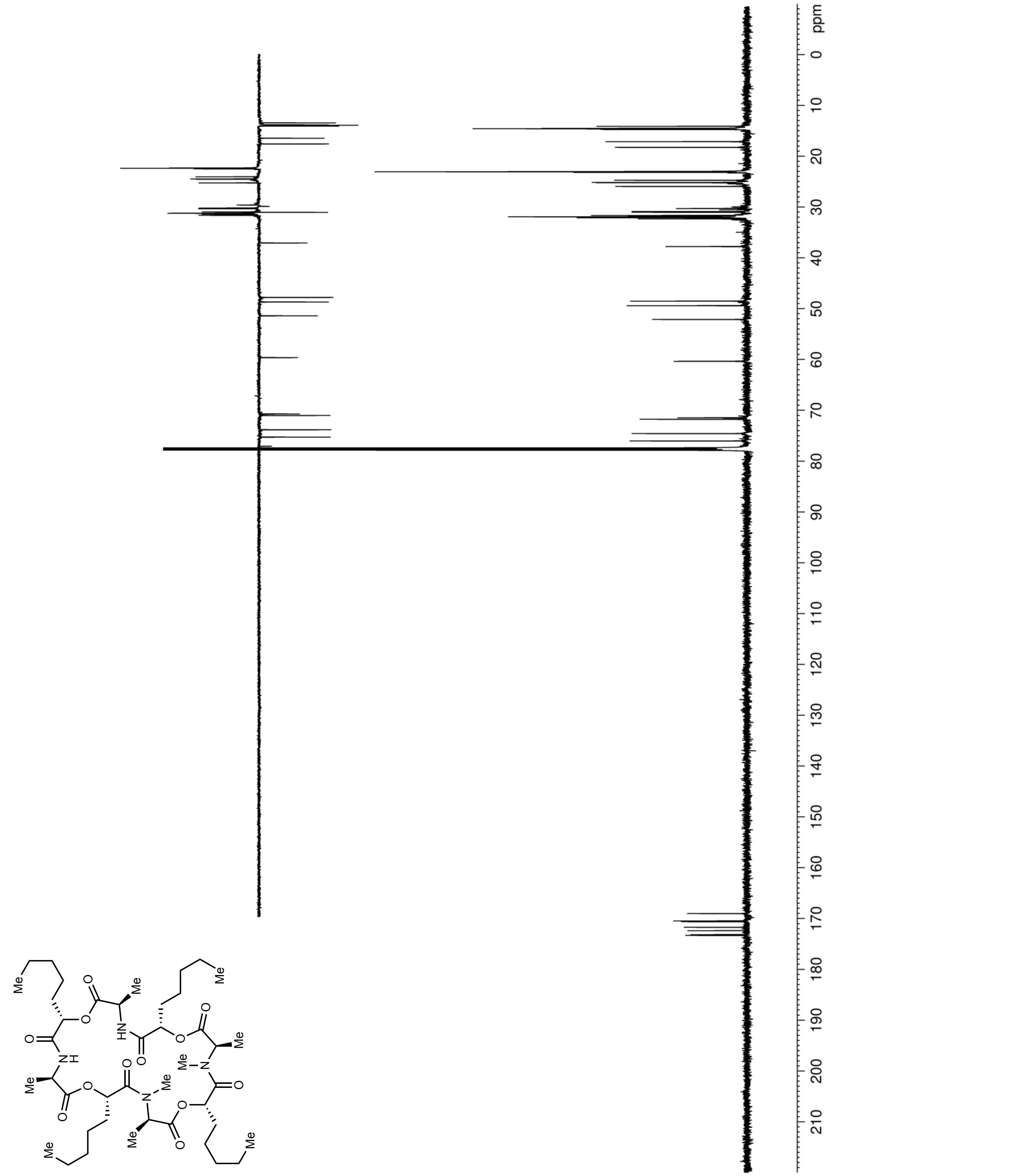

Figure S89. ^{1}H NMR (600 MHz, $CDCl_3$) of **1.3**

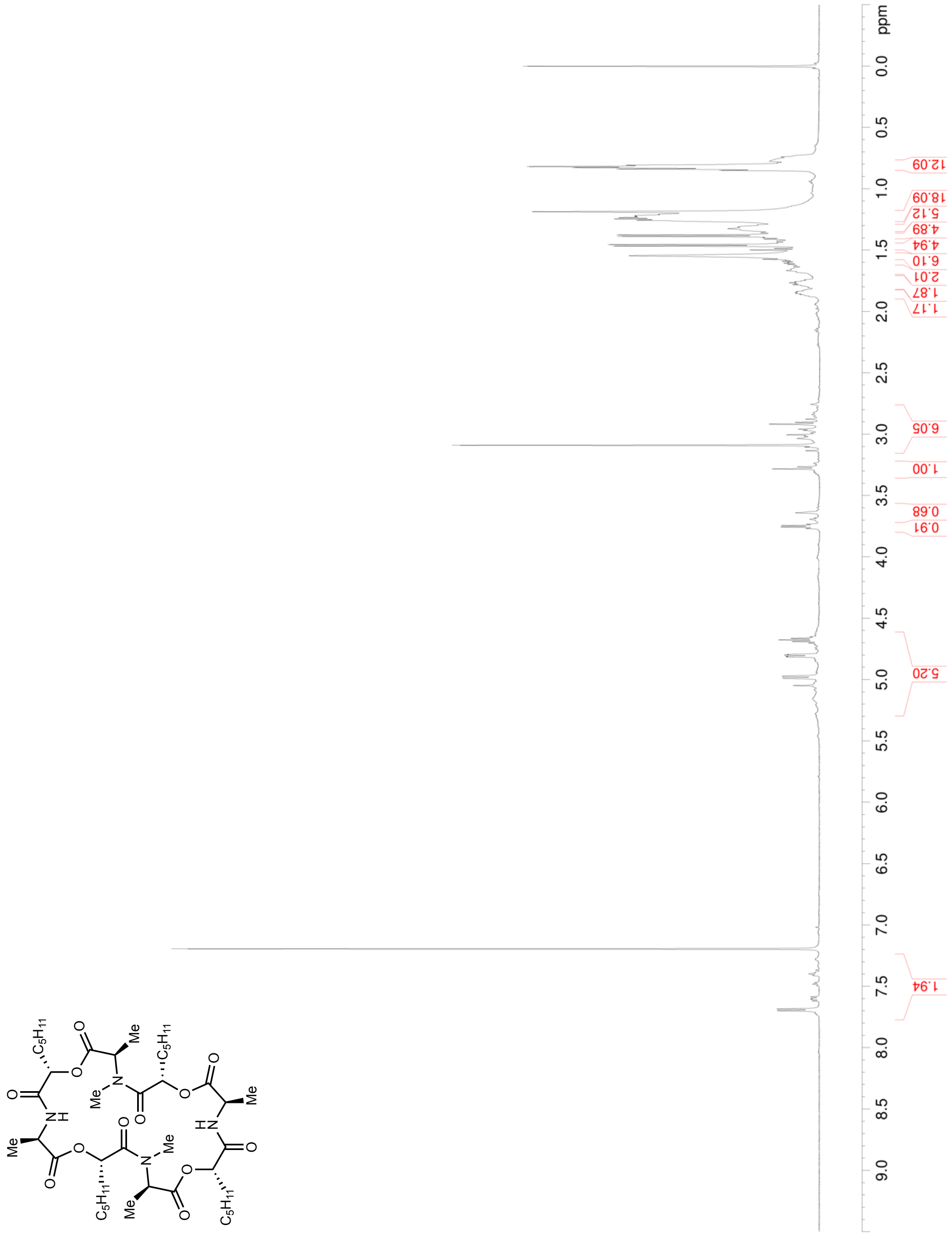

Figure S90. ^{13}C NMR (150 MHz, $CDCl_3$) of **1.3**

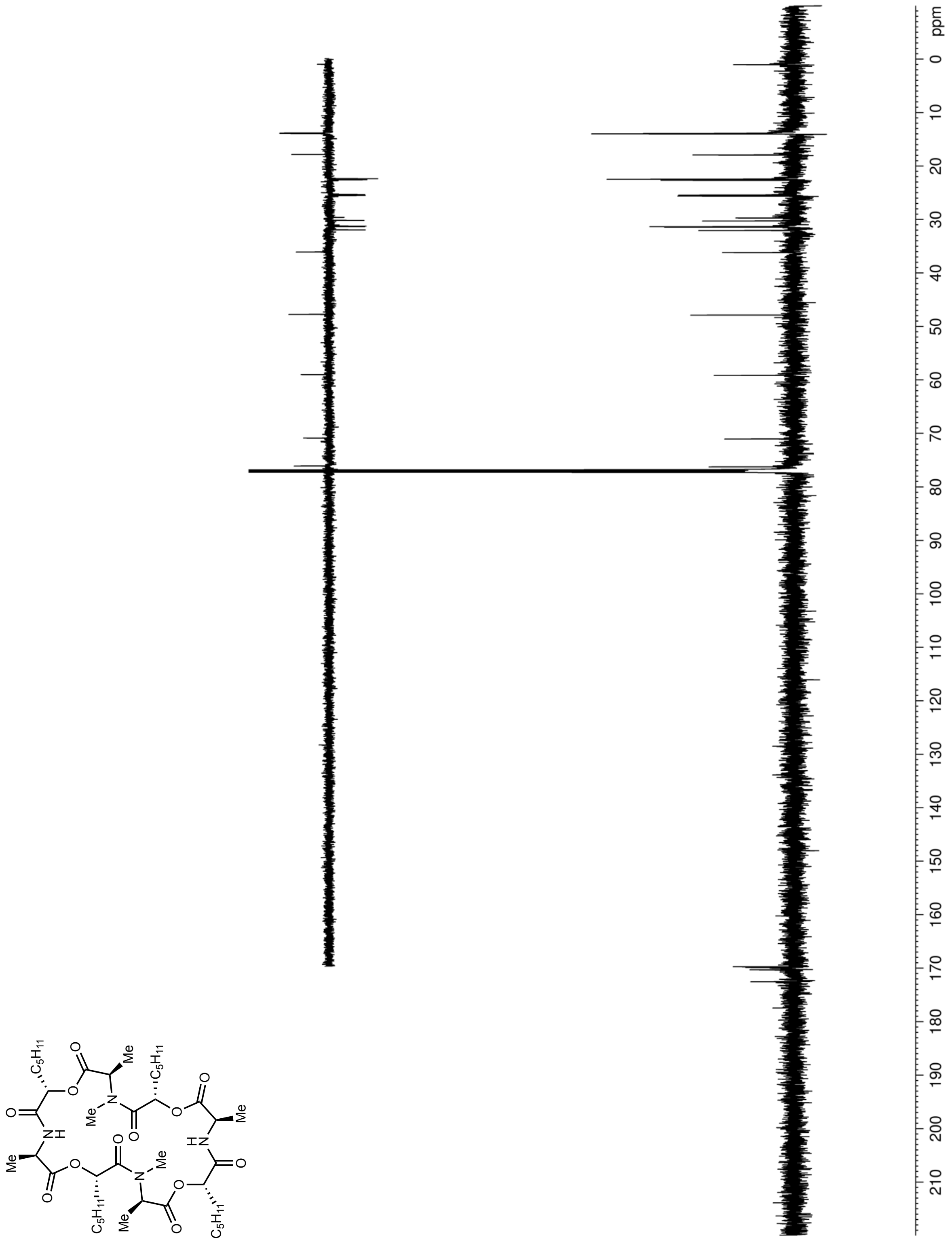

Figure S91. ^{1}H NMR (600 MHz, $CDCl_3$) of **1.4**

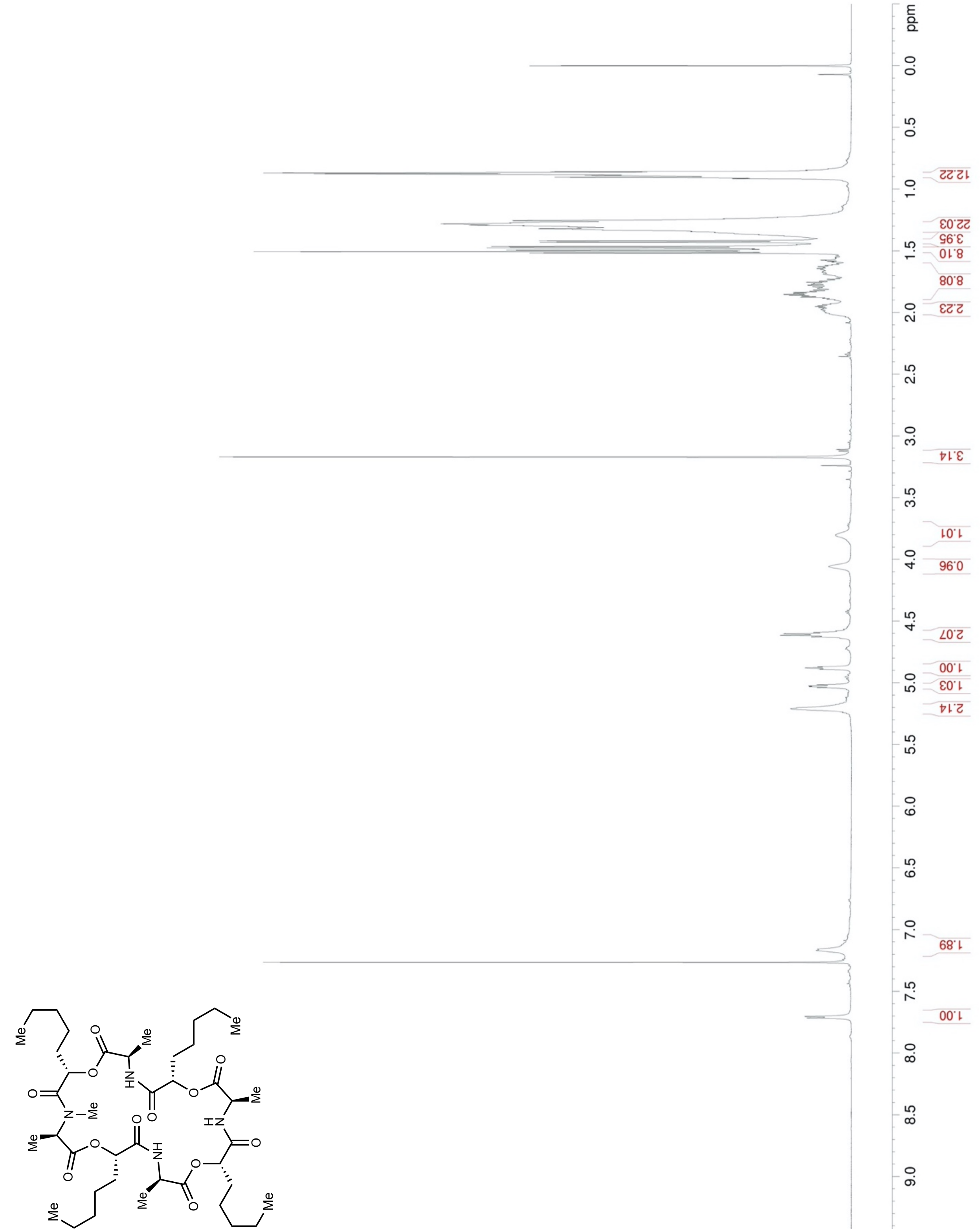

Figure S92. ^{13}C NMR (150 MHz, $CDCl_3$) of **1.4**

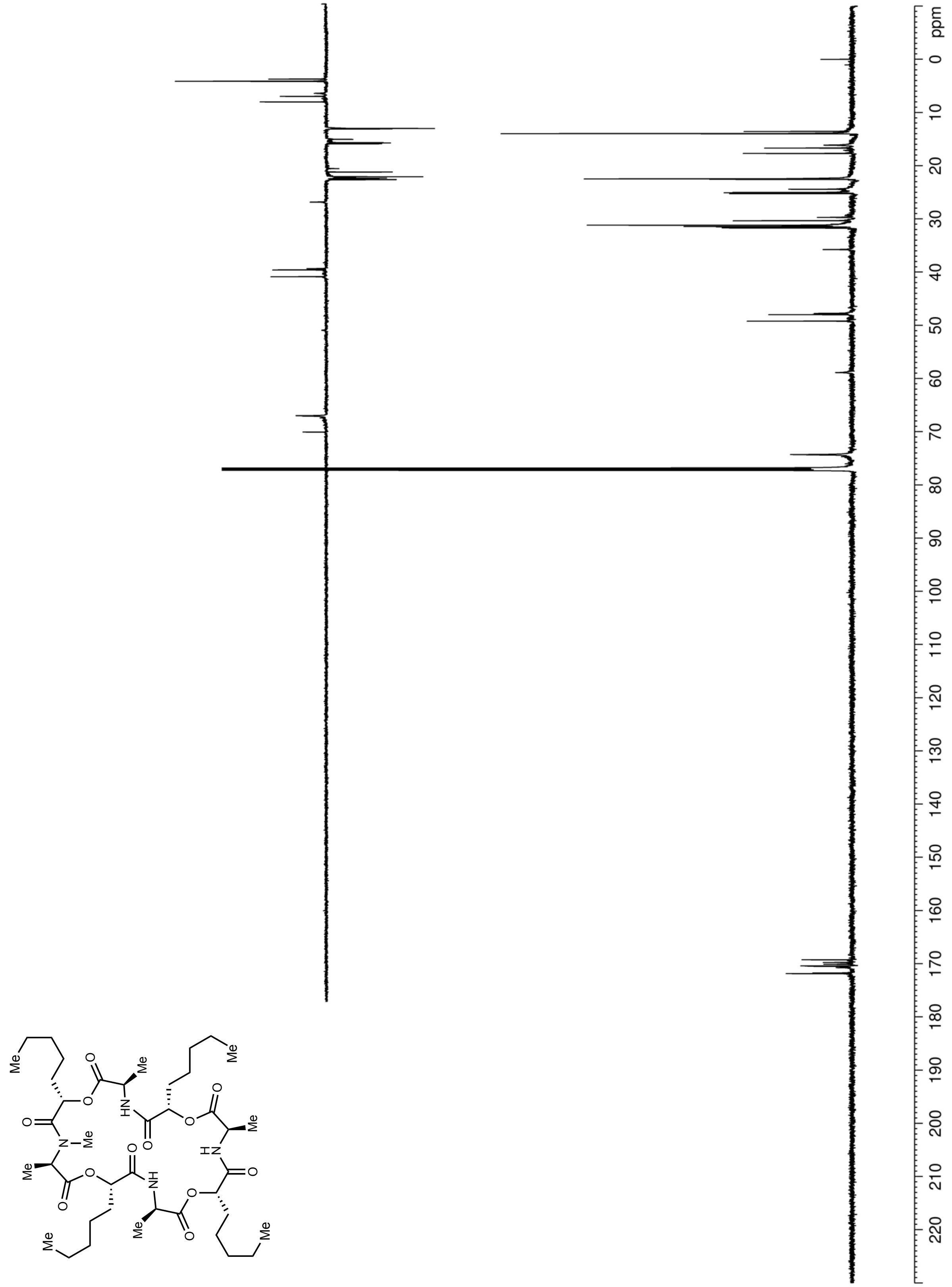

Figure S93. ^{1}H NMR (600 MHz, CD_3COCD_3) of **2.1**

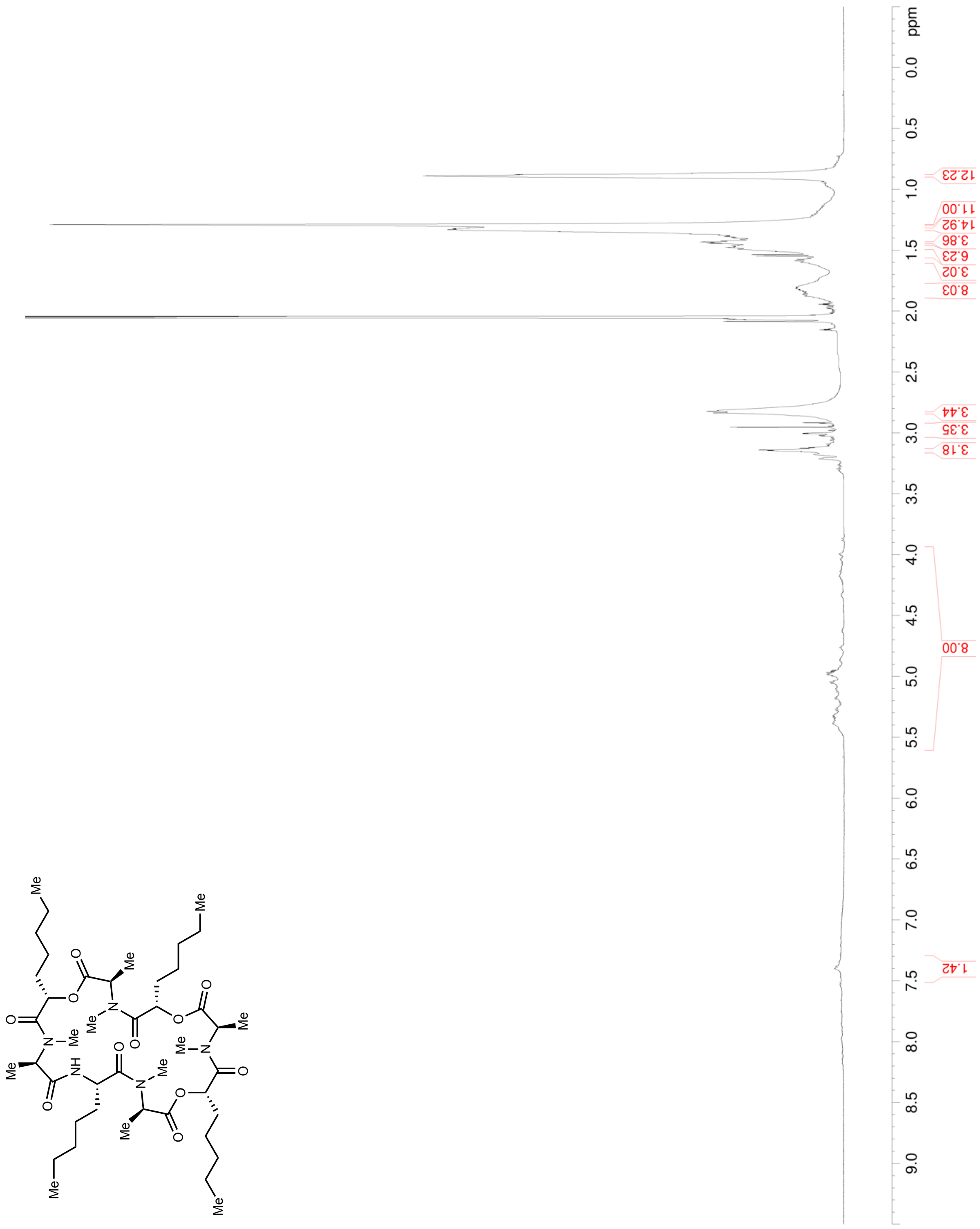

Figure S94. ^{13}C NMR (150 MHz, $CDCl_3$) of **2.1**

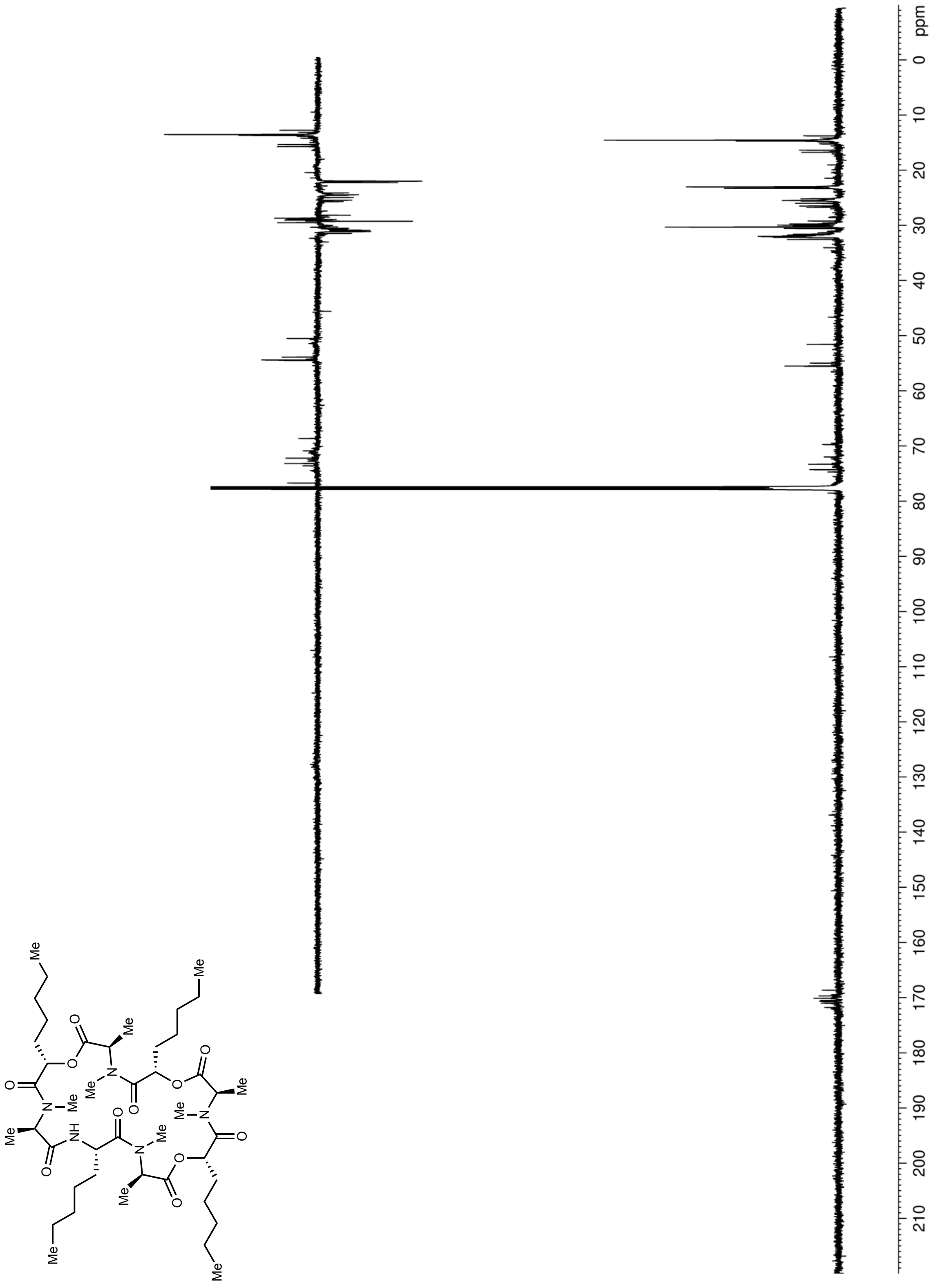

Figure S95. ^{1}H NMR (600 MHz, $CDCl_3$) of **2.2**

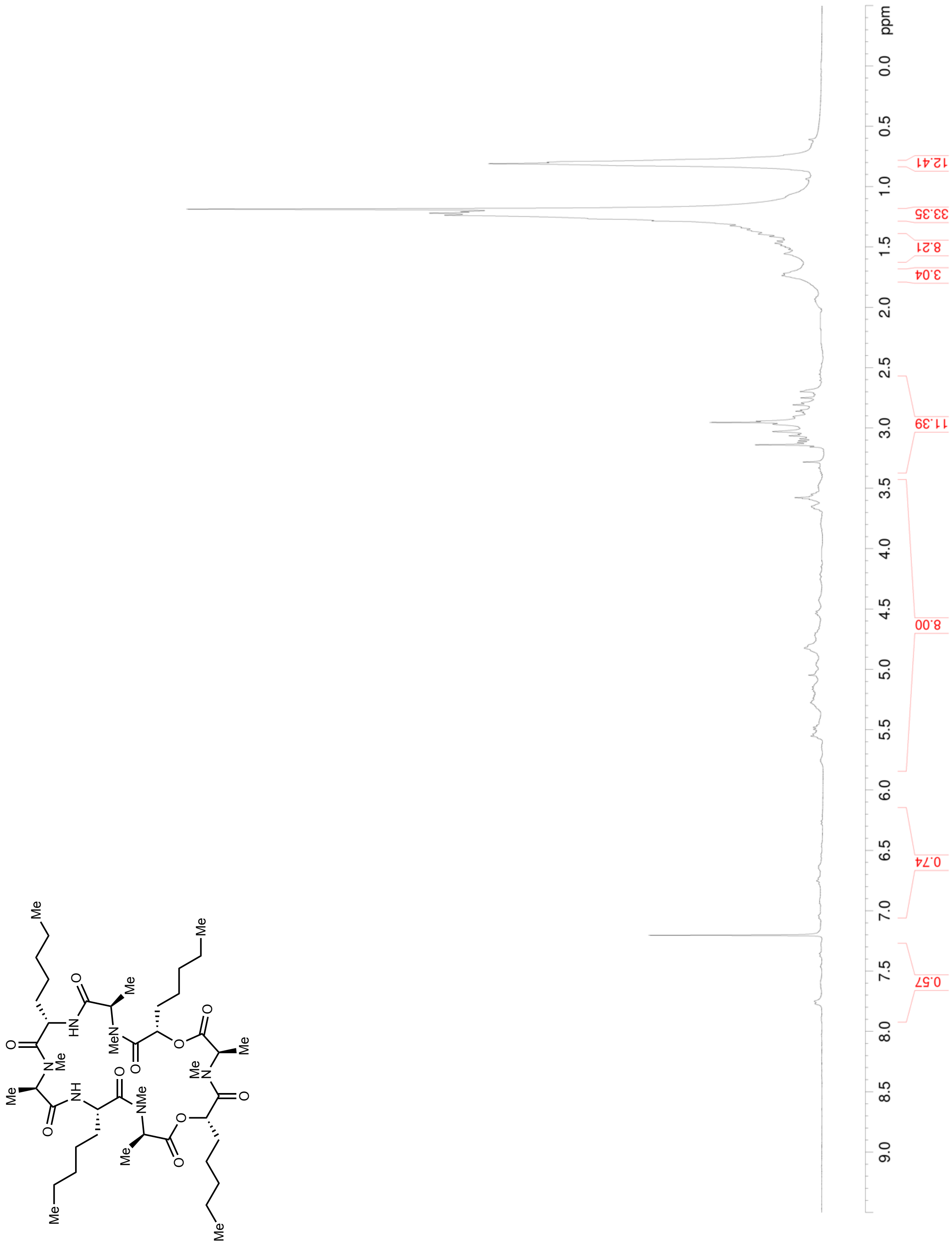

Figure S96. ^{13}C NMR (150 MHz, $CDCl_3$) of **2.2**

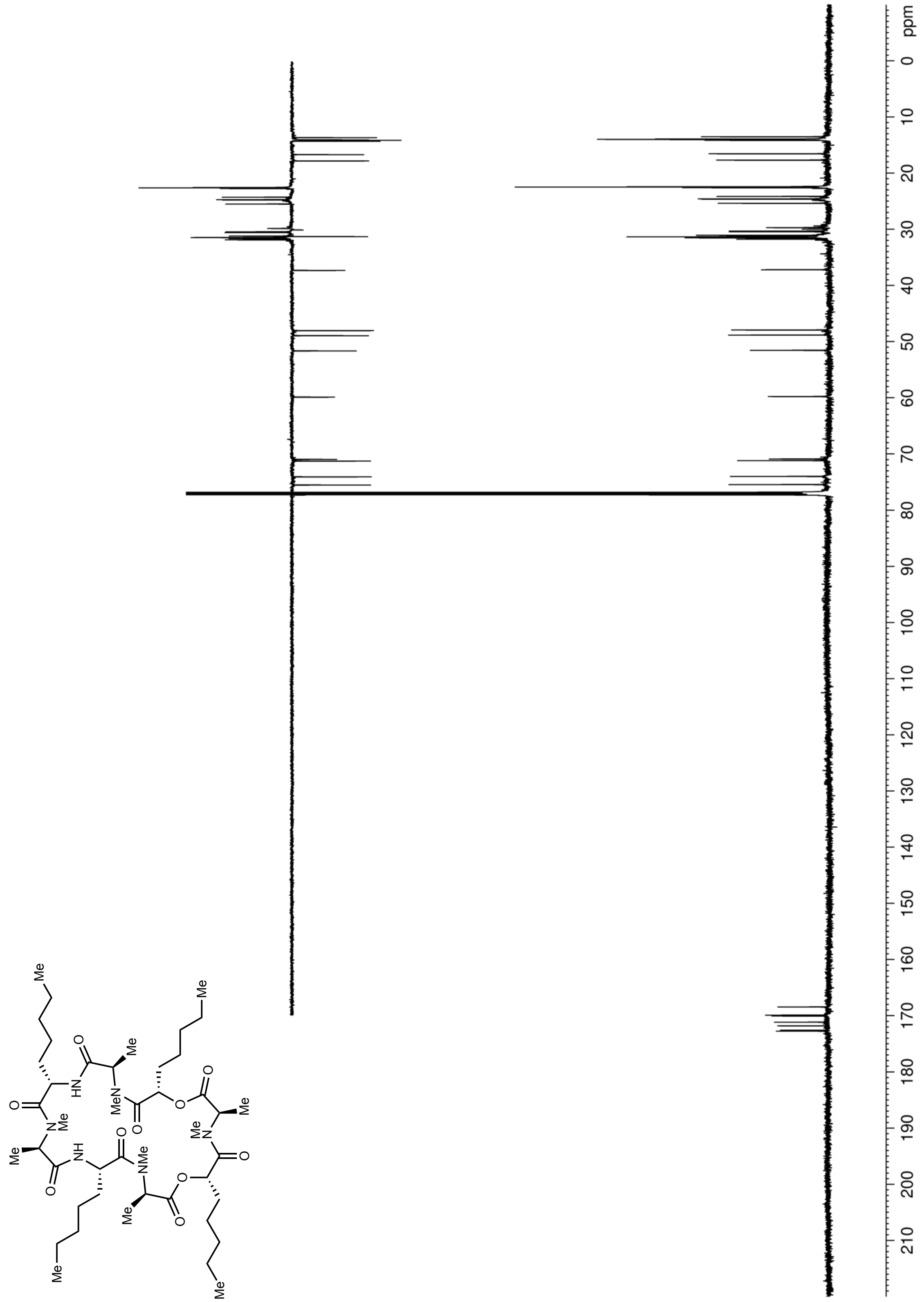

Figure S97. ^{1}H NMR (600 MHz, $CDCl_3$) of **2.3**

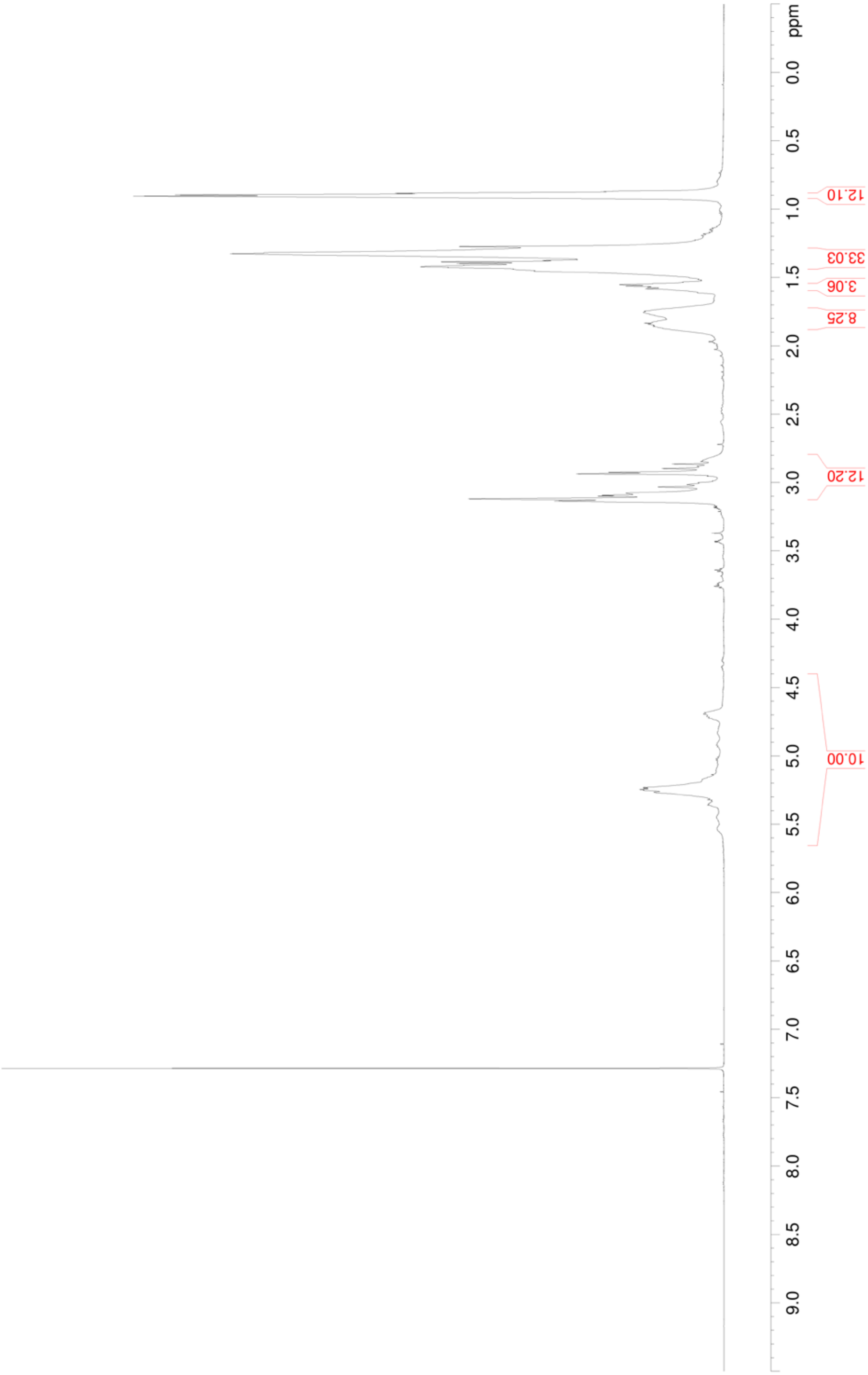

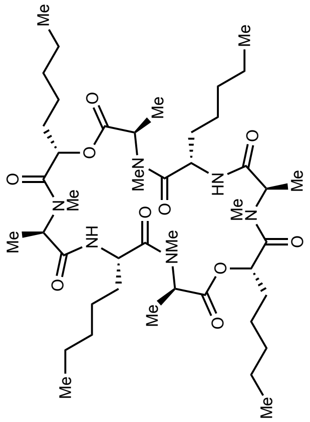

Figure S98. ^{13}C NMR (150 MHz, CDCl$_3$) of **2.3**

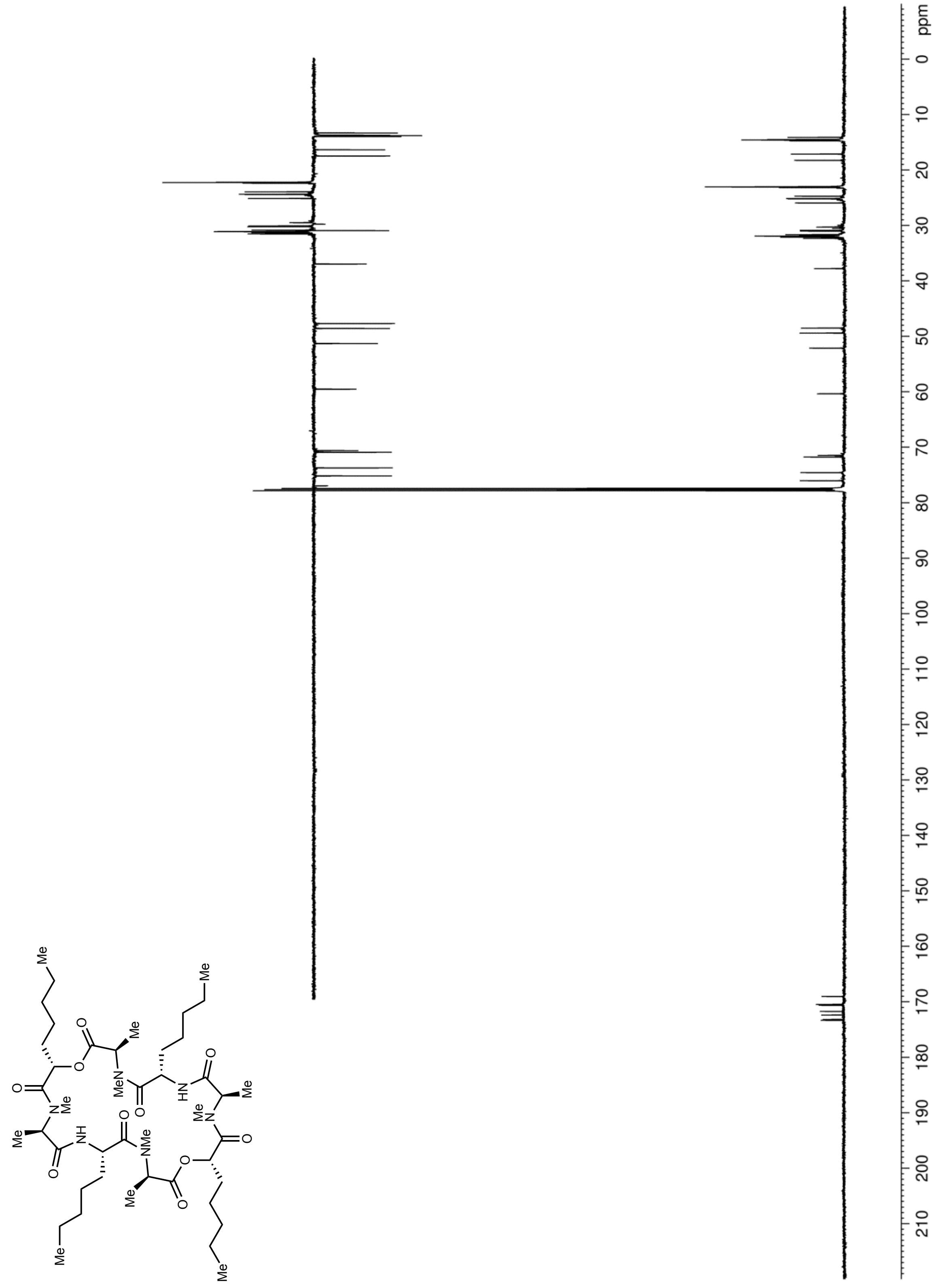

Figure S99. ^{1}H NMR (600 MHz, $CDCl_3$) of **2.4**

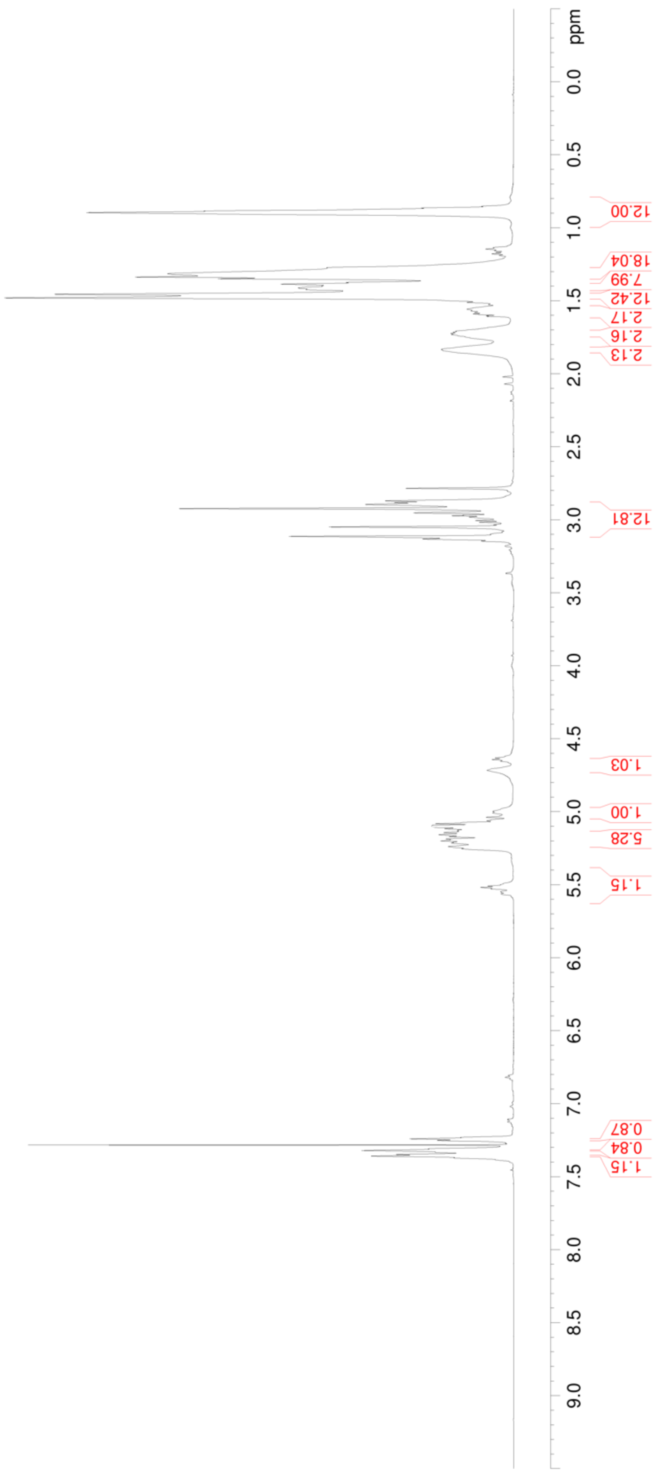

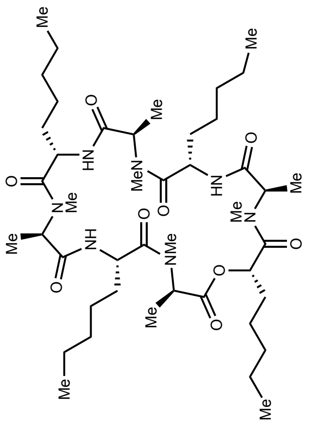

Figure S100. ^{13}C NMR (150 MHz, $CDCl_3$) of **2.4**

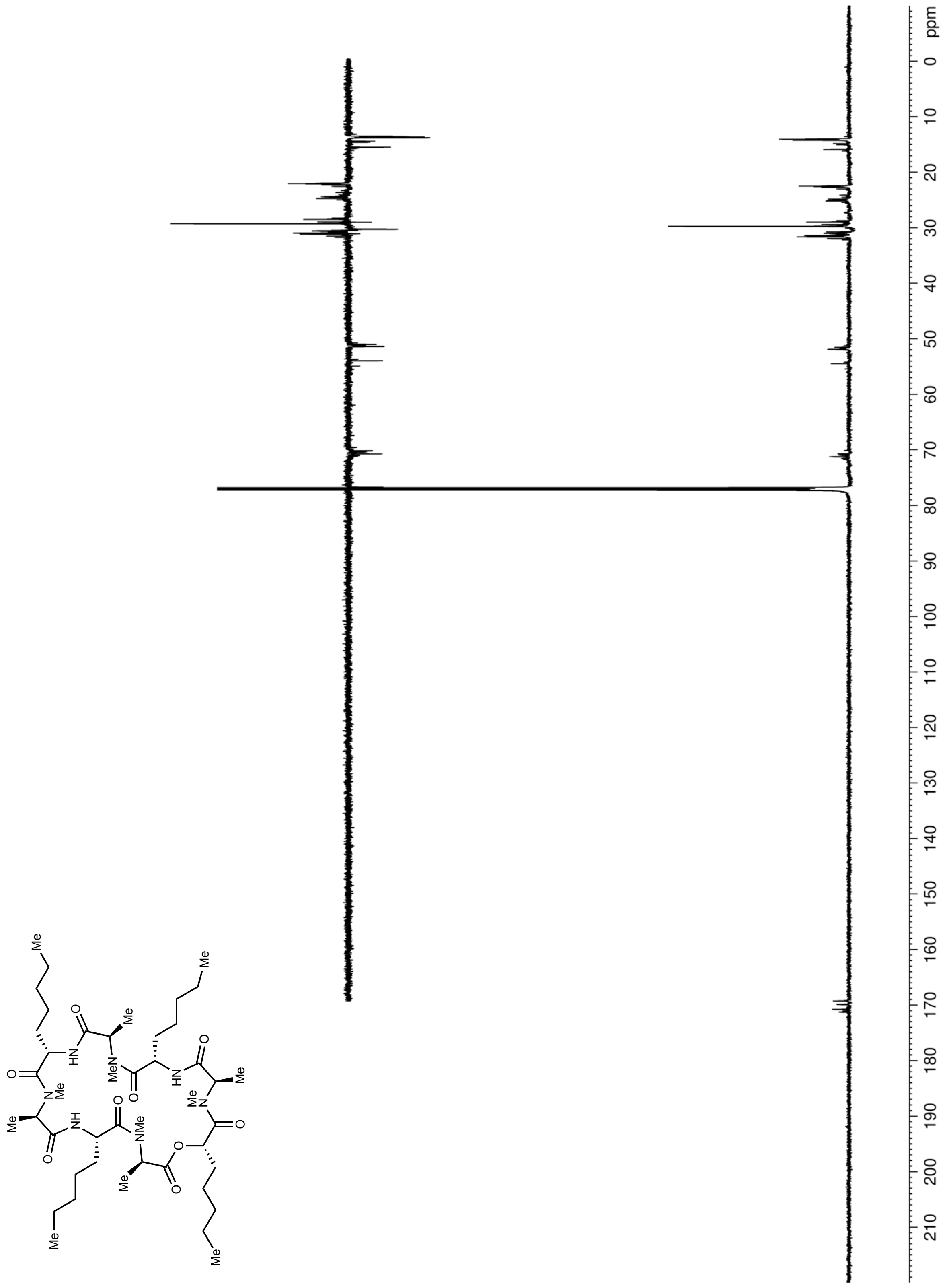

Figure S101. ^{1}H NMR (600 MHz, $CDCl_3$) of **2.5**

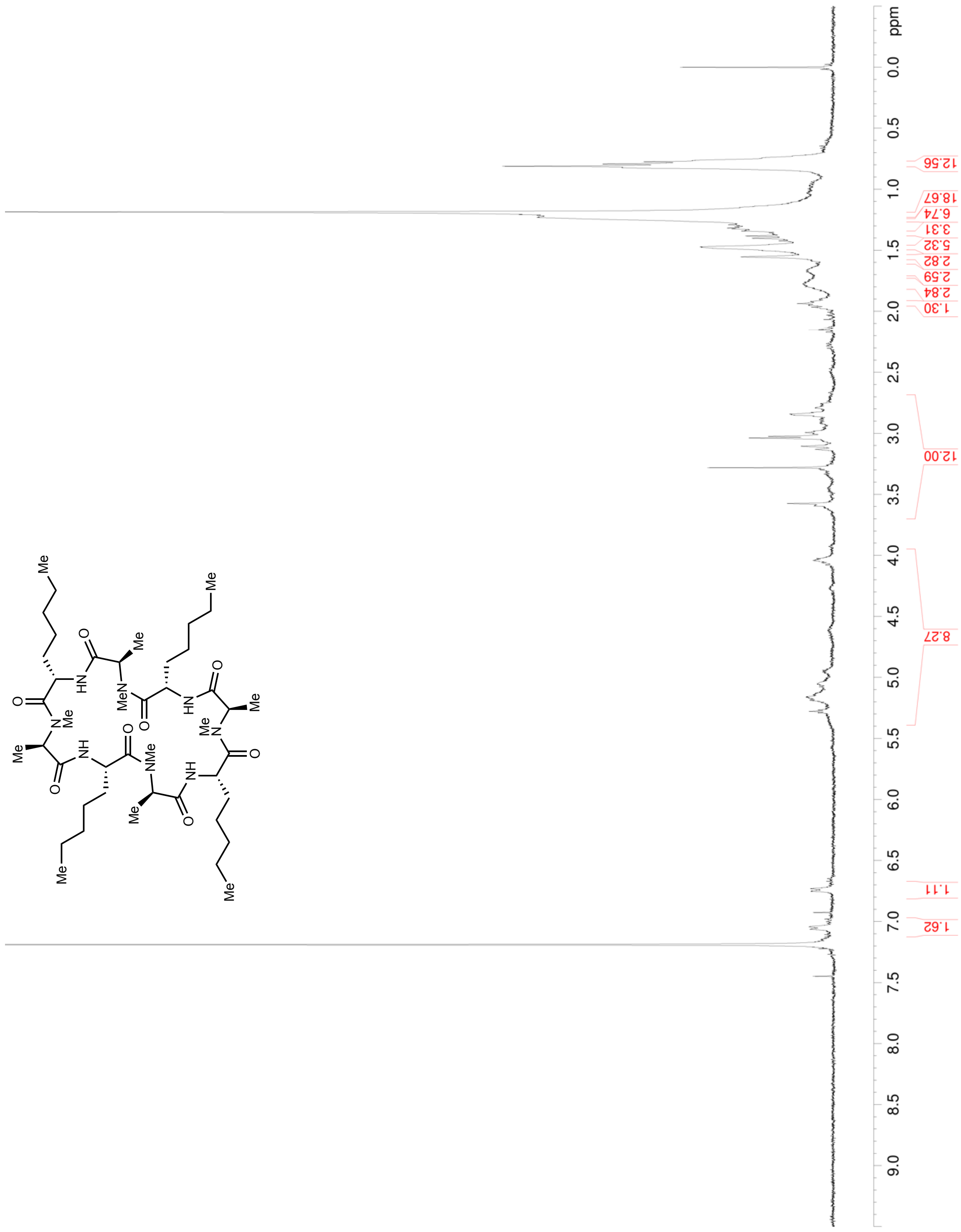

Figure S102. ^{13}C NMR (150 MHz, $CDCl_3$) of **2.5**

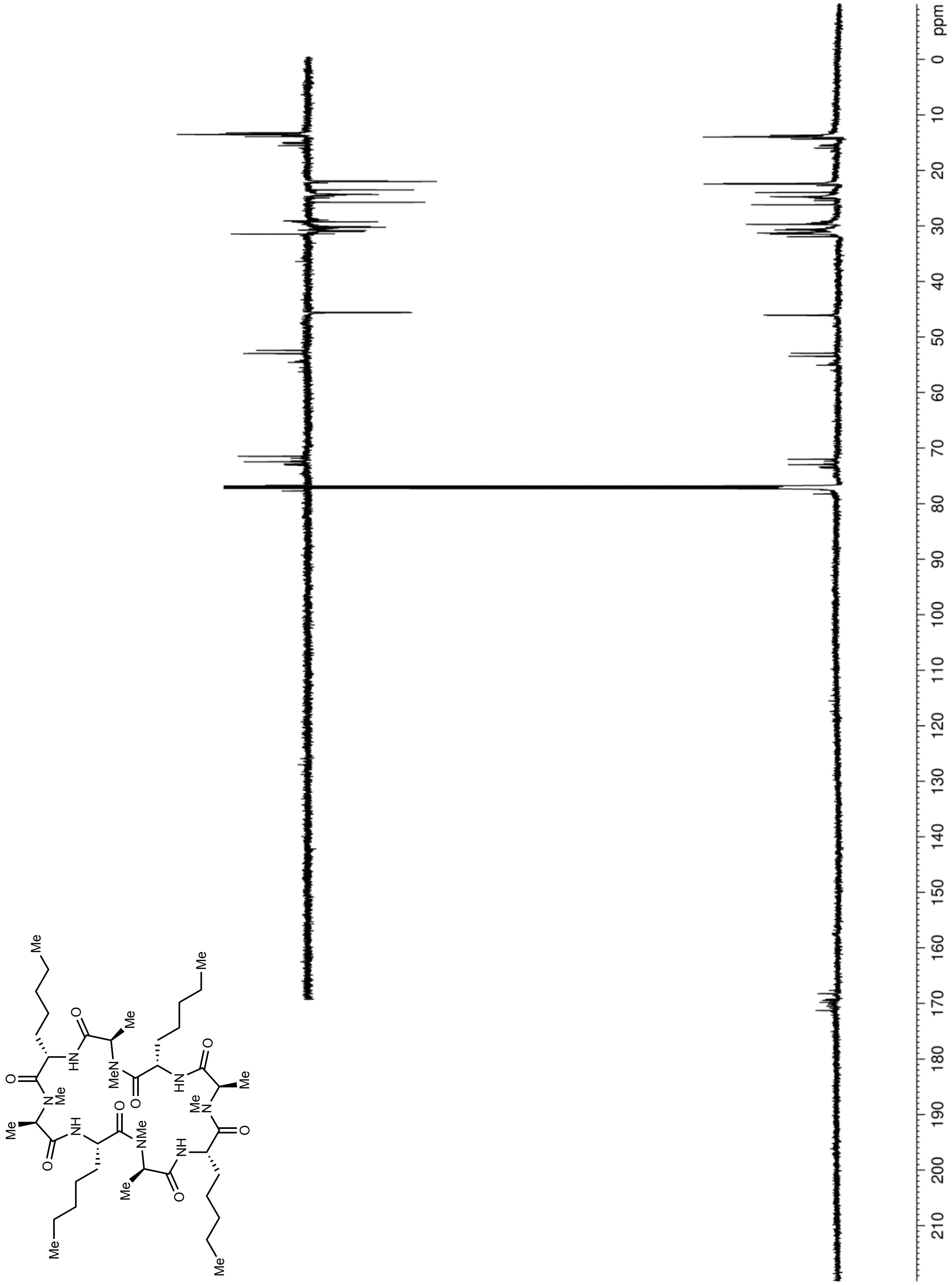

Figure S103. ^{1}H NMR (600 MHz, $CDCl_3$) of **3.1**

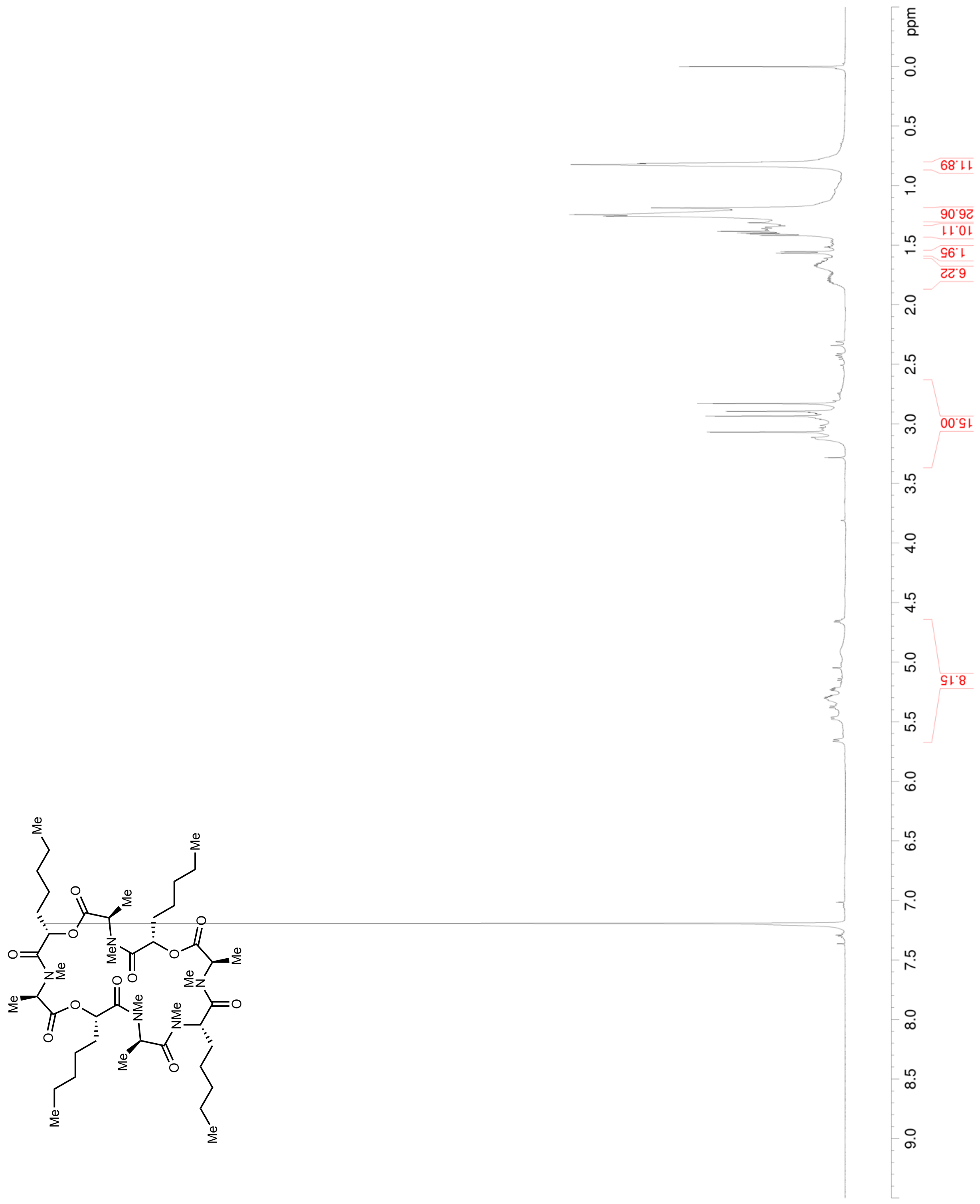

Figure S104. ^{13}C NMR (150 MHz, $CDCl_3$) of **3.1**

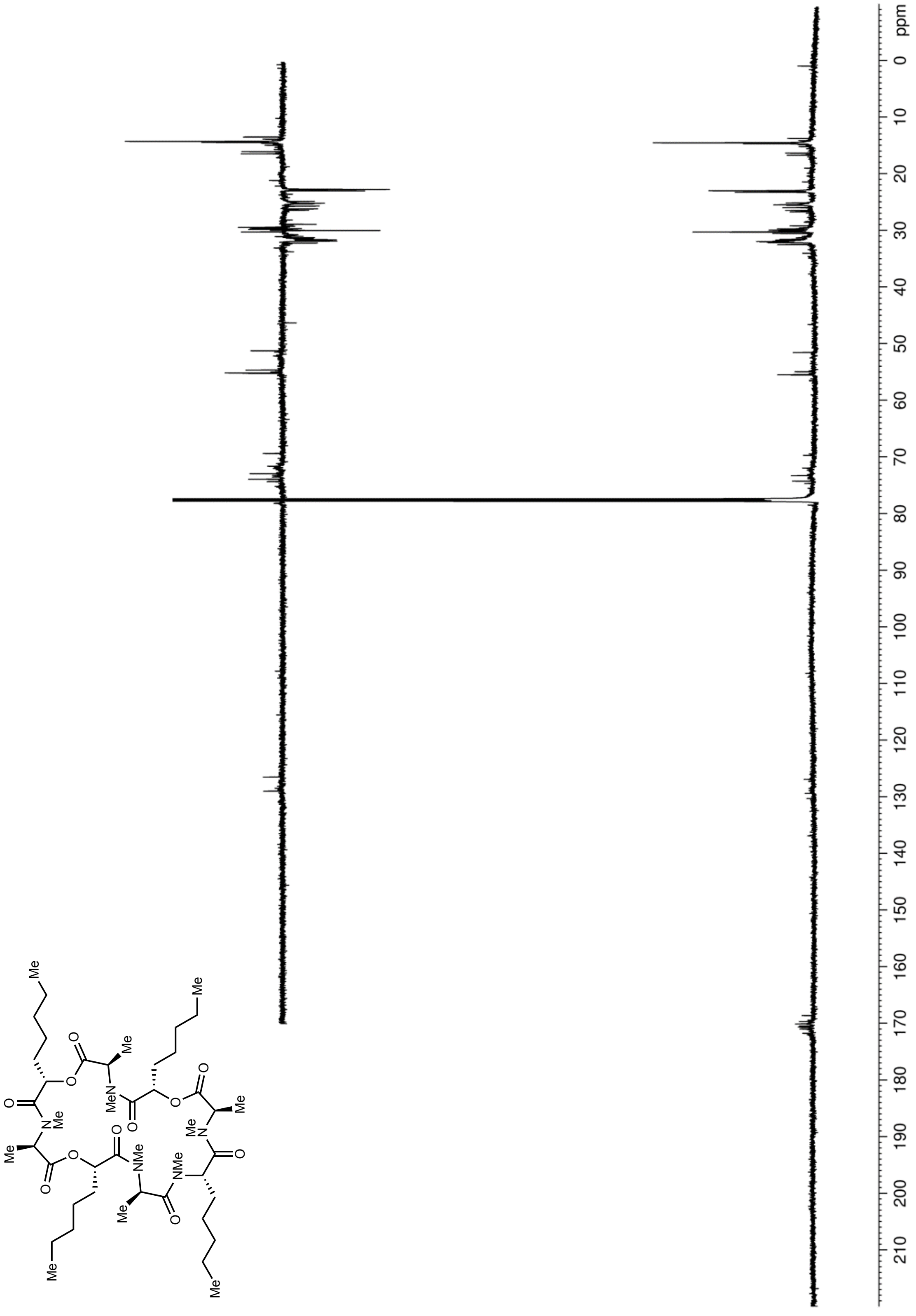

Figure S105. ^{1}H NMR (600 MHz, $CDCl_3$) of **3.2**

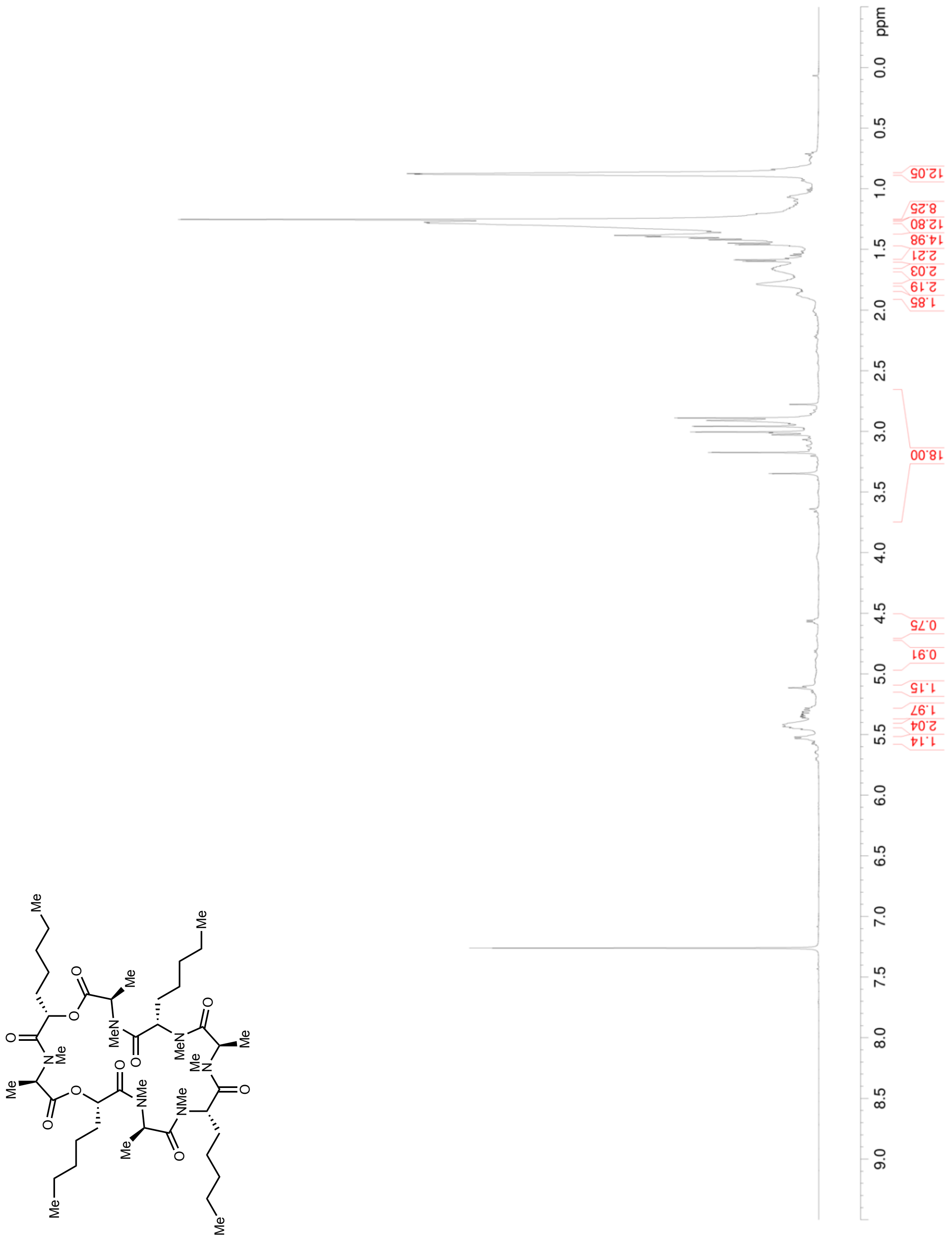

Figure S106. ^{13}C NMR (150 MHz, $CDCl_3$) of **3.2**

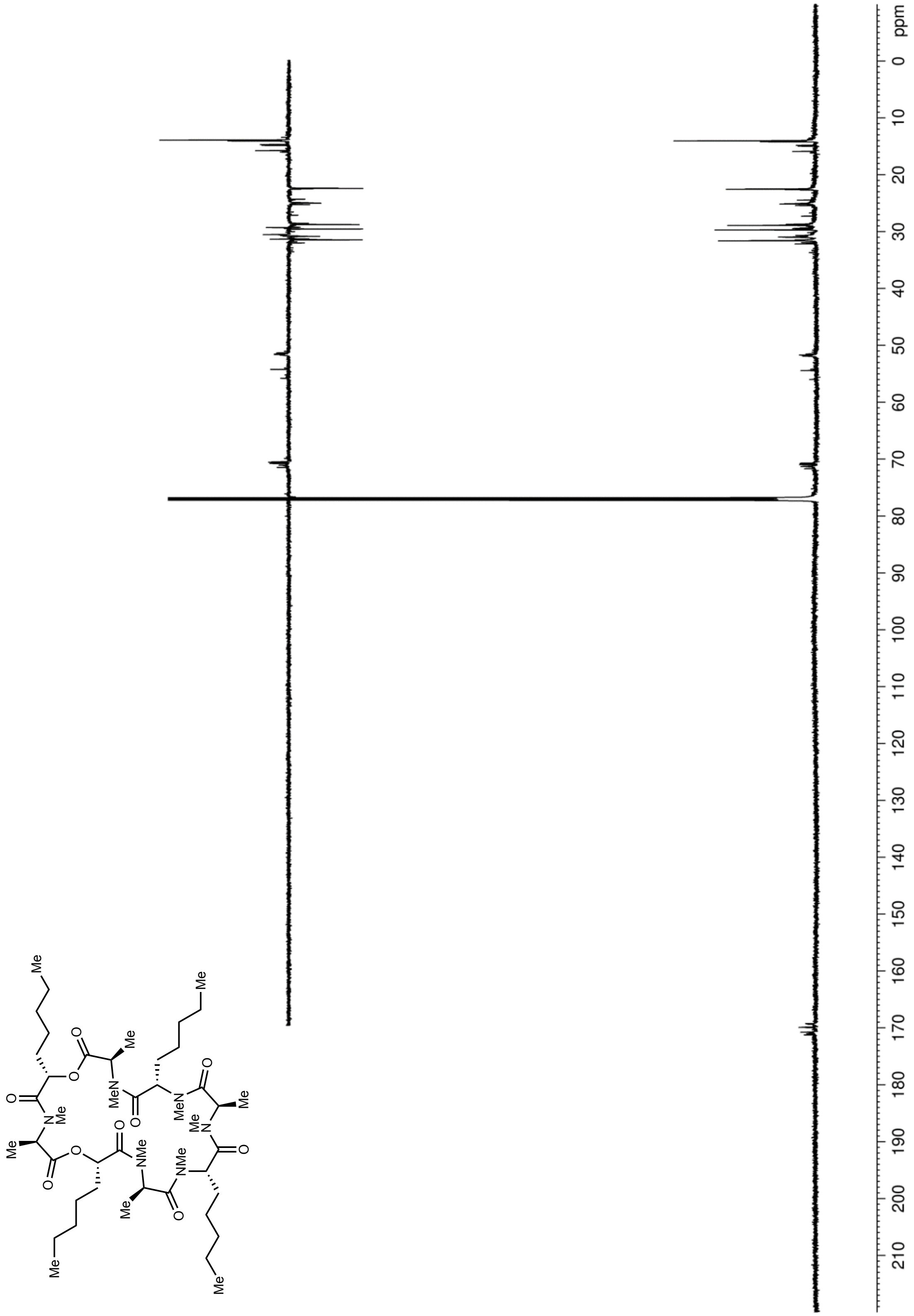

Figure S107. ^{1}H NMR (600 MHz, $CDCl_3$) of **3.3**

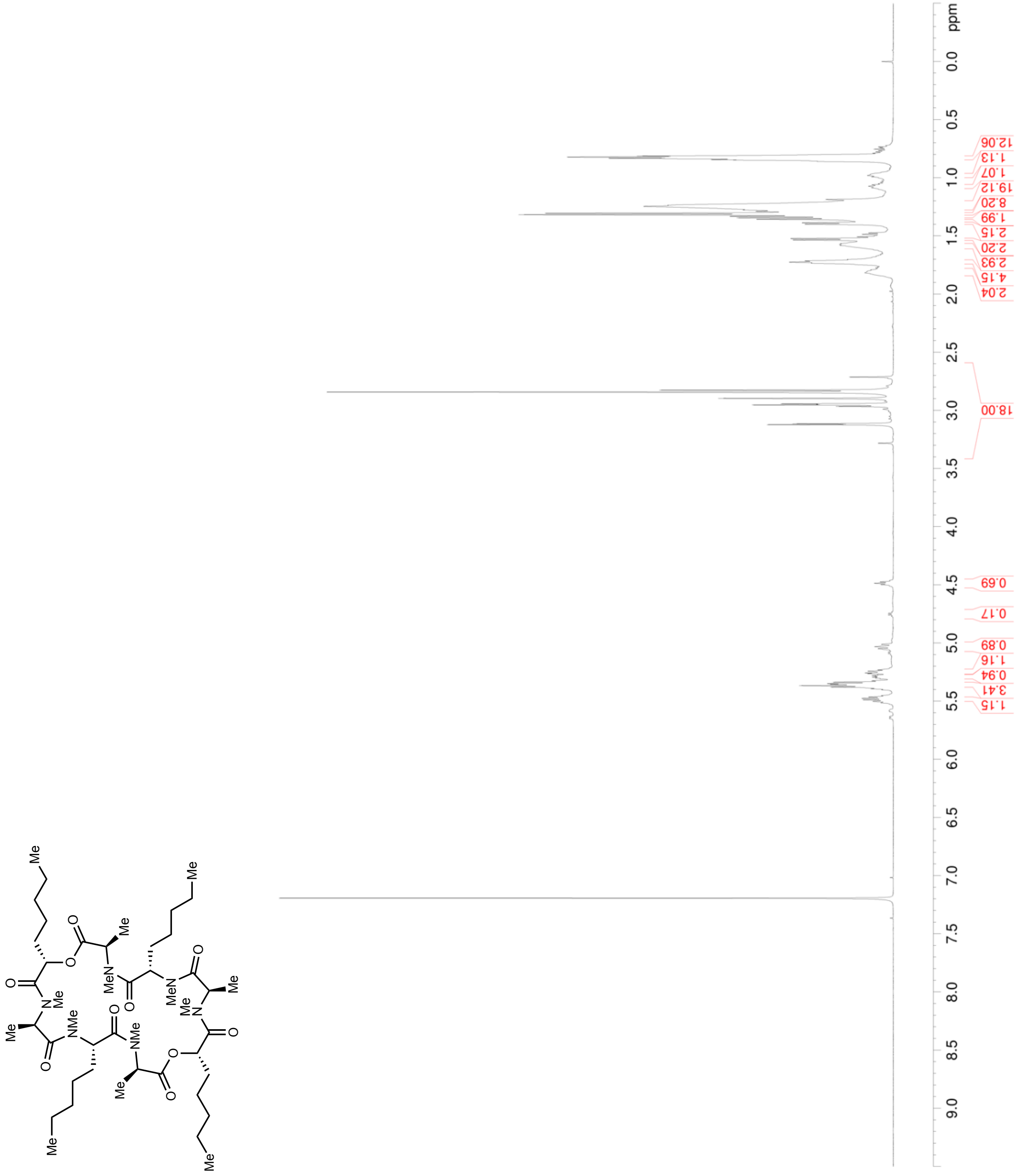

Figure S108. ^{13}C NMR (150 MHz, $CDCl_3$) of **3.3**

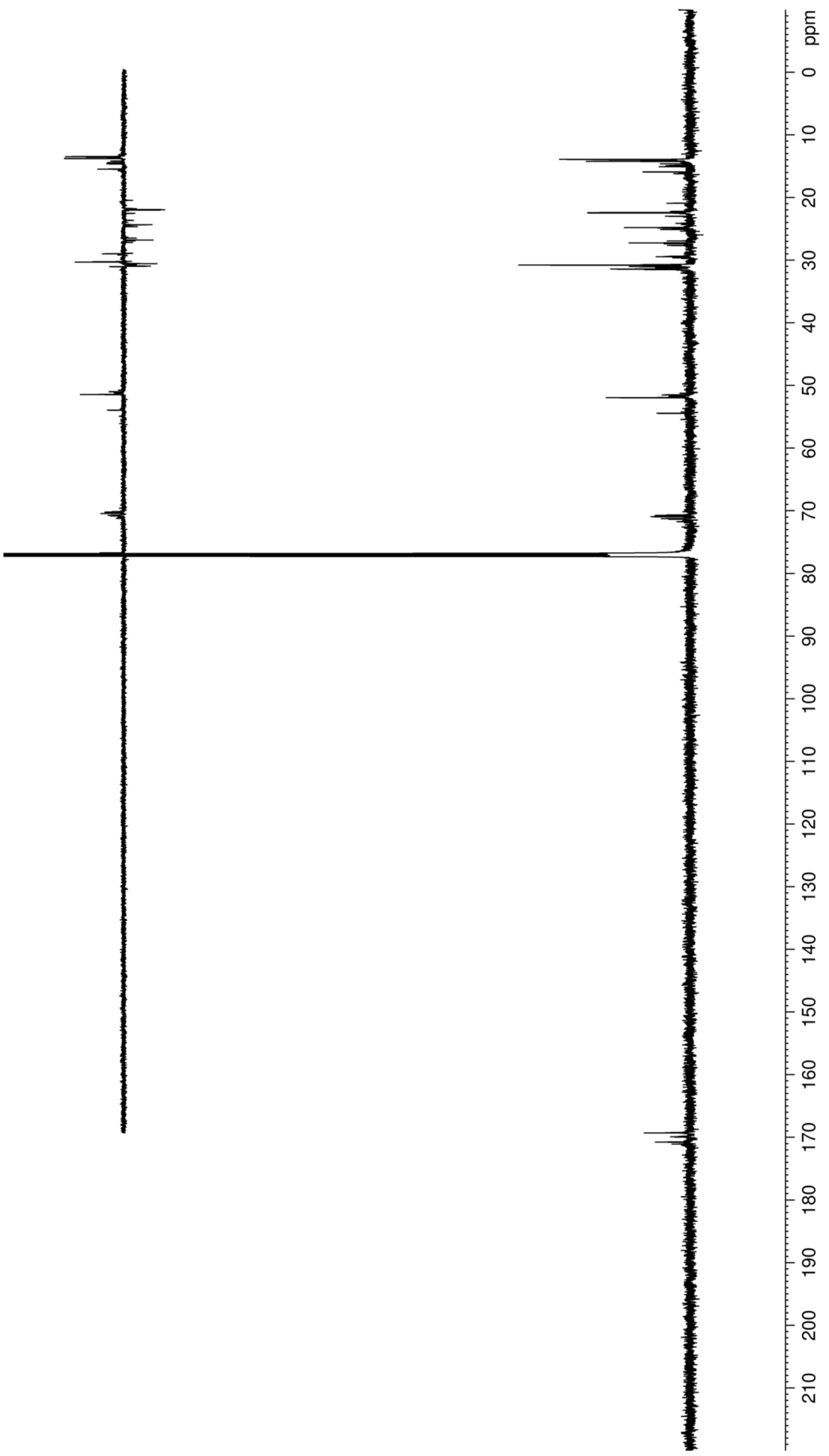

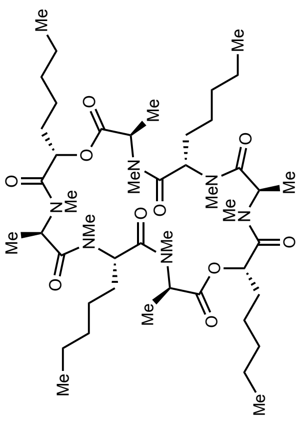

Figure S109. ^{1}H NMR (600 MHz, $CDCl_3$) of **3.4**

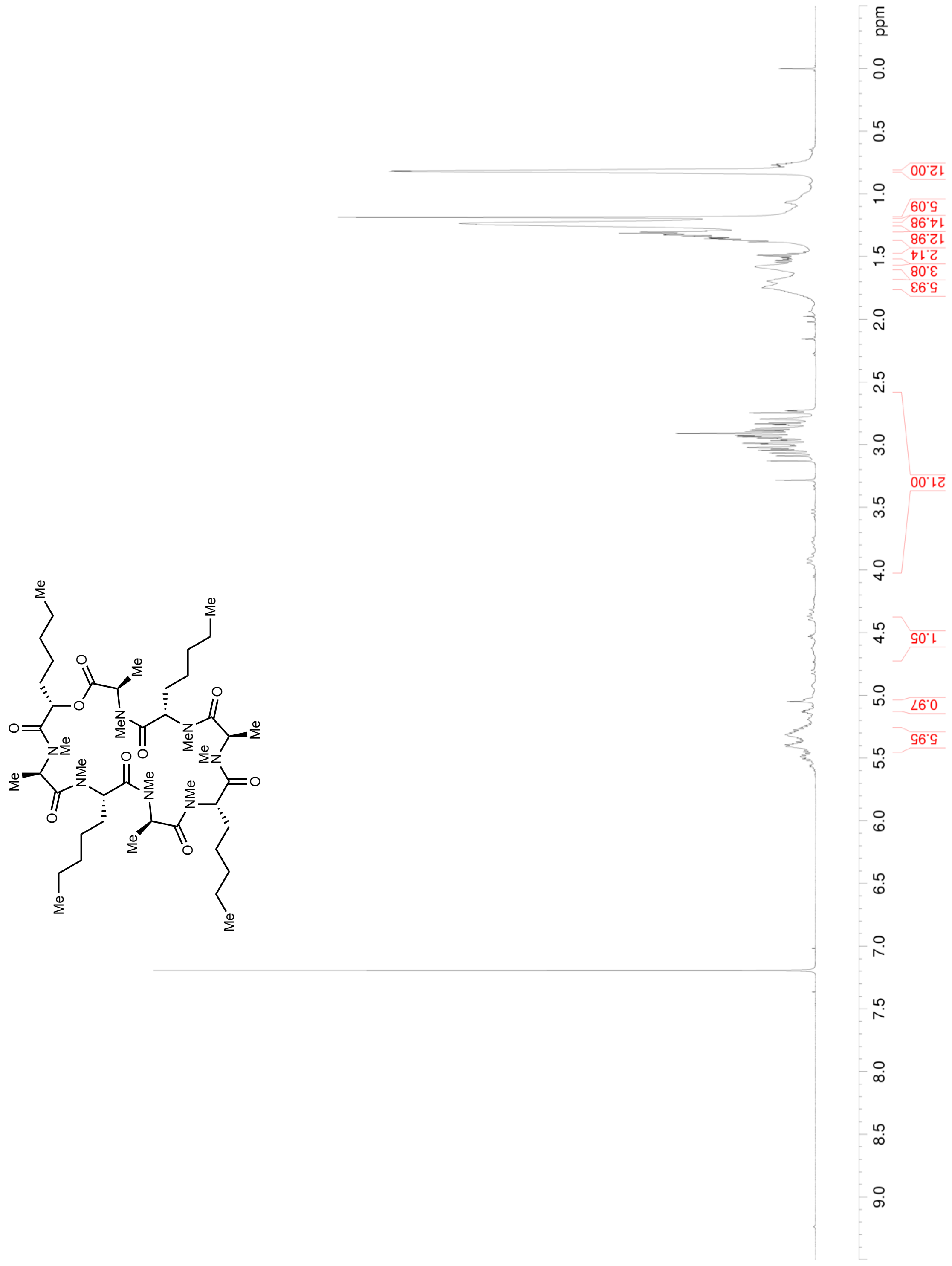

Figure S110. ^{13}C NMR (150 MHz, $CDCl_3$) of **3.4**

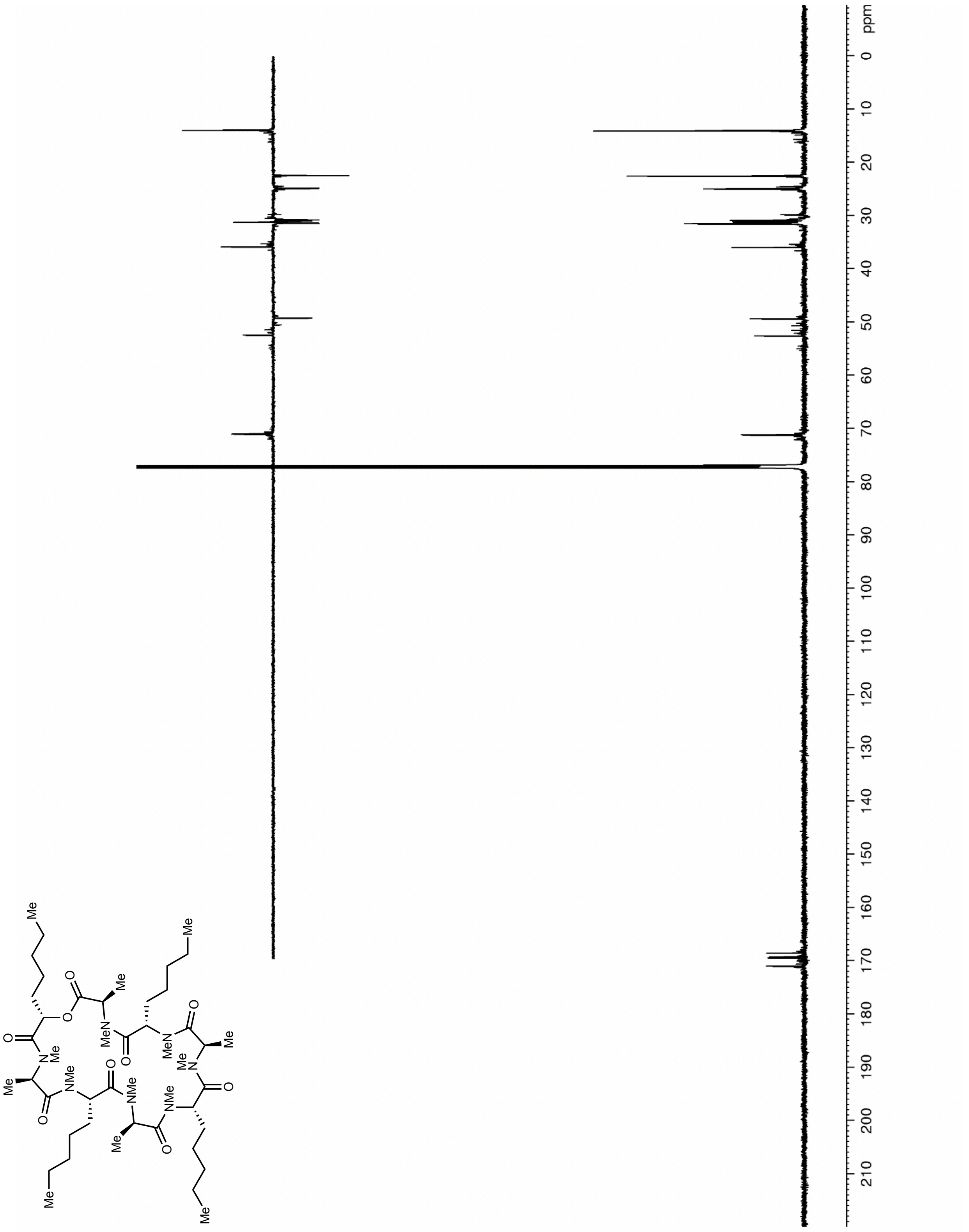

Figure S111. ^{1}H NMR (600 MHz, $CDCl_3$) of **3.5**

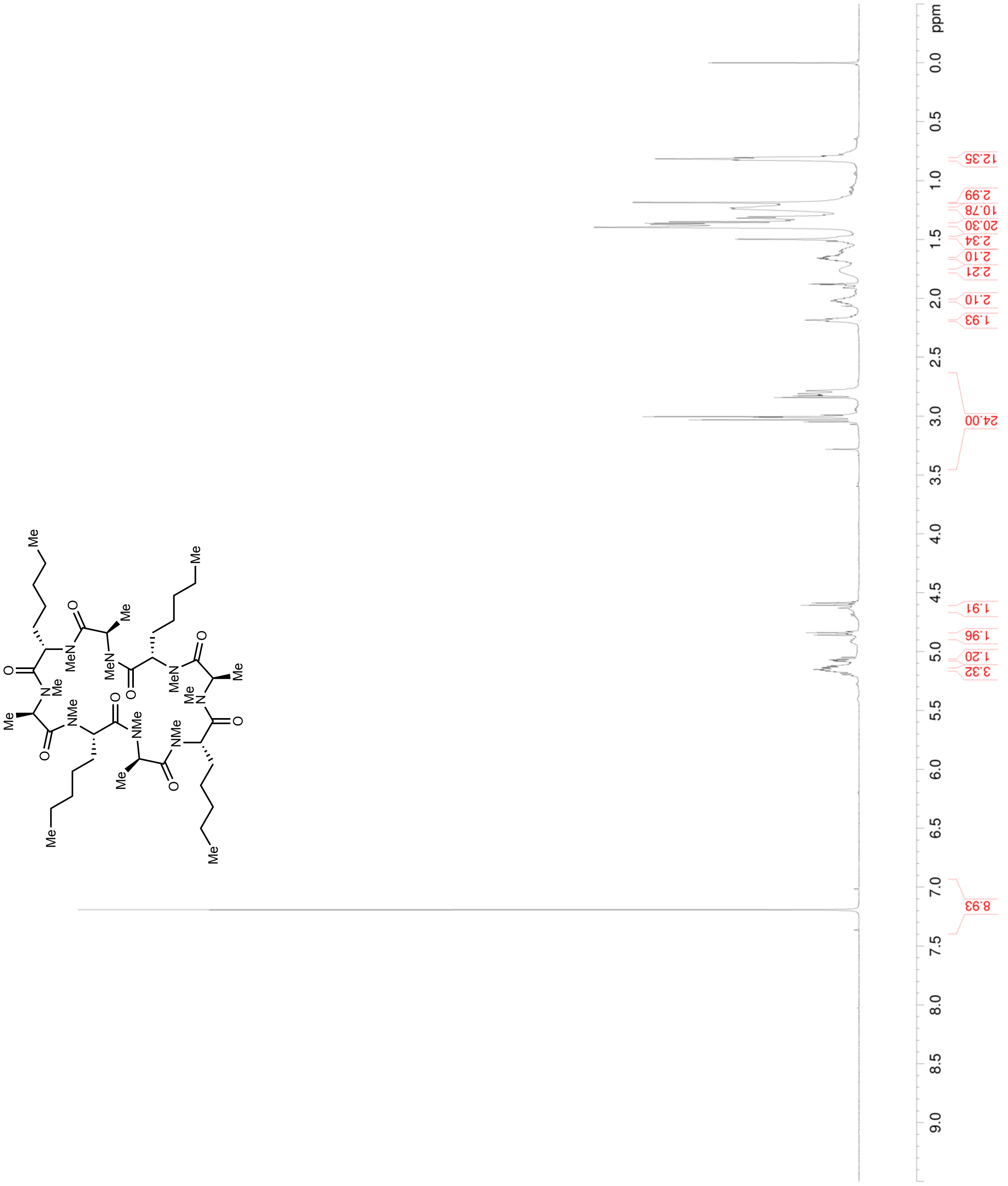

Figure S112. ^{13}C NMR (150 MHz, $CDCl_3$) of **3.5**

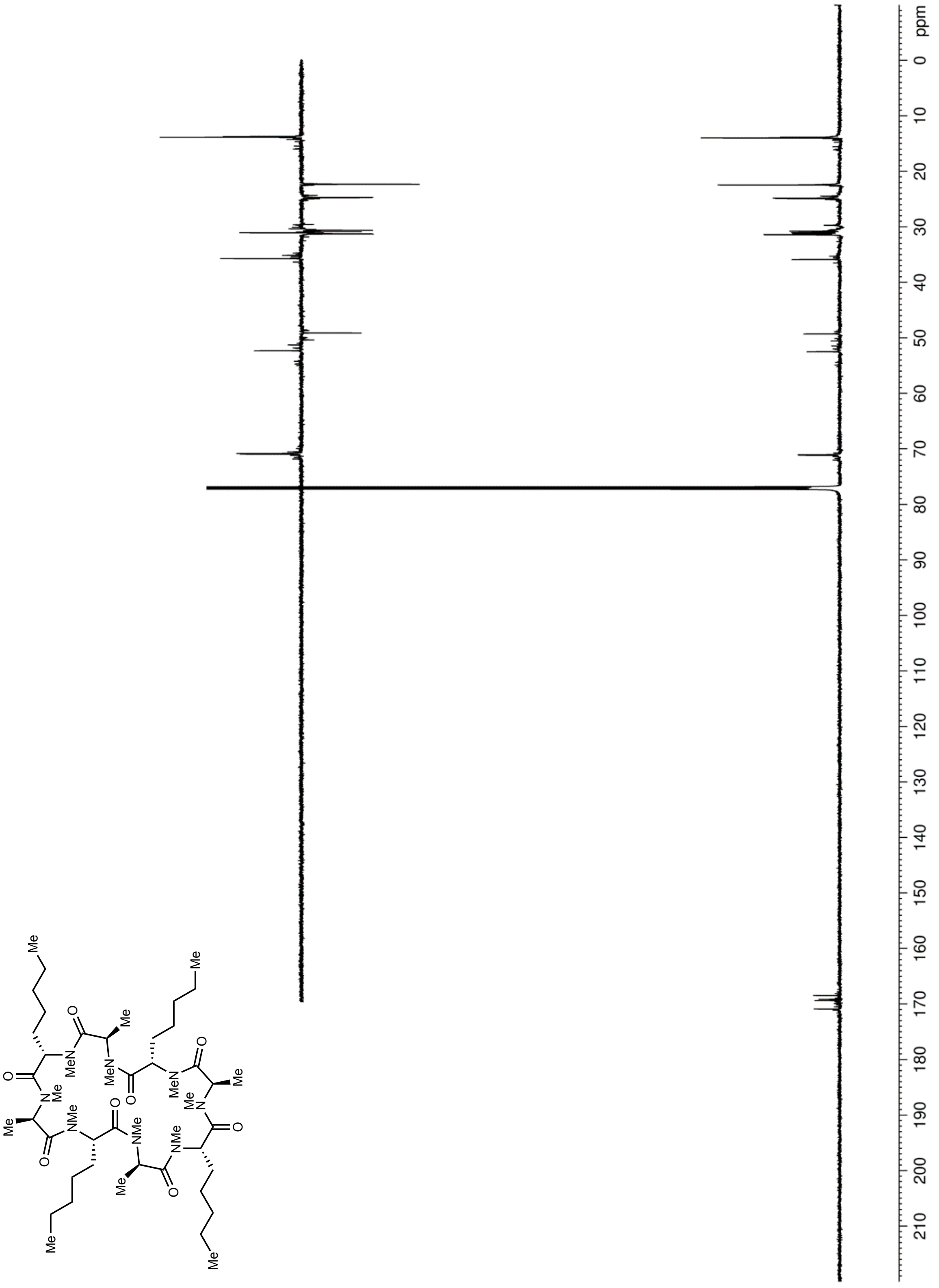

Figure S113. ^{1}H NMR (600 MHz, $CDCl_3$) of **4.2**

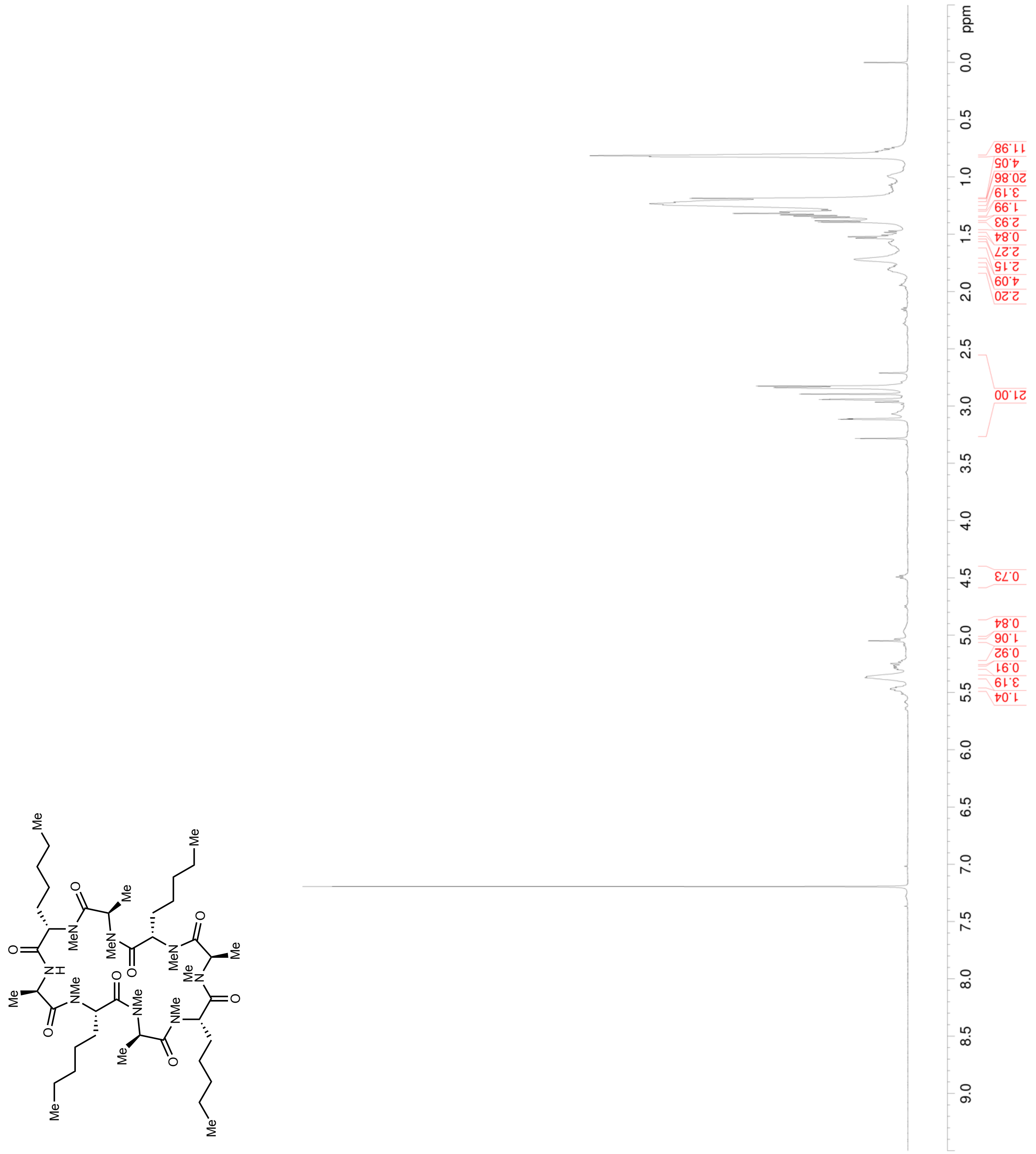

Figure S114. ^{13}C NMR (150 MHz, $CDCl_3$) of **4.2**

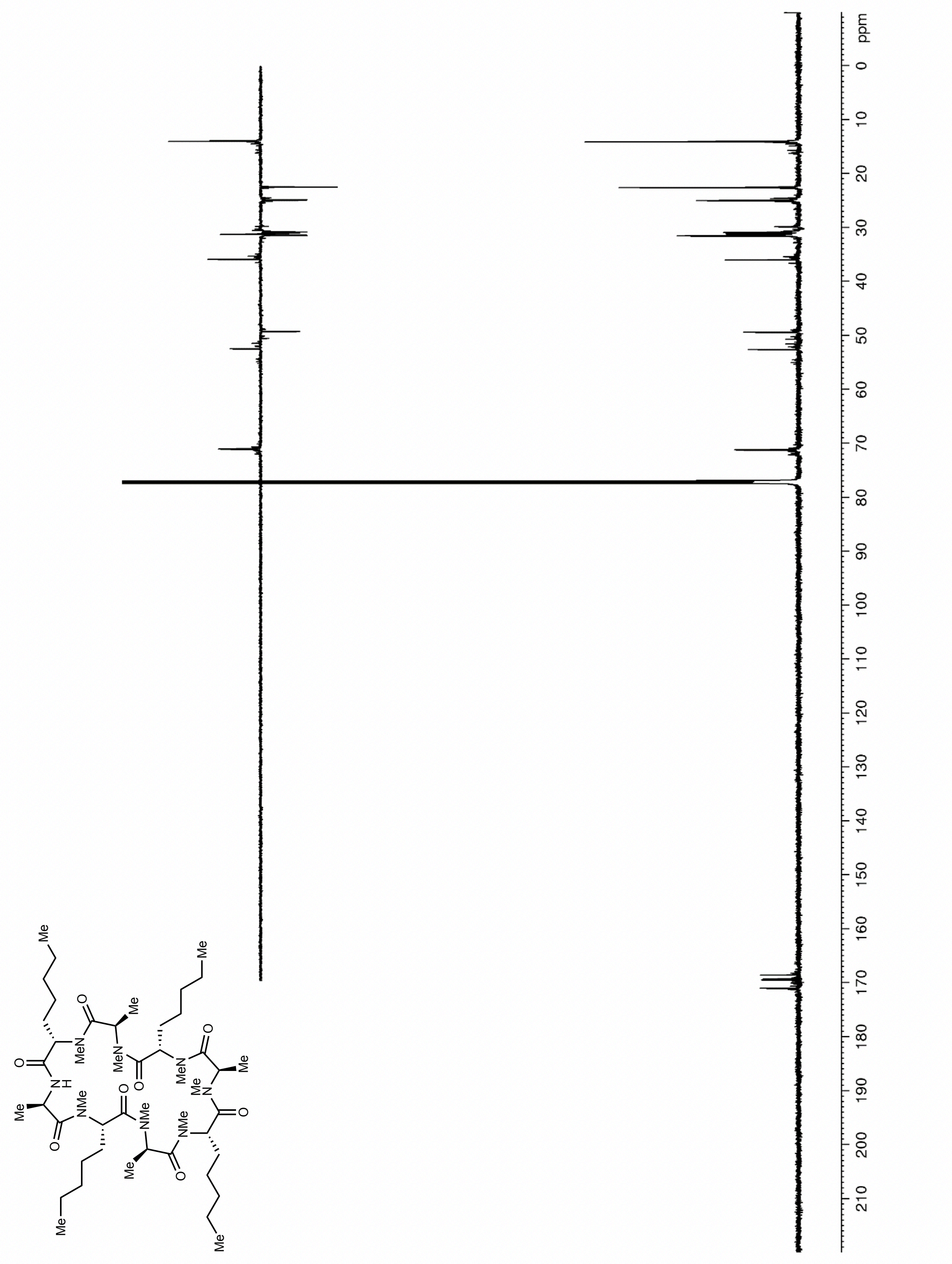

Figure S115. ^{1}H NMR (600 MHz, $CDCl_3$) of **4.3**

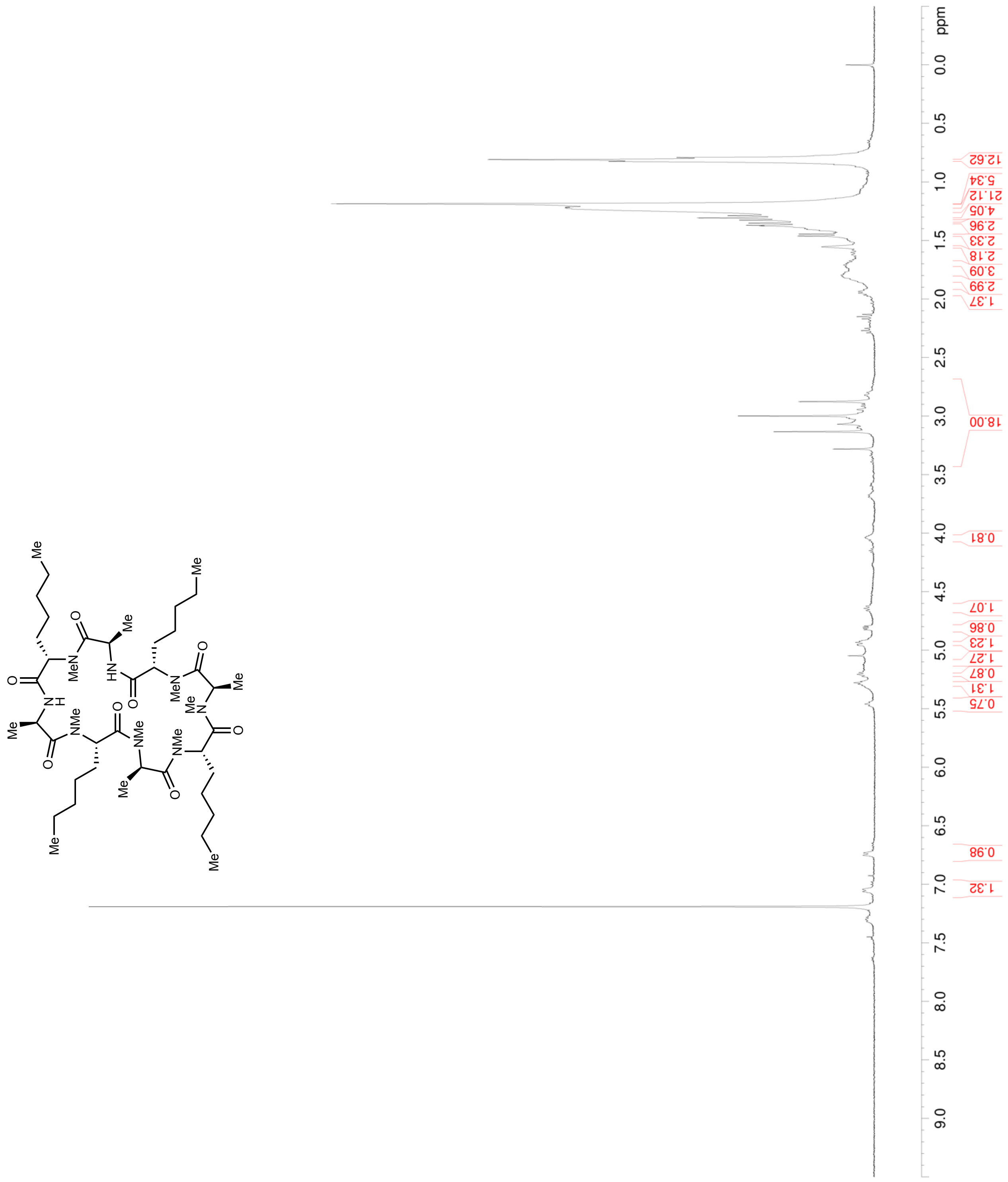

Figure S116. ^{13}C NMR (150 MHz, $CDCl_3$) of **4.3**

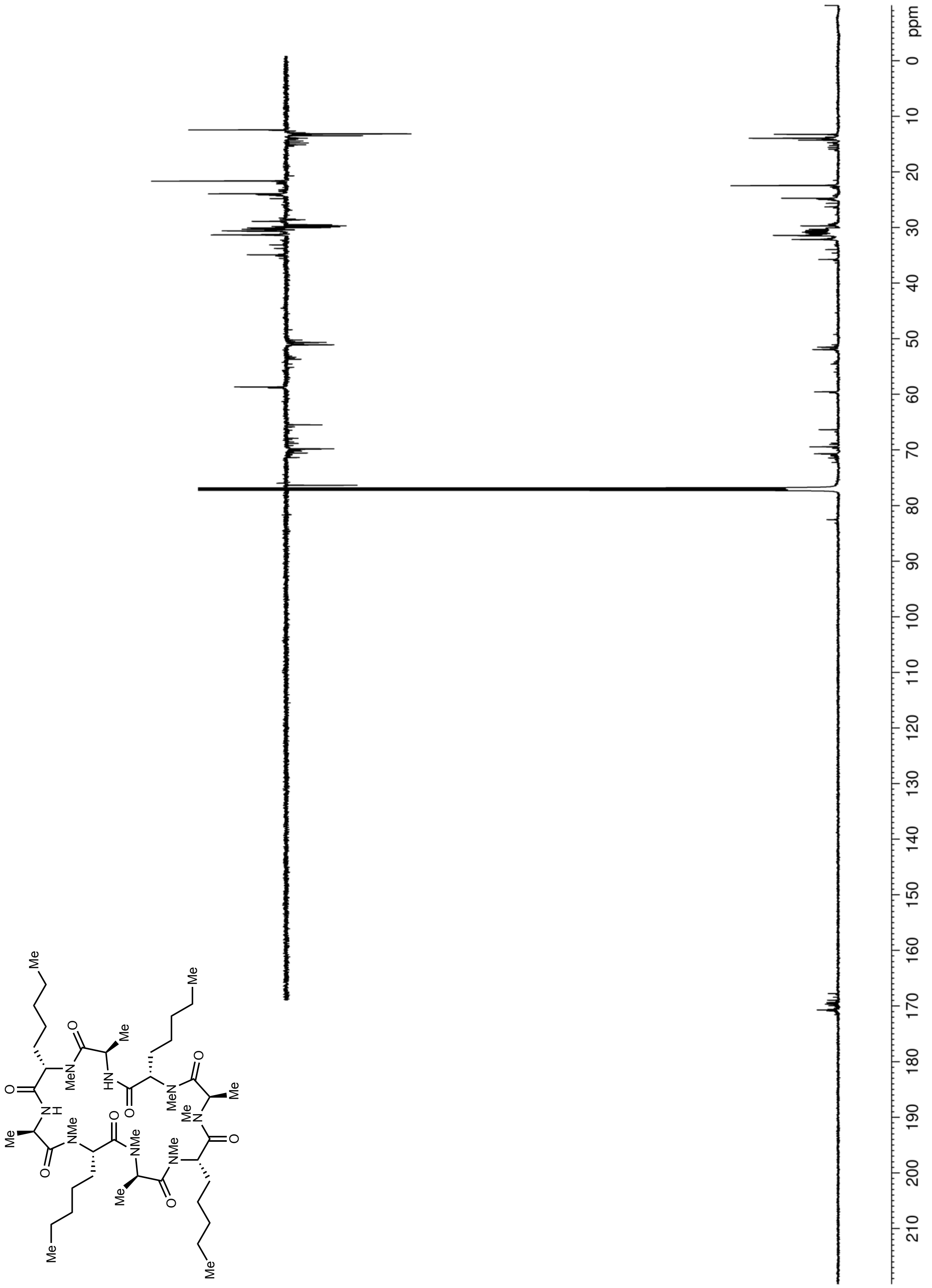

Figure S117. ^{1}H NMR (600 MHz, $CDCl_3$) of **4.4**

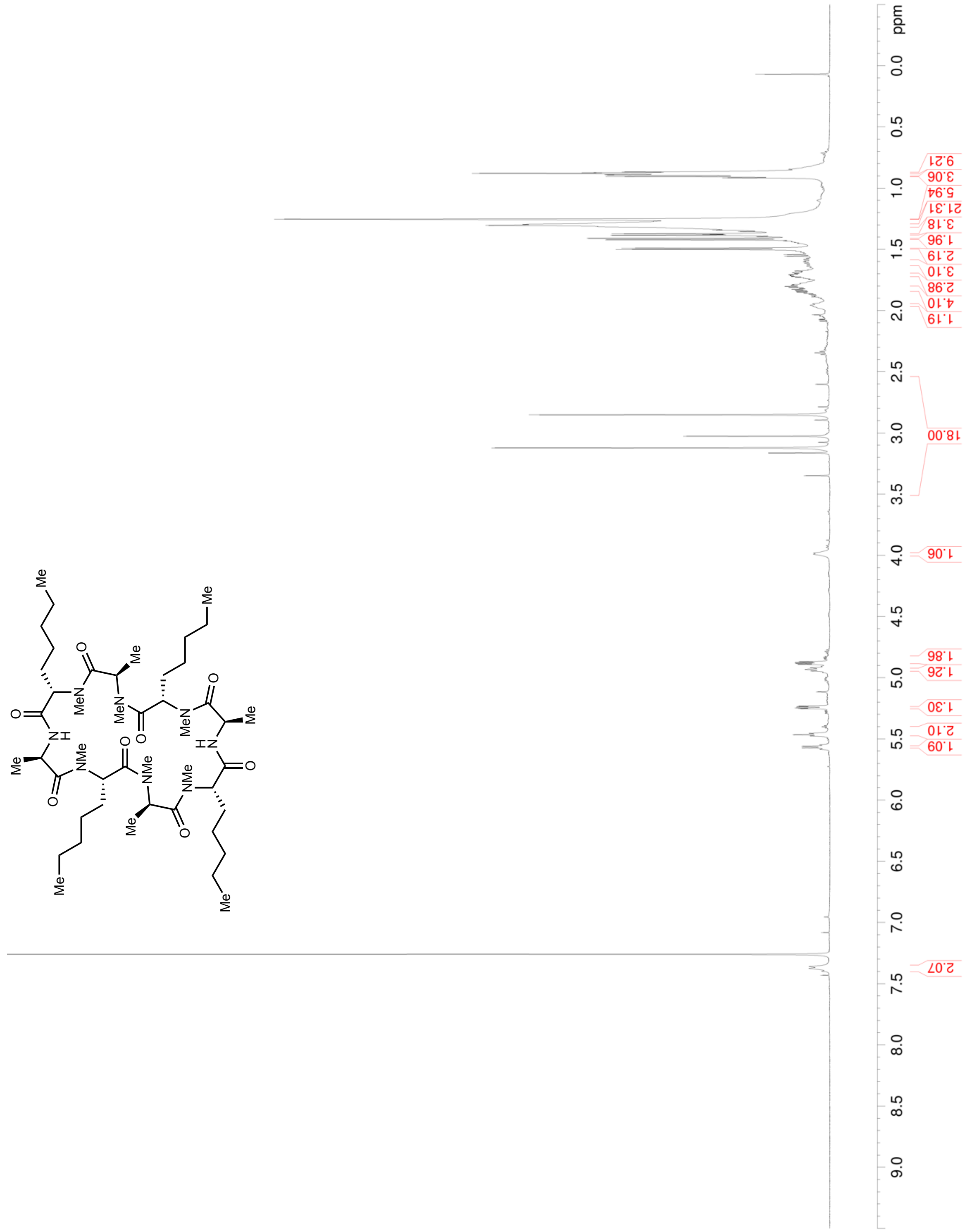

Figure S118. ^{13}C NMR (150 MHz, $CDCl_3$) of **4.4**

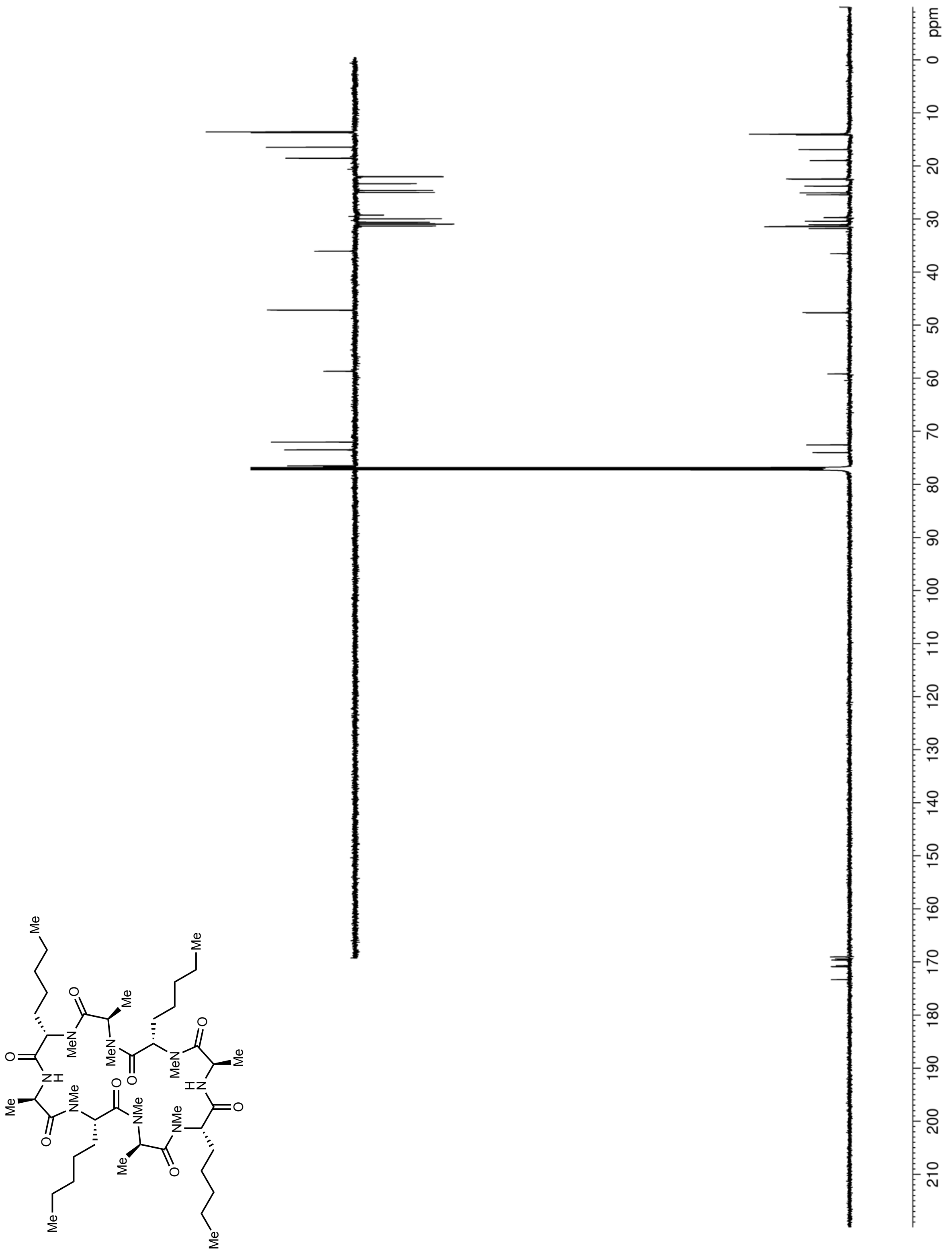

Figure S119. ^{1}H NMR (600 MHz, $CDCl_3$) of **4.5**

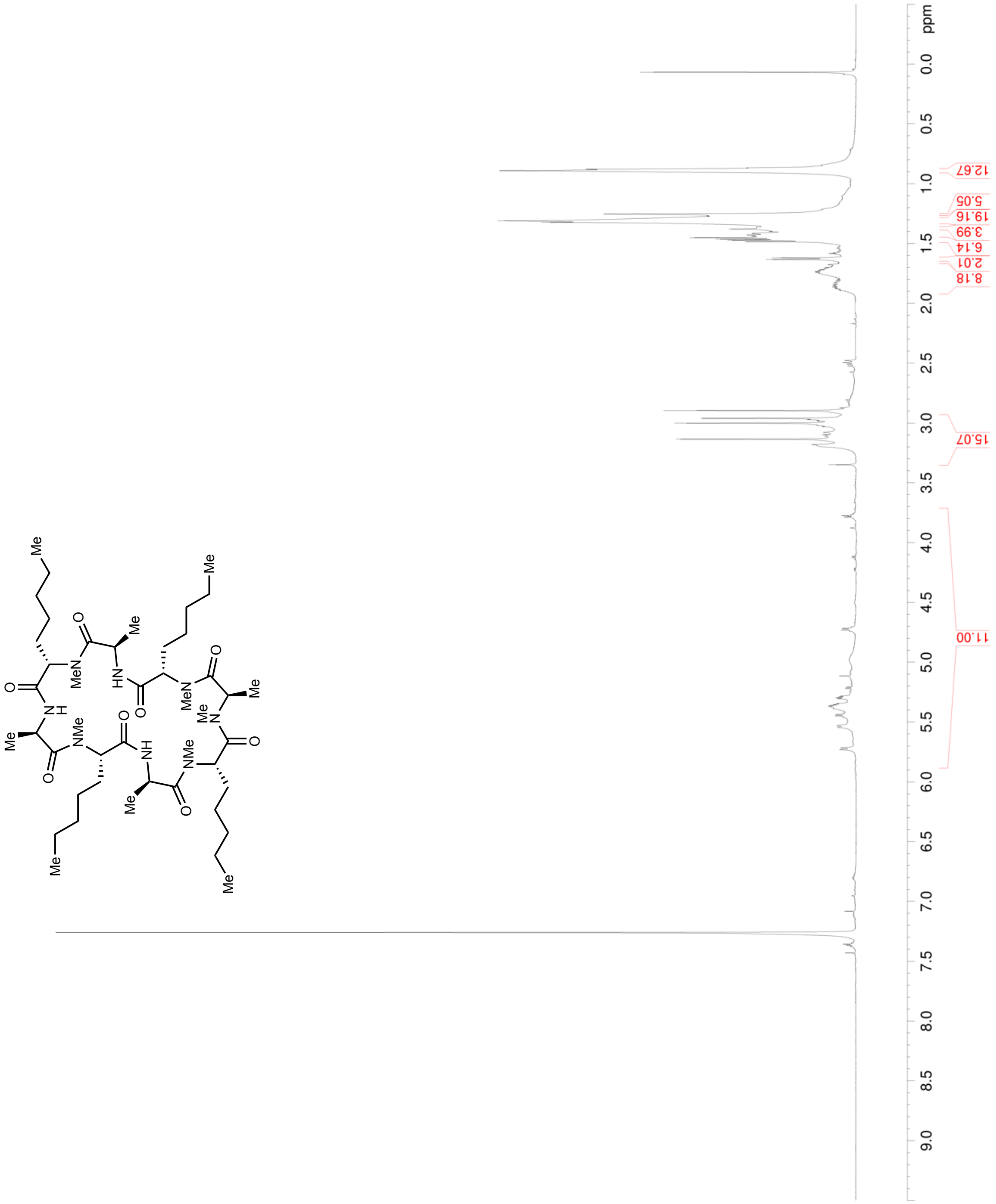

Figure S120. ^{13}C NMR (150 MHz, $CDCl_3$) of **4.5**

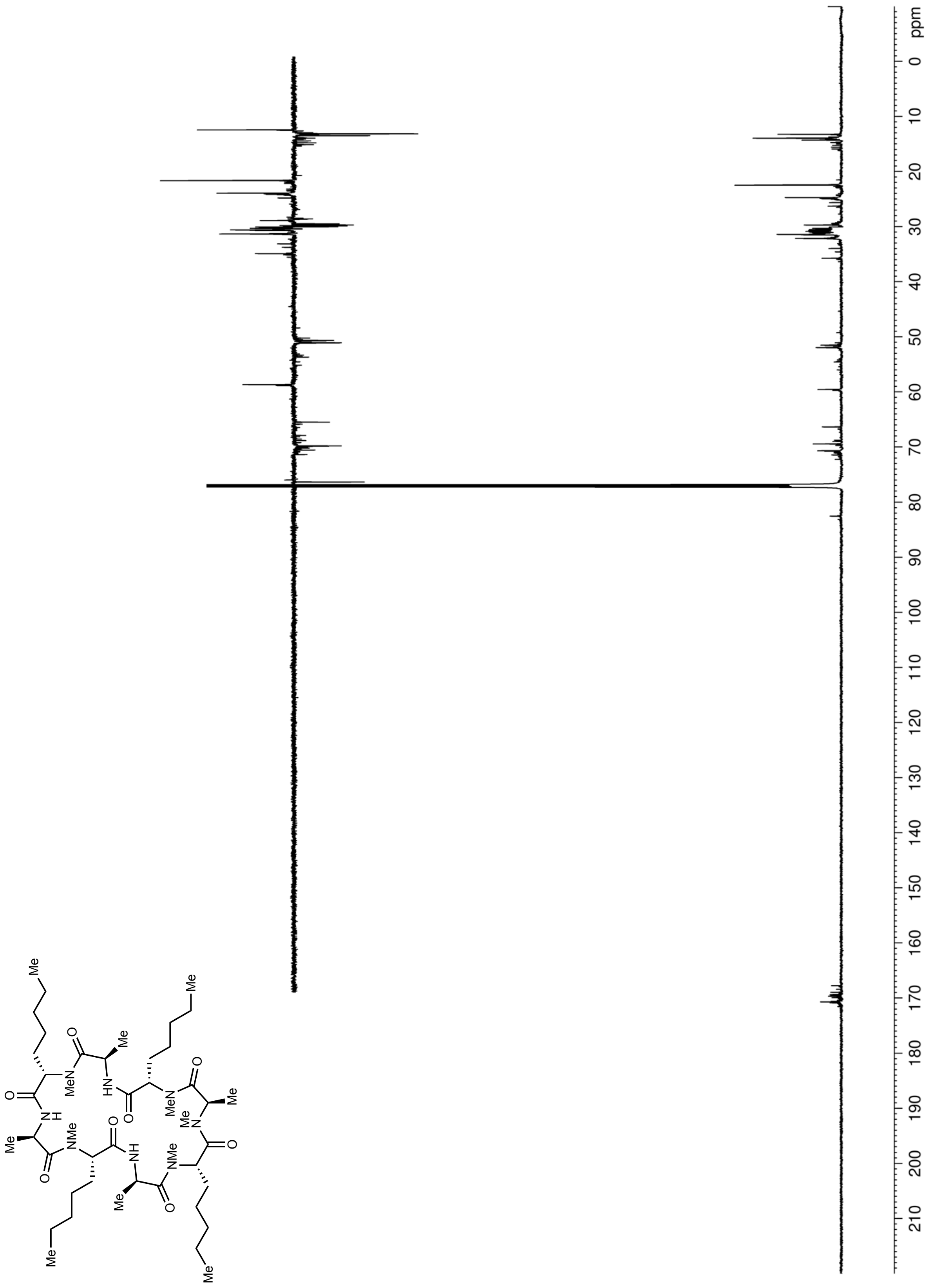

Figure S121. ^{1}H NMR (600 MHz, $CDCl_3$) of **4.6**

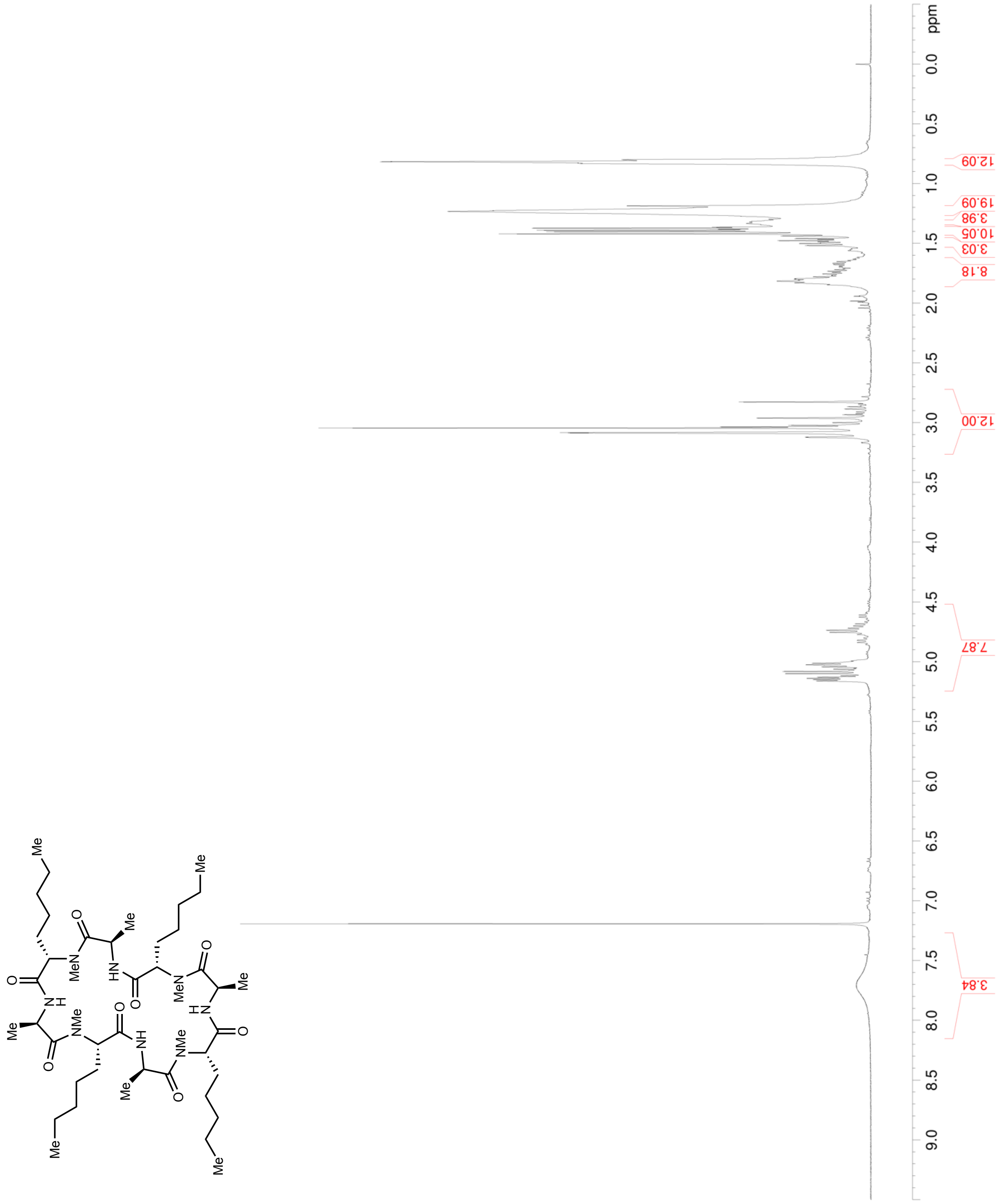

Figure S122. ^{13}C NMR (150 MHz, $CDCl_3$) of **4.6**

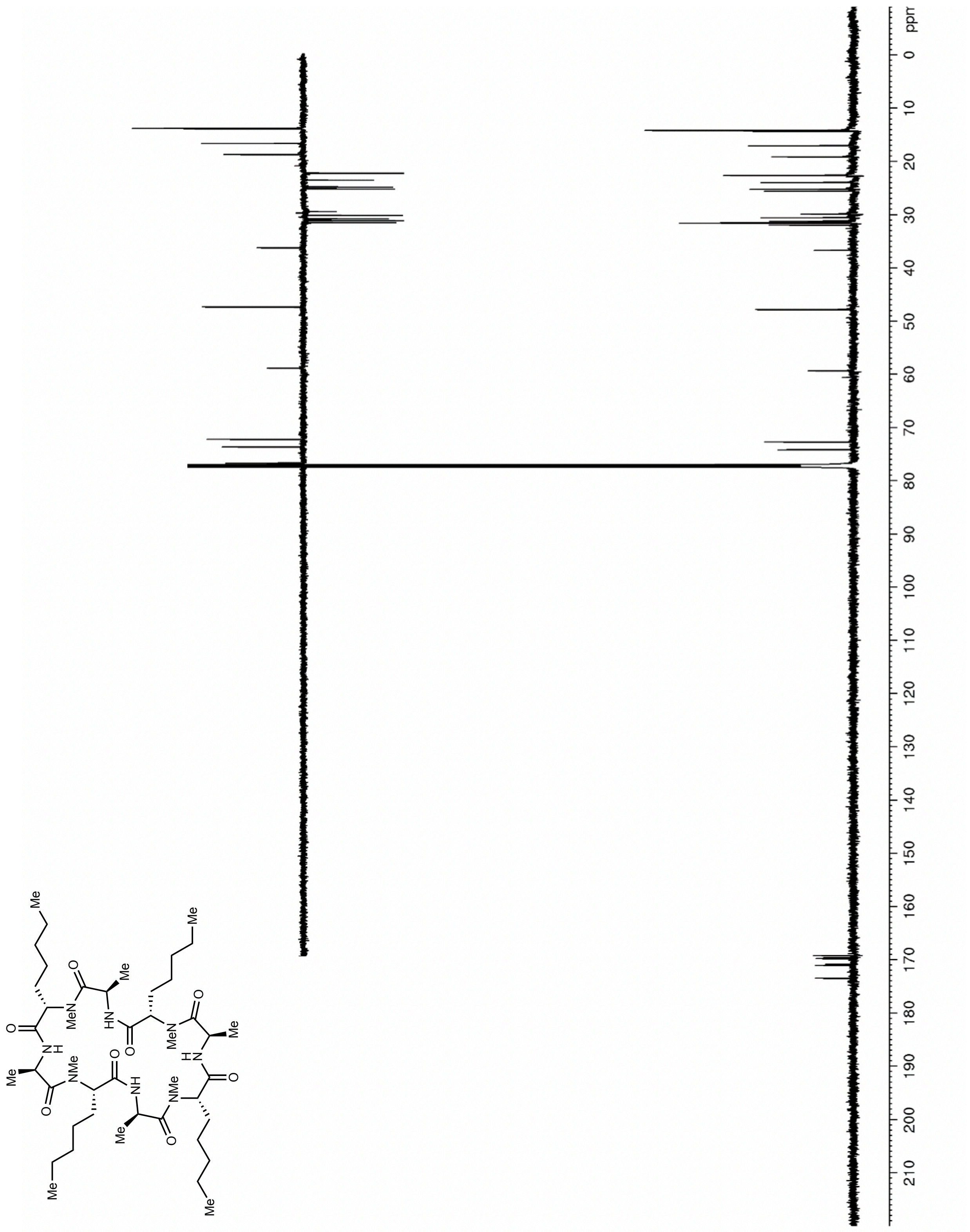

Figure S123. ^{1}H NMR (600 MHz, $CDCl_3$) of **5.2**

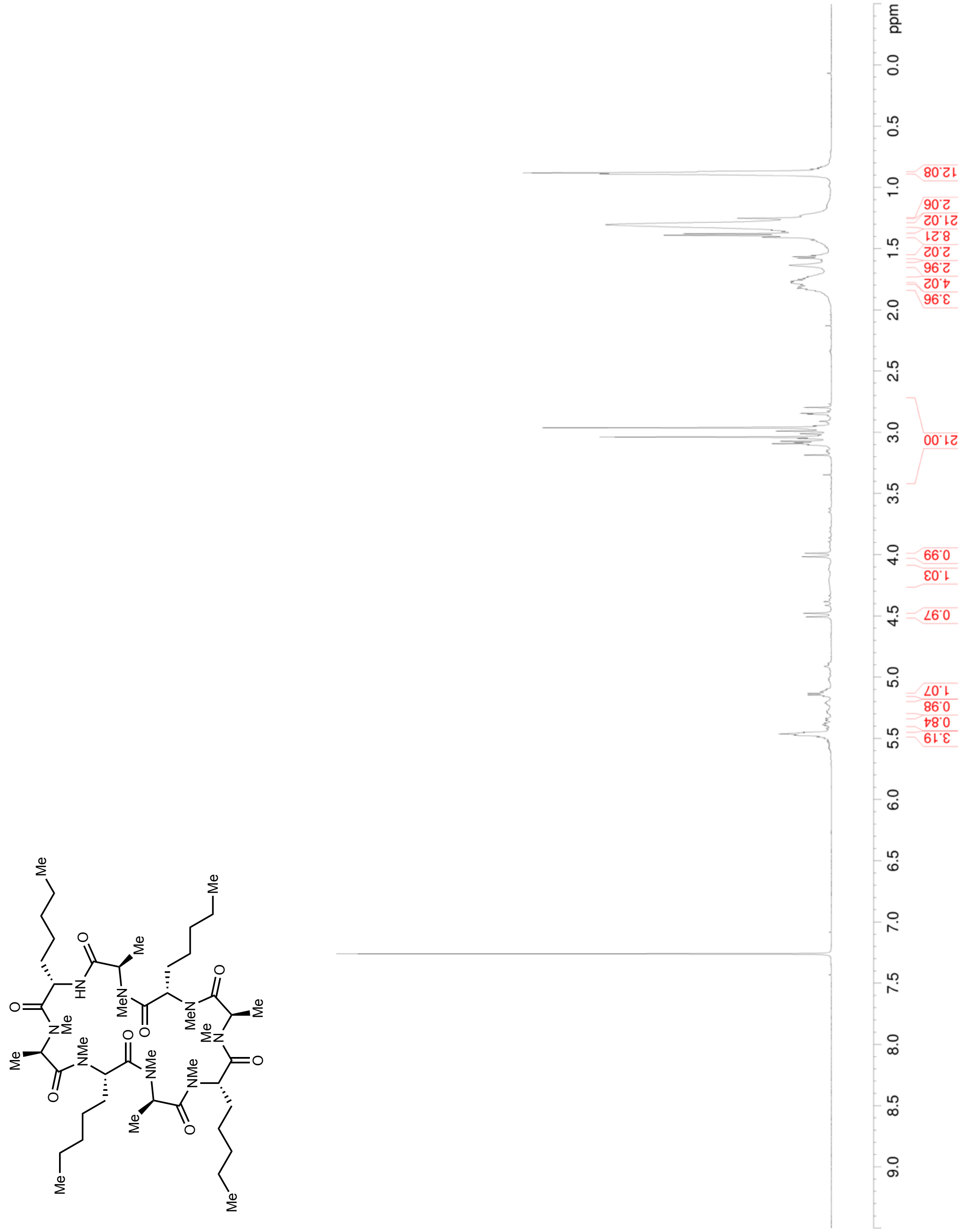

Figure S124. ^{13}C NMR (150 MHz, $CDCl_3$) of **5.2**

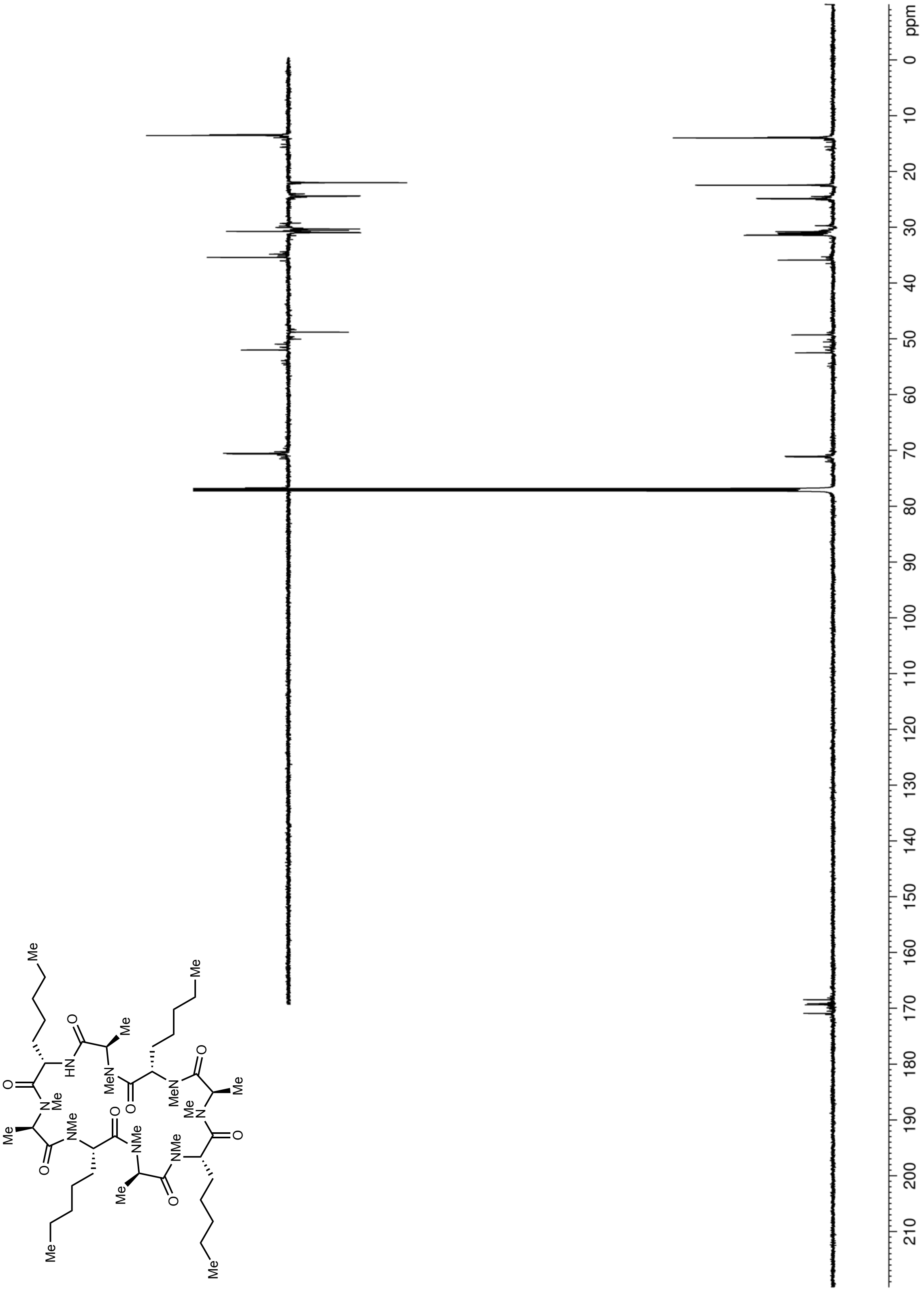

Figure S125. ^{1}H NMR (600 MHz, $CDCl_3$) of **5.3**

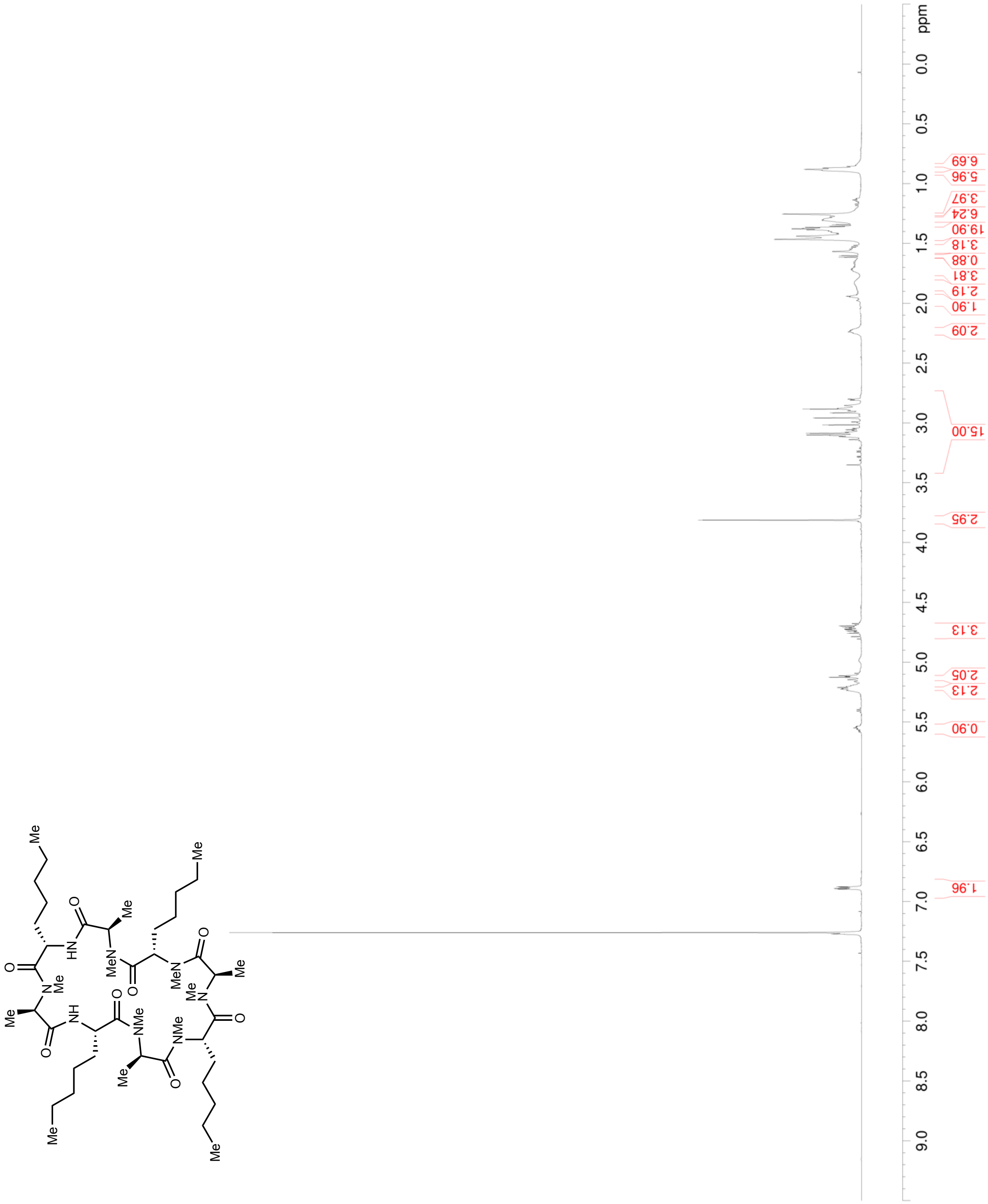

Figure S126. ^{13}C NMR (150 MHz, $CDCl_3$) of **5.3**

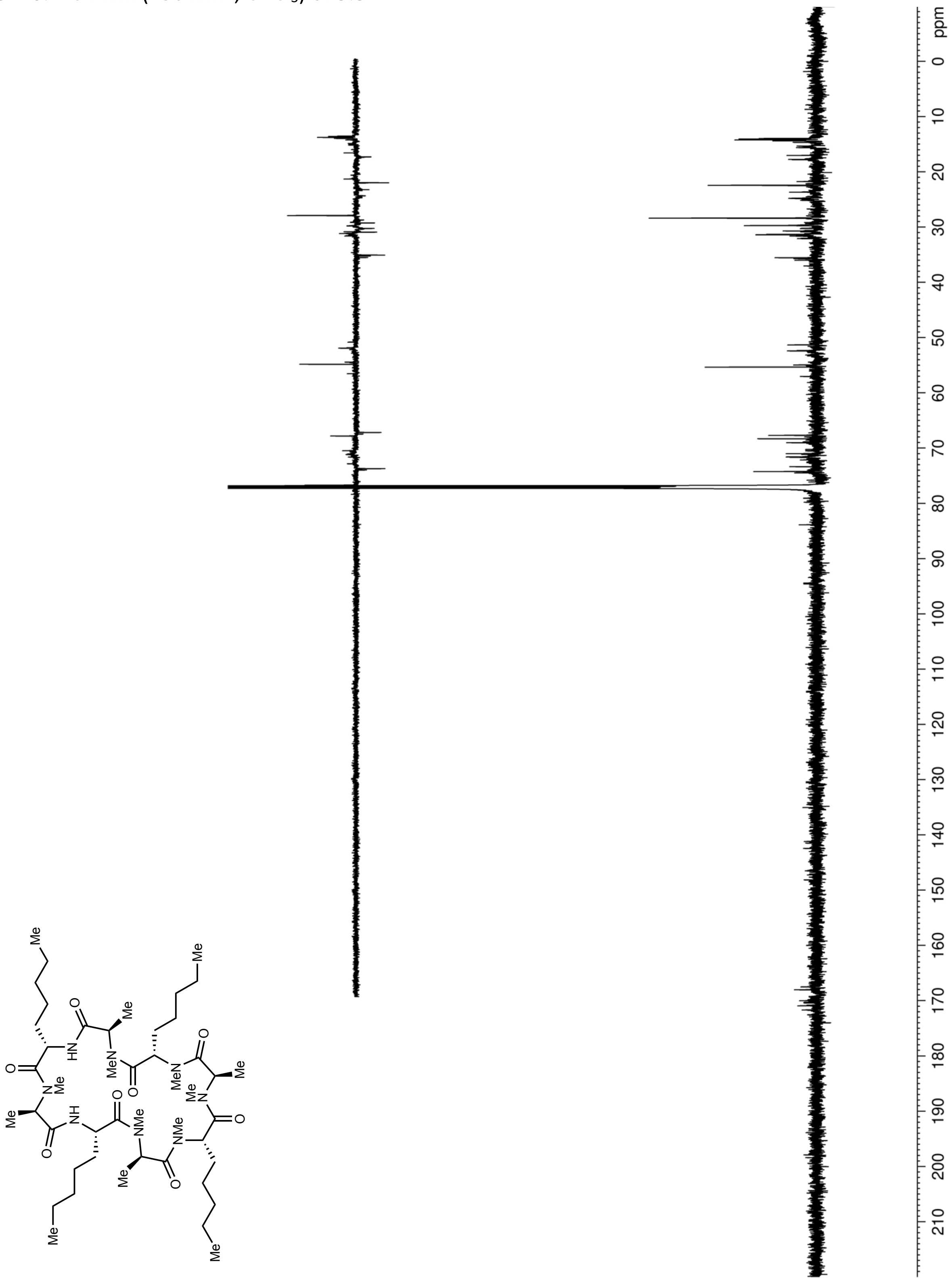

Figure S127. ^{1}H NMR (600 MHz, $CDCl_3$) of **5.4**

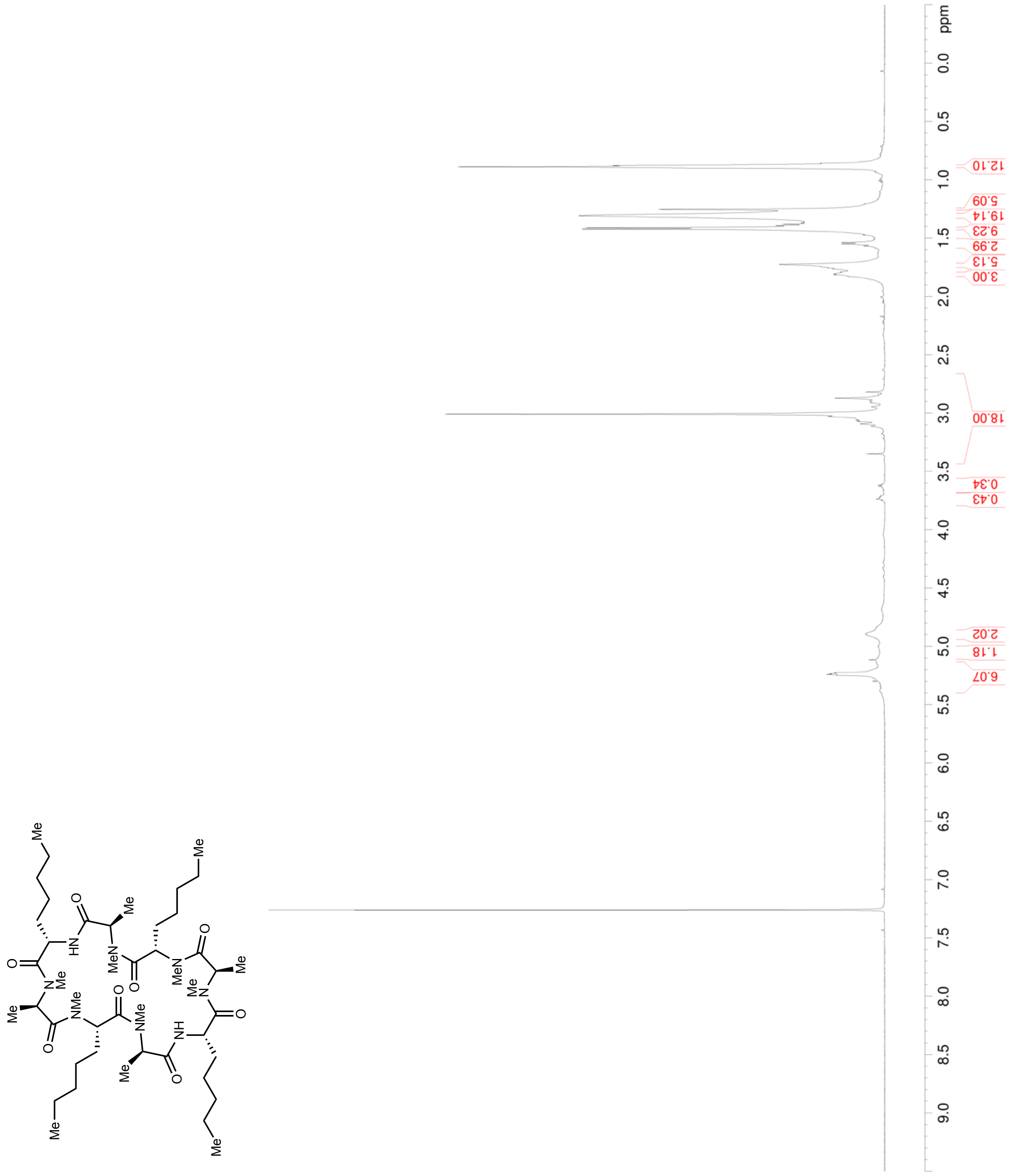

Figure S128. ^{13}C NMR (150 MHz, $CDCl_3$) of **5.4**

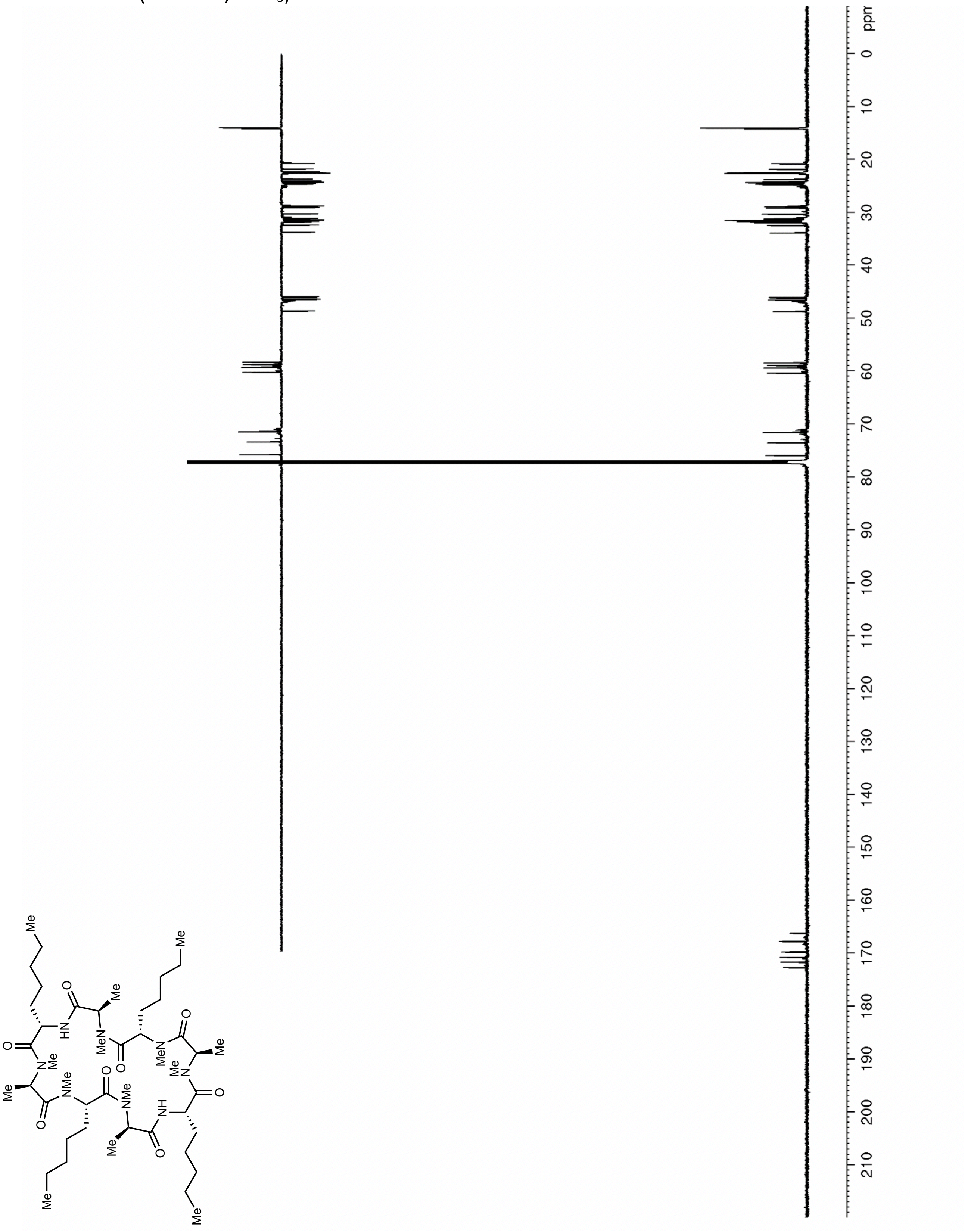

Figure S129. ^{1}H NMR (600 MHz, $CDCl_3$) of **5.5**

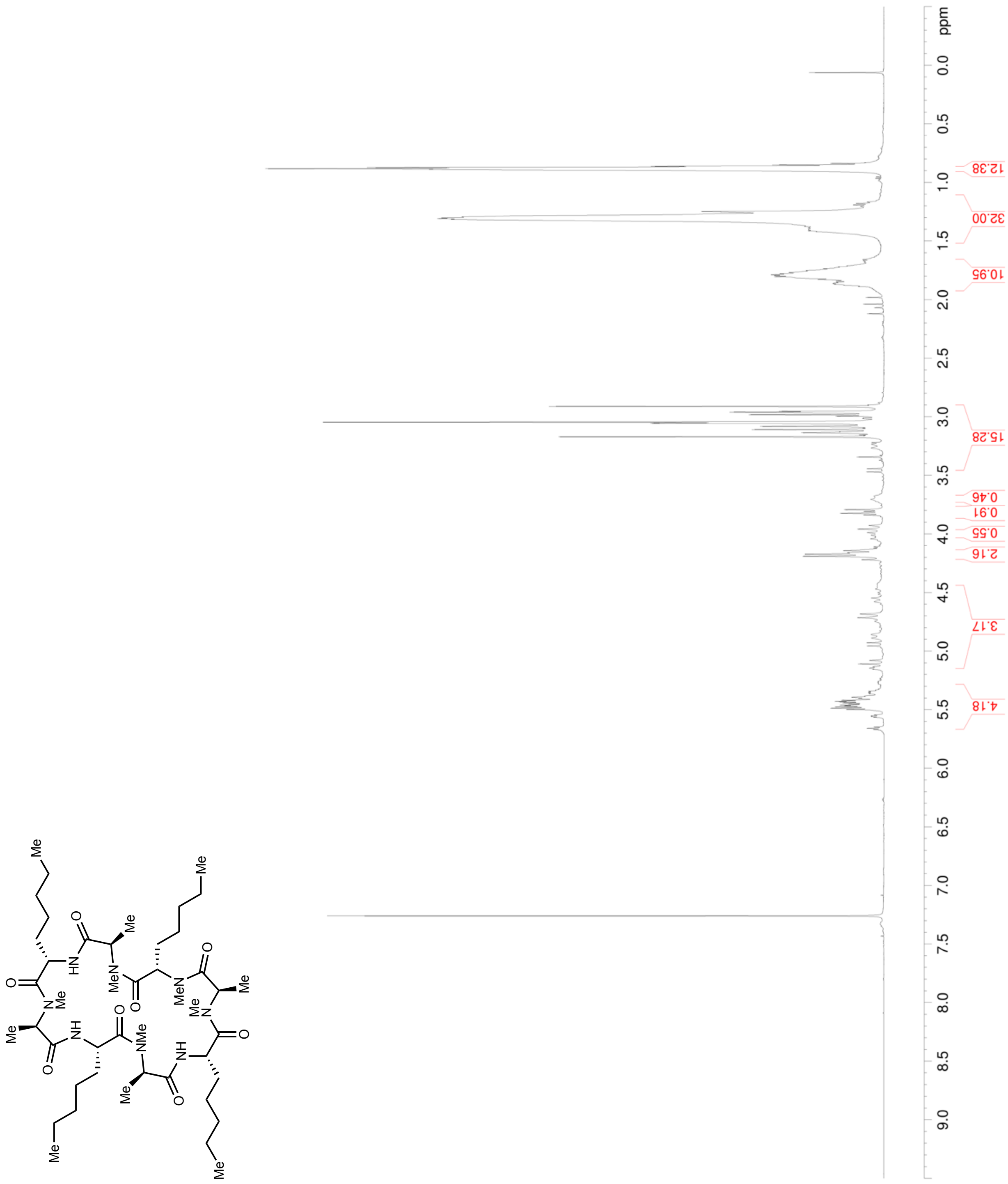

Figure S130. ^{13}C NMR (150 MHz, $CDCl_3$) of **5.5**

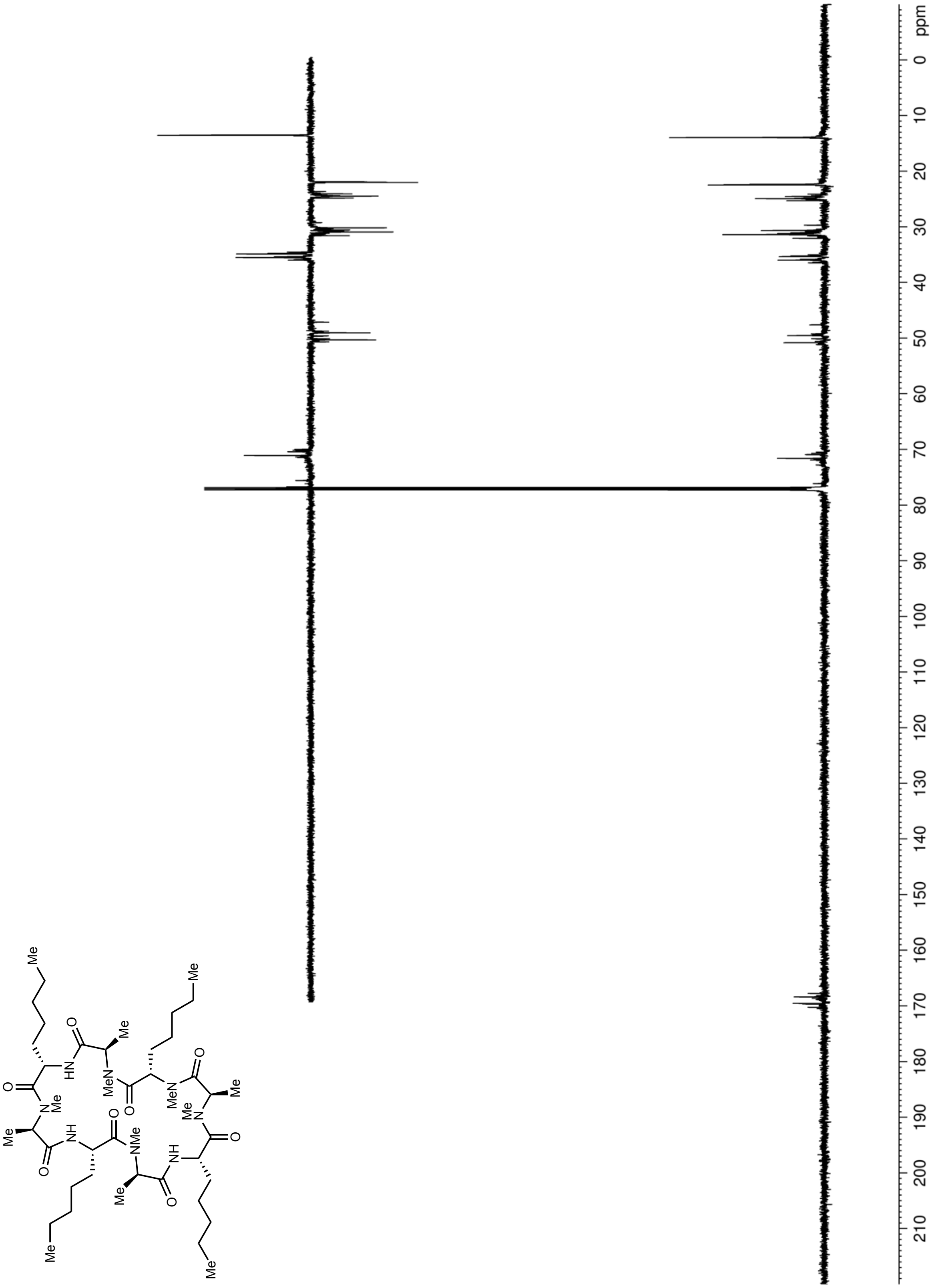

Supplement: Supplementary file 1 — jm4c00923_si_001.pdf [file jm4c00923_si_001.pdf]
