# Supplementary material for: Backbone-Determined Antiarrhythmic Structure–Activity Relationships for a Mirror Image, Oligomeric Depsipeptide Natural Product
Source: J Med Chem. 2024 Jul 3;67(14):12205–20. doi: 10.1021/acs.jmedchem.4c00923 (PMC11284792; doi:10.1021/acs.jmedchem.4c00923)
Supplement: Supplementary file 3 — jm4c00923_si_003.pdf [file jm4c00923_si_003.pdf]

Supporting Information 2

Backbone-Determined Antiarrhythmic Structure-Activity Relationships for a Mirror-Image, Oligomeric Depsipeptide Natural Product

Madelaine P. Thorpe,[1] Daniel J. Blackwell,[2] Bjorn C. Knollmann,[2] and Jeffrey N. Johnston[1]*

[1]Department of Chemistry and Vanderbilt Institute of Chemical Biology, Vanderbilt University, Nashville, Tennessee 37235

[2]Department of Medicine, Vanderbilt University Medical Center, Nashville, TN 37235

*E-mail: jeffrey.n.johnston@vanderbilt.edu

Analytical Traces for Final Compounds

Table of Contents

Column and Method Information 2
Detection Methods 2
Method Optimization 2
Analog 1.1 5
Analog 1.2 6
Analog 1.3 7
Analog 1.4 8
Analog 1.5 9
Analog 2.1 10
Analog 2.2 11
Analog 2.3 12
Analog 2.4 13
Analog 2.5 14
Analog 3.1 15
Analog 3.2 16
Analog 3.3 17
Analog 3.4 18
Analog 3.5 19
Analog 4.2 20
Analog 4.3 21
Analog 4.4 22
Analog 4.5 23
Analog 4.6 24

ND = not detected, [M] = macrocycle
Note: all solutions in 100% DMSO, 1-6 min = solvent front
Traces listed in order: ELSD, MSD1 (300-1000 m/z), MSD2 (700-2000 m/z), UV (254, 230, and 210 nm)

Analog 5.2 25
Analog 5.3 26
Analog 5.4 27
Analog 5.5 28

Column and Method Information

Samples analyzed by LC/MS were performed on an Agilent 6130 single quadrupole LC-MS system. Chromatographic separation was performed on an Agilent Eclipse XD-C18 column (4.6x150mm, 5 µm) with 0.1% (v/v) TFA in deionized water (mobile phase A) and 0.1% (v/v) TFA in acetonitrile (mobile phase B). The following elution gradient was used: 30% B for 0.5 min, 30-75% linear gradient for 0.5 min, 75-88% linear gradient for 1 min, 88-90% linear gradient for 8 min, 90-100% linear gradient for 3 min, 100-30% linear gradient for 2 min. The flow rate was 1 mL/min. The column temperature was 23 °C and the injection volume was 100 µL. The ion source parameters of the mass spectrometer include gas temperature of 300 °C, gas flow of 12 L/min, nebulizer gas pressure of 30 psi, and sheath gas temperature of 200 °C. All peak-picking and integration bounds were manually entered for each spectrum.

Detection Methods

To ensure rigorous experimental design, 3 different forms of analysis were utilized, when possible, for observing compound purity. A Varian 380-ELSD, an Agilent 6130 single quadrupole LC-MS system and an Agilent 1100 series HPLC were used for the data collection. For analysis of the HPLC traces, a 210 nm wavelength spectrum was used, which provided the strongest signals. For some macrocycles, only 2 of the 3 methods of quantification showed peaks, so in those cases no detection (ND) was recorded. Many of the macrocycles were detected with UV, ELSD and MS: analogs 2.3, 2.5, 4.1-4.6, and 5.6. Some of the analogs could only be detected by UV and ELSD: *ent*-vert, 1.1-1.5, 2.1, 2.2, 3.1, 5.4, and 5.5. Finally, the remaining analogs were only detected by UV and MS: 2.4, 3.2-3.5 and 5.1-5.3.

Detection Method	Series 1					Series 2					Series 3					Series 4						Series 5					
	1	2	3	4	5	1	2	3	4	5	1	2	3	4	5	1	2	3	4	5	6	1	2	3	4	5	6
UV	✓	✓	✓	✓	✓	✓	✓	✓	✓	✓	✓	✓	✓	✓	✓	✓	✓	✓	✓	✓	✓	✓	✓	✓	✓	✓	✓
ELSD	✓	✓	✓	✓	✓	✓	✓	✓		✓	✓					✓	✓	✓	✓	✓	✓				✓	✓	✓
MS								✓	✓	✓		✓	✓	✓	✓	✓	✓	✓	✓	✓	✓	✓	✓	✓			✓

Supplement Table 1. Methods of Analysis. *Each of the series were quantified using multiple detection methods. For series 1 through series 5, this summarizes the different methods and data to be reported within this supporting information. Due to lack of peaks in the experimental traces, not all methods could be used for every analog (blank = ND = not detected).*

Method Optimization

Due to a lack of significant chromophore, detection of these macrocyclic compounds was minimal by UV, even at higher concentrations (250-500 uM in 100% DMSO). Many samples had significant peak broadening, but the method was optimized with the available analytical system. Shown below is an example trace that was used to optimize the eluent gradient for *ent*-verticilide and analogs. Both of the following traces are 50 µM *ent*-verticilide in DMSO. The following elution gradients were used. The initial gradient (pre-optimization) was 30%-90% linear gradient of B (MeCN) for 5 min, 90-100% linear gradient for 8 min, 100-30% linear gradient for 2 min. The trace shown below is at 210 nm and the macrocycle elutes around 7 minutes. Many different gradients were tested, and the final conditions are as follows: 30% B for 0.5 min, 30-75% linear gradient for 0.5 min, 75-88% linear gradient for 1 min, 88-90% linear

ND = not detected, [M] = macrocycle
Note: all solutions in 100% DMSO, 1-6 min = solvent front
Traces listed in order: ELSD, MSD1 (300-1000 m/z), MSD2 (700-2000 m/z), UV (254, 230, and 210 nm)

gradient for 8 min, 90-100% linear gradient for 3 min, 100-30% linear gradient for 2 min. The trace shown below is at 210 nm and the macrocycle elutes around 10 minutes. All analogs produced similar traces to the example that follows.

ND = not detected, [M] = macrocycle
Note: all solutions in 100% DMSO, 1-6 min = solvent front
Traces listed in order: ELSD, MSD1 (300-1000 m/z), MSD2 (700-2000 m/z), UV (254, 230, and 210 nm)

A. *ent*-verticilide: HPLC trace pre-optimization (λ = 210 nm)

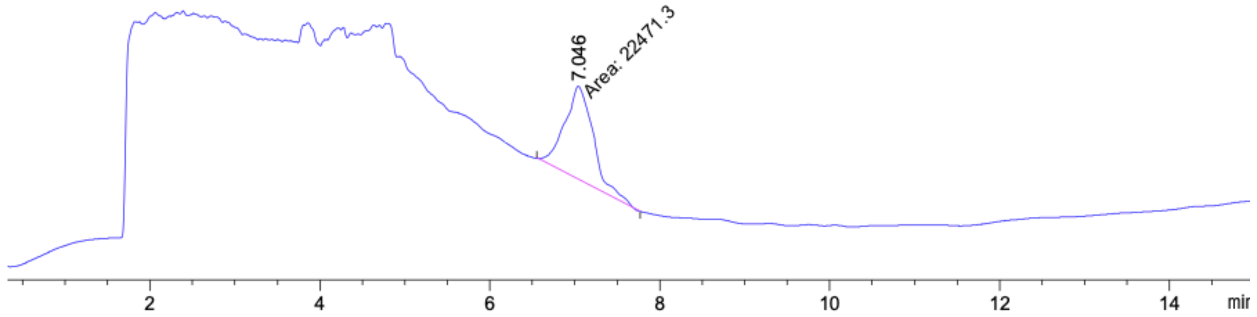

B. *ent*-verticilide: HPLC trace post-optimization (λ = 210 nm)

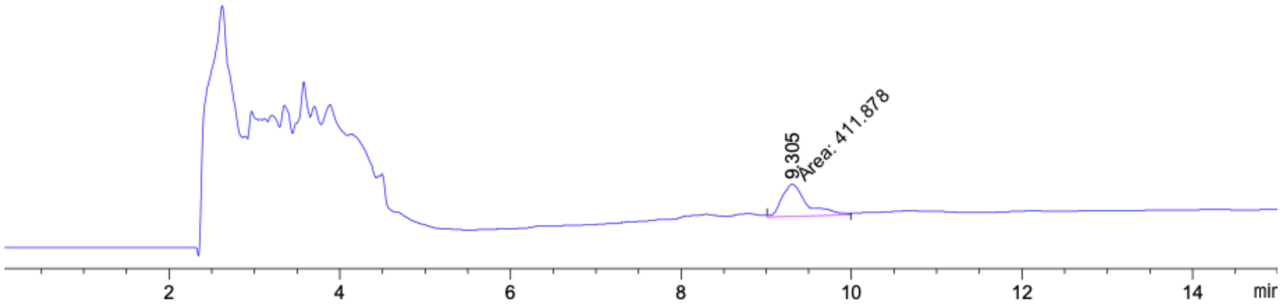

C. *ent*-verticilide: MSD trace

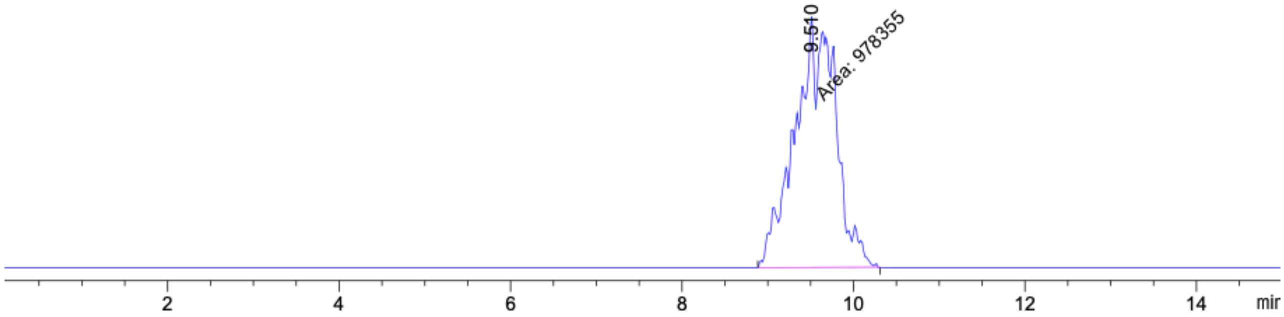

D. *ent*-verticilide: ELSD trace

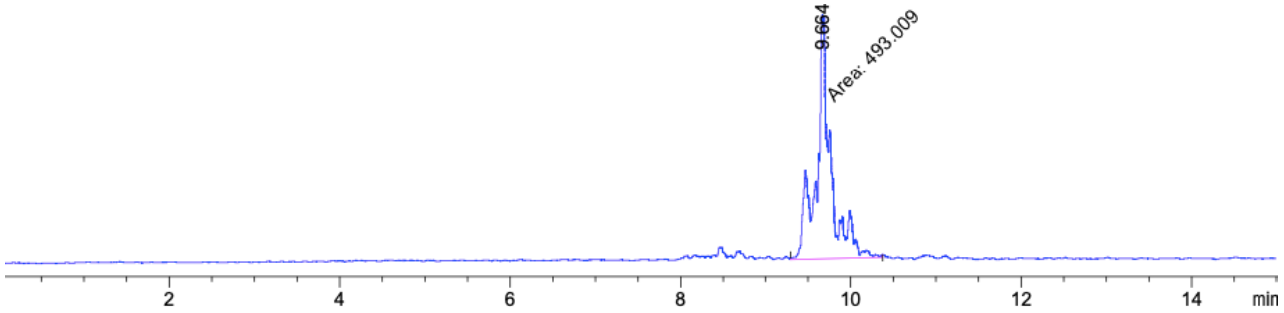

Supplement Figure 1. Traces from a 50 μM sample of ent-verticilide in DMSO. *Analyte peaks are integrated in each spectrum.* ***A*** *includes the trace using pre-optimization conditions. Analyte is found at 7 minutes.* ***B*** *is the HPLC trace using post-optimization conditions.* ***C*** *is the MSD trace using post-optimization conditions.* ***D*** *is the ELSD trace using post-optimization conditions. B-C shows the analyte eluting at 9.5-10 minutes.*

ND = not detected, [M] = macrocycle
Note: all solutions in 100% DMSO, 1-6 min = solvent front
Traces listed in order: ELSD, MSD1 (300-1000 m/z), MSD2 (700-2000 m/z), UV (254, 230, and 210 nm)

Analog 1.1

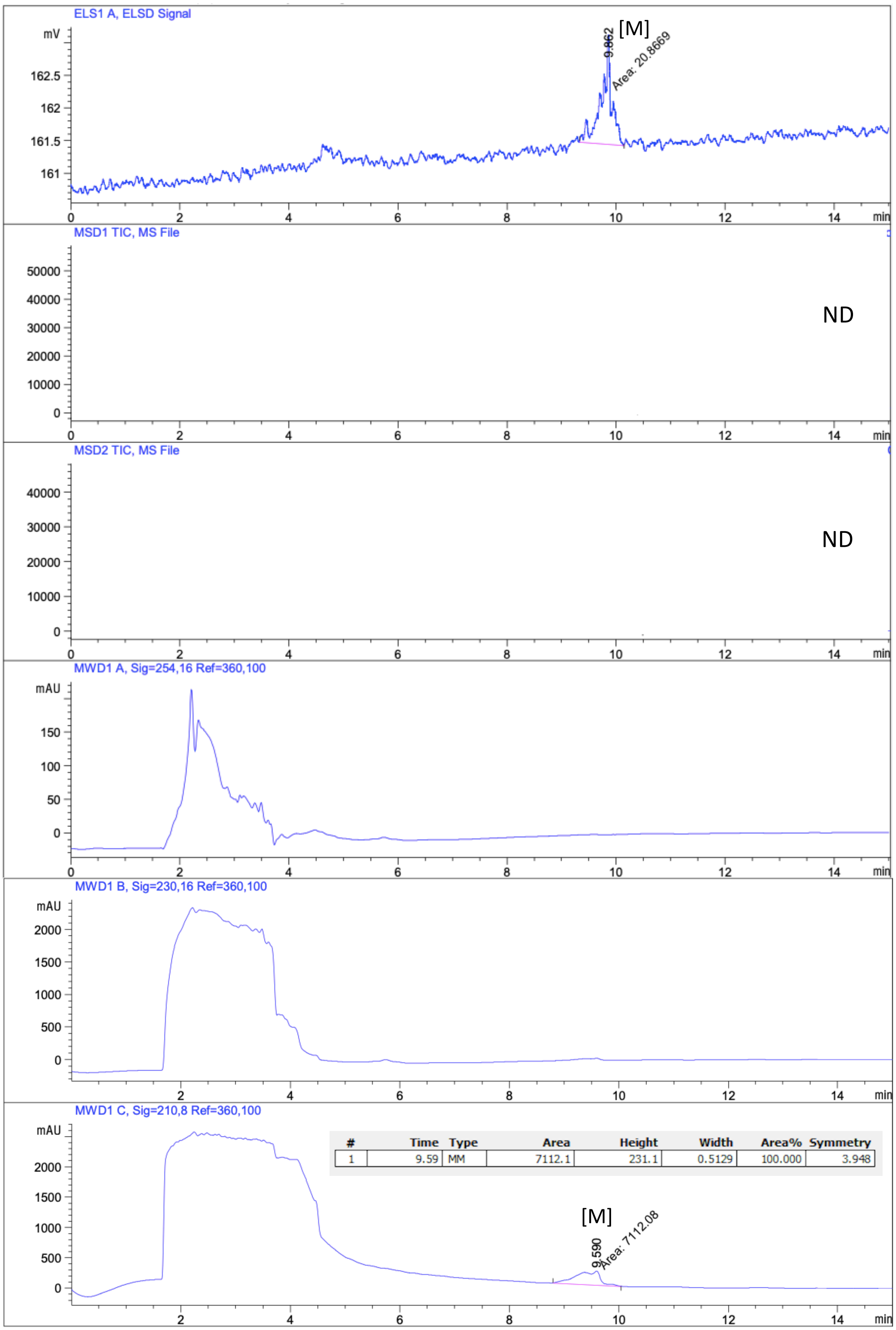

#	Time	Type	Area	Height	Width	Area%	Symmetry
1	9.59	MM	7112.1	231.1	0.5129	100.000	3.948

ND = not detected, [M] = macrocycle
Note: all solutions in 100% DMSO, 1-6 min = solvent front
Traces listed in order: ELSD, MSD1 (300-1000 m/z), MSD2 (700-2000 m/z), UV (254, 230, and 210 nm)

Analog 1.2

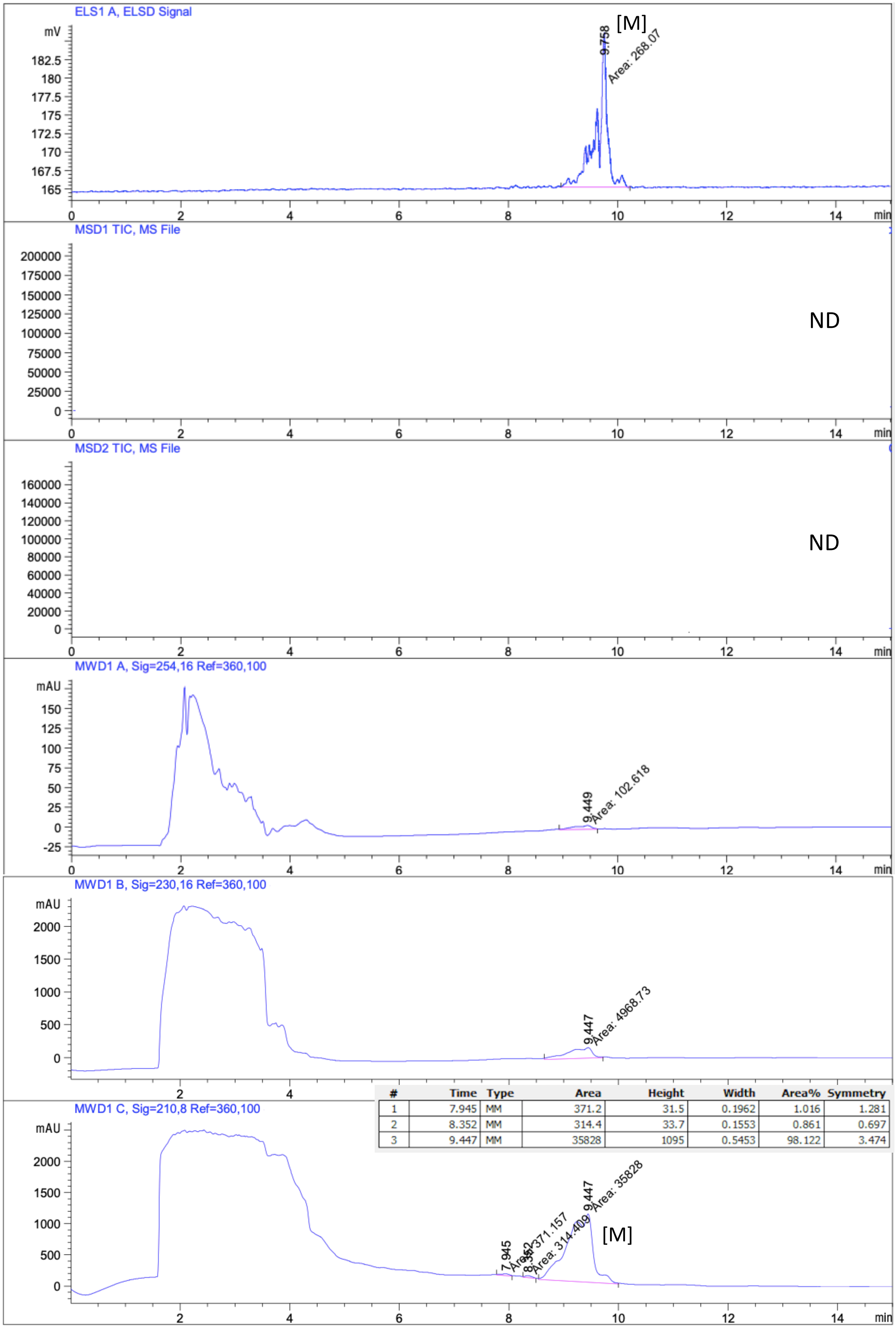

#	Time	Type	Area	Height	Width	Area%	Symmetry
1	7.945	MM	371.2	31.5	0.1962	1.016	1.281
2	8.352	MM	314.4	33.7	0.1553	0.861	0.697
3	9.447	MM	35828	1095	0.5453	98.122	3.474

ND = not detected, [M] = macrocycle
Note: all solutions in 100% DMSO, 1-6 min = solvent front
Traces listed in order: ELSD, MSD1 (300-1000 m/z), MSD2 (700-2000 m/z), UV (254, 230, and 210 nm)

Analog 1.3

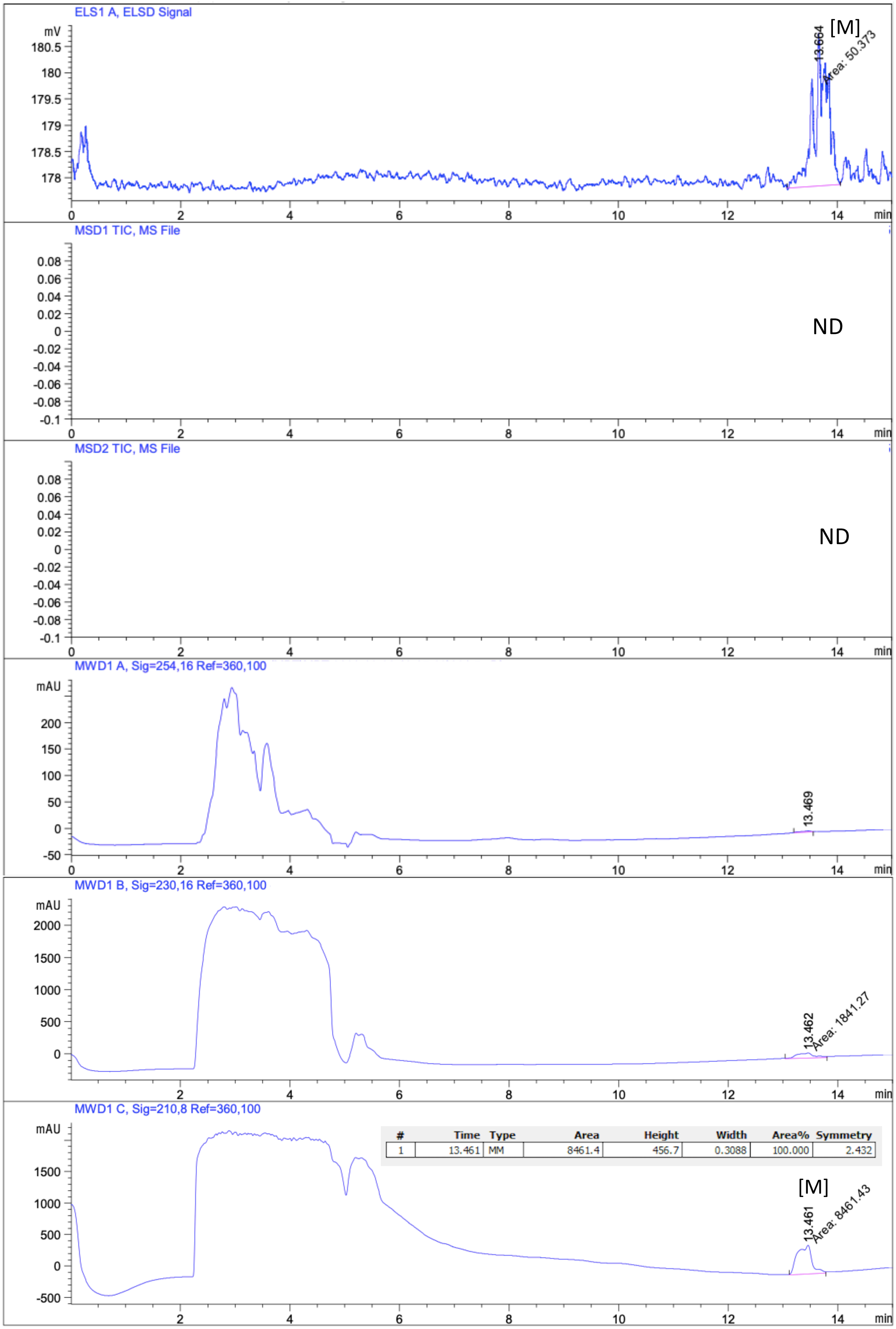

ND = not detected, [M] = macrocycle

Note: all solutions in 100% DMSO, 1-6 min = solvent front

Traces listed in order: ELSD, MSD1 (300-1000 m/z), MSD2 (700-2000 m/z), UV (254, 230, and 210 nm)

Analog 1.4

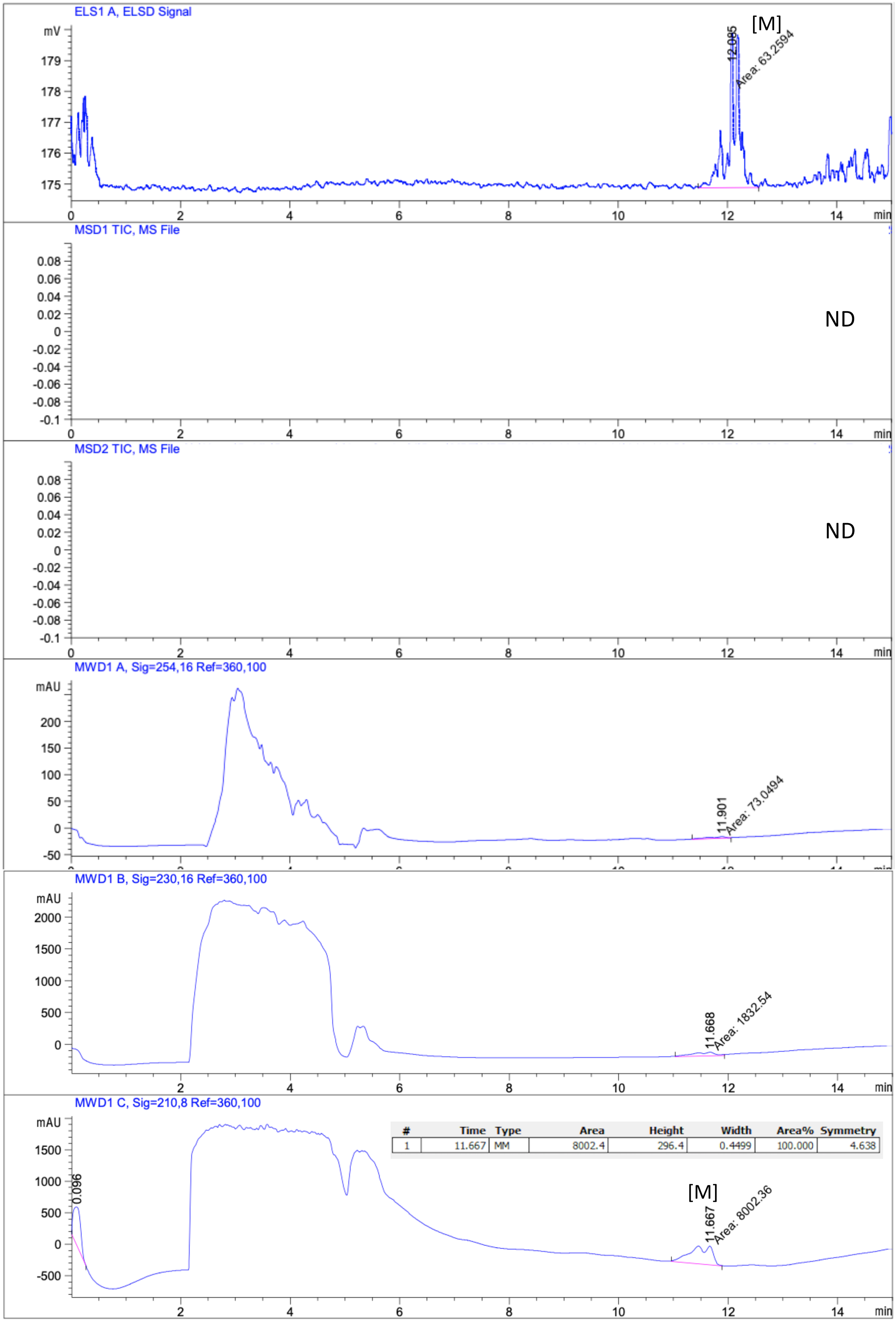

#	Time	Type	Area	Height	Width	Area%	Symmetry
1	11.667	MM	8002.4	296.4	0.4499	100.000	4.638

ND = not detected, [M] = macrocycle

Note: all solutions in 100% DMSO, 1-6 min = solvent front

Traces listed in order: ELSD, MSD1 (300-1000 m/z), MSD2 (700-2000 m/z), UV (254, 230, and 210 nm)

Analog 1.5

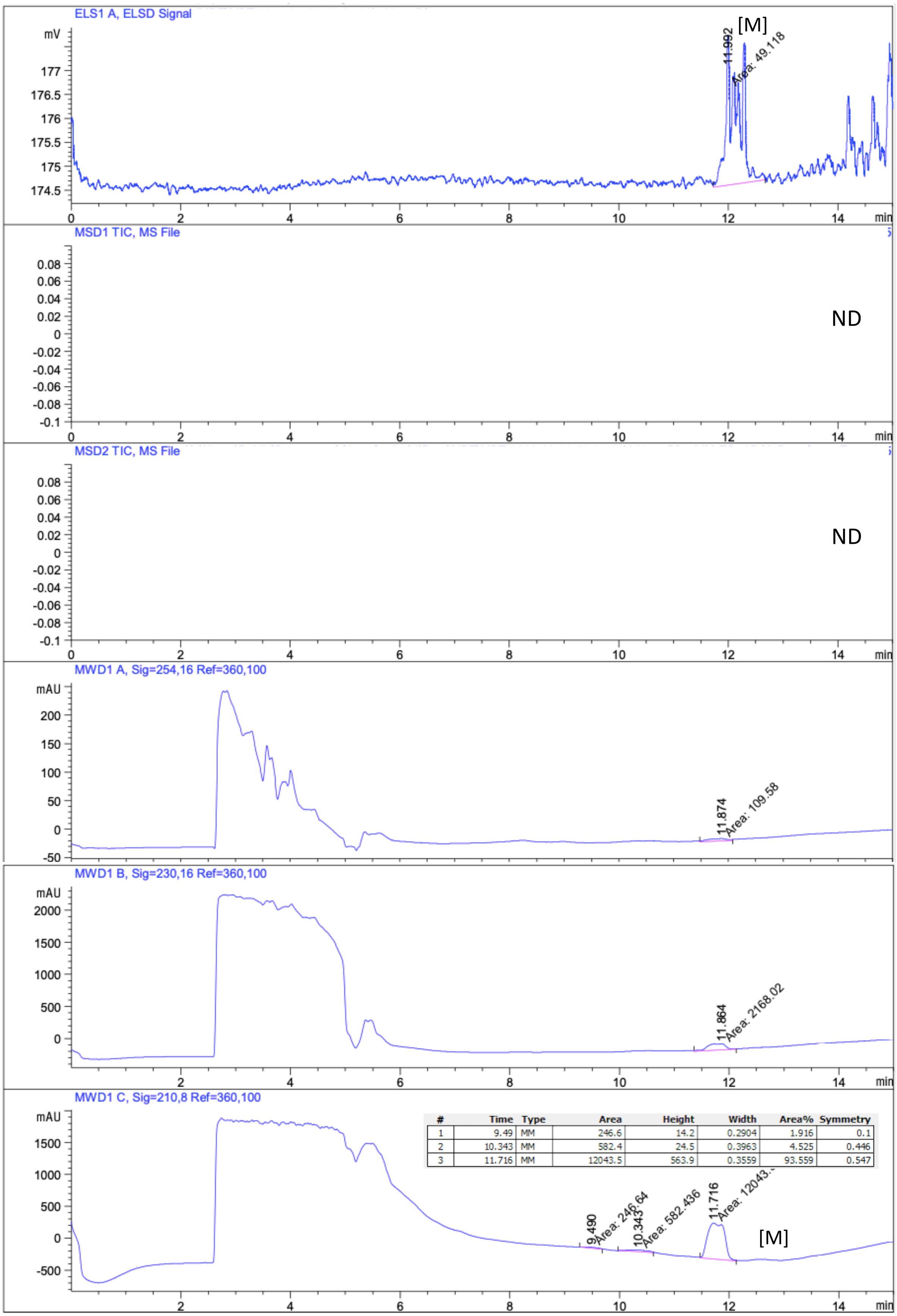

#	Time	Type	Area	Height	Width	Area%	Symmetry
1	9.49	MM	246.6	14.2	0.2904	1.916	0.1
2	10.343	MM	582.4	24.5	0.3963	4.525	0.446
3	11.716	MM	12043.5	563.9	0.3559	93.559	0.547

ND = not detected, [M] = macrocycle

Note: all solutions in 100% DMSO, 1-6 min = solvent front

Traces listed in order: ELSD, MSD1 (300-1000 m/z), MSD2 (700-2000 m/z), UV (254, 230, and 210 nm)

Analog 2.1

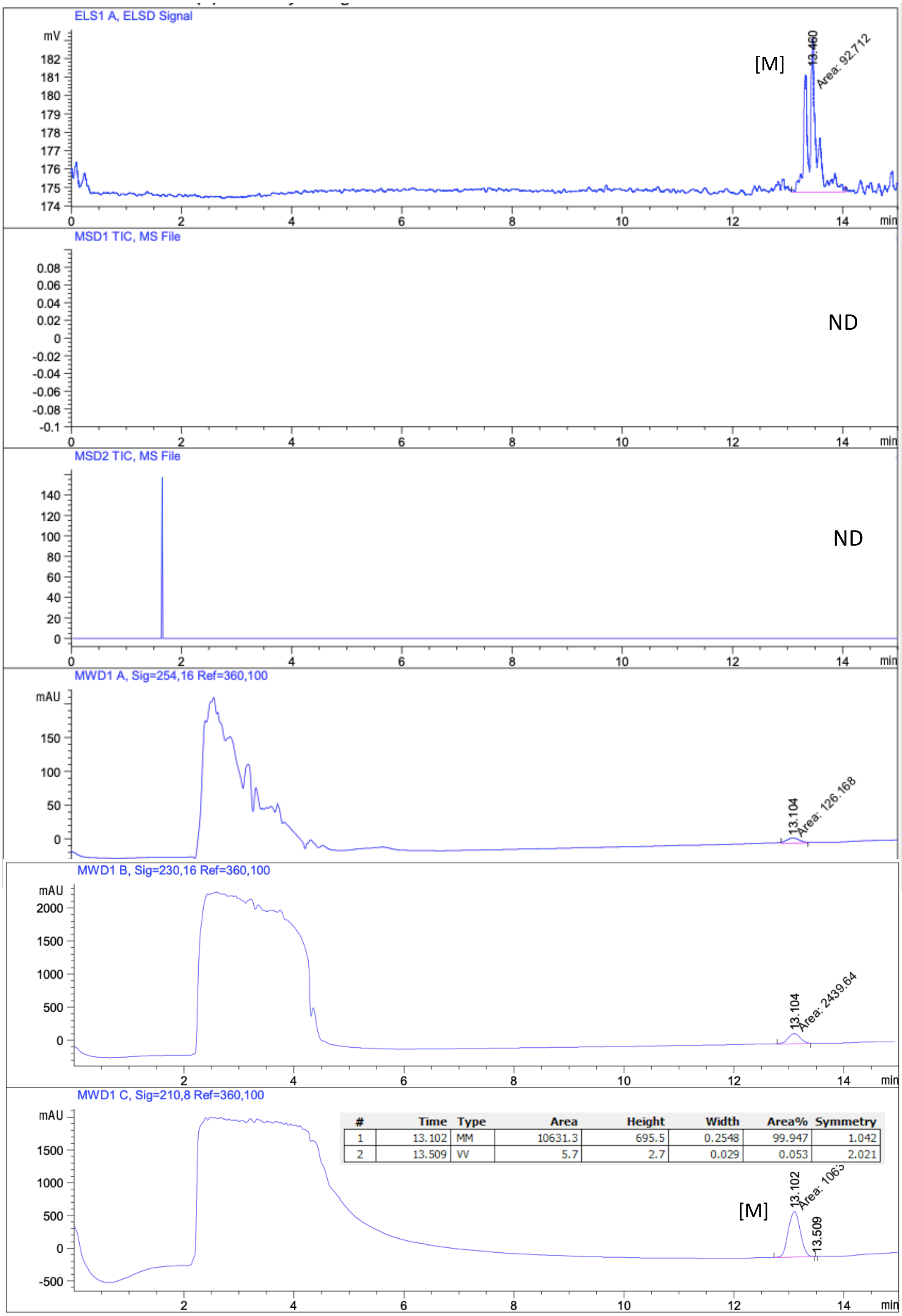

#	Time	Type	Area	Height	Width	Area%	Symmetry
1	13.102	MM	10631.3	695.5	0.2548	99.947	1.042
2	13.509	VV	5.7	2.7	0.029	0.053	2.021

ND = not detected, [M] = macrocycle

Note: all solutions in 100% DMSO, 1-6 min = solvent front

Traces listed in order: ELSD, MSD1 (300-1000 m/z), MSD2 (700-2000 m/z), UV (254, 230, and 210 nm)

Analog 2.2

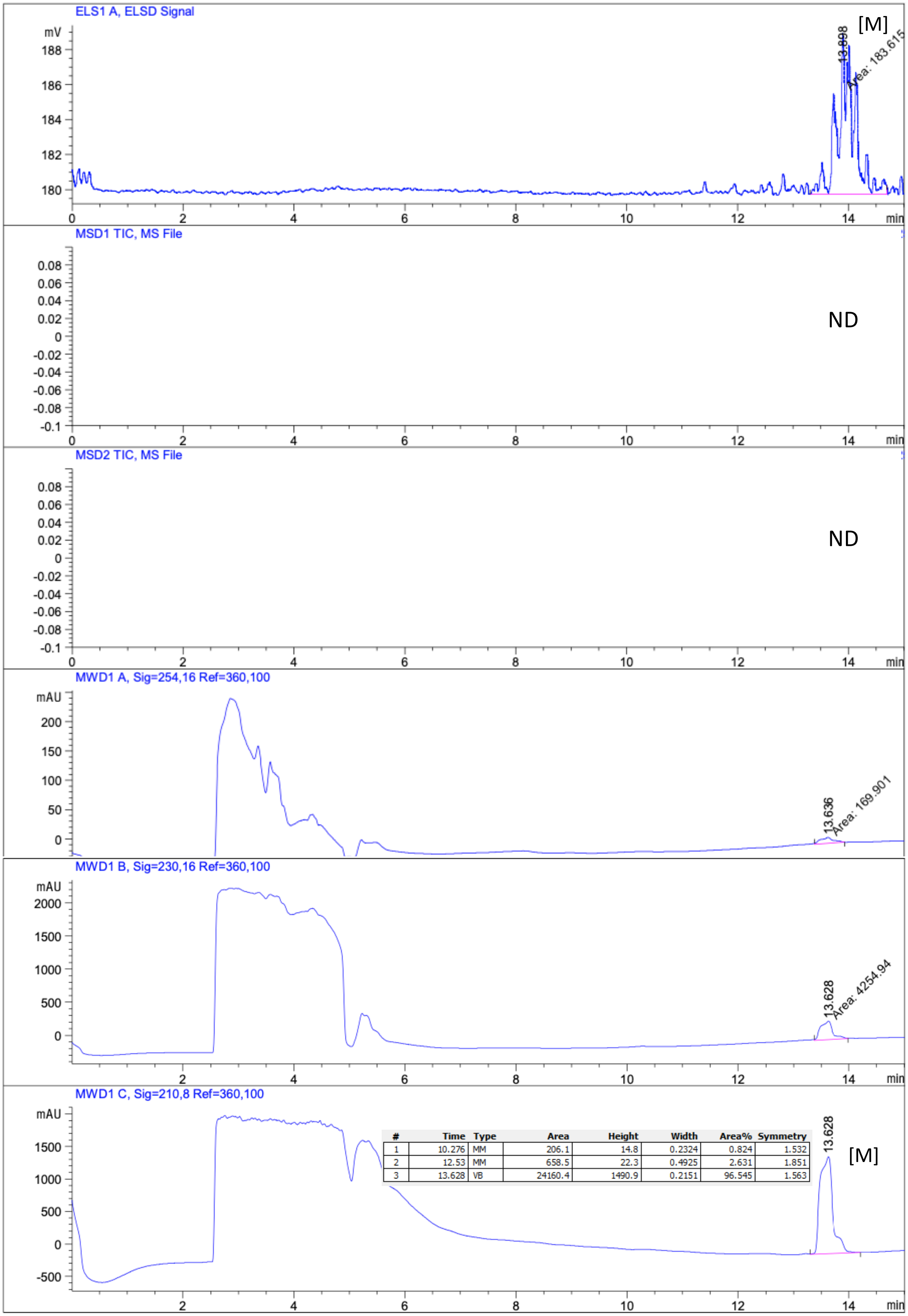

#	Time	Type	Area	Height	Width	Area%	Symmetry
1	10.276	MM	206.1	14.8	0.2324	0.824	1.532
2	12.53	MM	658.5	22.3	0.4925	2.631	1.851
3	13.628	VB	24160.4	1490.9	0.2151	96.545	1.563

ND = not detected, [M] = macrocycle
Note: all solutions in 100% DMSO, 1-6 min = solvent front
Traces listed in order: ELSD, MSD1 (300-1000 m/z), MSD2 (700-2000 m/z), UV (254, 230, and 210 nm)

Analog 2.3

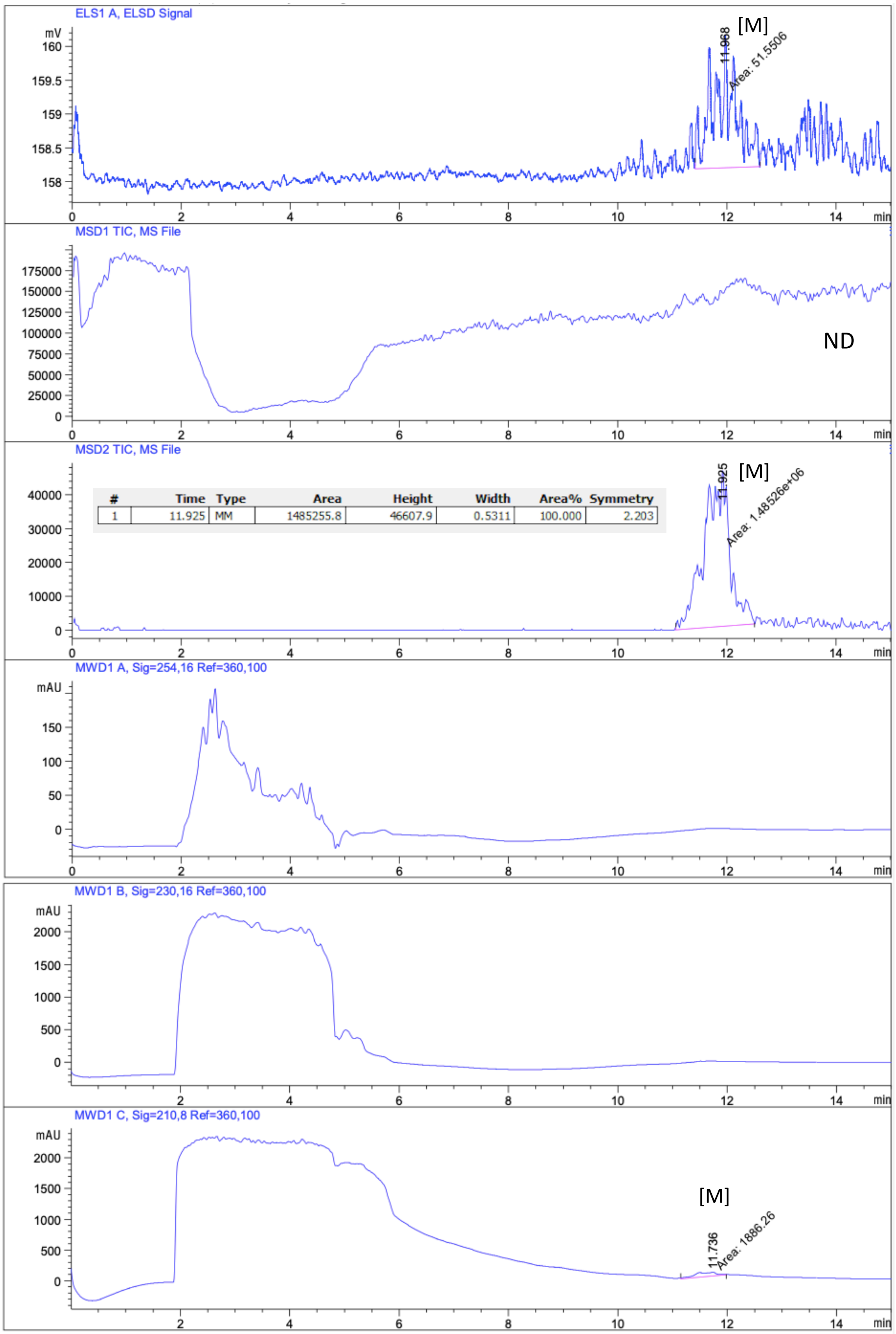

ND = not detected, [M] = macrocycle
Note: all solutions in 100% DMSO, 1-6 min = solvent front
Traces listed in order: ELSD, MSD1 (300-1000 m/z), MSD2 (700-2000 m/z), UV (254, 230, and 210 nm)

Analog 2.4

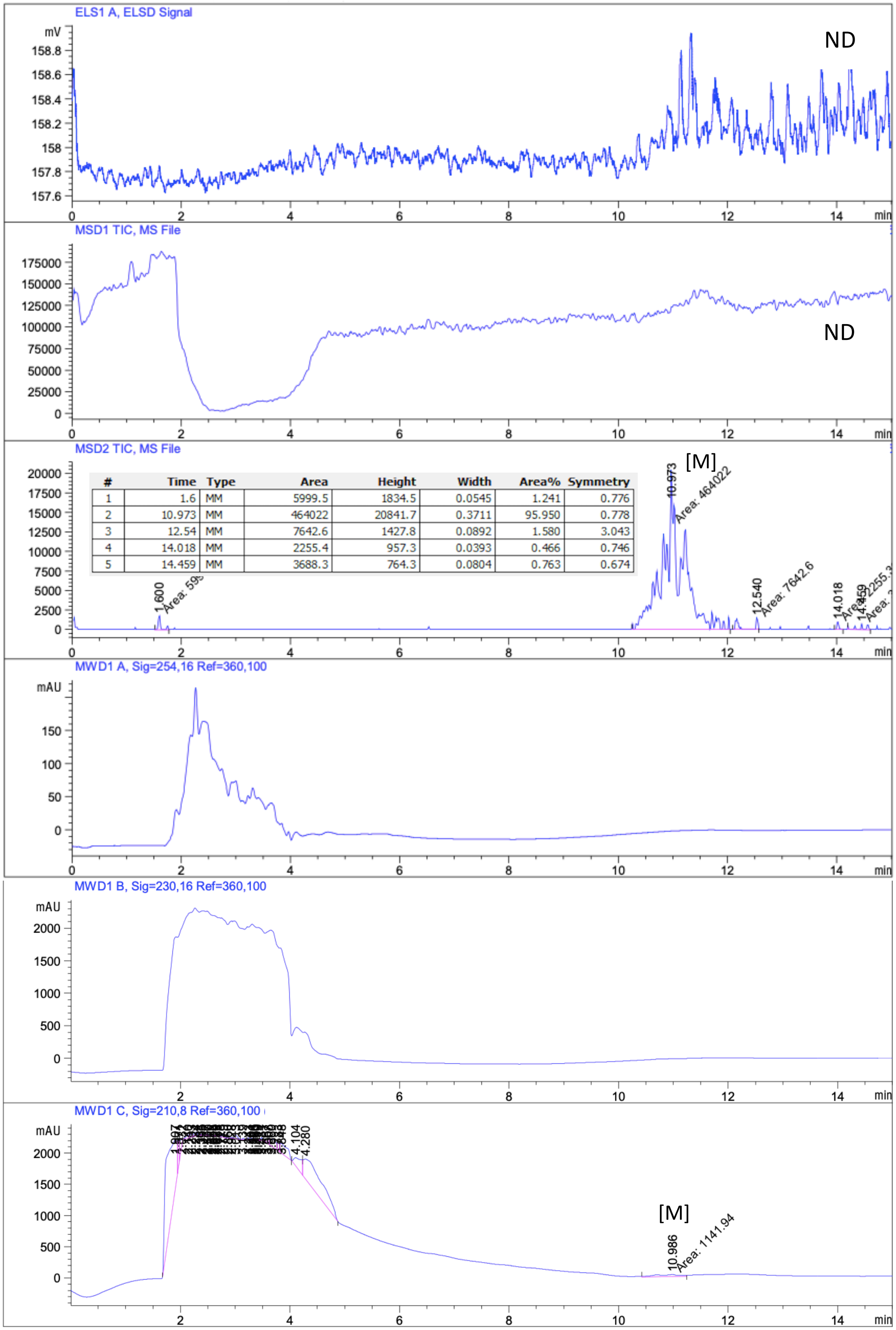

#	Time	Type	Area	Height	Width	Area%	Symmetry
1	1.6	MM	5999.5	1834.5	0.0545	1.241	0.776
2	10.973	MM	464022	20841.7	0.3711	95.950	0.778
3	12.54	MM	7642.6	1427.8	0.0892	1.580	3.043
4	14.018	MM	2255.4	957.3	0.0393	0.466	0.746
5	14.459	MM	3688.3	764.3	0.0804	0.763	0.674

ND = not detected, [M] = macrocycle
Note: all solutions in 100% DMSO, 1-6 min = solvent front
Traces listed in order: ELSD, MSD1 (300-1000 m/z), MSD2 (700-2000 m/z), UV (254, 230, and 210 nm)

Analog 2.5

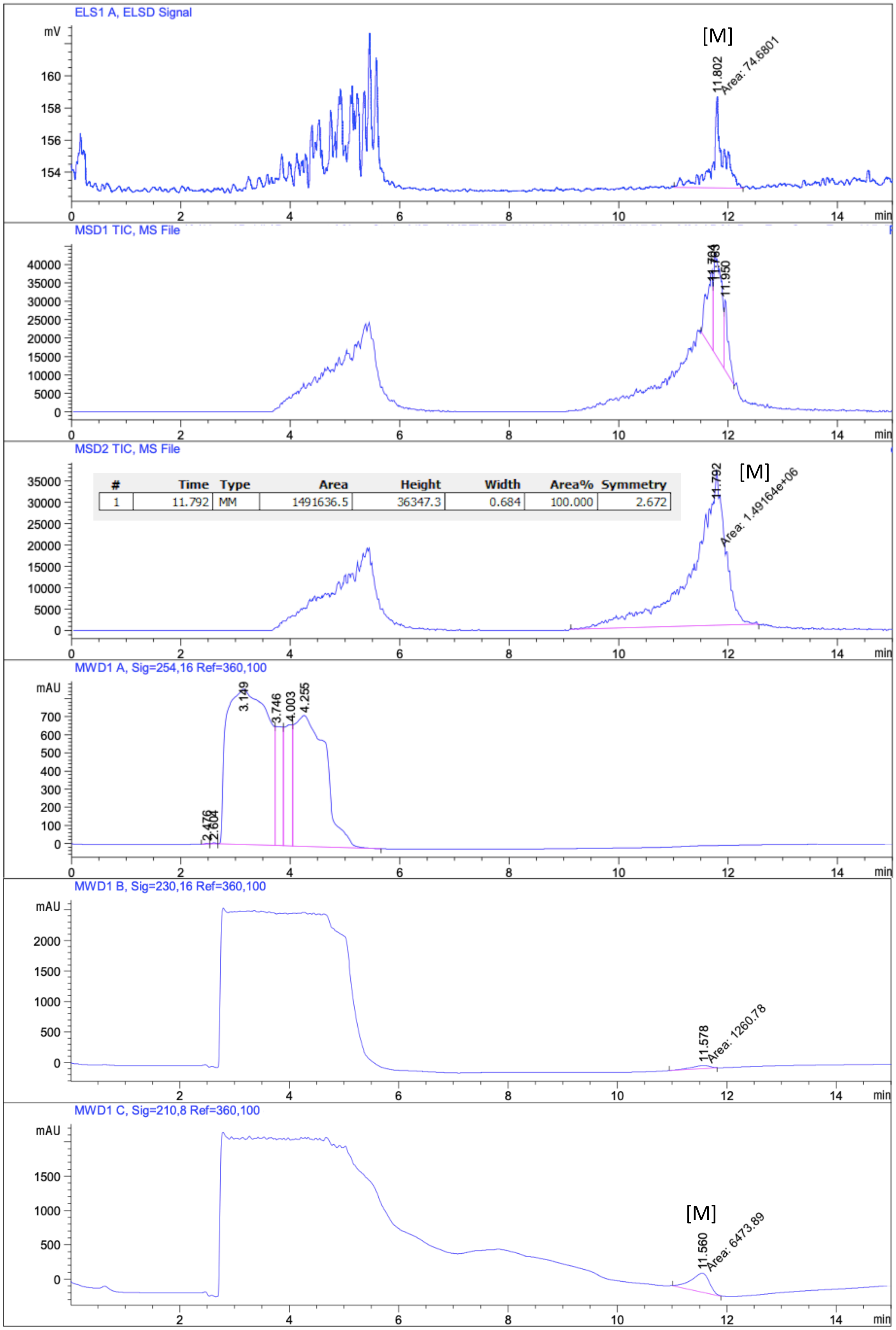

ND = not detected, [M] = macrocycle
Note: all solutions in 100% DMSO, 1-6 min = solvent front
Traces listed in order: ELSD, MSD1 (300-1000 m/z), MSD2 (700-2000 m/z), UV (254, 230, and 210 nm)

Analog 3.1

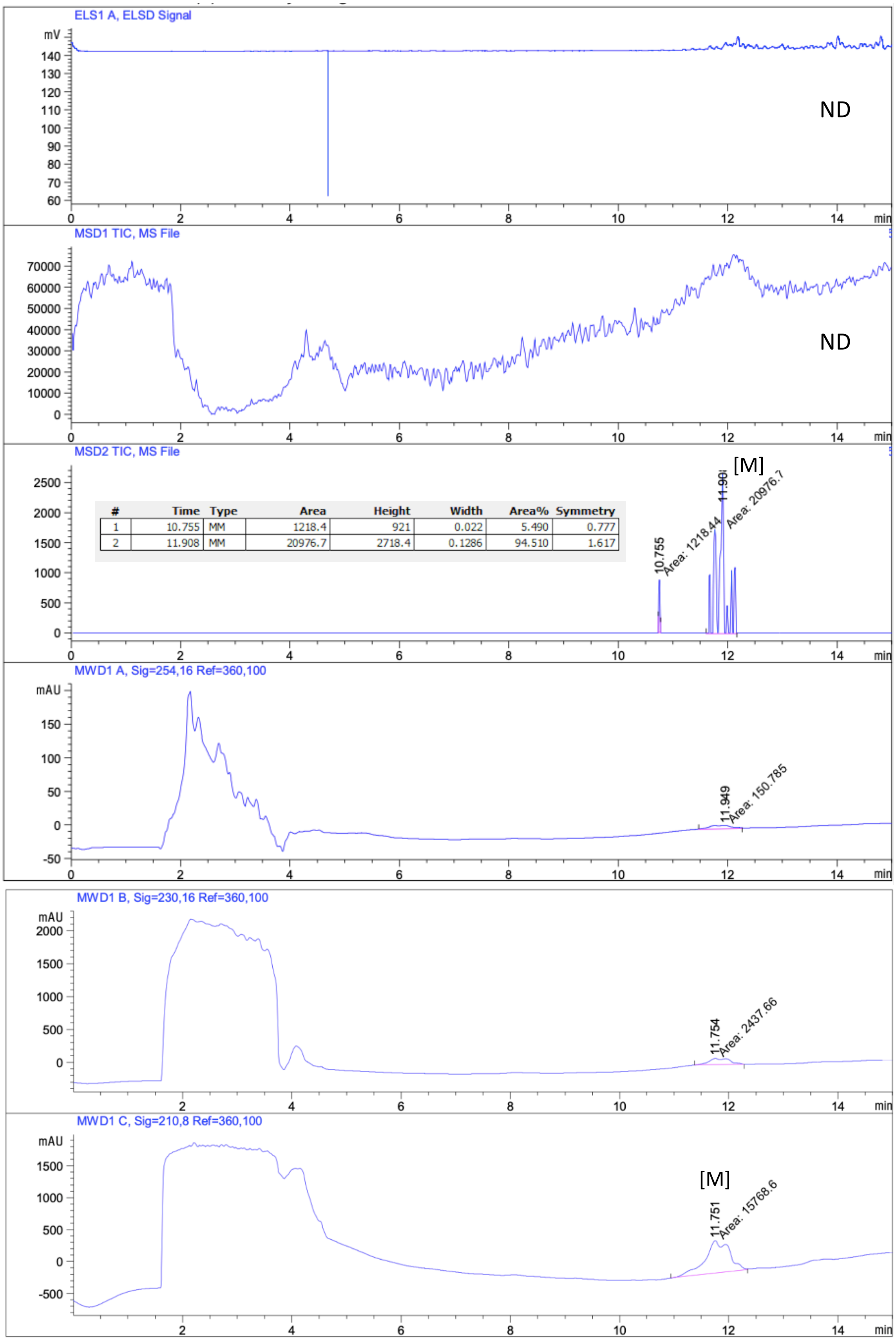

ND = not detected, [M] = macrocycle

Note: all solutions in 100% DMSO, 1-6 min = solvent front

Traces listed in order: ELSD, MSD1 (300-1000 m/z), MSD2 (700-2000 m/z), UV (254, 230, and 210 nm)

Analog 3.2

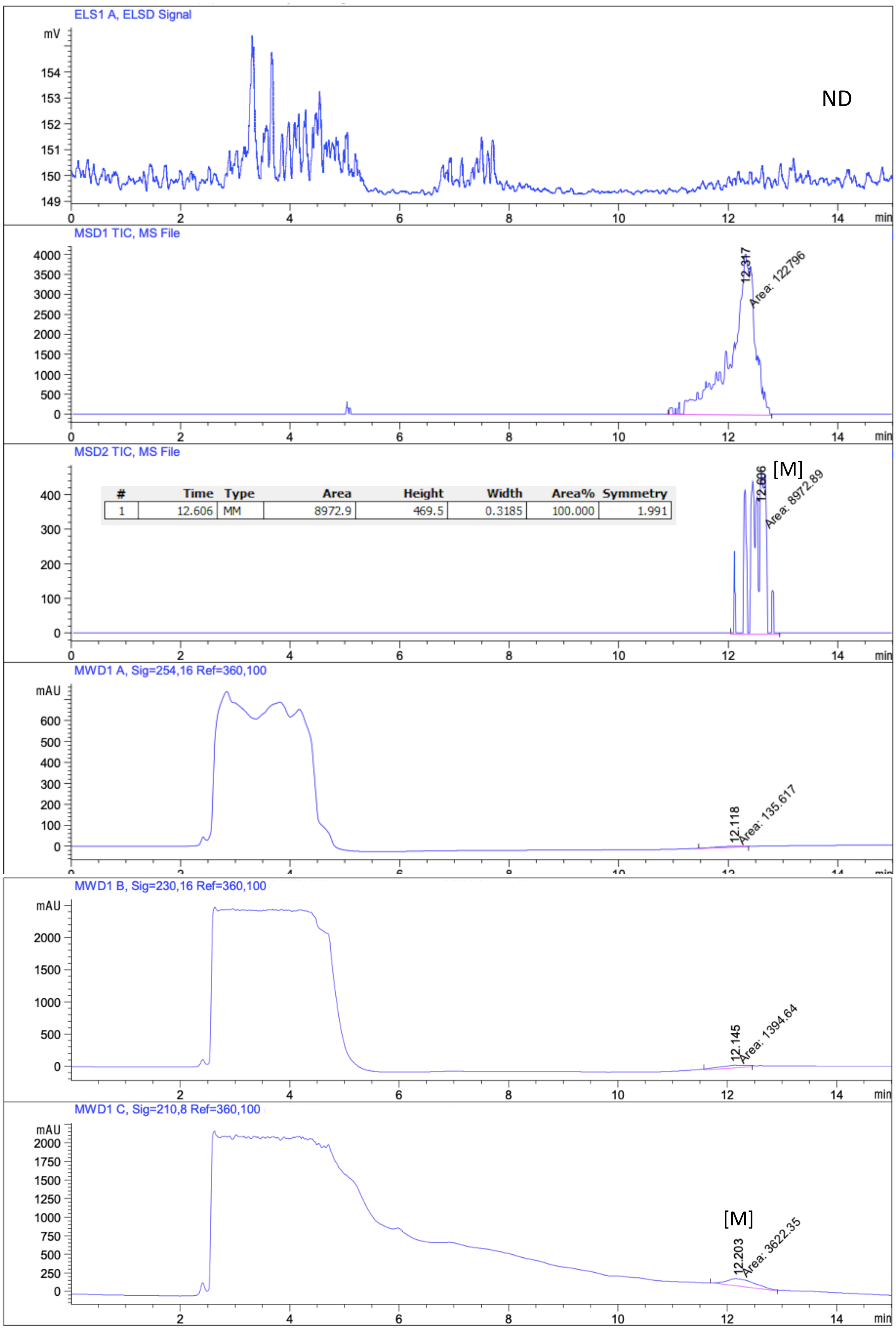

ND = not detected, [M] = macrocycle

Note: all solutions in 100% DMSO, 1-6 min = solvent front

Traces listed in order: ELSD, MSD1 (300-1000 m/z), MSD2 (700-2000 m/z), UV (254, 230, and 210 nm)

Analog 3.3

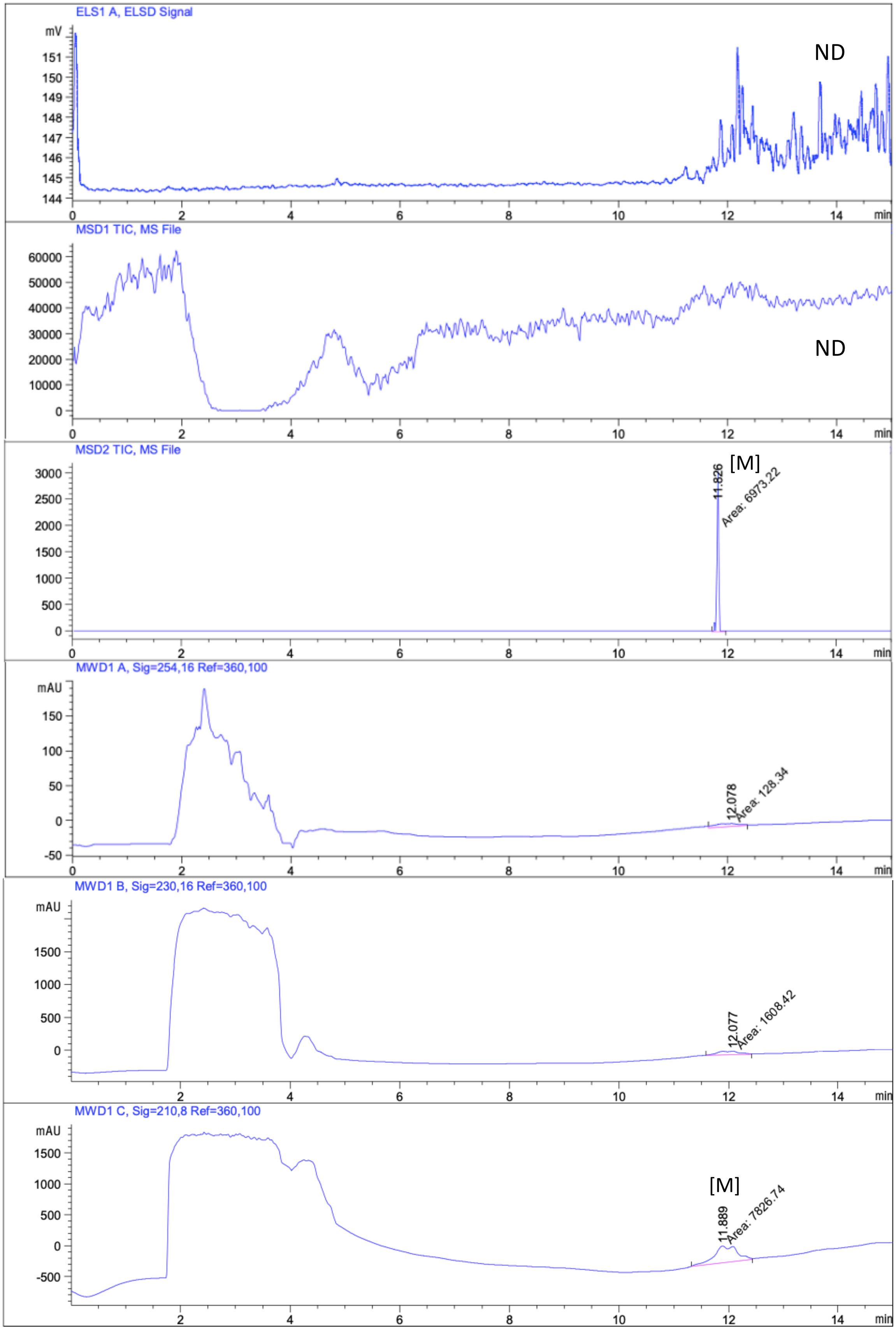

ND = not detected, [M] = macrocycle

Note: all solutions in 100% DMSO, 1-6 min = solvent front

Traces listed in order: ELSD, MSD1 (300-1000 m/z), MSD2 (700-2000 m/z), UV (254, 230, and 210 nm)

Analog 3.4

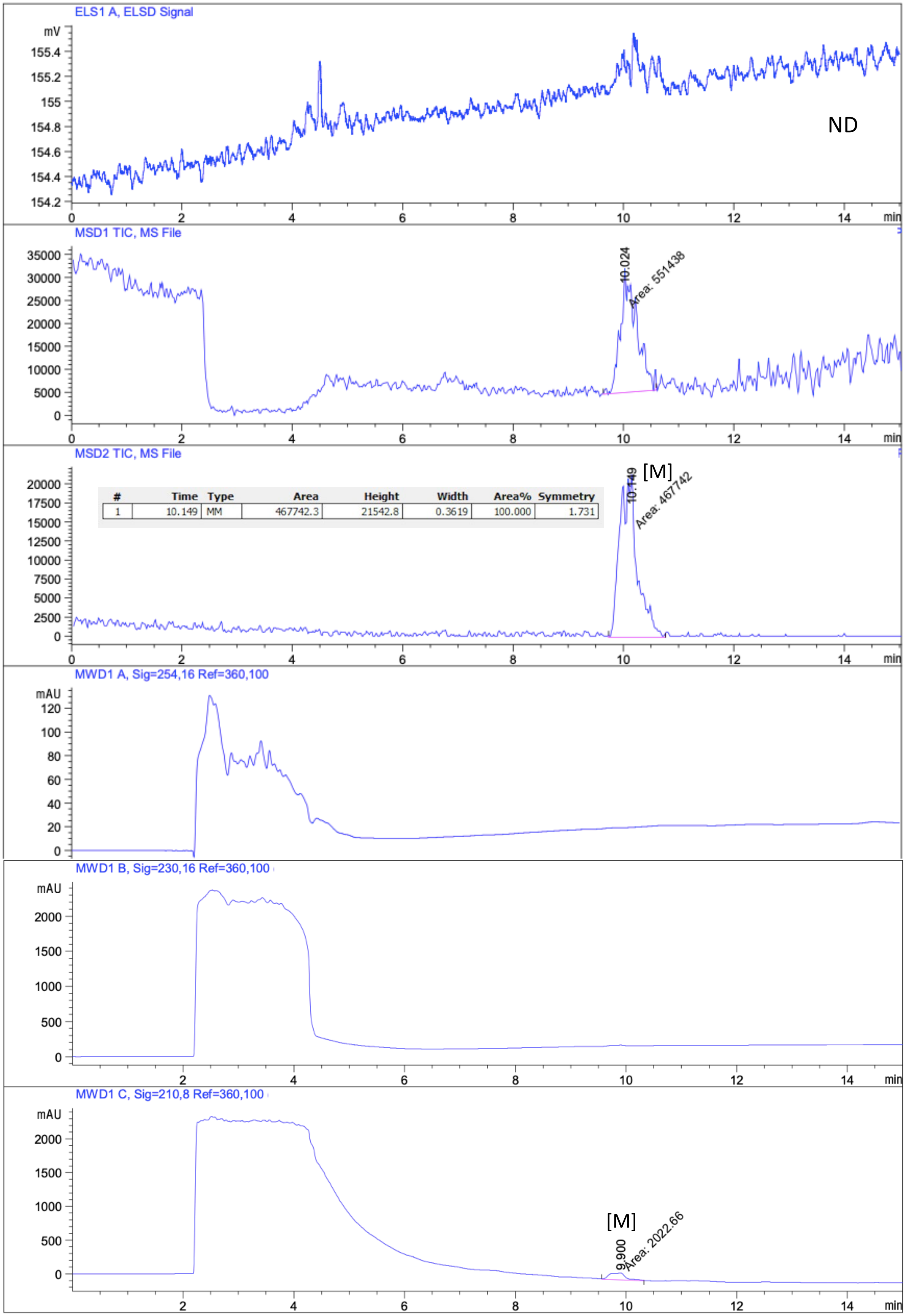

ND = not detected, [M] = macrocycle

Note: all solutions in 100% DMSO, 1-6 min = solvent front

Traces listed in order: ELSD, MSD1 (300-1000 m/z), MSD2 (700-2000 m/z), UV (254, 230, and 210 nm)

Analog 3.5

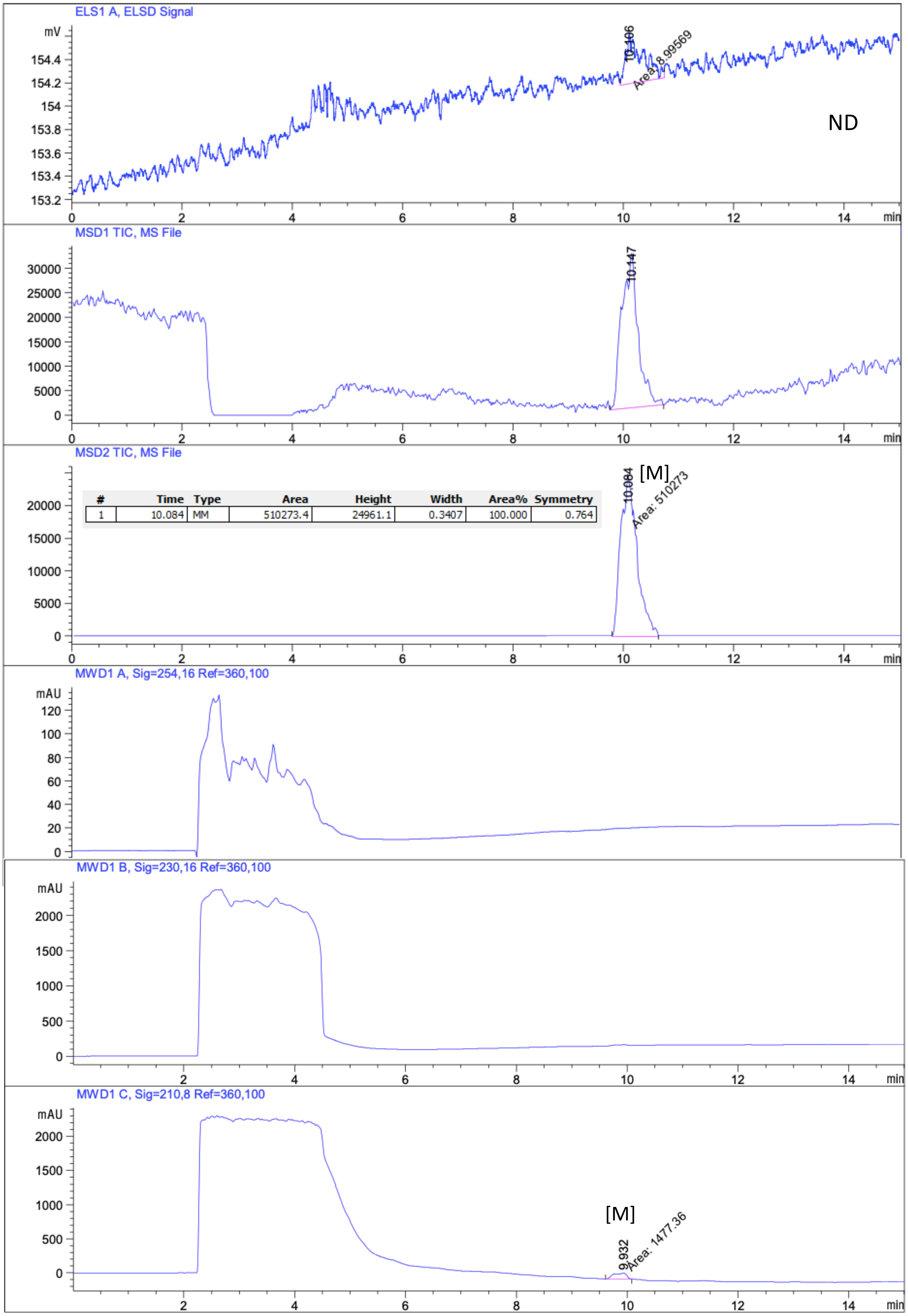

ND = not detected, [M] = macrocycle

Note: all solutions in 100% DMSO, 1-6 min = solvent front

Traces listed in order: ELSD, MSD1 (300-1000 m/z), MSD2 (700-2000 m/z), UV (254, 230, and 210 nm)

Analog 4.2

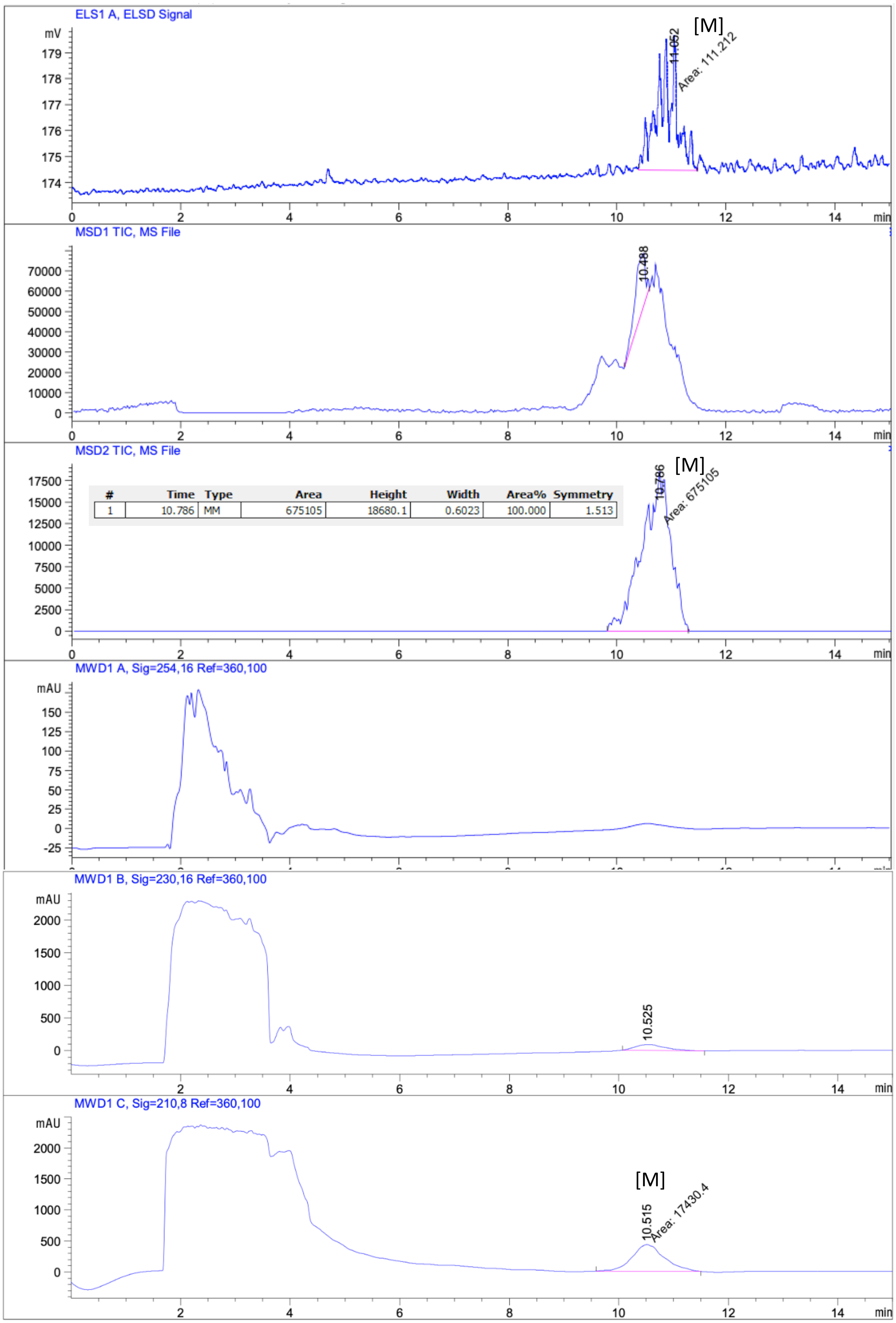

ND = not detected, [M] = macrocycle

Note: all solutions in 100% DMSO, 1-6 min = solvent front

Traces listed in order: ELSD, MSD1 (300-1000 m/z), MSD2 (700-2000 m/z), UV (254, 230, and 210 nm)

Analog 4.3

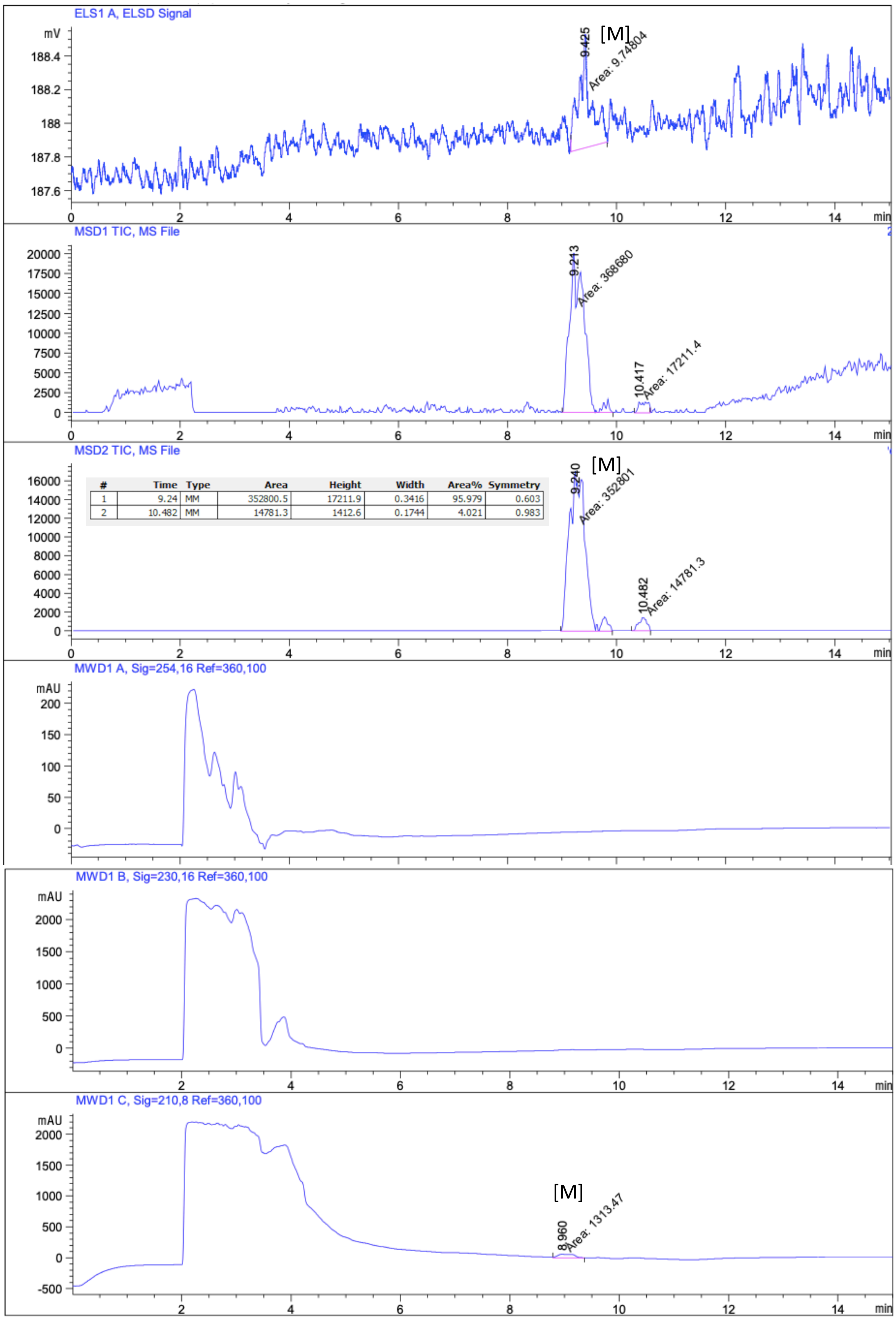

#	Time	Type	Area	Height	Width	Area%	Symmetry
1	9.24	MM	352800.5	17211.9	0.3416	95.979	0.603
2	10.482	MM	14781.3	1412.6	0.1744	4.021	0.983

ND = not detected, [M] = macrocycle

Note: all solutions in 100% DMSO, 1-6 min = solvent front

Traces listed in order: ELSD, MSD1 (300-1000 m/z), MSD2 (700-2000 m/z), UV (254, 230, and 210 nm)

Analog 4.4

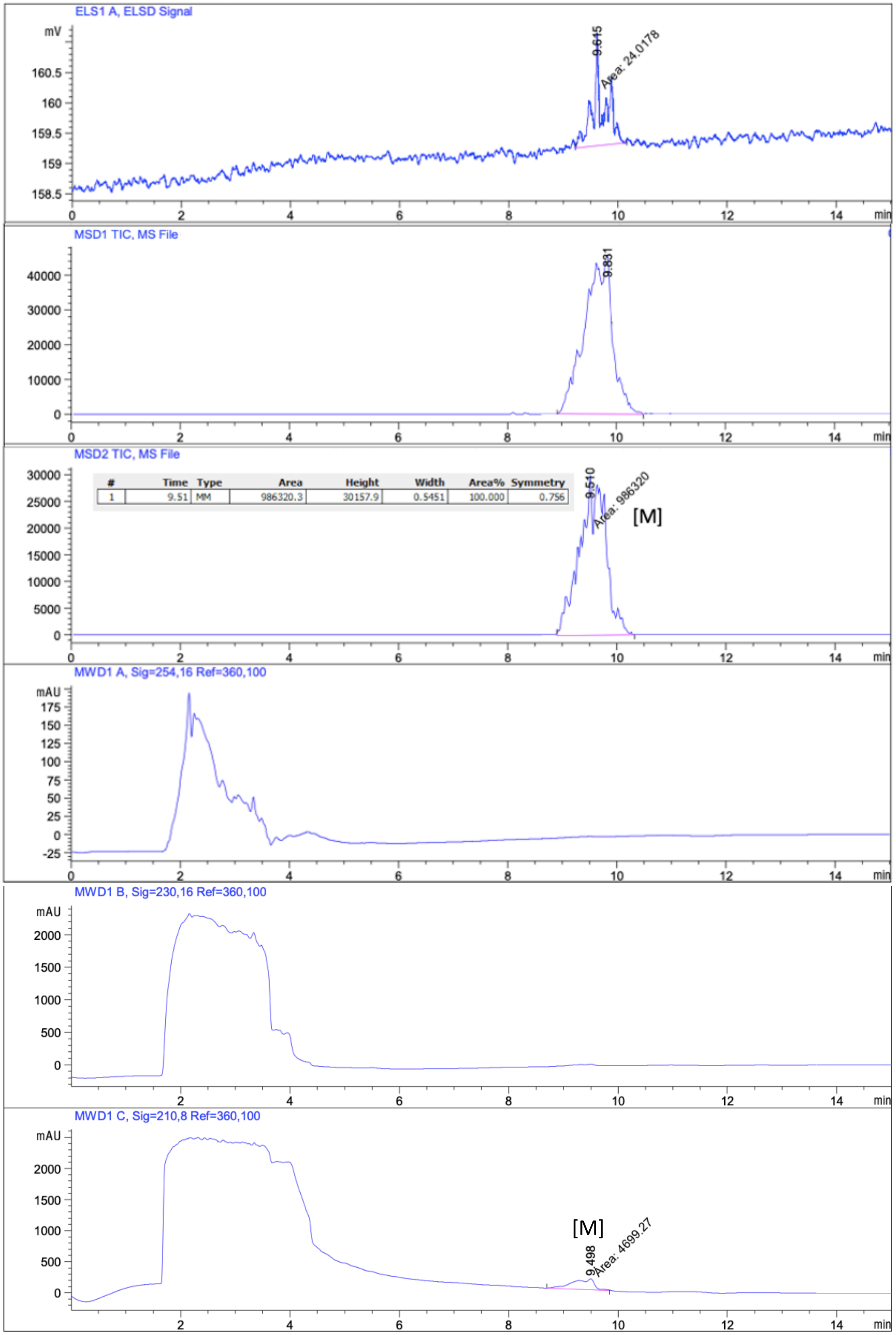

ND = not detected, [M] = macrocycle

Note: all solutions in 100% DMSO, 1-6 min = solvent front

Traces listed in order: ELSD, MSD1 (300-1000 m/z), MSD2 (700-2000 m/z), UV (254, 230, and 210 nm)

Analog 4.5

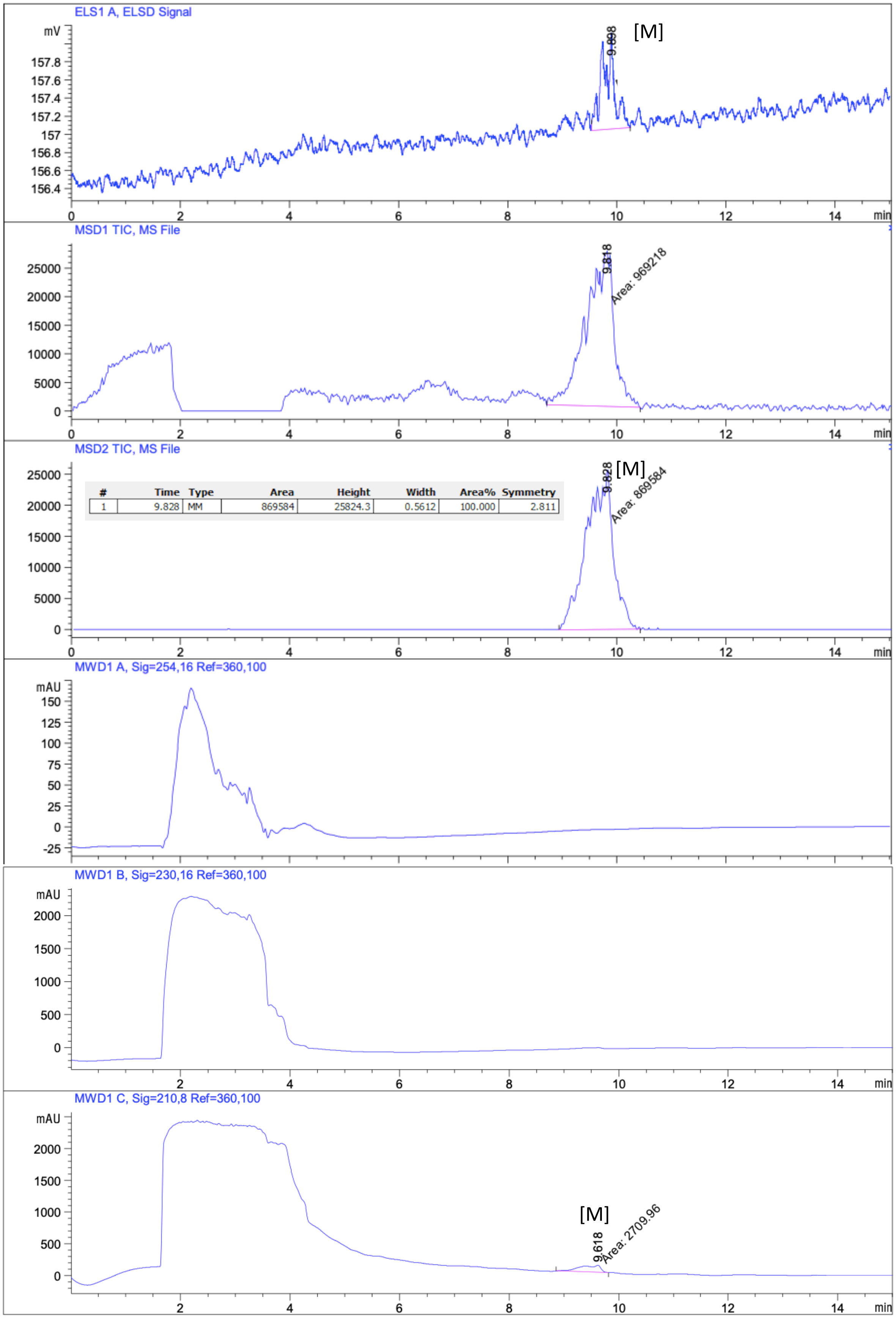

ND = not detected, [M] = macrocycle

Note: all solutions in 100% DMSO, 1-6 min = solvent front

Traces listed in order: ELSD, MSD1 (300-1000 m/z), MSD2 (700-2000 m/z), UV (254, 230, and 210 nm)

Analog 4.6

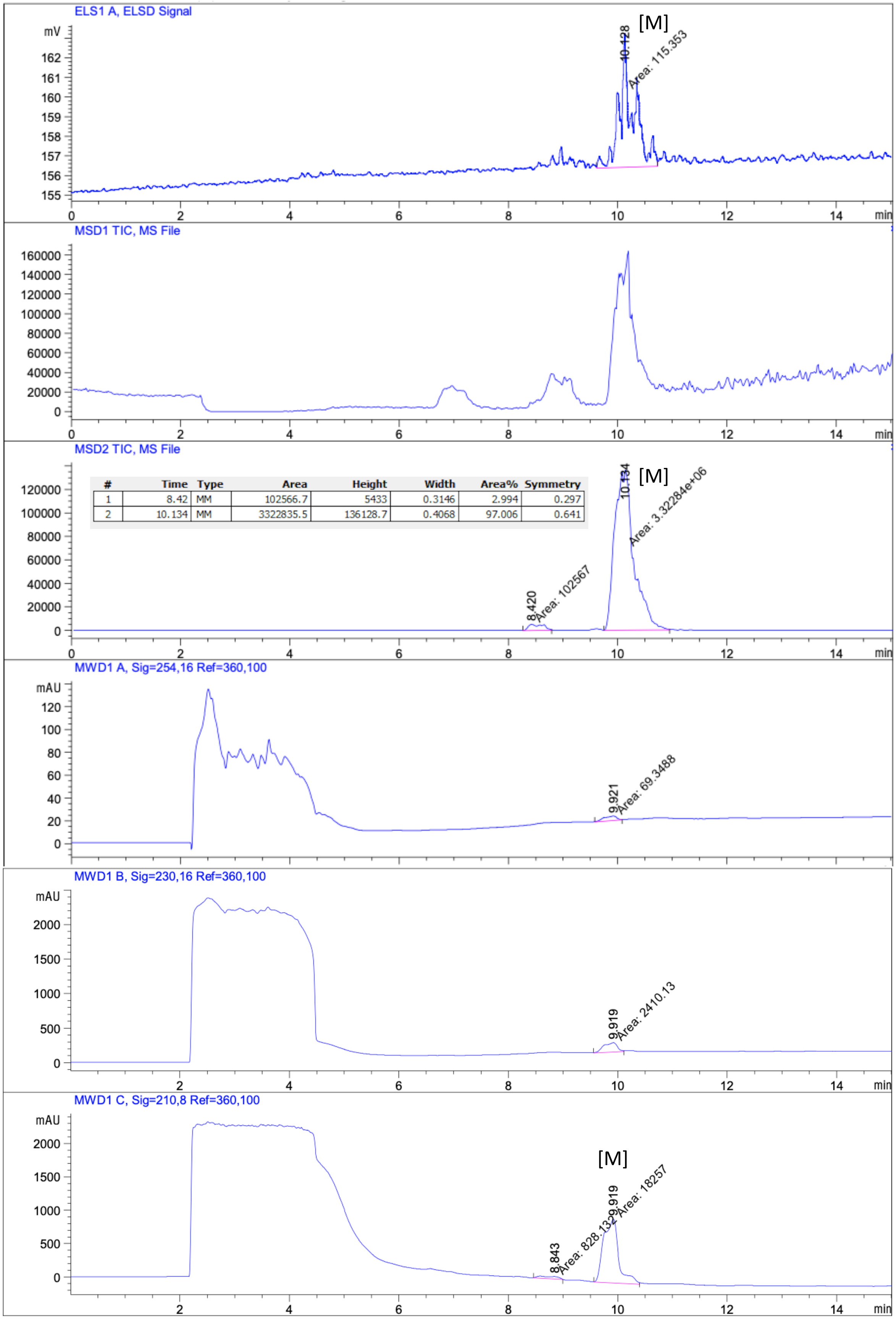

#	Time	Type	Area	Height	Width	Area%	Symmetry
1	8.42	MM	102566.7	5433	0.3146	2.994	0.297
2	10.134	MM	3322835.5	136128.7	0.4068	97.006	0.641

ND = not detected, [M] = macrocycle

Note: all solutions in 100% DMSO, 1-6 min = solvent front

Traces listed in order: ELSD, MSD1 (300-1000 m/z), MSD2 (700-2000 m/z), UV (254, 230, and 210 nm)

Analog 5.2

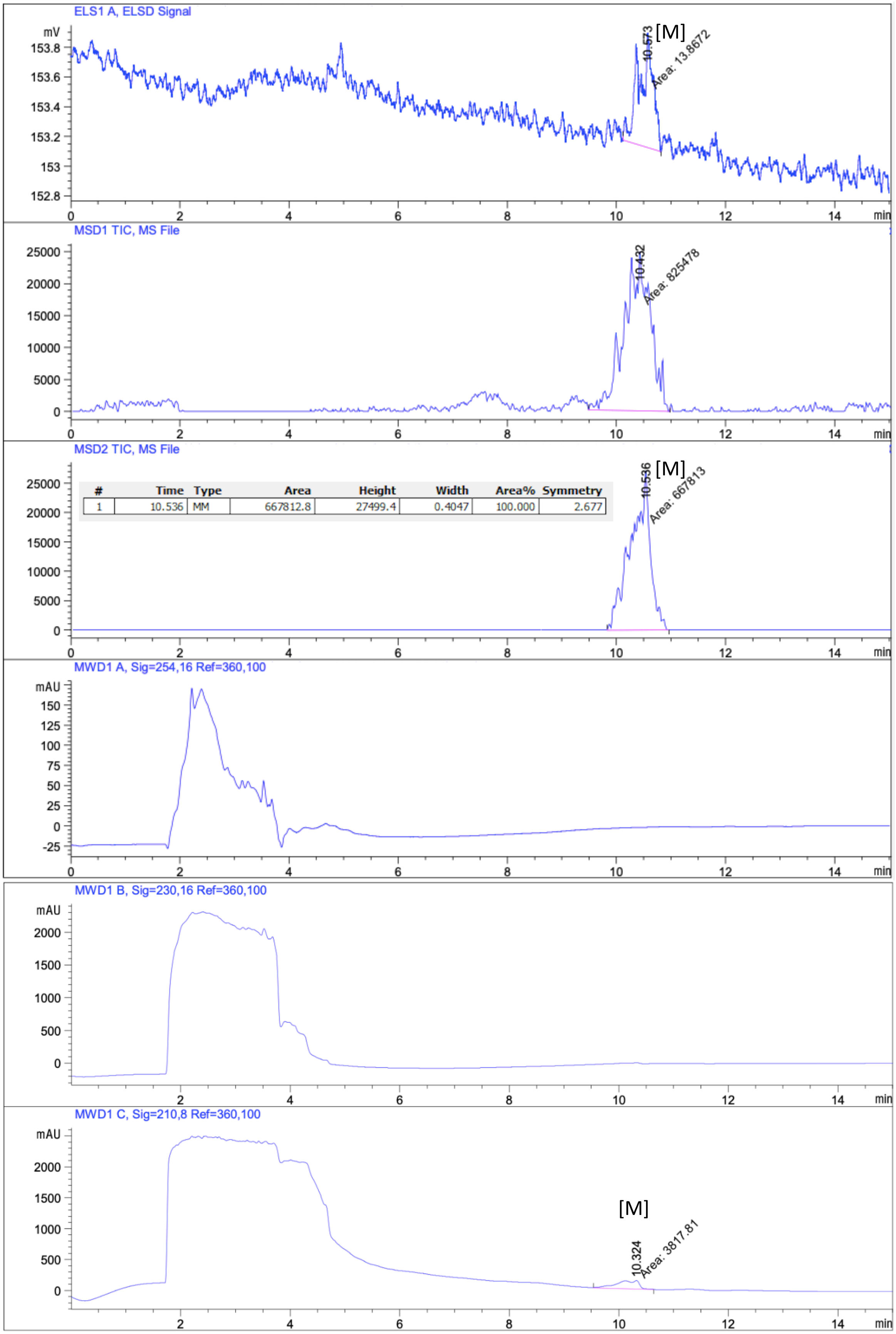

ND = not detected, [M] = macrocycle

Note: all solutions in 100% DMSO, 1-6 min = solvent front

Traces listed in order: ELSD, MSD1 (300-1000 m/z), MSD2 (700-2000 m/z), UV (254, 230, and 210 nm)

Analog 5.3

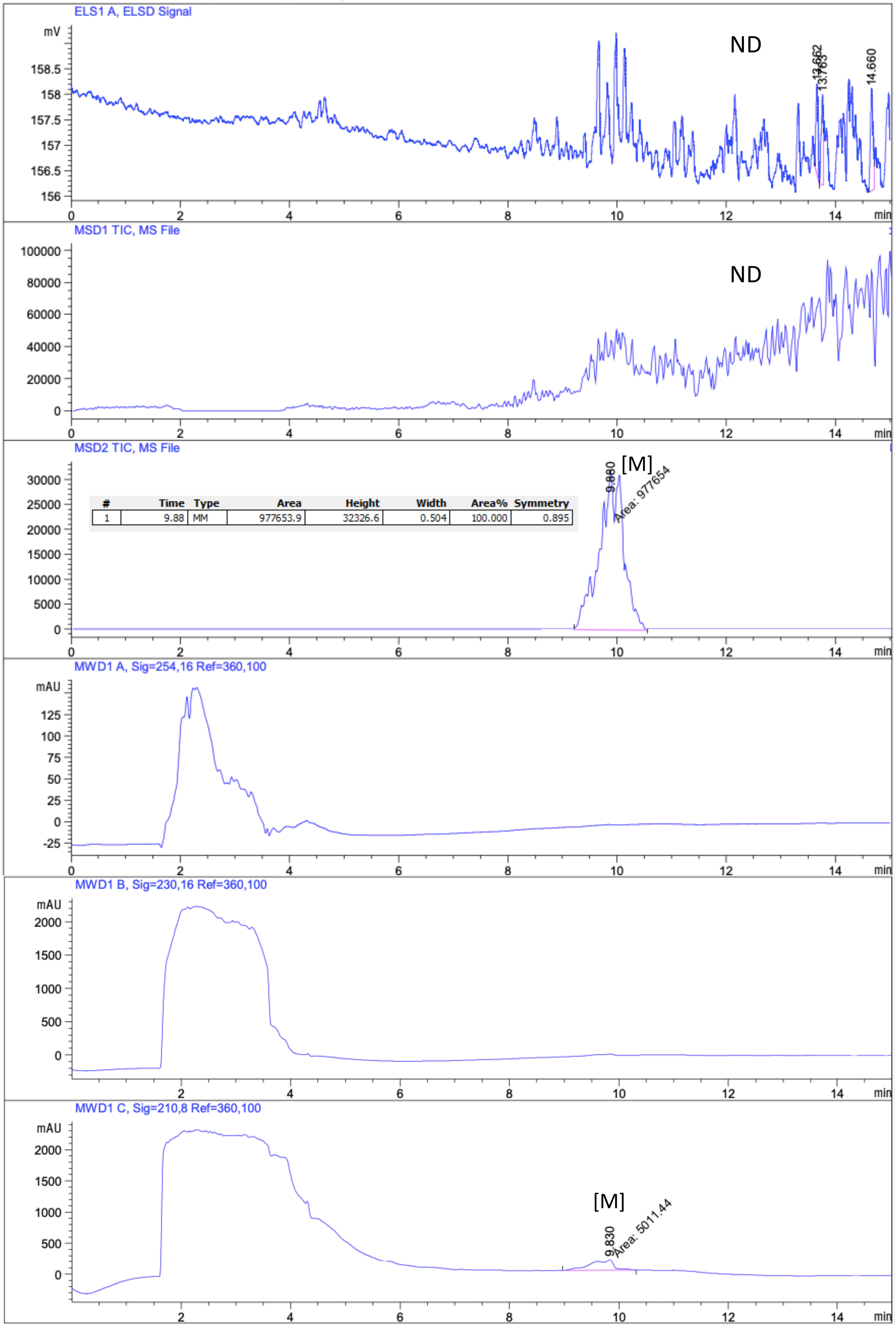

#	Time	Type	Area	Height	Width	Area%	Symmetry
1	9.88	MM	977653.9	32326.6	0.504	100.000	0.895

ND = not detected, [M] = macrocycle
Note: all solutions in 100% DMSO, 1-6 min = solvent front
Traces listed in order: ELSD, MSD1 (300-1000 m/z), MSD2 (700-2000 m/z), UV (254, 230, and 210 nm)

Analog 5.4

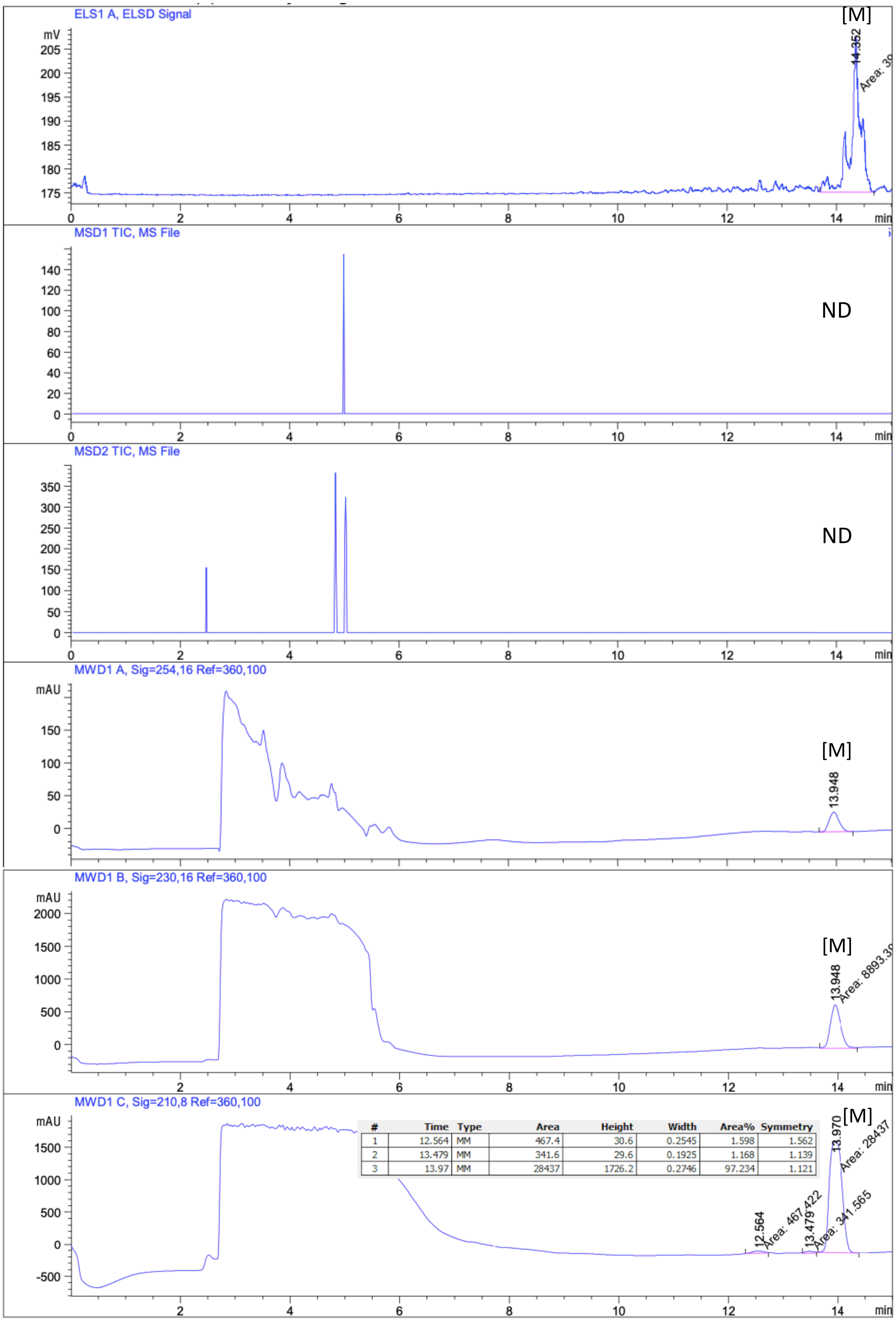

#	Time	Type	Area	Height	Width	Area%	Symmetry
1	12.564	MM	467.4	30.6	0.2545	1.598	1.562
2	13.479	MM	341.6	29.6	0.1925	1.168	1.139
3	13.97	MM	28437	1726.2	0.2746	97.234	1.121

ND = not detected, [M] = macrocycle

Note: all solutions in 100% DMSO, 1-6 min = solvent front

Traces listed in order: ELSD, MSD1 (300-1000 m/z), MSD2 (700-2000 m/z), UV (254, 230, and 210 nm)

Analog 5.5

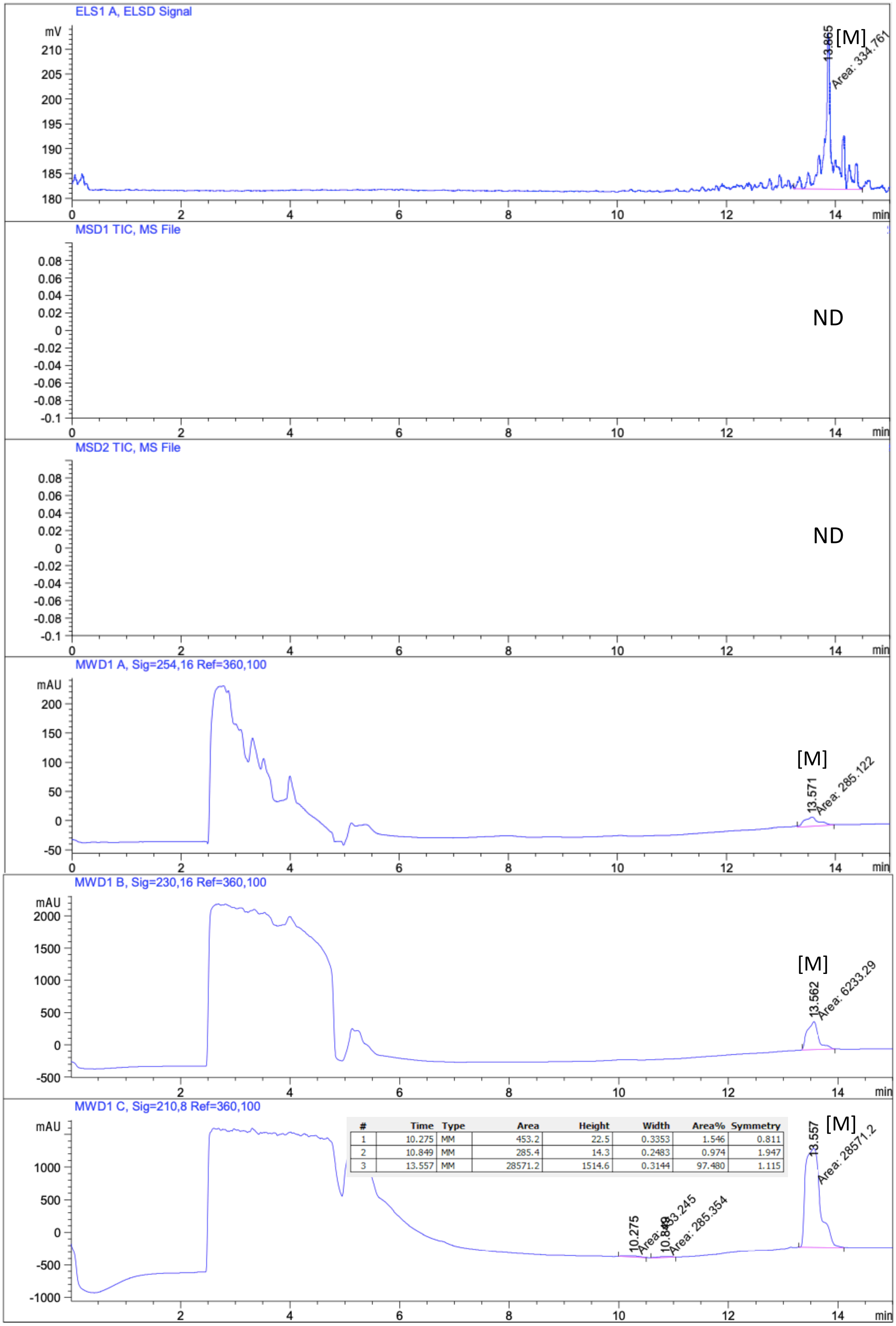

#	Time	Type	Area	Height	Width	Area%	Symmetry
1	10.275	MM	453.2	22.5	0.3353	1.546	0.811
2	10.849	MM	285.4	14.3	0.2483	0.974	1.947
3	13.557	MM	28571.2	1514.6	0.3144	97.480	1.115

ND = not detected, [M] = macrocycle

Note: all solutions in 100% DMSO, 1-6 min = solvent front

Traces listed in order: ELSD, MSD1 (300-1000 m/z), MSD2 (700-2000 m/z), UV (254, 230, and 210 nm)
